# Cocaine induces differential circular RNA expression in striatum

**DOI:** 10.1038/s41398-019-0527-1

**Published:** 2019-08-21

**Authors:** Qian Bu, Hailei Long, Xue Shao, Hui Gu, Jueying Kong, Li Luo, Bin Liu, Wei Guo, Hongbo Wang, Jingwei Tian, Yinglan Zhao, Xiaobo Cen

**Affiliations:** 1 National Chengdu Center for Safety Evaluation of Drugs, State Key Lab of Biotherapy/Collaborative Innovation Center of Biotherapy, West China Hospital, West China Medical School, Sichuan University, Chengdu, 610041 China; 2 Healthy Food Evaluation Research Center, Department of Food Science and Technology, College of Light Industry, Textile and Food Engineering, Sichuan University, Chengdu, 610065 China; 3 School of Pharmacy, Key Laboratory of Molecular Pharmacology and Drug Evaluation, Ministry of Education, Collaborative Innovation Center of Advanced Drug Delivery System and Biotech Drugs in Universities of Shandong, Yantai University, Yantai, 264005 China

**Keywords:** Epigenetics and behaviour, Molecular neuroscience

## Abstract

Circular RNA (circRNA), a novel type of endogenous non-coding RNA, plays natural miRNA sponge effect that represses the activities of corresponding miRNAs through binding with them, thus modulating transcriptional expression of genes. Recent studies indicate that circRNAs are significantly enriched in the brain and some of them are derived from synaptic protein-coding genes. In addition, miRNAs are involved in synaptic plasticity, memory formation, and cocaine addiction. However, the role of circRNAs in cocaine reward is unclear. This study aimed to investigate the expression profile of striatal circRNAs in the mice after cocaine self-administration. By using circRNA microarray analysis, we observed that 90 striatal circRNAs were differentially expressed in cocaine self-administering mice, of which 18 circRNAs were up-regulated and 72 down-regulated. Six circRNAs were selected randomly for validation by using quantitative reverse transcription-PCR, and their expression levels showed consistency with microarray analysis. We backward predicted the circRNAs and their binding sites of miRNAs associated with neuroplasticity. In functional validation test, mmu_circRNA_002381 may modulate the transcription of certain genes associated with neuroplasticity, such as *limk1* and *bdnf*. Taken together, circRNAs may participate in cocaine behavioral effect via interacting with miRNAs. Our findings reveal a potential role of circRNAs in cocaine effect.

## Introduction

It has been known that cocaine epigenetically alters the expression of genes and miRNAs through histone tail or DNA modification in brain’s reward circuitry^1–3^. Striatal miRNA plays a vital role in memory, neuroplasticity, and rewarding effect^3,4^. For example, striatal miR-212 represses the compulsive drug-taking behavior, and modulates the neuroplasticity to cocaine addiction^5–7^. Through interplaying with striatal miR-212, MeCP2 regulates the expression of brain-derived neurotrophic factor (BDNF) and cocaine intake^6^.

CircRNA is a novel type of non-coding RNA, which is involved in various biological functions^8^. CircRNAs are typically generated through a process of back-splicing from exons and a covalent bond linking the 3′- and 5′-end^9,10^. CircRNAs show a typical feature of RNase R resistant, because they do not have 3′- and 5′-terminals. Studies have shown that circRNAs act as competitive endogenous RNAs to sponge mRNAs^11^. Moreover, circRNA expression is concentrated especially in mammalian brain with significant differences compared to other tissues, suggesting its role in central nervous system^12,13^. Previous study found that many neuronal circRNAs are derived from synaptic gene locus and and expression levels of several circRNAs change with the formation of synapse as well as neuroplasticity^14^. In a word, neural circRNAs are derived from synaptic protein-coding genes and modulated by neuroplasticity and neuronal differentiation.

The expression profiles of circRNAs, miRNAs, and lncRNAs are regulated delicately to maintain cellular homeostasis. Evidence shows that circRNAs may play a role in various neurological disorders^15^. For example, CDR1as, a circRNA for miR-7 (CiRS-7) containing 63 selectively conserved miR-7 target sites, represses miR-7 activity and up-regulates the expression of targeted genes in Alzheimer’s or Parkinson’s disease^16,17^. Furthermore, loss-of-function CDR1as causes miR-7 and miR-671 deregulation and affects the function of mammalian brain^18,19^. However, the role of circRNAs in drug addiction is largely unknown.

Drug self-administration is commonly used to assess the reinforcing effects of a drug, which is critical for the initiation and maintenance of drug-seeking behavior. Striatum is a crucial component of brain reward circuitry^20^, which is involved in cue-induced reinstatement, depending on the conditions during reinstatement and the amount of drug exposure^21^. In this study, we aimed to investigate the potential role of striatal circRNAs in cocaine reward. We find that cocaine profoundly modifies the expression profiles of striatal circRNAs, which may reveal a novel molecular mechanism underlying the neuropsychopharmacological effect of cocaine.

## Materials and methods

### Animals and drugs

Eight-week-old male C57BL/6 mice (Vital River Laboratory Animal Technology Co. Ltd, Beijing, China) were used in all the studies. Mice were individually housed in a room with temperature- and humidity-control system. Food and water were available *ad libitum*, and lights operated on a 12 h/12 h cycle (on at 7:30 P.M.). The mice were allowed 7 days to acclimate to the environment before inducing anesthesia and implanting indwelling jugular catheters. All protocols in this study followed the guidelines established by the Experimental Animal Ethics Committee of Sichuan University (Chengdu, China). Cocaine-HCl was provided from National Institute for the Control of Pharmaceutical and Biological Products (Beijing, China). Cocaine was dissolved in 0.9% saline (1.25 mg/ml) for intravenous infusion and 1.5 mg/ml for intraperitoneal (i.p.) injection.

### Surgery for catheter implantation

Briefly, before surgical implantation of a jugular vein catheter, each mouse was anesthetized with sodium pentobarbital (50 mg/kg, i.p.)^22^. After surgery the mice were recovered for 1 week, and heparinized physiological saline (25 U/ml heparin) was used to flush the catheters once daily. To assess catheter patency during the period of cocaine self-administration, each catheter was injected 0.1 ml of brevital (1%), and loss of muscle tone within 5 s after injection indicated a patent catheter.

### Cocaine self-administration procedures

Self-administration procedures were conducted as previously described^23,24^. Briefly, mice were trained to perform an operant response (poking nose in a hole) to receive an intravenous infusion of cocaine or saline. Training continued until the behavior was well established. Seven days after surgery, mice were randomly divided into two groups. The mice in cocaine group (1.25 mg/ml cocaine), and control group (0.9% sodium chloride) were subjected to daily 2 h sessions of cocaine/saline self-administration, respectively, in which one response on the active nose-poke portal yielded one intravenous cocaine/saline infusion (FR1, 0.75 mg/kg/infusion, delivered as unit dose depending on the weight of the mice over a 2-s time period), paired with a 2-s cue light and 20-s timeout period following a nose-poke into the active portal. The inactive portal yielded no consequence. Mice in cocaine-treated group were received continually a cocaine self-administration schedule until they exhibited a stable response (10–12 d). The number of active lever presses for three consecutive days was <10% variation.

### Locomotor activity

Locomotor activity test was conducted as described previously^25^. Locomotor activity sessions were conducted once daily. Each mouse was placed in a locomotor activity chamber followed by cocaine (20 mg/kg, i.p.) or saline injection, and the locomotor activity was measured for 30 min. The chambers were black acrylic boxes (40.64 cm × 40.64 cm × 31 cm) that were equipped with a camera located above the boxes. Automated tracking was performed using the EthoVision 7.0 software (EthoVision 7.0; Noldus Information Technology, Leesburg, VA, USA).

### Tissue isolation

At the end of behavioral testing, mice were sacrificed within 2 h by rapid decapitation. The dorsal striatum was removed from the brain, snap frozen in liquid nitrogen, and stored in RNAlater solution (Beyotime Institute of Biotechnology) at −80 °C until assay.

### Striatal RNA isolation for microarray

Following manufacturer’s instructions, total RNA was isolated by using the RNeasy kit (Qiagen). RNA concentration of each sample was measured by NanoDrop ND-1000 instrument (Nanodrop Technologies, Wilmington, DE). The purity and integrity of the RNA were detected by formaldehyde denaturing agarose gel electrophoresis.

### Sample labeling and array hybridization

Based on Arraystar’s standard protocols, sample labeling and array hybridization were performed. Briefly, total RNA was treated with RNAase R to remove linear RNAs. Through a random priming method, each sample was amplified and transcribed into fluorescent complementary RNA (Arraystar Super RNA Labeling Kit), followed by purification of labeled cRNAs by using RNeasy Mini Kit (Qiagen). The concentrations of labeled cRNAs (pmol Cy3/μg cRNA) were analyzed by NanoDrop ND-1000, and hybridized onto the Arraystar Mouse circRNA Array (6′7K, Arraystar). The hybridized arrays were washed, fixed, and scanned using Axon GenePix 4000B microarray scanner (Molecular Devices, Inc.). KangChen Bio-Tech (Shanghai, China) performed the microarray hybridization and collection of data.

### Microarray analysis

The GenePix Pro 6.0 software (Axon) was utilized for grid alignment and data extraction from the scanned images. Quantile normalization and subsequent data analysis were conducted by using the R software package. After normalization the distributions of expression values for the eight samples are indicated in Box-plot. Through performing quantile normalization, low intensity signal was filtered. The circRNAs with flag expression two times more than the background were selected for further analyses. By using Volcano Plot and Scatter-Plot filtering, the expression levels of circRNAs were analyzed for statistical significance. Differentially expressed circRNAs between the two groups were identified through Fold Change and t-test filtering. CircRNAs which show fold changes ≥ 2.0 and *p* ≤ 0.05 are considered as significant. Hierarchical Clustering was conducted to show the significantly different expression pattern of circRNAs among brain samples.

### Real-time quantitative PCR

RNA sample of dorsal striatum was utilized to verify microarray data in the initial microarray study, and reverse transcription was conducted (Superscript III Reverse Transcriptase, Invitrogen). ABI PRISM7900 system (Applied Biosystems) was used to quantitate the RNA levels of brain sample by using GAPDH as an endogenous control. Through a log 2 transform the expression ratios (fold change) were obtained. The qRT-PCR primer sequences used for the validation of microarray data were shown in Supplementary Table S1.

### Annotation for circRNA/miRNA interaction

We used Arraystar’s home-made miRNA target prediction software based on TargetScan and miRanda to predict the interation of circRNA/miRNA^26^. The differentially expressed circRNAs between the two groups were annotated with the information of circRNA/miRNA interaction. There were 90 differentially expressed circRNAs with fold-change ≥ 2.0 and *p* ≤ 0.05, including 18 up-regulated and 72 down-regulated circRNAs. They were used to predict their miRNA response elements (MREs). Five MREs with good scores of miRNA support vector regression (mirSVR) for each circRNA were shown. Ultimately, the circRNA/miRNA network for the differentially expressed circRNAs was constructed and visualized using the Cytoscape software (http://www.cytoscape.org/)^27^.

### Backward prediction based on plasticity- and addiction-associated miRNAs

Based upon those plasticity- and addiction-associated miRNAs reported in literatures, we backward predicted the possible interactions of each miRNA with its circRNAs. Putative interactions between the sequenced miRNAs and the predicted circRNAs were evaluated by using miRanda and TargetScan, investigating both perfect and imperfect seed matching. To this end, the circRNA BED output file were converted into FASTA format DNA sequences. Then a hit between each miRNA and a target circRNA were considered for a miRanda structure scores of 1400 or higher^26^, corresponding to a perfect or imperfect seed match.

### RNA interference and transfection assay

Small interfering RNAs (siRNAs) targeting the back-splice junction of mmu_circRNA_002381 (si-mmu_circRNA_002381) was designed and synthesized by RiboBio Corporation (Guangzhou, China). For si-mmu_circRNA_002381, the sense strand sequence of functional siRNA was 5′CUCAUGCUUAGGCUUGAUU dTdT3′, and the antisense strand sequence was 3′dTdT GAGUACGAAUCCGAACUAA5′. N2a cells, a mouse neuroblastoma cell line, were transfected by lipofectamine 3000 (Invitrogen). Briefly, the cells were cultured with DMEM in a 96-well plate. Before transfection, a medium without antibiotics was added to the culture. RNAi lipofectamine 3000 complex was then added to each well. The cells were cultured in DMEM medium at 37 °C for 48 h. The transfection efficiency was determined using Cy3-tagged NS siRNA under a fluorescence microscope.

### Protein extraction and western blot

Transfected cells were solubilized with RIPA lysis buffer (Beyotime Biotechnology) containing protease inhibitors (Roche). The protein concentration was determined by the Bradford assay kit (Bio-Rad). The protein extracts were separated with 6–12% SDS-PAGE gels and transferred to polyvinylidene difluoride (PVDF) membranes (Millipore). The membranes were then blocked with 5% non-fat milk in TBST for 1 h at room temperature, followed by incubation with the primary antibodies overnight at 4 °C. Subsequently, the membranes were washed three times with TBST and the blots were incubated with secondary antibodies conjugated to horseradish peroxidase (ZSGB-BIO, Beijing, China) for 1 h at room temperature. Immunoreactivity was visualized using ECL Western blotting detection reagents and then analyzed through scanning densitometry. The following antibodies were used: LIMK1 (ab81046, abcam), BDNF(ab108319, abcam), SIRT1 (#8469, Cell Signaling), CREB (#9197, Cell Signaling), GAPDH (#5174, Cell Signaling).

### Data analysis

Two-way ANOVA with a repeated measures over day was used to analyze the behavioral data. Tukey’s post hoc comparison was performed to compare gene expressions of the two groups. Principal component analysis (PCA) score plots were obtained using the normalized fluorescence intensity data in SIMCA-P11.0 (Umetrics, AB). All statistics of the results are given as means ± SEM of more than three independent experiments. Differences between the two groups were analyzed at two-tailed Student’s t-test with α = 0.05. Results were considered statistically significant when *P* < 0.05.

## Results

### Cocaine self-administration modifies circRNA profile in dorsal striatum

**Fig. 1:**
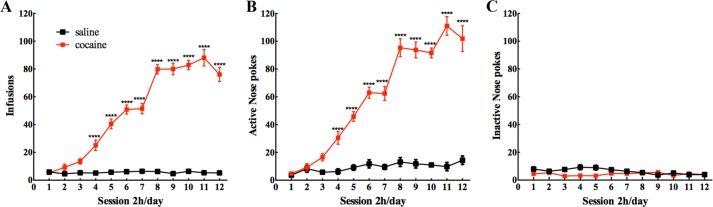
Cocaine self-administration in mice. **a** The number of infusions (Two-way repeated measures ANOVA, interaction effect *F* = 70.33, *p* < 0.0001; drug effect *F* = 69.09, *p* < 0.0001; session effect *F* = 1742, *p* < 0.0001). **b** Active nose pokes (Two-way repeated measures ANOVA, interaction effect *F* = 56.70, *p* < 0.0001; drug effect *F* = 42.37, *p* < 0.0001; session effect *F* = 1369, *p* < 0.0001) and (**c**) inactive nose pokes (Two-way repeated measures ANOVA, interaction effect *F* **=** 2.557, *p* = 0.0058; drug effect *F* = 0.9250, *p* = 0.05258; session effect *F* = 19.51, *p* < 0.0001) are shown respectively for mice that acquired and maintained cocaine (0.75 mg/kg/infusion) or saline self-administration. *n* = 4/group. Data are presented as Mean ± SEM

Self-administration, one of the best animal models to mimic human addiction, enables the measurement of voluntary cocaine taking and cocaine seeking, from which the degree of cocaine craving can be inferred. After 12 sessions, mice in two groups showed stable nose-poking rates during the last 3 days of self-administration with less than a 10% difference in their daily intake of cocaine. The mean number of cocaine/saline infusions (Mean ± SEM) per day during the last 3 days of self-administration in cocaine group and control group was 82 ± 6.0 and 6 ± 0.6 (Fig. 1a), respectively. Cocaine-treated mice significantly poked more on the active portals (Fig. 1b) than on the inactive portals (Fig. 1c) from the fourth day to the twelfth day (*P* < 0.001). These data showed that we successfully established cocaine self-administration behaviors in mice.

The expression profiles of circRNAs were analyzed immediately after the last session of self-administration with high throughput microarray (Arraystar Mouse CircRNA Microarray, Kangchen Corporation, Shanghai, China.). Quality control of RNA isolation and labeling efficiency including RNA quantification, RNA integrity, gDNA contamination, labeling efficiency, and array image of each sample were displayed in Supplementary Fig. 1. A total of 795 circRNAs were identified above background levels (representing 44.2% of the total 1797 circRNAs detected), 11.3% (90 of 795) of which were significantly differentially expressed. The expression was considered to be significantly altered through a combination of statistical significance (*p* ≤ 0.05 and fold change ≥ 2.0).

**Fig. 2:**
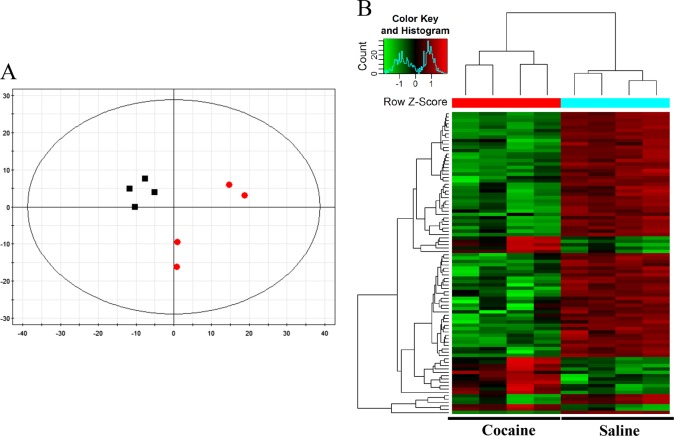
CircRNA expression profile in dorsal striatum of cocaine self-administrated mice. **a** PCA reveals separation between groups for 1797 circRNAs. The red represents the cocaine self-administration (*n* = 4) and the black represents the control subjects (*n* = 4). **b** Heat map and hierarchical cluster analysis of the differentiall expressed circRNAs after cocaine self-administration. Hierarchical clustering analysis was conducted to compare the differentially expressed circRNAs between the two groups (*n* = 4/group). Heat map shows the expression level of a panel of cocaine group relative to control. Each column represents the indicated one sample. Each row represents circRNAs. The color change indicates relative change according to the scale shown; red represents positive fold change and green represents negative fold change

Based on the initial circRNAs set, we used PCA to perform an unsupervised examination to discriminate cocaine-administrated group from control group. As shown in PCA plot, cocaine-administrated mice were clearly separated from saline-treated mice (Fig. 2a), suggesting that striatal circRNA expression was affected by cocaine self-administration. These selected circRNAs were confirmed again with clustered image map and heat map (Fig. 2b). Clustered image map showed the relative change of circRNA expression between cocaine and control groups. The data clearly illustrated circRNAs expressed in cocaine-administrated mice was different from that in saline-treated mice. Heat map represented the expression values of a panel of cocaine group relative to control group.

### Identification of differentially expressed circRNA profiles-induced by cocaine

**Fig. 3:**
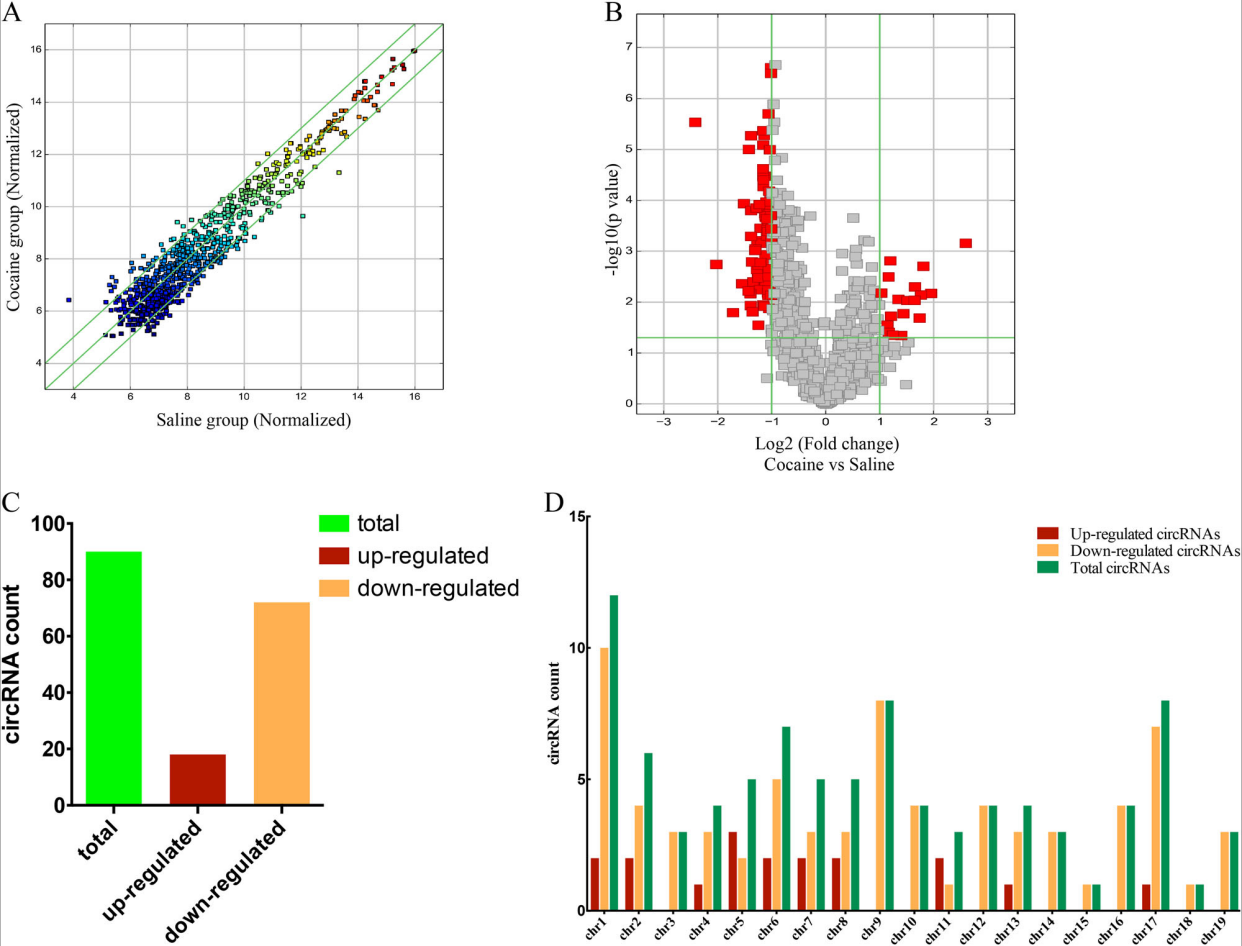
Expression levels of striatal circRNAs induced by cocaine. **a** The Scatter-Plot is used to analyze the variation of circRNA expression between samples or groups. The values of *X-* and *Y*-axis in the Scatter-Plot are normalized (log2 scaled). The green lines represent fold change lines. CircRNAs above the top green line and below the bottom green line indicate more than 2.0 fold change of circRNAs between cocaine group and saline group. **b** Volcano plot indicates the fold change (two-fold up and down), respectively [Log2 (fold change), *x*-axis] and *p* = 0.05 [Log10 (*p* value), *y*-axis] for circRNAs. The red point in the plot represents the differentially expressed circRNA with statistical significance. **c** Considering the fold change and *p*-values, 18 circRNAs were up-regulated and 72 were down-regulated. **d** The distribution of differentially expressed circRNAs in chromosomes

By using volcano plot filtering, differentially changed circRNAs with statistical significance between the two groups were identified. We utilized Scatter Plot to analyze circRNA expression variation between cocaine group and control group (Fig. 3a). The Volcano plot showed the alteration of fold change of two-fold up and down [Log2 (fold change), *x*-axis], respectively, and *p* = 0.05 [Log10 (*p* value), *y*-axis] for circRNA (Fig. 3b). As a result, 90 circRNAs were identified to be differentially modulated by fold change ≥ 2.0 and *P* ≤ 0.05. Among them, 72 circRNAs were down-regulated whereas 18 circRNAs were up-regulated (Fig. 3c). The distribution of the differentially expressed circRNAs on the chromosomes is shown in Fig. 3d. List of circRNAs with significant differences between two groups was displayed in Supplementary Table S2.

To validate the microarray results, we randomly selected six differentially expressed circRNAs (fold-change ≥ 2.0; *P* ≤ 0.05), including two up-regulated circRNAs and four down-regulated circRNAs, and validated their expression levels by qRT-qPCR analysis. The results showed that two circRNAs (mmu_circRNA_002179, mmu_circRNA_002381) were overexpressed, while four circRNAs (mmu_circRNA_003585, mmu_circRNA_012342, mmu_circRNA_014222, mmu_circRNA_017361) were under-expressed. These data showed that the result of circRNAs expression from microarray analysis was well consistent with that of qRT-PCR test (Supplementary Fig. 2).

### Construction of the circRNA/miRNA interaction network

**Fig. 4:**
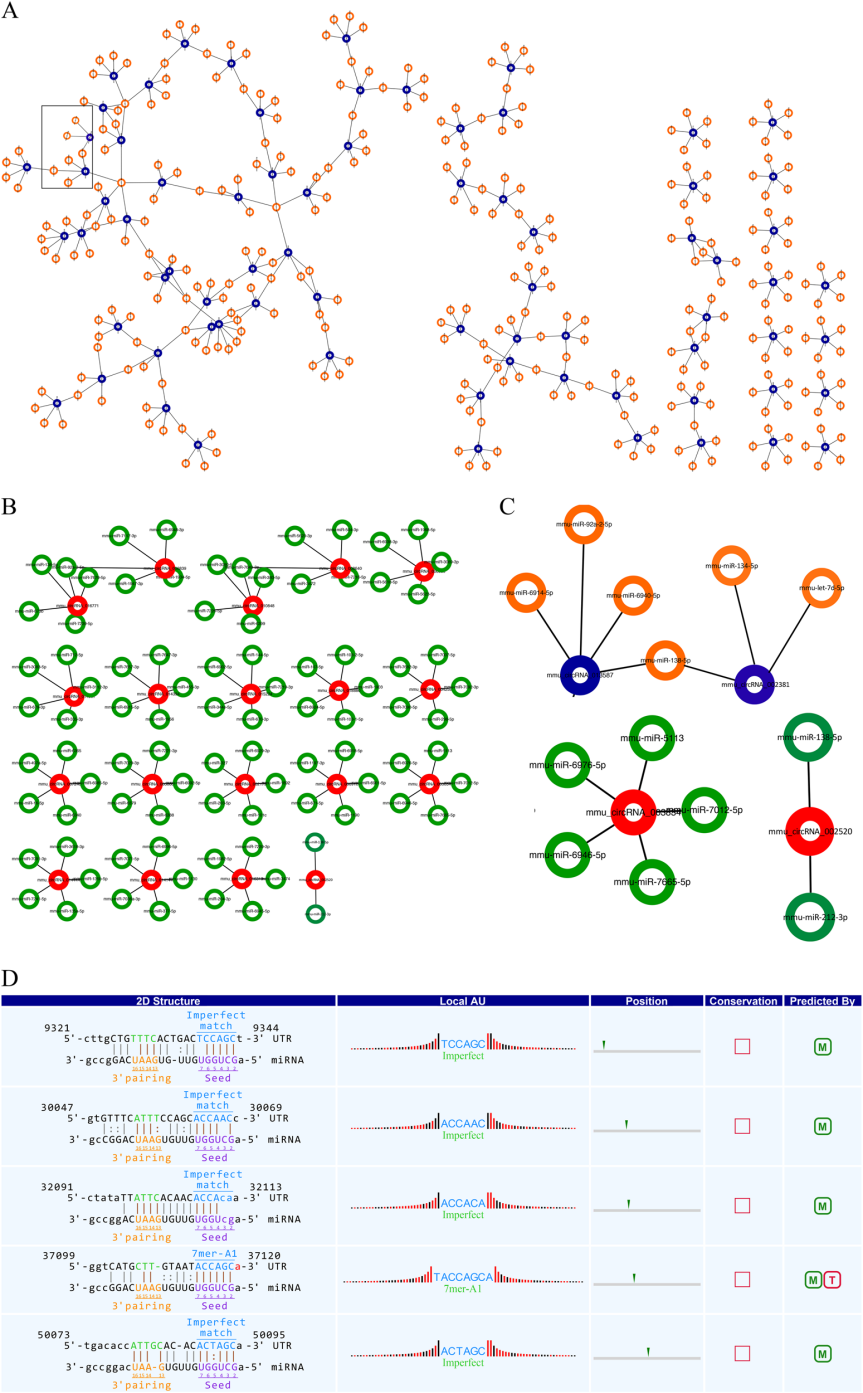
Cytoscape delineates the network of interaction between circRNA and miRNA. **a** The network consists of down-regulated circRNAs (blue nodes) and their target miRNAs (orange nodes). **b** The network contains up-regulated circRNAs (red nodes) and their target miRNAs (green nodes). **c** A magnified network of mmu_circRNA_002381, mmu_circRNA_013587, mmu_circRNA_003834 and mmu_circRNA_002520 and their target miRNAs is presented. **d** The detailed annotation for mmu-miR-138-5p/mmu_circRNA_002381 interaction

Based on TargetScan and miRanda database, we utilized a conserved seed-matching sequence and a software (Arraystar) for miRNA target prediction to theoretically predict the interaction between circRNAs and their target miRNAs^28^. All the differentially expressed circRNAs were predicted according to the complementary miRNA matching sequence. Each circRNA and its potential top five complementary binding miRNAs were shown (Supplementary Table S2). Furthermore, we used mirSVR algorithm to score and rank the efficiency of the predicted miRNA targets. The differentially expressed circRNAs were annotated with detailed information of circRNA/miRNA interactions. We used Cytoscape to delineat the entire network of circRNA/miRNA interaction (Fig. 4a–c). For example, the detailed annotation for mmu_circRNA_002381/mmu-miR-138-5p interaction was displayed in Fig. 4d. Other annotations for circRNA/miRNA interaction were displayed in Supplementary Figs. 3–5.

### Backward prediction based on plasticity- and addiction-associated miRNAs

**Fig. 5:**
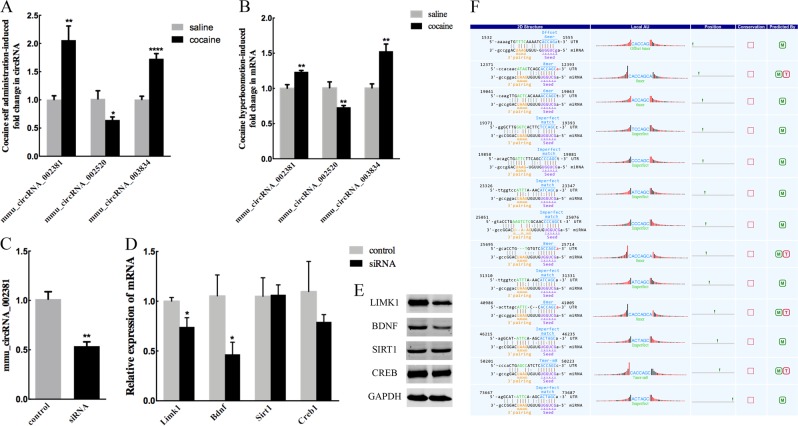
Knockdown of mmu_circRNA_002381 inhibits the expressions of certain genes. **a** Cocaine self-administration induces mmu_circRNA_002381 and mmu_circRNA_003834 up-regulation, while mmu_circRNA_002520 was down-regulated. **b** Cocaine hyperlocomotor behavior induces the same modification as those in mmu_circRNA_002381, mmu_circRNA_002520 and mmu_circRNA_003834. **c** The expression level of mmu_circRNA_002381 was detected after si-mmu_circRNA_002381 treatment in N2a cells. **d**, **e** The mRNA and protein expressions of neuroplasticity genes, such as *limk1, bdnf, sirt1*, and *creb1* were detected following knockdown of mmu_circRNA_002381 using si-mmu_circRNA_002381. **f** Detailed annotation for mmu-miR-138-5p/mmu_circRNA_003834 interaction is displayed. *P < 0.05, **P < 0.01, ***P < 0.001

Emerging evidence show that striatal miRNAs play important roles in neuroplasticity and memory. Moreover, circRNAs consist of shared MREs for binding miRNA. What if we backward predict the circRNAs based on those plasticity- and addiction-associated miRNAs? Striatal miRNA-212 controls cocaine intake through cyclic AMP response element-binding (CREB) signaling; moreover, MeCP2 controls BDNF and cocaine intake through homeostatic interactions with miRNA-212^5,6^. Cocaine-induced conditioned place preference was regulated by miR-124, let-7d and miR-181a in the nucleus accumbens^29,30^. MiR-134 modulates neuroplasticity and memory through SIRT1^31^. MiR-128 controls neuronal excitability and motor behavior in mice^32^. Interestingly, backward prediction based on those aforementioned plasticity- and addiction-associated miRNAs (Supplementary Table S3) revealed that mmu_circRNA_002381 contained 10 binding sites for miR-138, 10 binding sites for let-7d and 15 binding sites for miR-134, while mmu_circRNA_002520 contained 33 miR-138 binding sites and 29 miR-212 target sites, respectively. More interestingly, mmu_circRNA_003834 contained 13 miR-138 binding sites, which was significantly up-regulated in cocaine self-administration group. The associations between these miRNAs and their target circRNAs were also annotated in Supplementary Fig. 6. We found that cocaine self-administration up-regulated mmu_circRNA_002381 and mmu_circRNA_003834 while down-regulated mmu_circRNA_002520 (Fig. 5a). To confirm these changes in cocaine-treated striatum, we continued to detect the expression levels of these three circRNAs in the cocaine-induced locomotor activity model. Importantly, we found that cocaine induced the same alteration as those in mmu_circRNA_002381, mmu_circRNA_002520 and mmu_circRNA_003834 (Fig. 5b).

### RNA interference and transfection assay

To explore the potential role of mmu_circRNA_002381 in gene expression, we used mmu_circRNA_002381-specific siRNA to knockdown mmu_circRNA_002381 and then detected the expression level of genes associated with neural plasticity and drug addiction in N2a cells in vitro. We found that the specific siRNA clearly down-regulated the expression of mmu_circRNA_002381 (Fig. 5c); moreover, the mRNA and protein expressions of the genes measured were down-regulated, such as *limk1* and *bdnf* (Fig. 5d, e). These results indicated that mmu_circRNA_002381 might play a role in cocaine reward, at least in part, via acting as miRNAs sponges. Mmu-miR-138-5p was also observed to sponge to mmu_circRNA_003834 with 13 potential binding sites. The detailed annotation for mmu-miR-138-5p/mmu_circRNA_003834 interaction was displayed in Fig. 5f.

## Discussion

Cocaine induces profound alterations in the expression of genes in brain reward systems, and cocaine dependence is commonly thought to be a disorder of neuroplasticity. In these pathological processes, miRNAs play important roles in regulating the complex actions of cocaine in brain reward circuits and neuroadaptation associated with addiction. However, emerging evidence shows the interactions between circRNAs and miRNAs as well as their roles in various physiological and pathological processes, particularly through sequestering endogenous RNA mechanism. In this study, we used circRNA microarray and bioinformatic analysis to investigate the differentially expressed striatal circRNAs after cocaine self-administration. The circRNA expression profiles revealed that 18 circRNAs were significantly up-regulated and 72 circRNAs down-regulated in the dorsal striatum of cocaine self-administering mice. We annotated the circRNA/miRNA interaction for the differentially expressed circRNAs, and performed a detailed annotation for representing the binding sites of circRNAs and their conserved MREs. Our results show that circRNAs might be involved in the processes of cocaine reward via acting as miRNA sponges (Supplementary Fig. 7). Nevertheless, circRNA/miRNA interactions and their roles in cocaine effect need to be experimentally validated in future research.

### CircRNAs, acting as miRNA sponges, may be involved in cocaine effect

CircRNAs have many miRNA binding sites to bind competitively to miRNAs. Thus, circRNAs, usually acting as miRNA sponges, may alleviate the inhibitory effects of miRNAs on target molecules, thereby regulating gene expression. Here, we discuss the potential circRNAs involved in cocaine effect and the underlying mechanism. Mmu_circRNA_017196 was down-regulated in the dorsal striatum of cocaine-treated mice. Based on the analysis of circRNA/miRNA network, we found that mmu_circRNA_017196 interacts with miR-128-3p. Interestingly, it has been known that miR-128 controls the motor activity of mice by weakening the expression of ion channels and signaling molecules of extracellular signal-regulated kinase network that modulates neuronal excitability^32^. Moreover, miR-128 over-expression weakens neuronal responsiveness and motor activity of mice. Therefore, we assume that mmu_circRNA_017196 down-regulation induced by cocaine may be involved in cocaine effect through up-regulating miR-128. Further studies are needed to address this point. Mmu_circRNA_013587 was down-regulated in cocaine-treated mice. Through the analysis of circRNA/miRNA network, we found that mmu_circRNA_013587 interacts with miR-138, which directly targets depalmitoylation enzyme acyl-protein-thioesterase 1, thus regulating dendritic spine size^33,34^. As MiR-138 is locally enriched at synaptic sites, we assume that it may participate in synaptic mechanisms. In addition, *sirt1* has recently been identified to be a target of miR-138^35^, and is associated with synaptic plasticity modulation and memory formation^31^. Therefore, it is conceivable that mmu_circRNA_013587 might be involved in cocaine reward through modulating miR-138 expression.

Striatal mmu_circRNA_005522 was down-regulated after cocaine self-administration, and circRNA/miRNA network revealed that circRNA_005522 interacts with miR-135a-5p and miR-135b-5p. MiR-135 contributes to the spine remodeling in hippocampal neurons through modulating the expression of its target gene complexin-1/2^36,37^. In addition, we found that cocaine up-regulated mmu_circRNA_008640, which may modulate dopamine D1 receptor gene (DRD1) expression by interacting with miR-504-3p. It has been known that DRD1 is associated with nicotine dependence, and miR-504 up-regulates DRD1 expression by targeting the DRD1 3′ UTR^38^. Other potential regulatory circRNAs, such as mmu_circRNA_010647, mmu_circRNA_017361, and mmu_circRNA_012180, interact respectively with two miR-8 family members, miR-200c-5p and miR-141-5p, which are down-regulated by cocaine^39^.

In addition, we found that there are still some other circRNAs modified by cocaine, such as mmu_circRNA_017196/miR-188-3p, mmu_circRNA_003585/miR-29b-2-5p, mmu_circRNA_003195/miR-181d-5p, mmu_circRNA_013774/miR-32-3p, mmu_circRNA_001535/miR-219a-5p, and mmu_circRNA_004122/miR-26b-3p. These circRNAs target some neuroplasticity-specific miRNAs, which have been shown to participate in cocaine effects, such as miR-188, miR-29a/b, miR-181, miR-32, and miR-219. Therefore, these circRNAs may also be involved in sequestering miRNAs and blocking their interactions with target mRNAs. For example, miR-188, an activity-regulated miRNA that regulates synaptic plasticity, plays a role in synaptic plasticity by buffering the expression of Nrp-2, which induces the repulsion of neuronal growth cones expressing Nrp-2^40^. MiR-29a/b regulates dendritic spine morphology by directly targeting the mRNA encoding for *arpc3*, a subunit of ARP2/3 actin nucleation complex^41,42^. MiR-181 down-regulation leads to an up-regulation of the excitatory receptor GluA2^40^. Prohormone convertase PC2 (also known as *pcsk2*) indicates the dendritic synthesis and processing. It is targeted by miR-32, which is up-regulated by cocaine^3^. CaMKII family is critical for regulating NMDA signaling that regulates NMDA-R activity, and can be locally translated in dendrites to enhance neuroplasticity^43^. MiR-219 regulates the expression of CaMKII in the brains of mice^44^. Moreover, miR-26b down-regulates BDNF expression^45^.

### MiRNAs targeted by circRNA are closely associated with addiction-relevant genes

In this study, cocaine self-administration induced mmu_circRNA_002381 and mmu_circRNA_003834 up-regulation but mmu_circRNA_002520 down-regulation. Backward prediction based on plasticity- and addiction-associated miRNAs reveals that mmu_circRNA_002381 and mmu_circRNA_003834 contain 10 and 13 binding sites for miR-138, respectively; moreover, mmu_circRNA_002520 contains 33 miR-138 binding sites and 29 miR-212 target sites. In addition, miR-138 mediates the formation of spine structure and is involved in modulation of functional synaptic plasticity^46^. Although the *sirt1* is one of the miR-138 targets^35^, the expression of *sirt1* is no significantly changed by si-mmu_circRNA_002381 treatment (Fig. 5d, e).

Interestingly, in this study, siRNA-mediated mmu_circRNA_002381 down-regulation in N2a cells increased the expressions of *limk1* and *bdnf*, which are the targets of miR-138 (TargetScanMouse). Thinking above together with our findings, we assume that mmu_circRNA_002381 may play a role in cocaine reward via acting as miRNAs sponges.

Except for acting as miRNA sponges, circRNAs may affect neuronal function through various mechanisms. For example, circRNAs interact with proteins and regulate their activities; moreover, circRNAs recruit the components of different protein complexes, just like CDR1as binding with Argonaute protein^47–50^. CircRNAs act as translation templates to encode proteins^51^, thus exerting specific effects and modulating protein–protein interactions. In addition, circRNAs is localized in the cytoplasm and nucleus. Nuclear circRNAs could therefore modulate transcriptional activity of genes in the neurons^52,53^. For instance, PAIP2 up-regulation by circPAIP2 may modulate contextual memory^54^. Further research needs to be conducted to elucidate the potential effect of circRNAs in combining with certain proteins.

It was reported that by using deep RNA profiling, circHomer1 in the dendrites of neurons was found to be significantly up-regulated by development and plasticity^14^. However, we did not detect this circRNA in our microarray analysis. Limited circRNA probes may attribute to this phenomenon because Arraystar Mouse circRNA Array (6 × 7K, Arraystar) had no circHomer1 probe.

In summary, circRNAs which are potentially associated with diseases in Circ2Traits database, such as Parkinson’s disease and Alzheimer’s disease, show the role of circRNAs as miRNA sponges^55^. In this study, we show a modified expression pattern for a large set of striatal circRNAs related to neuroplasticity in cocaine-treated mice, suggesting the role of circRNAs in cocaine addiction. It is necessary to explore the underlying mechanisms by which circRNAs act as miRNA sponges to modulate cocaine effect in the future.

## Supplementary information

Supplementary figure legends

Figure S1 (A) Array images of saline sample and cocaine sample. sal, saline-treated mice; coc, cocaine-treated mice. (B) RNA quantification of quality assurance by NanoDrop ND-1000. (C) RNA integrity and gDNA contamination test by denaturing agarose gel electrophoresis.

Figure S2 (A) Quantitative RT-PCR confirmation for 6 selected circRNAs. (B) The expression of 6 selected circRNAs by microarray between cocaine group and saline group.

Figure S3 The annotations for circRNA/miRNA interaction are displayed in CircRNA-MREs.

Figure S4 The full size of image for the network consists of down-regulated circRNAs (blue nodes) and their target miRNAs (orange nodes).

Figure S5 The full size of image for the the network contains up-regulated circRNAs (red nodes) and their target miRNAs (green nodes).

Figure S6 The associations between the miRNAs and their target circRNAs are annotated in MREs for backward prediction.

Figure S7 The diagram models the mechanism of circRNA in cocaine self-administration.

Table S1 RT-PCR primers used to validate the microarray data.

Table S2 List of the differentially expressed circRNAs.

Table S3 List of circRNAs predicted from miRNAs.

## Data Availability

The datasets for this study can be found in GEO (https://www.ncbi.nlm.nih.gov/geo/query/acc.cgi?acc=GSE112370).
