# Supplementary material for: Cocaine induces differential circular RNA expression in striatum
Source: Transl Psychiatry. 2019 Aug 21;9:199. doi: 10.1038/s41398-019-0527-1 (PMC6704174; doi:10.1038/s41398-019-0527-1)
Supplement: Supplementary file 1 — Supplementary figure legends [file 41398_2019_527_MOESM1_ESM.docx]

**Supplementary figure legends**

**Supplementary Fig.1.** **(A)** Array images of saline sample and cocaine sample. sal, saline-treated mice; coc, cocaine-treated mice. **(B)** RNA quantification of quality assurance by NanoDrop ND-1000. **(C)** RNA integrity and gDNA contamination test by denaturing agarose gel electrophoresis.

**Supplementary Fig.2.** **(A)** Quantitative RT-PCR confirmation for 6 selected circRNAs. **(B)** The expression of 6 selected circRNAs by microarray between cocaine group and saline group.

**Supplementary Fig.3.** The annotations for circRNA/miRNA interaction are displayed in CircRNA-MREs.

**Supplementary Fig.4.** The full size of image for the network consists of down-regulated circRNAs (blue nodes) and their target miRNAs (orange nodes).

**Supplementary Fig.5.** The full size of image for the the network contains up-regulated circRNAs (red nodes) and their target miRNAs (green nodes).

**Supplementary Fig.6.** The associations between the miRNAs and their target circRNAs are annotated in MREs for backward prediction.

**Supplementary Fig.7.** The diagram models the mechanism of circRNA in cocaine self-administration.
