# Supplementary figures and images for: Cocaine induces differential circular RNA expression in striatum
Source: Transl Psychiatry. 2019 Aug 21;9:199. doi: 10.1038/s41398-019-0527-1 (PMC6704174; doi:10.1038/s41398-019-0527-1)

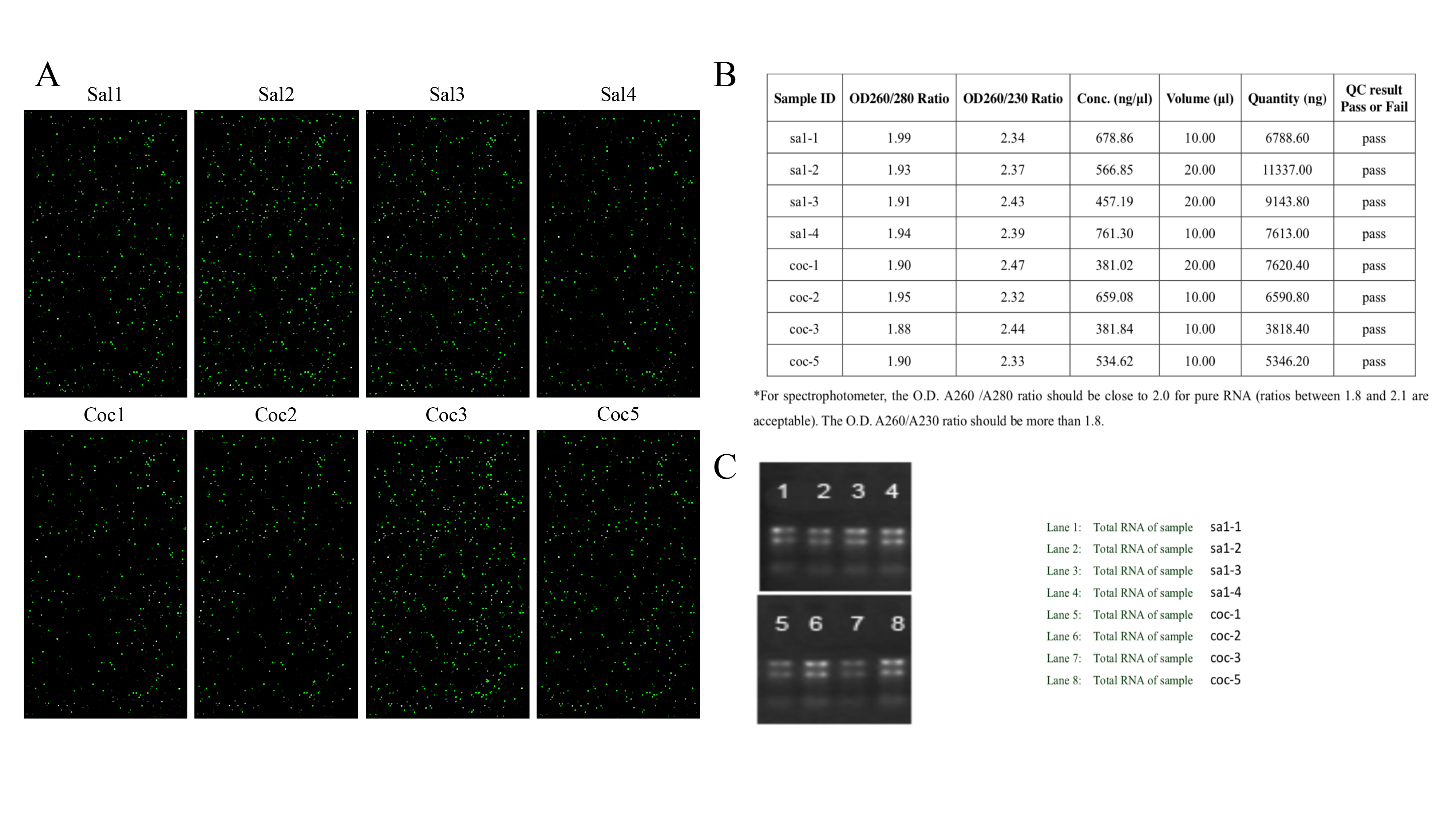

Supplement: Supplementary file 2 — Figure S1 (A) Array images of saline sample and cocaine sample. sal, saline-treated mice; coc, cocaine-treated mice. (B) RNA quantification of quality assurance by NanoDrop ND-1000. (C) RNA integrity and gDNA contamination test by denaturing agarose gel electrophoresis. [file 41398_2019_527_MOESM2_ESM.tif]

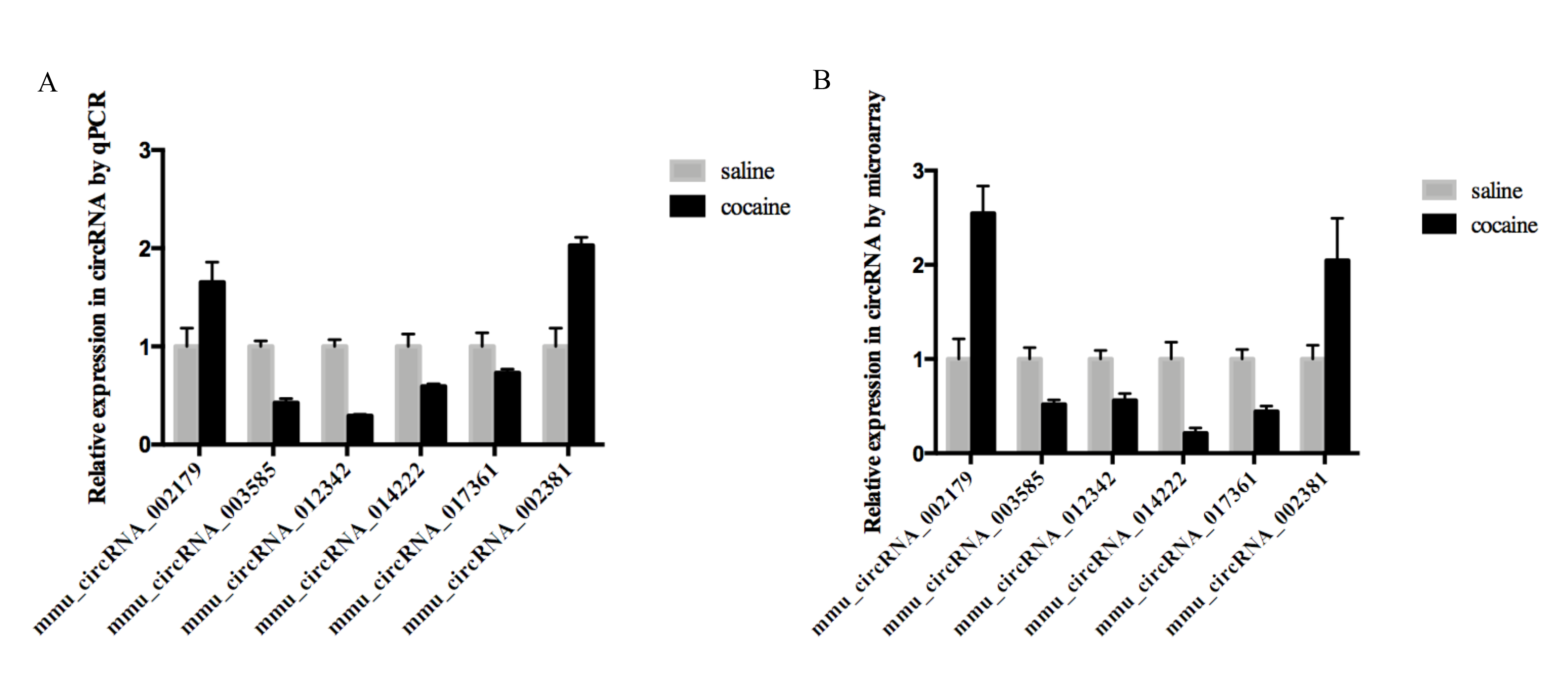

Supplement: Supplementary file 3 — Figure S2 (A) Quantitative RT-PCR confirmation for 6 selected circRNAs. (B) The expression of 6 selected circRNAs by microarray between cocaine group and saline group. [file 41398_2019_527_MOESM3_ESM.tif]

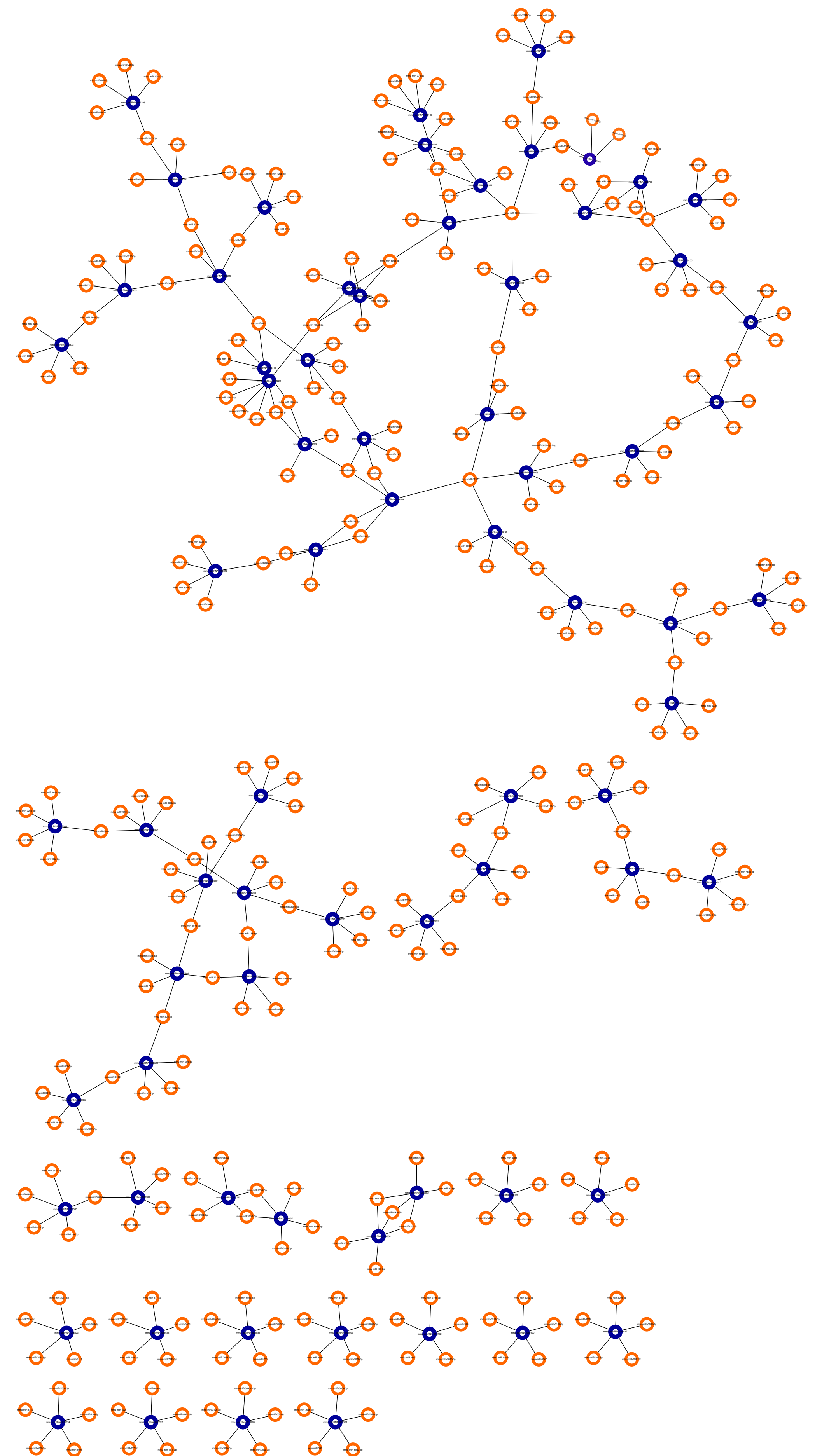

Supplement: Supplementary file 5 — Figure S4 The full size of image for the network consists of down-regulated circRNAs (blue nodes) and their target miRNAs (orange nodes). [file 41398_2019_527_MOESM5_ESM.pdf]

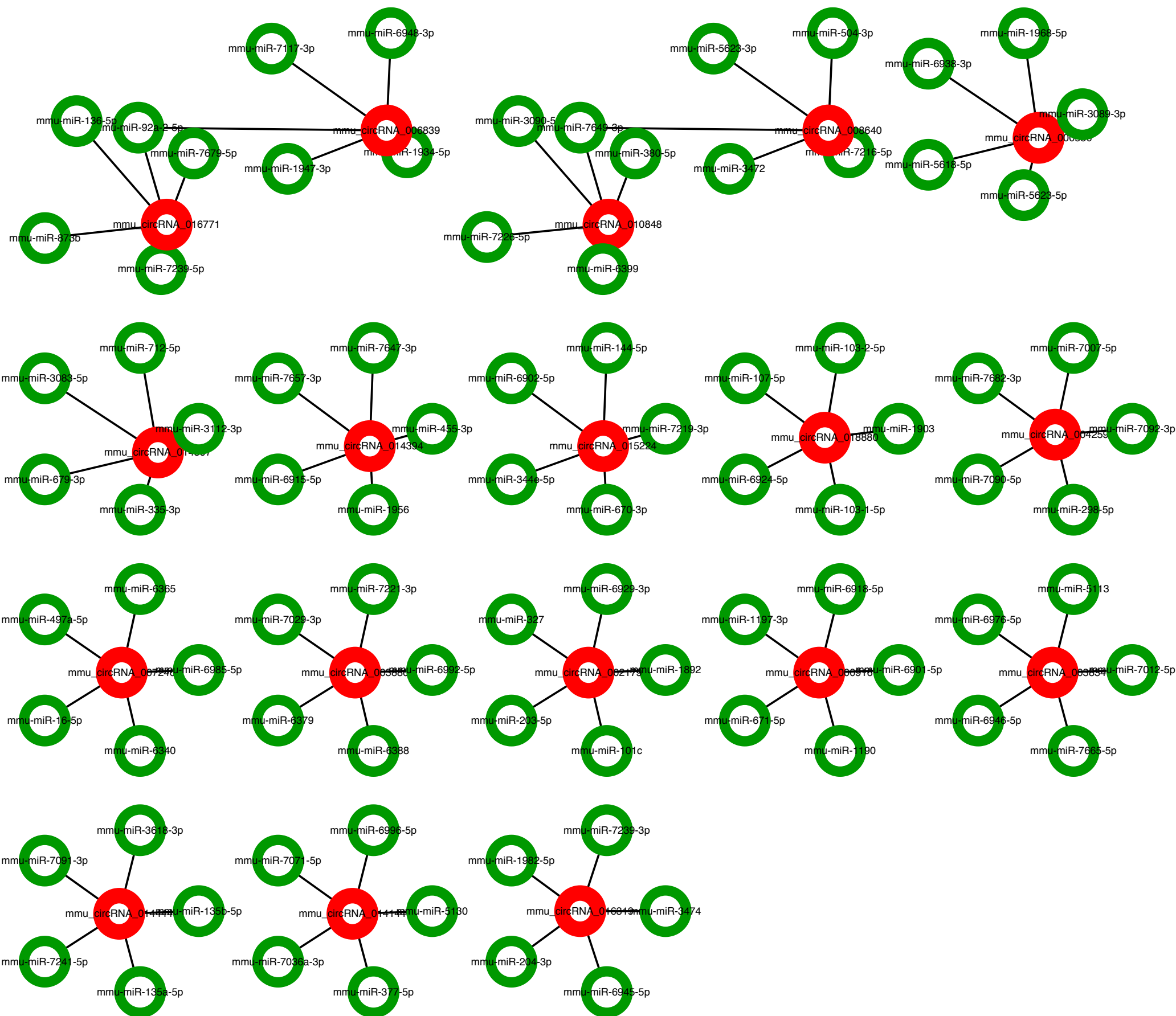

Supplement: Supplementary file 6 — Figure S5 The full size of image for the the network contains up-regulated circRNAs (red nodes) and their target miRNAs (green nodes). [file 41398_2019_527_MOESM6_ESM.pdf]

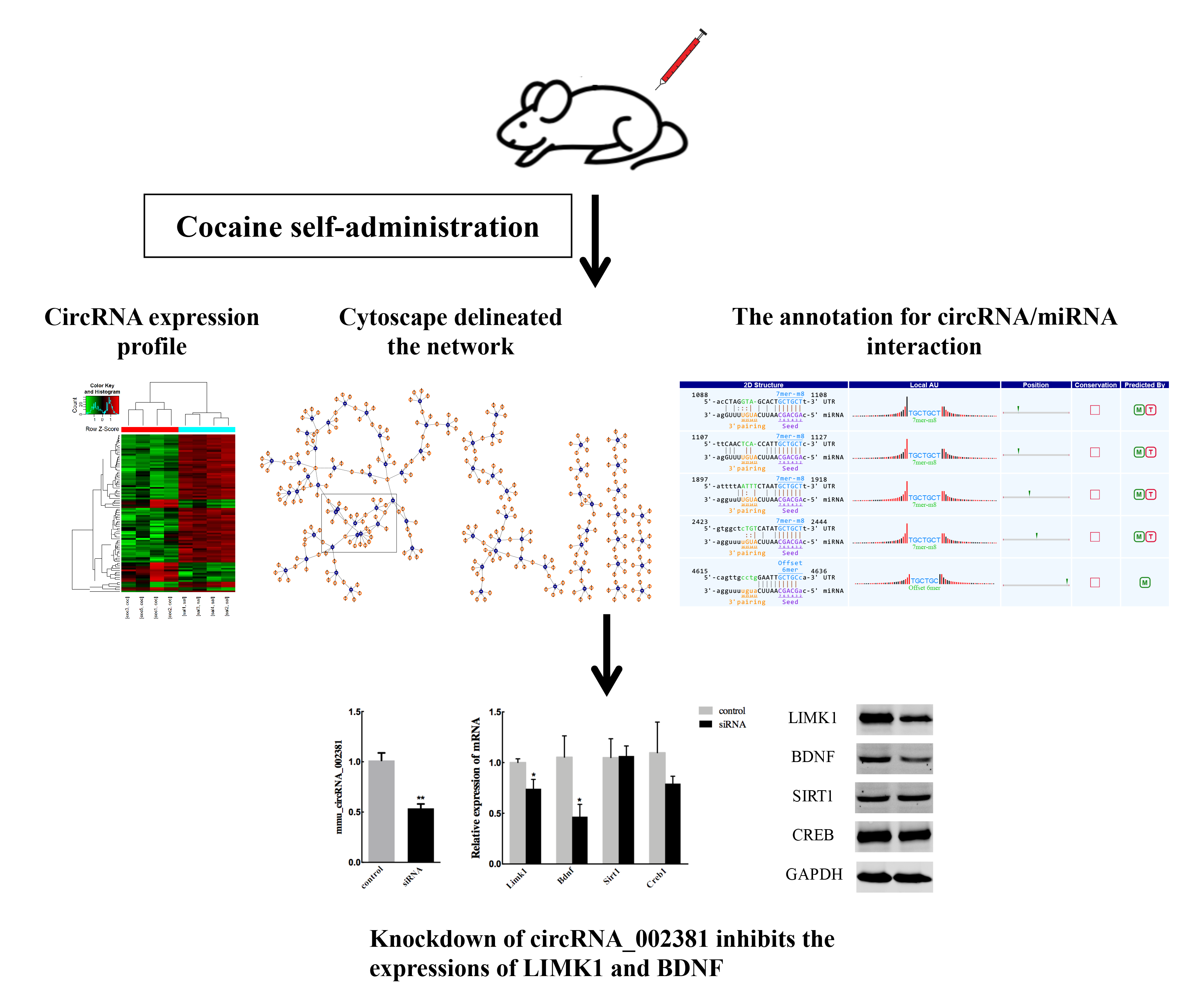

Supplement: Supplementary file 8 — Figure S7 The diagram models the mechanism of circRNA in cocaine self-administration. [file 41398_2019_527_MOESM8_ESM.tif]
