# Supplementary material for: Cocaine induces differential circular RNA expression in striatum
Source: Transl Psychiatry. 2019 Aug 21;9:199. doi: 10.1038/s41398-019-0527-1 (PMC6704174; doi:10.1038/s41398-019-0527-1)
Supplement: Supplementary file 7 — Figure S6 The associations between the miRNAs and their target circRNAs are annotated in MREs for backward prediction. [file 41398_2019_527_MOESM7_ESM.pdf]

2D Structure	Local AU	Position	Conservation	Predicted By
Imperfect match<br>7798 7818<br>5'-ggacATGTTAGCT-CTACTTCa-3' UTR<br>3'-uugaUACGUUGGAUGAUGGAGa-5' miRNA<br>3'pairing Seed	TACTTC<br>Imperfect			M
Imperfect match<br>23193 23214<br>5'-gtaTATGAAGGCCT-CTATCTCa-3' UTR<br>3'-uugAUACGU-UGGAUGAUGGAGa-5' miRNA<br>3'pairing Seed	TATCTC<br>Imperfect			M
Imperfect match<br>25583 25603<br>5'-ggggATTGACCT-CTATCTCc-3' UTR<br>3'-uugaUACGUUGGAUGAUGGAGa-5' miRNA<br>3'pairing Seed	TATCTC<br>Imperfect			M
Imperfect match<br>29042 29060<br>5'-cgCTATGCGGT---CTATCTCt-3' UTR<br>3'-uuGAUACGUUGGAUGAUGGAGa-5' miRNA<br>3'pairing Seed	TATCTC<br>Imperfect			M
Imperfect match<br>30177 30198<br>5'-cgCTACTACTA-CTACTACTTCt-3' UTR<br>3'-uuGAU-ACGUUGGAUGAUGGAGa-5' miRNA<br>3'pairing Seed	TACTTC<br>Imperfect			M
Imperfect match<br>30198 30222<br>5'-tagTACTGCTACTACTGCTACTTCt-3' UTR<br>3'-uugAU-ACGUUG--GAUGAUGGAGa-5' miRNA<br>3'pairing Seed	TACTTC<br>Imperfect			M
Imperfect match<br>31051 31076<br>5'-agaaATGGAATCTAGACCCTGCCTCa-3' UTR<br>3'-uugaUACGUUGGAU----GAUGGAGa-5' miRNA<br>3'pairing Seed	TGCCTC<br>Imperfect			M
7mer-m8<br>31325 31347<br>5'-tcCCAGGCAATCTGACTACCTCt-3' UTR<br>3'-uuGAUACGUUGGA-UGAUGGAGa-5' miRNA<br>3'pairing Seed	CTACCTC<br>7mer-m8			M T
Imperfect match<br>60228 60250<br>5'-ttcacaGTGATCTACCTGCCTCt-3' UTR<br>3'-uugauaCGUUGGAU-GAUGGAGa-5' miRNA<br>3'pairing Seed	TGCCTC<br>Imperfect			M
Imperfect match<br>65658 65680<br>5'-acTTATGCCCAACTCCTATCTCc-3' UTR<br>3'-uuGAUAC-GUUGGAUGAUGGAGa-5' miRNA<br>3'pairing Seed	TATCTC<br>Imperfect			M
Imperfect match<br>74013 74035<br>5'-ctcacaGTGATCTACCTGCCTCt-3' UTR<br>3'-uugauaCGUUGGAU-GAUGGAGa-5' miRNA<br>3'pairing Seed	TGCCTC<br>Imperfect			M
7mer-m8<br>82971 82993<br>5'-gcCTGCTGTGACTTCCTACCTCc-3' UTR<br>3'-uuGAU-ACGUUGGAUGAUGGAGa-5' miRNA<br>3'pairing Seed	CTACCTC<br>7mer-m8			M T

<table>
<tr><th>2D Structure</th><th>Local AU</th><th>Position</th><th>Conservation</th><th>Predicted By</th></tr>
<tr><td>Imperfect match
1318 1339
5'-tgCCATCTGACCTGCTGCCTCt -3' UTR
| || ::||||:||:||||
3'-uuGAUACGUUGGAUGAUGGAGa-5' miRNA
16 15 14 13   7 6 5 4 3 2
3'pairing Seed</td><td>TGCCTC
Imperfect</td><td></td><td></td><td>M</td></tr>
<tr><td>Imperfect match
4217 4237
5'-tgCTTTGC--CCTGACTGCCTCc -3' UTR
|| ||| ||| |||:||||
3'-uuGAUACGUUGGA-UGAUGGAGa-5' miRNA
16 15 14 13   7 6 5 4 3 2
3'pairing Seed</td><td>TGCCTC
Imperfect</td><td></td><td></td><td>M</td></tr>
<tr><td>Imperfect match
4820 4843
5'-tcCTAGGC-ACCTAGCTCTGCCTCt -3' UTR
||| || ||||| ||:||||
3'-uuGAUACGUUGGAU---GAUGGAGa-5' miRNA
16 15 14 13   7 6 5 4 3 2
3'pairing Seed</td><td>TGCCTC
Imperfect</td><td></td><td></td><td>M</td></tr>
<tr><td>Imperfect match
12306 12327
5'-aggccTTCACTCTGCTGCCTCc -3' UTR
| || :||:||:||||
3'-uugauACGUUGGAUGAUGGAGa-5' miRNA
16 15 14 13   7 6 5 4 3 2
3'pairing Seed</td><td>TGCCTC
Imperfect</td><td></td><td></td><td>M</td></tr>
<tr><td>Imperfect match
20005 20027
5'-acaatTCCAAGGCTGCTGCCTCt -3' UTR
| ||| ||:||:||||
3'-uugauACGUU-GGAUGAUGGAGa-5' miRNA
16 15 14 13   7 6 5 4 3 2
3'pairing Seed</td><td>TGCCTC
Imperfect</td><td></td><td></td><td>M</td></tr>
<tr><td>Imperfect match
20886 20907
5'-ggTTGTGCTGCTTGCTATCTTt -3' UTR
:|:||| :|:|:|||:||:
3'-uuGAUACGUUGGAUGAUGGAGa-5' miRNA
16 15 14 13   7 6 5 4 3 2
3'pairing Seed</td><td>TATCTT
Imperfect</td><td></td><td></td><td>M</td></tr>
<tr><td>Imperfect match
34396 34418
5'-aaCTCAGAAATCTGCCTGCCTCt -3' UTR
|| | ||:||: ||:||||
3'-uuGAUACGUUGGAU-GAUGGAGa-5' miRNA
16 15 14 13   7 6 5 4 3 2
3'pairing Seed</td><td>TGCCTC
Imperfect</td><td></td><td></td><td>M</td></tr>
<tr><td>8mer
47736 47757
5'-gaaaaagatagacACTACCTCa-3' UTR
||||||||
3'-uugauacguuggaUGAUGGAGa-5' miRNA
16 15 14 13   7 6 5 4 3 2
3'pairing Seed</td><td>CTACCTCA
8mer</td><td></td><td></td><td>M T</td></tr>
<tr><td>Imperfect match
71109 71128
5'-ctCAATG--ACTTCCTGCCTCt -3' UTR
| ||| ||:| ||:||||
3'-uuGAUACGUUGGAUGAUGGAGa-5' miRNA
16 15 14 13   7 6 5 4 3 2
3'pairing Seed</td><td>TGCCTC
Imperfect</td><td></td><td></td><td>M</td></tr>
<tr><td>Imperfect match
74361 74382
5'-caggAAGCCACTCACTGCCTCc -3' UTR
| || ||: |||:||||
3'-uugaUACGUUGGAUGAUGGAGa-5' miRNA
16 15 14 13   7 6 5 4 3 2
3'pairing Seed</td><td>TGCCTC
Imperfect</td><td></td><td></td><td>M</td></tr>
<tr><td>Offset 6mer
79778 79799
5'-ctgacTGCTTCCAACTACCTgg-3' UTR
||| || ||||||
3'-uugauACGUUGGAUGAUGGAga-5' miRNA
16 15 14 13   7 6 5 4 3 2
3'pairing Seed</td><td>CTACCT
Offset 6mer</td><td></td><td></td><td>M</td></tr>
</table>

<table>
<tr><th>2D Structure</th><th>Local AU</th><th>Position</th><th>Conservation</th><th>Predicted By</th></tr>
<tr><td>4021 7mer-m8 4041
5'-aaCTAGTGTGG--TTCTACCTCt-3' UTR
||| ||::: | |||||||
3'-uuGAU-ACGUUGGAUGAUGGAGa-5' miRNA
16 15 14 13 7 6 5 4 3 2
3'pairing Seed</td><td>CTACCTC
7mer-m8</td><td></td><td></td><td>M T</td></tr>
<tr><td>Imperfect match
5028 5048
5'-caggAAGTATCCT-CTACTTCt-3' UTR
| |:| ||| ||||:||
3'-uugaUACGUUGGAUGAUGGAGa-5' miRNA
16 15 14 13 7 6 5 4 3 2
3'pairing Seed</td><td>TACTTC
Imperfect</td><td></td><td></td><td>M</td></tr>
<tr><td>21264 7mer-m8 21285
5'-gtCTAGGGTGTAAACTACCTCt-3' UTR
||| | :: ||||||||
3'-uuGAUACGUUGGAUGAUGGAGa-5' miRNA
16 15 14 13 7 6 5 4 3 2
3'pairing Seed</td><td>CTACCTC
7mer-m8</td><td></td><td></td><td>M T</td></tr>
<tr><td>31267 7mer-m8 31287
5'-ctTTATTCTATGT-CTACCTCc-3' UTR
:||| | |: | |||||||
3'-uuGAUACGUUGGAUGAUGGAGa-5' miRNA
16 15 14 13 7 6 5 4 3 2
3'pairing Seed</td><td>CTACCTC
7mer-m8</td><td></td><td></td><td>M T</td></tr>
<tr><td>Imperfect match
31332 31353
5'-taaacacTAATCTTCTACTTCt-3' UTR
:||:|| ||||:||
3'-uugauacGUUGGAUGAUGGAGa-5' miRNA
16 15 14 13 7 6 5 4 3 2
3'pairing Seed</td><td>TACTTC
Imperfect</td><td></td><td></td><td>M</td></tr>
<tr><td>Imperfect match
33320 33343
5'-aaCTATAGAGACTTTCCTACTTCt-3' UTR
|||| | :|| :| ||||:||
3'-uuGAUA-CGUUG-GAUGAUGGAGa-5' miRNA
16 15 14 13 7 6 5 4 3 2
3'pairing Seed</td><td>TACTTC
Imperfect</td><td></td><td></td><td>M</td></tr>
<tr><td>Imperfect match
39633 39657
5'-caaTATGCCCAGTACCACTATCTCt-3' UTR
|||| ||:: | ||||:|||
3'-uugAUAC--GUUG-GAUGAUGGAGa-5' miRNA
16 15 14 13 7 6 5 4 3 2
3'pairing Seed</td><td>TATCTC
Imperfect</td><td></td><td></td><td>M</td></tr>
<tr><td>41196 7mer-m8 41217
5'-gaactccCAATCTTCTACCTCc-3' UTR
|||:|| |||||||
3'-uugauacGUUGGAUGAUGGAGa-5' miRNA
16 15 14 13 7 6 5 4 3 2
3'pairing Seed</td><td>CTACCTC
7mer-m8</td><td></td><td></td><td>M T</td></tr>
<tr><td>41734 7mer-m8 41756
5'-agtgATAGCATCCACCTACCTCt-3' UTR
|| ||| || |||||||
3'-uugaUA-CGUUGGAUGAUGGAGa-5' miRNA
16 15 14 13 7 6 5 4 3 2
3'pairing Seed</td><td>CTACCTC
7mer-m8</td><td></td><td></td><td>M T</td></tr>
<tr><td>Imperfect match
46631 46652
5'-atCTCACCAGCCCCCTGCCTCc-3' UTR
|| ||:|| ||:||||
3'-uuGAUACGUUGGAUGAUGGAGa-5' miRNA
16 15 14 13 7 6 5 4 3 2
3'pairing Seed</td><td>TGCCTC
Imperfect</td><td></td><td></td><td>M</td></tr>
<tr><td>Imperfect match
49418 49440
5'-gtCTGAGTCAGCCCTCTGCCTCt-3' UTR
||: | ||:|| ||:||||
3'-uuGAUAC-GUUGGAUGAUGGAGa-5' miRNA
16 15 14 13 7 6 5 4 3 2
3'pairing Seed</td><td>TGCCTC
Imperfect</td><td></td><td></td><td>M</td></tr>
</table>

<table>
<tr><th>2D Structure</th><th>Local AU</th><th>Position</th><th>Conservation</th><th>Predicted By</th></tr>
<tr><td>Imperfect match
5764 5785
5'-ccacAGGTAGTCAGCTATCTCa -3' UTR
| |:|::| :|||:|||
3'-uugaUACGUUGGAUGAUGGAGa-5' miRNA
16 15 14 13 7 6 5 4 3 2
3'pairing Seed</td><td>TATCTC
Imperfect</td><td></td><td>☐</td><td>M</td></tr>
<tr><td>Imperfect match
18951 18973
5'-taCTCTGAAATCCACCTGCCTCt -3' UTR
|| || ||:| | ||:||||
3'-uuGAUACGUUGGAU-GAUGGAGa-5' miRNA
16 15 14 13 7 6 5 4 3 2
3'pairing Seed</td><td>TGCCTC
Imperfect</td><td></td><td>☐</td><td>M</td></tr>
<tr><td>7mer-m8
19165 19187
5'-tcTTATG-AGGCTACTCTACCTCt-3' UTR
:|||| |: ||| |||||||
3'-uuGAUACGUUGGAU--GAUGGAGa-5' miRNA
16 15 14 13 7 6 5 4 3 2
3'pairing Seed</td><td>CTACCTC
7mer-m8</td><td></td><td>☐</td><td>M T</td></tr>
<tr><td>Imperfect match
20343 20364
5'-ttCCATAGGAGCC-ACTACTTCt -3' UTR
| || | |:|| |||||:||
3'-uuGAUA-CGUUGGAUGAUGGAGa-5' miRNA
16 15 14 13 7 6 5 4 3 2
3'pairing Seed</td><td>TACTTC
Imperfect</td><td></td><td>☐</td><td>M</td></tr>
<tr><td>Imperfect match
25953 25974
5'-ttCTGT-CAACCCAGCTGCCTCa -3' UTR
||:| ||||| | ||:||||
3'-uuGAUACGUUGGAU-GAUGGAGa-5' miRNA
16 15 14 13 7 6 5 4 3 2
3'pairing Seed</td><td>TGCCTC
Imperfect</td><td></td><td>☐</td><td>M</td></tr>
<tr><td>7mer-m8
43360 43379
5'-gtaacTCCAACT--CTACCTCt-3' UTR
| ||||: |||||||
3'-uugauACGUUGGAUGAUGGAGa-5' miRNA
16 15 14 13 7 6 5 4 3 2
3'pairing Seed</td><td>CTACCTC
7mer-m8</td><td></td><td>☐</td><td>M T</td></tr>
<tr><td>7mer-m8
46141 46163
5'-taCAAACCATTCTCTCTACCTCt-3' UTR
| | || :|| |||||||
3'-uuGAUACGUUGGA-UGAUGGAGa-5' miRNA
16 15 14 13 7 6 5 4 3 2
3'pairing Seed</td><td>CTACCTC
7mer-m8</td><td></td><td>☐</td><td>M T</td></tr>
<tr><td>Imperfect match
48481 48503
5'-ggcagcaCACCCATACTACTTCa -3' UTR
|| || ||||||:||
3'-uugauacGUUGG-AUGAUGGAGa-5' miRNA
16 15 14 13 7 6 5 4 3 2
3'pairing Seed</td><td>TACTTC
Imperfect</td><td></td><td>☐</td><td>M</td></tr>
<tr><td>7mer-m8
53354 53376
5'-ctgTAAACTACCTACCTACCTCt-3' UTR
|| | ||||| |||||||
3'-uugAUACGUUGGAU-GAUGGAGa-5' miRNA
16 15 14 13 7 6 5 4 3 2
3'pairing Seed</td><td>CTACCTC
7mer-m8</td><td></td><td>☐</td><td>M T</td></tr>
<tr><td>Imperfect match
65228 65250
5'-ggcagcaCACCCATACTACTTCa -3' UTR
|| || ||||||:||
3'-uugauacGUUGG-AUGAUGGAGa-5' miRNA
16 15 14 13 7 6 5 4 3 2
3'pairing Seed</td><td>TACTTC
Imperfect</td><td></td><td>☐</td><td>M</td></tr>
<tr><td>Imperfect match
67826 67847
5'-ttTTCTGCTACCTGCTGTCTCt -3' UTR
:| ||| ||||:||::|||
3'-uuGAUACGUUGGAUGAUGGAGa-5' miRNA
16 15 14 13 7 6 5 4 3 2
3'pairing Seed</td><td>TGTCTC
Imperfect</td><td></td><td>☐</td><td>M</td></tr>
</table>

2D Structure	Local AU	Position	Conservation	Predicted By
Imperfect match<br>4373 4394<br>5'-tgCCATCTGACCTGCTGCCTCt -3' UTR<br>3'-uuGAUACGUUGGAUGAUGGAGa-5' miRNA<br>16 15 14 13 7 6 5 4 3 2<br>3'pairing Seed	TGCCTC<br>Imperfect			M
Imperfect match<br>7272 7292<br>5'-tgCTTTGC--CCTGACTGCCTCc -3' UTR<br>3'-uuGAUACGUUGGA-UGAUGGAGa-5' miRNA<br>16 15 14 13 7 6 5 4 3 2<br>3'pairing Seed	TGCCTC<br>Imperfect			M
Imperfect match<br>7875 7898<br>5'-tcCTAGGC-ACCTAGCTCTGCCTCt -3' UTR<br>3'-uuGAUACGUUGGAU---GAUGGAGa-5' miRNA<br>16 15 14 13 7 6 5 4 3 2<br>3'pairing Seed	TGCCTC<br>Imperfect			M
Imperfect match<br>15361 15382<br>5'-aggccTTCACTCTGCTGCCTCc -3' UTR<br>3'-uugauACGUUGGAUGAUGGAGa-5' miRNA<br>16 15 14 13 7 6 5 4 3 2<br>3'pairing Seed	TGCCTC<br>Imperfect			M
Imperfect match<br>23060 23082<br>5'-acaatTCCAAGGCTGCTGCCTCt -3' UTR<br>3'-uugauACGUU-GGAUGAUGGAGa-5' miRNA<br>16 15 14 13 7 6 5 4 3 2<br>3'pairing Seed	TGCCTC<br>Imperfect			M
Imperfect match<br>23941 23962<br>5'-ggTTGTGCTGCTTGCTATCTTt -3' UTR<br>3'-uuGAUACGUUGGAUGAUGGAGa-5' miRNA<br>16 15 14 13 7 6 5 4 3 2<br>3'pairing Seed	TATCTT<br>Imperfect			M
Imperfect match<br>37451 37473<br>5'-aaCTCAGAAATCTGCCTGCCTCt -3' UTR<br>3'-uuGAUACGUUGGAU-GAUGGAGa-5' miRNA<br>16 15 14 13 7 6 5 4 3 2<br>3'pairing Seed	TGCCTC<br>Imperfect			M
8mer<br>50791 50812<br>5'-gaaaaagatagacACTACCTCa-3' UTR<br>3'-uugauacguuggaUGAUGGAGa-5' miRNA<br>16 15 14 13 7 6 5 4 3 2<br>3'pairing Seed	CTACCTCA<br>8mer			M T
Imperfect match<br>74164 74183<br>5'-ctCAATG--ACTTCCTGCCTCt -3' UTR<br>3'-uuGAUACGUUGGAUGAUGGAGa-5' miRNA<br>16 15 14 13 7 6 5 4 3 2<br>3'pairing Seed	TGCCTC<br>Imperfect			M
Imperfect match<br>77416 77437<br>5'-caggAAGCCACTCACTGCCTCc -3' UTR<br>3'-uugaUACGUUGGAUGAUGGAGa-5' miRNA<br>16 15 14 13 7 6 5 4 3 2<br>3'pairing Seed	TGCCTC<br>Imperfect			M

2D Structure	Local AU	Position	Conservation	Predicted By
Imperfect match<br>3430 5'-gcCT-TGAAATCTACCTGCCTCt -3' UTR 3451<br>3'-uuGAUACGUUGGAU-GAUGGAGa-5' miRNA<br>16 15 14 13 / 7 6 5 4 3 2<br>3'pairing Seed	TGCCTC<br>Imperfect			M
Imperfect match<br>3648 5'-ccCTA-GTGACATACTGCCTCt -3' UTR 3668<br>3'-uuGAUACGUUGGAUGAUGGAGa-5' miRNA<br>16 15 14 13 / 7 6 5 4 3 2<br>3'pairing Seed	TGCCTC<br>Imperfect			M
Imperfect match<br>8006 5'-aaCTCAGAAATCTGCCTGCCTCt -3' UTR 8028<br>3'-uuGAUACGUUGGAU-GAUGGAGa-5' miRNA<br>16 15 14 13 / 7 6 5 4 3 2<br>3'pairing Seed	TGCCTC<br>Imperfect			M
8mer<br>12762 5'-gaaaagaCAGAC-ACTACCTCa-3' UTR 12782<br>3'-uugauacGUUGGAUGAUGGAGa-5' miRNA<br>16 15 14 13 / 7 6 5 4 3 2<br>3'pairing Seed	CTACCTCA<br>8mer			M T
Imperfect match<br>19782 5'-gtaacaGCAGTTTCACTACTTCa -3' UTR 19804<br>3'-uugauaCGUUGGA-UGAUGGAGa-5' miRNA<br>16 15 14 13 / 7 6 5 4 3 2<br>3'pairing Seed	TACTTC<br>Imperfect			M
Imperfect match<br>38912 5'-gcgcctctctCCTGCTGCCTCt -3' UTR 38933<br>3'-uugauacguuGGAUGAUGGAGa-5' miRNA<br>16 15 14 13 / 7 6 5 4 3 2<br>3'pairing Seed	TGCCTC<br>Imperfect			M
Imperfect match<br>39476 5'-aaCTCAGAAATCTGCCTGCCTCt -3' UTR 39498<br>3'-uuGAUACGUUGGAU-GAUGGAGa-5' miRNA<br>16 15 14 13 / 7 6 5 4 3 2<br>3'pairing Seed	TGCCTC<br>Imperfect			M
8mer<br>40196 5'-gaTTGGCCAGCACTTCTACCTCa-3' UTR 40218<br>3'-uuGAUACGUUG-GAUGAUGGAGa-5' miRNA<br>16 15 14 13 / 7 6 5 4 3 2<br>3'pairing Seed	CTACCTCA<br>8mer			M T
8mer<br>47703 5'-acaaagaCAGAC-ACTACCTCa-3' UTR 47723<br>3'-uugauacGUUGGAUGAUGGAGa-5' miRNA<br>16 15 14 13 / 7 6 5 4 3 2<br>3'pairing Seed	CTACCTCA<br>8mer			M T
7mer-A1<br>48042 5'-atCTCTGCATGTATATTACCTCa-3' UTR 48064<br>3'-uuGAUACGU-UGGAUGAUGGAGa-5' miRNA<br>16 15 14 13 / 7 6 5 4 3 2<br>3'pairing Seed	TTACCTCA<br>7mer-A1			M T
Imperfect match<br>56586 5'-gcCTATGCTTCAGCTCCTGCCTCc -3' UTR 56609<br>3'-uuGAUACG--UUGGAUGAUGGAGa-5' miRNA<br>16 15 14 13 / 7 6 5 4 3 2<br>3'pairing Seed	TGCCTC<br>Imperfect			M
Imperfect match<br>69077 5'-gcaTATGTGACCTCAAAGCCTGCCTCc -3' UTR 69103<br>3'-uugAUACGUUGGA---U--GAUGGAGa-5' miRNA<br>16 15 14 13 / 7 6 5 4 3 2<br>3'pairing Seed	TGCCTC<br>Imperfect			M
8mer<br>77394 5'-tcaaagaCAAAC-ACTACCTCa-3' UTR 77414<br>3'-uugauacGUUGGAUGAUGGAGa-5' miRNA<br>16 15 14 13 / 7 6 5 4 3 2<br>3'pairing Seed	CTACCTCA<br>8mer			M T
Imperfect match<br>86748 5'-aaCTCAGAAATCTGCCTGCCTCt -3' UTR 86770<br>3'-uuGAUACGUUGGAU-GAUGGAGa-5' miRNA<br>16 15 14 13 / 7 6 5 4 3 2<br>3'pairing Seed	TGCCTC<br>Imperfect			M
7mer-m8<br>90403 5'-ttgagtctccagatCTACCTCt-3' UTR 90424<br>3'-uugauacguuggauGAUGGAGa-5' miRNA<br>16 15 14 13 / 7 6 5 4 3 2<br>3'pairing Seed	CTACCTC<br>7mer-m8			M T

<table>
<tr><th>2D Structure</th><th>Local AU</th><th>Position</th><th>Conservation</th><th>Predicted By</th></tr>
<tr><td>Imperfect match
642 663
5'-tgCCATCTGACCTGCTGCCTCt-3' UTR
| || ::||||:||:||||
3'-uuGAUACGUUGGAUGAUGGAGa-5' miRNA
16 15 14 13 7 6 5 4 3 2
3'pairing Seed</td><td>TGCCTC
Imperfect</td><td></td><td></td><td>M</td></tr>
<tr><td>Imperfect match
3541 3561
5'-tgCTTTGC--CCTGACTGCCTCc-3' UTR
|| ||| ||| |||:||||
3'-uuGAUACGUUGGA-UGAUGGAGa-5' miRNA
16 15 14 13 7 6 5 4 3 2
3'pairing Seed</td><td>TGCCTC
Imperfect</td><td></td><td></td><td>M</td></tr>
<tr><td>Imperfect match
4144 4167
5'-tcCTAGGC-ACCTAGCTCTGCCTCt-3' UTR
||| || ||||| ||:||||
3'-uuGAUACGUUGGAU---GAUGGAGa-5' miRNA
16 15 14 13 7 6 5 4 3 2
3'pairing Seed</td><td>TGCCTC
Imperfect</td><td></td><td></td><td>M</td></tr>
<tr><td>Imperfect match
11630 11651
5'-aggccTTCACTCTGCTGCCTCc-3' UTR
| || :||:||:||||
3'-uugauACGUUGGAUGAUGGAGa-5' miRNA
16 15 14 13 7 6 5 4 3 2
3'pairing Seed</td><td>TGCCTC
Imperfect</td><td></td><td></td><td>M</td></tr>
<tr><td>Imperfect match
19329 19351
5'-acaatTCCAAGGCTGCTGCCTCt-3' UTR
| ||| ||:||:||||
3'-uugauACGUU-GGAUGAUGGAGa-5' miRNA
16 15 14 13 7 6 5 4 3 2
3'pairing Seed</td><td>TGCCTC
Imperfect</td><td></td><td></td><td>M</td></tr>
<tr><td>Imperfect match
20210 20231
5'-ggTTGTGCTGCTTGCTATCTTt-3' UTR
:|:||| :|:|:|||:||:
3'-uuGAUACGUUGGAUGAUGGAGa-5' miRNA
16 15 14 13 7 6 5 4 3 2
3'pairing Seed</td><td>TATCTT
Imperfect</td><td></td><td></td><td>M</td></tr>
<tr><td>Imperfect match
33720 33742
5'-aaCTCAGAAATCTGCCTGCCTCt-3' UTR
|| | ||:||: ||:||||
3'-uuGAUACGUUGGAU-GAUGGAGa-5' miRNA
16 15 14 13 7 6 5 4 3 2
3'pairing Seed</td><td>TGCCTC
Imperfect</td><td></td><td></td><td>M</td></tr>
<tr><td>8mer
47060 47081
5'-gaaaaagatagacACTACCTCa-3' UTR
|||||||
3'-uugauacguuggaUGAUGGAGa-5' miRNA
16 15 14 13 7 6 5 4 3 2
3'pairing Seed</td><td>CTACCTCA
8mer</td><td></td><td></td><td>M T</td></tr>
<tr><td>Imperfect match
70433 70452
5'-ctCAATG--ACTTCCTGCCTCt-3' UTR
| ||| ||:| ||:||||
3'-uuGAUACGUUGGAUGAUGGAGa-5' miRNA
16 15 14 13 7 6 5 4 3 2
3'pairing Seed</td><td>TGCCTC
Imperfect</td><td></td><td></td><td>M</td></tr>
<tr><td>Imperfect match
73685 73706
5'-caggAAGCCACTCACTGCCTCc-3' UTR
| || ||: |||:||||
3'-uugaUACGUUGGAUGAUGGAGa-5' miRNA
16 15 14 13 7 6 5 4 3 2
3'pairing Seed</td><td>TGCCTC
Imperfect</td><td></td><td></td><td>M</td></tr>
</table>

2D Structure	Local AU	Position	Conservation	Predicted By
Imperfect match<br>193 213<br>5'-tgCTTTGC--CCTGACTGCCTCc -3' UTR<br>3'-uuGAUACGUUGGA-UGAUGGAGa-5' miRNA<br>16 15 14 13 7 6 5 4 3 2<br>3'pairing Seed	TGCCTC<br>Imperfect			M
Imperfect match<br>796 819<br>5'-tcCTAGGC-ACCTAGCTCTGCCTCt -3' UTR<br>3'-uuGAUACGUUGGAU---GAUGGAGa-5' miRNA<br>16 15 14 13 7 6 5 4 3 2<br>3'pairing Seed	TGCCTC<br>Imperfect			M
Imperfect match<br>8282 8303<br>5'-aggccTTCACTCTGCTGCCTCc -3' UTR<br>3'-uugauACGUUGGAUGAUGGAGa-5' miRNA<br>16 15 14 13 7 6 5 4 3 2<br>3'pairing Seed	TGCCTC<br>Imperfect			M
Imperfect match<br>15981 16003<br>5'-acaatTCCAAGGCTGCTGCCTCt -3' UTR<br>3'-uugauACGUU-GGAUGAUGGAGa-5' miRNA<br>16 15 14 13 7 6 5 4 3 2<br>3'pairing Seed	TGCCTC<br>Imperfect			M
Imperfect match<br>16862 16883<br>5'-ggTTGTGCTGCTTGCTATCTTt -3' UTR<br>3'-uuGAUACGUUGGAUGAUGGAGa-5' miRNA<br>16 15 14 13 7 6 5 4 3 2<br>3'pairing Seed	TATCTT<br>Imperfect			M
Imperfect match<br>30372 30394<br>5'-aaCTCAGAAATCTGCCTGCCTCt -3' UTR<br>3'-uuGAUACGUUGGAU-GAUGGAGa-5' miRNA<br>16 15 14 13 7 6 5 4 3 2<br>3'pairing Seed	TGCCTC<br>Imperfect			M
8mer<br>43712 43733<br>5'-gaaaaagatagacACTACCTCa-3' UTR<br>3'-uugauacguuggaUGAUGGAGa-5' miRNA<br>16 15 14 13 7 6 5 4 3 2<br>3'pairing Seed	CTACCTCA<br>8mer			M T
Imperfect match<br>67085 67104<br>5'-ctCAATG--ACTTCCTGCCTCt -3' UTR<br>3'-uuGAUACGUUGGAUGAUGGAGa-5' miRNA<br>16 15 14 13 7 6 5 4 3 2<br>3'pairing Seed	TGCCTC<br>Imperfect			M
Imperfect match<br>70337 70358<br>5'-caggAAGCCACTCACTGCCTCc -3' UTR<br>3'-uugaUACGUUGGAUGAUGGAGa-5' miRNA<br>16 15 14 13 7 6 5 4 3 2<br>3'pairing Seed	TGCCTC<br>Imperfect			M
Imperfect match<br>77947 77968<br>5'-tgCCATCTGACCTGCTGCCTCt -3' UTR<br>3'-uuGAUACGUUGGAUGAUGGAGa-5' miRNA<br>16 15 14 13 7 6 5 4 3 2<br>3'pairing Seed	TGCCTC<br>Imperfect			M
7mer-m8<br>79679 79703<br>5'-gggtcccTGACCTGTGGCTACCTCc-3' UTR<br>3'-uugauacGUUGGA---UGAUGGAGa-5' miRNA<br>16 15 14 13 7 6 5 4 3 2<br>3'pairing Seed	CTACCTC<br>7mer-m8			M T

2D Structure	Local AU	Position	Conservation	Predicted By
8087 8mer 8108<br>5'-gctccgtCATTAAACTACCTCa-3' UTR<br>3'-uugauacGUUGGAUGAUGGAGa-5' miRNA<br>16 15 14 13 / 7 6 5 4 3 2<br>3'pairing Seed	CTACCTCA<br>8mer			M T
12805 Imperfect match 12827<br>5'-aactcaaaAACCTGCCTGCCTCt -3' UTR<br>3'-uugauacgUUGGAU-GAUGGAGa-5' miRNA<br>16 15 14 13 / 7 6 5 4 3 2<br>3'pairing Seed	TGCCTC<br>Imperfect			M
16570 Imperfect match 16592<br>5'-aaCTCTGAAATCTGCCTGCCTCt -3' UTR<br>3'-uuGAUACGUUGGAU-GAUGGAGa-5' miRNA<br>16 15 14 13 / 7 6 5 4 3 2<br>3'pairing Seed	TGCCTC<br>Imperfect			M
19920 Imperfect match 19942<br>5'-aaCTCAGAAATTTACCTGCCTCt -3' UTR<br>3'-uuGAUACGUUGGAU-GAUGGAGa-5' miRNA<br>16 15 14 13 / 7 6 5 4 3 2<br>3'pairing Seed	TGCCTC<br>Imperfect			M
20246 Imperfect match 20268<br>5'-ttCTTGGGAGTCTAGCTGCCTCt -3' UTR<br>3'-uuGAUACGUUGGAU-GAUGGAGa-5' miRNA<br>16 15 14 13 / 7 6 5 4 3 2<br>3'pairing Seed	TGCCTC<br>Imperfect			M
23019 Imperfect match 23041<br>5'-aaCTCAGAAACCTGTCTGCCTCt -3' UTR<br>3'-uuGAUACGUUGGAU-GAUGGAGa-5' miRNA<br>16 15 14 13 / 7 6 5 4 3 2<br>3'pairing Seed	TGCCTC<br>Imperfect			M
26127 Imperfect match 26148<br>5'-aaaTCTGCCAGTCT-CTGCCTCc -3' UTR<br>3'-uugAUAC-GUUGGAUGAUGGAGa-5' miRNA<br>16 15 14 13 / 7 6 5 4 3 2<br>3'pairing Seed	TGCCTC<br>Imperfect			M
27192 7mer-m8 27214<br>5'-aaTTATGTACAATTGCTACCTCt-3' UTR<br>3'-uuGAUACGU-UGGAUGAUGGAGa-5' miRNA<br>16 15 14 13 / 7 6 5 4 3 2<br>3'pairing Seed	CTACCTC<br>7mer-m8			M T
32210 Imperfect match 32231<br>5'-gggtagagtACCTTCTACTTCt -3' UTR<br>3'-uugauacguUGGAUGAUGGAGa-5' miRNA<br>16 15 14 13 / 7 6 5 4 3 2<br>3'pairing Seed	TACTTC<br>Imperfect			M
38273 Imperfect match 38292<br>5'-atCCATGT-GCCT-CTGCCTCt -3' UTR<br>3'-uuGAUACGUUGGAUGAUGGAGa-5' miRNA<br>16 15 14 13 / 7 6 5 4 3 2<br>3'pairing Seed	TGCCTC<br>Imperfect			M
52115 8mer 52136<br>5'-acaacccTGAGATTCTACCTCa-3' UTR<br>3'-uugauacGUUGGAUGAUGGAGa-5' miRNA<br>16 15 14 13 / 7 6 5 4 3 2<br>3'pairing Seed	CTACCTCA<br>8mer			M T

<table>
<tr><th>2D Structure</th><th>Local AU</th><th>Position</th><th>Conservation</th><th>Predicted By</th></tr>
<tr><td>Imperfect match
6720 6741
5'-gcCT-TGAAATCTACCTGCCTCt -3' UTR
|| || ||:||| ||:||||
3'-uuGAUACGUUGGAU-GAUGGAGa-5' miRNA
16 15 14 13 7 6 5 4 3 2
3'pairing Seed</td><td>TGCCTC
Imperfect</td><td></td><td></td><td>M</td></tr>
<tr><td>Imperfect match
6938 6958
5'-ccCTA-GTGACATACTGCCTCt -3' UTR
||| |::|| ||||:||||
3'-uuGAUACGUUGGAUGAUGGAGa-5' miRNA
16 15 14 13 7 6 5 4 3 2
3'pairing Seed</td><td>TGCCTC
Imperfect</td><td></td><td></td><td>M</td></tr>
<tr><td>Imperfect match
11296 11318
5'-aaCTCAGAAATCTGCCTGCCTCt -3' UTR
|| | ||:||: ||:||||
3'-uuGAUACGUUGGAU-GAUGGAGa-5' miRNA
16 15 14 13 7 6 5 4 3 2
3'pairing Seed</td><td>TGCCTC
Imperfect</td><td></td><td></td><td>M</td></tr>
<tr><td>8mer
16052 16072
5'-gaaaagaCAGAC-ACTACCTCa-3' UTR
||: | ||||||||
3'-uugauacGUUGGAUGAUGGAGa-5' miRNA
16 15 14 13 7 6 5 4 3 2
3'pairing Seed</td><td>CTACCTCA
8mer</td><td></td><td></td><td>M T</td></tr>
<tr><td>Imperfect match
23072 23094
5'-gtaacaGCAGTTTCACTACTTCa -3' UTR
|||:::| |||||:||
3'-uugauaCGUUGGA-UGAUGGAGa-5' miRNA
16 15 14 13 7 6 5 4 3 2
3'pairing Seed</td><td>TACTTC
Imperfect</td><td></td><td></td><td>M</td></tr>
<tr><td>Imperfect match
42202 42223
5'-gcgcctctctCCTGCTGCCTCt -3' UTR
|||:||:||||
3'-uugauacguuGGAUGAUGGAGa-5' miRNA
16 15 14 13 7 6 5 4 3 2
3'pairing Seed</td><td>TGCCTC
Imperfect</td><td></td><td></td><td>M</td></tr>
<tr><td>Imperfect match
42766 42788
5'-aaCTCAGAAATCTGCCTGCCTCt -3' UTR
|| | ||:||: ||:||||
3'-uuGAUACGUUGGAU-GAUGGAGa-5' miRNA
16 15 14 13 7 6 5 4 3 2
3'pairing Seed</td><td>TGCCTC
Imperfect</td><td></td><td></td><td>M</td></tr>
<tr><td>8mer
43486 43508
5'-gaTTGGCCAGCACTTCTACCTCa-3' UTR
:|: ||:| || |||||||
3'-uuGAUACGUUG-GAUGAUGGAGa-5' miRNA
16 15 14 13 7 6 5 4 3 2
3'pairing Seed</td><td>CTACCTCA
8mer</td><td></td><td></td><td>M T</td></tr>
<tr><td>8mer
50993 51013
5'-acaaagaCAGAC-ACTACCTCa-3' UTR
||: | ||||||||
3'-uugauacGUUGGAUGAUGGAGa-5' miRNA
16 15 14 13 7 6 5 4 3 2
3'pairing Seed</td><td>CTACCTCA
8mer</td><td></td><td></td><td>M T</td></tr>
<tr><td>7mer-A1
51332 51354
5'-atCTCTGCATGTATATTACCTCa-3' UTR
|| |||| :: ||:||||||
3'-uuGAUACGU-UGGAUGAUGGAGa-5' miRNA
16 15 14 13 7 6 5 4 3 2
3'pairing Seed</td><td>TTACCTCA
7mer-A1</td><td></td><td></td><td>M T</td></tr>
<tr><td>Imperfect match
59876 59899
5'-gcCTATGCTTCAGCTCCTGCCTCc -3' UTR
|||||| | || ||:||||
3'-uuGAUACG--UUGGAUGAUGGAGa-5' miRNA
16 15 14 13 7 6 5 4 3 2
3'pairing Seed</td><td>TGCCTC
Imperfect</td><td></td><td></td><td>M</td></tr>
<tr><td>Imperfect match
72367 72393
5'-gcaTATGTGACCTCAAAGCCTGCCTCc -3' UTR
||||::|||| | ||:||||
3'-uugAUACGUUGGA---U--GAUGGAGa-5' miRNA
16 15 14 13 7 6 5 4 3 2
3'pairing Seed</td><td>TGCCTC
Imperfect</td><td></td><td></td><td>M</td></tr>
<tr><td>8mer
80684 80704
5'-tcaaagaCAAAC-ACTACCTCa-3' UTR
||| | ||||||||
3'-uugauacGUUGGAUGAUGGAGa-5' miRNA
16 15 14 13 7 6 5 4 3 2
3'pairing Seed</td><td>CTACCTCA
8mer</td><td></td><td></td><td>M T</td></tr>
</table>

<table>
<tr><th>2D Structure</th><th>Local AU</th><th>Position</th><th>Conservation</th><th>Predicted By</th></tr>
<tr><td>Imperfect match
3490   3511
5'-aatggcGCCACCTACTGCCTTc -3' UTR
|| |||||||:|||:
3'-uugauaCGUUGGAUGAUGGAGa-5' miRNA
16 15 14 13   7 6 5 4 3 2
3'pairing   Seed</td><td>TGCCTT
Imperfect</td><td></td><td></td><td>M</td></tr>
<tr><td>Imperfect match
22129   22150
5'-agCTTGGTGACCTCCTATCTCt -3' UTR
|| |::|||| |||:|||
3'-uuGAUACGUUGGAUGAUGGAGa-5' miRNA
16 15 14 13   7 6 5 4 3 2
3'pairing   Seed</td><td>TATCTC
Imperfect</td><td></td><td></td><td>M</td></tr>
<tr><td>Imperfect match
25927   25948
5'-tgCTGGGATGACTACTACTTCa -3' UTR
||: | : |||||||:||
3'-uuGAUACGUUGGAUGAUGGAGa-5' miRNA
16 15 14 13   7 6 5 4 3 2
3'pairing   Seed</td><td>TACTTC
Imperfect</td><td></td><td></td><td>M</td></tr>
<tr><td>Imperfect match
35090   35110
5'-ggggATTTGACCT-CTATCTCc -3' UTR
|| ::|||| |||:|||
3'-uugaUACGUUGGAUGAUGGAGa-5' miRNA
16 15 14 13   7 6 5 4 3 2
3'pairing   Seed</td><td>TATCTC
Imperfect</td><td></td><td></td><td>M</td></tr>
<tr><td>8mer
41961   41983
5'-tatgATGGAATGATGCTACCTCa-3' UTR
||| ||: |:|||||||
3'-uugaUACGUUG-GAUGAUGGAGa-5' miRNA
16 15 14 13   7 6 5 4 3 2
3'pairing   Seed</td><td>CTACCTCA
8mer</td><td></td><td></td><td>M T</td></tr>
<tr><td>Imperfect match
46574   46594
5'-caagGTGC-ACTTCCTGCCTCt -3' UTR
:||| ||:| ||:||||
3'-uugaUACGUUGGAUGAUGGAGa-5' miRNA
16 15 14 13   7 6 5 4 3 2
3'pairing   Seed</td><td>TGCCTC
Imperfect</td><td></td><td></td><td>M</td></tr>
<tr><td>Imperfect match
71395   71414
5'-tgCTATGC-ACC-ACTACTTTt -3' UTR
|||||| ||| |||||:|:
3'-uuGAUACGUUGGAUGAUGGAGa-5' miRNA
16 15 14 13   7 6 5 4 3 2
3'pairing   Seed</td><td>TACTTT
Imperfect</td><td></td><td></td><td>M</td></tr>
<tr><td>Imperfect match
72977   72998
5'-aatgggtCAACATTCTACTTCc -3' UTR
|||| | ||||:||
3'-uugauacGUUGGAUGAUGGAGa-5' miRNA
16 15 14 13   7 6 5 4 3 2
3'pairing   Seed</td><td>TACTTC
Imperfect</td><td></td><td></td><td>M</td></tr>
<tr><td>7mer-m8
78632   78651
5'-tataccGTGAC--ACTACCTCt-3' UTR
|::|| ||||||||
3'-uugauaCGUUGGAUGAUGGAGa-5' miRNA
16 15 14 13   7 6 5 4 3 2
3'pairing   Seed</td><td>CTACCTC
7mer-m8</td><td></td><td></td><td>M T</td></tr>
<tr><td>7mer-m8
84760   84780
5'-tagTGGGCAAATT-CTACCTCc-3' UTR
|: |||| :| |||||||
3'-uugAUACGUUGGAUGAUGGAGa-5' miRNA
16 15 14 13   7 6 5 4 3 2
3'pairing   Seed</td><td>CTACCTC
7mer-m8</td><td></td><td></td><td>M T</td></tr>
<tr><td>Imperfect match
91013   91036
5'-acaaATGCACCCCCATCTGCCTCt -3' UTR
||||| || | ||:||||
3'-uugaUACGU-UGGAU-GAUGGAGa-5' miRNA
16 15 14   13   7 6 5 4 3 2
3'pairing   Seed</td><td>TGCCTC
Imperfect</td><td></td><td></td><td>M</td></tr>
</table>

2D Structure	Local AU	Position	Conservation	Predicted By
Imperfect match<br>7228 7249<br>5'-aatggcGCCACCTACTGCCTTc-3' UTR<br>3'-uugauaCGUUGGAUGAUGGAGa-5' miRNA<br>16 15 14 13 7 6 5 4 3 2<br>3'pairing Seed	TGCCTT<br>Imperfect			M
Imperfect match<br>25867 25888<br>5'-agCTTGGTGACCTCCTATCTCt-3' UTR<br>3'-uuGAUACGUUGGAUGAUGGAGa-5' miRNA<br>16 15 14 13 7 6 5 4 3 2<br>3'pairing Seed	TATCTC<br>Imperfect			M
Imperfect match<br>29665 29686<br>5'-tgCTGGGATGACTACTACTTCa-3' UTR<br>3'-uuGAUACGUUGGAUGAUGGAGa-5' miRNA<br>16 15 14 13 7 6 5 4 3 2<br>3'pairing Seed	TACTTC<br>Imperfect			M
Imperfect match<br>38828 38848<br>5'-ggggATTTGACCT-CTATCTCc-3' UTR<br>3'-uugaUACGUUGGAUGAUGGAGa-5' miRNA<br>16 15 14 13 7 6 5 4 3 2<br>3'pairing Seed	TATCTC<br>Imperfect			M
8mer<br>45699 45721<br>5'-tatgATGGAATGATGCTACCTCa-3' UTR<br>3'-uugaUACGUUG-GAUGAUGGAGa-5' miRNA<br>16 15 14 13 7 6 5 4 3 2<br>3'pairing Seed	CTACCTCA<br>8mer			M T
Imperfect match<br>50312 50332<br>5'-caagGTGC-ACTTCCTGCCTCt-3' UTR<br>3'-uugaUACGUUGGAUGAUGGAGa-5' miRNA<br>16 15 14 13 7 6 5 4 3 2<br>3'pairing Seed	TGCCTC<br>Imperfect			M
Imperfect match<br>75133 75152<br>5'-tgCTATGC-ACC-ACTACTTTt-3' UTR<br>3'-uuGAUACGUUGGAUGAUGGAGa-5' miRNA<br>16 15 14 13 7 6 5 4 3 2<br>3'pairing Seed	TACTTT<br>Imperfect			M
Imperfect match<br>76715 76736<br>5'-aatgggtCAACATTCTACTTCc-3' UTR<br>3'-uugauacGUUGGAUGAUGGAGa-5' miRNA<br>16 15 14 13 7 6 5 4 3 2<br>3'pairing Seed	TACTTC<br>Imperfect			M
7mer-m8<br>82370 82389<br>5'-tataccGTGAC--ACTACCTCt-3' UTR<br>3'-uugauaCGUUGGAUGAUGGAGa-5' miRNA<br>16 15 14 13 7 6 5 4 3 2<br>3'pairing Seed	CTACCTC<br>7mer-m8			M T
7mer-m8<br>88498 88518<br>5'-tagTGGGCAAATT-CTACCTCc-3' UTR<br>3'-uugAUACGUUGGAUGAUGGAGa-5' miRNA<br>16 15 14 13 7 6 5 4 3 2<br>3'pairing Seed	CTACCTC<br>7mer-m8			M T
Imperfect match<br>94751 94774<br>5'-acaaATGCACCCCCATCTGCCTCt-3' UTR<br>3'-uugaUACGU-UGGAU-GAUGGAGa-5' miRNA<br>16 15 14 13 7 6 5 4 3 2<br>3'pairing Seed	TGCCTC<br>Imperfect			M

2D Structure	Local AU	Position	Conservation	Predicted By
8291 7mer-m8 8312<br>5'-caaacTACCACATTCTACCTCc-3' UTR<br>3'-uugauACGUUGGAUGAUGGAGa-5' miRNA<br>3'pairing Seed	CTACCTC<br>7mer-m8		□	M T
Imperfect match<br>10227 10248<br>5'-atCTAT-CTATCTATCTATCTCt -3' UTR<br>3'-uuGAUACGUUGGAU-GAUGGAGa-5' miRNA<br>3'pairing Seed	TATCTC<br>Imperfect		□	M
21447 7mer-m8 21468<br>5'-ctCAATTTCATGTTCTACCTCt-3' UTR<br>3'-uuGAUACGUUGGAUGAUGGAGa-5' miRNA<br>3'pairing Seed	CTACCTC<br>7mer-m8		□	M T
23077 7mer-m8 23097<br>5'-gaaccTGTAG-AGACTACCTCt-3' UTR<br>3'-uugauACGUUGGAUGAUGGAGa-5' miRNA<br>3'pairing Seed	CTACCTC<br>7mer-m8		□	M T
28929 7mer-m8 28950<br>5'-caacATGAAACGTTCTACCTCc-3' UTR<br>3'-uugaUACGUUGGAUGAUGGAGa-5' miRNA<br>3'pairing Seed	CTACCTC<br>7mer-m8		□	M T
Imperfect match<br>38922 38941<br>5'-atCT-TGTAACC-ACTGCCTCa -3' UTR<br>3'-uuGAUACGUUGGAUGAUGGAGa-5' miRNA<br>3'pairing Seed	TGCCTC<br>Imperfect		□	M
Offset 6mer<br>43566 43587<br>5'-ttTTCTGCAAGAAACTACCTTt-3' UTR<br>3'-uuGAUACGUUGGAUGAUGGAGa-5' miRNA<br>3'pairing Seed	CTACCT<br>Offset 6mer		□	M
51142 7mer-m8 51165<br>5'-tcCTCAGGAATACTACCTACCTCt-3' UTR<br>3'-uuGAUACGUUG-GAU-GAUGGAGa-5' miRNA<br>3'pairing Seed	CTACCTC<br>7mer-m8		□	M T
Imperfect match<br>79030 79051<br>5'-aatgATTCTCCCTGCTGCCTCa -3' UTR<br>3'-uugaUACGUUGGAUGAUGGAGa-5' miRNA<br>3'pairing Seed	TGCCTC<br>Imperfect		□	M
Imperfect match<br>96260 96282<br>5'-ccggGTGCTAGCCATCTATCTCt -3' UTR<br>3'-uugaUACG-UUGGAUGAUGGAGa-5' miRNA<br>3'pairing Seed	TATCTC<br>Imperfect		□	M

<table>
<tr><th>2D Structure</th><th>Local AU</th><th>Position</th><th>Conservation</th><th>Predicted By</th></tr>
<tr><td>17088 7mer-m8 17110
5'-ttaTAGGCTTTCAATCTACCTCc-3' UTR
|| || :| | |||||||
3'-uugAUACGUUGGAU-GAUGGAGa-5' miRNA
16 15 14 13 7 6 5 4 3 2
3'pairing Seed</td><td>CTACCTC
7mer-m8</td><td></td><td></td><td>M T</td></tr>
<tr><td>22471 8mer 22491
5'-ttgcAGGTAA-CAGCTACCTCa-3' UTR
| |:|| | :|||||||
3'-uugaUACGUUGGAUGAUGGAGa-5' miRNA
16 15 14 13 7 6 5 4 3 2
3'pairing Seed</td><td>CTACCTCA
8mer</td><td></td><td></td><td>M T</td></tr>
<tr><td>24206 7mer-m8 24228
5'-ggaTATAAATAGTAGCTACCTCt-3' UTR
||| | | : :|||||||
3'-uugAUACGU-UGGAUGAUGGAGa-5' miRNA
16 15 14 13 7 6 5 4 3 2
3'pairing Seed</td><td>CTACCTC
7mer-m8</td><td></td><td></td><td>M T</td></tr>
<tr><td>Imperfect match
29550 29572
5'-aaCTCAGAAATCTGCCTGCCTCt -3' UTR
|| | ||:||: ||:||||
3'-uuGAUACGUUGGAU-GAUGGAGa-5' miRNA
16 15 14 13 7 6 5 4 3 2
3'pairing Seed</td><td>TGCCTC
Imperfect</td><td></td><td></td><td>M</td></tr>
<tr><td>52140 7mer-m8 52161
5'-ttaatTTCATTCTACTACCTCt-3' UTR
| || :||||||||||
3'-uugauACGUUGGAUGAUGGAGa-5' miRNA
16 15 14 13 7 6 5 4 3 2
3'pairing Seed</td><td>CTACCTC
7mer-m8</td><td></td><td></td><td>M T</td></tr>
<tr><td>52656 7mer-m8 52676
5'-aaattcaaAATCT-CTACCTCt-3' UTR
||:|| |||||||
3'-uugauacgUUGGAUGAUGGAGa-5' miRNA
16 15 14 13 7 6 5 4 3 2
3'pairing Seed</td><td>CTACCTC
7mer-m8</td><td></td><td></td><td>M T</td></tr>
<tr><td>Imperfect match
57518 57540
5'-ccTTAATCAACTAAGCTATCTCa -3' UTR
:|| ||||: | |||:|||
3'-uuGAUACGUUGGAU-GAUGGAGa-5' miRNA
16 15 14 13 7 6 5 4 3 2
3'pairing Seed</td><td>TATCTC
Imperfect</td><td></td><td></td><td>M</td></tr>
<tr><td>Imperfect match
70264 70287
5'-acCTTTTCCATCTACACTATCTCc -3' UTR
|| | | |:|| ||||:|||
3'-uuGAUACGUUGGA--UGAUGGAGa-5' miRNA
16 15 14 13 7 6 5 4 3 2
3'pairing Seed</td><td>TATCTC
Imperfect</td><td></td><td></td><td>M</td></tr>
<tr><td>96747 8mer 96769
5'-ataTAAGTTGCTCACCTACCTCa-3' UTR
|| |: :|: | |||||||
3'-uugAUACGUUGGAU-GAUGGAGa-5' miRNA
16 15 14 13 7 6 5 4 3 2
3'pairing Seed</td><td>CTACCTCA
8mer</td><td></td><td></td><td>M T</td></tr>
<tr><td>Imperfect match
97031 97051
5'-aaaTAGGCAA-TGGCTGCCTCa -3' UTR
|| |||| : :||:||||
3'-uugAUACGUUGGAUGAUGGAGa-5' miRNA
16 15 14 13 7 6 5 4 3 2
3'pairing Seed</td><td>TGCCTC
Imperfect</td><td></td><td></td><td>M</td></tr>
</table>

2D Structure	Local AU	Position	Conservation	Predicted By
Imperfect match<br>21528 21551<br>5'-tgCTGTCCAGCCTGAGCTGCCTCa-3' UTR<br>3'-uuGAUACGUUGGA--UGAUGGAGa-5' miRNA<br>16 15 14 13 7 6 5 4 3 2<br>3'pairing Seed	TGCCTC<br>Imperfect			M
Imperfect match<br>23206 23228<br>5'-aaCTCAGAAATCTGCCTGCCTCt-3' UTR<br>3'-uuGAUACGUUGGAU-GAUGGAGa-5' miRNA<br>16 15 14 13 7 6 5 4 3 2<br>3'pairing Seed	TGCCTC<br>Imperfect			M
Imperfect match<br>33098 33119<br>5'-ctCTATCTCTATCT-CTATCTCt-3' UTR<br>3'-uuGAUA-CGUUGGAUGAUGGAGa-5' miRNA<br>16 15 14 13 7 6 5 4 3 2<br>3'pairing Seed	TATCTC<br>Imperfect			M
Imperfect match<br>33122 33143<br>5'-ctCTATCTCTATCT-CTATCTCt-3' UTR<br>3'-uuGAUA-CGUUGGAUGAUGGAGa-5' miRNA<br>16 15 14 13 7 6 5 4 3 2<br>3'pairing Seed	TATCTC<br>Imperfect			M
7mer-m8<br>33152 33173<br>5'-ctCTATCTCTACCT-CTACCTCt-3' UTR<br>3'-uuGAUA-CGUUGGAUGAUGGAGa-5' miRNA<br>16 15 14 13 7 6 5 4 3 2<br>3'pairing Seed	CTACCTC<br>7mer-m8			M T
7mer-m8<br>33165 33185<br>5'-tctacctCTACCT-CTACCTCt-3' UTR<br>3'-uugauacGUUGGAUGAUGGAGa-5' miRNA<br>16 15 14 13 7 6 5 4 3 2<br>3'pairing Seed	CTACCTC<br>7mer-m8			M T
7mer-m8<br>33177 33197<br>5'-tctacctCTACCT-CTACCTCt-3' UTR<br>3'-uugauacGUUGGAUGAUGGAGa-5' miRNA<br>16 15 14 13 7 6 5 4 3 2<br>3'pairing Seed	CTACCTC<br>7mer-m8			M T
7mer-m8<br>33189 33209<br>5'-tctacctCTACCT-CTACCTCt-3' UTR<br>3'-uugauacGUUGGAUGAUGGAGa-5' miRNA<br>16 15 14 13 7 6 5 4 3 2<br>3'pairing Seed	CTACCTC<br>7mer-m8			M T
7mer-m8<br>33201 33221<br>5'-tctacctCTACCT-CTACCTCt-3' UTR<br>3'-uugauacGUUGGAUGAUGGAGa-5' miRNA<br>16 15 14 13 7 6 5 4 3 2<br>3'pairing Seed	CTACCTC<br>7mer-m8			M T
Imperfect match<br>33244 33267<br>5'-ctaTATCTATATCTATCTATCTCt-3' UTR<br>3'-uugAUACGU-UGGAU-GAUGGAGa-5' miRNA<br>16 15 14 13 7 6 5 4 3 2<br>3'pairing Seed	TATCTC<br>Imperfect			M
Offset 6mer<br>46999 47021<br>5'-agagcTGCACATCTGCTACCTac-3' UTR<br>3'-uugauACGU-UGGAUGAUGGAga-5' miRNA<br>16 15 14 13 7 6 5 4 3 2<br>3'pairing Seed	CTACCT<br>Offset 6mer			M

<table>
<tr><th>2D Structure</th><th>Local AU</th><th>Position</th><th>Conservation</th><th>Predicted By</th></tr>
<tr><td>Imperfect match
3461 3483
5'-ctgcGTGCAGACTTCCTGCCTCa -3' UTR
:||| ||:| ||:||||
3'-uugaUACGU-UGGAUGAUGGAGa-5' miRNA
16 15 14 13 7 6 5 4 3 2
3'pairing Seed</td><td>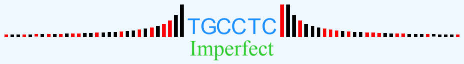
</td><td></td><td></td><td>M</td></tr>
<tr><td>Imperfect match
21760 21779
5'-ctCTAT-CTGCCT-CTGCCTCc -3' UTR
|||| | :||| ||:||||
3'-uuGAUACGUUGGAUGAUGGAGa-5' miRNA
16 15 14 13 7 6 5 4 3 2
3'pairing Seed</td><td>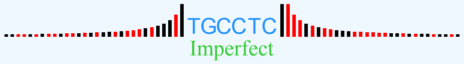
</td><td></td><td></td><td>M</td></tr>
<tr><td>Imperfect match
22058 22080
5'-ctcacaGCGATCCTCCTGCCTCt -3' UTR
||:| ||| ||:||||
3'-uugauaCGUU-GGAUGAUGGAGa-5' miRNA
16 15 14 13 7 6 5 4 3 2
3'pairing Seed</td><td>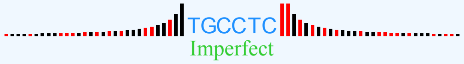
</td><td></td><td></td><td>M</td></tr>
<tr><td>Imperfect match
23685 23706
5'-tcTTATTTGACCTTCAACCTCt -3' UTR
:||| ::|||| | |||||
3'-uuGAUACGUUGGAUGAUGGAGa-5' miRNA
16 15 14 13 7 6 5 4 3 2
3'pairing Seed</td><td>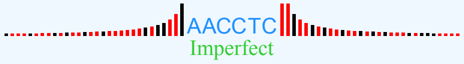
</td><td></td><td></td><td>M</td></tr>
<tr><td>Imperfect match
26122 26143
5'-gtCTGTTCATCCTTCAACCTCt -3' UTR
||:| || ||| | |||||
3'-uuGAUACGUUGGAUGAUGGAGa-5' miRNA
16 15 14 13 7 6 5 4 3 2
3'pairing Seed</td><td>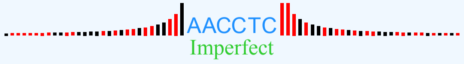
</td><td></td><td></td><td>M</td></tr>
<tr><td>7mer-m8
26293 26314
5'-gagcATATATTTGACTACCTCt-3' UTR
|| :| :: |||||||
3'-uugaUACGUUGGAUGAUGGAGa-5' miRNA
16 15 14 13 7 6 5 4 3 2
3'pairing Seed</td><td>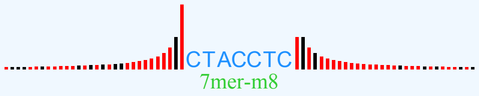
</td><td></td><td></td><td>M T</td></tr>
<tr><td>7mer-m8
28735 28757
5'-ccattaaaAAAGTACCTACCTCt-3' UTR
|| || |||||||
3'-uugauacgUUGGAU-GAUGGAGa-5' miRNA
16 15 14 13 7 6 5 4 3 2
3'pairing Seed</td><td>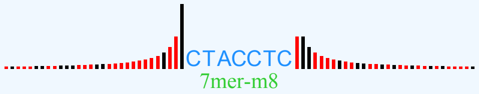
</td><td></td><td></td><td>M T</td></tr>
<tr><td>Imperfect match
29935 29957
5'-agaTGTGTCCCTTCACTACTTCa -3' UTR
|:||: |:| |||||:||
3'-uugAUACGUUGGA-UGAUGGAGa-5' miRNA
16 15 14 13 7 6 5 4 3 2
3'pairing Seed</td><td>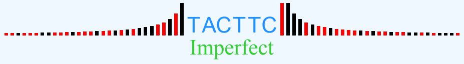
</td><td></td><td></td><td>M</td></tr>
<tr><td>Imperfect match
31667 31686
5'-ggCGATG--ACTTCCTATCTCa -3' UTR
| ||| ||:| |||:|||
3'-uuGAUACGUUGGAUGAUGGAGa-5' miRNA
16 15 14 13 7 6 5 4 3 2
3'pairing Seed</td><td>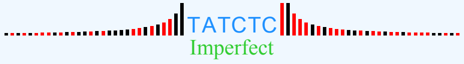
</td><td></td><td></td><td>M</td></tr>
<tr><td>Imperfect match
39010 39029
5'-atCTGTGT-GCCT-CTGCCTCc -3' UTR
||:||: :||| ||:||||
3'-uuGAUACGUUGGAUGAUGGAGa-5' miRNA
16 15 14 13 7 6 5 4 3 2
3'pairing Seed</td><td>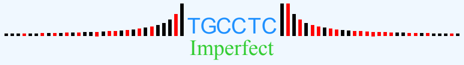
</td><td></td><td></td><td>M</td></tr>
</table>

2D Structure	Local AU	Position	Conservation	Predicted By
Imperfect match<br>149 170<br>5'-aaCAAGGCAGCCTACAACCTCc-3' UTR<br>3'-uuGAUACGUUGGAUGAUGGAGa-5' miRNA<br>3'pairing Seed	AACCTC<br>Imperfect			M
Imperfect match<br>15239 15259<br>5'-atCT-TGCCCCCACTGCCTCc-3' UTR<br>3'-uuGAUACGUUGGAUGAUGGAGa-5' miRNA<br>3'pairing Seed	TGCCTC<br>Imperfect			M
Imperfect match<br>16921 16942<br>5'-ccagAAGCAACATGCTGCCTCt-3' UTR<br>3'-uugaUACGUUGGAUGAUGGAGa-5' miRNA<br>3'pairing Seed	TGCCTC<br>Imperfect			M
Imperfect match<br>32374 32395<br>5'-ccTTAGGTATTCTCCTGCCTCc-3' UTR<br>3'-uuGAUACGUUGGAUGAUGGAGa-5' miRNA<br>3'pairing Seed	TGCCTC<br>Imperfect			M
Imperfect match<br>41023 41045<br>5'-ggacGTACGACTTAACTGCCTCc-3' UTR<br>3'-uugaUACGUUGGA-UGAUGGAGa-5' miRNA<br>3'pairing Seed	TGCCTC<br>Imperfect			M
Imperfect match<br>43134 43156<br>5'-aaCTCAGAAATCCTCCTGCCTCt-3' UTR<br>3'-uuGAUACGUU-GGAUGAUGGAGa-5' miRNA<br>3'pairing Seed	TGCCTC<br>Imperfect			M
7mer-m8<br>49882 49902<br>5'-aaatcccttGTCT-CTACCTCc-3' UTR<br>3'-uugauacguuGGAUGAUGGAGa-5' miRNA<br>3'pairing Seed	CTACCTC<br>7mer-m8			M T
Imperfect match<br>54753 54775<br>5'-tcTTTTTCAGTTGTACTACTTCt-3' UTR<br>3'-uuGAUACGUUGG-AUGAUGGAGa-5' miRNA<br>3'pairing Seed	TACTTC<br>Imperfect			M
Imperfect match<br>58505 58527<br>5'-acagATGCTCCCTCTCTGCCTCc-3' UTR<br>3'-uugaUACGUUGGA-UGAUGGAGa-5' miRNA<br>3'pairing Seed	TGCCTC<br>Imperfect			M
7mer-m8<br>60994 61014<br>5'-ctTTCTG-AAGTTTCTACCTCc-3' UTR<br>3'-uuGAUACGUUGGAUGAUGGAGa-5' miRNA<br>3'pairing Seed	CTACCTC<br>7mer-m8			M T
Imperfect match<br>71275 71296<br>5'-ataatccCAGCCTTCTGCCTCt-3' UTR<br>3'-uugauacGUUGGAUGAUGGAGa-5' miRNA<br>3'pairing Seed	TGCCTC<br>Imperfect			M
Imperfect match<br>83251 83273<br>5'-tcCTGTAACCATCTACTGCCTCt-3' UTR<br>3'-uuGAUA-CGUUGGAUGAUGGAGa-5' miRNA<br>3'pairing Seed	TGCCTC<br>Imperfect			M

2D Structure	Local AU	Position	Conservation	Predicted By
Imperfect match<br>1588 … 1608<br>5'-agagATGC-TCTTGCTGCCTCt-3' UTR<br>3'-uugaUACGUUGGAUGAUGGAGa-5' miRNA<br>3'pairing Seed	TGCCTC<br>Imperfect			M
Imperfect match<br>3299 … 3320<br>5'-aaCAAAGCATGTGACTATCTCa-3' UTR<br>3'-uuGAUACGUUGGAUGAUGGAGa-5' miRNA<br>3'pairing Seed	TATCTC<br>Imperfect			M
Imperfect match<br>8096 … 8117<br>5'-caTTGTGCAACTTACTATCTTg-3' UTR<br>3'-uuGAUACGUUGGAUGAUGGAGa-5' miRNA<br>3'pairing Seed	TATCTT<br>Imperfect			M
Imperfect match<br>11285 … 11305<br>5'-ttagATG-AGTCTGCTATCTCt-3' UTR<br>3'-uugaUACGUUGGAUGAUGGAGa-5' miRNA<br>3'pairing Seed	TATCTC<br>Imperfect			M
7mer-m8<br>14426 … 14449<br>5'-tgCTACTCACTACCCTCTACCTCt-3' UTR<br>3'-uuGAUACGU--UGGAUGAUGGAGa-5' miRNA<br>3'pairing Seed	CTACCTC<br>7mer-m8			M T
8mer<br>15797 … 15820<br>5'-ccCTATGTAGCCCAGGCTACCTCa-3' UTR<br>3'-uuGAUACGUUGGAU--GAUGGAGa-5' miRNA<br>3'pairing Seed	CTACCTCA<br>8mer			M T
Imperfect match<br>17735 … 17755<br>5'-ttggATTCAGCCT-CTATCTCt-3' UTR<br>3'-uugaUACGUUGGAUGAUGGAGa-5' miRNA<br>3'pairing Seed	TATCTC<br>Imperfect			M
7mer-m8<br>19139 … 19160<br>5'-ttcagcGTTATCTGCTACCTCt-3' UTR<br>3'-uugauaCGUUGGAUGAUGGAGa-5' miRNA<br>3'pairing Seed	CTACCTC<br>7mer-m8			M T
8mer<br>20237 … 20257<br>5'-tgCTATGT-GCATTCTACCTCa-3' UTR<br>3'-uuGAUACGUUGGAUGAUGGAGa-5' miRNA<br>3'pairing Seed	CTACCTCA<br>8mer			M T
Imperfect match<br>25568 … 25589<br>5'-atggtTGCTATTTTCTATCTCt-3' UTR<br>3'-uugauACGUUGGAUGAUGGAGa-5' miRNA<br>3'pairing Seed	TATCTC<br>Imperfect			M
Imperfect match<br>25820 … 25841<br>5'-taaggaGAAATCTTCTGCCTCt-3' UTR<br>3'-uugauaCGUUGGAUGAUGGAGa-5' miRNA<br>3'pairing Seed	TGCCTC<br>Imperfect			M

2D Structure	Local AU	Position	Conservation	Predicted By
Imperfect match<br>17874 17895<br>5'-agCCAAGCAATCTGCTACTTGg -3' UTR<br>| | ||||:||:||||:| 3'-uuGAUACGUUGGAUGAUGGAGa-5' miRNA<br>16 15 14 13 7 6 5 4 3 2<br>3'pairing Seed	TACTTG<br>Imperfect		☐	M
Imperfect match<br>20292 20314<br>5'-tctcaccCATATCTACTACTTCc -3' UTR<br>|| |:|||||||:|| 3'-uugauacGU-UGGAUGAUGGAGa-5' miRNA<br>16 15 14 13 7 6 5 4 3 2<br>3'pairing Seed	TACTTC<br>Imperfect		☐	M
Imperfect match<br>30535 30556<br>5'-gggtagaatACCTTCTACTTCt -3' UTR<br>|||| ||||:|| 3'-uugauacguUGGAUGAUGGAGa-5' miRNA<br>16 15 14 13 7 6 5 4 3 2<br>3'pairing Seed	TACTTC<br>Imperfect		☐	M
Imperfect match<br>33848 33869<br>5'-gcCCACGCAGGGTGCTGCCTCa -3' UTR<br>| | |||: |:||:|||| 3'-uuGAUACGUUGGAUGAUGGAGa-5' miRNA<br>16 15 14 13 7 6 5 4 3 2<br>3'pairing Seed	TGCCTC<br>Imperfect		☐	M
Imperfect match<br>56099 56120<br>5'-tcCTGTCCAAATTGCTCCCTCc -3' UTR<br>||:| ||| :|:|| |||| 3'-uuGAUACGUUGGAUGAUGGAGa-5' miRNA<br>16 15 14 13 7 6 5 4 3 2<br>3'pairing Seed	TCCCTC<br>Imperfect		☐	M
Imperfect match<br>60045 60066<br>5'-tcagAAGCAGTTAGCTATCTCc -3' UTR<br>| |||::: :|||:||| 3'-uugaUACGUUGGAUGAUGGAGa-5' miRNA<br>16 15 14 13 7 6 5 4 3 2<br>3'pairing Seed	TATCTC<br>Imperfect		☐	M
7mer-m8<br>72883 72903<br>5'-acaaagaCAGAC-ACTACCTCt-3' UTR<br>||: | ||||||| 3'-uugauacGUUGGAUGAUGGAGa-5' miRNA<br>16 15 14 13 7 6 5 4 3 2<br>3'pairing Seed	CTACCTC<br>7mer-m8		☐	M T
Imperfect match<br>79964 79986<br>5'-aaCTCAGAAATCTGCCTGCCTCt -3' UTR<br>|| | ||:||: ||:|||| 3'-uuGAUACGUUGGAU-GAUGGAGa-5' miRNA<br>16 15 14 13 7 6 5 4 3 2<br>3'pairing Seed	TGCCTC<br>Imperfect		☐	M
Imperfect match<br>81344 81366<br>5'-aaCTCAGAAATCTGCCTGCCTCt -3' UTR<br>|| | ||:||: ||:|||| 3'-uuGAUACGUUGGAU-GAUGGAGa-5' miRNA<br>16 15 14 13 7 6 5 4 3 2<br>3'pairing Seed	TGCCTC<br>Imperfect		☐	M
Imperfect match<br>83842 83864<br>5'-atgaATGTGTATTGACTATCTCt -3' UTR<br>|||:: |:: ||||:||| 3'-uugaUACGU-UGGAUGAUGGAGa-5' miRNA<br>16 15 14 13 7 6 5 4 3 2<br>3'pairing Seed	TATCTC<br>Imperfect		☐	M

<table>
<tr><th>2D Structure</th><th>Local AU</th><th>Position</th><th>Conservation</th><th>Predicted By</th></tr>
<tr><td>Offset 6mer
2480 2499
5'-ttCCTCT-GTTGG-CAGTCAgt-3' UTR
||||| ||::: ||||||
3'-ggGGAGACCAGUUGGUCAGUGu-5' miRNA
16 15 14 13 7 6 5 4 3 2
3'pairing Seed</td><td>CAGTCA
Offset 6mer</td><td></td><td></td><td>M</td></tr>
<tr><td>7mer-m8
6307 6329
5'-tgacaagaGTCAGCACAGTCACc-3' UTR
||||:| |||||||
3'-ggggagacCAGUUG-GUCAGUGu-5' miRNA
16 15 14 13 7 6 5 4 3 2
3'pairing Seed</td><td>CAGTCAC
7mer-m8</td><td></td><td></td><td>M T</td></tr>
<tr><td>8mer
9433 9454
5'-gacagCTCATTATTCAGTCACa-3' UTR
|| |:| :|||||||
3'-ggggaGACCAGUUGGUCAGUGu-5' miRNA
16 15 14 13 7 6 5 4 3 2
3'pairing Seed</td><td>CAGTCACA
8mer</td><td></td><td></td><td>M T</td></tr>
<tr><td>7mer-A1
20323 20344
5'-ttCTTTATGTATACTAGTCACa-3' UTR
|:|: || ||:||||||
3'-ggGGAGACCAGUUGGUCAGUGu-5' miRNA
16 15 14 13 7 6 5 4 3 2
3'pairing Seed</td><td>TAGTCACA
7mer-A1</td><td></td><td></td><td>M T</td></tr>
<tr><td>6mer
23669 23690
5'-gggCTTGGATCCACTAGTCACt-3' UTR
||: | || ||:||||||
3'-gggGAGACCAGUUGGUCAGUGu-5' miRNA
16 15 14 13 7 6 5 4 3 2
3'pairing Seed</td><td>AGTCAC
6mer</td><td></td><td></td><td>M</td></tr>
<tr><td>Offset 6mer
33847 33866
5'-ttCCTCT-GTTGG-CAGTCAgt-3' UTR
||||| ||::: ||||||
3'-ggGGAGACCAGUUGGUCAGUGu-5' miRNA
16 15 14 13 7 6 5 4 3 2
3'pairing Seed</td><td>CAGTCA
Offset 6mer</td><td></td><td></td><td>M</td></tr>
<tr><td>7mer-m8
37675 37697
5'-tgacaagaGTCAGCACAGTCACc-3' UTR
||||:| |||||||
3'-ggggagacCAGUUG-GUCAGUGu-5' miRNA
16 15 14 13 7 6 5 4 3 2
3'pairing Seed</td><td>CAGTCAC
7mer-m8</td><td></td><td></td><td>M T</td></tr>
<tr><td>8mer
40801 40822
5'-gacagCTCATTATTCAGTCACa-3' UTR
|| |:| :|||||||
3'-ggggaGACCAGUUGGUCAGUGu-5' miRNA
16 15 14 13 7 6 5 4 3 2
3'pairing Seed</td><td>CAGTCACA
8mer</td><td></td><td></td><td>M T</td></tr>
<tr><td>7mer-A1
51691 51712
5'-ttCTTTATGTATACTAGTCACa-3' UTR
|:|: || ||:||||||
3'-ggGGAGACCAGUUGGUCAGUGu-5' miRNA
16 15 14 13 7 6 5 4 3 2
3'pairing Seed</td><td>TAGTCACA
7mer-A1</td><td></td><td></td><td>M T</td></tr>
<tr><td>6mer
55044 55065
5'-gggCTTGGATCCACTAGTCACt-3' UTR
||: | || ||:||||||
3'-gggGAGACCAGUUGGUCAGUGu-5' miRNA
16 15 14 13 7 6 5 4 3 2
3'pairing Seed</td><td>AGTCAC
6mer</td><td></td><td></td><td>M</td></tr>
</table>

<table>
<tr><th>2D Structure</th><th>Local AU</th><th>Position</th><th>Conservation</th><th>Predicted By</th></tr>
<tr><td>Offset 6mer
14076 14097
5'-gaCCTTTAGACTTTCAGTCATc-3' UTR
|||:| | | :||||||:
3'-ggGGAGACCAGUUGGUCAGUGu-5' miRNA
16 15 14 13 7 6 5 4 3 2
3'pairing Seed</td><td>CAGTCA
Offset 6mer</td><td></td><td>☐</td><td>M</td></tr>
<tr><td>Offset 6mer
18758 18778
5'-atCCTGCTGGGC--CCAGTCATt-3' UTR
||| |||| | |||||||:
3'-ggGGA-GACCAGUUGGUCAGUGu-5' miRNA
16 15 14 13 7 6 5 4 3 2
3'pairing Seed</td><td>CAGTCA
Offset 6mer</td><td></td><td>☐</td><td>M</td></tr>
<tr><td>7mer-m8
24546 24567
5'-caaCTCTCCTAATACAGTCACt-3' UTR
|||| | | |||||||
3'-gggGAGACCAGUUGGUCAGUGu-5' miRNA
16 15 14 13 7 6 5 4 3 2
3'pairing Seed</td><td>CAGTCAC
7mer-m8</td><td></td><td>☐</td><td>M T</td></tr>
<tr><td>6mer
26453 26475
5'-ctgCTGTGTGACCACTAGTCACt-3' UTR
|| || | | ||:||||||
3'-gggGAGAC-CAGUUGGUCAGUGu-5' miRNA
16 15 14 13 7 6 5 4 3 2
3'pairing Seed</td><td>AGTCAC
6mer</td><td></td><td>☐</td><td>M</td></tr>
<tr><td>Imperfect match
30827 30848
5'-tgggcCTGGTCAGTCAGTCGCt -3' UTR
|||||||::|||||:|
3'-ggggaGACCAGUUGGUCAGUGu-5' miRNA
16 15 14 13 7 6 5 4 3 2
3'pairing Seed</td><td>AGTCGC
Imperfect</td><td></td><td>☐</td><td>M</td></tr>
<tr><td>7mer-A1
44323 44343
5'-acaCCCTTCTCAA-TAGTCACa-3' UTR
| || |||| :||||||
3'-gggGAGACCAGUUGGUCAGUGu-5' miRNA
16 15 14 13 7 6 5 4 3 2
3'pairing Seed</td><td>TAGTCACA
7mer-A1</td><td></td><td>☐</td><td>M T</td></tr>
<tr><td>7mer-A1
64451 64471
5'-acaCCCTTCTCAA-TAGTCACa-3' UTR
| || |||| :||||||
3'-gggGAGACCAGUUGGUCAGUGu-5' miRNA
16 15 14 13 7 6 5 4 3 2
3'pairing Seed</td><td>TAGTCACA
7mer-A1</td><td></td><td>☐</td><td>M T</td></tr>
<tr><td>Imperfect match
72758 72779
5'-ggCTTCTGGACCTCCAGTCTCc -3' UTR
|:||||| | |||||| |
3'-ggGGAGACCAGUUGGUCAGUGu-5' miRNA
16 15 14 13 7 6 5 4 3 2
3'pairing Seed</td><td>AGTCTC
Imperfect</td><td></td><td>☐</td><td>M</td></tr>
<tr><td>Imperfect match
79586 79606
5'-cgCCTCT-CCCACTCAGTTACc -3' UTR
||||| || :||||:||
3'-ggGGAGACCAGUUGGUCAGUGu-5' miRNA
16 15 14 13 7 6 5 4 3 2
3'pairing Seed</td><td>AGTTAC
Imperfect</td><td></td><td>☐</td><td>M</td></tr>
<tr><td>7mer-m8
82257 82281
5'-caCCTGTGGATTAATTACAGTCACt-3' UTR
||| ||| |:||: |||||||
3'-ggGGAGACC-AGUUG--GUCAGUGu-5' miRNA
16 15 14 13 7 6 5 4 3 2
3'pairing Seed</td><td>CAGTCAC
7mer-m8</td><td></td><td>☐</td><td>M T</td></tr>
<tr><td>7mer-m8
85896 85917
5'-caatgCTTTTCTCTCAGTCACt-3' UTR
|| || :|||||||
3'-ggggaGACCAGUUGGUCAGUGu-5' miRNA
16 15 14 13 7 6 5 4 3 2
3'pairing Seed</td><td>CAGTCAC
7mer-m8</td><td></td><td>☐</td><td>M T</td></tr>
</table>

2D Structure	Local AU	Position	Conservation	Predicted By
Offset 6mer<br>1487 1508<br>5'-gaCCTCTGGAAGAGCAGTCAGt-3' UTR<br>||||||| :| |||||| 3'-ggGGAGACCAGUUGGUCAGUGu-5' miRNA<br>3'pairing Seed	CAGTCA<br>Offset 6mer			M
Imperfect match<br>6992 7012<br>5'-aggCTTT-ATAAGCCAGTTACa-3' UTR<br>||:| | |:|||||:|| 3'-gggGAGACCAGUUGGUCAGUGu-5' miRNA<br>3'pairing Seed	AGTTAC<br>Imperfect			M
7mer-m8<br>24738 24758<br>5'-tagCTCAGG-CTATCAGTCACt-3' UTR<br>||| || | |:||||||| 3'-gggGAGACCAGUUGGUCAGUGu-5' miRNA<br>3'pairing Seed	CAGTCAC<br>7mer-m8			M T
7mer-m8<br>24808 24828<br>5'-tagCTCAGG-CTATCAGTCACt-3' UTR<br>||| || | |:||||||| 3'-gggGAGACCAGUUGGUCAGUGu-5' miRNA<br>3'pairing Seed	CAGTCAC<br>7mer-m8			M T
7mer-m8<br>25014 25034<br>5'-tagCTCAGG-CTATCAGTCACt-3' UTR<br>||| || | |:||||||| 3'-gggGAGACCAGUUGGUCAGUGu-5' miRNA<br>3'pairing Seed	CAGTCAC<br>7mer-m8			M T
7mer-m8<br>25049 25069<br>5'-tagCTCAGG-CTATCAGTCACt-3' UTR<br>||| || | |:||||||| 3'-gggGAGACCAGUUGGUCAGUGu-5' miRNA<br>3'pairing Seed	CAGTCAC<br>7mer-m8			M T
7mer-m8<br>25216 25236<br>5'-tagCTCAGG-CTATCAGTCACt-3' UTR<br>||| || | |:||||||| 3'-gggGAGACCAGUUGGUCAGUGu-5' miRNA<br>3'pairing Seed	CAGTCAC<br>7mer-m8			M T
7mer-m8<br>25251 25271<br>5'-tagCTCAGG-CTATCAGTCACt-3' UTR<br>||| || | |:||||||| 3'-gggGAGACCAGUUGGUCAGUGu-5' miRNA<br>3'pairing Seed	CAGTCAC<br>7mer-m8			M T
7mer-m8<br>25418 25438<br>5'-tagCTCAGG-CTATCAGTCACt-3' UTR<br>||| || | |:||||||| 3'-gggGAGACCAGUUGGUCAGUGu-5' miRNA<br>3'pairing Seed	CAGTCAC<br>7mer-m8			M T
Offset 6mer<br>40719 40740<br>5'-gaCCTTTGGAAGAGCAGTCAGc-3' UTR<br>|||:||| :| |||||| 3'-ggGGAGACCAGUUGGUCAGUGu-5' miRNA<br>3'pairing Seed	CAGTCA<br>Offset 6mer			M
Imperfect match<br>44680 44702<br>5'-ttaTTCTGGTCAAAACAATCACt-3' UTR<br>:|||||| || || |||| 3'-gggGAGACCAG-UUGGUCAGUGu-5' miRNA<br>3'pairing Seed	AATCAC<br>Imperfect			M
Imperfect match<br>50894 50913<br>5'-tcCCACTGGTCG--CAGTTACt-3' UTR<br>|| ||||||: |||:|| 3'-ggGGAGACCAGUUGGUCAGUGu-5' miRNA<br>3'pairing Seed	AGTTAC<br>Imperfect			M
Offset 6mer<br>51572 51592<br>5'-agaCACT-GTTGGCCAGTCAGg-3' UTR<br>| || ||:::|||||| 3'-gggGAGACCAGUUGGUCAGUGu-5' miRNA<br>3'pairing Seed	CAGTCA<br>Offset 6mer			M
7mer-m8<br>65408 65430<br>5'-aaaCCCTGACTAAGACAGTCACt-3' UTR<br>| ||| | |: ||||||| 3'-gggGAGAC-CAGUUGGUCAGUGu-5' miRNA<br>3'pairing Seed	CAGTCAC<br>7mer-m8			M T
Offset 6mer<br>76222 76244<br>5'-ggTCTGTGAGTTCAGCAGTCATg-3' UTR<br>:|| || ||: | ||||||:<br>3'-ggGGAGAC-CAGUUGGUCAGUGu-5' miRNA<br>3'pairing Seed	CAGTCA<br>Offset 6mer			M
7mer-m8<br>76762 76784<br>5'-gtCCTAAGTATCTCTCAGTCACt-3' UTR<br>||| | || :||||||| 3'-ggGGAGAC-CAGUUGGUCAGUGu-5' miRNA<br>3'pairing Seed	CAGTCAC<br>7mer-m8			M T
Offset 6mer<br>77923 77943<br>5'-gttgcCT-GTCAACCAGTCAGt-3' UTR<br>|| |||||||||||| 3'-ggggaGACCAGUUGGUCAGUGu-5' miRNA<br>3'pairing Seed	CAGTCA<br>Offset 6mer			M
Imperfect match<br>87014 87035<br>5'-tgCCTCTATTTAATTAGTCATa-3' UTR<br>|||| |:||::|||||:<br>3'-ggGGAGACCAGUUGGUCAGUGu-5' miRNA<br>3'pairing Seed	AGTCAT<br>Imperfect			M
7mer-m8<br>89236 89255<br>5'-ttTTTCTAATCA--CAGTCACt-3' UTR<br>::||| ||| ||||||| 3'-ggGGAGACCAGUUGGUCAGUGu-5' miRNA<br>3'pairing Seed	CAGTCAC<br>7mer-m8			M T

<table>
<tr><th>2D Structure</th><th>Local AU</th><th>Position</th><th>Conservation</th><th>Predicted By</th></tr>
<tr><td>Offset 6mer
5225 5246
5'-gaCCTCTGGAAGAGCAGTCAgt-3' UTR
3'-ggGGAGACCAGUUGGUCAGUGu-5' miRNA
16 15 14 13 7 6 5 4 3 2
3'pairing Seed</td><td>CAGTCA
Offset 6mer</td><td></td><td>☐</td><td>M</td></tr>
<tr><td>Imperfect match
10730 10750
5'-aggCTTT-ATAAGCCAGTTACa -3' UTR
3'-gggGAGACCAGUUGGUCAGUGu-5' miRNA
16 15 14 13 7 6 5 4 3 2
3'pairing Seed</td><td>AGTTAC
Imperfect</td><td></td><td>☐</td><td>M</td></tr>
<tr><td>7mer-m8
28476 28496
5'-tagCTCAGG-CTATCAGTCACt-3' UTR
3'-gggGAGACCAGUUGGUCAGUGu-5' miRNA
16 15 14 13 7 6 5 4 3 2
3'pairing Seed</td><td>CAGTCAC
7mer-m8</td><td></td><td>☐</td><td>M T</td></tr>
<tr><td>7mer-m8
28546 28566
5'-tagCTCAGG-CTATCAGTCACt-3' UTR
3'-gggGAGACCAGUUGGUCAGUGu-5' miRNA
16 15 14 13 7 6 5 4 3 2
3'pairing Seed</td><td>CAGTCAC
7mer-m8</td><td></td><td>☐</td><td>M T</td></tr>
<tr><td>7mer-m8
28752 28772
5'-tagCTCAGG-CTATCAGTCACt-3' UTR
3'-gggGAGACCAGUUGGUCAGUGu-5' miRNA
16 15 14 13 7 6 5 4 3 2
3'pairing Seed</td><td>CAGTCAC
7mer-m8</td><td></td><td>☐</td><td>M T</td></tr>
<tr><td>7mer-m8
28787 28807
5'-tagCTCAGG-CTATCAGTCACt-3' UTR
3'-gggGAGACCAGUUGGUCAGUGu-5' miRNA
16 15 14 13 7 6 5 4 3 2
3'pairing Seed</td><td>CAGTCAC
7mer-m8</td><td></td><td>☐</td><td>M T</td></tr>
<tr><td>7mer-m8
28954 28974
5'-tagCTCAGG-CTATCAGTCACt-3' UTR
3'-gggGAGACCAGUUGGUCAGUGu-5' miRNA
16 15 14 13 7 6 5 4 3 2
3'pairing Seed</td><td>CAGTCAC
7mer-m8</td><td></td><td>☐</td><td>M T</td></tr>
<tr><td>7mer-m8
28989 29009
5'-tagCTCAGG-CTATCAGTCACt-3' UTR
3'-gggGAGACCAGUUGGUCAGUGu-5' miRNA
16 15 14 13 7 6 5 4 3 2
3'pairing Seed</td><td>CAGTCAC
7mer-m8</td><td></td><td>☐</td><td>M T</td></tr>
<tr><td>7mer-m8
29156 29176
5'-tagCTCAGG-CTATCAGTCACt-3' UTR
3'-gggGAGACCAGUUGGUCAGUGu-5' miRNA
16 15 14 13 7 6 5 4 3 2
3'pairing Seed</td><td>CAGTCAC
7mer-m8</td><td></td><td>☐</td><td>M T</td></tr>
<tr><td>Offset 6mer
44457 44478
5'-gaCCTTTGGAAGAGCAGTCAgc-3' UTR
3'-ggGGAGACCAGUUGGUCAGUGu-5' miRNA
16 15 14 13 7 6 5 4 3 2
3'pairing Seed</td><td>CAGTCA
Offset 6mer</td><td></td><td>☐</td><td>M</td></tr>
<tr><td>Imperfect match
48418 48440
5'-ttaTTCTGGTCAAAACAATCACt -3' UTR
3'-gggGAGACCAG-UUGGUCAGUGu-5' miRNA
16 15 14 13 7 6 5 4 3 2
3'pairing Seed</td><td>AATCAC
Imperfect</td><td></td><td>☐</td><td>M</td></tr>
<tr><td>Imperfect match
54632 54651
5'-tcCCACTGGTCG--CAGTTACt -3' UTR
3'-ggGGAGACCAGUUGGUCAGUGu-5' miRNA
16 15 14 13 7 6 5 4 3 2
3'pairing Seed</td><td>AGTTAC
Imperfect</td><td></td><td>☐</td><td>M</td></tr>
<tr><td>Offset 6mer
55310 55330
5'-agaCACT-GTTGGCCAGTCAgg-3' UTR
3'-gggGAGACCAGUUGGUCAGUGu-5' miRNA
16 15 14 13 7 6 5 4 3 2
3'pairing Seed</td><td>CAGTCA
Offset 6mer</td><td></td><td>☐</td><td>M</td></tr>
<tr><td>7mer-m8
69146 69168
5'-aaaCCCTGACTAAGACAGTCACt-3' UTR
3'-gggGAGAC-CAGUUGGUCAGUGu-5' miRNA
16 15 14 13 7 6 5 4 3 2
3'pairing Seed</td><td>CAGTCAC
7mer-m8</td><td></td><td>☐</td><td>M T</td></tr>
<tr><td>Offset 6mer
79960 79982
5'-ggTCTGTGAGTTCAGCAGTCATg-3' UTR
3'-ggGGAGAC-CAGUUGGUCAGUGu-5' miRNA
16 15 14 13 7 6 5 4 3 2
3'pairing Seed</td><td>CAGTCA
Offset 6mer</td><td></td><td>☐</td><td>M</td></tr>
<tr><td>7mer-m8
80500 80522
5'-gtCCTAAGTATCTCTCAGTCACt-3' UTR
3'-ggGGAGAC-CAGUUGGUCAGUGu-5' miRNA
16 15 14 13 7 6 5 4 3 2
3'pairing Seed</td><td>CAGTCAC
7mer-m8</td><td></td><td>☐</td><td>M T</td></tr>
<tr><td>Offset 6mer
81661 81681
5'-gttgcCT-GTCAACCAGTCAgt-3' UTR
3'-ggggaGACCAGUUGGUCAGUGu-5' miRNA
16 15 14 13 7 6 5 4 3 2
3'pairing Seed</td><td>CAGTCA
Offset 6mer</td><td></td><td>☐</td><td>M</td></tr>
<tr><td>Imperfect match
90752 90773
5'-tgCCTCTATTTAATTAGTCATa -3' UTR
3'-ggGGAGACCAGUUGGUCAGUGu-5' miRNA
16 15 14 13 7 6 5 4 3 2
3'pairing Seed</td><td>AGTCAT
Imperfect</td><td></td><td>☐</td><td>M</td></tr>
<tr><td>7mer-m8
92974 92993
5'-ttTTTCTAATCA--CAGTCACt-3' UTR
3'-ggGGAGACCAGUUGGUCAGUGu-5' miRNA
16 15 14 13 7 6 5 4 3 2
3'pairing Seed</td><td>CAGTCAC
7mer-m8</td><td></td><td>☐</td><td>M T</td></tr>
</table>

<table>
<tr><th>2D Structure</th><th>Local AU</th><th>Position</th><th>Conservation</th><th>Predicted By</th></tr>
<tr><td>Offset 6mer
24544 24565
5'-aaCTCCAGGGCATGCAGTCATc-3' UTR
|: | || || ||||||:
3'-ggGGAGACCAGUUGGUCAGUGu-5' miRNA
16 15 14 13 7 6 5 4 3 2
3'pairing Seed</td><td>CAGTCA
Offset 6mer</td><td></td><td></td><td>M</td></tr>
<tr><td>Offset 6mer
26489 26510
5'-gggCTTTTGTTATTCAGTCAgc-3' UTR
||:| ||:| :||||||
3'-gggGAGACCAGUUGGUCAGUgu-5' miRNA
16 15 14 13 7 6 5 4 3 2
3'pairing Seed</td><td>CAGTCA
Offset 6mer</td><td></td><td></td><td>M</td></tr>
<tr><td>Offset 6mer
31400 31421
5'-gaCCTCTGGAAGAGCAGTCAat-3' UTR
||||||| :| ||||||
3'-ggGGAGACCAGUUGGUCAGUgu-5' miRNA
16 15 14 13 7 6 5 4 3 2
3'pairing Seed</td><td>CAGTCA
Offset 6mer</td><td></td><td></td><td>M</td></tr>
<tr><td>Imperfect match
37737 37759
5'-tctgTGTGGTTATGCCAGTTACt -3' UTR
| ||||:| :||||||:||
3'-ggggAGACCAGU-UGGUCAGUGu-5' miRNA
16 15 14 13 7 6 5 4 3 2
3'pairing Seed</td><td>AGTTAC
Imperfect</td><td></td><td></td><td>M</td></tr>
<tr><td>Imperfect match
43153 43174
5'-ctgtgtaGGTAAATCAGTTACt -3' UTR
||| ||:||||:||
3'-ggggagaCCAGUUGGUCAGUGu-5' miRNA
16 15 14 13 7 6 5 4 3 2
3'pairing Seed</td><td>AGTTAC
Imperfect</td><td></td><td></td><td>M</td></tr>
<tr><td>6mer
44509 44531
5'-ttttaaaGGTCTAATTAGTCACc-3' UTR
|||| ||::||||||
3'-ggggagaCCAG-UUGGUCAGUGu-5' miRNA
16 15 14 13 7 6 5 4 3 2
3'pairing Seed</td><td>AGTCAC
6mer</td><td></td><td></td><td>M</td></tr>
<tr><td>6mer
47665 47687
5'-tcCCTATGGGAAACATAGTCACt-3' UTR
||| ||| ||| :||||||
3'-ggGGAGACCAGUUG-GUCAGUGu-5' miRNA
16 15 14 13 7 6 5 4 3 2
3'pairing Seed</td><td>AGTCAC
6mer</td><td></td><td></td><td>M</td></tr>
<tr><td>Imperfect match
51657 51677
5'-tgCTTGTGGGT-ACCAGTTACt -3' UTR
|:| ||| : ||||||:||
3'-ggGGAGACCAGUUGGUCAGUGu-5' miRNA
16 15 14 13 7 6 5 4 3 2
3'pairing Seed</td><td>AGTTAC
Imperfect</td><td></td><td></td><td>M</td></tr>
<tr><td>Offset 6mer
63198 63219
5'-taCTTCTACTTACACAGTCAac-3' UTR
|:||| |:| ||||||
3'-ggGGAGACCAGUUGGUCAGUgu-5' miRNA
16 15 14 13 7 6 5 4 3 2
3'pairing Seed</td><td>CAGTCA
Offset 6mer</td><td></td><td></td><td>M</td></tr>
<tr><td>Imperfect match
63656 63677
5'-catagTTTGTCTATCAGTTACt -3' UTR
:| ||| |:||||:||
3'-ggggaGACCAGUUGGUCAGUGu-5' miRNA
16 15 14 13 7 6 5 4 3 2
3'pairing Seed</td><td>AGTTAC
Imperfect</td><td></td><td></td><td>M</td></tr>
<tr><td>Offset 6mer
65300 65321
5'-aaggTTTATTTAAGCAGTCATt-3' UTR
|:| |:|| ||||||:
3'-ggggAGACCAGUUGGUCAGUGu-5' miRNA
16 15 14 13 7 6 5 4 3 2
3'pairing Seed</td><td>CAGTCA
Offset 6mer</td><td></td><td></td><td>M</td></tr>
<tr><td>7mer-m8
65311 65333
5'-aagcagtcaTTATACCAGTCACt-3' UTR
|:| |||||||||
3'-ggggagaccAGU-UGGUCAGUGu-5' miRNA
16 15 14 13 7 6 5 4 3 2
3'pairing Seed</td><td>CAGTCAC
7mer-m8</td><td></td><td></td><td>M T</td></tr>
<tr><td>8mer
70768 70791
5'-tgctatattTCAATATCAGTCACa-3' UTR
||| |:|||||||
3'-ggggagaccAGU--UGGUCAGUGu-5' miRNA
16 15 14 13 7 6 5 4 3 2
3'pairing Seed</td><td>CAGTCACA
8mer</td><td></td><td></td><td>M T</td></tr>
<tr><td>Imperfect match
76167 76188
5'-tgCCTTTGGGAAAACATTCACa -3' UTR
|||:||| || || ||||
3'-ggGGAGACCAGUUGGUCAGUGu-5' miRNA
16 15 14 13 7 6 5 4 3 2
3'pairing Seed</td><td>ATTCAC
Imperfect</td><td></td><td></td><td>M</td></tr>
<tr><td>8mer
87977 88004
5'-acaTTCTGATCTAACTTTTACAGTCACa-3' UTR
:|||| || ||| |||||||
3'-gggGAGACCAG-UUG-----GUCAGUGu-5' miRNA
16 15 14 13 7 6 5 4 3 2
3'pairing Seed</td><td>CAGTCACA
8mer</td><td></td><td></td><td>M T</td></tr>
</table>

<table>
<tr><th>2D Structure</th><th>Local AU</th><th>Position</th><th>Conservation</th><th>Predicted By</th></tr>
<tr><td>Offset 6mer
1161 1183
5'-ttataCTGTGTGTATCAGTCATt-3' UTR
||| || |:|||||:
3'-ggggaGAC-CAGUUGGUCAGUGu-5' miRNA
16 15 14 13 7 6 5 4 3 2
3'pairing Seed</td><td>CAGTCA
Offset 6mer</td><td></td><td>☐</td><td>M</td></tr>
<tr><td>Offset 6mer
3938 3957
5'-gcCCTTTGGT--GCCAGTCATt-3' UTR
|||:|||| :|||||||:
3'-ggGGAGACCAGUUGGUCAGUGu-5' miRNA
16 15 14 13 7 6 5 4 3 2
3'pairing Seed</td><td>CAGTCA
Offset 6mer</td><td></td><td>☐</td><td>M</td></tr>
<tr><td>7mer-A1
22152 22174
5'-aaataTTGGTATAGGTAGTCACa-3' UTR
:|||| :|: :||||||
3'-ggggaGACCA-GUUGGUCAGUGu-5' miRNA
16 15 14 13 7 6 5 4 3 2
3'pairing Seed</td><td>TAGTCACA
7mer-A1</td><td></td><td>☐</td><td>M T</td></tr>
<tr><td>Offset 6mer
42143 42164
5'-ttTCTGTGGAGCATCAGTCATg-3' UTR
:|| ||| |:|||||:
3'-ggGGAGACCAGUUGGUCAGUGu-5' miRNA
16 15 14 13 7 6 5 4 3 2
3'pairing Seed</td><td>CAGTCA
Offset 6mer</td><td></td><td>☐</td><td>M</td></tr>
<tr><td>Imperfect match
45654 45675
5'-tgagagTGCTGAGCCAGTTACt -3' UTR
|| | |:||||:||
3'-ggggagACCAGUUGGUCAGUGu-5' miRNA
16 15 14 13 7 6 5 4 3 2
3'pairing Seed</td><td>AGTTAC
Imperfect</td><td></td><td>☐</td><td>M</td></tr>
<tr><td>Offset 6mer
47184 47205
5'-atcaatcaaTCAATCAGTCAgt-3' UTR
||||:||||||
3'-ggggagaccAGUUGGUCAGUgu-5' miRNA
16 15 14 13 7 6 5 4 3 2
3'pairing Seed</td><td>CAGTCA
Offset 6mer</td><td></td><td>☐</td><td>M</td></tr>
<tr><td>8mer
57963 57986
5'-ctgtgtactTCAGTGTCAGTCACa-3' UTR
||| ::|||||||
3'-ggggagaccAGU--UGGUCAGUGu-5' miRNA
16 15 14 13 7 6 5 4 3 2
3'pairing Seed</td><td>CAGTCACA
8mer</td><td></td><td>☐</td><td>M T</td></tr>
<tr><td>Imperfect match
61073 61095
5'-gataaaTGGGCGAATCAGTTACt -3' UTR
||| | ||:||||:||
3'-ggggagACCAG-UUGGUCAGUGu-5' miRNA
16 15 14 13 7 6 5 4 3 2
3'pairing Seed</td><td>AGTTAC
Imperfect</td><td></td><td>☐</td><td>M</td></tr>
<tr><td>Imperfect match
62237 62258
5'-tatataaGGTAATCCAGTTACc -3' UTR
||| | |||||:||
3'-ggggagaCCAGUUGGUCAGUGu-5' miRNA
16 15 14 13 7 6 5 4 3 2
3'pairing Seed</td><td>AGTTAC
Imperfect</td><td></td><td>☐</td><td>M</td></tr>
<tr><td>Offset 6mer
63140 63161
5'-gcataCTGGTCTAGCAGTCATc-3' UTR
|||||| | ||||||:
3'-ggggaGACCAGUUGGUCAGUGu-5' miRNA
16 15 14 13 7 6 5 4 3 2
3'pairing Seed</td><td>CAGTCA
Offset 6mer</td><td></td><td>☐</td><td>M</td></tr>
<tr><td>7mer-m8
66221 66242
5'-catcatactTCATTCAGTCACg-3' UTR
||| :|||||||
3'-ggggagaccAGUUGGUCAGUGu-5' miRNA
16 15 14 13 7 6 5 4 3 2
3'pairing Seed</td><td>CAGTCAC
7mer-m8</td><td></td><td>☐</td><td>M T</td></tr>
<tr><td>6mer
72992 73016
5'-atCCTCTCCCGGTCTCATAGTCACt-3' UTR
|||| |||| :||||||
3'-ggGGAGA---CCAGUUGGUCAGUGu-5' miRNA
16 15 14 13 7 6 5 4 3 2
3'pairing Seed</td><td>AGTCAC
6mer</td><td></td><td>☐</td><td>M</td></tr>
<tr><td>7mer-m8
81033 81055
5'-caaacTTGCTTGAACCAGTCACc-3' UTR
:|| |: |||||||||
3'-ggggaGACCAG-UUGGUCAGUGu-5' miRNA
16 15 14 13 7 6 5 4 3 2
3'pairing Seed</td><td>CAGTCAC
7mer-m8</td><td></td><td>☐</td><td>M T</td></tr>
</table>

2D Structure	Local AU	Position	Conservation	Predicted By
7243 7mer-m8 7264<br>5'-aacCCTGACT-AAGGCACCAGCt-3' UTR<br>3'-gccGGACUAAGUGUUGUGGUCGa-5' miRNA<br>16 15 14 13 7 6 5 4 3 2<br>3'pairing Seed	CACCAGC<br>7mer-m8			M T
8844 7mer-m8 8866<br>5'-ggGTGTCATAGAAGCCACCAGCt-3' UTR<br>3'-gcCGGACUAAGUGUUGUGGUCGa-5' miRNA<br>16 15 14 13 7 6 5 4 3 2<br>3'pairing Seed	CACCAGC<br>7mer-m8			M T
13581 Imperfect match 13605<br>5'-agGCACTGATTCAAAATCATCAGCc -3' UTR<br>3'-gcCG-GACUAAGUGUU-GUGGUCGa-5' miRNA<br>16 15 14 13 7 6 5 4 3 2<br>3'pairing Seed	ATCAGC<br>Imperfect			M
23345 7mer-m8 23367<br>5'-ataatgctgggAGATCACCAGCt-3' UTR<br>3'-gccggacuaagUGUUGUGGUCGa-5' miRNA<br>16 15 14 13 7 6 5 4 3 2<br>3'pairing Seed	CACCAGC<br>7mer-m8			M T
27230 Imperfect match 27252<br>5'-cacCCCCATTTGGGGCATCAGCa -3' UTR<br>3'-gccGGACUAAGUGUUGUGGUCGa-5' miRNA<br>16 15 14 13 7 6 5 4 3 2<br>3'pairing Seed	ATCAGC<br>Imperfect			M
30945 7mer-m8 30969<br>5'-ggcCCAGGTCCTGCTAACACCAGCt-3' UTR<br>3'-gccGGACUAAG-UG-UUGUGGUCGa-5' miRNA<br>16 15 14 13 7 6 5 4 3 2<br>3'pairing Seed	CACCAGC<br>7mer-m8			M T
32809 Imperfect match 32831<br>5'-ctcaggGGTTAGGAGCACCGGCt -3' UTR<br>3'-gccggaCUAAGUGUUGUGGUCGa-5' miRNA<br>16 15 14 13 7 6 5 4 3 2<br>3'pairing Seed	ACCGGC<br>Imperfect			M
40958 Imperfect match 40981<br>5'-acGTTTAACTTTAATACGCCAGCa -3' UTR<br>3'-gcCGGACU-AAGUGUUGUGGUCGa-5' miRNA<br>16 15 14 13 7 6 5 4 3 2<br>3'pairing Seed	GCCAGC<br>Imperfect			M
47856 Imperfect match 47878<br>5'-tccaaaGATTC-CAGTCATCAGCt -3' UTR<br>3'-gccggaCUAAGUGUU-GUGGUCGa-5' miRNA<br>16 15 14 13 7 6 5 4 3 2<br>3'pairing Seed	ATCAGC<br>Imperfect			M
60006 7mer-m8 60025<br>5'-gacaCTGAT---CGTCACCAGCc-3' UTR<br>3'-gccgGACUAAGUGUUGUGGUCGa-5' miRNA<br>16 15 14 13 7 6 5 4 3 2<br>3'pairing Seed	CACCAGC<br>7mer-m8			M T
60509 8mer 60533<br>5'-ggcCTTGAACCTAGGACCACCAGCa-3' UTR<br>3'-gccGGACUAAG-UGUU-GUGGUCGa-5' miRNA<br>16 15 14 13 7 6 5 4 3 2<br>3'pairing Seed	CACCAGCA<br>8mer			M T
64957 Imperfect match 64979<br>5'-ttctgTGATTGGGGGCATCAGCa -3' UTR<br>3'-gccggACUAAGUGUUGUGGUCGa-5' miRNA<br>16 15 14 13 7 6 5 4 3 2<br>3'pairing Seed	ATCAGC<br>Imperfect			M
73737 7mer-m8 73756<br>5'-gacaCTGATTT---TCACCAGCc-3' UTR<br>3'-gccgGACUAAGUGUUGUGGUCGa-5' miRNA<br>16 15 14 13 7 6 5 4 3 2<br>3'pairing Seed	CACCAGC<br>7mer-m8			M T
80628 Imperfect match 80650<br>5'-taGGCTGACTGAGACCATCAGCc -3' UTR<br>3'-gcCGGACUAAGUGUUGUGGUCGa-5' miRNA<br>16 15 14 13 7 6 5 4 3 2<br>3'pairing Seed	ATCAGC<br>Imperfect			M

<table>
<tr><th>2D Structure</th><th>Local AU</th><th>Position</th><th>Conservation</th><th>Predicted By</th></tr>
<tr><td>Imperfect match
736 758
5'-cacTTTGATTCATAACACCAagg -3' UTR
::|||||||:|||||||
3'-gccGGACUAAGUGUUGUGGUcga-5' miRNA
16 15 14 13 7 6 5 4 3 2
3'pairing Seed</td><td>ACCAAG
Imperfect</td><td></td><td></td><td>M</td></tr>
<tr><td>Imperfect match
2609 2631
5'-cacTTTGATTCATAACACCAagg -3' UTR
::|||||||:|||||||
3'-gccGGACUAAGUGUUGUGGUcga-5' miRNA
16 15 14 13 7 6 5 4 3 2
3'pairing Seed</td><td>ACCAAG
Imperfect</td><td></td><td></td><td>M</td></tr>
<tr><td>Imperfect match
4482 4504
5'-cacTTTGATTCATAACACCAagg -3' UTR
::|||||||:|||||||
3'-gccGGACUAAGUGUUGUGGUcga-5' miRNA
16 15 14 13 7 6 5 4 3 2
3'pairing Seed</td><td>ACCAAG
Imperfect</td><td></td><td></td><td>M</td></tr>
<tr><td>Imperfect match
6355 6377
5'-cacTTTGATTCATAACACCAagg -3' UTR
::|||||||:|||||||
3'-gccGGACUAAGUGUUGUGGUcga-5' miRNA
16 15 14 13 7 6 5 4 3 2
3'pairing Seed</td><td>ACCAAG
Imperfect</td><td></td><td></td><td>M</td></tr>
<tr><td>Imperfect match
8228 8250
5'-cacTTTGATTCATAACACCAagg -3' UTR
::|||||||:|||||||
3'-gccGGACUAAGUGUUGUGGUcga-5' miRNA
16 15 14 13 7 6 5 4 3 2
3'pairing Seed</td><td>ACCAAG
Imperfect</td><td></td><td></td><td>M</td></tr>
<tr><td>Imperfect match
10100 10122
5'-cacTTTGATTCATAACACCAagg -3' UTR
::|||||||:|||||||
3'-gccGGACUAAGUGUUGUGGUcga-5' miRNA
16 15 14 13 7 6 5 4 3 2
3'pairing Seed</td><td>ACCAAG
Imperfect</td><td></td><td></td><td>M</td></tr>
<tr><td>Imperfect match
11973 11995
5'-cacTTTGATTCATAACACCAagg -3' UTR
::|||||||:|||||||
3'-gccGGACUAAGUGUUGUGGUcga-5' miRNA
16 15 14 13 7 6 5 4 3 2
3'pairing Seed</td><td>ACCAAG
Imperfect</td><td></td><td></td><td>M</td></tr>
<tr><td>Imperfect match
13846 13868
5'-cacTTTGATTCATAACACCAagg -3' UTR
::|||||||:|||||||
3'-gccGGACUAAGUGUUGUGGUcga-5' miRNA
16 15 14 13 7 6 5 4 3 2
3'pairing Seed</td><td>ACCAAG
Imperfect</td><td></td><td></td><td>M</td></tr>
<tr><td>Imperfect match
15718 15740
5'-cacTTTGATTCATAACACCAagg -3' UTR
::|||||||:|||||||
3'-gccGGACUAAGUGUUGUGGUcga-5' miRNA
16 15 14 13 7 6 5 4 3 2
3'pairing Seed</td><td>ACCAAG
Imperfect</td><td></td><td></td><td>M</td></tr>
<tr><td>Imperfect match
17590 17612
5'-cacTTTGATTCATAACACCAagg -3' UTR
::|||||||:|||||||
3'-gccGGACUAAGUGUUGUGGUcga-5' miRNA
16 15 14 13 7 6 5 4 3 2
3'pairing Seed</td><td>ACCAAG
Imperfect</td><td></td><td></td><td>M</td></tr>
<tr><td>Imperfect match
19460 19482
5'-cacTTTGATTCATAACACCAagt -3' UTR
::|||||||:|||||||
3'-gccGGACUAAGUGUUGUGGUcga-5' miRNA
16 15 14 13 7 6 5 4 3 2
3'pairing Seed</td><td>ACCAAG
Imperfect</td><td></td><td></td><td>M</td></tr>
<tr><td>Imperfect match
21340 21362
5'-cacTTTGATTCATAACACCAagg -3' UTR
::|||||||:|||||||
3'-gccGGACUAAGUGUUGUGGUcga-5' miRNA
16 15 14 13 7 6 5 4 3 2
3'pairing Seed</td><td>ACCAAG
Imperfect</td><td></td><td></td><td>M</td></tr>
<tr><td>Imperfect match
23212 23234
5'-cacTTTGATTCATAACACCAagg -3' UTR
::|||||||:|||||||
3'-gccGGACUAAGUGUUGUGGUcga-5' miRNA
16 15 14 13 7 6 5 4 3 2
3'pairing Seed</td><td>ACCAAG
Imperfect</td><td></td><td></td><td>M</td></tr>
<tr><td>Imperfect match
25085 25107
5'-cacTTTGATTCATAACACCAagg -3' UTR
::|||||||:|||||||
3'-gccGGACUAAGUGUUGUGGUcga-5' miRNA
16 15 14 13 7 6 5 4 3 2
3'pairing Seed</td><td>ACCAAG
Imperfect</td><td></td><td></td><td>M</td></tr>
<tr><td>Imperfect match
26957 26979
5'-cacTTTGATTCATAACACCAagg -3' UTR
::|||||||:|||||||
3'-gccGGACUAAGUGUUGUGGUcga-5' miRNA
16 15 14 13 7 6 5 4 3 2
3'pairing Seed</td><td>ACCAAG
Imperfect</td><td></td><td></td><td>M</td></tr>
<tr><td>Imperfect match
28829 28851
5'-cacTTTGATTCATAACACCAagg -3' UTR
::|||||||:|||||||
3'-gccGGACUAAGUGUUGUGGUcga-5' miRNA
16 15 14 13 7 6 5 4 3 2
3'pairing Seed</td><td>ACCAAG
Imperfect</td><td></td><td></td><td>M</td></tr>
<tr><td>Imperfect match
30700 30722
5'-cacTTTGATTCATAACACCAagg -3' UTR
::|||||||:|||||||
3'-gccGGACUAAGUGUUGUGGUcga-5' miRNA
16 15 14 13 7 6 5 4 3 2
3'pairing Seed</td><td>ACCAAG
Imperfect</td><td></td><td></td><td>M</td></tr>
<tr><td>Imperfect match
32571 32593
5'-cacTTTGATTCATAACACCAagg -3' UTR
::|||||||:|||||||
3'-gccGGACUAAGUGUUGUGGUcga-5' miRNA
16 15 14 13 7 6 5 4 3 2
3'pairing Seed</td><td>ACCAAG
Imperfect</td><td></td><td></td><td>M</td></tr>
<tr><td>Imperfect match
34442 34464
5'-cacTTTGATTCATAACACCAagg -3' UTR
::|||||||:|||||||
3'-gccGGACUAAGUGUUGUGGUcga-5' miRNA
16 15 14 13 7 6 5 4 3 2
3'pairing Seed</td><td>ACCAAG
Imperfect</td><td></td><td></td><td>M</td></tr>
<tr><td>Imperfect match
36312 36334
5'-cacTTTGATTCATAACACCAagg -3' UTR
::|||||||:|||||||
3'-gccGGACUAAGUGUUGUGGUcga-5' miRNA
16 15 14 13 7 6 5 4 3 2
3'pairing Seed</td><td>ACCAAG
Imperfect</td><td></td><td></td><td>M</td></tr>
<tr><td>Imperfect match
38184 38206
5'-cacTTTGATTCATAACACCAagg -3' UTR
::|||||||:|||||||
3'-gccGGACUAAGUGUUGUGGUcga-5' miRNA
16 15 14 13 7 6 5 4 3 2
3'pairing Seed</td><td>ACCAAG
Imperfect</td><td></td><td></td><td>M</td></tr>
<tr><td>Imperfect match
40056 40078
5'-cacTTTGATTCATAACACCAagg -3' UTR
::|||||||:|||||||
3'-gccGGACUAAGUGUUGUGGUcga-5' miRNA
16 15 14 13 7 6 5 4 3 2
3'pairing Seed</td><td>ACCAAG
Imperfect</td><td></td><td></td><td>M</td></tr>
<tr><td>Imperfect match
41928 41950
5'-cacTTTGATTCATAACACCAcgg -3' UTR
::|||||||:|||||||
3'-gccGGACUAAGUGUUGUGGUcga-5' miRNA
16 15 14 13 7 6 5 4 3 2
3'pairing Seed</td><td>ACCACG
Imperfect</td><td></td><td></td><td>M</td></tr>
<tr><td>Imperfect match
43800 43822
5'-cacTTTGATTCATAACACCAagg -3' UTR
::|||||||:|||||||
3'-gccGGACUAAGUGUUGUGGUcga-5' miRNA
16 15 14 13 7 6 5 4 3 2
3'pairing Seed</td><td>ACCAAG
Imperfect</td><td></td><td></td><td>M</td></tr>
<tr><td>Imperfect match
45672 45694
5'-cacTTTGATTCATAACACCAagg -3' UTR
::|||||||:|||||||
3'-gccGGACUAAGUGUUGUGGUcga-5' miRNA
16 15 14 13 7 6 5 4 3 2
3'pairing Seed</td><td>ACCAAG
Imperfect</td><td></td><td></td><td>M</td></tr>
<tr><td>Imperfect match
47544 47566
5'-cacTTTGATTCATAACACCAagg -3' UTR
::|||||||:|||||||
3'-gccGGACUAAGUGUUGUGGUcga-5' miRNA
16 15 14 13 7 6 5 4 3 2
3'pairing Seed</td><td>ACCAAG
Imperfect</td><td></td><td></td><td>M</td></tr>
<tr><td>Imperfect match
49416 49438
5'-cacTTTGATTCATAACACCAagg -3' UTR
::|||||||:|||||||
3'-gccGGACUAAGUGUUGUGGUcga-5' miRNA
16 15 14 13 7 6 5 4 3 2
3'pairing Seed</td><td>ACCAAG
Imperfect</td><td></td><td></td><td>M</td></tr>
<tr><td>Imperfect match
51288 51310
5'-cacTTTGATTCATAACACCAagg -3' UTR
::|||||||:|||||||
3'-gccGGACUAAGUGUUGUGGUcga-5' miRNA
16 15 14 13 7 6 5 4 3 2
3'pairing Seed</td><td>ACCAAG
Imperfect</td><td></td><td></td><td>M</td></tr>
<tr><td>Imperfect match
53160 53182
5'-cacTTTGATTCATAACACCAagg -3' UTR
::|||||||:|||||||
3'-gccGGACUAAGUGUUGUGGUcga-5' miRNA
16 15 14 13 7 6 5 4 3 2
3'pairing Seed</td><td>ACCAAG
Imperfect</td><td></td><td></td><td>M</td></tr>
<tr><td>Imperfect match
55032 55054
5'-cacTTTGATTCATAACACCAagg -3' UTR
::|||||||:|||||||
3'-gccGGACUAAGUGUUGUGGUcga-5' miRNA
16 15 14 13 7 6 5 4 3 2
3'pairing Seed</td><td>ACCAAG
Imperfect</td><td></td><td></td><td>M</td></tr>
<tr><td>Imperfect match
56905 56927
5'-cacTTTGATTCATAACACCAagg -3' UTR
::|||||||:|||||||
3'-gccGGACUAAGUGUUGUGGUcga-5' miRNA
16 15 14 13 7 6 5 4 3 2
3'pairing Seed</td><td>ACCAAG
Imperfect</td><td></td><td></td><td>M</td></tr>
<tr><td>Imperfect match
58765 58787
5'-cacTTTGATTCATAACACCAagg -3' UTR
::|||||||:|||||||
3'-gccGGACUAAGUGUUGUGGUcga-5' miRNA
16 15 14 13 7 6 5 4 3 2
3'pairing Seed</td><td>ACCAAG
Imperfect</td><td></td><td></td><td>M</td></tr>
<tr><td>Imperfect match
60632 60654
5'-cacTTTGATTCATAACACCAagg -3' UTR
::|||||||:|||||||
3'-gccGGACUAAGUGUUGUGGUcga-5' miRNA
16 15 14 13 7 6 5 4 3 2
3'pairing Seed</td><td>ACCAAG
Imperfect</td><td></td><td></td><td>M</td></tr>
</table>

2D Structure	Local AU	Position	Conservation	Predicted By
Imperfect match<br>1450 1471<br>5'-tgGCCTG--TCACAGACACCACCa -3' UTR<br>3'-gcCGGACUAAGUGU-UGUGGUCGa-5' miRNA<br>3'pairing Seed	ACCACC<br>Imperfect		☐	M
Imperfect match<br>2309 2334<br>5'-tccCTTTATTTGCCCAATCATCAGCt -3' UTR<br>3'-gccGGACUAA--GUGUU-GUGGUCGa-5' miRNA<br>3'pairing Seed	ATCAGC<br>Imperfect		☐	M
8mer<br>3154 3177<br>5'-tgGCGGAATGTACTACCACCAGCa-3' UTR<br>3'-gcCGGACUAAGUG-UUGUGGUCGa-5' miRNA<br>3'pairing Seed	CACCAGCA<br>8mer		☐	M T
8mer<br>3765 3786<br>5'-ttggtccATTTA-AACACCAGCa-3' UTR<br>3'-gccggacUAAGUGUUGUGGUCGa-5' miRNA<br>3'pairing Seed	CACCAGCA<br>8mer		☐	M T
7mer-m8<br>8491 8515<br>5'-taaCTTAAGTGATAACACACCAGCt-3' UTR<br>3'-gccGGACUAAGUGU--UGUGGUCGa-5' miRNA<br>3'pairing Seed	CACCAGC<br>7mer-m8		☐	M T
Imperfect match<br>9437 9460<br>5'-gctCATTATTCAGTCACACTAGCa -3' UTR<br>3'-gccGGACUAAGU-GUUGUGGUCGa-5' miRNA<br>3'pairing Seed	ACTAGC<br>Imperfect		☐	M
Imperfect match<br>15491 15513<br>5'-accaCTGAGTCATCTCGCCAGCc -3' UTR<br>3'-gccgGACUAAGUGUUGUGGUCGa-5' miRNA<br>3'pairing Seed	GCCAGC<br>Imperfect		☐	M
Imperfect match<br>23669 23694<br>5'-ggGCTTGGATCCACTAGTCACTAGCt -3' UTR<br>3'-gcCGGA-CUAAGUGUU--GUGGUCGa-5' miRNA<br>3'pairing Seed	ACTAGC<br>Imperfect		☐	M
8mer<br>28472 28494<br>5'-tattgTTACTATTAACACCAGCa-3' UTR<br>3'-gccggACUAAGUGUUGUGGUCGa-5' miRNA<br>3'pairing Seed	CACCAGCA<br>8mer		☐	M T
Imperfect match<br>32818 32839<br>5'-tgGCCTG--TCACAGACACCACCa -3' UTR<br>3'-gcCGGACUAAGUGU-UGUGGUCGa-5' miRNA<br>3'pairing Seed	ACCACC<br>Imperfect		☐	M
Imperfect match<br>33676 33701<br>5'-tccCTTTATTTGCCCAATCATCAGCt -3' UTR<br>3'-gccGGACUAA--GUGUU-GUGGUCGa-5' miRNA<br>3'pairing Seed	ATCAGC<br>Imperfect		☐	M
8mer<br>34522 34545<br>5'-tgGCAGAATGTACTACCACCAGCa-3' UTR<br>3'-gcCGGACUAAGUG-UUGUGGUCGa-5' miRNA<br>3'pairing Seed	CACCAGCA<br>8mer		☐	M T
8mer<br>35133 35154<br>5'-ttggtccATTTA-AACACCAGCa-3' UTR<br>3'-gccggacUAAGUGUUGUGGUCGa-5' miRNA<br>3'pairing Seed	CACCAGCA<br>8mer		☐	M T
7mer-m8<br>39859 39883<br>5'-taaCTTAAGTGATAACACACCAGCt-3' UTR<br>3'-gccGGACUAAGUGU--UGUGGUCGa-5' miRNA<br>3'pairing Seed	CACCAGC<br>7mer-m8		☐	M T
Imperfect match<br>40805 40828<br>5'-gctCATTATTCAGTCACACTAGCa -3' UTR<br>3'-gccGGACUAAGU-GUUGUGGUCGa-5' miRNA<br>3'pairing Seed	ACTAGC<br>Imperfect		☐	M
Imperfect match<br>46858 46880<br>5'-accaCTGAGTCATCTCGCCAGCc -3' UTR<br>3'-gccgGACUAAGUGUUGUGGUCGa-5' miRNA<br>3'pairing Seed	GCCAGC<br>Imperfect		☐	M
Imperfect match<br>55044 55069<br>5'-ggGCTTGGATCCACTAGTCACTAGCt -3' UTR<br>3'-gcCGGA-CUAAGUGUU--GUGGUCGa-5' miRNA<br>3'pairing Seed	ACTAGC<br>Imperfect		☐	M
8mer<br>59848 59870<br>5'-tattgTTACTATTAACACCAGCa-3' UTR<br>3'-gccggACUAAGUGUUGUGGUCGa-5' miRNA<br>3'pairing Seed	CACCAGCA<br>8mer		☐	M T

<table>
<tr><th>2D Structure</th><th>Local AU</th><th>Position</th><th>Conservation</th><th>Predicted By</th></tr>
<tr><td>Imperfect match
9043 9064
5'-ctcaaTGAACTAC-ACACCGGCt -3' UTR
||| :|| |||||:||
3'-gccggACUAAGUGUUGUGGUCGa-5' miRNA
16 15 14 13 7 6 5 4 3 2
3'pairing Seed</td><td>ACCGGC
Imperfect</td><td></td><td></td><td>M</td></tr>
<tr><td>Imperfect match
10775 10795
5'-gttCCTG--TCTCGCCACCGGCt -3' UTR
|||| || |: ||||:||
3'-gccGGACUAAGUGUUGUGGUCGa-5' miRNA
16 15 14 13 7 6 5 4 3 2
3'pairing Seed</td><td>ACCGGC
Imperfect</td><td></td><td></td><td>M</td></tr>
<tr><td>Imperfect match
12234 12255
5'-tgGCCTGACCTGC-TCACTAGCc -3' UTR
|||||| ::| |||:|||
3'-gcCGGACUAAGUGUUGUGGUCGa-5' miRNA
16 15 14 13 7 6 5 4 3 2
3'pairing Seed</td><td>ACTAGC
Imperfect</td><td></td><td></td><td>M</td></tr>
<tr><td>Imperfect match
23262 23284
5'-acttaaaATTTGTGACATCAGCa -3' UTR
|||::::|||:||||
3'-gccggacUAAGUGUUGUGGUCGa-5' miRNA
16 15 14 13 7 6 5 4 3 2
3'pairing Seed</td><td>ATCAGC
Imperfect</td><td></td><td></td><td>M</td></tr>
<tr><td>Imperfect match
36259 36280
5'-ctcaCTGAACTAC-ACACCGGCt -3' UTR
|||| :|| |||||:||
3'-gccgGACUAAGUGUUGUGGUCGa-5' miRNA
16 15 14 13 7 6 5 4 3 2
3'pairing Seed</td><td>ACCGGC
Imperfect</td><td></td><td></td><td>M</td></tr>
<tr><td>Imperfect match
39321 39342
5'-cgGCCTGACCTGC-TCACTAGCc -3' UTR
|||||| ::| |||:|||
3'-gcCGGACUAAGUGUUGUGGUCGa-5' miRNA
16 15 14 13 7 6 5 4 3 2
3'pairing Seed</td><td>ACTAGC
Imperfect</td><td></td><td></td><td>M</td></tr>
<tr><td>7mer-m8
39457 39482
5'-acacagaATTCGGAAGCTCACCAGCg-3' UTR
||||: || |||||||
3'-gccggacUAAGUGUU---GUGGUCGa-5' miRNA
16 15 14 13 7 6 5 4 3 2
3'pairing Seed</td><td>CACCAGC
7mer-m8</td><td></td><td></td><td>M T</td></tr>
<tr><td>7mer-m8
45503 45523
5'-acagCTAAGTCTT--CACCAGCc-3' UTR
|| | || : |||||||
3'-gccgGACUAAGUGUUGUGGUCGa-5' miRNA
16 15 14 13 7 6 5 4 3 2
3'pairing Seed</td><td>CACCAGC
7mer-m8</td><td></td><td></td><td>M T</td></tr>
<tr><td>7mer-m8
52973 52992
5'-aaGCCACAT---CATCACCAGCc-3' UTR
||| || || |||||||
3'-gcCGGACUAAGUGUUGUGGUCGa-5' miRNA
16 15 14 13 7 6 5 4 3 2
3'pairing Seed</td><td>CACCAGC
7mer-m8</td><td></td><td></td><td>M T</td></tr>
<tr><td>7mer-A1
63998 64020
5'-aatTCTCTATCATCATACCAGCa-3' UTR
:|| |||: |:||||||
3'-gccGGACUAAGUGUUGUGGUCGa-5' miRNA
16 15 14 13 7 6 5 4 3 2
3'pairing Seed</td><td>TACCAGCA
7mer-A1</td><td></td><td></td><td>M T</td></tr>
<tr><td>8mer
67678 67699
5'-ggcCCTCCATC-CATCACCAGCa-3' UTR
||| || || |||||||
3'-gccGGACUAAGUGUUGUGGUCGa-5' miRNA
16 15 14 13 7 6 5 4 3 2
3'pairing Seed</td><td>CACCAGCA
8mer</td><td></td><td></td><td>M T</td></tr>
</table>

2D Structure	Local AU	Position	Conservation	Predicted By
Imperfect match<br>19436 19458<br>5'-gtGCCTGGCAAGACACACTAGCc -3' UTR<br>3'-gcCGGACUAAGUGUUGUGGUCGa-5' miRNA<br>16 15 14 13 7 6 5 4 3 2<br>3'pairing Seed	ACTAGC<br>Imperfect			M
7mer-A1<br>24104 24127<br>5'-tcagagGATTCAGAATTACCAGCa-3' UTR<br>3'-gccggaCUAAGUGUU-GUGGUCGa-5' miRNA<br>16 15 14 13 7 6 5 4 3 2<br>3'pairing Seed	TACCAGCA<br>7mer-A1			M T
Offset 6mer<br>26928 26949<br>5'-tgGCTTGATT-ATTGCACCAGAa-3' UTR<br>3'-gcCGGACUAAGUGUUGUGGUCga-5' miRNA<br>16 15 14 13 7 6 5 4 3 2<br>3'pairing Seed	CACCAG<br>Offset 6mer			M
Offset 6mer<br>27995 28016<br>5'-attgCTGATTCA-GCCACCAGAg-3' UTR<br>3'-gccgGACUAAGUGUUGUGGUCga-5' miRNA<br>16 15 14 13 7 6 5 4 3 2<br>3'pairing Seed	CACCAG<br>Offset 6mer			M
7mer-m8<br>28623 28649<br>5'-ttggaTGATGCTGGAAGATCACCAGCt-3' UTR<br>3'-gccggACUAAG----UGUUGUGGUCGa-5' miRNA<br>16 15 14 13 7 6 5 4 3 2<br>3'pairing Seed	CACCAGC<br>7mer-m8			M T
Imperfect match<br>49886 49907<br>5'-accataGATGC-CAGCATCAGCa -3' UTR<br>3'-gccggaCUAAGUGUUGUGGUCGa-5' miRNA<br>16 15 14 13 7 6 5 4 3 2<br>3'pairing Seed	ATCAGC<br>Imperfect			M
7mer-m8<br>58577 58600<br>5'-atcaTTAATGTATTCACACCAGCc-3' UTR<br>3'-gccgGACUAAGU-GUUGUGGUCGa-5' miRNA<br>16 15 14 13 7 6 5 4 3 2<br>3'pairing Seed	CACCAGC<br>7mer-m8			M T
Imperfect match<br>60728 60751<br>5'-attCCTGGATTTGCCACACTGGCa -3' UTR<br>3'-gccGGA-CUAAGUGUUGUGGUCGa-5' miRNA<br>16 15 14 13 7 6 5 4 3 2<br>3'pairing Seed	ACTGGC<br>Imperfect			M
Imperfect match<br>60789 60813<br>5'-ttaCCTGGACCAGAGAACATCAGCt -3' UTR<br>3'-gccGGACUAAG--UGUUGUGGUCGa-5' miRNA<br>16 15 14 13 7 6 5 4 3 2<br>3'pairing Seed	ATCAGC<br>Imperfect			M
Imperfect match<br>75958 75980<br>5'-ccaCCTGAGTTATTGCTCCAGCc -3' UTR<br>3'-gccGGACUAAGUGUUGUGGUCGa-5' miRNA<br>16 15 14 13 7 6 5 4 3 2<br>3'pairing Seed	TCCAGC<br>Imperfect			M

2D Structure	Local AU	Position	Conservation	Predicted By
Imperfect match<br>3545 3571<br>5'-tttTTTAATTCATATGAGACACTAGCt-3' UTR<br>3'-gccGGACUAAGUGU----UGUGGUCGa-5' miRNA<br>16 15 14 13 7 6 5 4 3 2<br>3'pairing Seed	ACTAGC<br>Imperfect			M
Offset 6mer<br>11280 11301<br>5'-gaGGCAGATTCG-AACACCAGat-3' UTR<br>3'-gcCGGACUAAGUGUUGUGGUCga-5' miRNA<br>16 15 14 13 7 6 5 4 3 2<br>3'pairing Seed	CACCAG<br>Offset 6mer			M
Imperfect match<br>11856 11877<br>5'-accaTTGA-TGGTAGCATCAGCa-3' UTR<br>3'-gccgGACUAAGUGUUGUGGUCGa-5' miRNA<br>16 15 14 13 7 6 5 4 3 2<br>3'pairing Seed	ATCAGC<br>Imperfect			M
7mer-m8<br>28734 28759<br>5'-ttaCCTGATGAGCCATCTCACCAGCc-3' UTR<br>3'-gccGGACUA---AGUGUUGUGGUCGa-5' miRNA<br>16 15 14 13 7 6 5 4 3 2<br>3'pairing Seed	CACCAGC<br>7mer-m8			M T
Imperfect match<br>33768 33791<br>5'-attCTTGATCTTACAGCACAAGCc-3' UTR<br>3'-gccGGACUA-AGUGUUGUGGUCGa-5' miRNA<br>16 15 14 13 7 6 5 4 3 2<br>3'pairing Seed	ACAAGC<br>Imperfect			M
Imperfect match<br>38996 39021<br>5'-gaGATTGGTTCACAAGAGCAGCAGCa-3' UTR<br>3'-gcCGGACUAAGUGUU---GUGGUCGa-5' miRNA<br>16 15 14 13 7 6 5 4 3 2<br>3'pairing Seed	AGCAGC<br>Imperfect			M
7mer-m8<br>53754 53776<br>5'-cccaCTGAGCCATCTCACCAGCc-3' UTR<br>3'-gccgGACUAAGUGUUGUGGUCGa-5' miRNA<br>16 15 14 13 7 6 5 4 3 2<br>3'pairing Seed	CACCAGC<br>7mer-m8			M T
7mer-m8<br>57829 57852<br>5'-aaatagaACTCAGGACCACCAGCc-3' UTR<br>3'-gccggacUAAGUGUU-GUGGUCGa-5' miRNA<br>16 15 14 13 7 6 5 4 3 2<br>3'pairing Seed	CACCAGC<br>7mer-m8			M T
7mer-m8<br>64477 64500<br>5'-gtGACTCAAATACCAACACCAGCt-3' UTR<br>3'-gcCGGACUAAGU-GUUGUGGUCGa-5' miRNA<br>16 15 14 13 7 6 5 4 3 2<br>3'pairing Seed	CACCAGC<br>7mer-m8			M T
Imperfect match<br>64556 64578<br>5'-taaCTGGGTTTGCAACACTGGCc-3' UTR<br>3'-gccGGACUAAGUGUUGUGGUCGa-5' miRNA<br>16 15 14 13 7 6 5 4 3 2<br>3'pairing Seed	ACTGGC<br>Imperfect			M
Imperfect match<br>64661 64682<br>5'-ggcCCTG-CTGACTGCGCCAGCc-3' UTR<br>3'-gccGGACUAAGUGUUGUGGUCGa-5' miRNA<br>16 15 14 13 7 6 5 4 3 2<br>3'pairing Seed	GCCAGC<br>Imperfect			M
7mer-m8<br>64934 64954<br>5'-tcaaagcATTATC--CACCAGCc-3' UTR<br>3'-gccggacUAAGUGUUGUGGUCGa-5' miRNA<br>16 15 14 13 7 6 5 4 3 2<br>3'pairing Seed	CACCAGC<br>7mer-m8			M T
7mer-m8<br>68091 68114<br>5'-tgtTCTAGTACATCTTCACCAGCt-3' UTR<br>3'-gccGGACUAAGU-GUUGUGGUCGa-5' miRNA<br>16 15 14 13 7 6 5 4 3 2<br>3'pairing Seed	CACCAGC<br>7mer-m8			M T

2D Structure	Local AU	Position	Conservation	Predicted By

```
13492                 Imperfect match  13516
5'-aatCCTCGATCTTACAGCACAAGCc -3'  UTR
      ||| ||| |:|||:||| |||
3'-gccGGA-CUA-AGUGUUGUGGUCGa-5'  miRNA
          16 15 14 13   7 6 5 4 3 2
        3'pairing          Seed
```
Local AU: ACAAGC — Imperfect
Predicted By: M

```
22512                  7mer-m8  22537
5'-tgtaccaACTTGCAATCCCACCAGCc-3'  UTR
          | |::|||   |||||||
3'-gccggacUAAGUGUU---GUGGUCGa-5'  miRNA
          16 15 14 13   7 6 5 4 3 2
        3'pairing          Seed
```
Local AU: CACCAGC — 7mer-m8
Predicted By: M T

```
22942                Imperfect match  22965
5'-atcCTTGATCTTACAACACAAGCt -3'  UTR
      |:|||| |:||||||| |||
3'-gccGGACUA-AGUGUUGUGGUCGa-5'  miRNA
          16 15 14 13  7 6 5 4 3 2
        3'pairing         Seed
```
Local AU: ACAAGC — Imperfect
Predicted By: M

```
28764               Imperfect match  28786
5'-tctTCTGATTATCACCACCACCa -3'  UTR
      :||||||  || ||||| |
3'-gccGGACUAAGUGUUGUGGUCGa-5'  miRNA
          16 15 14 13  7 6 5 4 3 2
        3'pairing        Seed
```
Local AU: ACCACC — Imperfect
Predicted By: M

```
29394               Imperfect match  29415
5'-aaGCCAG-TTTGAATCATCAGCt -3'  UTR
     ||| | ||:: | ||:||||
3'-gcCGGACUAAGUGUUGUGGUCGa-5'  miRNA
          16 15 14 13  7 6 5 4 3 2
        3'pairing        Seed
```
Local AU: ATCAGC — Imperfect
Predicted By: M

```
36814                  7mer-m8  36837
5'-actTCTGGTAAGGACCCACCAGCt-3'  UTR
      :|||:|  : |  |||||||
3'-gccGGACUAAGUGU-UGUGGUCGa-5'  miRNA
          16 15 14 13  7 6 5 4 3 2
        3'pairing         Seed
```
Local AU: CACCAGC — 7mer-m8
Predicted By: M T

```
38926               Imperfect match  38948
5'-gcaagTGCTTCCTGGCATCAGCa -3'  UTR
        || ||| :::||:||||
3'-gccggACUAAGUGUUGUGGUCGa-5'  miRNA
          16 15 14 13  7 6 5 4 3 2
        3'pairing        Seed
```
Local AU: ATCAGC — Imperfect
Predicted By: M

```
43422               Imperfect match  43443
5'-ggGCCTAAGGT-CAGCATCAGCc -3'  UTR
     |||| |  : ||:||:||||
3'-gcCGGACUAAGUGUUGUGGUCGa-5'  miRNA
          16 15 14 13  7 6 5 4 3 2
        3'pairing        Seed
```
Local AU: ATCAGC — Imperfect
Predicted By: M

```
48093                   8mer  48116
5'-tacaagttTTCACTCCCACCAGCa-3'  UTR
           |||||   |||||||
3'-gccggacuAAGUG-UUGUGGUCGa-5'  miRNA
          16 15 14 13  7 6 5 4 3 2
        3'pairing         Seed
```
Local AU: CACCAGCA — 8mer
Predicted By: M T

```
50201                  8mer  50223
5'-agggtacacgCCTGTCACCAGCa-3'  UTR
             | ::  |||||||
3'-gccggacuaaGUGUUGUGGUCGa-5'  miRNA
          16 15 14 13  7 6 5 4 3 2
        3'pairing        Seed
```
Local AU: CACCAGCA — 8mer
Predicted By: M T

```
59937               Imperfect match  59959
5'-gtaagaGGTTTGCCCCATCAGCt -3'  UTR
         |:||::|  ||:||||
3'-gccggaCUAAGUGUUGUGGUCGa-5'  miRNA
          16 15 14 13  7 6 5 4 3 2
        3'pairing        Seed
```
Local AU: ATCAGC — Imperfect
Predicted By: M

```
60584                         8mer  60612
5'-tgGCCATAATCACCATGGTCTCACCAGCa-3'  UTR
     |||  | |||| |      |||||||
3'-gcCGGACUAAGUGUU------GUGGUCGa-5'  miRNA
          16 15 14 13        7 6 5 4 3 2
        3'pairing               Seed
```
Local AU: CACCAGCA — 8mer
Predicted By: M T

```
64985                  Imperfect match  65010
5'-actTCTGTATTTGTCAGGCACTAGCa -3'  UTR
      :||| |||:: || :|||:|||
3'-gccGGAC-UAAGU-GU-UGUGGUCGa-5'  miRNA
           16 15 14 13  7 6 5 4 3 2
        3'pairing           Seed
```
Local AU: ACTAGC — Imperfect
Predicted By: M

```
67719                Imperfect match  67742
5'-aaGTTTGATTTTTGTCCACTAGCt -3'  UTR
     |::|||||: ::  |||:|||
3'-gcCGGACUAAGUGU-UGUGGUCGa-5'  miRNA
          16 15 14 13  7 6 5 4 3 2
        3'pairing         Seed
```
Local AU: ACTAGC — Imperfect
Predicted By: M

```
78297                Imperfect match  78320
5'-ggGTGTGATTCCCAAGCACCATCa -3'  UTR
     |: |||||| ||| ||||| |
3'-gcCGGACUAAGUGUU-GUGGUCGa-5'  miRNA
          16 15 14 13  7 6 5 4 3 2
        3'pairing         Seed
```
Local AU: ACCATC — Imperfect
Predicted By: M

<table>
<tr><th>2D Structure</th><th>Local AU</th><th>Position</th><th>Conservation</th><th>Predicted By</th></tr>
<tr><td>Imperfect match
15705 15727
5'-gtGCCTGGCAAGACACACTAGCc -3' UTR
     |||||:   : ||||:|||
3'-gcCGGACUAAGUGUUGUGGUCGa-5' miRNA
16 15 14 13 7 6 5 4 3 2
3'pairing Seed</td><td>ACTAGC
Imperfect</td><td></td><td></td><td>M</td></tr>
<tr><td>7mer-A1
20373 20396
5'-tcagagGATTCAGAATTACCAGCa-3' UTR
         |||||| || :||||||
3'-gccggaCUAAGUGUU-GUGGUCGa-5' miRNA
16 15 14 13 7 6 5 4 3 2
3'pairing Seed</td><td>TACCAGCA
7mer-A1</td><td></td><td></td><td>M T</td></tr>
<tr><td>Offset 6mer
23197 23218
5'-tgGCTTGATT-ATTGCACCAGaa-3' UTR
     ||:||||| |: :||||||
3'-gcCGGACUAAGUGUUGUGGUCga-5' miRNA
16 15 14 13 7 6 5 4 3 2
3'pairing Seed</td><td>CACCAG
Offset 6mer</td><td></td><td></td><td>M</td></tr>
<tr><td>Offset 6mer
24264 24285
5'-attgCTGATTCA-GCCACCAGag-3' UTR
       |||||||| : ||||||
3'-gccgGACUAAGUGUUGUGGUCga-5' miRNA
16 15 14 13 7 6 5 4 3 2
3'pairing Seed</td><td>CACCAG
Offset 6mer</td><td></td><td></td><td>M</td></tr>
<tr><td>7mer-m8
24892 24918
5'-ttggaTGATGCTGGAAGATCACCAGCt-3' UTR
        |||| | | | |||||||
3'-gccggACUAAG----UGUUGUGGUCGa-5' miRNA
16 15 14 13 7 6 5 4 3 2
3'pairing Seed</td><td>CACCAGC
7mer-m8</td><td></td><td></td><td>M T</td></tr>
<tr><td>Imperfect match
46155 46176
5'-accataGATGC-CAGCATCAGCa -3' UTR
         ||| | ||:||:||||
3'-gccggaCUAAGUGUUGUGGUCGa-5' miRNA
16 15 14 13 7 6 5 4 3 2
3'pairing Seed</td><td>ATCAGC
Imperfect</td><td></td><td></td><td>M</td></tr>
<tr><td>7mer-m8
54846 54869
5'-atcaTTAATGTATTCACACCAGCc-3' UTR
       :| || :| : ||||||||
3'-gccgGACUAAGU-GUUGUGGUCGa-5' miRNA
16 15 14 13 7 6 5 4 3 2
3'pairing Seed</td><td>CACCAGC
7mer-m8</td><td></td><td></td><td>M T</td></tr>
<tr><td>Imperfect match
56997 57020
5'-attCCTGGATTTGCCACACTGGCa -3' UTR
      ||| ||||::| ||||::||
3'-gccGGA-CUAAGUGUUGUGGUCGa-5' miRNA
16 15 14 13 7 6 5 4 3 2
3'pairing Seed</td><td>ACTGGC
Imperfect</td><td></td><td></td><td>M</td></tr>
<tr><td>Imperfect match
57058 57082
5'-ttaCCTGGACCAGAGAACATCAGCt -3' UTR
      ||||: | | ||||:||||
3'-gccGGACUAAG--UGUUGUGGUCGa-5' miRNA
16 15 14 13 7 6 5 4 3 2
3'pairing Seed</td><td>ATCAGC
Imperfect</td><td></td><td></td><td>M</td></tr>
<tr><td>Imperfect match
72227 72249
5'-ccaCCTGAGTTATTGCTCCAGCc -3' UTR
      ||||| |:|: :| |||||
3'-gccGGACUAAGUGUUGUGGUCGa-5' miRNA
16 15 14 13 7 6 5 4 3 2
3'pairing Seed</td><td>TCCAGC
Imperfect</td><td></td><td></td><td>M</td></tr>
<tr><td>Offset 6mer
80533 80554
5'-catCCTGGCTCAC-CCACCAGgg-3' UTR
      ||||: |||| ||||||
3'-gccGGACUAAGUGUUGUGGUCga-5' miRNA
16 15 14 13 7 6 5 4 3 2
3'pairing Seed</td><td>CACCAG
Offset 6mer</td><td></td><td></td><td>M</td></tr>
</table>

<table>
<tr><th>2D Structure</th><th>Local AU</th><th>Position</th><th>Conservation</th><th>Predicted By</th></tr>
<tr><td>Imperfect match
12357 12379
5'-gtGCCTGGCAAGACACACTAGCc -3' UTR
|||||: : ||||:|||
3'-gcCGGACUAAGUGUUGUGGUCGa-5' miRNA
16 15 14 13 7 6 5 4 3 2
3'pairing Seed</td><td>ACTAGC
Imperfect</td><td></td><td></td><td>M</td></tr>
<tr><td>7mer-A1
17025 17048
5'-tcagagGATTCAGAATTACCAGCa-3' UTR
|||||| || :||||||
3'-gccggaCUAAGUGUU-GUGGUCGa-5' miRNA
16 15 14 13 7 6 5 4 3 2
3'pairing Seed</td><td>TACCAGCA
7mer-A1</td><td></td><td></td><td>M T</td></tr>
<tr><td>Offset 6mer
19849 19870
5'-tgGCTTGATT-ATTGCACCAGaa-3' UTR
||:||||| |: :||||||
3'-gcCGGACUAAGUGUUGUGGUCga-5' miRNA
16 15 14 13 7 6 5 4 3 2
3'pairing Seed</td><td>CACCAG
Offset 6mer</td><td></td><td></td><td>M</td></tr>
<tr><td>Offset 6mer
20916 20937
5'-attgCTGATTCA-GCCACCAGag-3' UTR
|||||||| : ||||||
3'-gccgGACUAAGUGUUGUGGUCga-5' miRNA
16 15 14 13 7 6 5 4 3 2
3'pairing Seed</td><td>CACCAG
Offset 6mer</td><td></td><td></td><td>M</td></tr>
<tr><td>7mer-m8
21544 21570
5'-ttggaTGATGCTGGAAGATCACCAGCt-3' UTR
|||| | | | |||||||
3'-gccggACUAAG----UGUUGUGGUCGa-5' miRNA
16 15 14 13 7 6 5 4 3 2
3'pairing Seed</td><td>CACCAGC
7mer-m8</td><td></td><td></td><td>M T</td></tr>
<tr><td>Imperfect match
42807 42828
5'-accataGATGC-CAGCATCAGCa -3' UTR
||| | ||:||:||||
3'-gccggaCUAAGUGUUGUGGUCGa-5' miRNA
16 15 14 13 7 6 5 4 3 2
3'pairing Seed</td><td>ATCAGC
Imperfect</td><td></td><td></td><td>M</td></tr>
<tr><td>7mer-m8
51498 51521
5'-atcaTTAATGTATTCACACCAGCc-3' UTR
:| || :| : ||||||||
3'-gccgGACUAAGU-GUUGUGGUCGa-5' miRNA
16 15 14 13 7 6 5 4 3 2
3'pairing Seed</td><td>CACCAGC
7mer-m8</td><td></td><td></td><td>M T</td></tr>
<tr><td>Imperfect match
53649 53672
5'-attCCTGGATTTGCCACACTGGCa -3' UTR
||| ||||::| ||||::||
3'-gccGGA-CUAAGUGUUGUGGUCGa-5' miRNA
16 15 14 13 7 6 5 4 3 2
3'pairing Seed</td><td>ACTGGC
Imperfect</td><td></td><td></td><td>M</td></tr>
<tr><td>Imperfect match
53710 53734
5'-ttaCCTGGACCAGAGAACATCAGCt -3' UTR
||||: | | ||||:||||
3'-gccGGACUAAG--UGUUGUGGUCGa-5' miRNA
16 15 14 13 7 6 5 4 3 2
3'pairing Seed</td><td>ATCAGC
Imperfect</td><td></td><td></td><td>M</td></tr>
<tr><td>Imperfect match
68879 68901
5'-ccaCCTGAGTTATTGCTCCAGCc -3' UTR
||||| |:|: :| |||||
3'-gccGGACUAAGUGUUGUGGUCGa-5' miRNA
16 15 14 13 7 6 5 4 3 2
3'pairing Seed</td><td>TCCAGC
Imperfect</td><td></td><td></td><td>M</td></tr>
</table>

2D Structure	Local AU	Position	Conservation	Predicted By
Imperfect match<br>6835 6861<br>5'-tttTTTAATTCATATGAGACACTAGCt-3' UTR<br>3'-gccGGACUAAGUGU----UGUGGUCGa-5' miRNA<br>16 15 14 13 7 6 5 4 3 2<br>3'pairing Seed	ACTAGC<br>Imperfect			M
Offset 6mer<br>14570 14591<br>5'-gaGGCAGATTCG-AACACCAGat-3' UTR<br>3'-gcCGGACUAAGUGUUGUGGUCga-5' miRNA<br>16 15 14 13 7 6 5 4 3 2<br>3'pairing Seed	CACCAG<br>Offset 6mer			M
Imperfect match<br>15146 15167<br>5'-accaTTGA-TGGTAGCATCAGCa-3' UTR<br>3'-gccgGACUAAGUGUUGUGGUCGa-5' miRNA<br>16 15 14 13 7 6 5 4 3 2<br>3'pairing Seed	ATCAGC<br>Imperfect			M
7mer-m8<br>32024 32049<br>5'-ttaCCTGATGAGCCATCTCACCAGCc-3' UTR<br>3'-gccGGACUA---AGUGUUGUGGUCGa-5' miRNA<br>16 15 14 13 7 6 5 4 3 2<br>3'pairing Seed	CACCAGC<br>7mer-m8			M T
Imperfect match<br>37058 37081<br>5'-attCTTGATCTTACAGCACAAGCc-3' UTR<br>3'-gccGGACUA-AGUGUUGUGGUCGa-5' miRNA<br>16 15 14 13 7 6 5 4 3 2<br>3'pairing Seed	ACAAGC<br>Imperfect			M
Imperfect match<br>42286 42311<br>5'-gaGATTGGTTCACAAGAGCAGCAGCa-3' UTR<br>3'-gcCGGACUAAGUGUU---GUGGUCGa-5' miRNA<br>16 15 14 13 7 6 5 4 3 2<br>3'pairing Seed	AGCAGC<br>Imperfect			M
7mer-m8<br>57044 57066<br>5'-cccaCTGAGCCATCTCACCAGCc-3' UTR<br>3'-gccgGACUAAGUGUUGUGGUCGa-5' miRNA<br>16 15 14 13 7 6 5 4 3 2<br>3'pairing Seed	CACCAGC<br>7mer-m8			M T
7mer-m8<br>61119 61142<br>5'-aaatagaACTCAGGACCACCAGCc-3' UTR<br>3'-gccggacUAAGUGUU-GUGGUCGa-5' miRNA<br>16 15 14 13 7 6 5 4 3 2<br>3'pairing Seed	CACCAGC<br>7mer-m8			M T
7mer-m8<br>67767 67790<br>5'-gtGACTCAAATACCAACACCAGCt-3' UTR<br>3'-gcCGGACUAAGU-GUUGUGGUCGa-5' miRNA<br>16 15 14 13 7 6 5 4 3 2<br>3'pairing Seed	CACCAGC<br>7mer-m8			M T
Imperfect match<br>67846 67868<br>5'-taaCTGGGTTTGCAACACTGGCc-3' UTR<br>3'-gccGGACUAAGUGUUGUGGUCGa-5' miRNA<br>16 15 14 13 7 6 5 4 3 2<br>3'pairing Seed	ACTGGC<br>Imperfect			M
Imperfect match<br>67951 67972<br>5'-ggcCCTG-CTGACTGCGCCAGCc-3' UTR<br>3'-gccGGACUAAGUGUUGUGGUCGa-5' miRNA<br>16 15 14 13 7 6 5 4 3 2<br>3'pairing Seed	GCCAGC<br>Imperfect			M
7mer-m8<br>68224 68244<br>5'-tcaaagcATTATC--CACCAGCc-3' UTR<br>3'-gccggacUAAGUGUUGUGGUCGa-5' miRNA<br>16 15 14 13 7 6 5 4 3 2<br>3'pairing Seed	CACCAGC<br>7mer-m8			M T
7mer-m8<br>71381 71404<br>5'-tgtTCTAGTACATCTTCACCAGCt-3' UTR<br>3'-gccGGACUAAGU-GUUGUGGUCGa-5' miRNA<br>16 15 14 13 7 6 5 4 3 2<br>3'pairing Seed	CACCAGC<br>7mer-m8			M T

2D Structure	Local AU	Position	Conservation	Predicted By
Imperfect match<br>7903 7925<br>5'-ctcCGTGATAGAGGCCATCAGCc -3' UTR<br>3'-gccGGACUAAGUGUUGUGGUCGa-5' miRNA<br>16 15 14 13 7 6 5 4 3 2<br>3'pairing Seed	ATCAGC<br>Imperfect			M
Imperfect match<br>8982 9004<br>5'-acGCCTGGGTCACTTCAACAGCt -3' UTR<br>3'-gcCGGACUAAGUGUUGUGGUCGa-5' miRNA<br>16 15 14 13 7 6 5 4 3 2<br>3'pairing Seed	AACAGC<br>Imperfect			M
7mer-m8<br>12831 12852<br>5'-tgGGTTGTCTTTC-ACACCAGCt-3' UTR<br>3'-gcCGGACUAAGUGUUGUGGUCGa-5' miRNA<br>16 15 14 13 7 6 5 4 3 2<br>3'pairing Seed	CACCAGC<br>7mer-m8			M T
7mer-m8<br>17826 17848<br>5'-gtagCTCCTTAAACACACCAGCg-3' UTR<br>3'-gccgGACUAAGUGUUGUGGUCGa-5' miRNA<br>16 15 14 13 7 6 5 4 3 2<br>3'pairing Seed	CACCAGC<br>7mer-m8			M T
7mer-m8<br>21234 21255<br>5'-ggcaagaATGTAC-ACACCAGCt-3' UTR<br>3'-gccggacUAAGUGUUGUGGUCGa-5' miRNA<br>16 15 14 13 7 6 5 4 3 2<br>3'pairing Seed	CACCAGC<br>7mer-m8			M T
Imperfect match<br>23456 23479<br>5'-ataggTTATTTGAAACCATCAGCa -3' UTR<br>3'-gccggACUAAGUGUU-GUGGUCGa-5' miRNA<br>16 15 14 13 7 6 5 4 3 2<br>3'pairing Seed	ATCAGC<br>Imperfect			M
8mer<br>31862 31887<br>5'-ttGTTTGTTTTACTTTCTCACCAGCa-3' UTR<br>3'-gcCGGACUAAGUG---UUGUGGUCGa-5' miRNA<br>16 15 14 13 7 6 5 4 3 2<br>3'pairing Seed	CACCAGCA<br>8mer			M T
6mer<br>32511 32536<br>5'-taGTTTGATTTTGCATTTTACCAGCt-3' UTR<br>3'-gcCGGACUAA---GUGUUGUGGUCGa-5' miRNA<br>16 15 14 13 7 6 5 4 3 2<br>3'pairing Seed	ACCAGC<br>6mer			M
Imperfect match<br>33365 33387<br>5'-tgggacGGTTACCATCACCGGCg -3' UTR<br>3'-gccggaCUAAGUGUUGUGGUCGa-5' miRNA<br>16 15 14 13 7 6 5 4 3 2<br>3'pairing Seed	ACCGGC<br>Imperfect			M
7mer-A1<br>39712 39734<br>5'-gtaggTGTCTCATCATACCAGCa-3' UTR<br>3'-gccggACUAAGUGUUGUGGUCGa-5' miRNA<br>16 15 14 13 7 6 5 4 3 2<br>3'pairing Seed	TACCAGCA<br>7mer-A1			M T
7mer-m8<br>40263 40284<br>5'-ttagTTCATT-ATCACACCAGCc-3' UTR<br>3'-gccgGACUAAGUGUUGUGGUCGa-5' miRNA<br>16 15 14 13 7 6 5 4 3 2<br>3'pairing Seed	CACCAGC<br>7mer-m8			M T
7mer-m8<br>45336 45359<br>5'-ttaCTTGGTCTCCAATCACCAGCc-3' UTR<br>3'-gccGGACUAAGUGUU-GUGGUCGa-5' miRNA<br>16 15 14 13 7 6 5 4 3 2<br>3'pairing Seed	CACCAGC<br>7mer-m8			M T
Imperfect match<br>51037 51058<br>5'-agGTCTGCTT-AGGGCACCGGCc -3' UTR<br>3'-gcCGGACUAAGUGUUGUGGUCGa-5' miRNA<br>16 15 14 13 7 6 5 4 3 2<br>3'pairing Seed	ACCGGC<br>Imperfect			M
8mer<br>56216 56239<br>5'-aatCCTGAGTGAACAACACCAGCa-3' UTR<br>3'-gccGGACU-AAGUGUUGUGGUCGa-5' miRNA<br>16 15 14 13 7 6 5 4 3 2<br>3'pairing Seed	CACCAGCA<br>8mer			M T
8mer<br>56381 56403<br>5'-acccacagcTAAGAGCACCAGCa-3' UTR<br>3'-gccggacuaAGUGUUGUGGUCGa-5' miRNA<br>16 15 14 13 7 6 5 4 3 2<br>3'pairing Seed	CACCAGCA<br>8mer			M T
Imperfect match<br>63104 63124<br>5'-gaGCATGGCTCAT--CATCAGCg -3' UTR<br>3'-gcCGGACUAAGUGUUGUGGUCGa-5' miRNA<br>16 15 14 13 7 6 5 4 3 2<br>3'pairing Seed	ATCAGC<br>Imperfect			M
Imperfect match<br>71306 71328<br>5'-taatcacAATCGCTACATCAGCt -3' UTR<br>3'-gccggacUAAGUGUUGUGGUCGa-5' miRNA<br>16 15 14 13 7 6 5 4 3 2<br>3'pairing Seed	ATCAGC<br>Imperfect			M
7mer-m8<br>73933 73954<br>5'-ccaCCGTCTTTAT-CCACCAGCc-3' UTR<br>3'-gccGGACUAAGUGUUGUGGUCGa-5' miRNA<br>16 15 14 13 7 6 5 4 3 2<br>3'pairing Seed	CACCAGC<br>7mer-m8			M T
8mer<br>76771 76798<br>5'-atcTCTCAGTCACTAGAAGTCACCAGCa-3' UTR<br>3'-gccGGACUAAGUG---UU--GUGGUCGa-5' miRNA<br>16 15 14 13 7 6 5 4 3 2<br>3'pairing Seed	CACCAGCA<br>8mer			M T
7mer-m8<br>80620 80641<br>5'-cacaCT-AGTCACCACACCAGCc-3' UTR<br>3'-gccgGACUAAGUGUUGUGGUCGa-5' miRNA<br>16 15 14 13 7 6 5 4 3 2<br>3'pairing Seed	CACCAGC<br>7mer-m8			M T

2D Structure	Local AU	Position	Conservation	Predicted By
11691 8mer 11716<br>5'-gaaagcacTTCACAGATACACCAGCa-3' UTR<br>3'-gccggacuAAGUGU---UGUGGUCGa-5' miRNA<br>16 15 14 13 7 6 5 4 3 2<br>3' pairing Seed	CACCAGCA<br>8mer			M T
Imperfect match<br>16315 16337<br>5'-gacCCTGACTTCCATCCCCAGCa -3' UTR<br>3'-gccGGACUAAGUGUUGUGGUCGa-5' miRNA<br>16 15 14 13 7 6 5 4 3 2<br>3' pairing Seed	CCCAGC<br>Imperfect			M
21251 7mer-m8 21273<br>5'-ttcTCTAATCCACCTCACCAGCt-3' UTR<br>3'-gccGGACUAAGUGUUGUGGUCGa-5' miRNA<br>16 15 14 13 7 6 5 4 3 2<br>3' pairing Seed	CACCAGC<br>7mer-m8			M T
24647 7mer-m8 24669<br>5'-gcaagtccTTTGAAACACCAGCc-3' UTR<br>3'-gccggacuAAGUGUUGUGGUCGa-5' miRNA<br>16 15 14 13 7 6 5 4 3 2<br>3' pairing Seed	CACCAGC<br>7mer-m8			M T
31830 7mer-m8 31850<br>5'-agaCTTG--TAAAAGCACCAGCt-3' UTR<br>3'-gccGGACUAAGUGUUGUGGUCGa-5' miRNA<br>16 15 14 13 7 6 5 4 3 2<br>3' pairing Seed	CACCAGC<br>7mer-m8			M T
39921 8mer 39941<br>5'-acGACTG--TGAGGTCACCAGCa-3' UTR<br>3'-gcCGGACUAAGUGUUGUGGUCGa-5' miRNA<br>16 15 14 13 7 6 5 4 3 2<br>3' pairing Seed	CACCAGCA<br>8mer			M T
Imperfect match<br>42229 42254<br>5'-ttGCTGGGATTACAAACGCATCAGCt -3' UTR<br>3'-gcCGGACUAAGUGUU---GUGGUCGa-5' miRNA<br>16 15 14 13 7 6 5 4 3 2<br>3' pairing Seed	ATCAGC<br>Imperfect			M
Imperfect match<br>49606 49628<br>5'-cctagacAGTAACAGCACTAGCc -3' UTR<br>3'-gccggacUAAGUGUUGUGGUCGa-5' miRNA<br>16 15 14 13 7 6 5 4 3 2<br>3' pairing Seed	ACTAGC<br>Imperfect			M
Imperfect match<br>76699 76721<br>5'-ctcgCTGAGCCATCTCGCCAGCc -3' UTR<br>3'-gccgGACUAAGUGUUGUGGUCGa-5' miRNA<br>16 15 14 13 7 6 5 4 3 2<br>3' pairing Seed	GCCAGC<br>Imperfect			M
76934 7mer-m8 76956<br>5'-cccaCTGAGCCATCTCACCAGCc-3' UTR<br>3'-gccgGACUAAGUGUUGUGGUCGa-5' miRNA<br>16 15 14 13 7 6 5 4 3 2<br>3' pairing Seed	CACCAGC<br>7mer-m8			M T

2D Structure	Local AU	Position	Conservation	Predicted By
8mer<br>70 92<br>5'-gccctccaagaggccCACCAGCa-3' UTR<br>3'-gccggacuaaguguuGUGGUCGa-5' miRNA<br>16 15 14 13 7 6 5 4 3 2<br>3'pairing Seed	CACCAGCA<br>8mer			M T
7mer-m8<br>3532 3553<br>5'-tcagTTCATTCA-GACACCAGCt-3' UTR<br>3'-gccgGACUAAGUGUUGUGGUCGa-5' miRNA<br>16 15 14 13 7 6 5 4 3 2<br>3'pairing Seed	CACCAGC<br>7mer-m8			M T
Imperfect match<br>11641 11663<br>5'-ctcCGTGATAGAGGCCATCAGCc -3' UTR<br>3'-gccGGACUAAGUGUUGUGGUCGa-5' miRNA<br>16 15 14 13 7 6 5 4 3 2<br>3'pairing Seed	ATCAGC<br>Imperfect			M
Imperfect match<br>12720 12742<br>5'-acGCCTGGGTCACTTCAACAGCt -3' UTR<br>3'-gcCGGACUAAGUGUUGUGGUCGa-5' miRNA<br>16 15 14 13 7 6 5 4 3 2<br>3'pairing Seed	AACAGC<br>Imperfect			M
7mer-m8<br>16569 16590<br>5'-tgGGTTGTCTTTC-ACACCAGCt-3' UTR<br>3'-gcCGGACUAAGUGUUGUGGUCGa-5' miRNA<br>16 15 14 13 7 6 5 4 3 2<br>3'pairing Seed	CACCAGC<br>7mer-m8			M T
7mer-m8<br>21564 21586<br>5'-gtagCTCCTTAAACACACCAGCg-3' UTR<br>3'-gccgGACUAAGUGUUGUGGUCGa-5' miRNA<br>16 15 14 13 7 6 5 4 3 2<br>3'pairing Seed	CACCAGC<br>7mer-m8			M T
7mer-m8<br>24972 24993<br>5'-ggcaagaATGTAC-ACACCAGCt-3' UTR<br>3'-gccggacUAAGUGUUGUGGUCGa-5' miRNA<br>16 15 14 13 7 6 5 4 3 2<br>3'pairing Seed	CACCAGC<br>7mer-m8			M T
Imperfect match<br>27194 27217<br>5'-ataggTTATTTGAAACCATCAGCa -3' UTR<br>3'-gccggACUAAGUGUU-GUGGUCGa-5' miRNA<br>16 15 14 13 7 6 5 4 3 2<br>3'pairing Seed	ATCAGC<br>Imperfect			M
8mer<br>35600 35625<br>5'-ttGTTTGTTTTACTTTCTCACCAGCa-3' UTR<br>3'-gcCGGACUAAGUG---UUGUGGUCGa-5' miRNA<br>16 15 14 13 7 6 5 4 3 2<br>3'pairing Seed	CACCAGCA<br>8mer			M T
6mer<br>36249 36274<br>5'-taGTTTGATTTTGCATTTTACCAGCt-3' UTR<br>3'-gcCGGACUAA---GUGUUGUGGUCGa-5' miRNA<br>16 15 14 13 7 6 5 4 3 2<br>3'pairing Seed	ACCAGC<br>6mer			M
Imperfect match<br>37103 37125<br>5'-tgggacGGTTACCATCACCGGCg -3' UTR<br>3'-gccggaCUAAGUGUUGUGGUCGa-5' miRNA<br>16 15 14 13 7 6 5 4 3 2<br>3'pairing Seed	ACCGGC<br>Imperfect			M
7mer-A1<br>43450 43472<br>5'-gtaggTGTCTCATCATACCAGCa-3' UTR<br>3'-gccggACUAAGUGUUGUGGUCGa-5' miRNA<br>16 15 14 13 7 6 5 4 3 2<br>3'pairing Seed	TACCAGCA<br>7mer-A1			M T
7mer-m8<br>44001 44022<br>5'-ttagTTCATT-ATCACACCAGCc-3' UTR<br>3'-gccgGACUAAGUGUUGUGGUCGa-5' miRNA<br>16 15 14 13 7 6 5 4 3 2<br>3'pairing Seed	CACCAGC<br>7mer-m8			M T
7mer-m8<br>49074 49097<br>5'-ttaCTTGGTCTCCAATCACCAGCc-3' UTR<br>3'-gccGGACUAAGUGUU-GUGGUCGa-5' miRNA<br>16 15 14 13 7 6 5 4 3 2<br>3'pairing Seed	CACCAGC<br>7mer-m8			M T
Imperfect match<br>54775 54796<br>5'-agGTCTGCTT-AGGGCACCGGCc -3' UTR<br>3'-gcCGGACUAAGUGUUGUGGUCGa-5' miRNA<br>16 15 14 13 7 6 5 4 3 2<br>3'pairing Seed	ACCGGC<br>Imperfect			M
8mer<br>59954 59977<br>5'-aatCCTGAGTGAACAACACCAGCa-3' UTR<br>3'-gccGGACU-AAGUGUUGUGGUCGa-5' miRNA<br>16 15 14 13 7 6 5 4 3 2<br>3'pairing Seed	CACCAGCA<br>8mer			M T
8mer<br>60119 60141<br>5'-acccacagcTAAGAGCACCAGCa-3' UTR<br>3'-gccggacuaAGUGUUGUGGUCGa-5' miRNA<br>16 15 14 13 7 6 5 4 3 2<br>3'pairing Seed	CACCAGCA<br>8mer			M T
Imperfect match<br>66842 66862<br>5'-gaGCATGGCTCAT--CATCAGCg -3' UTR<br>3'-gcCGGACUAAGUGUUGUGGUCGa-5' miRNA<br>16 15 14 13 7 6 5 4 3 2<br>3'pairing Seed	ATCAGC<br>Imperfect			M
Imperfect match<br>75044 75066<br>5'-taatcacAATCGCTACATCAGCt -3' UTR<br>3'-gccggacUAAGUGUUGUGGUCGa-5' miRNA<br>16 15 14 13 7 6 5 4 3 2<br>3'pairing Seed	ATCAGC<br>Imperfect			M
7mer-m8<br>77671 77692<br>5'-ccaCCGTCTTTAT-CCACCAGCc-3' UTR<br>3'-gccGGACUAAGUGUUGUGGUCGa-5' miRNA<br>16 15 14 13 7 6 5 4 3 2<br>3'pairing Seed	CACCAGC<br>7mer-m8			M T
8mer<br>80509 80536<br>5'-atcTCTCAGTCACTAGAAGTCACCAGCa-3' UTR<br>3'-gccGGACUAAGUG---UU--GUGGUCGa-5' miRNA<br>16 15 14 13 7 6 5 4 3 2<br>3'pairing Seed	CACCAGCA<br>8mer			M T
7mer-m8<br>84358 84379<br>5'-cacaCT-AGTCACCACACCAGCc-3' UTR<br>3'-gccgGACUAAGUGUUGUGGUCGa-5' miRNA<br>16 15 14 13 7 6 5 4 3 2<br>3'pairing Seed	CACCAGC<br>7mer-m8			M T
Offset 6mer<br>94001 94023<br>5'-gtcgaTGATGTTCTACACCAGat-3' UTR<br>3'-gccggACUAAGUGUUGUGGUCga-5' miRNA<br>16 15 14 13 7 6 5 4 3 2<br>3'pairing Seed	CACCAG<br>Offset 6mer			M

2D Structure	Local AU	Position	Conservation	Predicted By
102 Imperfect match 124<br>5'-cctaCGGATTCATGGTACCAGTa -3' UTR<br>3'-gccgGACUAAGUGUUGUGGUCGa-5' miRNA<br>16 15 14 13 / 7 6 5 4 3 2<br>3'pairing Seed	ACCAGT<br>Imperfect		☐	M
7251 Imperfect match 7273<br>5'-tcaggTCATTAATTACATCAGCa -3' UTR<br>3'-gccggACUAAGUGUUGUGGUCGa-5' miRNA<br>16 15 14 13 / 7 6 5 4 3 2<br>3'pairing Seed	ATCAGC<br>Imperfect		☐	M
10835 7mer-A1 10859<br>5'-tgGCATTGTATTTTGAGTACCAGCa-3' UTR<br>3'-gcCG-GAC-UAAGUGUUGUGGUCGa-5' miRNA<br>16 15 14 13 / 7 6 5 4 3 2<br>3'pairing Seed	TACCAGCA<br>7mer-A1		☐	M T
12007 8mer 12027<br>5'-aaGCTTGCAATC-C--CACCAGCa-3' UTR<br>3'-gcCGGAC-UAAGUGUUGUGGUCGa-5' miRNA<br>16 15 14 13 / 7 6 5 4 3 2<br>3'pairing Seed	CACCAGCA<br>8mer		☐	M T
12429 Imperfect match 12452<br>5'-attCTTGATCTTATAGCACAAGCc -3' UTR<br>3'-gccGGACUA-AGUGUUGUGGUCGa-5' miRNA<br>16 15 14 13 / 7 6 5 4 3 2<br>3'pairing Seed	ACAAGC<br>Imperfect		☐	M
17475 8mer 17497<br>5'-ataCTAGTTTTATTTCACCAGCa-3' UTR<br>3'-gccGGACUAAGUGUUGUGGUCGa-5' miRNA<br>16 15 14 13 / 7 6 5 4 3 2<br>3'pairing Seed	CACCAGCA<br>8mer		☐	M T
22186 8mer 22210<br>5'-aaaagaaATATATAAGTCACCAGCa-3' UTR<br>3'-gccggacUAAGUGUU--GUGGUCGa-5' miRNA<br>16 15 14 13 / 7 6 5 4 3 2<br>3'pairing Seed	CACCAGCA<br>8mer		☐	M T
31299 8mer 31319<br>5'-aaGCTTGCAATC-C--CACCAGCa-3' UTR<br>3'-gcCGGAC-UAAGUGUUGUGGUCGa-5' miRNA<br>16 15 14 13 / 7 6 5 4 3 2<br>3'pairing Seed	CACCAGCA<br>8mer		☐	M T
43950 Imperfect match 43971<br>5'-atagCTCAATCAC-ACACTAGCc -3' UTR<br>3'-gccgGACUAAGUGUUGUGGUCGa-5' miRNA<br>16 15 14 13 / 7 6 5 4 3 2<br>3'pairing Seed	ACTAGC<br>Imperfect		☐	M
49378 Imperfect match 49400<br>5'-ggGGTTGGGTCACATCTCCAGCt -3' UTR<br>3'-gcCGGACUAAGUGUUGUGGUCGa-5' miRNA<br>16 15 14 13 / 7 6 5 4 3 2<br>3'pairing Seed	TCCAGC<br>Imperfect		☐	M
49956 7mer-m8 49979<br>5'-actTCT-ATTAAATTATCACCAGCt-3' UTR<br>3'-gccGGACUAAGU--GUUGUGGUCGa-5' miRNA<br>16 15 14 13 / 7 6 5 4 3 2<br>3'pairing Seed	CACCAGC<br>7mer-m8		☐	M T
66235 7mer-m8 66259<br>5'-ctgtagttTTGAAAATACACCAGCt-3' UTR<br>3'-gccggacuAAGUGU--UGUGGUCGa-5' miRNA<br>16 15 14 13 / 7 6 5 4 3 2<br>3'pairing Seed	CACCAGC<br>7mer-m8		☐	M T
70592 Imperfect match 70614<br>5'-ctaCCTGTTTTGTAGCCCCAGCc -3' UTR<br>3'-gccGGACUAAGUGUUGUGGUCGa-5' miRNA<br>16 15 14 13 / 7 6 5 4 3 2<br>3'pairing Seed	CCCAGC<br>Imperfect		☐	M
71033 8mer 71057<br>5'-tgtTCTGAGCTACTGTGCACCAGCa-3' UTR<br>3'-gccGGACUAAGUG--UUGUGGUCGa-5' miRNA<br>16 15 14 13 / 7 6 5 4 3 2<br>3'pairing Seed	CACCAGCA<br>8mer		☐	M T
75228 Imperfect match 75250<br>5'-tactggGACCCACATCACTAGCt -3' UTR<br>3'-gccggaCUAAGUGUUGUGGUCGa-5' miRNA<br>16 15 14 13 / 7 6 5 4 3 2<br>3'pairing Seed	ACTAGC<br>Imperfect		☐	M
83446 7mer-m8 83468<br>5'-gtaggTGAAAACCATCACCAGCt-3' UTR<br>3'-gccggACUAAGUGUUGUGGUCGa-5' miRNA<br>16 15 14 13 / 7 6 5 4 3 2<br>3'pairing Seed	CACCAGC<br>7mer-m8		☐	M T
95744 8mer 95764<br>5'-ctGCCTGTGTC-C-ACACCAGCa-3' UTR<br>3'-gcCGGACUAAGUGUUGUGGUCGa-5' miRNA<br>16 15 14 13 / 7 6 5 4 3 2<br>3'pairing Seed	CACCAGCA<br>8mer		☐	M T

2D Structure	Local AU	Position	Conservation	Predicted By
Imperfect match<br>9321 5'-cttgCTGTTTCACTGACTCCAGCt-3' UTR 9344<br>3'-gccgGACUAAGUG-UUGUGGUCGa-5' miRNA<br>3' pairing Seed	TCCAGC<br>Imperfect			M
Imperfect match<br>30047 5'-gtGTTTCATTTCCAGCACCAACc-3' UTR 30069<br>3'-gcCGGACUAAGUGUUGUGGUCGa-5' miRNA<br>3' pairing Seed	ACCAAC<br>Imperfect			M
Imperfect match<br>32091 5'-ctataTTATTCACAACACCAcaa-3' UTR 32113<br>3'-gccggACUAAGUGUUGUGGUcga-5' miRNA<br>3' pairing Seed	ACCACA<br>Imperfect			M
7mer-A1<br>37099 5'-ggtCATGCTT-GTAATACCAGCa-3' UTR 37120<br>3'-gccGGACUAAGUGUUGUGGUCGa-5' miRNA<br>3' pairing Seed	TACCAGCA<br>7mer-A1			M T
Imperfect match<br>50073 5'-tgacaccATTGCAC-ACACTAGCa-3' UTR 50095<br>3'-gccggacUAA-GUGUUGUGGUCGa-5' miRNA<br>3' pairing Seed	ACTAGC<br>Imperfect			M
7mer-m8<br>58838 5'-ttGCTTTCTCTTAT-CCACCAGCt-3' UTR 58860<br>3'-gcCGGACUA-AGUGUUGUGGUCGa-5' miRNA<br>3' pairing Seed	CACCAGC<br>7mer-m8			M T
7mer-A1<br>67470 5'-caGCTTGATCCACTTCACAAATATACCAGCa-3' UTR 67500<br>3'-gcCGGACU-----AAGUGU---UGUGGUCGa-5' miRNA<br>3' pairing Seed	TACCAGCA<br>7mer-A1			M T
Imperfect match<br>72421 5'-aaGCTTGATCTTACAGCACAAGCc-3' UTR 72444<br>3'-gcCGGACUA-AGUGUUGUGGUCGa-5' miRNA<br>3' pairing Seed	ACAAGC<br>Imperfect			M
Imperfect match<br>78229 5'-gtgaacacaTCAGAACATCAGCg-3' UTR 78251<br>3'-gccggacuaAGUGUUGUGGUCGa-5' miRNA<br>3' pairing Seed	ATCAGC<br>Imperfect			M
Imperfect match<br>85173 5'-tccaCTGGTTTTCACAACACTAGac-3' UTR 85197<br>3'-gccgGACU--AAGUGUUGUGGUCga-5' miRNA<br>3' pairing Seed	ACTAGA<br>Imperfect			M

2D Structure	Local AU	Position	Conservation	Predicted By
Offset 6mer<br>1532 5'-aaaagTGTTTCAAAATCACCAGat-3' UTR 1555<br>3'-gccggACUAAGUGUU-GUGGUCga-5' miRNA<br>16 15 14 13 7 6 5 4 3 2<br>3'pairing Seed	CACCAG<br>Offset 6mer			M
8mer<br>12371 5'-ccacaacATAGTCAGCACCAGCa-3' UTR 12393<br>3'-gccggacUAAGUGUUGUGGUCGa-5' miRNA<br>16 15 14 13 7 6 5 4 3 2<br>3'pairing Seed	CACCAGCA<br>8mer			M T
6mer<br>19041 5'-caagTTGACTCACAAAACCAGCt-3' UTR 19063<br>3'-gccgGACUAAGUGUUGUGGUCGa-5' miRNA<br>16 15 14 13 7 6 5 4 3 2<br>3'pairing Seed	ACCAGC<br>6mer			M
Imperfect match<br>19371 5'-ggGCTTGGGTCACTTCTCCAGCc -3' UTR 19393<br>3'-gcCGGACUAAGUGUUGUGGUCGa-5' miRNA<br>16 15 14 13 7 6 5 4 3 2<br>3'pairing Seed	TCCAGC<br>Imperfect			M
Imperfect match<br>19858 5'-acagCTGATTCTTCAGCCCCAGCt -3' UTR 19881<br>3'-gccgGACUAAG-UGUUGUGGUCGa-5' miRNA<br>16 15 14 13 7 6 5 4 3 2<br>3'pairing Seed	CCCAGC<br>Imperfect			M
Imperfect match<br>23326 5'-ttggtccATTTA-AACATCAGCa -3' UTR 23347<br>3'-gccggacUAAGUGUUGUGGUCGa-5' miRNA<br>16 15 14 13 7 6 5 4 3 2<br>3'pairing Seed	ATCAGC<br>Imperfect			M
Imperfect match<br>25051 5'-gtaCCTGAAGTCTCGCAACCCCAGCt -3' UTR 25076<br>3'-gccGGACU--A-AGUGUUGUGGUCGa-5' miRNA<br>16 15 14 13 7 6 5 4 3 2<br>3'pairing Seed	CCCAGC<br>Imperfect			M
8mer<br>25695 5'-gcaCCTG---TGTGTCACCAGCa-3' UTR 25714<br>3'-gccGGACUAAGUGUUGUGGUCGa-5' miRNA<br>16 15 14 13 7 6 5 4 3 2<br>3'pairing Seed	CACCAGCA<br>8mer			M T
Imperfect match<br>31310 5'-ttggtccATTTA-AACATCAGCa -3' UTR 31331<br>3'-gccggacUAAGUGUUGUGGUCGa-5' miRNA<br>16 15 14 13 7 6 5 4 3 2<br>3'pairing Seed	ATCAGC<br>Imperfect			M
8mer<br>40986 5'-acttagcATTC-C--CACCAGCa-3' UTR 41005<br>3'-gccggacUAAGUGUUGUGGUCGa-5' miRNA<br>16 15 14 13 7 6 5 4 3 2<br>3'pairing Seed	CACCAGCA<br>8mer			M T
Imperfect match<br>46215 5'-agGCAT-ATTCA-AGCACTAGCa -3' UTR 46235<br>3'-gcCGGACUAAGUGUUGUGGUCGa-5' miRNA<br>16 15 14 13 7 6 5 4 3 2<br>3'pairing Seed	ACTAGC<br>Imperfect			M
7mer-m8<br>50201 5'-cccaCTGAGCCATCTCACCAGCc-3' UTR 50223<br>3'-gccgGACUAAGUGUUGUGGUCGa-5' miRNA<br>16 15 14 13 7 6 5 4 3 2<br>3'pairing Seed	CACCAGC<br>7mer-m8			M T
Imperfect match<br>73667 5'-agGCAT-ATTCA-AGCACTAGCa -3' UTR 73687<br>3'-gcCGGACUAAGUGUUGUGGUCGa-5' miRNA<br>16 15 14 13 7 6 5 4 3 2<br>3'pairing Seed	ACTAGC<br>Imperfect			M

<table>
<tr><th>2D Structure</th><th>Local AU</th><th>Position</th><th>Conservation</th><th>Predicted By</th></tr>
<tr><td>12468 7mer-m8 12488
5'-agcCCTGCATCAT--CACCAGCt-3' UTR
|||| |||: |||||||
3'-gccGGACUAAGUGUUGUGGUCGa-5' miRNA
16 15 14 13 7 6 5 4 3 2
3'pairing Seed</td><td>CACCAGC
7mer-m8</td><td></td><td></td><td>M T</td></tr>
<tr><td>16367 7mer-m8 16389
5'-cttCCTGATTCTAAACACCAGCg-3' UTR
|||||||| ||||||||
3'-gccGGACUAAGUGUUGUGGUCGa-5' miRNA
16 15 14 13 7 6 5 4 3 2
3'pairing Seed</td><td>CACCAGC
7mer-m8</td><td></td><td></td><td>M T</td></tr>
<tr><td>16477 7mer-m8 16498
5'-agtCATAAATT-TGACACCAGCc-3' UTR
| | | |: ::|||||||
3'-gccGGACUAAGUGUUGUGGUCGa-5' miRNA
16 15 14 13 7 6 5 4 3 2
3'pairing Seed</td><td>CACCAGC
7mer-m8</td><td></td><td></td><td>M T</td></tr>
<tr><td>19867 7mer-A1 19889
5'-atGTCTGAGTAACCAGACCAGCa-3' UTR
|:|||| | || | ||||||
3'-gcCGGACUAAGUGUUGUGGUCGa-5' miRNA
16 15 14 13 7 6 5 4 3 2
3'pairing Seed</td><td>GACCAGCA
7mer-A1</td><td></td><td></td><td>M T</td></tr>
<tr><td>27379 8mer 27405
5'-gttgacGATTGCAGTTCCTCACCAGCa-3' UTR
|||| || | |||||||
3'-gccggaCUAA-GU---GUUGUGGUCGa-5' miRNA
16 15 14 13 7 6 5 4 3 2
3'pairing Seed</td><td>CACCAGCA
8mer</td><td></td><td></td><td>M T</td></tr>
<tr><td>Imperfect match
39797 39819
5'-taGCCTG--TCGCAGTTCACTAGCa -3' UTR
||||| ||:||: |||:|||
3'-gcCGGACUAAGUGUU--GUGGUCGa-5' miRNA
16 15 14 13 7 6 5 4 3 2
3'pairing Seed</td><td>ACTAGC
Imperfect</td><td></td><td></td><td>M</td></tr>
<tr><td>Imperfect match
43671 43693
5'-cctgCTGAGCCATGTCGCCAGCc -3' UTR
|||| ||:: |:|||||
3'-gccgGACUAAGUGUUGUGGUCGa-5' miRNA
16 15 14 13 7 6 5 4 3 2
3'pairing Seed</td><td>GCCAGC
Imperfect</td><td></td><td></td><td>M</td></tr>
<tr><td>58401 8mer 58427
5'-caGTCTCACTCTTCAGACACACCAGCa-3' UTR
|:|| | |||| | ||||||||
3'-gcCGGACU---AAGUGU-UGUGGUCGa-5' miRNA
16 15 14 13 7 6 5 4 3 2
3'pairing Seed</td><td>CACCAGCA
8mer</td><td></td><td></td><td>M T</td></tr>
<tr><td>Offset 6mer
63574 63598
5'-atGTTTGTTTCCACAGGCACCAGat-3' UTR
|::|| || |||| :||||||
3'-gcCGGACUAA-GUGU-UGUGGUCga-5' miRNA
16 15 14 13 7 6 5 4 3 2
3'pairing Seed</td><td>CACCAG
Offset 6mer</td><td></td><td></td><td>M</td></tr>
<tr><td>64566 7mer-m8 64590
5'-ccaCGTGGTCCTGAGGGCACCAGCt-3' UTR
| ||:| | | ::|||||||
3'-gccGGACUA--AGUGUUGUGGUCGa-5' miRNA
16 15 14 13 7 6 5 4 3 2
3'pairing Seed</td><td>CACCAGC
7mer-m8</td><td></td><td></td><td>M T</td></tr>
</table>

<table>
<tr><th>2D Structure</th><th>Local AU</th><th>Position</th><th>Conservation</th><th>Predicted By</th></tr>
<tr><td>Imperfect match
1105 1124
5'-cacCTGTGAT--GTGGCTGTTg -3' UTR
|:||||: | |:|||||
3'-accGGCACUGACCUCUGACAAu-5' miRNA
16 15 14 13 7 6 5 4 3 2
3'pairing Seed</td><td>GCTGTT
Imperfect</td><td></td><td></td><td>M</td></tr>
<tr><td>Imperfect match
2978 2997
5'-cacCTGTGAT--GTGGCTGTTg -3' UTR
|:||||: | |:|||||
3'-accGGCACUGACCUCUGACAAu-5' miRNA
16 15 14 13 7 6 5 4 3 2
3'pairing Seed</td><td>GCTGTT
Imperfect</td><td></td><td></td><td>M</td></tr>
<tr><td>Imperfect match
4851 4870
5'-cacCTGTGAT--GTGGCTGTTg -3' UTR
|:||||: | |:|||||
3'-accGGCACUGACCUCUGACAAu-5' miRNA
16 15 14 13 7 6 5 4 3 2
3'pairing Seed</td><td>GCTGTT
Imperfect</td><td></td><td></td><td>M</td></tr>
<tr><td>Imperfect match
6724 6743
5'-cacCTGTGAT--GTGGCTGTTg -3' UTR
|:||||: | |:|||||
3'-accGGCACUGACCUCUGACAAu-5' miRNA
16 15 14 13 7 6 5 4 3 2
3'pairing Seed</td><td>GCTGTT
Imperfect</td><td></td><td></td><td>M</td></tr>
<tr><td>Imperfect match
8597 8616
5'-cacCTGTGAT--GTGGCTGTTg -3' UTR
|:||||: | |:|||||
3'-accGGCACUGACCUCUGACAAu-5' miRNA
16 15 14 13 7 6 5 4 3 2
3'pairing Seed</td><td>GCTGTT
Imperfect</td><td></td><td></td><td>M</td></tr>
<tr><td>Imperfect match
10469 10488
5'-cacCTGTGAT--GTGGCTGTTg -3' UTR
|:||||: | |:|||||
3'-accGGCACUGACCUCUGACAAu-5' miRNA
16 15 14 13 7 6 5 4 3 2
3'pairing Seed</td><td>GCTGTT
Imperfect</td><td></td><td></td><td>M</td></tr>
<tr><td>Imperfect match
12342 12361
5'-cacCTGTGAT--GTGGCTGTTg -3' UTR
|:||||: | |:|||||
3'-accGGCACUGACCUCUGACAAu-5' miRNA
16 15 14 13 7 6 5 4 3 2
3'pairing Seed</td><td>GCTGTT
Imperfect</td><td></td><td></td><td>M</td></tr>
<tr><td>Imperfect match
14215 14234
5'-cacCTGTGAT--GTGGCTGTTg -3' UTR
|:||||: | |:|||||
3'-accGGCACUGACCUCUGACAAu-5' miRNA
16 15 14 13 7 6 5 4 3 2
3'pairing Seed</td><td>GCTGTT
Imperfect</td><td></td><td></td><td>M</td></tr>
<tr><td>Imperfect match
16087 16106
5'-cacCTGTGAT--GTGGCTGTTg -3' UTR
|:||||: | |:|||||
3'-accGGCACUGACCUCUGACAAu-5' miRNA
16 15 14 13 7 6 5 4 3 2
3'pairing Seed</td><td>GCTGTT
Imperfect</td><td></td><td></td><td>M</td></tr>
<tr><td>Imperfect match
17959 17978
5'-cacCTGTGAT--GTGGCTGTTg -3' UTR
|:||||: | |:|||||
3'-accGGCACUGACCUCUGACAAu-5' miRNA
16 15 14 13 7 6 5 4 3 2
3'pairing Seed</td><td>GCTGTT
Imperfect</td><td></td><td></td><td>M</td></tr>
<tr><td>Imperfect match
19837 19856
5'-cacCTGTGAT--GTGGCTGTTg -3' UTR
|:||||: | |:|||||
3'-accGGCACUGACCUCUGACAAu-5' miRNA
16 15 14 13 7 6 5 4 3 2
3'pairing Seed</td><td>GCTGTT
Imperfect</td><td></td><td></td><td>M</td></tr>
<tr><td>Imperfect match
21709 21728
5'-cacCTGTGAT--GTGGCTGTTg -3' UTR
|:||||: | |:|||||
3'-accGGCACUGACCUCUGACAAu-5' miRNA
16 15 14 13 7 6 5 4 3 2
3'pairing Seed</td><td>GCTGTT
Imperfect</td><td></td><td></td><td>M</td></tr>
<tr><td>Imperfect match
23581 23600
5'-cacCTGTGAT--GTGGCTGTTg -3' UTR
|:||||: | |:|||||
3'-accGGCACUGACCUCUGACAAu-5' miRNA
16 15 14 13 7 6 5 4 3 2
3'pairing Seed</td><td>GCTGTT
Imperfect</td><td></td><td></td><td>M</td></tr>
<tr><td>Imperfect match
25454 25473
5'-cacCTGTGAT--GTGGCTGTTg -3' UTR
|:||||: | |:|||||
3'-accGGCACUGACCUCUGACAAu-5' miRNA
16 15 14 13 7 6 5 4 3 2
3'pairing Seed</td><td>GCTGTT
Imperfect</td><td></td><td></td><td>M</td></tr>
<tr><td>Imperfect match
27326 27345
5'-cacCTGTGAT--GTGGCTGTTg -3' UTR
|:||||: | |:|||||
3'-accGGCACUGACCUCUGACAAu-5' miRNA
16 15 14 13 7 6 5 4 3 2
3'pairing Seed</td><td>GCTGTT
Imperfect</td><td></td><td></td><td>M</td></tr>
<tr><td>Imperfect match
36681 36700
5'-cacCTGTGAT--GTGGCTGTTg -3' UTR
|:||||: | |:|||||
3'-accGGCACUGACCUCUGACAAu-5' miRNA
16 15 14 13 7 6 5 4 3 2
3'pairing Seed</td><td>GCTGTT
Imperfect</td><td></td><td></td><td>M</td></tr>
<tr><td>Imperfect match
38553 38572
5'-cacCTGTGAT--GTGGCTGTTg -3' UTR
|:||||: | |:|||||
3'-accGGCACUGACCUCUGACAAu-5' miRNA
16 15 14 13 7 6 5 4 3 2
3'pairing Seed</td><td>GCTGTT
Imperfect</td><td></td><td></td><td>M</td></tr>
<tr><td>Imperfect match
40425 40444
5'-cacCTGTGAT--GTGGCTGTTg -3' UTR
|:||||: | |:|||||
3'-accGGCACUGACCUCUGACAAu-5' miRNA
16 15 14 13 7 6 5 4 3 2
3'pairing Seed</td><td>GCTGTT
Imperfect</td><td></td><td></td><td>M</td></tr>
<tr><td>Imperfect match
42297 42316
5'-cacCTGTGAT--GTGGCTGTTg -3' UTR
|:||||: | |:|||||
3'-accGGCACUGACCUCUGACAAu-5' miRNA
16 15 14 13 7 6 5 4 3 2
3'pairing Seed</td><td>GCTGTT
Imperfect</td><td></td><td></td><td>M</td></tr>
<tr><td>Imperfect match
44169 44188
5'-cacCTGTGAT--GTGGCTGTTg -3' UTR
|:||||: | |:|||||
3'-accGGCACUGACCUCUGACAAu-5' miRNA
16 15 14 13 7 6 5 4 3 2
3'pairing Seed</td><td>GCTGTT
Imperfect</td><td></td><td></td><td>M</td></tr>
<tr><td>Imperfect match
46041 46060
5'-cacCTGTGAT--GTGGCTGTTg -3' UTR
|:||||: | |:|||||
3'-accGGCACUGACCUCUGACAAu-5' miRNA
16 15 14 13 7 6 5 4 3 2
3'pairing Seed</td><td>GCTGTT
Imperfect</td><td></td><td></td><td>M</td></tr>
<tr><td>Imperfect match
47912 47931
5'-cacCTGTGAT--GTGGCTGTTg -3' UTR
|:||||: | |:|||||
3'-accGGCACUGACCUCUGACAAu-5' miRNA
16 15 14 13 7 6 5 4 3 2
3'pairing Seed</td><td>GCTGTT
Imperfect</td><td></td><td></td><td>M</td></tr>
<tr><td>Imperfect match
49785 49804
5'-cacCTGTGAT--GTGGCTGTTg -3' UTR
|:||||: | |:|||||
3'-accGGCACUGACCUCUGACAAu-5' miRNA
16 15 14 13 7 6 5 4 3 2
3'pairing Seed</td><td>GCTGTT
Imperfect</td><td></td><td></td><td>M</td></tr>
<tr><td>Imperfect match
51657 51676
5'-cacCTGTGAT--GTGGCTGTTg -3' UTR
|:||||: | |:|||||
3'-accGGCACUGACCUCUGACAAu-5' miRNA
16 15 14 13 7 6 5 4 3 2
3'pairing Seed</td><td>GCTGTT
Imperfect</td><td></td><td></td><td>M</td></tr>
<tr><td>Imperfect match
53529 53548
5'-cacCTGTGAT--GTGGCTGTTg -3' UTR
|:||||: | |:|||||
3'-accGGCACUGACCUCUGACAAu-5' miRNA
16 15 14 13 7 6 5 4 3 2
3'pairing Seed</td><td>GCTGTT
Imperfect</td><td></td><td></td><td>M</td></tr>
<tr><td>Imperfect match
55401 55420
5'-cacCTGTGAT--GTGGCTGTTg -3' UTR
|:||||: | |:|||||
3'-accGGCACUGACCUCUGACAAu-5' miRNA
16 15 14 13 7 6 5 4 3 2
3'pairing Seed</td><td>GCTGTT
Imperfect</td><td></td><td></td><td>M</td></tr>
<tr><td>Imperfect match
57274 57293
5'-cacCTGTGAT--GTGGCTGTTg -3' UTR
|:||||: | |:|||||
3'-accGGCACUGACCUCUGACAAu-5' miRNA
16 15 14 13 7 6 5 4 3 2
3'pairing Seed</td><td>GCTGTT
Imperfect</td><td></td><td></td><td>M</td></tr>
<tr><td>Imperfect match
59132 59151
5'-cacCTGTGAT--GTGGCTGTTg -3' UTR
|:||||: | |:|||||
3'-accGGCACUGACCUCUGACAAu-5' miRNA
16 15 14 13 7 6 5 4 3 2
3'pairing Seed</td><td>GCTGTT
Imperfect</td><td></td><td></td><td>M</td></tr>
<tr><td>Imperfect match
60999 61018
5'-cacCTGTGAT--GTGGCTGTTg -3' UTR
|:||||: | |:|||||
3'-accGGCACUGACCUCUGACAAu-5' miRNA
16 15 14 13 7 6 5 4 3 2
3'pairing Seed</td><td>GCTGTT
Imperfect</td><td></td><td></td><td>M</td></tr>
</table>
