# Supplementary material for: Cocaine induces differential circular RNA expression in striatum
Source: Transl Psychiatry. 2019 Aug 21;9:199. doi: 10.1038/s41398-019-0527-1 (PMC6704174; doi:10.1038/s41398-019-0527-1)
Supplement: Supplementary file 9 — Table S1 RT-PCR primers used to validate the microarray data. [file 41398_2019_527_MOESM9_ESM.pdf]

Supplementary Table 1 RT-PCR primers used to validate the microarray data

Primer name	Sequence	Product length(bp)
GAPDH-for	5’ GTTGTCTCCTGCGACTTCA3’	293
GAPDH-rev	5’ GCCCCTCCTGTTATTATGG3’	
mmu_circRNA_017361-for	5’ AAACTTGGCAGGCTACCTC3’	194
mmu_circRNA_017361-rev	5’ CAGACTGAACAGCAAATGATG3’	
mmu_circRNA_002179-for	5’ CAGAAAACATCATGCTGGTGGA3’	224
mmu_circRNA_002179-rev	5’ TCCCAGTTGGAACTTGGCTCT3’	
mmu_circRNA_003585-for	5’ ACGAGCAAAGGGAATAAGGA3’	105
mmu_circRNA_003585-rev	5’ TGTTGTGGTGTCAAGGCAAA3’	
mmu_circRNA_012342-for	5’ ACGGCCAGCAGTGCGAGTT3’	78
mmu_circRNA_012342-rev	5’ TCCTGCCCAGTCTGCCCAAG 3’	
mmu_circRNA_014222-for	5’ GTGGAAAGAAGCAAAACCTGA3’	175
mmu_circRNA_014222-rev	5’ GGCGAGGTGCTGTAGTCTTC3’	
