# Supplementary material for: Cocaine induces differential circular RNA expression in striatum
Source: Transl Psychiatry. 2019 Aug 21;9:199. doi: 10.1038/s41398-019-0527-1 (PMC6704174; doi:10.1038/s41398-019-0527-1)
Supplement: Supplementary file 10 — Table S2 List of the differentially expressed circRNAs. [file 41398_2019_527_MOESM10_ESM.pdf]

P-value	Fold change and Regulation		Annotations								miRNA Binding Sites				
P-value	FC (abs)	Regulation	circRNA	chrom	strand	txStart	txEnd	best_transcript	GeneSymbol	Sequence	MRE1	MRE2	MRE3	MRE4	MRE5
0.011707095	2.6294086	down	**mmu_circRNA_017196**	chr9	-	82945757	82959851	NM_001081216	Phip	GATATTTACTGTGAAGTATTATAGACACCTTGCACCTGATCACTTG	mmu-miR-128-3p	mmu-miR-7033-5p	mmu-miR-101a-3p	mmu-miR-7a-2-3p	mmu-miR-188-3p
0.000624858	2.4114366	down	**mmu_circRNA_017142**	chr6	+	65869180	65901859	NM_027547	Prdm5	CGTACTCACACCAGGATTCATGAGATATTTGATTGTCAAGAATGTA	mmu-miR-27a-3p	mmu-miR-27b-3p	mmu-miR-6964-3p	mmu-miR-6992-3p	mmu-miR-6979-3p
0.00444599	2.5322249	down	**mmu_circRNA_001863**	chr10	-	59599927	59600693	ENSMUST00000161435	Mcu	AAACTTGAACATTCGCAGGGCTTTCTTTCATGACCATTCCTCAGTG	mmu-miR-92a-2-5p	mmu-miR-7241-5p	mmu-miR-3057-3p	mmu-miR-6908-3p	mmu-miR-8096
0.003986671	2.5407522	down	**mmu_circRNA_013774**	chr13	-	103884142	103889285	NM_001005868	Erbb2ip	GTCAGCAAAAATGAATGTTGGCTCAATTAAGAAACATCAGGGAGAT	mmu-miR-21b	mmu-miR-6399	mmu-miR-32-3p	mmu-miR-450b-3p	mmu-miR-450a-1-3p
0.000691721	2.3070231	down	**mmu_circRNA_009232**	chr9	+	22655224	22679064	NM_178415	Bbs9	ATACAAGCAGGTATTCCTCGGGTTGTCCAATGTAAATTTAGACTTC	mmu-miR-7049-5p	mmu-miR-7653-5p	mmu-miR-3084-5p	mmu-miR-294-5p	mmu-miR-346-3p
0.000116904	2.8664544	down	**mmu_circRNA_007027**	chr9	+	32170089	32208265	NM_001195632	Arhgap32	CCAAAACCAGCTCTCACTATCAGAAGAACAGAATGAAGTAATGAAA	mmu-miR-7025-5p	mmu-miR-335-3p	mmu-miR-7226-5p	mmu-miR-1224-3p	mmu-miR-6954-5p
0.002689008	2.3925386	down	**mmu_circRNA_002236**	chr6	+	72128263	72132163	ENSMUST00000069994	St3gal5	CCGGATAAAGCAATGCCAAGTGAGTTCACCTCTGCAAAGCTGAGAA	mmu-miR-7669-3p	mmu-miR-5621-5p	mmu-miR-1943-5p	mmu-miR-7659-5p	mmu-miR-7662-3p
0.004490171	2.1685599	down	**mmu_circRNA_015248**	chr7	-	99935161	99955540	NM_175388	Rnf169	TCCTGGCAGGAGTTTATATTCAGAACGCCAATCAAGTTAAGCAAGC	mmu-miR-3095-5p	mmu-miR-693-3p	mmu-miR-207	mmu-miR-6344	mmu-miR-7038-3p
0.000354957	2.3229132	down	**mmu_circRNA_016623**	chr1	-	8583204	8607152	NM_027671	Sntg1	TTTCAAACCTCACAAAGCACAATGTGCTCCAAGTGACCAAAGCAGT	mmu-miR-7674-5p	mmu-miR-666-5p	mmu-miR-3061-5p	mmu-miR-7031-5p	mmu-miR-8111
0.000336815	2.0898834	down	**mmu_circRNA_011031**	chr3	-	120772681	120799747	TCONS_00021080	XLOC_016164	TGCATGACAAGTTTCTTCCTAAGCTGAGTGAATCTGGACACTTTCT	mmu-miR-7062-5p	mmu-miR-7116-3p	mmu-miR-106a-3p	mmu-miR-691	mmu-miR-7093-3p
5.35614E-05	2.2238249	down	**mmu_circRNA_007295**	chr11	-	85077264	85104026	NM_001029934	Usp32	GTCACACATTTTACAGATGTTGAGCTGAAACGACTCAAAGATGCCT	mmu-miR-670-5p	mmu-miR-6967-3p	mmu-miR-299b-5p	mmu-miR-335-3p	mmu-miR-7019-5p
0.016079049	3.2871183	down	**mmu_circRNA_012180**	chr8	+	85896429	85922242	NM_199446	Phkb	TGGTAGTACCGAACTGCACTCAAGGTCCAACAGTTTAAACAAGATC	mmu-miR-7014-3p	mmu-miR-7231-3p	mmu-miR-759	mmu-miR-130b-5p	mmu-miR-6972-5p
7.63396E-05	2.0321594	down	**mmu_circRNA_014015**	chr18	-	6111684	6115850	NM_001039692	Arhgap12	GCAGATGAGGGTTTAAATGAAACACCAGATGTTTACAGCCTTCCAA	mmu-miR-30c-1-3p	mmu-miR-450a-5p	mmu-miR-30c-2-3p	mmu-miR-592-5p	mmu-miR-320-5p
3.35234E-05	2.1856347	down	**mmu_circRNA_004501**	chr9	-	65794621	65795495	NM_172536	Zfp609	AGTCAAGTCTGAAAAGCAATGATGTTGTCCACTGGACATGTTCTGA	mmu-miR-7009-3p	mmu-miR-3971	mmu-miR-6974-3p	mmu-miR-6975-3p	mmu-miR-6961-3p
0.002714098	2.3521293	down	**mmu_circRNA_001407**	chr12	+	3617605	3686862	NM_001162465	Dtnb	CACATCTAGTTGAGGGCCGAGGCAAGTTGACCGTGTTTTCAGTTAA	mmu-miR-3066-5p	mmu-miR-6938-3p	mmu-miR-6929-5p	mmu-miR-103-1-5p	mmu-miR-103-2-5p
0.001738812	2.009608	down	**mmu_circRNA_001009**	chr19	-	4784723	4794009			ATCTTTGGGAAAAATGAAGGTAATTGGCGTTCCGGGTAACCAATCA	mmu-miR-7007-5p	mmu-miR-6946-5p	mmu-miR-6418-5p	mmu-miR-8100	mmu-miR-7072-5p
3.39058E-05	2.2316757	down	**mmu_circRNA_000155**	chr16	+	33421320	33434974	NM_011749	Zfp148	CAATGTCCCTGATAGAAGAGAAGAATCAGTGGTTTGGAAATTTAGC	mmu-miR-17-3p	mmu-miR-6925-5p	mmu-miR-106a-3p	mmu-let-7j	mmu-miR-20b-3p
0.005679453	2.3331492	down	**mmu_circRNA_003195**	chr5	+	33843111	33846267	NM_001081102	Whsc1	TGACCCACTCCTTCACAATCATACCAAACTTAAAGTGTTCTAAGAA	mmu-miR-6340	mmu-miR-7011-3p	mmu-miR-8103	mmu-miR-181d-5p	mmu-miR-329-5p
0.000892024	2.470038	down	**mmu_circRNA_010490**	chr8	+	120553583	120553802	NM_001145896	Gse1	AGAGGAGCCCAGAGGCATGAGCCATGAGCCCAAGTCCCCTTCCATA	mmu-miR-6911-5p	mmu-miR-3102-5p	mmu-miR-6982-5p	mmu-miR-1946a	mmu-miR-6347
2.92176E-06	5.34828	down	**mmu_circRNA_014222**	chr17	-	65592290	65595054	NM_013933	Vapa	TGATAAGCTGGCCCCTTCACAGATGTAGTCACTACAAATCTTAAAT	mmu-miR-1298-5p	mmu-miR-672-3p	mmu-miR-19b-1-5p	mmu-miR-7212-3p	mmu-miR-19b-2-5p
8.19861E-06	2.2311364	down	**mmu_circRNA_003388**	chr1	-	119721863	119765922	NM_019933	Ptpn4	TCCGGTACTGGTATCAGTACTTTTTGCAAATTAAGCAAGACATTCT	mmu-miR-7214-5p	mmu-miR-876-5p	mmu-miR-196a-5p	mmu-miR-186-5p	mmu-miR-7039-3p
0.00622242	2.0590802	down	**mmu_circRNA_001535**	chr1	-	53256628	53282092	NM_153556	Pms1	ACATAATAAGCTACTCAGGTGCAAGCTAGGATGAAACAGTTGCCTG	mmu-miR-6902-5p	mmu-miR-7026-5p	mmu-miR-344e-5p	mmu-miR-344h-5p	mmu-miR-219a-5p
0.0067951	2.6380816	down	**mmu_circRNA_004122**	chr17	+	26739542	26743131	NM_029870	Crebrf	TATCAGCGACACGTTCTCTGAACCAGCCCAGCGTAAGCGGAATGGA	mmu-miR-3544-5p	mmu-miR-1903	mmu-miR-6961-3p	mmu-miR-6974-3p	mmu-miR-26b-3p
2.0081E-06	2.0822771	down	**mmu_circRNA_017361**	chr1	-	119765030	119765922	NM_019933	Ptpn4	AGCAGCACGTGTATCAGTACTTTTTGCAAATTAAGCAAGACATTCT	mmu-miR-7678-5p	mmu-miR-6948-5p	mmu-miR-141-5p	mmu-miR-7063-5p	mmu-miR-6970-5p
0.00021611	2.1645173	down	**mmu_circRNA_002352**	chr1	+	139039999	139062971	NM_001166501	Dennd1b	ATTATTACCTCGAGCAAATTAAGCACTGAAGTTCTGCAGAGCGTGC	mmu-miR-145a-5p	mmu-miR-145b	mmu-miR-6383	mmu-miR-93-5p	mmu-miR-122-5p
0.000155019	2.1142667	down	**mmu_circRNA_015175**	chr6	-	31139728	31181149			GTAAGCCACCAAAGAGGCATCTCGTTGATTCCTAGCGTTAGAAGAA	mmu-miR-1187	mmu-miR-466a-5p	mmu-miR-466e-5p	mmu-miR-207	mmu-miR-466p-5p
0.000505211	2.1318865	down	**mmu_circRNA_018765**	chr16	+	33421320	33468183	NM_011749	Zfp148	ATCCATACCGGATAGAAGAGAAGAATCAGTGGTTTGGAAATTTAGC	mmu-miR-17-3p	mmu-miR-7660-3p	mmu-miR-7235-3p	mmu-miR-1896	mmu-miR-149-5p
0.000183545	2.186618	down	**mmu_circRNA_016108**	chr8	-	64811679	64834568	ENSMUST00000034017	Klhl2	GAACGTGCAGATGCACAAAGCAGGGTCATCAGAAGCCCCTCGATTC	mmu-miR-6961-3p	mmu-miR-216b-5p	mmu-miR-6957-3p	mmu-miR-692	mmu-miR-770-5p
0.006613796	2.1045624	down	**mmu_circRNA_011374**	chr3	-	127046724	127057091	uc008rgv.1	Ank2	ATTGATGCCAAAACTAGGAATGGCTTCACTCCTTTGTACATGGCTG	mmu-miR-5113	mmu-miR-298-5p	mmu-miR-7086-5p	mmu-miR-466h-5p	mmu-miR-8091
0.004340324	2.9308863	down	**mmu_circRNA_014984**	chr4	+	150468694	150510038	NM_001085492	Rere	GCACTGAACACAAGTAAGAGGGACCATCTACTCATGAACGTCAAAT	mmu-miR-5110	mmu-miR-6353	mmu-miR-27b-3p	mmu-miR-322-5p	mmu-miR-27a-3p
0.001172887	2.1802234	down	**mmu_circRNA_009519**	chr9	+	3634438	3635157	NM_001033322	Gucy1a2	TGAAGATAAGGACTGCAGTTCCAAGAAATTCAAGAAAGATTTGGTG	mmu-miR-291a-5p	mmu-miR-291b-5p	mmu-miR-1958	mmu-miR-3071-3p	mmu-miR-7014-3p
1.01471E-05	2.0449228	down	**mmu_circRNA_013873**	chr14	+	35321640	35323434	NM_008166	Grid1	ACATGCGCAAGATCTCCAATCTCTACCTGTATGACAGTGTTCTGAT	mmu-miR-8095	mmu-miR-6237	mmu-miR-7685-5p	mmu-miR-1930-5p	mmu-miR-338-3p
5.31245E-06	2.2049683	down	**mmu_circRNA_001729**	chr9	-	96591949	96611499	NM_053268	Rasa2	GCCAGCAGCTTGTTGTACAATGAAGCAAAAAATTTATTGCCATATC	mmu-miR-147-5p	mmu-miR-6481	mmu-miR-17-3p	mmu-miR-1903	mmu-miR-743b-3p
2.5089E-07	2.0213545	down	**mmu_circRNA_012342**	chr14	-	50807499	50807650			AGTTCAATGGCTGAGGTGAGTTCCCAGAGAGCAGGGCTCTACGCTT	mmu-miR-3103-5p	mmu-miR-7235-5p	mmu-miR-6965-5p	mmu-miR-1943-5p	mmu-miR-6967-5p
0.000144951	2.3706375	down	**mmu_circRNA_005522**	chr6	+	31418930	31451584	NM_013791	Mkln1	GTCCAGACAGGAACATTTTAGTGGATAAACCAAATGATCAGTCTTC	mmu-miR-296-5p	mmu-miR-8103	mmu-miR-135a-5p	mmu-miR-135b-5p	mmu-miR-30d-3p
0.013243658	2.0710625	down	**mmu_circRNA_006097**	chr2	+	92214048	92230724	NM_001109691	Phf21a	AAATCACAGCTTTGAGTGAGAAACAGGGGACTGGAGAGCTGAAGGA	mmu-miR-432	mmu-miR-6971-3p	mmu-miR-448-3p	mmu-miR-337-3p	mmu-miR-16-1-3p
0.000107617	2.1513459	down	**mmu_circRNA_001794**	chr13	-	9720772	9735466	NM_001199141	Zmynd11	GAGATGAGATTCTAAAGAAGTAAACAGGTCATGGCACGTTTAACAA	mmu-miR-208a-5p	mmu-miR-6415	mmu-miR-208b-5p	mmu-miR-129b-5p	mmu-miR-693-3p
0.011271087	2.2322281	down	**mmu_circRNA_016411**	chr2	+	158035995	158058827	NM_027434	Rprd1b	GAAAAAACTAGAACCAAATCAAATAGAAAGCTTACTTTTCTATACT	mmu-miR-6958-3p	mmu-miR-6999-3p	mmu-miR-6961-3p	mmu-miR-1903	mmu-miR-6980-5p
9.96067E-06	2.6887822	down	**mmu_circRNA_001434**	chr17	+	70761074	70787209	NM_001128180	Dlgap1	CCAGAAGACATGTCATCTTGCATTACAACATATAAGAAGACACCAC	mmu-miR-207	mmu-miR-667-5p	mmu-miR-7677-5p	mmu-miR-7217-5p	mmu-miR-7044-5p
0.000965354	2.4746568	down	**mmu_circRNA_016242**	chr16	-	11116794	11128703	NM_029582	Txndc11	TCTCTCCAGCTATGAGTCGAGCAAAAGACGTGATAATACCAGCAAA	mmu-miR-21a-3p	mmu-miR-5110	mmu-miR-7038-5p	mmu-miR-331-3p	mmu-miR-3064-3p
0.001807778	4.0761675	down	**mmu_circRNA_000551**	chr7	+	16883126	16883510	NM_001081655	Dact3	ATCTATGCCAGTGAGAGACCCAAGTCCTTAGGAGGCCCTGCCTGGG	mmu-miR-7080-5p	mmu-miR-7009-5p	mmu-miR-7038-5p	mmu-miR-7662-3p	mmu-miR-210-5p
0.007372002	2.0944049	down	**mmu_circRNA_006905**	chr15	+	39506965	39517871	NM_001256382	Rims2	GGGATGGAAGGGTCGAAGAGAATAAGTGACAGTGAAGTGTCTGACT	mmu-miR-6899-3p	mmu-miR-6981-5p	mmu-miR-5110	mmu-miR-489-3p	mmu-miR-3102-3p.2-3p
0.012062301	2.2127551	down	**mmu_circRNA_013905**	chr19	-	5798105	5798224			GATGGTCAGAGCAGGAAAGACAAATTTTATTCTCCATGCAGTGATG	mmu-miR-3106-3p	mmu-miR-532-3p	mmu-miR-344d-3-5p	mmu-miR-101a-5p	mmu-miR-7000-3p
0.000374905	2.1252426	down	**mmu_circRNA_009558**	chr1	+	139053345	139062971	NM_001166501	Dennd1b	CCTCGAGCAAATTAAGCACTTTACTTGCCATGGTTTGAAGTATATT	mmu-miR-145a-5p	mmu-miR-145b	mmu-miR-122-5p	mmu-miR-103-3p	mmu-miR-107-3p
4.25464E-06	2.234184	down	**mmu_circRNA_017958**	chr10	+	90895098	90923602	uc007gsz.2	Anks1b	GCAAAGATGCGGGTTTTAAAAATCAGTTTGATTGGCCACAGGAAAC	mmu-miR-5110	mmu-miR-6900-3p	mmu-miR-3473c	mmu-miR-6985-5p	mmu-miR-468-3p
0.028331087	2.3768551	down	**mmu_circRNA_010312**	chr2	-	24719044	24720056	uc008ipf.1	Cacna1b	AAGAATGCAGAAGAGAAATCCCCTTTGGATGAGAGTTTGCCAAGGA	mmu-miR-7052-3p	mmu-miR-6934-3p	mmu-miR-6964-3p	mmu-miR-3098-3p	mmu-miR-544-3p
0.005733651	2.0953514	down	**mmu_circRNA_017369**	chr4	+	150439342	150477468	NM_001085492	Rere	TTTTCTACATATTAGGATACAACCCTGAGACAAGGTCCACAGCTCC	mmu-miR-6353	mmu-miR-322-5p	mmu-miR-6419	mmu-miR-667-5p	mmu-miR-1907
0.000158178	2.6111829	down	**mmu_circRNA_009396**	chr6	+	31418930	31433131	NM_013791	Mkln1	GCGAGGAGTTGATTGAAAAAGCTGTAAATGGAACATTTTAGTGGAT	mmu-miR-30d-3p	mmu-miR-3071-3p	mmu-miR-7214-5p	mmu-miR-5615-5p	mmu-miR-7b-5p
0.002283503	2.4233001	down	**mmu_circRNA_016955**	chr2	+	69914413	69922933	NM_001081548	Ubr3	GCAGATAGACTCTTCAGATGAGGTTTTGTAGAAGACATCAGATTAA	mmu-miR-344i	mmu-miR-6402	mmu-miR-320-5p	mmu-miR-6948-5p	mmu-miR-763
0.000600386	2.0106362	down	**mmu_circRNA_015645**	chr17	-	66024075	66053091	NM_001025572	Ankrd12	AGTTATACAGATCCAGGATGAGAAGACTGTTAGAAGAAGCAGCTAG	mmu-miR-7092-3p	mmu-miR-683	mmu-miR-6944-3p	mmu-miR-7684-3p	mmu-miR-6899-3p
0.007319113	2.0191177	down	**mmu_circRNA_003783**	chr12	+	102991349	103021876	NM_001081017	Unc79	CAGTACACAGTTGCTTCCAAGATCCGGTACTTGCAGGAATATCATA	mmu-miR-1966-5p	mmu-miR-302d-3p	mmu-miR-17-5p	mmu-miR-7016-5p	mmu-miR-344h-5p
0.003330401	2.3860661	down	**mmu_circRNA_016934**	chr7	-	59479061	59481464			AGGTAATCAGGAAAGACATTCTTCCAAGTTGCTTCCCTGGAAGATA	mmu-miR-6955-5p	mmu-miR-7665-5p	mmu-miR-6379	mmu-miR-6988-3p	mmu-miR-5621-5p
5.36413E-06	2.609846	down	**mmu_circRNA_017561**	chr1	+	128193459	128223641	NM_181750	R3hdm1	GTATGGACTTTAGCAACATGGATAATATTGTTCAGGATAACCTTGG	mmu-miR-7656-3p	mmu-miR-7679-5p	mmu-miR-3064-5p	mmu-miR-546	mmu-miR-6997-5p
2.39769E-05	2.2413073	down	**mmu_circRNA_018162**	chr17	-	80653861	80655921	NM_001081357	Map4k3	AGATATAAAGGCGAGATAAACTTTGGATTTGCATGGAATTCTGTGG	mmu-miR-7681-3p	mmu-miR-1968-3p	mmu-miR-5098	mmu-miR-146a-3p	mmu-miR-374c-5p
0.015498941	2.5644223	down	**mmu_circRNA_014309**	chr14	-	70678078	70682850			TCGAATGGATTAGCGTAAGGAAGTTAGTGAAGCTTAGCGCTGTACA	mmu-miR-7661-5p	mmu-miR-322-5p	mmu-miR-1960	mmu-miR-466a-5p	mmu-miR-466e-5p
0.000200738	2.1706575	down	**mmu_circRNA_005155**	chr16	+	72933212	72935786	NM_019413	Robo1	ACAGTTCAAGATACGAGGAGGAAAACTCATGATCACTTATACGCGT	mmu-miR-3095-5p	mmu-miR-193a-5p	mmu-miR-7211-5p	mmu-miR-7685-5p	mmu-miR-7213-5p
0.000200038	2.0165531	down	**mmu_circRNA_004868**	chr10	-	12439626	12455564	NM_011682	Utrn	TTGTTGGGTTCAGTGGATCTCTTAGAGCTGAATACGACGAATGAAG	mmu-miR-6958-3p	mmu-miR-1968-5p	mmu-miR-22-3p	mmu-miR-19b-2-5p	mmu-miR-450b-5p
0.000230895	2.1253088	down	**mmu_circRNA_012594**	chr1	+	140362948	140383095	NM_001081027	Kcnt2	TTATCTTCACTTGGTCTCACATATTTTGGGTTAACCGAAGTCTACC	mmu-miR-883b-5p	mmu-miR-8104	mmu-miR-503-3p	mmu-miR-7210-5p	mmu-miR-551b-5p
0.000141374	2.4439415	down	**mmu_circRNA_014302**	chr17	-	81647808	81649638	NM_001112798	Slc8a1	ATGAAATAGTGTTGGAGCAGTTGGAAGTCTTATTGTACAACATGCT	mmu-miR-6916-3p	mmu-miR-7033-5p	mmu-miR-8113	mmu-miR-7657-3p	mmu-miR-6344
0.003151016	2.0808711	down	**mmu_circRNA_006733**	chr9	-	96582742	96611499	NM_053268	Rasa2	GGGCCCCTCTAGATGAAGCAAAAAATTTATTGCCATATCTTGGACC	mmu-miR-7650-3p	mmu-miR-147-5p	mmu-miR-673-5p	mmu-miR-6481	mmu-miR-17-3p
0.000412077	2.1088759	down	**mmu_circRNA_016636**	chr12	-	100415134	100495440	NM_001033213	Ttc7b	TCAGGAGAGAATCAGAATTCCTACAAGAATCCAATCTGGTCATGGC	mmu-miR-7239-3p	mmu-miR-6408	mmu-miR-877-5p	mmu-miR-149-3p	mmu-miR-7047-5p
3.9765E-05	2.2220323	down	**mmu_circRNA_009056**	chr10	-	12436280	12455564	NM_011682	Utrn	ATACTGCATACCGTGGATCTCTTAGAGCTGAATACGACGAATGAAG	mmu-miR-6958-3p	mmu-miR-1968-5p	mmu-miR-22-3p	mmu-miR-328-3p	mmu-miR-19b-2-5p
0.001888681	2.3763162	down	**mmu_circRNA_017753**	chr17	+	28713773	28715441	NM_001168508	Mapk14	AATTCAATGACGTTGCTGCTTTTGATACAAAGACGGGGCATCGTGT	mmu-miR-6409	mmu-miR-103-2-5p	mmu-miR-103-1-5p	mmu-miR-7675-3p	mmu-miR-196b-3p
3.2007E-07	2.0180657	down	**mmu_circRNA_003585**	chr9	-	62912749	62923655	NM_019663	Pias1	AAGGAATCCGGATCATTCTAGAGCTTTAACTTCAGACAACAGCCAG	mmu-miR-29b-2-5p	mmu-miR-3093-3p	mmu-miR-6983-5p	mmu-miR-7078-5p	mmu-miR-705
6.64922E-05	2.0693605	down	**mmu_circRNA_016043**	chr1	+	140362948	140450304	NM_001081027	Kcnt2	ATCAAATTTGCAGGTCTCACATATTTTGGGTTAACCGAAGTCTACC	mmu-miR-8104	mmu-miR-465a-5p	mmu-miR-465d-5p	mmu-miR-7216-3p	mmu-miR-326-5p
0.000120303	2.3401861	down	**mmu_circRNA_012091**	chr12	-	111858209	111872433	NM_011625	Ppp1r13b	TGAAAATGGGATGATATTAACCGTGTTCTTGAGCAACAATGAACAG	mmu-miR-7649-3p	mmu-miR-6946-3p	mmu-miR-7083-3p	mmu-miR-3473c	mmu-miR-1903
0.002192403	2.0773416	down	**mmu_circRNA_015633**	chr13	-	23551474	23581741			TCTCCGGTCTCATCTACGAGGAAACCCGCGATGCGCTTCACTCCTC	mmu-miR-7092-3p	mmu-miR-7116-3p	mmu-miR-7054-5p	mmu-miR-466k	mmu-miR-7026-3p
0.000507827	2.6103213	down	**mmu_circRNA_006083**	chr1	-	119773048	119783589	NM_019933	Ptpn4	AGTATACAAGAAACATGATCAGGGGCAAGTTCTGTTGGATATAGTC	mmu-miR-3094-5p	mmu-miR-3058-5p	mmu-miR-551b-5p	mmu-miR-186-5p	mmu-miR-3084-5p
0.00161692	2.5838289	down	**mmu_circRNA_016732**	chr19	-	45885951	45892148	NM_198296	9130011E15Rik	GGTGAATATCAGCCTTGATAAACTTGCTGAAGTTCCTTATGTCAAA	mmu-miR-1970	mmu-miR-599	mmu-miR-3101-3p	mmu-miR-466f	mmu-miR-7686-5p
0.006037011	2.7000069	down	**mmu_circRNA_017690**	chr4	+	13835632	13846957	NM_001111026	Runx1t1	ACTCCAGACAGATCGTACCGAGAAGCACTCCACAATGCCAGACTCA	mmu-miR-7023-5p	mmu-miR-6954-5p	mmu-miR-93-3p	mmu-miR-7117-5p	mmu-miR-7030-5p
0.000366483	2.0116653	down	**mmu_circRNA_013587**	chr5	-	110755396	110756766	NM_029337	Ep400	GAAGGAAAATCTGAAGTTATCAACGATGAGGGGAACAGTACATTGG	mmu-miR-6940-5p	mmu-miR-6914-5p	mmu-miR-92a-2-5p	mmu-miR-138-5p	mmu-miR-1903
0.001654365	2.3090491	down	**mmu_circRNA_010647**	chr3	+	29148346	29153601	NM_001167748	Egfem1	AAGGATTTCATGGACCCCACTGTCAATATGGCCAAATGTTTGCAGA	mmu-miR-8119	mmu-miR-3065-3p	mmu-miR-3091-3p	mmu-miR-362-5p	mmu-miR-200c-5p
0.006762375	3.861374	up	**mmu_circRNA_000550**	chr1	-	66801048	66807604	uc011wml.1	Kansl1l	ATGTGGCAGTATGTTTTTAAGAGTTTAAGCTTTTACATGTAGCCCA	mmu-miR-3089-3p	mmu-miR-1968-5p	mmu-miR-5623-5p	mmu-miR-6938-3p	mmu-miR-5618-5p
0.00503462	3.1411233	up	**mmu_circRNA_014597**	chr1	+	163848660	163888305	NM_010629	Kifap3	TTCCTGGCAGGCAATGAAAACTACAAACAAATAGCAATGTGTGTGC	mmu-miR-3112-3p	mmu-miR-712-5p	mmu-miR-335-3p	mmu-miR-3083-5p	mmu-miR-679-3p
0.02827145	2.2100387	up	**mmu_circRNA_014394**	chr5	+	81465194	81560943	NM_198702	Lphn3	CCTGTCAGAGATGCAATAACAGAACCCAGTGTGCAGTGGTGGCAGG	mmu-miR-455-3p	mmu-miR-7647-3p	mmu-miR-1956	mmu-miR-7657-3p	mmu-miR-6915-5p
0.039135052	2.2440572	up	**mmu_circRNA_015224**	chr8	-	80800100	80800395	NM_021356	Gab1	CCCACAGAAGAAGGCGTGGAAGAGAAGGTGGTTTGTGTTGCGCAGT	mmu-miR-7219-3p	mmu-miR-144-5p	mmu-miR-670-3p	mmu-miR-6902-5p	mmu-miR-344e-5p
0.008877264	2.532564	up	**mmu_circRNA_018880**	chr5	+	21455781	21459293	NM_177869	Fam185a	ACGGTTACAGATTCGTCCTGTGGATGTCTAAAAGCCTCCAGTCATC	mmu-miR-1903	mmu-miR-103-2-5p	mmu-miR-103-1-5p	mmu-miR-107-5p	mmu-miR-6924-5p
0.02062254	3.330475	up	**mmu_circRNA_004259**	chr2	+	119946171	119961970	NM_001164274	Mga	TACTATGTCTCAGTCTGAAAATGCCCCTCAAATTCCAGTGACTACT	mmu-miR-7092-3p	mmu-miR-7007-5p	mmu-miR-298-5p	mmu-miR-7682-3p	mmu-miR-7090-5p
0.000699718	6.012606	up	**mmu_circRNA_007240**	chr5	+	32001360	32007317	NM_144541	Bre	TGCTGATGTGGAAAGACTTTTGTTTTCTTGTCCACATGCACTCGGA	mmu-miR-6985-5p	mmu-miR-6365	mmu-miR-6340	mmu-miR-497a-5p	mmu-miR-16-5p
0.00725595	3.3883055	up	**mmu_circRNA_006839**	chr17	+	78259863	78260066			TTGAGGGGATTTTTGTGGTTTCCAGTTTGAACCTGTGTCAGTACAG	mmu-miR-6948-3p	mmu-miR-1934-5p	mmu-miR-7117-3p	mmu-miR-1947-3p	mmu-miR-92a-2-5p
0.009243796	2.8410782	up	**mmu_circRNA_003880**	chr11	+	60706192	60706355	NM_001159404	Llgl1	AGGTAACCAGTGTGCCAGATGATTACCGGTGTGGAAAGGCCTTGGG	mmu-miR-6992-5p	mmu-miR-7221-3p	mmu-miR-6388	mmu-miR-7029-3p	mmu-miR-6379
0.001550779	2.2948171	up	**mmu_circRNA_002179**	chr6	-	38818229	38819313	NM_001136065	Hipk2	GATACTACCGGTATGGCCTCACATGTGCAAGTTTTCTCCCCTCACA	mmu-miR-1892	mmu-miR-6929-3p	mmu-miR-101c	mmu-miR-327	mmu-miR-203-5p
0.0091627	3.1200601	up	**mmu_circRNA_008640**	chr6	+	55460156	55461614	ENSMUST00000070736	Adcyap1r1	ATGAGTCTTCCCCAGCGCTGCATGCACAGTGGAGGGTGGTGACTGA	mmu-miR-504-3p	mmu-miR-7216-5p	mmu-miR-5623-3p	mmu-miR-7649-3p	mmu-miR-3472
0.01889726	2.3087798	up	**mmu_circRNA_000916**	chr7	-	4643000	4643555	NM_172894	Ppp6r1	ATCAAAGGATGACAATGTACCCTAGTGTAGCCTGTGAAATCCTGAC	mmu-miR-6901-5p	mmu-miR-6918-5p	mmu-miR-1190	mmu-miR-1197-3p	mmu-miR-671-5p
0.003212462	2.2419553	up	**mmu_circRNA_003834**	chr13	-	67596648	67673093			ATGACAATCTTGTGTTCCTGGGTCTTGCTTCGTCTAAGCCATATCT	mmu-miR-7012-5p	mmu-miR-5113	mmu-miR-7665-5p	mmu-miR-6976-5p	mmu-miR-6946-5p
0.00196586	3.4966493	up	**mmu_circRNA_014444**	chr4	-	86600750	86604289	NM_173400	Haus6	GATTCAAAAGGCTGAATGTGGGAGTAGTTTTCCTCAAGTTGTTGGC	mmu-miR-135b-5p	mmu-miR-3618-3p	mmu-miR-135a-5p	mmu-miR-7091-3p	mmu-miR-7241-5p
0.006602972	2.0413383	up	**mmu_circRNA_016771**	chr2	-	60779730	60792780	NM_001141931	Rbms1	AGGAGTTTCTGGTTATGGTTTTGTTGACTTTGACAGTCCTGCAGCA	mmu-miR-7239-5p	mmu-miR-873b	mmu-miR-7679-5p	mmu-miR-136-5p	mmu-miR-92a-2-5p
0.0167755	2.7109924	up	**mmu_circRNA_014144**	chr8	+	108856721	108856953	NM_007496	Zfhx3	ACTGACCCAGCACCTGCAGCAGCATGAGAGTGGCGTGGAGGGTGAG	mmu-miR-5130	mmu-miR-6996-5p	mmu-miR-377-5p	mmu-miR-7071-5p	mmu-miR-7036a-3p
0.044937367	2.6314573	up	**mmu_circRNA_016319**	chr11	+	121602987	121605340	NM_029878	Tbcd	TCTTTACTGCCGAGGAGAACTGTGAGCGCGTCATGTGTTGCGTTGC	mmu-miR-3474	mmu-miR-7239-3p	mmu-miR-6945-5p	mmu-miR-1982-5p	mmu-miR-204-3p
0.044410057	2.3667844	up	**mmu_circRNA_010848**	chr7	-	34157978	34158939	NM_016682	Uba2	GAGGCTGCTTGGTGTGACTGAGTGTTACGAATGTCACCCTAAGCCT	mmu-miR-7649-3p	mmu-miR-6399	mmu-miR-7226-5p	mmu-miR-380-5p	mmu-miR-3090-5p
