# Supplementary material for: Cocaine induces differential circular RNA expression in striatum
Source: Transl Psychiatry. 2019 Aug 21;9:199. doi: 10.1038/s41398-019-0527-1 (PMC6704174; doi:10.1038/s41398-019-0527-1)
Supplement: Supplementary file 11 — Table S3 List of circRNAs predicted from miRNAs. [file 41398_2019_527_MOESM11_ESM.pdf]

Predicted target genes for results

Column A: Seqname, the name the sequence.
Column B: GeneSymbol, the official gene symbol of the sequence.
Column C: Type, the type of the circRNA.
Column D: Diagrams, the diagrams illustrate key features for miRNA binding. See Ref [1,2] for details.
Column E: Total, the total number of the binding sites on the targets.
Column F: Context+, the sum of the context+ scores used in TargetScan after version 6.0. More negative is better. See Ref [3,4] for details.
Column G: Context, the sum of the context scores used in TargetScan before version 5.x. More negative is better. See Ref [1] for details.
Column H: Structure, the sum of the structure scores used in miRanda (Ref [5]). The higher the better.
Column I: Energy, the sum of the free energy predicted by miRanda (Ref [5]). More negative is better.
Column J: Branch Length, the sum of the branch length in the phylogenetic tree of different species. The higher means the binding site is more conserved (Ref [3]).
Column K: Pct, the probability of preferentially conserved targeting (Ref [3]). This score reflected the Bayesian estimate of the probability that a site is conserved due to selective maintenance of miRNA targeting rather than by chance or any other reason not pertinent to miRNA targeting.
Column L ~ Column W: Counting numbers for different seed match types.

Note:
Currently, there is not enough data for constructing phylogenetic tree of different species for LncRNAs. The "Conservation" section is meaningless for LncRNA. All sites on LncRNA are treated as non-conserved ones.

Reference:
[1] Grimson A., et al. MicroRNA Targeting Specificity in Mammals: Determinants beyond Seed Pairing. Molecular Cell, Volume 27, Issue 1, 91-105, 6 July 2007.
[2] Amy E. Pasquinelli. MicroRNAs and their targets: recognition, regulation and an emerging reciprocal relationship. Nature Reviews Genetics, Volume 13, 271-282, 1 April 2012.
[3] Robin C Friedman, et al. Most Mammalian mRNAs Are Conserved Targets of MicroRNAs. Genome Research, Volume 19, 92-105, 2009.
[4] David M Garcia., et al. Weak Seed-Pairing Stability and High Target-Site Abundance Decrease the Proficiency of lsy-6 and Other miRNAs. Nat. Struct. Mol. Biol., Volume 18, 1139-1146, 2011.
[5] Enright AJ., el al. miRanda algorithm: MicroRNA targets in Drosophila. Genome Biology, Volume 5, R1, 2003.

miRNA	Target			Sites		TargetScan		miRanda		Conservation		Conserved Sites						Poorly Conserved Sites						Annotations
miRNA	Seqname	GeneSymbol	Type	Diagrams	Total	Context+	Context	Structure	Energy	Branch Len	Pct	8mer	7mer-m8	7mer-A1	6mer	Offset 6mer	Imperfect	8mer	7mer-m8	7mer-A1	6mer	Offset 6mer	Imperfect	UTR Length
mmu-miR-138-5p	mm9_circ_002520	mm9_circ_002520	circRNA	Click Me	33			4884.0	-825.69	0.0		0.0	0.0	0.0	0.0	0.0	0.0	0.0	0.0	0.0	0.0	0.0	33.0	61406.0
mmu-miR-212-3p	mm9_circ_002520	mm9_circ_002520	circRNA	Click Me	29			4060.0	-595.66	0.0		0.0	0.0	0.0	0.0	0.0	0.0	0.0	0.0	0.0	0.0	0.0	29.0	61406.0
mmu-miR-138-5p	mm9_circ_018180	mm9_circ_018180	circRNA	Click Me	23	-1.129	-1.602	3455.0	-472.74	0.0	0.276	0.0	0.0	0.0	0.0	0.0	0.0	5.0	8.0	1.0	1.0	1.0	7.0	95434.0
mmu-miR-138-5p	mm9_circ_016497	mm9_circ_016497	circRNA	Click Me	20	-0.727	-1.234	3008.0	-415.15	0.0	0.251	0.0	0.0	0.0	0.0	0.0	0.0	4.0	7.0	1.0	1.0	0.0	7.0	94941.0
mmu-miR-134-5p	mm9_circ_018180	mm9_circ_018180	circRNA	Click Me	19	-0.287	-0.795	2888.0	-409.81	0.0		0.0	0.0	0.0	0.0	0.0	0.0	0.0	10.0	0.0	0.0	5.0	4.0	95434.0
mmu-miR-134-5p	mm9_circ_016497	mm9_circ_016497	circRNA	Click Me	19	-0.287	-0.795	2888.0	-409.81	0.0		0.0	0.0	0.0	0.0	0.0	0.0	0.0	10.0	0.0	0.0	5.0	4.0	94941.0
mmu-miR-138-5p	mm9_circ_008688	mm9_circ_008688	circRNA	Click Me	18	-0.745	-1.112	2702.0	-381.83	0.0	6.6e-2	0.0	0.0	0.0	0.0	0.0	0.0	6.0	2.0	0.0	0.0	0.0	10.0	62003.0
mmu-miR-138-5p	mmu_circRNA_000396	mmu_circRNA_000396	circRNA	Click Me	17	-1.156	-1.583	2512.0	-341.81	0.0	0.141	0.0	0.0	0.0	0.0	0.0	0.0	6.0	3.0	1.0	0.0	0.0	7.0	96703.0
mmu-let-7d-5p	mm9_circ_011562	mm9_circ_011562	circRNA	Click Me	15	-0.286	-0.759	2200.0	-284.87	0.0	0.0	0.0	0.0	0.0	0.0	0.0	0.0	4.0	1.0	1.0	0.0	0.0	9.0	94321.0
mmu-miR-134-5p	mmu_circRNA_002381	mmu_circRNA_002381	circRNA	Click Me	15	-0.253	-0.586	2194.0	-303.26	0.0		0.0	0.0	0.0	0.0	0.0	0.0	2.0	1.0	0.0	2.0	5.0	5.0	98008.0
mmu-miR-138-5p	mm9_circ_011599	mm9_circ_011599	circRNA	Click Me	15	-0.223	-0.408	2164.0	-297.83	0.0	6.6e-2	0.0	0.0	0.0	0.0	0.0	0.0	3.0	2.0	0.0	0.0	0.0	10.0	92702.0
mmu-miR-138-5p	mm9_circ_000802	mm9_circ_000802	circRNA	Click Me	14	-0.354	-0.618	2094.0	-298.54	0.0	0.185	0.0	0.0	0.0	0.0	0.0	0.0	1.0	6.0	0.0	0.0	0.0	7.0	93919.0
mmu-let-7d-5p	mm9_circ_013985	mm9_circ_013985	circRNA	Click Me	13	-0.276	-0.7	1917.0	-247.9	0.0	0.0	0.0	0.0	0.0	0.0	0.0	0.0	4.0	0.0	1.0	0.0	0.0	8.0	85570.0
mmu-miR-134-5p	mmu_circRNA_007853	mmu_circRNA_007853	circRNA	Click Me	13	-9.8e-2	-0.286	1901.0	-271.18	0.0		0.0	0.0	0.0	0.0	0.0	0.0	1.0	2.0	1.0	1.0	5.0	3.0	81902.0
mmu-miR-138-5p	mm9_circ_011562	mm9_circ_011562	circRNA	Click Me	13	-0.222	-0.447	1925.0	-276.07	0.0	0.185	0.0	0.0	0.0	0.0	0.0	0.0	0.0	6.0	0.0	0.0	1.0	6.0	94321.0
mmu-miR-138-5p	mm9_circ_013985	mm9_circ_013985	circRNA	Click Me	13	-0.222	-0.447	1925.0	-276.07	0.0	0.185	0.0	0.0	0.0	0.0	0.0	0.0	0.0	6.0	0.0	0.0	1.0	6.0	85570.0
mmu-miR-138-5p	mmu_circRNA_003834	mmu_circRNA_003834	circRNA	Click Me	13	-0.197	-0.311	1909.0	-281.87	0.0	3.4e-2	0.0	0.0	0.0	0.0	0.0	0.0	3.0	1.0	0.0	1.0	1.0	7.0	76465.0
mmu-let-7d-5p	mm9_circ_000802	mm9_circ_000802	circRNA	Click Me	12	-0.117	-0.299	1770.0	-232.94	0.0	0.0	0.0	0.0	0.0	0.0	0.0	0.0	0.0	2.0	0.0	0.0	0.0	10.0	93919.0
mmu-let-7d-5p	mmu_circRNA_011148	mmu_circRNA_011148	circRNA	Click Me	12	-7.8e-2	-0.205	1763.0	-238.97	0.0	0.0	0.0	0.0	0.0	0.0	0.0	0.0	0.0	2.0	0.0	0.0	0.0	10.0	90472.0
mmu-miR-134-5p	mmu_circRNA_013002	mmu_circRNA_013002	circRNA	Click Me	12	-5.9e-2	7.6e-2	1731.0	-270.37	0.0		0.0	0.0	0.0	0.0	0.0	0.0	1.0	1.0	0.0	0.0	6.0	4.0	64767.0
mmu-miR-138-5p	mm9_circ_004002	mm9_circ_004002	circRNA	Click Me	12	-0.178	-0.191	1754.0	-263.22	0.0	0.141	0.0	0.0	0.0	0.0	0.0	0.0	0.0	3.0	1.0	0.0	3.0	5.0	80594.0
mmu-let-7d-5p	mm9_circ_013657	mm9_circ_013657	circRNA	Click Me	11	-0.229	-0.566	1603.0	-221.93	0.0	0.0	0.0	0.0	0.0	0.0	0.0	0.0	2.0	1.0	0.0	0.0	0.0	8.0	55149.0
mmu-let-7d-5p	mmu_circRNA_003161	mmu_circRNA_003161	circRNA	Click Me	11	-0.161	-0.481	1642.0	-225.68	0.0	0.0	0.0	0.0	0.0	0.0	0.0	0.0	0.0	5.0	0.0	0.0	1.0	5.0	47676.0
mmu-let-7d-5p	mm9_circ_018180	mm9_circ_018180	circRNA	Click Me	11	-0.159	-0.41	1610.0	-213.14	0.0	0.0	0.0	0.0	0.0	0.0	0.0	0.0	1.0	2.0	0.0	0.0	0.0	8.0	95434.0
mmu-let-7d-5p	mm9_circ_016497	mm9_circ_016497	circRNA	Click Me	11	-0.159	-0.41	1610.0	-213.14	0.0	0.0	0.0	0.0	0.0	0.0	0.0	0.0	1.0	2.0	0.0	0.0	0.0	8.0	94941.0
mmu-let-7d-5p	mm9_circ_006425	mm9_circ_006425	circRNA	Click Me	11	-0.15	-0.492	1623.0	-211.84	0.0	0.0	0.0	0.0	0.0	0.0	0.0	0.0	0.0	5.0	0.0	0.0	0.0	6.0	53256.0
mmu-let-7d-5p	mm9_circ_008850	mm9_circ_008850	circRNA	Click Me	11	-0.13	-0.42	1623.0	-211.57	0.0	0.0	0.0	0.0	0.0	0.0	0.0	0.0	0.0	4.0	0.0	0.0	0.0	7.0	78864.0
mmu-let-7d-5p	mmu_circRNA_014343	mmu_circRNA_014343	circRNA	Click Me	11	-0.114	-0.319	1673.0	-234.27	0.0	0.0	0.0	0.0	0.0	0.0	0.0	0.0	2.0	2.0	0.0	0.0	0.0	7.0	38888.0
mmu-let-7d-5p	mm9_circ_013353	mm9_circ_013353	circRNA	Click Me	11	-5.6e-2	-0.195	1591.0	-218.76	0.0	0.0	0.0	0.0	0.0	0.0	0.0	0.0	1.0	1.0	0.0	0.0	0.0	9.0	80654.0
mmu-let-7d-5p	mm9_circ_004002	mm9_circ_004002	circRNA	Click Me	11	-5.1e-2	-0.147	1586.0	-217.54	0.0	0.0	0.0	0.0	0.0	0.0	0.0	0.0	1.0	0.0	0.0	0.0	1.0	9.0	80594.0
mmu-miR-134-5p	mm9_circ_016337	mm9_circ_016337	circRNA	Click Me	11	-0.211	-0.503	1625.0	-249.71	0.0		0.0	0.0	0.0	0.0	0.0	0.0	0.0	3.0	2.0	1.0	2.0	3.0	94872.0
mmu-miR-138-5p	mm9_circ_008850	mm9_circ_008850	circRNA	Click Me	11	-0.225	-0.371	1602.0	-230.65	0.0	0.141	0.0	0.0	0.0	0.0	0.0	0.0	1.0	3.0	1.0	0.0	0.0	6.0	78864.0
mmu-miR-138-5p	mm9_circ_012873	mm9_circ_012873	circRNA	Click Me	11	-0.187	-0.177	1603.0	-240.53	0.0	0.111	0.0	0.0	0.0	0.0	0.0	0.0	0.0	2.0	1.0	0.0	3.0	5.0	80605.0
mmu-let-7d-5p	mmu_circRNA_002381	mmu_circRNA_002381	circRNA	Click Me	10	-0.226	-0.614	1485.0	-167.89	0.0	0.0	0.0	0.0	0.0	0.0	0.0	0.0	2.0	4.0	0.0	0.0	0.0	4.0	98008.0
mmu-let-7d-5p	mmu_circRNA_000396	mmu_circRNA_000396	circRNA	Click Me	10	-0.212	-0.581	1508.0	-189.38	0.0	0.0	0.0	0.0	0.0	0.0	0.0	0.0	0.0	5.0	0.0	0.0	1.0	4.0	96703.0
mmu-let-7d-5p	mmu_circRNA_006194	mmu_circRNA_006194	circRNA	Click Me	10	-7.9e-2	-0.207	1442.0	-183.25	0.0	0.0	0.0	0.0	0.0	0.0	0.0	0.0	0.0	2.0	0.0	0.0	0.0	8.0	48795.0
mmu-let-7d-5p	mm9_circ_012873	mm9_circ_012873	circRNA	Click Me	10	-4.7e-2	-0.17	1445.0	-201.42	0.0	0.0	0.0	0.0	0.0	0.0	0.0	0.0	1.0	0.0	0.0	0.0	0.0	9.0	80605.0
mmu-let-7d-5p	mm9_circ_010282	mm9_circ_010282	circRNA	Click Me	10	-4.7e-2	-0.17	1445.0	-201.42	0.0	0.0	0.0	0.0	0.0	0.0	0.0	0.0	1.0	0.0	0.0	0.0	0.0	9.0	80414.0
mmu-let-7d-5p	mmu_circRNA_018737	mmu_circRNA_018737	circRNA	Click Me	10	-2.8e-2	-9.3e-2	1429.0	-185.64	0.0	0.0	0.0	0.0	0.0	0.0	0.0	0.0	0.0	1.0	0.0	0.0	0.0	9.0	86040.0
mmu-miR-134-5p	mm9_circ_008688	mm9_circ_008688	circRNA	Click Me	10	-0.152	-0.558	1490.0	-206.48	0.0		0.0	0.0	0.0	0.0	0.0	0.0	2.0	2.0	2.0	2.0	2.0	0.0	62003.0
mmu-miR-138-5p	mmu_circRNA_013002	mmu_circRNA_013002	circRNA	Click Me	10	-0.639	-0.796	1528.0	-224.27	0.0	0.17	0.0	0.0	0.0	0.0	0.0	0.0	2.0	4.0	1.0	0.0	1.0	2.0	64767.0
mmu-miR-138-5p	mm9_circ_017914	mm9_circ_017914	circRNA	Click Me	10	-0.316	-0.506	1510.0	-207.55	0.0	0.127	0.0	0.0	0.0	0.0	0.0	0.0	2.0	4.0	0.0	0.0	0.0	4.0	83355.0
mmu-miR-138-5p	mm9_circ_013353	mm9_circ_013353	circRNA	Click Me	10	-0.131	-0.191	1457.0	-215.41	0.0	0.111	0.0	0.0	0.0	0.0	0.0	0.0	0.0	2.0	1.0	0.0	2.0	5.0	80654.0
mmu-miR-138-5p	mm9_circ_010282	mm9_circ_010282	circRNA	Click Me	10	-0.131	-0.191	1457.0	-215.41	0.0	0.111	0.0	0.0	0.0	0.0	0.0	0.0	0.0	2.0	1.0	0.0	2.0	5.0	80414.0
mmu-miR-138-5p	mmu_circRNA_002381	mmu_circRNA_002381	circRNA	Click Me	10	-0.109	-0.146	1441.0	-208.02	0.0	0.125	0.0	0.0	0.0	0.0	0.0	0.0	0.0	1.0	2.0	0.0	0.0	7.0	98008.0
