# Supplementary material for: Cocaine induces differential circular RNA expression in striatum
Source: Transl Psychiatry. 2019 Aug 21;9:199. doi: 10.1038/s41398-019-0527-1 (PMC6704174; doi:10.1038/s41398-019-0527-1)
Supplement: Supplementary file 4 — Figure S3 The annotations for circRNA/miRNA interaction are displayed in CircRNA-MREs. [file 41398_2019_527_MOESM4_ESM.pdf]

| 2D Structure | Local AU | Position | Conservation | Predicted By |
|---|---|---|---|---|
| Offset 6mer<br>45 68<br>5'-ctGGCGGGATGGGCTTATACCTga-3' UTR<br>: : \| : \|  \| : : : : \| \|  \| \| \| \| \| \|<br>3'-uaUUGUCGUGUUUGAUUAUGGAgu-5' miRNA<br>16 15 14 13    7 6 5 4 3 2<br>3'pairing Seed | 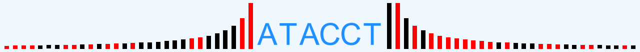 ATACCT<br>Offset 6mer | 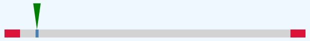 | | M |
| Imperfect match<br>354 377<br>5'-agAACTGCAGGAGTCAGTACTTCa-3' UTR<br>\| \| \|  \| \| \|  : \| : :  \| : \| \| \| : \| \|<br>3'-uaUUGUCGUGUUUGAUUAUGGAGu-5' miRNA<br>16 15 14 13    7 6 5 4 3 2<br>3'pairing Seed | 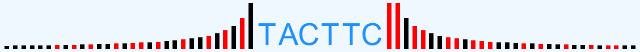 TACTTC<br>Imperfect | 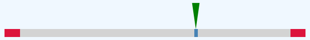 | | M |

| 2D Structure | Local AU | Position | Conservation | Predicted By |
|---|---|---|---|---|
| 283 7mer-m8 307<br>5'-aaataCAGACTTGAACGACTTGTTc-3' UTR<br>|||||| | ||||||||<br>3'-acaccGUCUGACC---CUGAACAAc-5' miRNA<br>16 15 14 13 7 6 5 4 3 2<br>3'pairing Seed | 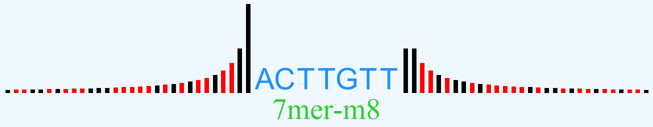 | 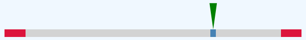 | | M T |

| 2D Structure | Local AU | Position | Conservation | Predicted By |
|---|---|---|---|---|
| See alignment 1 below | 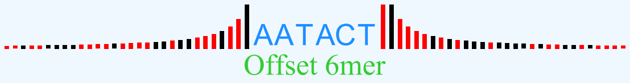 | 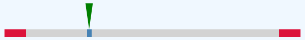 | | M |
| See alignment 2 below | 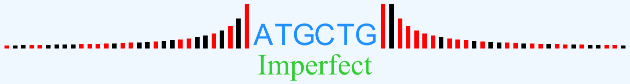 | 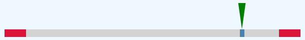 | | M |





Alignment 1:

```
                      Offset
                       6mer
102                           124
   5'-tatGCAGCATTCTTGAATACTTc-3'  UTR
         ||||||:   |: ||||||
   3'-aguCGUCGUGUCAG-UUAUGAcc-5'  miRNA
            16 15 14 13   7 6 5 4 3 2
          3'pairing        Seed
```

Alignment 2:

```
                      Imperfect
                        match
315                              338
   5'-gaAGCAGGATCCACAG--AATGCTGa -3'  UTR
        |||||     |||||  |||:|||
   3'-agUCGUC----GUGUCAGUUAUGACc-5'  miRNA
           16    15 14 13   7 6 5 4 3 2
          3'pairing           Seed
```

| 2D Structure | Local AU | Position | Conservation | Predicted By |
|---|---|---|---|---|
| 201 7mer-m8 223<br>5'-tcaCAAAACTTACTCTGCTGCTg-3' UTR<br>3'-gcgGUUAUAAAUG-CACGACGAu-5' miRNA<br>16 15 14 13 7 6 5 4 3 2<br>3'pairing Seed | 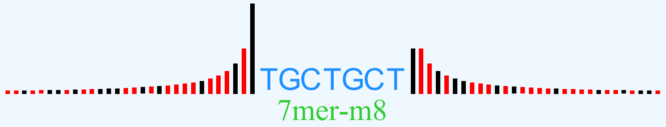 | 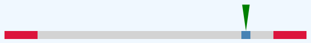 | ☐ | M T |

| 2D Structure | Local AU | Position | Conservation | Predicted By |
|---|---|---|---|---|
| 343 7mer-m8 364<br>5'-gccgtgtctggagaACTGCAGg-3' UTR<br>|||||||<br>3'-gauguucacgggagUGACGUCa-5' miRNA<br>16 15 14 13 7 6 5 4 3 2<br>3'pairing Seed | 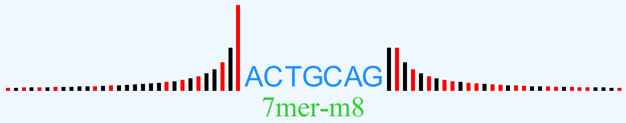 | 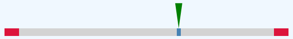 | | M T |
| 394 7mer-A1 416<br>5'-cacCAGGAGATCCTTGCTGCAGa-3' UTR<br>||:| | :||||::||||||<br>3'-gauGUUCAC-GGGAGUGACGUCa-5' miRNA<br>16 15 14 13 7 6 5 4 3 2<br>3'pairing Seed | 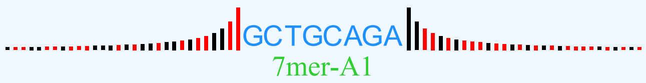 | 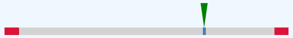 | | M T |
| 522 7mer-m8 550<br>5'-ccACGAGCGGCTTCCCCAAGGACTGCAGt-3' UTR<br>||:|| | |:|| |||||||<br>3'-gaUGUUCACGGGAG-------UGACGUCa-5' miRNA<br>16 15 14 13 7 6 5 4 3 2<br>3'pairing Seed | 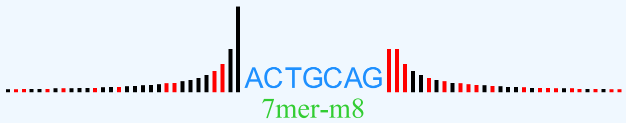 | 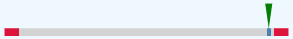 | | M T |

| 2D Structure | Local AU | Position | Conservation | Predicted By |
|---|---|---|---|---|
| 270 7mer-m8 291<br>5'-gaaacttgGTTTTCACTGCAGc-3' UTR<br>\|:::\|\|\|\|\|\|\|\|\|\|<br>3'-gauguucaCGGGAGUGACGUCa-5' miRNA<br>16 15 14 13 7 6 5 4 3 2<br>3'pairing Seed | 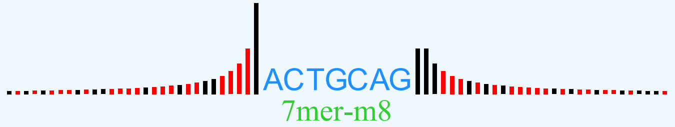 | 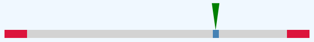 | | M T |

| 2D Structure | Local AU | Position | Conservation | Predicted By |
| --- | --- | --- | --- | --- |
| 270 7mer-m8 291<br>5'-gaaacttgGTTTTCACTGCAGc-3' UTR<br>3'-gauguucaCGGGAGUGACGUCa-5' miRNA<br>16 15 14 13 7 6 5 4 3 2<br>3'pairing Seed | 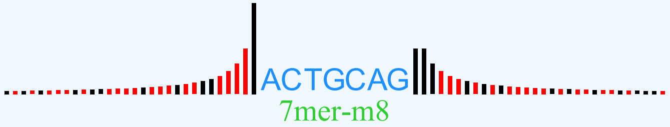 | 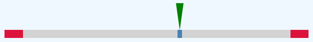 | | M T |

| 2D Structure | Local AU | Position | Conservation | Predicted By |
|---|---|---|---|---|
| 343 7mer-m8 364<br>5'-gccgtgtctggagaACTGCAGg-3' UTR<br>\|\|\|\|\|\|\|<br>3'-gauguucacgggagUGACGUCa-5' miRNA<br>16 15 14 13 7 6 5 4 3 2<br>3'pairing Seed | ACTGCAG<br>7mer-m8 | | | M T |
| 394 7mer-A1 416<br>5'-cacCAGGAGATCCTTGCTGCAGa-3' UTR<br>\|\|:\| \| :\|\|\|::\|\|\|\|\|\|<br>3'-gauGUUCAC-GGGAGUGACGUCa-5' miRNA<br>16 15 14 13 7 6 5 4 3 2<br>3'pairing Seed | GCTGCAGA<br>7mer-A1 | | | M T |
| 522 7mer-m8 550<br>5'-ccACGAGCGGCTTCCCCAAGGACTGCAGt-3' UTR<br>\|\|:\|\| \| \|:\|\| \|\|\|\|\|\|<br>3'-gaUGUUCACGGGAG-------UGACGUCa-5' miRNA<br>16 15 14 13 7 6 5 4 3 2<br>3'pairing Seed | ACTGCAG<br>7mer-m8 | | | M T |

| 2D Structure | Local AU | Position | Conservation | Predicted By |
|---|---|---|---|---|
| Imperfect match<br>84 105<br>5'-gcaaacaCCCTG-AAGTACTTTTt-3' UTR<br>3'-gauggacGUGACAUUCGUGAAAc-5' miRNA<br>16 15 14 13 7 6 5 4 3 2<br>3'pairing Seed | TACTTTT<br>Imperfect | | | M |
| Imperfect match<br>105 128<br>5'-tctcagaCCTTGTTAAGCATTTTg-3' UTR<br>3'-gauggacGUGAC-AUUCGUGAAAc-5' miRNA<br>16 15 14 13 7 6 5 4 3 2<br>3'pairing Seed | CATTTT<br>Imperfect | | | M |
| 7mer-m8<br>477 500<br>5'-aatgaTGATGTTCTGAGCACTTTg-3' UTR<br>3'-gauggAC-GUGACAUUCGUGAAAc-5' miRNA<br>16 15 14 13 7 6 5 4 3 2<br>3'pairing Seed | GCACTTT<br>7mer-m8 | | | M T |

| 2D Structure | Local AU | Position | Conservation | Predicted By |
| --- | --- | --- | --- | --- |
| Offset 6mer<br>23 45<br>5'-tacaaATCTTAAATTGCAAAATc-3' UTR<br>\|\| : \|\| :\|\|\|\|\|\|:<br>3'-cgaccUACGUUUGGACGUUUUGa-5' miRNA<br>16 15 14 13 7 6 5 4 3 2<br>3'pairing Seed | 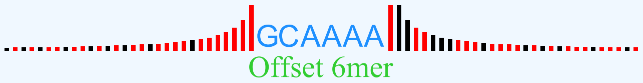 | 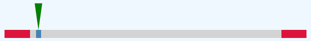 | | M |
| 7mer-m8<br>252 274<br>5'-gcTGTGTGGAAAGAAGCAAAACc-3' UTR<br>\|\| :\|\| \|\|\| \|\|\|\|\|\|<br>3'-cgACCUACGUUUGGACGUUUUGa-5' miRNA<br>16 15 14 13 7 6 5 4 3 2<br>3'pairing Seed | 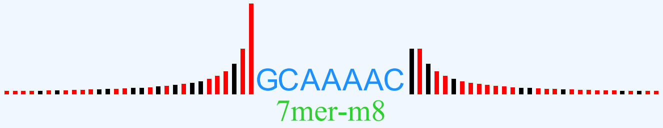 | 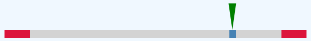 | | M T |

| 2D Structure | Local AU | Position | Conservation | Predicted By |
|---|---|---|---|---|
| 541 8mer 565<br>5'-tcgcAGCTGCTGAGACTGCAAAACa-3' UTR<br>\|:\|\|\|\| :\|: \|\|\|\|\|\|\|\|\|<br>3'-cgacUUGACG-UUUAGACGUUUUGa-5' miRNA<br>16 15 14 13 7 6 5 4 3 2<br>3'pairing Seed | 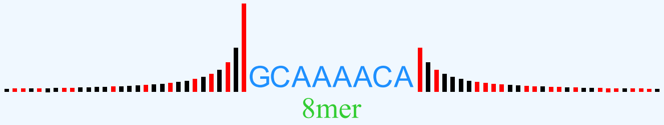 | 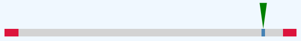 | | M T |
| Imperfect match<br>569 593<br>5'-ggccAAATGCAACATCTGCAAAGaa -3' UTR<br>\|\| \|\|\|\|\| \|\|\|\|\|\|\|\|\|:<br>3'-cgacUUGACGUU-UAGACGUUUUga-5' miRNA<br>16 15 14 13 7 6 5 4 3 2<br>3'pairing Seed | 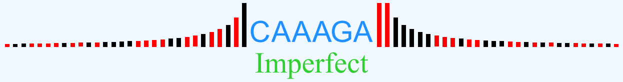 | 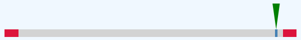 | | M |

<table>
<tr><th>2D Structure</th><th>Local AU</th><th>Position</th><th>Conservation</th><th>Predicted By</th></tr>
<tr><td>8mer<br>541 565<br>5'-tcgcAGCTGCTGAGACTGCAAAACa-3' UTR<br>|:|||| :|: |||||||||<br>3'-cgacUUGACG-UUUAGACGUUUUGa-5' miRNA<br>16 15 14 13 7 6 5 4 3 2<br>3'pairing Seed</td><td>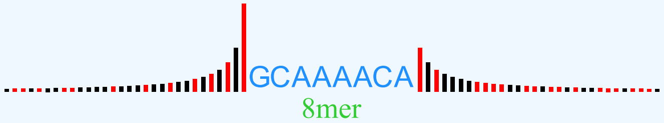
</td><td>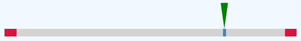</td><td>☐</td><td>M T</td></tr>
<tr><td>Imperfect match<br>569 593<br>5'-ggccAAATGCAACATCTGCAAAGaa -3' UTR<br>|| ||||| |||||||||:<br>3'-cgacUUGACGUU-UAGACGUUUUga-5' miRNA<br>16 15 14 13 7 6 5 4 3 2<br>3'pairing Seed</td><td>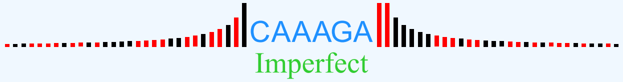
</td><td>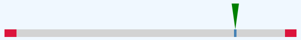</td><td>☐</td><td>M</td></tr>
</table>

| 2D Structure | Local AU | Position | Conservation | Predicted By |
|---|---|---|---|---|
| Offset 6mer<br>23 45<br>5'-tacaAATCTTAAAT-TGCAAAATc-3' UTR<br>3'-cgacUUGACGUUUAGACGUUUUGa-5' miRNA<br>16 15 14 13 7 6 5 4 3 2<br>3'pairing Seed | 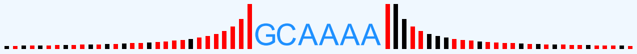 | 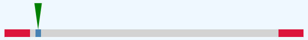 | | M |
| 7mer-m8<br>251 274<br>5'-agctgtgTGGAAAGAAGCAAAACc-3' UTR<br>3'-cgacuugACGUUUAGACGUUUUGa-5' miRNA<br>16 15 14 13 7 6 5 4 3 2<br>3'pairing Seed | 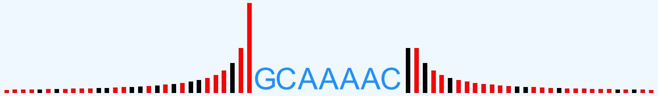 | 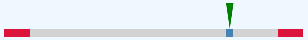 | | M T |

| 2D Structure | Local AU | Position | Conservation | Predicted By |
|---|---|---|---|---|
| 343 7mer-m8 364<br>5'-gccgtgtctggagaACTGCAGg-3' UTR<br>\|\|\|\|\|\|\|<br>3'-gaucuucacgagugUGACGUCa-5' miRNA<br>16 15 14 13 7 6 5 4 3 2<br>3'pairing Seed | 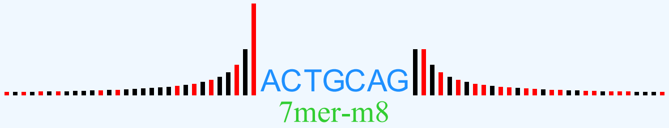 | 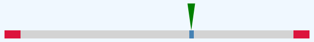 | ☐ | M T |
| 529 7mer-m8 550<br>5'-cggcttccccaaggACTGCAGt-3' UTR<br>\|\|\|\|\|\|\|<br>3'-gaucuucacgagugUGACGUCa-5' miRNA<br>16 15 14 13 7 6 5 4 3 2<br>3'pairing Seed | 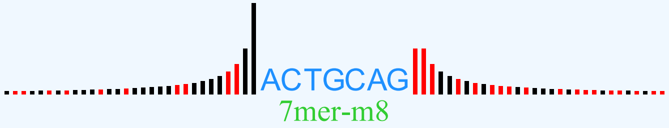 | 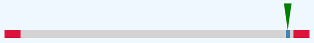 | ☐ | M T |

| 2D Structure | Local AU | Position | Conservation | Predicted By |
|---|---|---|---|---|
| 118 7mer-m8 139<br>5'-ttctgaggtggtggTGCTGTTc-3' UTR<br>\|\|\|\|\|\|\|<br>3'-cugucgguagcugACGACAAc-5' miRNA<br>16 15 14 13 7 6 5 4 3 2<br>3'pairing Seed | 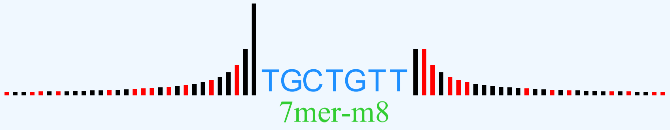 | 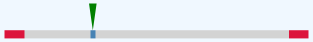 | ☐ | M T |
| 335 7mer-m8 356<br>5'-aaaGGCCCCATGAGTGCTGTTt-3' UTR<br>:\|\|\|\| :\|\| \|\|\|\|\|\|\|<br>3'-cugUCGGGUAGCUGACGACAAc-5' miRNA<br>16 15 14 13 7 6 5 4 3 2<br>3'pairing Seed | 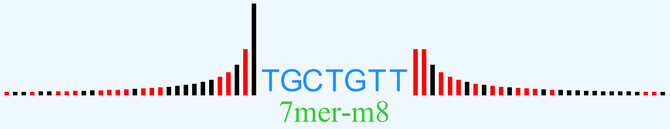 | 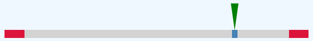 | ☐ | M T |

| 2D Structure | Local AU | Position | Conservation | Predicted By |
|---|---|---|---|---|
| Imperfect match<br>17 39<br>5'-agAAACATCAGGGAGATAAATTc -3' UTR<br>\|\|\| \|\|\|\| \|\|\|\|\|\|:\|<br>3'-ccUUUAUAGUCAGACUAUUUGAu-5' miRNA<br>16 15 14 13 7 6 5 4 3 2<br>3'pairing Seed | 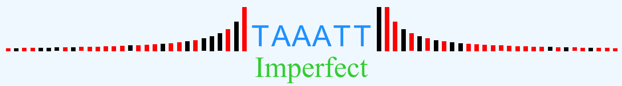 | 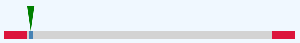 | | M |
| 8mer<br>254 279<br>5'-ttAATTGTCAGTCTCTACATAAACTa-3' UTR<br>\|\| \|:\|\|\|\|\|\|\| \|\|\|\|\|\|\|<br>3'-ccUUUAUAGUCAGA---CUAUUUGAu-5' miRNA<br>16 15 14 13 7 6 5 4 3 2<br>3'pairing Seed | 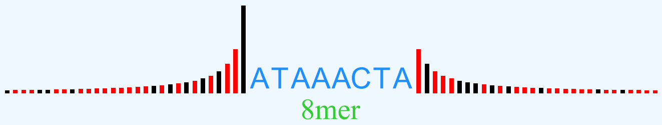 | 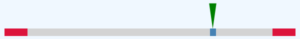 | | M T |

| 2D Structure | Local AU | Position | Conservation | Predicted By |
|---|---|---|---|---|
| Imperfect match<br>167 188<br>5'-tatGTGTCTCAACTGGCTGCTc -3' UTR<br>3'-uguCAAGAAGUUGACCGUCGAa-5' miRNA<br>16 15 14 13 7 6 5 4 3 2<br>3'pairing Seed | 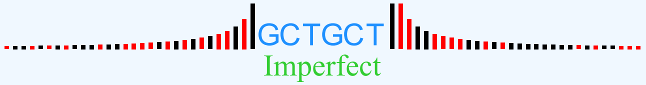 | 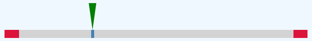 | ☐ | M |
| 7mer-m8<br>321 341<br>5'-atgtgtgacCAGC-GGCAGCTt-3' UTR<br>3'-ugucaagaaGUUGACCGUCGAa-5' miRNA<br>16 15 14 13 7 6 5 4 3 2<br>3'pairing Seed | 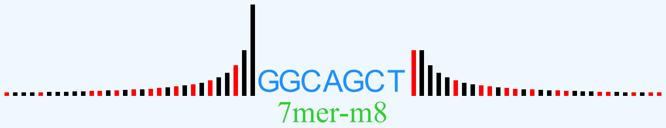 | 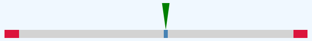 | ☐ | M T |
| 7mer-m8<br>362 383<br>5'-catccagaTCCCTAGGCAGCTg-3' UTR<br>3'-ugucaagaAGUUGACCGUCGAa-5' miRNA<br>16 15 14 13 7 6 5 4 3 2<br>3'pairing Seed | 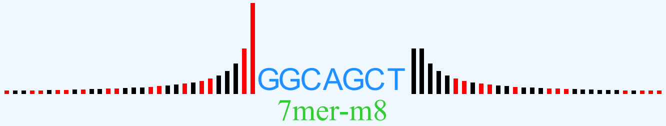 | 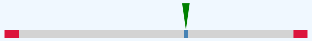 | ☐ | M T |

| 2D Structure | Local AU | Position | Conservation | Predicted By |
|---|---|---|---|---|
| Imperfect match<br>167 188<br>5'-tatGTGTCTCAACTGGCTGCTc -3' UTR<br>3'-uguCAAGAAGUUGACCGUCGAa-5' miRNA<br>16 15 14 13 7 6 5 4 3 2<br>3'pairing Seed | GCTGCT<br>Imperfect | | | M |
| 7mer-m8<br>321 341<br>5'-atgtgtgacCAGC-GGCAGCTt-3' UTR<br>3'-ugucaagaaGUUGACCGUCGAa-5' miRNA<br>16 15 14 13 7 6 5 4 3 2<br>3'pairing Seed | GGCAGCT<br>7mer-m8 | | | M T |
| 7mer-m8<br>362 383<br>5'-catccagaTCCCTAGGCAGCTg-3' UTR<br>3'-ugucaagaAGUUGACCGUCGAa-5' miRNA<br>16 15 14 13 7 6 5 4 3 2<br>3'pairing Seed | GGCAGCT<br>7mer-m8 | | | M T |

<table>
<tr><th>2D Structure</th><th>Local AU</th><th>Position</th><th>Conservation</th><th>Predicted By</th></tr>
<tr><td>45 8mer 67<br>5'-aaGCCACACCTTTTCAGAACAGAa-3' UTR<br>|||| | | |||||||<br>3'-cuCGGU-UCAUUACCUCUUGUCc-5' miRNA<br>16 15 14 13 7 6 5 4 3 2<br>3'pairing Seed</td><td>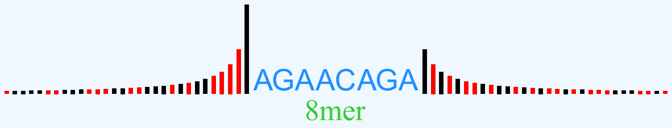<br></td><td>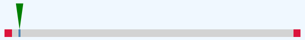</td><td></td><td>M T</td></tr>
<tr><td>765 Offset 6mer 786<br>5'-agatgcAGCAAAGGAGAACAcc-3' UTR<br>|| || |||||||||<br>3'-cucgguUCAUUACCUCUUGUCc-5' miRNA<br>16 15 14 13 7 6 5 4 3 2<br>3'pairing Seed</td><td>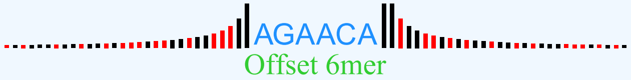<br></td><td>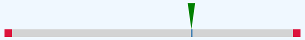</td><td></td><td>M</td></tr>
<tr><td>1016 7mer-m8 1039<br>5'-tctCTGACAACATGAGAGAACAGc-3' UTR<br>|::| | ||| ||||||||<br>3'-cucGGUUCAU-UAC-CUCUUGUCc-5' miRNA<br>16 15 14 13 7 6 5 4 3 2<br>3'pairing Seed</td><td>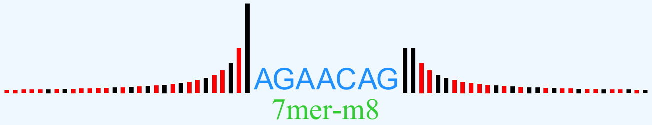<br></td><td>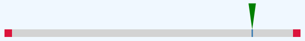</td><td></td><td>M T</td></tr>
</table>

| 2D Structure | Local AU | Position | Conservation | Predicted By |
|---|---|---|---|---|
| 390 7mer-m8 410<br>5'-tccagatggtGATACTGTGAc-3' UTR<br>3'-cgccuugaauCGGUGACACUu-5' miRNA<br>16 15 14 13 7 6 5 4 3 2<br>3'pairing Seed | 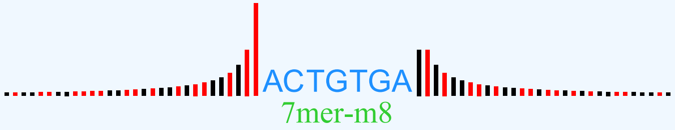 | 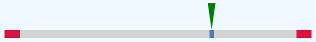 | | M T |
| 436 7mer-m8 456<br>5'-cctGGAGTCAGTGACTGTGAc-3' UTR<br>3'-cgcCUUGAAUCGGUGACACUu-5' miRNA<br>16 15 14 13 7 6 5 4 3 2<br>3'pairing Seed | 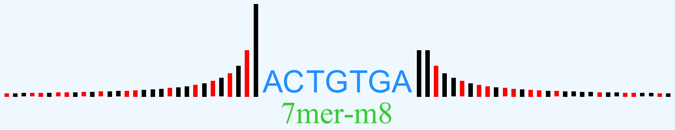 | 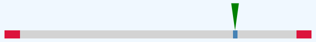 | | M T |

| 2D Structure | Local AU | Position | Conservation | Predicted By |
|---|---|---|---|---|
| 89 7mer-m8 108<br>5'-agcGGCCTTA-TAACTGTGAg-3' UTR<br>3'-cgcCUUGAAUCGGUGACACUu-5' miRNA<br>16 15 14 13 7 6 5 4 3 2<br>3'pairing Seed | ACTGTGA<br>7mer-m8 | | ☐ | M T |
| 349 7mer-m8 369<br>5'-tttaAGTGTCACCACTGTGAt-3' UTR<br>3'-cgccUUGAAUCGGUGACACUu-5' miRNA<br>16 15 14 13 7 6 5 4 3 2<br>3'pairing Seed | ACTGTGA<br>7mer-m8 | | ☐ | M T |
| 604 8mer 624<br>5'-tatcaatgcccgTACTGTGAa-3' UTR<br>3'-cgccuugaaucgGUGACACUu-5' miRNA<br>16 15 14 13 7 6 5 4 3 2<br>3'pairing Seed | ACTGTGAA<br>8mer | | ☐ | M T |

| 2D Structure | Local AU | Position | Conservation | Predicted By |
| --- | --- | --- | --- | --- |
| 391 7mer-m8 410<br>5'-ccAGATGGT-GATACTGTGAc-3' UTR<br>\|\|\| \| \| :\|\|\|\|\|\|\|<br>3'-cgUCUUGAAUCGGUGACACUu-5' miRNA<br>16 15 14 13 7 6 5 4 3 2<br>3'pairing Seed | 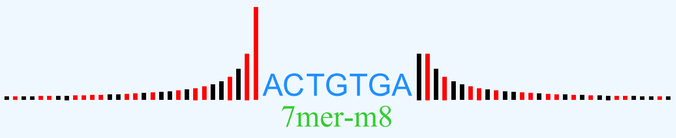 | 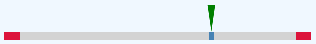 | | M T |
| 436 7mer-m8 456<br>5'-cctGGAGTCAGTGACTGTGAc-3' UTR<br>\|:\| \| \|\|: \|\|\|\|\|\|\|<br>3'-cguCUUGAAUCGGUGACACUu-5' miRNA<br>16 15 14 13 7 6 5 4 3 2<br>3'pairing Seed | 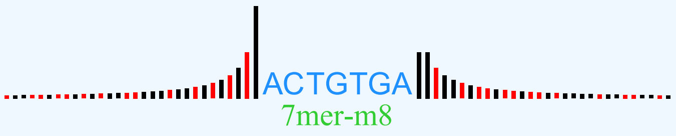 | 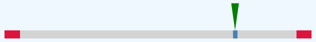 | | M T |

| 2D Structure | Local AU | Position | Conservation | Predicted By |
|---|---|---|---|---|
| 89 7mer-m8 108<br>5'-agcGGCCTTA-TAACTGTGAg-3' UTR<br>\|: \|\|\|\| : \|\|\|\|\|\|\|<br>3'-cguCUUGAAUCGGUGACACUu-5' miRNA<br>16 15 14 13 7 6 5 4 3 2<br>3'pairing Seed | ACTGTGA<br>7mer-m8 | | ☐ | M T |
| 349 7mer-m8 369<br>5'-tttaAGTGTCACCACTGTGAt-3' UTR<br>\|:: \| \|\|\|\|\|\|\|\|\|<br>3'-cgucUUGAAUCGGUGACACUu-5' miRNA<br>16 15 14 13 7 6 5 4 3 2<br>3'pairing Seed | ACTGTGA<br>7mer-m8 | | ☐ | M T |
| 604 8mer 624<br>5'-tatcaatgcccgTACTGTGAa-3' UTR<br>:\|\|\|\|\|\|\|<br>3'-cgucuugaaucgGUGACACUu-5' miRNA<br>16 15 14 13 7 6 5 4 3 2<br>3'pairing Seed | ACTGTGAA<br>8mer | | ☐ | M T |

| 2D Structure | Local AU | Position | Conservation | Predicted By |
|---|---|---|---|---|
| 133 7mer-m8 154<br>5'-ggTTTTGTTTATCA---GAAACCAg-3' UTR<br>\|:\| : ::\|:\|\| \|\|\|\|\|\|\|<br>3'-uuAGAUUCGGUGGUACACUUUGGUc-5' miRNA<br>16 15 14 13 7 6 5 4 3 2<br>3'pairing Seed | 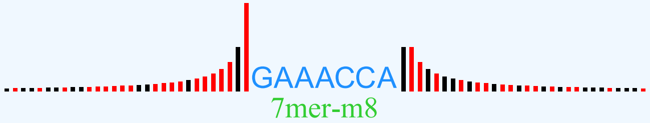 | 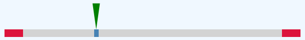 | ☐ | M T |
| Imperfect match<br>238 261<br>5'-ctaCTAA-AAACGGTGTGGAACCAa -3' UTR<br>\|\|\|\| \|\| :\|\|\|\|:\|\|\|\|\|<br>3'-uuaGAUUCGGUGGUACACUUUGGUc-5' miRNA<br>16 15 14 13 7 6 5 4 3 2<br>3'pairing Seed | 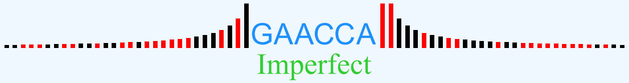 | 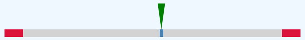 | ☐ | M |

<table>
<tr><th>2D Structure</th><th>Local AU</th><th>Position</th><th>Conservation</th><th>Predicted By</th></tr>
<tr><td>Imperfect match<br>587 608<br>5'-acAGTGACTCACATTCTCCTAa-3' UTR<br>|||:| : || :|||||:|<br>3'-ccUCAUUUGUUGGGAGAGGGUc-5' miRNA<br>16 15 14 13 7 6 5 4 3 2<br>3'pairing Seed</td><td>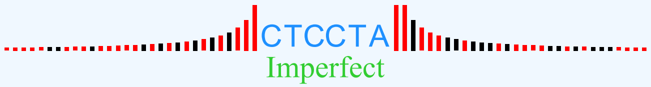</td><td>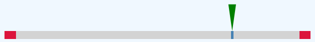</td><td></td><td>M</td></tr>
<tr><td>Imperfect match<br>672 693<br>5'-agAGAAAACTAGCTTCTCTCAg-3' UTR<br>|| |||| | |:||||:||<br>3'-ccUCAUUUGUUGGGAGAGGGUc-5' miRNA<br>16 15 14 13 7 6 5 4 3 2<br>3'pairing Seed</td><td>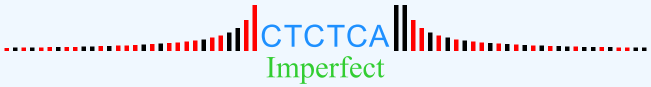</td><td>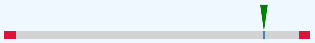</td><td></td><td>M</td></tr>
</table>

| 2D Structure | Local AU | Position | Conservation | Predicted By |
|---|---|---|---|---|
| Imperfect match<br>587 608<br>5'-acAGTGACTCACATTCTCCTAa-3' UTR<br>\|\|\|:\| : \| \|\|\|\|\|\|:\|<br>3'-ucUCAUUUGUCGGAAGAGGGUc-5' miRNA<br>16 15 14 13 7 6 5 4 3 2<br>3'pairing Seed | 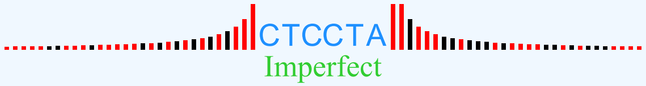 | 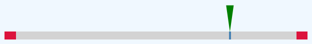 | | M |
| Imperfect match<br>672 693<br>5'-agAGAAAACTAG-CTTCTCTCAg-3' UTR<br>\|\| \|\|\|\| \|\| \|\|\|\|\|\|:\|\|<br>3'-ucUCAUUUG-UCGGAAGAGGGUc-5' miRNA<br>16 15 14 13 7 6 5 4 3 2<br>3'pairing Seed | 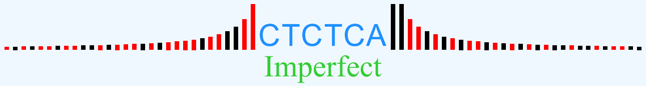 | 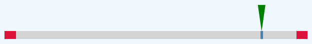 | | M |

| 2D Structure | Local AU | Position | Conservation | Predicted By |
|---|---|---|---|---|
| Imperfect match<br>497 518<br>5'-aaAACCAAGATTGCACTGGAAc-3' UTR<br>| | || ||: ||||:||<br>3'-cgUCGUUUGUAGACUGACUUUc-5' miRNA<br>16 15 14 13 7 6 5 4 3 2<br>3'pairing Seed | 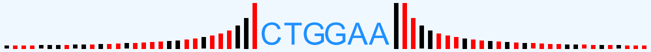 CTGGAA Imperfect | 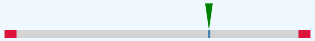 | ☐ | M |
| Imperfect match<br>573 594<br>5'-cttGCGAGGAGTTGATTGAAAa-3' UTR<br>||:|: | :|||:|||||<br>3'-cguCGUUUGUAGACUGACUUUc-5' miRNA<br>16 15 14 13 7 6 5 4 3 2<br>3'pairing Seed | 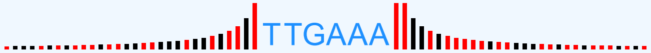 TTGAAA Imperfect | 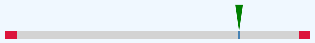 | ☐ | M |

| 2D Structure | Local AU | Position | Conservation | Predicted By |
|---|---|---|---|---|
| Imperfect match<br>497 … 518<br>5'-aaAACCAAGATTGCACTGGAAc -3' UTR<br>| | || ||: ||||:||<br>3'-cgUCGUUUGUAGACUGACUUUc-5' miRNA<br>16 15 14 13 · 7 6 5 4 3 2<br>3'pairing · Seed | CTGGAA<br>Imperfect | | | M |
| Imperfect match<br>573 … 594<br>5'-cttGCGAGGAGTTGATTGAAAa -3' UTR<br>||:|: | :|||:|||||<br>3'-cguCGUUUGUAGACUGACUUUc-5' miRNA<br>16 15 14 13 · 7 6 5 4 3 2<br>3'pairing · Seed | TTGAAA<br>Imperfect | | | M |

<table>
<tr><th>2D Structure</th><th>Local AU</th><th>Position</th><th>Conservation</th><th>Predicted By</th></tr>
<tr><td>Imperfect<br>match<br>124 143<br>5'-acaGTCAC-TACTCTAGATTa -3' UTR<br>:|||| :|| |||:|||<br>3'-uuaUAGUGUGUGUGAUUUAAc-5' miRNA<br>16 15 14 13 7 6 5 4 3 2<br>3'pairing Seed</td><td>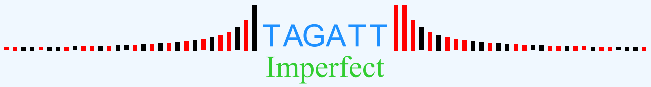
</td><td>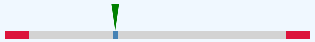</td><td></td><td>M</td></tr>
<tr><td>Imperfect<br>match<br>263 284<br>5'-agTCTCTACATAAACTAAGTTt -3' UTR<br>| || |||:| |||||:||<br>3'-uuAUAG-UGUGUGUGAUUUAAc-5' miRNA<br>16 15 14 13 7 6 5 4 3 2<br>3'pairing Seed</td><td>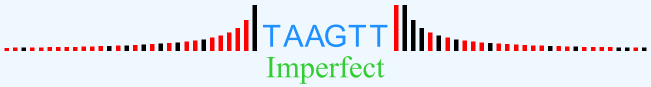
</td><td>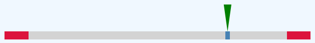</td><td></td><td>M</td></tr>
</table>

| 2D Structure | Local AU | Position | Conservation | Predicted By |
| --- | --- | --- | --- | --- |
| 23 7mer-m8 47<br>5'-tcAGTGGCTGGCAGGATCCCCACCt-3' UTR<br>\|:\| \|\|:: \|\| \|\|\|\|\|\|\|\|\|<br>3'-caUUA-CGGUGGU--UAGGGGUGGa-5' miRNA<br>16 15 14 13 7 6 5 4 3 2<br>3'pairing Seed | 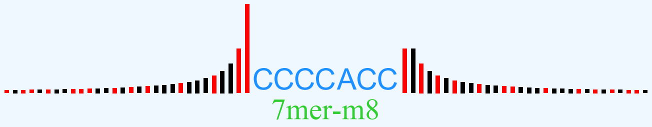 | 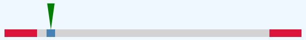 | ☐ | M T |
| Imperfect match<br>36 59<br>5'-ggatccCCACCTCACTCTCCACCt -3' UTR<br>\|\|\|\| \|\| \|\|:\|\|\|\|\|<br>3'-cauuacGGUG--GUUAGGGGUGGa-5' miRNA<br>16 15 14 13 7 6 5 4 3 2<br>3'pairing Seed | 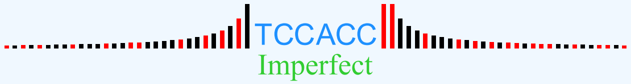 | 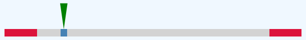 | ☐ | M |

| 2D Structure | Local AU | Position | Conservation | Predicted By |
|---|---|---|---|---|
| 127 7mer-m8 148<br>5'-gacGTTCCATAAAACCCCACCt-3' UTR<br>:\| \|\|\|: \|\| \|\|\|\|\|\|\|<br>3'-cauUACGGUGGUUAGGGGUGGa-5' miRNA<br>16 15 14 13 7 6 5 4 3 2<br>3'pairing Seed | 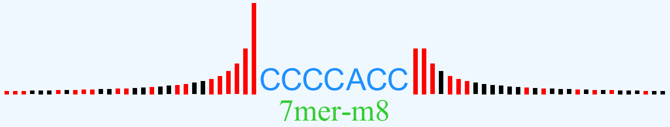 | 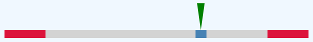 | | M T |

<table>
<tr><th>2D Structure</th><th>Local AU</th><th>Position</th><th>Conservation</th><th>Predicted By</th></tr>
<tr><td>Imperfect match<br>121 143<br>5'-cccATCCCAACTTACCCCCATCt-3' UTR<br>||  ||  ||:  |  ||||:|<br>3'-cauUACGG-UGGUUAGGGGUGGa-5' miRNA<br>16 15 14 13 7 6 5 4 3 2<br>3'pairing Seed</td><td>CCCATC<br>Imperfect</td><td></td><td></td><td>M</td></tr>
<tr><td>Offset 6mer<br>309 333<br>5'-ccccTGCCACTGCCCAACCCCACTt-3' UTR<br>|||||  ||  |  ||||||:<br>3'-cauuACGGU---GGUUAGGGGUGGa-5' miRNA<br>16 15 14 13 7 6 5 4 3 2<br>3'pairing Seed</td><td>CCCCAC<br>Offset 6mer</td><td></td><td></td><td>M</td></tr>
<tr><td>Offset 6mer<br>512 534<br>5'-catATGCAGCAACAGCCCCACTg-3' UTR<br>||||  :|  |  |  ||||||:<br>3'-cauUACGGUGGU-UAGGGGUGGa-5' miRNA<br>16 15 14 13 7 6 5 4 3 2<br>3'pairing Seed</td><td>CCCCAC<br>Offset 6mer</td><td></td><td></td><td>M</td></tr>
<tr><td>8mer<br>964 986<br>5'-cattTGGGATGACATCCCCACCa-3' UTR<br>||  |:  |  ||||||||||<br>3'-cauuACGGUGGU-UAGGGGUGGa-5' miRNA<br>16 15 14 13 7 6 5 4 3 2<br>3'pairing Seed</td><td>CCCCACCA<br>8mer</td><td></td><td></td><td>M T</td></tr>
</table>

| 2D Structure | Local AU | Position | Conservation | Predicted By |
|---|---|---|---|---|
| 299 8mer 320<br>5'-aaAAT-TCATCAAGACCCCACCa-3' UTR<br>\|\|\| :\|\|:\|\|\| \|\|\|\|\|\|\|<br>3'-caUUACGGUGGUU-AGGGGUGGa-5' miRNA<br>16 15 14 13 7 6 5 4 3 2<br>3'pairing Seed | 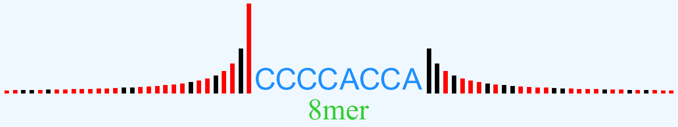 | 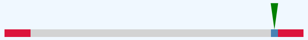 | | M T |

| 2D Structure | Local AU | Position | Conservation | Predicted By |
|---|---|---|---|---|
| 7mer-m8<br>232 252<br>5'-cttctctgGCTA-ATCAGCAGc-3' UTR<br>3'-gcccuucaCGAUCGAGUCGUCa-5' miRNA<br>16 15 14 13 7 6 5 4 3 2<br>3'pairing Seed | 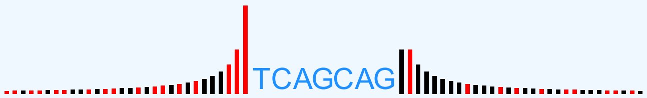<br>TCAGCAG<br>7mer-m8 | 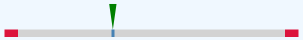 | ☐ | M T |
| Offset 6mer<br>262 287<br>5'-cctGTGGTGCTAGGCAACTCAGCAag-3' UTR<br>3'-gccCUUCACGAUC----GAGUCGUca-5' miRNA<br>16 15 14 13 7 6 5 4 3 2<br>3'pairing Seed | 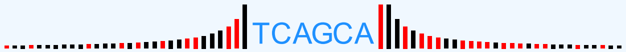<br>TCAGCA<br>Offset 6mer | 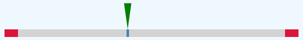 | ☐ | M |
| Offset 6mer<br>533 555<br>5'-tgcccAGTACCTCGCTCAGCAcg-3' UTR<br>3'-gcccuUCA-CGAUCGAGUCGUca-5' miRNA<br>16 15 14 13 7 6 5 4 3 2<br>3'pairing Seed | 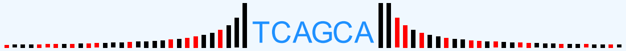<br>TCAGCA<br>Offset 6mer | 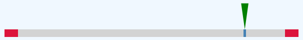 | ☐ | M |

| 2D Structure | Local AU | Position | Conservation | Predicted By |
|---|---|---|---|---|
| 60 8mer 82<br>5'-gaatcaagtTGGACAGCACTTTa-3' UTR<br>:\|:\|\|\|\|\|\|\|\|\|\|<br>3'-gauggacguGCUUGUCGUGAAAc-5' miRNA<br>16 15 14 13 7 6 5 4 3 2<br>3'pairing Seed | 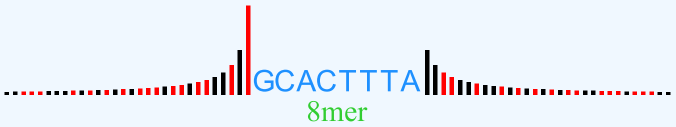 | 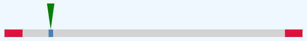 | | M T |
| Imperfect match<br>378 397<br>5'-aaACCTGACCG---AGTACTTTg-3' UTR<br>\|\|\|\|\| \|\| \|\|:\|\|\|\|\|<br>3'-gaUGGACGUGCUUGUCGUGAAAc-5' miRNA<br>16 15 14 13 7 6 5 4 3 2<br>3'pairing Seed | 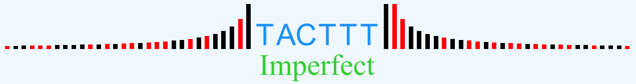 | 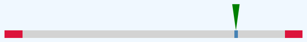 | | M |

| 2D Structure | Local AU | Position | Conservation | Predicted By |
|---|---|---|---|---|
| 353 7mer-m8 376<br>5'-gaCACTTATCATCGGTGTACTGTg-3' UTR<br>3'-aaGUCAAUAGU-GU--CAUGACAu-5' miRNA<br>16 15 14 13 / 7 6 5 4 3 2<br>3'pairing Seed | 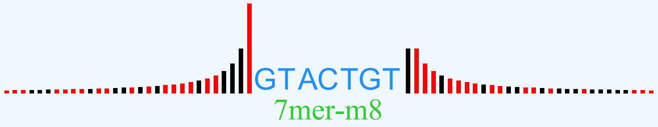 | 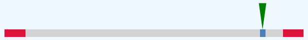 | | M T |

| 2D Structure | Local AU | Position | Conservation | Predicted By |
| --- | --- | --- | --- | --- |
| Imperfect match<br>32 5'-tgATGAGCATT-TAATAATTGc -3' 52 UTR<br>3'-cgUAGUCGUGACACUAUUGACu-5' miRNA<br>16 15 14 13 7 6 5 4 3 2<br>3'pairing Seed | 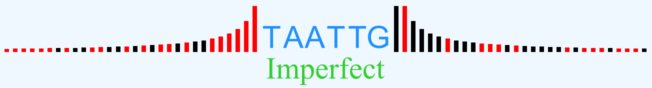 | 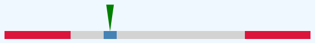 | | M |

| 2D Structure | Local AU | Position | Conservation | Predicted By |
|---|---|---|---|---|
| 235 7mer-m8 253<br>5'-tcttcccaggaAGTACTGg-3' UTR<br>\|\|\|\|\|\|\|<br>3'-agucaauaguguCAUGACa-5' miRNA<br>16 15 14 13 7 6 5 4 3 2<br>3'pairing Seed | 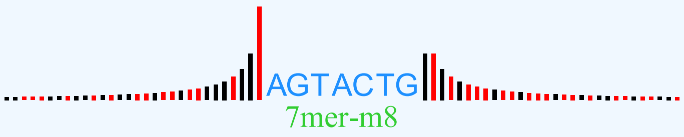 | 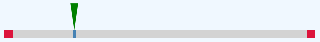 | ☐ | M T |
| 541 7mer-m8 558<br>5'-tgcagcATGA-AGTACTGt-3' UTR<br>\|\| \| \|\|\|\|\|\|\|<br>3'-agucaaUAGUGUCAUGACa-5' miRNA<br>16 15 14 13 7 6 5 4 3 2<br>3'pairing Seed | 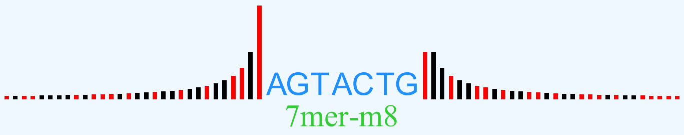 | 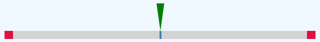 | ☐ | M T |
| Imperfect match<br>615 632<br>5'-caAGTGGTCA-AGTGCTGg -3' UTR<br>\|\|\| :\|\|\| \|\|\|:\|\|\|<br>3'-agUCAAUAGUGUCAUGACa-5' miRNA<br>16 15 14 13 7 6 5 4 3 2<br>3'pairing Seed | 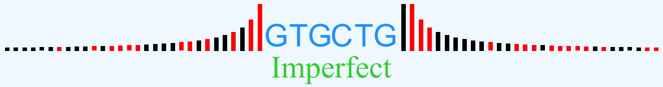 | 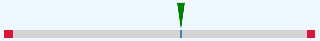 | ☐ | M |

<table>
<tr><th>2D Structure</th><th>Local AU</th><th>Position</th><th>Conservation</th><th>Predicted By</th></tr>
<tr><td>Imperfect match<br>480 504<br>5'-gcaacCAGCACCAGATGAAGGAGCa-3' UTR<br>| | | | |  |  |  | : | | | : | | |<br>3'-guuccGUCGU-GAC-AUUUCUUCGg-5' miRNA<br>16 15 14 13 7 6 5 4 3 2<br>3'pairing Seed</td><td>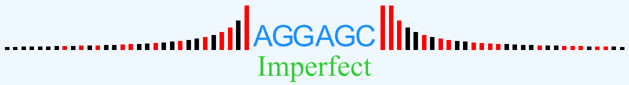</td><td>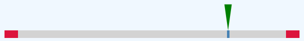</td><td></td><td>M</td></tr>
<tr><td>7mer-m8<br>512 534<br>5'-tggaaatcCCCTGCAAAGAAGCt-3' UTR<br>|  | | |  | | | | | | | |<br>3'-guuccgucGUGACAUUUCUUCGg-5' miRNA<br>16 15 14 13 7 6 5 4 3 2<br>3'pairing Seed</td><td>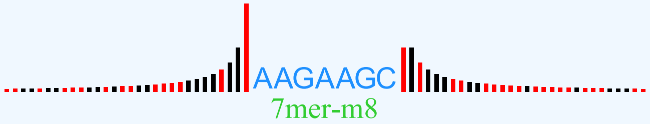</td><td>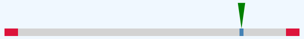</td><td></td><td>M T</td></tr>
</table>

| 2D Structure | Local AU | Position | Conservation | Predicted By |
|---|---|---|---|---|
| 28 7mer-m8 48<br>5'-tcgtGTGGCA--GTTAAGAAGCt-3' UTR<br>3'-guucCGUCGUGACAUUUCUUCGg-5' miRNA<br>16 15 14 13 7 6 5 4 3 2<br>3'pairing Seed | 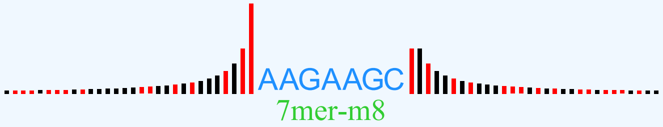 | 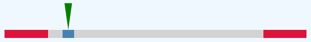 | | M T |

<table>
<tr><th>2D Structure</th><th>Local AU</th><th>Position</th><th>Conservation</th><th>Predicted By</th></tr>
<tr>
<td>155 7mer-m8 179<br>5'-agAGGTAGTTGTGGATGAAGAAGCt-3' UTR<br>     |||:||:  || |:|||||||<br>3'-guUCCGUCGUGAC--AUUUCUUCGg-5' miRNA<br>16 15 14 13 7 6 5 4 3 2<br>3'pairing Seed</td>
<td>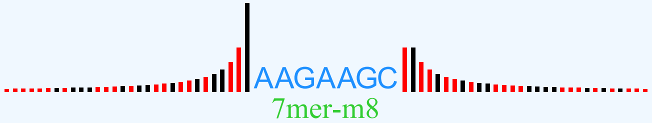</td>
<td>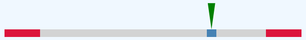</td>
<td>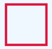</td>
<td>M T</td>
</tr>
</table>

<table>
<tr><th>2D Structure</th><th>Local AU</th><th>Position</th><th>Conservation</th><th>Predicted By</th></tr>
<tr><td>Imperfect match<br>480 504<br>5'-gcaacCAGCACCAGATGAAGGAGCa-3' UTR<br>||||| | | |:|||:|||<br>3'-guuccGUCGU-GAC-AUUUCUUCGa-5' miRNA<br>16 15 14 13 7 6 5 4 3 2<br>3'pairing Seed</td><td>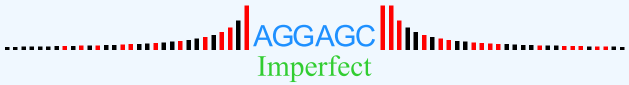
</td><td>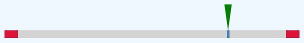</td><td>☐</td><td>M</td></tr>
<tr><td>7mer-m8<br>512 534<br>5'-tggaaatcCCCTGCAAAGAAGCt-3' UTR<br>| ||| |||||||<br>3'-guuccgucGUGACAUUUCUUCGa-5' miRNA<br>16 15 14 13 7 6 5 4 3 2<br>3'pairing Seed</td><td>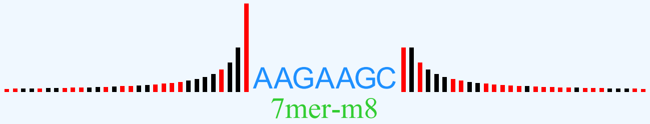
</td><td>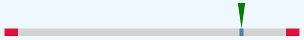</td><td>☐</td><td>M T</td></tr>
</table>

| 2D Structure | Local AU | Position | Conservation | Predicted By |
| --- | --- | --- | --- | --- |
| 28 7mer-m8 48<br>5'-tcgtGTGGCA--GTTAAGAAGCt-3' UTR<br>|:: ||| || |||||||<br>3'-guucCGUCGUGACAUUUCUUCGa-5' miRNA<br>16 15 14 13 7 6 5 4 3 2<br>3'pairing Seed | 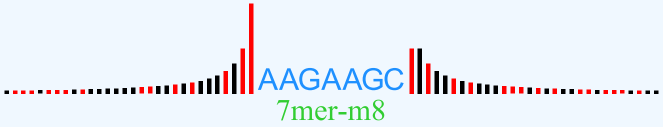 | 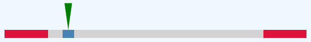 | | M T |

| 2D Structure | Local AU | Position | Conservation | Predicted By |
|---|---|---|---|---|
| Offset 6mer<br>45 … 68<br>5'-ctGGCGGGATGGGCTTATACCTga-3' UTR<br>3'-uaUUGUCGUGUUUGAUUAUGGAgu-5' miRNA<br>16 15 14 13 … 7 6 5 4 3 2<br>3'pairing … Seed | ATACCT<br>Offset 6mer | | | M |
| Imperfect match<br>354 … 377<br>5'-agAACTGCAGGAGTCAGTACTTCa -3' UTR<br>3'-uaUUGUCGUGUUUGAUUAUGGAGu-5' miRNA<br>16 15 14 13 … 7 6 5 4 3 2<br>3'pairing … Seed | TACTTC<br>Imperfect | | | M |

<table>
<tr><th>2D Structure</th><th>Local AU</th><th>Position</th><th>Conservation</th><th>Predicted By</th></tr>
<tr><td>181 7mer-m8 203<br>5'-gccctGGCTGCACTGCACTGTGg-3' UTR<br>|:|:|   |  ||||||<br>3'-uuucuCUGGC--CAAGUGACACu-5' miRNA<br>16 15 14 13 7 6 5 4 3 2<br>3'pairing Seed</td><td>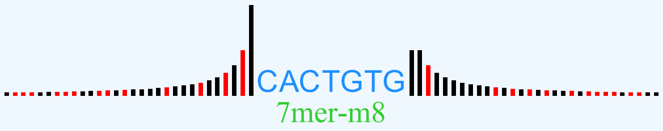</td><td>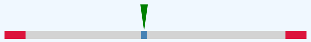</td><td></td><td>M T</td></tr>
<tr><td>357 6mer 377<br>5'-cttatcATCGGTGTACTGTGt-3' UTR<br>|:||||  :||||||<br>3'-uuucucUGGCCAAGUGACACu-5' miRNA<br>16 15 14 13 7 6 5 4 3 2<br>3'pairing Seed</td><td>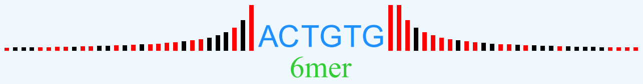</td><td>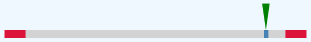</td><td></td><td>M</td></tr>
</table>

| 2D Structure | Local AU | Position | Conservation | Predicted By |
|---|---|---|---|---|
| 155 7mer-m8 179<br>5'-agAGGTAGTTGTGGATGAAGAAGCt-3' UTR<br>\|\|\|:\|\|\|: \|\| \|:\|\|\|\|\|\|\|<br>3'-guUCCGUCGUGAC--AUUUCUUCGa-5' miRNA<br>16 15 14 13 7 6 5 4 3 2<br>3'pairing Seed | 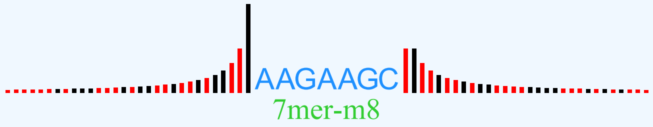 | 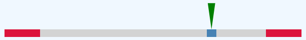 | | M T |

| 2D Structure | Local AU | Position | Conservation | Predicted By |
|---|---|---|---|---|
| 231 7mer-m8 255<br>5'-ccGTGGATGTCAACAACATGCTGCg-3' UTR<br>:\|:\| : \| \|\|\| \|\|\|\|\|\|\|<br>3'-agUAUCGGGACAUGU--UACGACGa-5' miRNA<br>16 15 14 13 7 6 5 4 3 2<br>3'pairing Seed | 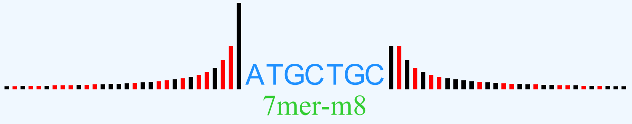 | 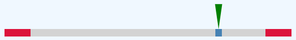 | | M T |
| 249 8mer 276<br>5'-tgctgcgaCTGTACGCCAGCATGCTGCa-3' UTR<br>\|\|\|\|\|\|\|: \|\|\|\|\|\|\|<br>3'-aguaucggGACAUGU-----UACGACGa-5' miRNA<br>16 15 14 13 7 6 5 4 3 2<br>3'pairing Seed | 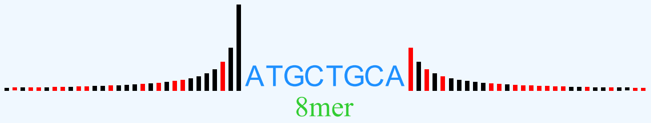 | 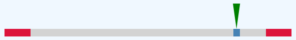 | | M T |

<table>
<tr><th>2D Structure</th><th>Local AU</th><th>Position</th><th>Conservation</th><th>Predicted By</th></tr>
<tr><td>339 7mer-m8 364<br>5'-gtccgccGTGTCTGGAGAACTGCAGg-3' UTR<br>          ||| |||  | |||||||<br>3'-cauucuuCAC-GACC--CGUGACGUCa-5' miRNA<br>16 15 14 13 7 6 5 4 3 2<br>3'pairing Seed</td><td>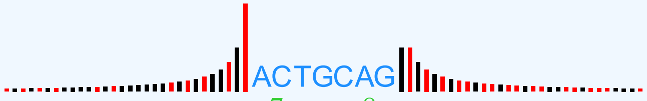<br>ACTGCAG<br>7mer-m8</td><td>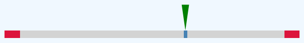</td><td>☐</td><td>M T</td></tr>
<tr><td>528 7mer-m8 550<br>5'-gcggcttccccaaGGACTGCAGt-3' UTR<br>                | |||||||<br>3'-cauucuucacgacCGUGACGUCa-5' miRNA<br>16 15 14 13 7 6 5 4 3 2<br>3'pairing Seed</td><td>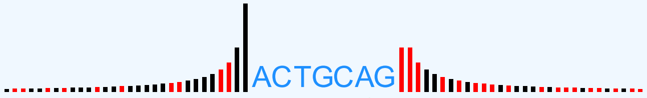<br>ACTGCAG<br>7mer-m8</td><td>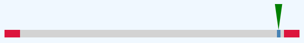</td><td>☐</td><td>M T</td></tr>
</table>

<table>
<tr><th>2D Structure</th><th>Local AU</th><th>Position</th><th>Conservation</th><th>Predicted By</th></tr>
<tr><td>Offset<br>6mer<br>84 108<br>5'-ccAGGAAGTGTCTGTCCACTGCAag-3' UTR<br>|:||||| ||| ||||||<br>3'-caUUCUUCAC-GAC-CGUGACGUca-5' miRNA<br>16 15 14 13 7 6 5 4 3 2<br>3'pairing Seed</td><td>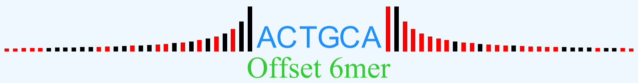</td><td>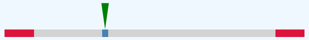</td><td></td><td>M</td></tr>
</table>

| 2D Structure | Local AU | Position | Conservation | Predicted By |
| --- | --- | --- | --- | --- |
| 231 7mer-m8 255<br>5'-ccGTGGATGTCAACAACATGCTGCg-3' UTR<br>:\|:\| : \| \|\|\| \|\|\|\|\|\|\|<br>3'-acUAUCGGGACAUGU--UACGACGa-5' miRNA<br>16 15 14 13 7 6 5 4 3 2<br>3'pairing Seed | 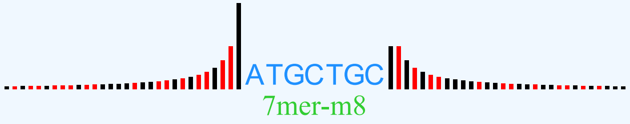 | 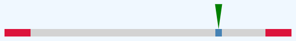 | | M T |
| 249 8mer 276<br>5'-tgctgcgaCTGTACGCCAGCATGCTGCa-3' UTR<br>\|\|\|\|\|\|\|: \|\|\|\|\|\|\|<br>3'-acuaucggGACAUGU-----UACGACGa-5' miRNA<br>16 15 14 13 7 6 5 4 3 2<br>3'pairing Seed | 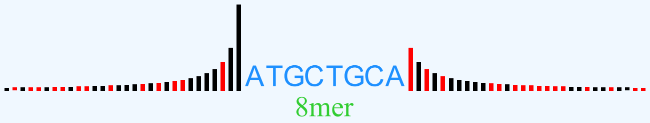 | 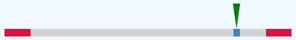 | | M T |

| 2D Structure | Local AU | Position | Conservation | Predicted By |
|---|---|---|---|---|

```
155                    7mer-m8  179
   5'-agAGGTAGTTGTGGATGAAGAAGCt-3'  UTR
        |||:|::  ||  |:||||||||
   3'-guUCCGUUGUGAC--AUUUCUUCGa-5'  miRNA
         16 15 14 13     7 6 5 4 3 2
           3'pairing        Seed
```

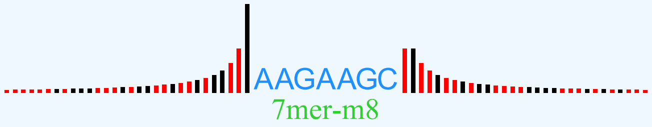


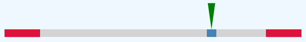

M T

| 2D Structure | Local AU | Position | Conservation | Predicted By |
|---|---|---|---|---|
| 283 7mer-m8 306<br>5'-ccAGTACCAAAGGCAAACACTCCt-3' UTR<br>\|::\|\|\|\| \| \| \|\|\|\|\|\|\|<br>3'-guUUGUGGUAACAG--UGUGAGGu-5' miRNA<br>16 15 14 13 7 6 5 4 3 2<br>3'pairing Seed | 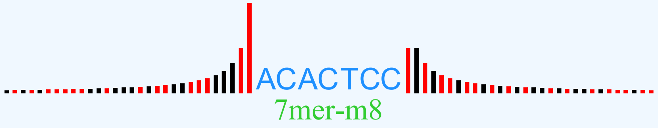 | | | M T |
| 308 6mer 328<br>5'-tcAACTTGAGTGT-GCACTCCt-3' UTR<br>\|\|\| : \| \|\|\| :\|\|\|\|\|\|<br>3'-guUUGUGGUAACAGUGUGAGGu-5' miRNA<br>16 15 14 13 7 6 5 4 3 2<br>3'pairing Seed | 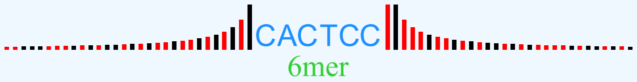 | | | M |

<table>
<tr><th>2D Structure</th><th>Local AU</th><th>Position</th><th>Conservation</th><th>Predicted By</th></tr>
<tr><td>113 7mer-m8 136<br>5'-ccAGTACCAAAGGCAAACACTCCt-3' UTR<br>|::|||  |  |  |||||||<br>3'-guUUGUGGUAACAG--UGUGAGGu-5' miRNA<br>16 15 14 13 7 6 5 4 3 2<br>3'pairing Seed</td><td>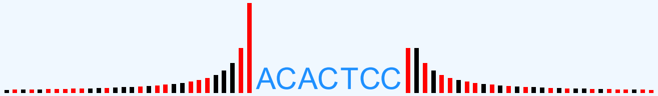</td><td>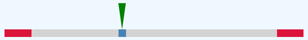</td><td></td><td>M T</td></tr>
<tr><td>138 6mer 158<br>5'-tcAACTTGAGTGT-GCACTCCt-3' UTR<br>||| : | ||| :||||||<br>3'-guUUGUGGUAACAGUGUGAGGu-5' miRNA<br>16 15 14 13 7 6 5 4 3 2<br>3'pairing Seed</td><td>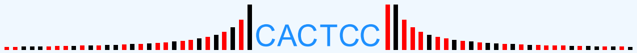</td><td>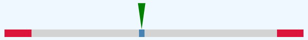</td><td></td><td>M</td></tr>
</table>

<table>
<tr><th>2D Structure</th><th>Local AU</th><th>Position</th><th>Conservation</th><th>Predicted By</th></tr>
<tr><td>181 7mer-m8 203<br>5'-gccctGGCTGCACTGCACTGTGg-3' UTR<br>|:|:| | |||||||<br>3'-uuucuCUGGC--CAAGUGACACu-5' miRNA<br>16 15 14 13 7 6 5 4 3 2<br>3'pairing Seed</td><td>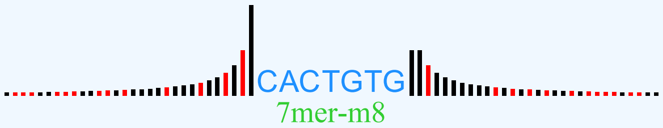</td><td>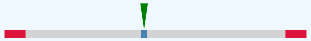</td><td></td><td>M T</td></tr>
<tr><td>357 6mer 377<br>5'-cttatcATCGGTGTACTGTGt-3' UTR<br>|:|||| :||||||<br>3'-uuucucUGGCCAAGUGACACu-5' miRNA<br>16 15 14 13 7 6 5 4 3 2<br>3'pairing Seed</td><td>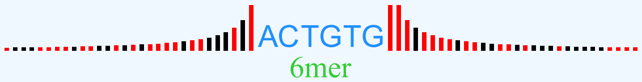</td><td>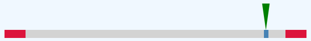</td><td></td><td>M</td></tr>
</table>

| 2D Structure | Local AU | Position | Conservation | Predicted By |
|---|---|---|---|---|
| 19 8mer 40<br>5'-catGGCACGTTTAACAAAAAGa-3' UTR<br>: \| \| \| \| : \| \| \| \| \| \| \|<br>3'-ccuUCGGGAAUGGGGUUUUUCg-5' miRNA<br>16 15 14 13 7 6 5 4 3 2<br>3'pairing Seed | 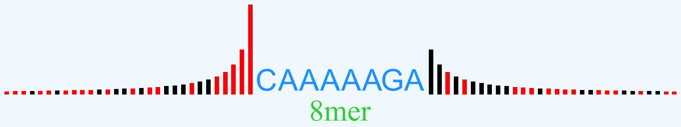 | 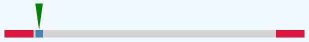 | | M T |
| Imperfect match<br>152 173<br>5'-cacggtatgcATCCCAAAGAGa-3' UTR<br>\| : \| \| \| \| \| \| : \| \|<br>3'-ccuucgggaaUGGGGUUUUUCg-5' miRNA<br>16 15 14 13 7 6 5 4 3 2<br>3'pairing Seed | 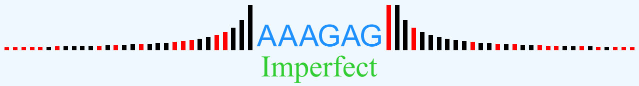 | 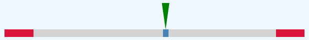 | | M |

| 2D Structure | Local AU | Position | Conservation | Predicted By |
|---|---|---|---|---|
| 247 7mer-m8 266<br>5'-ttTGGTGT--CTGGGAAAGAGg-3' UTR<br>|:||||: | |||||||||<br>3'-ucAUCACGUUGUCCCUUUCUCa-5' miRNA<br>16 15 14 13 7 6 5 4 3 2<br>3'pairing Seed | 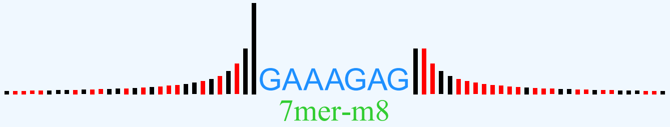 | 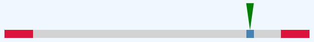 | | M T |

<table>
<tr><th>2D Structure</th><th>Local AU</th><th>Position</th><th>Conservation</th><th>Predicted By</th></tr>
<tr><td>411 7mer-m8 433<br>5'-agtaccgtGAACAGGAAGCCATt-3' UTR<br>||| |::|||||||<br>3'-aguguaucCUUAUUUUUCGGUAu-5' miRNA<br>16 15 14 13 7 6 5 4 3 2<br>3'pairing Seed</td><td>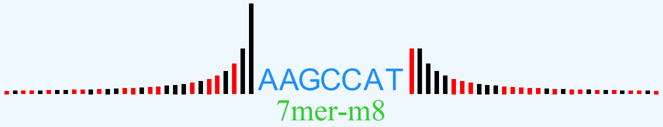</td><td>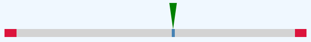</td><td>☐</td><td>M T</td></tr>
<tr><td>Offset 6mer<br>630 651<br>5'-gtcaACAGGAGT-ATAAGCCAcg-3' UTR<br>| ||||:| | ||||||<br>3'-agugUAUCCUUAUUUUUCGGUau-5' miRNA<br>16 15 14 13 7 6 5 4 3 2<br>3'pairing Seed</td><td>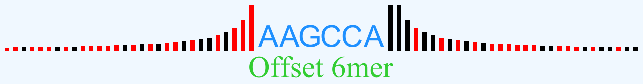</td><td>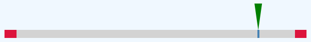</td><td>☐</td><td>M</td></tr>
</table>

| 2D Structure | Local AU | Position | Conservation | Predicted By |
|---|---|---|---|---|
| Offset 6mer<br>152 173<br>5'-aaACAT-TTAATGTAAAGCCACa-3' UTR<br>3'-agUGUAUCCUUAUUUUUCGGUau-5' miRNA<br>16 15 14 13 / 7 6 5 4 3 2<br>3'pairing Seed | 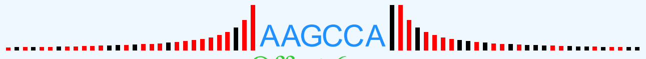 | 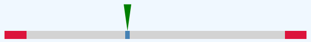 | | M |
| 7mer-m8<br>183 205<br>5'-caagtgccttgccAGAAGCCATg-3' UTR<br>3'-aguguauccuuauUUUUCGGUAu-5' miRNA<br>16 15 14 13 / 7 6 5 4 3 2<br>3'pairing Seed | 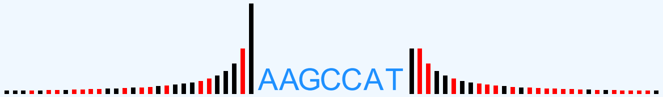 | 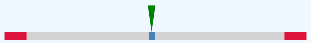 | | M T |

| 2D Structure | Local AU | Position | Conservation | Predicted By |
|---|---|---|---|---|
| 411 7mer-m8 433<br>5'-agtaccgtGAACAGGAAGCCATt-3' UTR<br>\|\|\|   ::\|\|\|\|\|\|<br>3'-aguguaucCUUACUUUUCGGUAu-5' miRNA<br>16 15 14 13 7 6 5 4 3 2<br>3'pairing Seed | 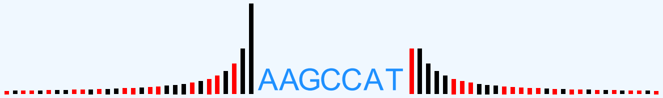  | 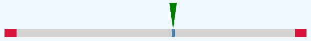 | | M T |
| Offset 6mer<br>630 651<br>5'-gtcaACAGGAGT-ATAAGCCAcg-3' UTR<br>\|  \|\|\|\|:\|  \|  \|\|\|\|\|\|<br>3'-agugUAUCCUUACUUUUCGGUau-5' miRNA<br>16 15 14 13 7 6 5 4 3 2<br>3'pairing Seed | 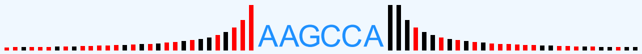  | 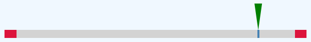 | | M |

| 2D Structure | Local AU | Position | Conservation | Predicted By |
| --- | --- | --- | --- | --- |
| Offset 6mer<br>152 173<br>5'-aaACAT-TTAATGTAAAGCCACa-3' UTR<br>\|\|\|\| \|\|\|\| \|\|\|\|\|\|\|<br>3'-agUGUAUCCUUACUUUUCGGUAu-5' miRNA<br>16 15 14 13 7 6 5 4 3 2<br>3'pairing Seed | 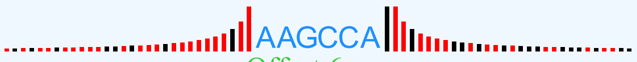 | 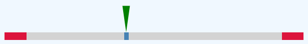 | ☐ | M |
| 183 7mer-m8 205<br>5'-caagtgccttgccAGAAGCCATg-3' UTR<br>\|:\|\|\|\|\|\|\|<br>3'-aguguaucuccuuacUUUUCGGUAu-5' miRNA<br>16 15 14 13 7 6 5 4 3 2<br>3'pairing Seed | 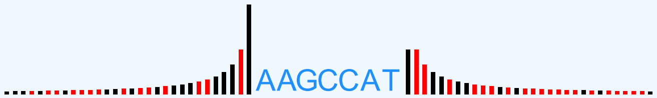 | 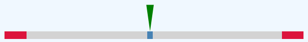 | ☐ | M T |
| 336 7mer-A1 356<br>5'-aatTAAAAGAATG--GAGCCATa-3' UTR<br>:\| \| \|\|\|\|\| :\|\|\|\|\|\|<br>3'-aguGUAUCCUUACUUUUCGGUAu-5' miRNA<br>16 15 14 13 7 6 5 4 3 2<br>3'pairing Seed | 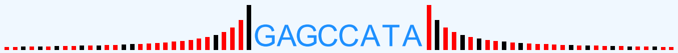 | 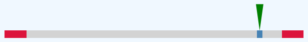 | ☐ | M T |

<table>
<tr><th>2D Structure</th><th>Local AU</th><th>Position</th><th>Conservation</th><th>Predicted By</th></tr>
<tr><td>52 7mer-m8 73<br>5'-ccgcCCTGAAGGCGAATGGAGt-3' UTR<br>| | ||::|:||||||<br>3'-gguaGUAGUUUUGUUUACCUCa-5' miRNA<br>16 15 14 13 7 6 5 4 3 2<br>3'pairing Seed</td><td>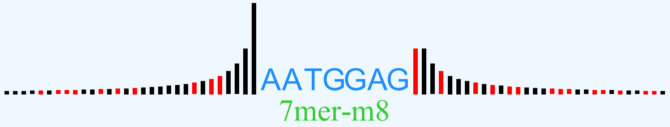
</td><td>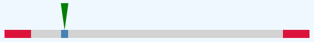</td><td></td><td>M T</td></tr>
</table>

<table>
<tr><th>2D Structure</th><th>Local AU</th><th>Position</th><th>Conservation</th><th>Predicted By</th></tr>
<tr><td>Imperfect match<br>54 76<br>5'-cctcagaATGTCCAACATCAGCt -3' UTR<br>|| : |||||:||||<br>3'-gccggacUAAGUGUUGUGGUCGa-5' miRNA<br>16 15 14 13 7 6 5 4 3 2<br>3'pairing Seed</td><td>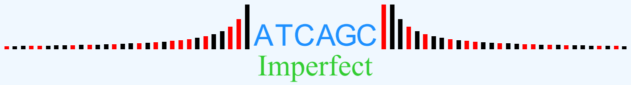
</td><td>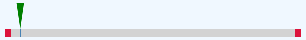</td><td>☐</td><td>M</td></tr>
<tr><td>Imperfect match<br>150 170<br>5'-gcGCCT--TTCGCTCCATCAGCc -3' UTR<br>|||| |||:| ||:||||<br>3'-gcCGGACUAAGUGUUGUGGUCGa-5' miRNA<br>16 15 14 13 7 6 5 4 3 2<br>3'pairing Seed</td><td>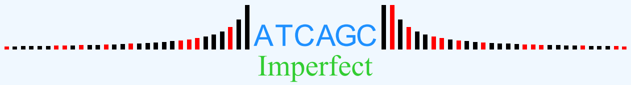
</td><td>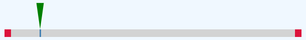</td><td>☐</td><td>M</td></tr>
<tr><td>Imperfect match<br>854 877<br>5'-ccagTTGATACAGCAACAGCAGCa -3' UTR<br>:|||| || ||||| ||||<br>3'-gccgGACUAAGU-GUUGUGGUCGa-5' miRNA<br>16 15 14 13 7 6 5 4 3 2<br>3'pairing Seed</td><td>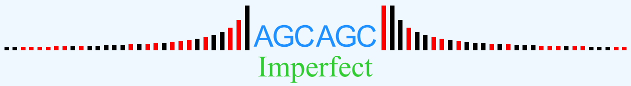
</td><td>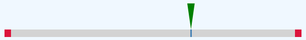</td><td>☐</td><td>M</td></tr>
<tr><td>7mer-m8<br>975 999<br>5'-acatccccacCACCACCCACCAGCc-3' UTR<br>||| | |||||||<br>3'-gccggacuaaGUGUU--GUGGUCGa-5' miRNA<br>16 15 14 13 7 6 5 4 3 2<br>3'pairing Seed</td><td>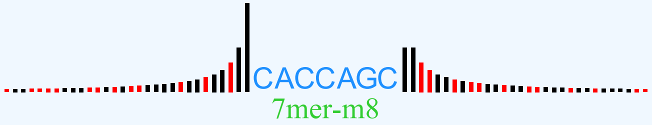
</td><td>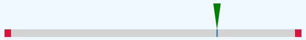</td><td>☐</td><td>M T</td></tr>
</table>

| 2D Structure | Local AU | Position | Conservation | Predicted By |
| --- | --- | --- | --- | --- |
| 26 7mer-m8 48<br>5'-caaGACATTCTTACTGGAAGATt-3' UTR<br>:\|\|\|:\|  :\|\|\|\|\|\|\|\|\|\|<br>3'-aggUUGUGA-CGUGACCUUCUAc-5' miRNA<br>16 15 14 13 7 6 5 4 3 2<br>3'pairing Seed | 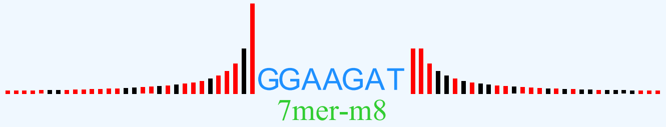 | 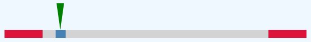 | | M T |

<table>
<tr><th>2D Structure</th><th>Local AU</th><th>Position</th><th>Conservation</th><th>Predicted By</th></tr>
<tr><td>166 8mer 190<br>5'-gaaaACAGCTATATCTTTGATATCa-3' UTR<br>|||| ||||| |||||||<br>3'-ugaaUGUC-AUAUA-CUACUAUAGg-5' miRNA<br>16 15 14 13 7 6 5 4 3 2<br>3'pairing Seed</td><td>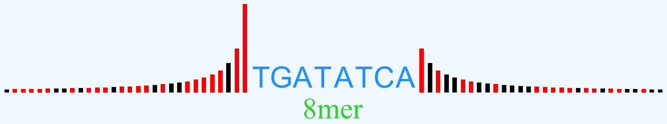
</td><td>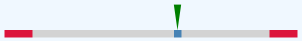</td><td></td><td>M T</td></tr>
</table>

| 2D Structure | Local AU | Position | Conservation | Predicted By |
| --- | --- | --- | --- | --- |
| 217 8mer 243<br>5'-gcaGATTACTTGGCCAAGGAACTGGAa-3' UTR<br>3'-uccCUAAGGACCC--UU--UUGACCUg-5' miRNA<br>16 15 14 13 7 6 5 4 3 2<br>3'pairing Seed | 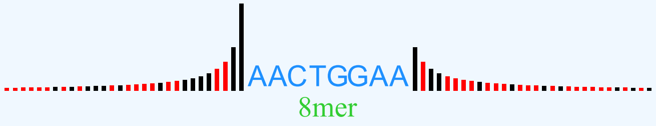 | 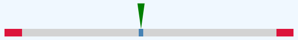 | | M T |
| 333 7mer-m8 355<br>5'-catcacaCCTGACATAACTGGAc-3' UTR<br>3'-ucccuaaGGACCCUUUUGACCUg-5' miRNA<br>16 15 14 13 7 6 5 4 3 2<br>3'pairing Seed | 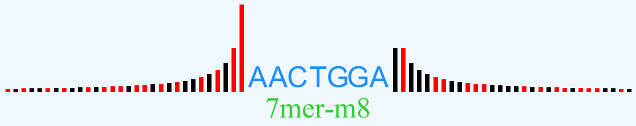 | 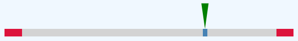 | | M T |

| 2D Structure | Local AU | Position | Conservation | Predicted By |
| --- | --- | --- | --- | --- |
| 47 8mer 73<br>5'-gcaGATTACTTGGCCAAGGAACTGGAa-3' UTR<br>3'-uccCUAAGGACCC--UU--UUGACCUg-5' miRNA<br>16 15 14 13 7 6 5 4 3 2<br>3'pairing Seed | 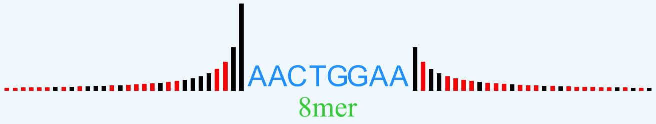 | 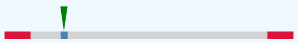 | | M T |
| 163 7mer-m8 185<br>5'-catcacaCCTGACATAACTGGAc-3' UTR<br>3'-ucccuaaGGACCCUUUUGACCUg-5' miRNA<br>16 15 14 13 7 6 5 4 3 2<br>3'pairing Seed | 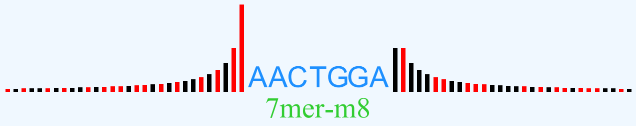 | 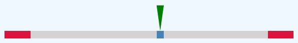 | | M T |

<table>
<tr><th>2D Structure</th><th>Local AU</th><th>Position</th><th>Conservation</th><th>Predicted By</th></tr>
<tr><td>220 8mer 243<br>5'-gaTTACTTGGCCAAGGAACTGGAa-3' UTR<br>| : | : | | | | | | | | | | | |<br>3'-ucAGAGGACC-CUU--UUGACCUg-5' miRNA<br>16 15 14 13 7 6 5 4 3 2<br>3'pairing Seed</td><td>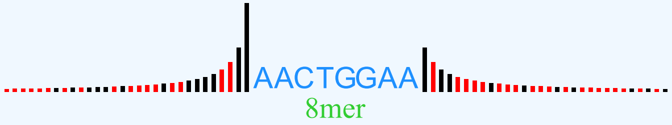</td><td>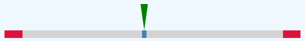</td><td>☐</td><td>M T</td></tr>
<tr><td>335 7mer-m8 355<br>5'-tcaCACCTGACATAACTGGAc-3' UTR<br>| | | | | | | | | | | | |<br>3'-ucaGAGGACCCUUUUGACCUg-5' miRNA<br>16 15 14 13 7 6 5 4 3 2<br>3'pairing Seed</td><td>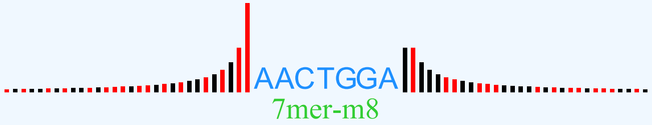</td><td>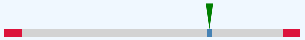</td><td>☐</td><td>M T</td></tr>
</table>

| 2D Structure | Local AU | Position | Conservation | Predicted By |
|---|---|---|---|---|
| 50 8mer 73<br>5'-gaTTACTTGGCCAAGGAACTGGAa-3' UTR<br>\|: \|:\|\|\| \|\| \|\|\|\|\|\|\|<br>3'-ucAGAGGACC-CUU--UUGACCUg-5' miRNA<br>16 15 14 13 7 6 5 4 3 2<br>3'pairing Seed | 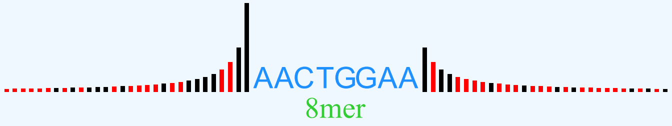 | 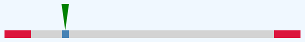 | ☐ | M T |
| 165 7mer-m8 185<br>5'-tcaCACCTGACATAACTGGAc-3' UTR<br>\| \|\|\|\| \| \|\|\|\|\|\|\|<br>3'-ucaGAGGACCCUUUUGACCUg-5' miRNA<br>16 15 14 13 7 6 5 4 3 2<br>3'pairing Seed | 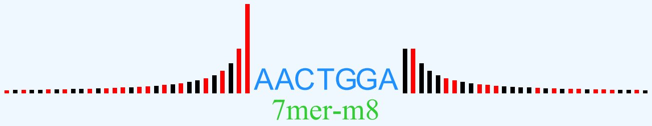 | 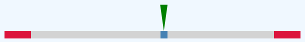 | ☐ | M T |

| 2D Structure | Local AU | Position | Conservation | Predicted By |
| --- | --- | --- | --- | --- |

```
117                     7mer-m8  142
 5'-tacAGGGATTGTATTACCTTCACAGt-3' UTR
       |:|:|:|| ||    |||||||
 3'-gacUUCUUGACUUA----AAGUGUCc-5' miRNA
          16151413     765432
         3'pairing      Seed
```

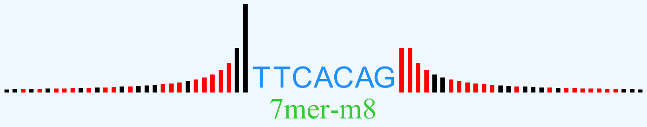


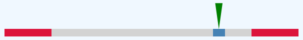

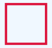

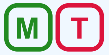

<table>
<tr><th>2D Structure</th><th>Local AU</th><th>Position</th><th>Conservation</th><th>Predicted By</th></tr>
<tr><td>Offset 6mer<br>35 58<br>5'-caacaaaaTGCGCGATTGTTTCTg-3' UTR<br>|||: :| ||||||:<br>3'-gaucaaacACGUCUUUACAAAGGu-5' miRNA<br>16 15 14 13 7 6 5 4 3 2<br>3'pairing Seed</td><td>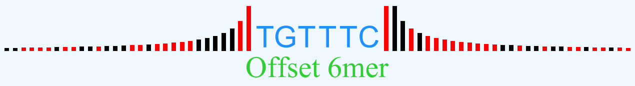<br></td><td></td><td></td><td>M</td></tr>
<tr><td>Imperfect match<br>180 204<br>5'-tatGTTTATGATAAAAATGTTTTac -3' UTR<br>|||| || :| ||||||||:<br>3'-gauCAAACAC-GUCUUUACAAAGgu-5' miRNA<br>16 15 14 13 7 6 5 4 3 2<br>3'pairing Seed</td><td>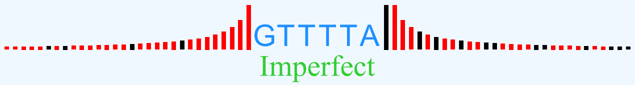<br></td><td></td><td></td><td>M</td></tr>
</table>

| 2D Structure | Local AU | Position | Conservation | Predicted By |
| --- | --- | --- | --- | --- |
| Offset 6mer<br>35 58<br>5'-caacaaaaTGCGCGATTGTTTCTg-3' UTR<br>\|\|\|: :\| \|\|\|\|\|:<br>3'-gaucaaacACGUCUUUACAAAGGu-5' miRNA<br>16 15 14 13 7 6 5 4 3 2<br>3'pairing Seed | 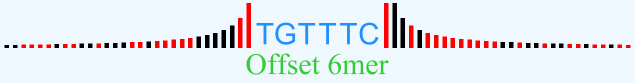 | 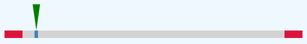 | ☐ | M |
| Imperfect match<br>180 204<br>5'-tatGTTTATGATAAAAATGTTTTac -3' UTR<br>\|\|\|\| \|\| :\| \|\|\|\|\|\|\|\|:<br>3'-gauCAAACAC-GUCUUUACAAAGgu-5' miRNA<br>16 15 14 13 7 6 5 4 3 2<br>3'pairing Seed | 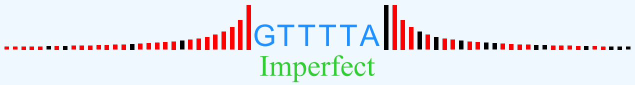 | 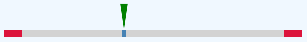 | ☐ | M |

| 2D Structure | Local AU | Position | Conservation | Predicted By |
|---|---|---|---|---|
| 109 8mer 131<br>5'-ccGCTGACAGCCGTGCCTCCCTa-3' UTR<br>:\|:\| \| \|\|\|\| \|\|\|\|\|\|\|<br>3'-cgUGGC-GGGGGCAGGGAGGGAg-5' miRNA<br>16 15 14 13 7 6 5 4 3 2<br>3'pairing Seed | 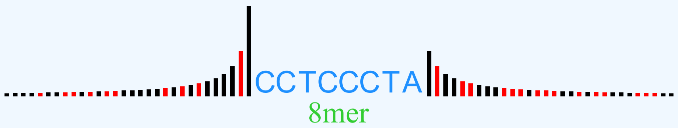 | 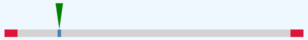 | ☐ | M T |
| 563 8mer 584<br>5'-gccctctggaggagCCTCCCTa-3' UTR<br>\|\|\|\|\|\|\|<br>3'-cguggcggggcagGGAGGGAg-5' miRNA<br>16 15 14 13 7 6 5 4 3 2<br>3'pairing Seed | 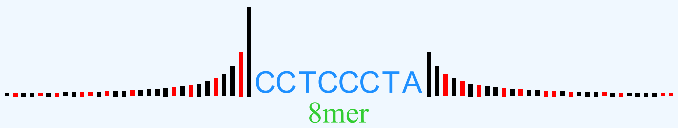 | 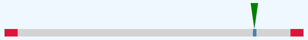 | ☐ | M T |

| 2D Structure | Local AU | Position | Conservation | Predicted By |
|---|---|---|---|---|
| Imperfect match<br>346 371<br>5'-gtgtcTGGAGAACTGCAGGAGTCAGt -3' UTR<br>\|\|:\|\|\| ::\| \|\|\|\|:\|\|\|<br>3'-cccucACUUCU--GUG-CCUCGGUCu-5' miRNA<br>16 15 14 13   7 6 5 4 3 2<br>3'pairing   Seed | 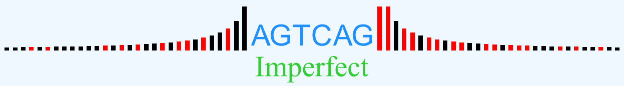 | 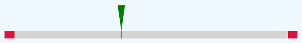 | | M |
| 7mer-m8<br>625 647<br>5'-aaaaaacAAGTCAGAGAGCCAGt-3' UTR<br>\|\|\| \|\| \|\|\|\|\|\|\|<br>3'-cccucacUUCUGUGCCUCGGUCu-5' miRNA<br>16 15 14 13   7 6 5 4 3 2<br>3'pairing   Seed | 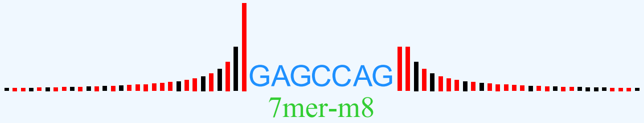 | 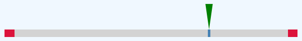 | | M T |

| 2D Structure | Local AU | Position | Conservation | Predicted By |
|---|---|---|---|---|
| 7mer-m8<br>140 162<br>5'-ctgtgaagtgAATCATGAATGTt-3' UTR<br>\|\|: \|\|\|\|\|\|\|\|<br>3'-ugggugg cugU UGUUA CUUACA a-5' miRNA<br>16 15 14 13 7 6 5 4 3 2<br>3'pairing Seed | 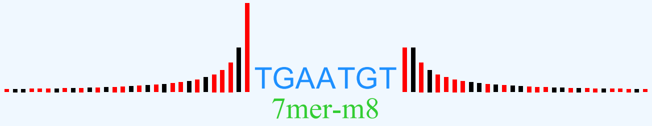 | 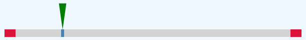 | ☐ | M T |
| Offset 6mer<br>410 434<br>5'-aaTCACCAAAACATACATGAATGgg-3' UTR<br>:\|\|\|\| \| \| \|:\| \|\|\|\|\|\|\|<br>3'-ugGGUGG CUGU-UGU-UA CUUACa a-5' miRNA<br>16 15 14 13 7 6 5 4 3 2<br>3'pairing Seed | 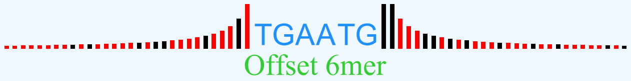 | 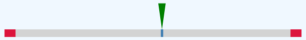 | ☐ | M |

| 2D Structure | Local AU | Position | Conservation | Predicted By |
| --- | --- | --- | --- | --- |
| 7mer-m8<br>139 5'-ctacctctcaGATTATTCTTTc-3' 160 UTR<br>\|\| \|\|\|\|\|\|\|<br>3'-ucggguuuucCUCUUAAGAAAc-5' miRNA<br>16 15 14 13 7 6 5 4 3 2<br>3'pairing Seed | 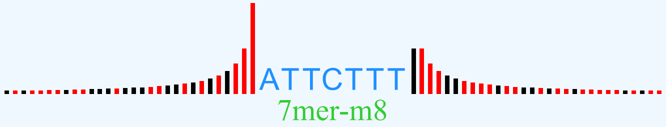 | 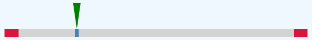 | ☐ | M T |
| Imperfect match<br>577 5'-acCTCAAAA--A-AATTTTTTt-3' 595 UTR<br>\|:\|\|\|\|\| \| \|\|\|\|:\|\|\|<br>3'-ucGGGUUUUCCUCUUAAGAAAc-5' miRNA<br>16 15 14 13 7 6 5 4 3 2<br>3'pairing Seed | 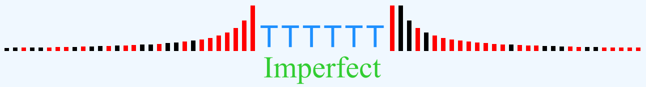 | 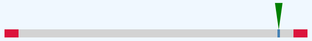 | ☐ | M |

| 2D Structure | Local AU | Position | Conservation | Predicted By |
| --- | --- | --- | --- | --- |
| 172 7mer-m8 193<br>5'-aaCTTTAGAGTCAAATTCTTTg-3' UTR<br>3'-ucGGGUUUUCCUCUUAAGAAAc-5' miRNA<br>16 15 14 13 7 6 5 4 3 2<br>3'pairing Seed | 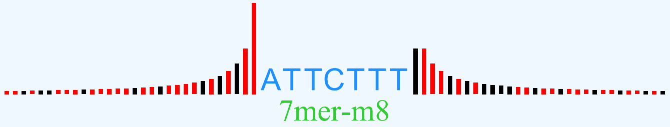  | 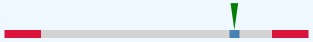 | | M T |

| 2D Structure | Local AU | Position | Conservation | Predicted By |
| --- | --- | --- | --- | --- |
| 185 7mer-m8 206<br>5'-tggctGCACTGCACTGTGGGAg-3' UTR<br>:\| \|\|\|\|\| \|\|\|\|\|\|\|<br>3'-acguuUGGGACGU-ACACCCUc-5' miRNA<br>16 15 14 13 7 6 5 4 3 2<br>3'pairing Seed | 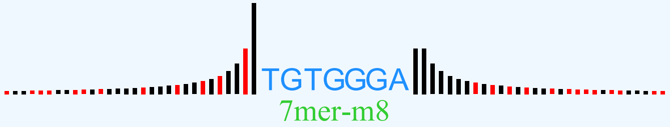 | 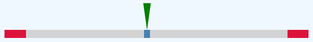 | | M T |

| 2D Structure | Local AU | Position | Conservation | Predicted By |
|---|---|---|---|---|
| 127 7mer-A1 148<br>5'-ccATCGTTTGTGAAGAGACCCa-3' UTR<br>\|\|\| \| : :\| \|:\|\|\|\|\|\|<br>3'-agUAGAACGGGCGUUUCUGGGu-5' miRNA<br>16 15 14 13 7 6 5 4 3 2<br>3'pairing Seed | 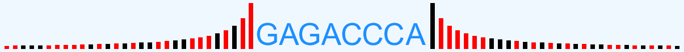<br>GAGACCCA<br>7mer-A1 | 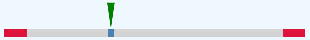 | ☐ | M T |
| 189 6mer 210<br>5'-atgtgagGCCCGAGGAGACCCt-3' UTR<br>\|\|\|\|\| ::\|\|\|\|\|\|<br>3'-aguagaaCGGGCGUUUCUGGGu-5' miRNA<br>16 15 14 13 7 6 5 4 3 2<br>3'pairing Seed | 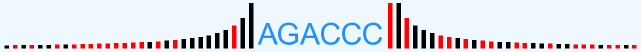<br>AGACCC<br>6mer | 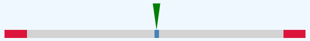 | ☐ | M |

| 2D Structure | Local AU | Position | Conservation | Predicted By |
|---|---|---|---|---|
| 122 6mer 145<br>5'-tcaGAAAACTTGGCAGGCTACCTc-3' UTR<br>:\| \|\|\| \|\| \|::\|\|\|\|\|\|<br>3'-gggUUGUUGUAC--UUUGAUGGAu-5' miRNA<br>16 15 14 13 7 6 5 4 3 2<br>3'pairing Seed | 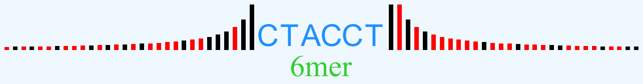<br> | 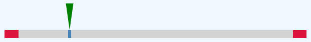 | | M |
| Imperfect match<br>235 256<br>5'-agaAGCAGAATTTAATTACCTa-3' UTR<br>\|:\|\|: \|\| \|\|:\|\|\|\|\|<br>3'-gggUUGUUGUACUUUGAUGGAu-5' miRNA<br>16 15 14 13 7 6 5 4 3 2<br>3'pairing Seed | 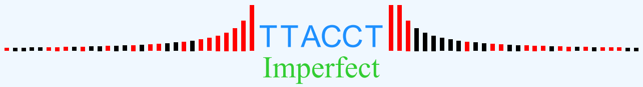<br> | 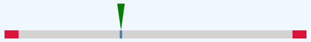 | | M |

| 2D Structure | Local AU | Position | Conservation | Predicted By |
| --- | --- | --- | --- | --- |
| 31 8mer 53<br>5'-tgtGGCAGTTAAGAAGCTGTCGa-3' UTR<br>\|\|\|\|\|\| \| \|\|\|\|\|\|\|<br>3'-cuuCCGUCACAGC-ACGACAGCu-5' miRNA<br>16 15 14 13 7 6 5 4 3 2<br>3'pairing Seed | 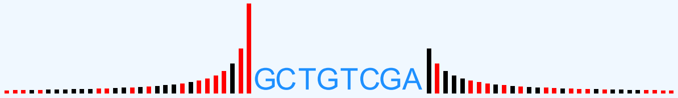 GCTGTCGA 8mer | 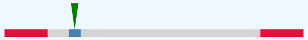 | 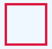 | M T |

| 2D Structure | Local AU | Position | Conservation | Predicted By |
|---|---|---|---|---|

```
18                        7mer-m8   42
 5'-agGAGCAGCTGACCTTGGTAAGACt-3'  UTR
        :|:|| |||   ||  |||||||
 3'-ggUUUGU-GAC--GACCCAUUCUGc-5'  miRNA
         16  15 14 13   7 6 5 4 3 2
       3'pairing        Seed
```

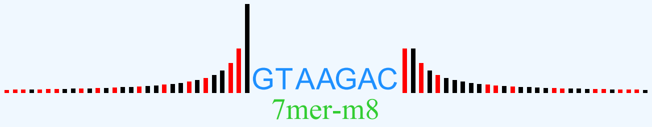


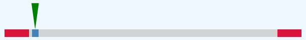

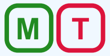

<table>
<tr><th>2D Structure</th><th>Local AU</th><th>Position</th><th>Conservation</th><th>Predicted By</th></tr>
<tr><td>659 7mer-m8 681<br>5'-caTTAAGATCCTCAAGAACCACc-3' UTR<br>|| | |: ||||||||||<br>3'-acAACU-UGACAGUUCUUGGUGa-5' miRNA<br>16 15 14 13 7 6 5 4 3 2<br>3'pairing Seed</td><td>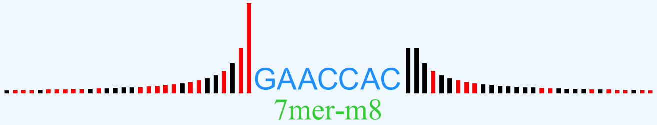</td><td>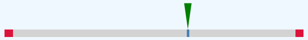</td><td>☐</td><td>M T</td></tr>
<tr><td>777 8mer 798<br>5'-tgcttccagcaCAAGAACCACa-3' UTR<br>||||||||||<br>3'-acaacuugacaGUUCUUGGUGa-5' miRNA<br>16 15 14 13 7 6 5 4 3 2<br>3'pairing Seed</td><td>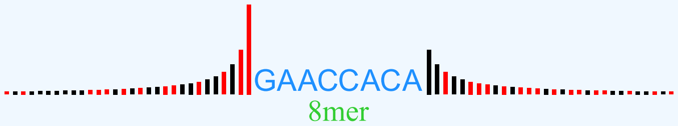</td><td>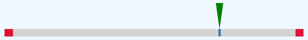</td><td>☐</td><td>M T</td></tr>
</table>

| 2D Structure | Local AU | Position | Conservation | Predicted By |
|---|---|---|---|---|
| 141 7mer-m8 160<br>5'-tggaaagTCTG-TTTCCCAGg-3' UTR<br>3'-ugcagggAAACGGAAGGGUCg-5' miRNA<br>16 15 14 13 7 6 5 4 3 2<br>3'pairing Seed | 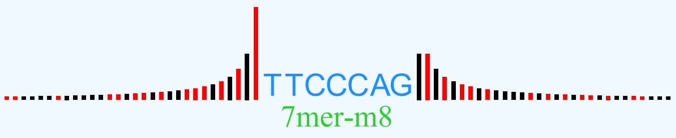 | 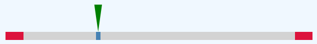 | | M T |
| 180 7mer-m8 200<br>5'-actggaacgcaCCTTCCCAGg-3' UTR<br>3'-ugcagggaaacGGAAGGGUCg-5' miRNA<br>16 15 14 13 7 6 5 4 3 2<br>3'pairing Seed | 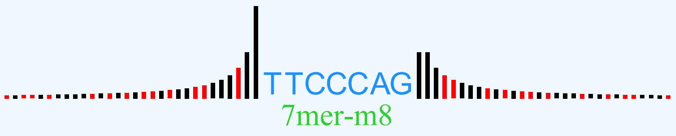 | 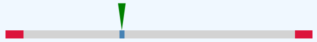 | | M T |

| 2D Structure | Local AU | Position | Conservation | Predicted By |
| --- | --- | --- | --- | --- |
| Offset 6mer<br>378 399<br>5'-gatgccGAAGAGCC-GGAGAAAa-3' UTR<br>3'-cucccuCUCUCGGUCCUCUUCg-5' miRNA<br>16 15 14 13 7 6 5 4 3 2<br>3'pairing Seed | 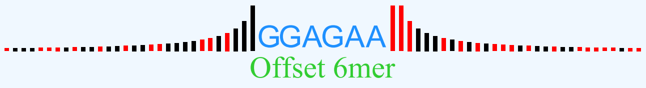 | 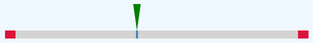 | | M |
| Imperfect match<br>432 454<br>5'-tccGTGGGAGTGCAAGTAGAAGa -3' UTR<br>3'-cucCCUCCUCUCGGUCCUCUUCg-5' miRNA<br>16 15 14 13 7 6 5 4 3 2<br>3'pairing Seed | 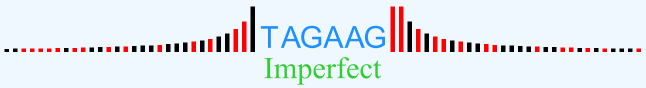 | 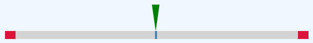 | | M |
| Offset 6mer<br>849 870<br>5'-gaGAGAGAAGAG-AGGGAGAACa-3' UTR<br>3'-cuCCCUCCUCUCGGUCCUCUUCg-5' miRNA<br>16 15 14 13 7 6 5 4 3 2<br>3'pairing Seed | 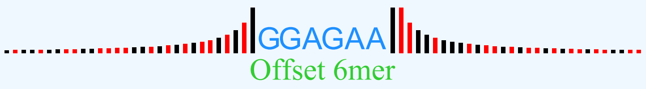 | 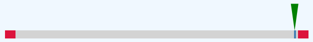 | | M |

| 2D Structure | Local AU | Position | Conservation | Predicted By |
|---|---|---|---|---|
| 7mer-m8; 689 5'-agtcacacAGAGCAGGGAGAAGg-3' UTR 711; 3'-cucccucCUCUCGGUCCUCUUCg-5' miRNA; 3'pairing Seed | GGAGAAG 7mer-m8 | | | M T |
| Imperfect match; 1696 5'-gagcaAGCAATCCCAGGGGAAGg -3' UTR 1718; 3'-cucccUCCUCUCGGUCCUCUUCg-5' miRNA; 3'pairing Seed | GGGAAG Imperfect | | | M |
| Imperfect match; 1782 5'-ttgcctGGACAGGAGGGAGAGGt -3' UTR 1804; 3'-cucccuCCUCUCGGUCCUCUUCg-5' miRNA; 3'pairing Seed | GAGAGG Imperfect | | | M |
| Offset 6mer; 2498 5'-atGGGCAAGGATAATTGGGAGAAtg-3' UTR 2522; 3'-cuCCC--UCCUCUCGGUCCUCUUcg-5' miRNA; 3'pairing Seed | GGAGAA Offset 6mer | | | M |
| Imperfect match; 5591 5'-ctgttGGGAGGG-AAGGAGAGGc -3' UTR 5612; 3'-cucccUCCUCUCGGUCCUCUUCg-5' miRNA; 3'pairing Seed | GAGAGG Imperfect | | | M |
| Imperfect match; 6842 5'-attcGAAGA-AGCCAGGAGAGGa -3' UTR 6863; 3'-cuccCUCCUCUCGGUCCUCUUCg-5' miRNA; 3'pairing Seed | GAGAGG Imperfect | | | M |
| Imperfect match; 7255 5'-agGGAAGCAGGGCAGGGGGGAGg -3' UTR 7277; 3'-cuCCCUCCUCUCGGUCCUCUUCg-5' miRNA; 3'pairing Seed | GGGGAG Imperfect | | | M |
| Imperfect match; 13447 5'-cttaGAGAAGAG-AGGGAGAGGt -3' UTR 13468; 3'-cuccCUCCUCUCGGUCCUCUUCg-5' miRNA; 3'pairing Seed | GAGAGG Imperfect | | | M |
| Imperfect match; 13990 5'-ttgtagccAGAGCTCGGGGAAGg -3' UTR 14012; 3'-cucccuccCUCUCGGUCCUCUUCg-5' miRNA; 3'pairing Seed | GGGAAG Imperfect | | | M |
| Offset 6mer; 17659 5'-agagactGAGTCTCAGGAGAAac-3' UTR 17681; 3'-cucccucCUCUCGGUCCUCUUcg-5' miRNA; 3'pairing Seed | GGAGAA Offset 6mer | | | M |
| Imperfect match; 18644 5'-atGGGAGGCAGAGACAGGAGGTGt -3' UTR 18667; 3'-cuCCCUCC-UCUCGGUCCUCUUCg-5' miRNA; 3'pairing Seed | GAGGTG Imperfect | | | M |
| Imperfect match; 20181 5'-attGGGGGAAGGAAACGGGGAAGa -3' UTR 20204; 3'-cucCCUCCUCUCGGU-CCUCUUCg-5' miRNA; 3'pairing Seed | GGGAAG Imperfect | | | M |
| 8mer; 21107 5'-gccaccGGATGGAT-GGAGAAGa-3' UTR 21128; 3'-cucccuCCUCUCGGUCCUCUUCg-5' miRNA; 3'pairing Seed | GGAGAAGA 8mer | | | M T |
| Imperfect match; 23114 5'-gtgcttGGAGCTTGAGGAGAGGg -3' UTR 23136; 3'-cucccuCCUCUCGGUCCUCUUCg-5' miRNA; 3'pairing Seed | GAGAGG Imperfect | | | M |
| Imperfect match; 23635 5'-actGTAGGGATTCCCGGAGGAGt -3' UTR 23657; 3'-cucCCUCCUCUCGGUCCUCUUCg-5' miRNA; 3'pairing Seed | GAGGAG Imperfect | | | M |
| Imperfect match; 25832 5'-ctGAGAGGTGGAGGCAGGAGGATc -3' UTR 25855; 3'-cuCCCUCC-UCUCGGUCCUCUUCg-5' miRNA; 3'pairing Seed | GAGGAT Imperfect | | | M |
| Imperfect match; 27565 5'-ctGGGAGTTGAACTCAGGAGTAGt -3' UTR 27588; 3'-cuCCCUCCUCUCG-GUCCUCUUCg-5' miRNA; 3'pairing Seed | GAGTAG Imperfect | | | M |
| 6mer; 28267 5'-ctaGGAAGAGCGTCTAGAGAAGg-3' UTR 28289; 3'-cucCCUCCUCUCGGUCCUCUUCg-5' miRNA; 3'pairing Seed | GAGAAG 6mer | | | M |
| Imperfect match; 30880 5'-agGGGAAGGGAGAGAGGGGAGAGGg -3' UTR 30904; 3'-cuCCCU--CCUCUCGGUCCUCUUCg-5' miRNA; 3'pairing Seed | GAGAGG Imperfect | | | M |
| Offset 6mer; 31357 5'-tgGGCATGAGGATCAGGGAGAAaa-3' UTR 31380; 3'-cuCCCUCCUCUCGGU-CCUCUUcg-5' miRNA; 3'pairing Seed | GGAGAA Offset 6mer | | | M |
| Imperfect match; 32388 5'-ggaGGGGGAGGG-GAGGAGGAGg -3' UTR 32409; 3'-cucCCUCCUCUCGGUCCUCUUCg-5' miRNA; 3'pairing Seed | GAGGAG Imperfect | | | M |
| Imperfect match; 33394 5'-ctgctctGGGAGTAGGGAGAGGa -3' UTR 33416; 3'-cucccucCUCUCGGUCCUCUUCg-5' miRNA; 3'pairing Seed | GAGAGG Imperfect | | | M |
| Offset 6mer; 41065 5'-aaggatcctGAGTCAGGAGAAcc-3' UTR 41087; 3'-cucccuccuCUCGGUCCUCUUcg-5' miRNA; 3'pairing Seed | GGAGAA Offset 6mer | | | M |

| 2D Structure | Local AU | Position | Conservation | Predicted By |
|---|---|---|---|---|
| Offset 6mer<br>25 5'-agttaAGCA-AGCCGGGAGAAct-3' UTR 46<br>3'-cucccUCCUCUCGGUCCUCUUcg-5' miRNA<br>16 15 14 13 / 7 6 5 4 3 2<br>3'pairing Seed | 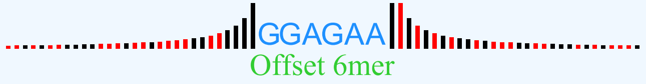 | 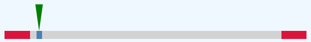 | | M |
| 7mer-m8<br>68 5'-gagaaAGCTGAGAGGGGAGAAGt-3' UTR 90<br>3'-cucccUCCUCUCGGUCCUCUUCg-5' miRNA<br>16 15 14 13 / 7 6 5 4 3 2<br>3'pairing Seed | 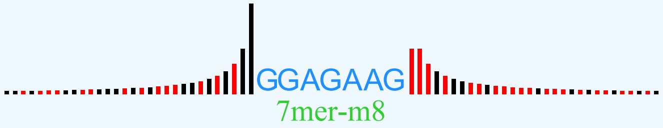 | 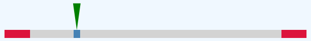 | | M T |

<table>
<tr><th>2D Structure</th><th>Local AU</th><th>Position</th><th>Conservation</th><th>Predicted By</th></tr>
<tr><td>40 8mer 62<br>5'-acgacaggCGGATACAAAAGCTa-3' UTR<br>||| : |||||||<br>3'-cauauuggGCCCG-GUUUUCGAg-5' miRNA<br>16 15 14 13 7 6 5 4 3 2<br>3'pairing Seed</td><td>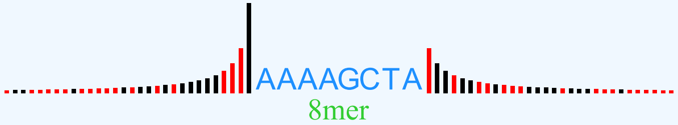</td><td>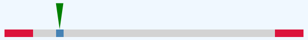</td><td></td><td>M T</td></tr>
<tr><td>233 7mer-m8 254<br>5'-ggctgcaaaGGTTCAAAAGCTg-3' UTR<br>|| : |||||||<br>3'-cauauugggCCCGGUUUUCGAg-5' miRNA<br>16 15 14 13 7 6 5 4 3 2<br>3'pairing Seed</td><td>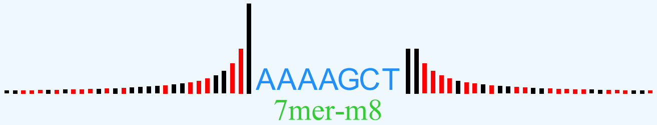</td><td>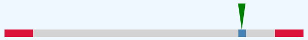</td><td></td><td>M T</td></tr>
</table>

<table>
<tr><th>2D Structure</th><th>Local AU</th><th>Position</th><th>Conservation</th><th>Predicted By</th></tr>
<tr><td>40 8mer 62<br>5'-acgacAGGCGGATACAAAAGCTa-3' UTR<br>| |||: :| |||||||<br>3'-uguauUGCGCUCGU-UUUCGAa-5' miRNA<br>16 15 14 13 7 6 5 4 3 2<br>3'pairing Seed</td><td>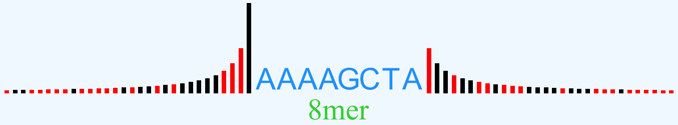
</td><td>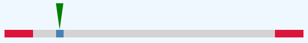</td><td>☐</td><td>M T</td></tr>
<tr><td>233 7mer-m8 254<br>5'-ggctgcaaaGGTTCAAAAGCTg-3' UTR<br>|: : |||||||<br>3'-uguauugcgCUCGUUUUUCGAa-5' miRNA<br>16 15 14 13 7 6 5 4 3 2<br>3'pairing Seed</td><td>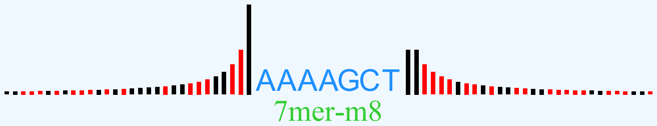
</td><td>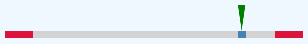</td><td>☐</td><td>M T</td></tr>
</table>

| 2D Structure | Local AU | Position | Conservation | Predicted By |
|---|---|---|---|---|
| 149 7mer-m8 169<br>5'-cctTCTGCGG-GGACAGTGGCt-3' UTR<br>3'-gucACACGCCACCCGUCACCGa-5' miRNA<br>16 15 14 13 7 6 5 4 3 2<br>3'pairing Seed | 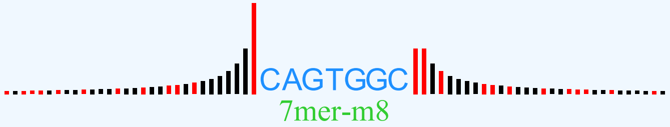 | 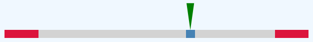 | | M T |

| 2D Structure | Local AU | Position | Conservation | Predicted By |
|---|---|---|---|---|
| 310 7mer-m8 332<br>5'-ttACGTTTACACGGCAGAGATTc-3' UTR<br>\|\|:\|\|\| \| \| \|\|\|\|\|\|\|\|\|<br>3'-agUGUAAACG-GACGUCUCUAAa-5' miRNA<br>16 15 14 13 7 6 5 4 3 2<br>3'pairing Seed | 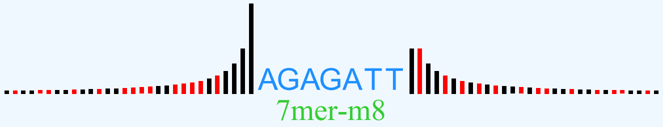 | 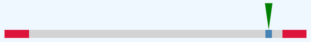 | | M T |

| 2D Structure | Local AU | Position | Conservation | Predicted By |
|---|---|---|---|---|
| 55 8mer 75<br>5'-tgtccagttctcaGACAATCa-3' UTR<br>\|\|\|\|\|\|\|<br>3'-ucuuaacgcaaacCUGUUAGu-5' miRNA<br>16 15 14 13 7 6 5 4 3 2<br>3'pairing Seed | 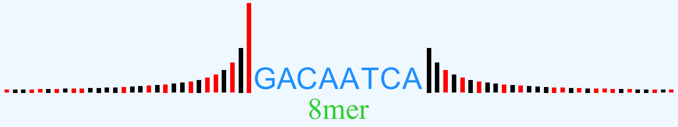 | 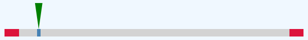 | ☐ | M T |
| Offset 6mer<br>171 193<br>5'-aaAATTGAGATTCGGGACAATgg-3' UTR<br>\|\|\|\|\| \| \|\| \|\|\|\|\|\|\|<br>3'-ucUUAACGCAAA--CCUGUUAGgu-5' miRNA<br>16 15 14 13 7 6 5 4 3 2<br>3'pairing Seed | 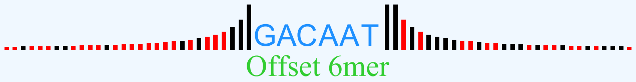 | 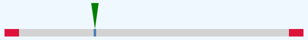 | ☐ | M |

<table>
<tr><th>2D Structure</th><th>Local AU</th><th>Position</th><th>Conservation</th><th>Predicted By</th></tr>
<tr><td>42 7mer-m8 63<br>5'-ttctttaagaTATGCTTTGATg-3' UTR<br>| ::|||||||<br>3'-ucucucccggAGGUGAAACUAc-5' miRNA<br>16 15 14 13 7 6 5 4 3 2<br>3'pairing Seed</td><td>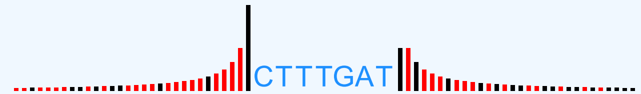<br>CTTTGAT<br>7mer-m8</td><td>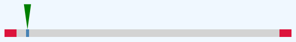</td><td></td><td>M T</td></tr>
<tr><td>99 7mer-m8 126<br>5'-ttgcAGGACTTCTTCAATGGCTTTGATg-3' UTR<br>|||| ||:|| |||||||<br>3'-ucucUCCCG--GAGGU----GAAACUAc-5' miRNA<br>16 15 14 13 7 6 5 4 3 2<br>3'pairing Seed</td><td>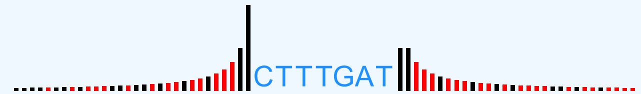<br>CTTTGAT<br>7mer-m8</td><td>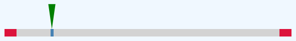</td><td></td><td>M T</td></tr>
<tr><td>330 7mer-m8 351<br>5'-aatGAGAAACTCTGCTTTGATg-3' UTR<br>||| |||::|||||||<br>3'-ucuCUCCCGGAGGUGAAACUAc-5' miRNA<br>16 15 14 13 7 6 5 4 3 2<br>3'pairing Seed</td><td>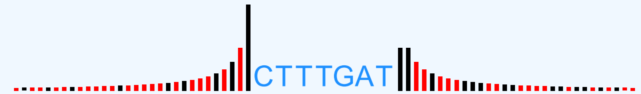<br>CTTTGAT<br>7mer-m8</td><td>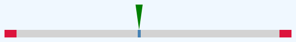</td><td></td><td>M T</td></tr>
<tr><td>620 8mer 642<br>5'-aatgattaatgCCACCTTTGATa-3' UTR<br>||| |||||||<br>3'-ucucucccggaGGU-GAAACUAc-5' miRNA<br>16 15 14 13 7 6 5 4 3 2<br>3'pairing Seed</td><td>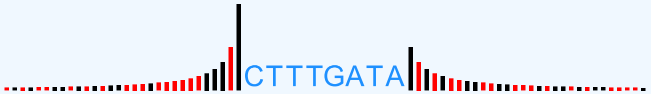<br>CTTTGATA<br>8mer</td><td>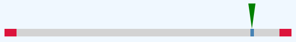</td><td></td><td>M T</td></tr>
</table>

<table>
<tr><th>2D Structure</th><th>Local AU</th><th>Position</th><th>Conservation</th><th>Predicted By</th></tr>
<tr><td>42 7mer-m8 63<br>5'-ttctttaagaTATGCTTTGATg-3' UTR<br>| ::|||||||<br>3'-ccucucccggAGGUGAAACUAg-5' miRNA<br>16 15 14 13 7 6 5 4 3 2<br>3'pairing Seed</td><td>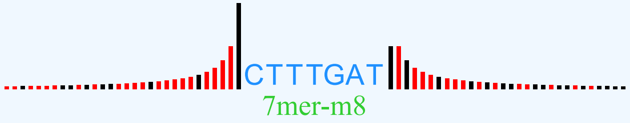</td><td>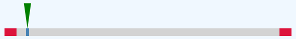</td><td>☐</td><td>M T</td></tr>
<tr><td>99 7mer-m8 126<br>5'-ttgcAGGACTTCTTCAATGGCTTTGATg-3' UTR<br>||| | ||:|| |||||||<br>3'-ccucUCCCG--GAGGU----GAAACUAg-5' miRNA<br>16 15 14 13 7 6 5 4 3 2<br>3'pairing Seed</td><td>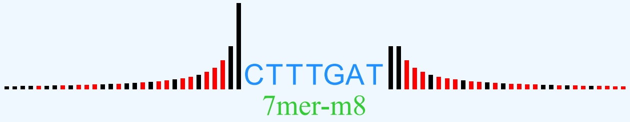</td><td>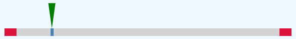</td><td>☐</td><td>M T</td></tr>
<tr><td>330 7mer-m8 351<br>5'-aatGAGAAACTCTGCTTTGATg-3' UTR<br>||| |||::|||||||<br>3'-ccuCUCCCGGAGGUGAAACUAg-5' miRNA<br>16 15 14 13 7 6 5 4 3 2<br>3'pairing Seed</td><td>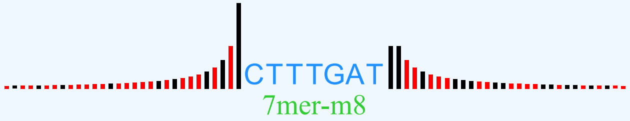</td><td>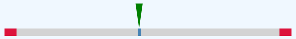</td><td>☐</td><td>M T</td></tr>
<tr><td>620 8mer 642<br>5'-aatgattaatgCCACCTTTGATa-3' UTR<br>||| |||||||<br>3'-ccucucccggaGGU-GAAACUAg-5' miRNA<br>16 15 14 13 7 6 5 4 3 2<br>3'pairing Seed</td><td>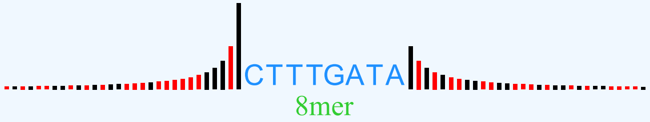</td><td>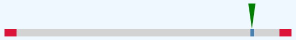</td><td>☐</td><td>M T</td></tr>
</table>

| 2D Structure | Local AU | Position | Conservation | Predicted By |
|---|---|---|---|---|
| 369 7mer-m8 390<br>5'-ccccttcaagaaTATTTTGAGt-3' UTR<br>:\|\|\|\|\|\|\|\|<br>3'-ucuaucccggagGUAAAACUCa-5' miRNA<br>16 15 14 13 7 6 5 4 3 2<br>3'pairing Seed | TTTTGAG<br>7mer-m8 | | | M T |
| 390 7mer-m8 411<br>5'-ttgattGATCATCATTTTGAGc-3' UTR<br>\| :\| :\|\|\|\|\|\|\|\|<br>3'-ucuaucCCGGAGGUAAAACUCa-5' miRNA<br>16 15 14 13 7 6 5 4 3 2<br>3'pairing Seed | TTTTGAG<br>7mer-m8 | | | M T |

| 2D Structure | Local AU | Position | Conservation | Predicted By |
|---|---|---|---|---|
| 8mer<br>306 326<br>5'-cactttgtcctGGGGGCCCa-3' UTR<br>\|\|\|\|\|\|\|\|<br>3'-uguccuaacuccCCCCGGGa-5' miRNA<br>16 15 14 13 7 6 5 4 3 2<br>3'pairing Seed | 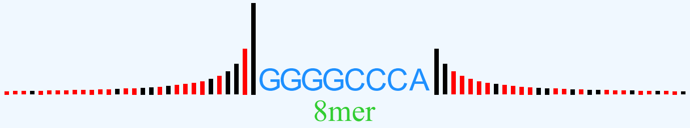 | 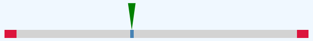 | | M T |
| Offset 6mer<br>704 724<br>5'-ccAGGAATGAGAGGGGGCCat-3' UTR<br>\|\|\|\| \|\|\|\| \|\|\|\|\|\|\|<br>3'-ugUCCUAACUCCCCCCCGGga-5' miRNA<br>16 15 14 13 7 6 5 4 3 2<br>3'pairing Seed | 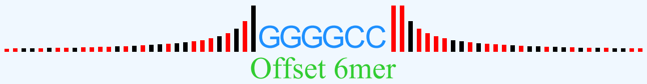 | 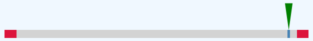 | | M |

| 2D Structure | Local AU | Position | Conservation | Predicted By |
|---|---|---|---|---|
| 346 7mer-m8 368<br>5'-catcttctgctTCATCCTCTGCc-3' UTR<br>:/ ///////<br>3'-cccuucuugucGGGAGGAGACGg-5' miRNA<br>16 15 14 13 7 6 5 4 3 2<br>3'pairing Seed | 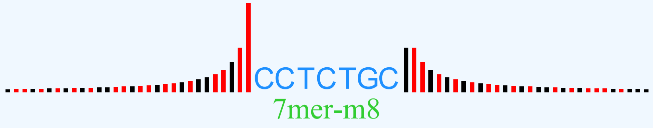 | 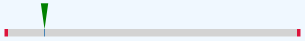 | | M T |
| 436 Offset 6mer 458<br>5'-caccttcACTGCTTTCCTCTGga-3' UTR<br>// //:://////<br>3'-cccuucuUGUCGGGAGGAGACgg-5' miRNA<br>16 15 14 13 7 6 5 4 3 2<br>3'pairing Seed | 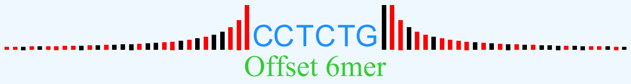 | 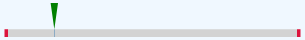 | | M |
| 585 8mer 607<br>5'-actgctAGCCTCATTCCTCTGCa-3' UTR<br>/:/ / :///////<br>3'-cccuucuUGUCGGGAGGAGACGg-5' miRNA<br>16 15 14 13 7 6 5 4 3 2<br>3'pairing Seed | 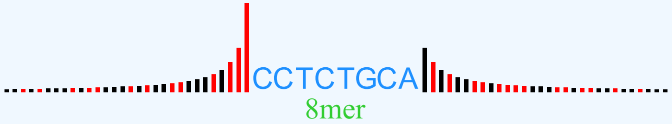 | 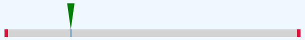 | | M T |
| 1887 7mer-A1 1910<br>5'-cagtgtcATAGCCATATCTCTGCa-3' UTR<br>/://// / ://////<br>3'-cccuucuUGUCGGGA-GGAGACGg-5' miRNA<br>16 15 14 13 7 6 5 4 3 2<br>3'pairing Seed | 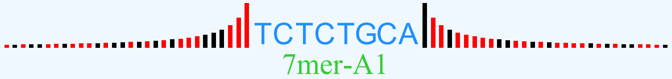 | 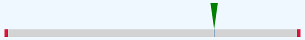 | | M T |

| 2D Structure | Local AU | Position | Conservation | Predicted By |
|---|---|---|---|---|
| Offset 6mer<br>94 119<br>5'-acaGAGGACGGCTTTACTCCTCTGgc-3' UTR<br>:\|\|:\|\|:\|\|: \|\|\|\|\|\|\|\|<br>3'-cccUUCUUGUCGG---GAGGAGACgg-5' miRNA<br>16 15 14 13 7 6 5 4 3 2<br>3'pairing Seed | 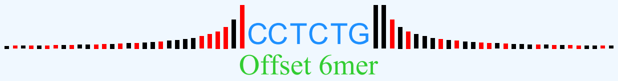 | 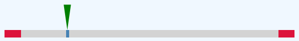 | ☐ | M |
| Imperfect match<br>304 329<br>5'-acaGAGAGTGGCTTCACTCCTTTGCa -3' UTR<br>:\|\|\|:::\|\|: \|\|\|\|\|:\|\|\|<br>3'-cccUUCUUGUCGG---GAGGAGACGg-5' miRNA<br>16 15 14 13 7 6 5 4 3 2<br>3'pairing Seed | 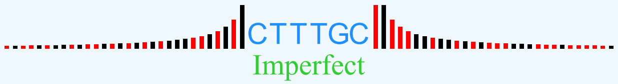 | 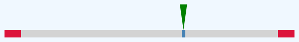 | ☐ | M |
| 8mer<br>406 428<br>5'-agGAATGGAATCACTCCTCTGCa-3' UTR<br>\|\|\| :: \| \| \|\|\|\|\|\|\|\|\|<br>3'-ccCUUCUUGUCGGGAGGAGACGg-5' miRNA<br>16 15 14 13 7 6 5 4 3 2<br>3'pairing Seed | 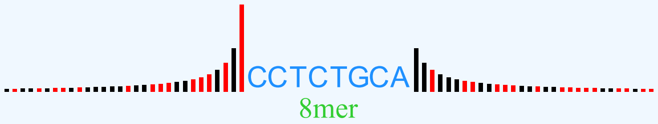 | 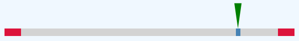 | ☐ | M T |

| 2D Structure | Local AU | Position | Conservation | Predicted By |
|---|---|---|---|---|
| Offset 6mer<br>300 5'-ctccATGTGGTGGATGGTAAAgt-3' UTR 322<br>3'-uacaUACAC--CCUGCCAUUUgg-5' miRNA<br>16 15 14 13 · 7 6 5 4 3 2<br>3'pairing · Seed | 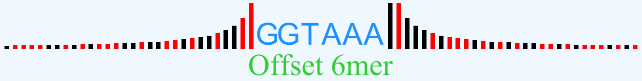 | 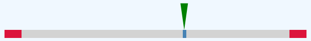 | ☐ | M |
| 7mer-m8<br>349 5'-cagaggGTGAAAAGGTAAACt-3' UTR 369<br>3'-uacauaCACCCUGCCAUUUGg-5' miRNA<br>16 15 14 13 · 7 6 5 4 3 2<br>3'pairing · Seed | 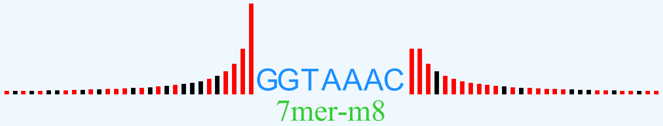 | 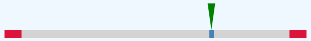 | ☐ | M T |

| 2D Structure | Local AU | Position | Conservation | Predicted By |
|---|---|---|---|---|
| Imperfect match<br>79 104<br>5'-atATTGCAAACACCCTGAAGTACTTt-3' UTR<br>3'-ugUGA-GUUUGU--ACCUUCGUGAAu-5' miRNA<br>16 15 14 13 7 6 5 4 3 2<br>3'pairing Seed | 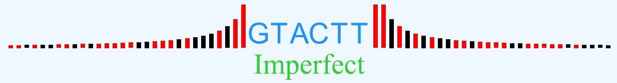 | 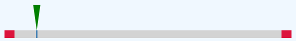 | | M |
| Imperfect match<br>103 127<br>5'-tttCTCAGACCTTGTTAAGCATTTt-3' UTR<br>3'-uguGAGUUUGUACC--UUCGUGAAu-5' miRNA<br>16 15 14 13 7 6 5 4 3 2<br>3'pairing Seed | 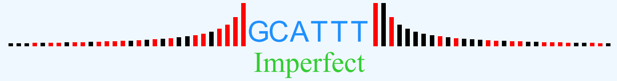 | 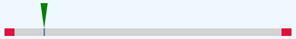 | | M |
| 7mer-m8<br>471 499<br>5'-tttCTCAATGATGATGTTCTGAGCACTTt-3' UTR<br>3'-uguGAGUUUGUAC--C----UUCGUGAAu-5' miRNA<br>16 15 14 13 7 6 5 4 3 2<br>3'pairing Seed | 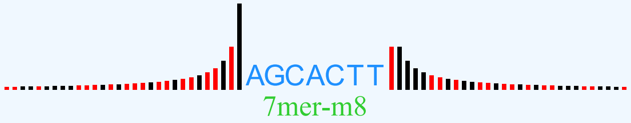 | 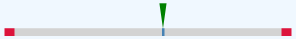 | | M T |

| 2D Structure | Local AU | Position | Conservation | Predicted By |
| --- | --- | --- | --- | --- |
| Offset 6mer<br>108 130<br>5'-agtAGAAATGGCCGAGAGAAGtg-3' UTR<br>3'-ccuUCUUGGCC-CUUCUCUUCcg-5' miRNA<br>16 15 14 13 7 6 5 4 3 2<br>3'pairing Seed | 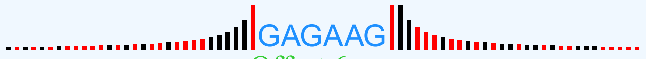 | 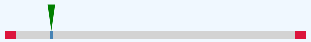 | | M |
| 7mer-m8<br>702 723<br>5'-atgtGACTCAGCTGGAGAAGGc-3' UTR<br>3'-ccuuCUUGGCCCUUCUCUUCCg-5' miRNA<br>16 15 14 13 7 6 5 4 3 2<br>3'pairing Seed | 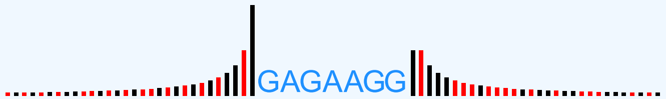 | 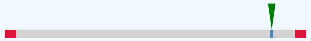 | | M T |

<table>
<tr><th>2D Structure</th><th>Local AU</th><th>Position</th><th>Conservation</th><th>Predicted By</th></tr>
<tr><td>108 7mer-m8 127<br>5'-ttcAGTACT--TAAGAGAAGGc-3' UTR<br>||  ||:   ||||||||<br>3'-ccuUCUUGGCCCUUCUCUUCCg-5' miRNA<br>16 15 14 13 7 6 5 4 3 2<br>3'pairing Seed</td><td>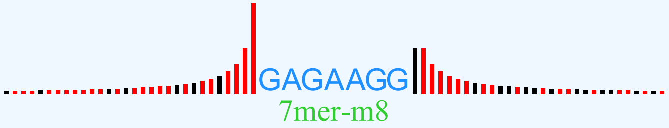
</td><td>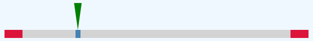</td><td></td><td>M T</td></tr>
<tr><td>Offset<br>318 6mer 339<br>5'-caAAGAAAAGTCACGAGAAGTa-3' UTR<br>|||||  |  |  ||||||<br>3'-ccUUCUUGGCCCUUCUCUUCcg-5' miRNA<br>16 15 14 13 7 6 5 4 3 2<br>3'pairing Seed</td><td>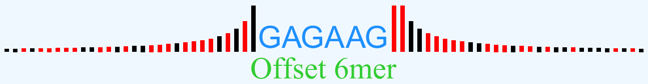
</td><td>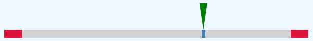</td><td></td><td>M</td></tr>
</table>

| 2D Structure | Local AU | Position | Conservation | Predicted By |
|---|---|---|---|---|
| 1088 7mer-m8 1108<br>5'-acCTAGGTA-GCACTGCTGCTt-3' UTR<br>3'-agGUUUUGUACUUAACGACGAc-5' miRNA<br>16 15 14 13 7 6 5 4 3 2<br>3'pairing Seed | TGCTGCT<br>7mer-m8 | | | M T |
| 1107 7mer-m8 1127<br>5'-ttCAACTCA-CCATTGCTGCTc-3' UTR<br>3'-agGUUUUGUACUUAACGACGAc-5' miRNA<br>16 15 14 13 7 6 5 4 3 2<br>3'pairing Seed | TGCTGCT<br>7mer-m8 | | | M T |
| 1897 7mer-m8 1918<br>5'-attttAATTTCTAATGCTGCTt-3' UTR<br>3'-agguuUUGUACUUAACGACGAc-5' miRNA<br>16 15 14 13 7 6 5 4 3 2<br>3'pairing Seed | TGCTGCT<br>7mer-m8 | | | M T |
| 2423 7mer-m8 2444<br>5'-gtggctcTGTCATATGCTGCTt-3' UTR<br>3'-agguuuuGUACUUAACGACGAc-5' miRNA<br>16 15 14 13 7 6 5 4 3 2<br>3'pairing Seed | TGCTGCT<br>7mer-m8 | | | M T |
| 4615 Offset 6mer 4636<br>5'-cagttgcctgGAATTGCTGCca-3' UTR<br>3'-agguuuuguaCUUAACGACGac-5' miRNA<br>16 15 14 13 7 6 5 4 3 2<br>3'pairing Seed | TGCTGC<br>Offset 6mer | | | M |

| 2D Structure | Local AU | Position | Conservation | Predicted By |
|---|---|---|---|---|
| 175 7mer-m8 196<br>5'-gtTGACACCTACCATGCTGCTg-3' UTR<br>3'-agGUUUUGUACUUAACGACGAc-5' miRNA<br>16 15 14 13 7 6 5 4 3 2<br>3'pairing Seed | 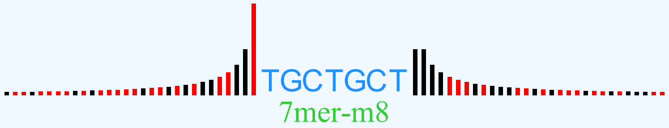 | 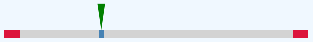 | | M T |
| 227 7mer-m8 247<br>5'-tttctGACAT-ATTTGCTGCTc-3' UTR<br>3'-agguuUUGUACUUAACGACGAc-5' miRNA<br>16 15 14 13 7 6 5 4 3 2<br>3'pairing Seed | 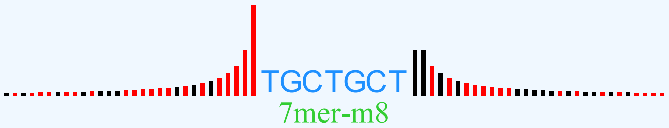 | 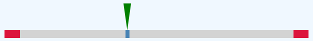 | | M T |

| 2D Structure | Local AU | Position | Conservation | Predicted By |
|---|---|---|---|---|
| 301 7mer-m8 322<br>5'-gtTGACACCTACCATGCTGCTg-3' UTR<br>::\| \|\| \| \|\|\|\|\|\|\|<br>3'-agGUUUUGUACUUAACGACGAc-5' miRNA<br>16 15 14 13 7 6 5 4 3 2<br>3'pairing Seed | 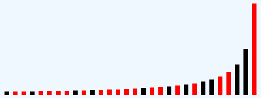<br>TGCTGCT<br>7mer-m8 | 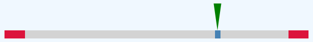 | | M T |
| 353 7mer-m8 373<br>5'-tttctGACAT-ATTTGCTGCTc-3' UTR<br>:\|\|\|\| \| \|\|\|\|\|\|\|\|<br>3'-agguuUUGUACUUAACGACGAc-5' miRNA<br>16 15 14 13 7 6 5 4 3 2<br>3'pairing Seed | 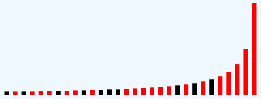<br>TGCTGCT<br>7mer-m8 | 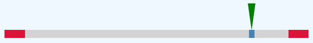 | | M T |

| 2D Structure | Local AU | Position | Conservation | Predicted By |
|---|---|---|---|---|
| Offset 6mer<br>800 ... 820<br>5'-taCCTTC---AAGGCTCTGCCCTt-3' UTR<br>\|\|\|\|\|   \|\|\|\|\|:\|\|\|\|\|\|:<br>3'-gcGGAAGUGUUUCCGGGACGGGGg-5' miRNA<br>16 15 14 13 ... 7 6 5 4 3 2<br>3'pairing ... Seed | CTGCCC<br>Offset 6mer | | ☐ | M |
| 7mer-A1<br>935 ... 958<br>5'-tgggctgtgAAAGATTTTGCCCCa-3' UTR<br>\|\|\|\| :::\|\|\|\|\|\|<br>3'-gcggaagugUUUCCGGGACGGGGg-5' miRNA<br>16 15 14 13 ... 7 6 5 4 3 2<br>3'pairing ... Seed | TTGCCCCA<br>7mer-A1 | | ☐ | M T |

<table>
<tr><th>2D Structure</th><th>Local AU</th><th>Position</th><th>Conservation</th><th>Predicted By</th></tr>
<tr><td>657 8mer 675<br>5'-gcCATTAAGATCCTCAAGa-3' UTR<br>| |:| | :|||||||<br>3'-uaGGAGUACGGGGAGUUCa-5' miRNA<br>16 15 14 13 7 6 5 4 3 2<br>3'pairing Seed</td><td>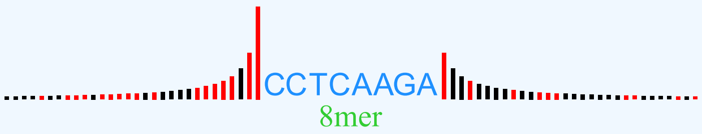
</td><td>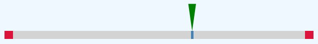</td><td></td><td>M T</td></tr>
<tr><td>861 7mer-m8 879<br>5'-agTCCCTTGCCCCTCAAGt-3' UTR<br>:| | |||||||||||<br>3'-uaGGAGUACGGGGAGUUCa-5' miRNA<br>16 15 14 13 7 6 5 4 3 2<br>3'pairing Seed</td><td>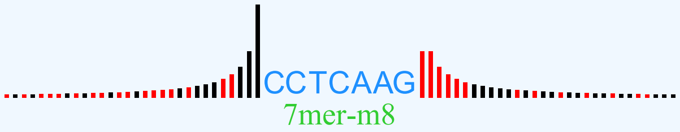
</td><td>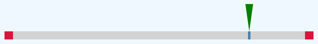</td><td></td><td>M T</td></tr>
</table>

<table>
<tr><th>2D Structure</th><th>Local AU</th><th>Position</th><th>Conservation</th><th>Predicted By</th></tr>
<tr><td><pre>293                     8mer    315
   5'-ctttAAGGAGGTGGCAGGGCCAa-3'  UTR
          |||| |  |: |||||||
   3'-ugccUUCC-CGUCUCUCCCGGUc-5'  miRNA
           16 15  14 13  7 6 5 4 3 2
        3'pairing       Seed</pre></td><td>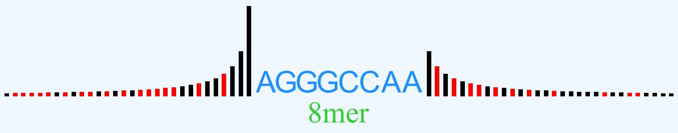
</td><td>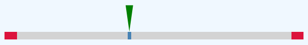</td><td></td><td>M T</td></tr>
<tr><td><pre>667                    7mer-m8  689
   5'-ctGGAAGAACAGCAAAGGGCCAc-3'  UTR
        |||||  ||| | |||||||
   3'-ugCCUUCCCGUC-UCUCCCGGUc-5'  miRNA
           16 15 14 13   7 6 5 4 3 2
        3'pairing       Seed</pre></td><td>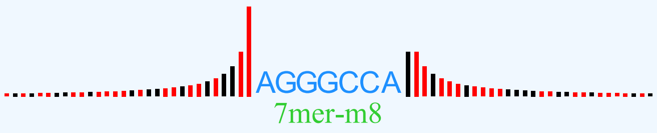
</td><td>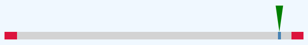</td><td></td><td>M T</td></tr>
</table>

| 2D Structure | Local AU | Position | Conservation | Predicted By |
|---|---|---|---|---|
| 421 7mer-m8 442<br>5'-catacatgaaTGGGAAACCTCt-3' UTR<br>::\|:\|\|\|\|\|\|\|<br>3'-uugucucuggGUCUUUUGGAGa-5' miRNA<br>16 15 14 13 7 6 5 4 3 2<br>3'pairing Seed | 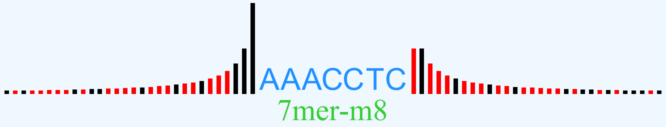<br> | 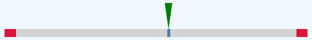 | | M T |
| Imperfect match<br>660 678<br>5'-ggCAGAGA--CAG-AGACCTCt-3' UTR<br>\|\|\|\|\|\| \|\|\| \|:\|\|\|\|\|<br>3'-uuGUCUCUGGGUCUUUUGGAGa-5' miRNA<br>16 15 14 13 7 6 5 4 3 2<br>3'pairing Seed | 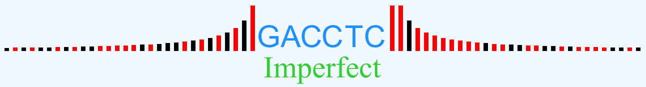<br> | 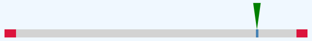 | | M |

| 2D Structure | Local AU | Position | Conservation | Predicted By |
| --- | --- | --- | --- | --- |
| 66 7mer-m8 86<br>5'-tccttgacctctTCCAGGGGc-3' UTR<br>:\|\|\|\|\|\|\|<br>3'-aagauccuauccGGGUCCCCg-5' miRNA<br>16 15 14 13 7 6 5 4 3 2<br>3'pairing Seed | 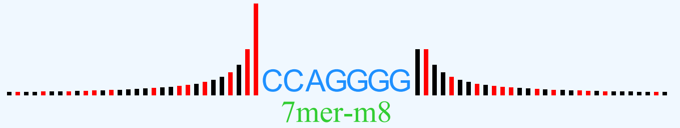 | | | M T |
| 237 8mer 257<br>5'-actgttGGTGGAACCAGGGGa-3' UTR<br>\|:\|:\| \|\|\|\|\|\|\|<br>3'-aagaucCUAUCCGGGUCCCCg-5' miRNA<br>16 15 14 13 7 6 5 4 3 2<br>3'pairing Seed | 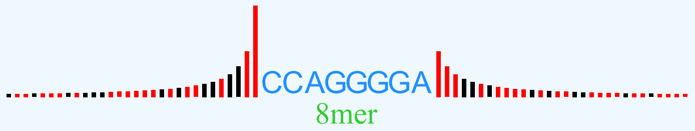 | | | M T |

| 2D Structure | Local AU | Position | Conservation | Predicted By |
|---|---|---|---|---|
| 16 8mer 38<br>5'-gaaCAGAATGAAGTAATGAAAAa-3' UTR<br>||| | | |:||||||||<br>3'-ccaGUCCU-CGUUAUUACUUUUu-5' miRNA<br>16 15 14 13 7 6 5 4 3 2<br>3'pairing Seed | 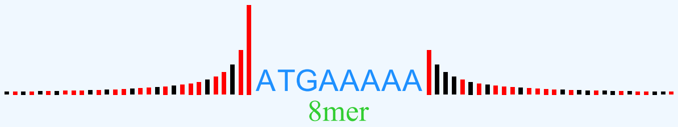 | 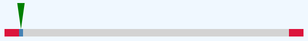 | | M T |
| Imperfect match<br>102 123<br>5'-tgTTAAAAGAAGTTATGAAGAt-3' UTR<br>|:| || |:| |||||:|<br>3'-ccAGUCCUCGUUAUUACUUUUu-5' miRNA<br>16 15 14 13 7 6 5 4 3 2<br>3'pairing Seed | 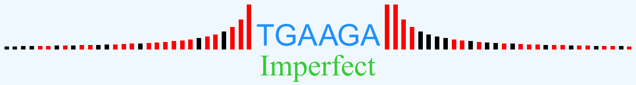 | 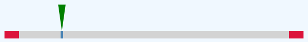 | | M |

<table>
<tr><th>2D Structure</th><th>Local AU</th><th>Position</th><th>Conservation</th><th>Predicted By</th></tr>
<tr><td>Imperfect match<br>203 224<br>5'-tacaAGAGGCAAAGATGAAGAg-3' UTR<br>|| :|||| :|||||:|<br>3'-ccagUCCUCGUUAUUACUUUUu-5' miRNA<br>16 15 14 13 7 6 5 4 3 2<br>3'pairing Seed</td><td>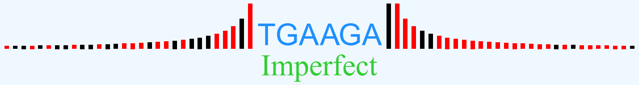<br></td><td>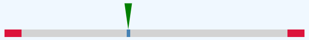</td><td></td><td>M</td></tr>
<tr><td>8mer<br>356 377<br>5'-tgaaAAGGTAAACTATGAAAAa-3' UTR<br>| |: || |||||||<br>3'-ccagUCCUCGUUAUUACUUUUu-5' miRNA<br>16 15 14 13 7 6 5 4 3 2<br>3'pairing Seed</td><td>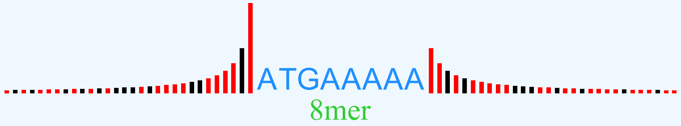<br></td><td>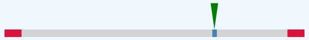</td><td></td><td>M T</td></tr>
</table>

| 2D Structure | Local AU | Position | Conservation | Predicted By |
|---|---|---|---|---|
| Imperfect match<br>347 368<br>5'-ccagatttcCAGTGATGAAGAg-3' UTR<br>\|\|:\|:\|\|\|\|\|:\|<br>3'-ccagucucGUUAUUACUUUUu-5' miRNA<br>16 15 14 13 7 6 5 4 3 2<br>3'pairing Seed | TGAAGA<br>Imperfect | | ☐ | M |
| Offset 6mer<br>743 764<br>5'-gcatgataaaAATAATGAAAtc-3' UTR<br>\|\|\|\|\|\|\|\|\|\|<br>3'-ccaguccucgUUAUUACUUUUuu-5' miRNA<br>16 15 14 13 7 6 5 4 3 2<br>3'pairing Seed | ATGAAA<br>Offset 6mer | | ☐ | M |
| Imperfect match<br>796 815<br>5'-tcgCA-GAGT-ATGATGAAGAg-3' UTR<br>\|\| \|\|\|: \|\|:\|\|\|\|\|:\|<br>3'-ccaGUCCUCGUUAUUACUUUUu-5' miRNA<br>16 15 14 13 7 6 5 4 3 2<br>3'pairing Seed | TGAAGA<br>Imperfect | | ☐ | M |

| 2D Structure | Local AU | Position | Conservation | Predicted By |
|---|---|---|---|---|
| Offset 6mer<br>155 175<br>5'-tgcaGCAACA-AGAGGAGCTtg-3' UTR<br>\|\|\| \|\| \| \|\|\|\|\|\|\|<br>3'-uuucCGUAGUAUAUCCUCGAcu-5' miRNA<br>16 15 14 13 7 6 5 4 3 2<br>3'pairing Seed | 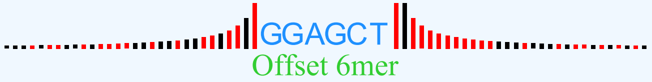 | 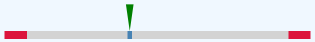 | ☐ | M |
| Imperfect match<br>233 253<br>5'-gaAGGCAGAGGAT-GGAGTTGc -3' UTR<br>\|\|\|\|\| : \|\| \|\|\|\|:\|\|<br>3'-uuUCCGUAGUAUAUCCUCGACu-5' miRNA<br>16 15 14 13 7 6 5 4 3 2<br>3'pairing Seed | 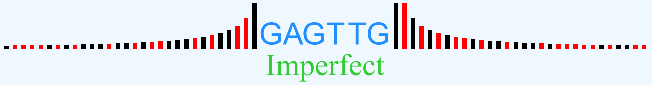 | 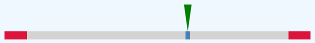 | ☐ | M |

| 2D Structure | Local AU | Position | Conservation | Predicted By |
| --- | --- | --- | --- | --- |
| 21 7mer-m8 41<br>5'-tgACAGTGTT-CTGATGCTGGc-3' UTR<br>\|\|\|: :\|: \|\|\|\|\|\|\|\|\|\|<br>3'-guUGUUUUAGUGACUACGACCu-5' miRNA<br>16 15 14 13 7 6 5 4 3 2<br>3'pairing Seed | 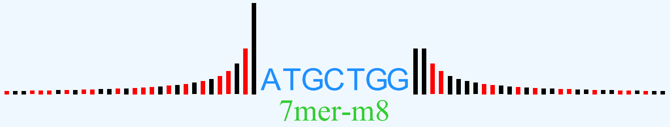 | 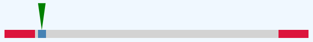 | 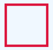 | M T |

| 2D Structure | Local AU | Position | Conservation | Predicted By |
|---|---|---|---|---|
| Imperfect match<br>40 62<br>5'-atttaATAATTGCAGGCCTGGCa -3' UTR<br>\|\|\|\|:::\| :\|\|\|\|:\|<br>3'-ccucaUAUUGGUGAUCGGACUGa-5' miRNA<br>16 15 14 13 7 6 5 4 3 2<br>3'pairing Seed | 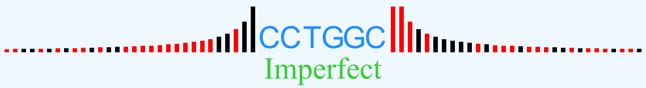 | 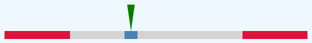 | | M |

<table>
<tr><th>2D Structure</th><th>Local AU</th><th>Position</th><th>Conservation</th><th>Predicted By</th></tr>
<tr><td>291 7mer-m8 310<br>5'-ggttttcgtggtGAAGCCTt-3' UTR<br>|||||||<br>3'-ccuuauaucgguCUUCGGAc-5' miRNA<br>16 15 14 13 7 6 5 4 3 2<br>3'pairing Seed</td><td>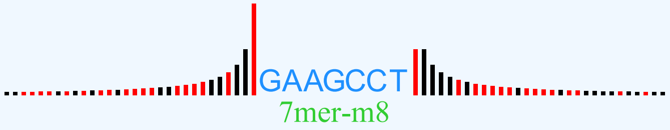</td><td>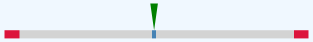</td><td></td><td>M T</td></tr>
<tr><td>402 7mer-m8 423<br>5'-cacATACTTTCTCAGAAGCCTt-3' UTR<br>||| | :|||||||||<br>3'-ccuUAU--AUCGGUCUUCGGAc-5' miRNA<br>16 15 14 13 7 6 5 4 3 2<br>3'pairing Seed</td><td>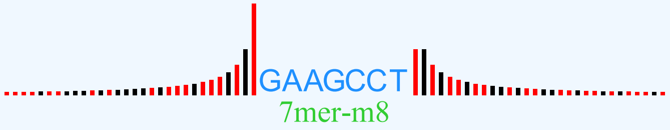</td><td>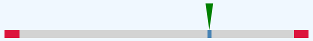</td><td></td><td>M T</td></tr>
</table>

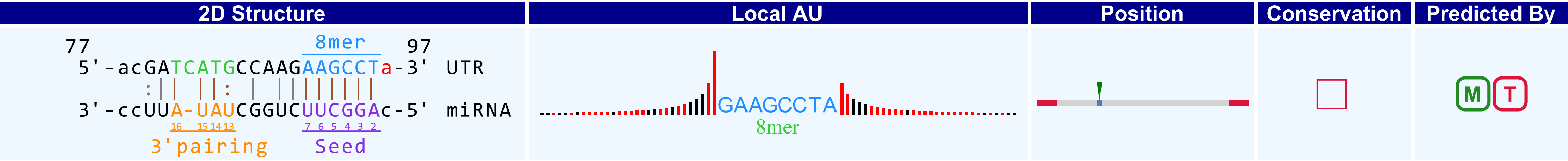
2D Structure
Local AU
Position
Conservation
Predicted By
77
8mer
97
5'-acGATCATGCCAAGAAGCCTa-3' UTR
3'-ccUUA-UAUCGGUCUUCGGAc-5' miRNA
16 15 14 13
7 6 5 4 3 2
3'pairing
Seed
GAAGCCTA
8mer
M
T

| 2D Structure | Local AU | Position | Conservation | Predicted By |
|---|---|---|---|---|
| 291 7mer-m8 310<br>5'-ggtttttcgtggtGAAGCCTt-3' UTR<br>\|\|\|\|\|\|\|<br>3'-ccuuauaucgguCUUCGGAc-5' miRNA<br>16 15 14 13 7 6 5 4 3 2<br>3'pairing Seed | 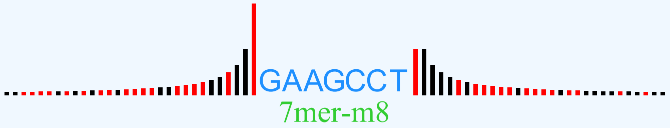 | 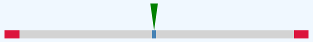 | ☐ | M T |
| 402 7mer-m8 423<br>5'-cacATACTTTCTCAGAAGCCTt-3' UTR<br>\|\|\| \| :\|\|\|\|\|\|\|\|\|<br>3'-ccuUAU--AUCGGUCUUCGGAc-5' miRNA<br>16 15 14 13 7 6 5 4 3 2<br>3'pairing Seed | 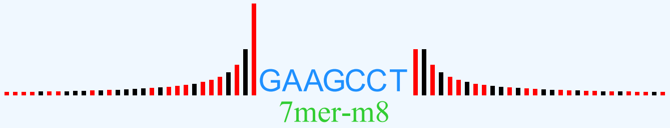 | 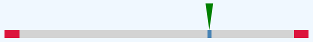 | ☐ | M T |

| 2D Structure | Local AU | Position | Conservation | Predicted By |
|---|---|---|---|---|
| 201 8mer 221<br>5'-ccttttTTAGAAGACCTGACTa-3' UTR<br>::|| || |||||||<br>3'-aggucGGUCCUC-GGACUGAa-5' miRNA<br>16 15 14 13 7 6 5 4 3 2<br>3'pairing Seed | 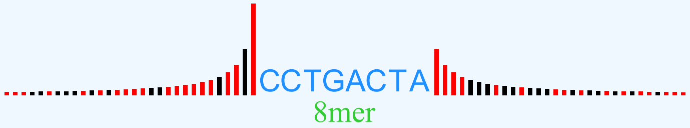 | 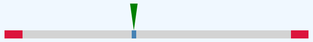 | | M T |
| 275 7mer-m8 293<br>5'-aactaCAAAGA-CCTGACTg-3' UTR<br>| | || |||||||<br>3'-aggucGGUCCUCGGACUGAa-5' miRNA<br>16 15 14 13 7 6 5 4 3 2<br>3'pairing Seed | 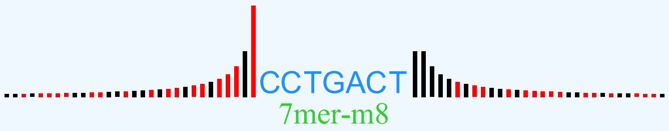 | 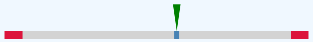 | | M T |

| 2D Structure | Local AU | Position | Conservation | Predicted By |
|---|---|---|---|---|
| 496 7mer-m8 519<br>5'-gaTTCAGAGACAAGACCCCTGCCc-3' UTR<br>3'-cgACGUC-CGGGUC-GGGGACGGa-5' miRNA<br>16 15 14 13 7 6 5 4 3 2<br>3'pairing Seed | CCCTGCC<br>7mer-m8 | | | M T |
| Offset 6mer<br>521 545<br>5'-acTGCAGCACCTGGACACCCTGCTg-3' UTR<br>3'-cgACGUC-CGGGUC-G-GGGACGGa-5' miRNA<br>16 15 14 13 7 6 5 4 3 2<br>3'pairing Seed | CCCTGC<br>Offset 6mer | | | M |

| 2D Structure | Local AU | Position | Conservation | Predicted By |
|---|---|---|---|---|

```
303                    7mer-m8  327
   5'-ggcaGTGCCTTTGTTCACAAGGATt-3'  UTR
          :::|||   ||||  |||||||
   3'-uaagUGUGGAUCCAAG-GUUCCUAa-5'  miRNA
              16 15 14 13    7 6 5 4 3 2
            3'pairing        Seed
```

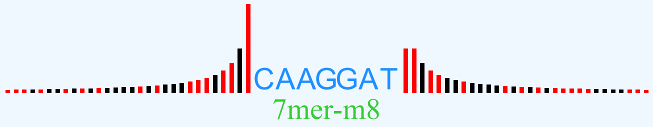


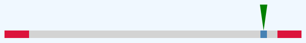

M T

| 2D Structure | Local AU | Position | Conservation | Predicted By |
|---|---|---|---|---|
| see below | 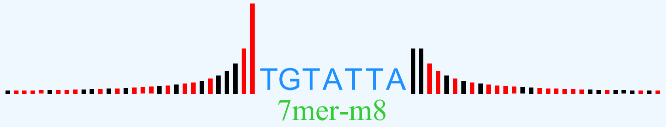 | 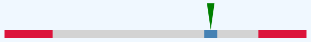 | | M T |



```
112                 7mer-m8  133
   5'-aaCTCTA-CAGGGATTGTATTAc-3'  UTR
           || ||  ||||  ||||||||
   3'-guGA-AUCGUCC--AACAUAAUa-5'  miRNA
          16 15 14 13   7 6 5 4 3 2
        3'pairing       Seed
```

| 2D Structure | Local AU | Position | Conservation | Predicted By |
|---|---|---|---|---|
| 73 8mer 94<br>5'-accaagGCCAAGCTCAACCTCa-3' UTR<br>:\|\|\|\|\|  \|\|\|\|\|\|\|<br>3'-cuuaagUGGUUCCCGUUGGAGa-5' miRNA<br>16 15 14 13 7 6 5 4 3 2<br>3'pairing Seed | 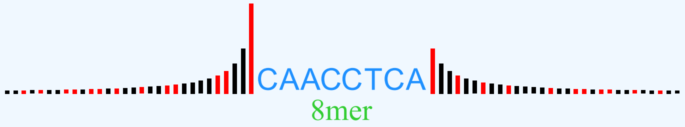 | 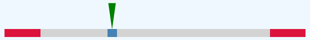 | 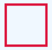 | 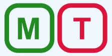 |

| 2D Structure | Local AU | Position | Conservation | Predicted By |
|---|---|---|---|---|
| 106 7mer-m8 126<br>5'-gcCAAGTATT-TGTTCAACCAg-3' UTR<br>\|\| \|\| :\| \|\| \|\|\|\|\|\|\|<br>3'-gcGUACAAGAUACCAGUUGGUa-5' miRNA<br>16 15 14 13 7 6 5 4 3 2<br>3'pairing Seed | 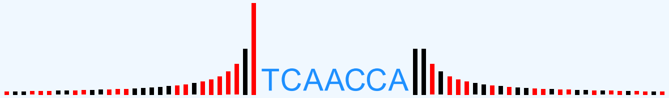<br> | 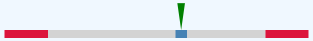 | | M T |

<table>
<tr><th>2D Structure</th><th>Local AU</th><th>Position</th><th>Conservation</th><th>Predicted By</th></tr>
<tr><td>68 8mer 90<br>5'-atGTGCATTG-TGAATCTCCAAGa-3' UTR<br>|: ||:|| | | |||||||<br>3'-gaCGGGUGACUAGAU-GAGGUUCu-5' miRNA<br>16 15 14 13 7 6 5 4 3 2<br>3'pairing Seed</td><td>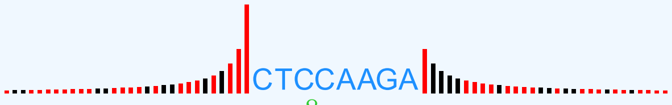</td><td>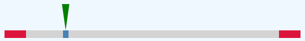</td><td></td><td>M T</td></tr>
<tr><td>346 7mer-m8 368<br>5'-aaacagcTTCATGAACTCCAAGc-3' UTR<br>:| || |||||||<br>3'-gacggguGACUAGAUGAGGUUCu-5' miRNA<br>16 15 14 13 7 6 5 4 3 2<br>3'pairing Seed</td><td>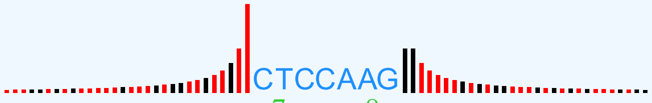</td><td>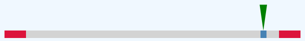</td><td></td><td>M T</td></tr>
</table>

<table>
<tr><th>2D Structure</th><th>Local AU</th><th>Position</th><th>Conservation</th><th>Predicted By</th></tr>
<tr><td>Imperfect match<br>55 75<br>5'-ctttaATATGCTA-ATGTGCAt-3' UTR<br>|:|| ||| ||:||||<br>3'-uacccUGUAGGAUGUAUACGUu-5' miRNA<br>16 15 14 13 7 6 5 4 3 2<br>3'pairing Seed</td><td>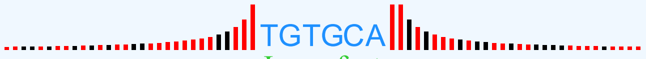</td><td>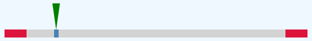</td><td></td><td>M</td></tr>
<tr><td>7mer-m8<br>91 108<br>5'-tgGGGATAT----GATATGCAg-3' UTR<br>||||:|| |||||||<br>3'-uaCCCUGUAGGAUGUAUACGUu-5' miRNA<br>16 15 14 13 7 6 5 4 3 2<br>3'pairing Seed</td><td>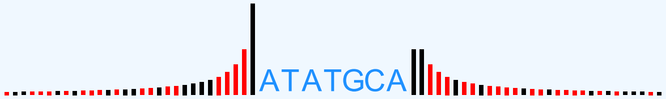</td><td>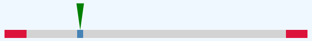</td><td></td><td>M T</td></tr>
</table>

| 2D Structure | Local AU | Position | Conservation | Predicted By |
| --- | --- | --- | --- | --- |
| 153 8mer 174<br>5'-cagctTGGAACAAGTTCCCAAa-3' UTR<br>3'-uaaauACGUUUUACAAGGGUUa-5' miRNA<br>16 15 14 13 7 6 5 4 3 2<br>3'pairing Seed | 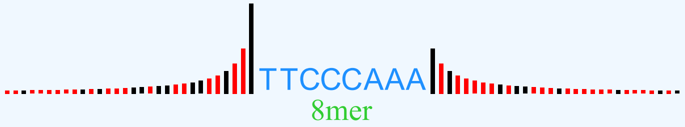 | 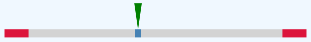 | | M T |

| 2D Structure | Local AU | Position | Conservation | Predicted By |
|---|---|---|---|---|
| 171 6mer 192<br>5'-tgATTATGAGTATGACGCAAAg-3' UTR<br>\|\|\|\|  \|\|::\|::  \|\|\|\|\|\|<br>3'-uaUAAUCCUUGUGUAGCGUUUu-5' miRNA<br>16 15 14 13 7 6 5 4 3 2<br>3'pairing Seed | 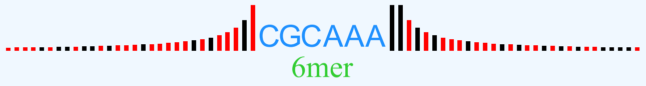  | 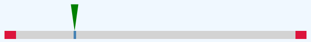 | | M |
| 312 7mer-m8 334<br>5'-tgtcaAGGAGGTCACTCGCAAAg-3' UTR<br>\|\|\|\|:  \|\|  \|\|\|\|\|\|\|<br>3'-uauaaUCCUUGUGU-AGCGUUUu-5' miRNA<br>16 15 14 13 7 6 5 4 3 2<br>3'pairing Seed | 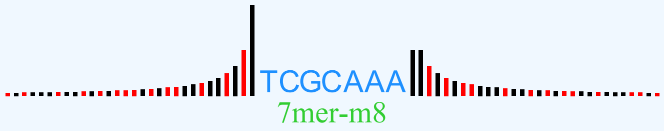  | 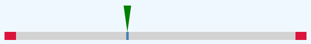 | | M T |

| 2D Structure | Local AU | Position | Conservation | Predicted By |
|---|---|---|---|---|
| 153 8mer 174<br>5'-caGCTTGGAACAAGTTCCCAAa-3' UTR<br>3'-uaCGUACGUUUUACAAGGGUUa-5' miRNA<br>16 15 14 13 7 6 5 4 3 2<br>3'pairing Seed | 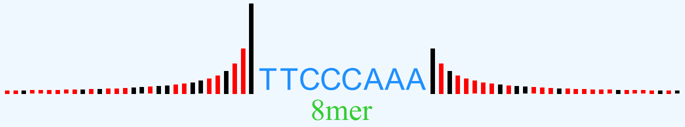 | 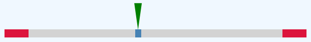 | | M T |

<table>
<tr><th>2D Structure</th><th>Local AU</th><th>Position</th><th>Conservation</th><th>Predicted By</th></tr>
<tr><td>540 8mer 563<br>5'-gtcgCAGCTGCTGAGACTGCAAAa-3' UTR<br>||| :| | |||||||<br>3'-auaaGUCCUUG--UAUGACGUUUu-5' miRNA<br>16 15 14 13 7 6 5 4 3 2<br>3'pairing Seed</td><td>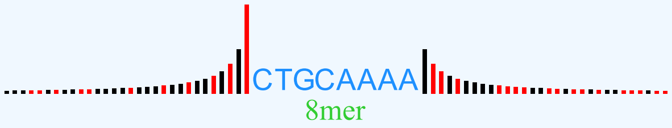
</td><td>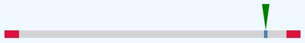</td><td>☐</td><td>M T</td></tr>
<tr><td>571 7mer-m8 591<br>5'-ccaaatGCAACAT-CTGCAAAg-3' UTR<br>| ||||| |||||||<br>3'-auaaguCCUUGUAUGACGUUUu-5' miRNA<br>16 15 14 13 7 6 5 4 3 2<br>3'pairing Seed</td><td>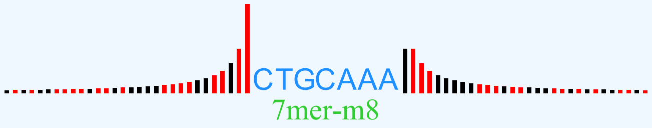
</td><td>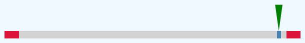</td><td>☐</td><td>M T</td></tr>
</table>

| 2D Structure | Local AU | Position | Conservation | Predicted By |
|---|---|---|---|---|
| 127 7mer-m8 146<br>5'-tttTCTTTGTCC-TGGACTGc-3' UTR<br>\| \| \|\|:\|\| \|\|\|\|\|\|\|<br>3'-cacAUAUACGGGCACCUGACg-5' miRNA<br>16 15 14 13 7 6 5 4 3 2<br>3'pairing Seed | TGGACTG<br>7mer-m8 | | | M T |
| Offset 6mer<br>239 258<br>5'-tacTATATGCCC-TGGACTcc-3' UTR<br>\|\|\|\|\|\|\|\|\| \|\|\|\|\|\|<br>3'-cacAUAUACGGGCACCUGACg-5' miRNA<br>16 15 14 13 7 6 5 4 3 2<br>3'pairing Seed | TGGACT<br>Offset 6mer | | | M |

| 2D Structure | Local AU | Position | Conservation | Predicted By |
|---|---|---|---|---|
| 207 7mer-m8 233<br>5'-atcTATTTGTTCCAGTTTTTCTAAATt-3' UTR<br>3'-aguGUAGUCACGGU----AAGAUUUAu-5' miRNA<br>16 15 14 13 7 6 5 4 3 2<br>3'pairing Seed | 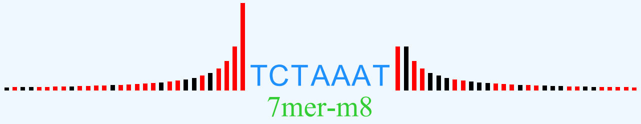  | 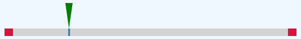 | | M T |
| 662 7mer-m8 683<br>5'-ttAC-TTATGGATTTTCTAAATg-3' UTR<br>3'-agUGUAGUCACGGUAAGAUUUAu-5' miRNA<br>16 15 14 13 7 6 5 4 3 2<br>3'pairing Seed | 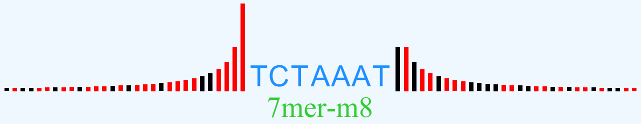  | 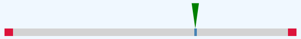 | | M T |

<table>
<tr><th>2D Structure</th><th>Local AU</th><th>Position</th><th>Conservation</th><th>Predicted By</th></tr>
<tr><td>207 7mer-m8 233<br>5'-atcTATTTGTTCCAGTTTTTCTAAATt-3' UTR<br>:||: || ||| ||||||||<br>3'-aguGUAGUCAUGGU----AAGAUUUAu-5' miRNA<br>16 15 14 13 7 6 5 4 3 2<br>3'pairing Seed</td><td>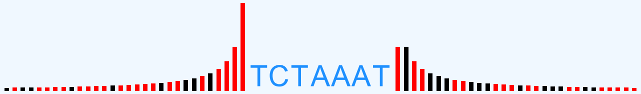<br>TCTAAAT<br>7mer-m8</td><td>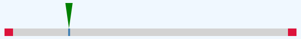</td><td></td><td>M T</td></tr>
<tr><td>662 7mer-m8 683<br>5'-ttACTTATGGATT-TTCTAAATg-3' UTR<br>|| | | |:: ||||||||<br>3'-agUGUAGUCAUGGUAAGAUUUAu-5' miRNA<br>16 15 14 13 7 6 5 4 3 2<br>3'pairing Seed</td><td>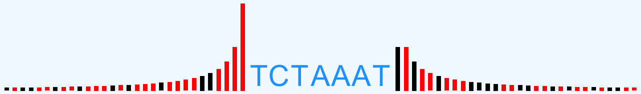<br>TCTAAAT<br>7mer-m8</td><td>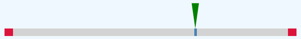</td><td></td><td>M T</td></tr>
</table>

<table>
<tr><th>2D Structure</th><th>Local AU</th><th>Position</th><th>Conservation</th><th>Predicted By</th></tr>
<tr><td>Imperfect match<br>2610 2630<br>5'-ttTTTAC-TTTGTGCATACATt-3' UTR<br>| ||| | |:::||:||||<br>3'-auACAUGUACAUGUGUGUGUAu-5' miRNA<br>16 15 14 13 7 6 5 4 3 2<br>3'pairing Seed</td><td>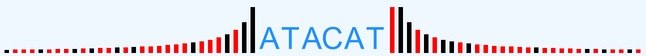</td><td>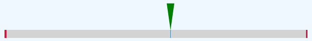</td><td></td><td>M</td></tr>
<tr><td>Offset 6mer<br>2860 2880<br>5'-agTCTCCAT-TACCCACACAgt-3' UTR<br>| | ||| ||| ||||||<br>3'-auACAUGUACAUGUGUGUGUau-5' miRNA<br>16 15 14 13 7 6 5 4 3 2<br>3'pairing Seed</td><td>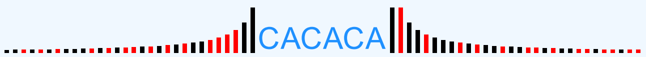</td><td>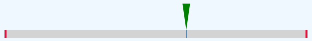</td><td></td><td>M</td></tr>
<tr><td>Offset 6mer<br>2946 2967<br>5'-caTGTGTGTGCTCACACACAaa-3' UTR<br>|||:::|| |||||||<br>3'-auACAUGUACAUGUGUGUGUau-5' miRNA<br>16 15 14 13 7 6 5 4 3 2<br>3'pairing Seed</td><td>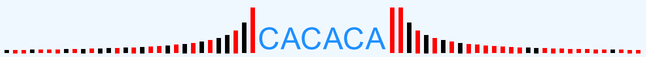</td><td>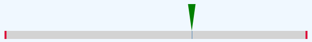</td><td></td><td>M</td></tr>
<tr><td>Offset 6mer<br>2956 2977<br>5'-ctcacACACAAACACACACAaa-3' UTR<br>||| ||||||||<br>3'-auacaUGUACAUGUGUGUGUau-5' miRNA<br>16 15 14 13 7 6 5 4 3 2<br>3'pairing Seed</td><td>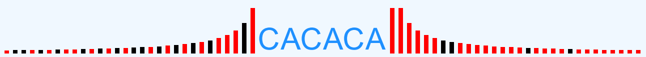</td><td>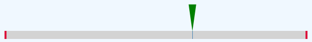</td><td></td><td>M</td></tr>
<tr><td>7mer-m8<br>4375 4395<br>5'-aaaGTGC-TTTTCTCACACATg-3' UTR<br>||:| | | | ||||||<br>3'-auaCAUGUACAUGUGUGUGUAu-5' miRNA<br>16 15 14 13 7 6 5 4 3 2<br>3'pairing Seed</td><td>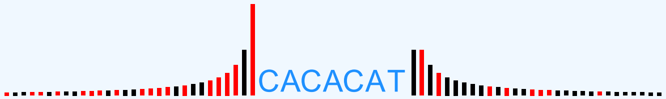</td><td>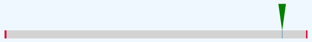</td><td></td><td>M T</td></tr>
</table>

| 2D Structure | Local AU | Position | Conservation | Predicted By |
|---|---|---|---|---|
| Offset 6mer<br>172 5'-agTGTGCGCATGCGCACACAga-3' UTR 193<br>3'-auACAUGUACAUGUGUGUGUAu-5' miRNA<br>3'pairing Seed | CACACA Offset 6mer | | | M |
| Imperfect match<br>951 5'-cagcctCTTGGCCACACGCATc-3' UTR 972<br>3'-auacauGUACAUGUGUGUGUAu-5' miRNA<br>3'pairing Seed | ACGCAT Imperfect | | | M |
| Imperfect match<br>5167 5'-tgTGTAGCACGGTGTTCATACATg-3' UTR 5190<br>3'-auACAU-GU-ACAUGUGUGUGUAu-5' miRNA<br>3'pairing Seed | ATACAT Imperfect | | | M |
| Imperfect match<br>5499 5'-caTG-ACAATGTAAACATACATa-3' UTR 5520<br>3'-auACAUG-UACAUGUGUGUGUAu-5' miRNA<br>3'pairing Seed | ATACAT Imperfect | | | M |
| Imperfect match<br>6811 5'-tgTGTGGGAGCACACGCACATg-3' UTR 6832<br>3'-auACAUGUACAUGUGUGUGUAu-5' miRNA<br>3'pairing Seed | GCACAT Imperfect | | | M |
| Offset 6mer<br>8363 5'-ccgccACACACACACACACAca-3' UTR 8384<br>3'-auacaUGUACAUGUGUGUGUau-5' miRNA<br>3'pairing Seed | CACACA Offset 6mer | | | M |
| Offset 6mer<br>11629 5'-atcacaATATGTGCAGCACACAgg-3' UTR 11651<br>3'-auacaUGUACAUGU-GUGUGUau-5' miRNA<br>3'pairing Seed | CACACA Offset 6mer | | | M |
| Imperfect match<br>11839 5'-caTGTATAT-TACACCACAAATa-3' UTR 11860<br>3'-auACAUGUACAUGU-GUGUGUAu-5' miRNA<br>3'pairing Seed | ACAAAT Imperfect | | | M |
| Imperfect match<br>12035 5'-tgTATATATGTGCACCACATGTg-3' UTR 12057<br>3'-auACAUGUACAUGU-GUGUGUAu-5' miRNA<br>3'pairing Seed | ACATGT Imperfect | | | M |
| 8mer<br>12727 5'-tgTATATACTCTCTCTCACACATa-3' UTR 12750<br>3'-auACAUGU-ACA-UGUGUGUGUAu-5' miRNA<br>3'pairing Seed | CACACATA 8mer | | | M T |
| Imperfect match<br>12839 5'-aaaagATGTGTGGTCACATATc-3' UTR 12860<br>3'-auacaUGUACAUGUGUGUGUAu-5' miRNA<br>3'pairing Seed | ACATAT Imperfect | | | M |
| Offset 6mer<br>14872 5'-taTGTACGTACACACCACACACa-3' UTR 14894<br>3'-auACAUGUACAUGU-GUGUGUau-5' miRNA<br>3'pairing Seed | CACACA Offset 6mer | | | M |
| Imperfect match<br>15001 5'-tgTATATATATATACATACATa-3' UTR 15022<br>3'-auACAUGUACAUGUGUGUGUAu-5' miRNA<br>3'pairing Seed | ATACAT Imperfect | | | M |
| 6mer<br>15021 5'-tacaTATATATATATACACATt-3' UTR 15042<br>3'-auacAUGUACAUGUGUGUGUAu-5' miRNA<br>3'pairing Seed | ACACAT 6mer | | | M |
| Offset 6mer<br>18766 5'-caccatggcGTGCACACACAct-3' UTR 18787<br>3'-auacauguaCAUGUGUGUGUau-5' miRNA<br>3'pairing Seed | CACACA Offset 6mer | | | M |
| Offset 6mer<br>18778 5'-cacacACACTCACACACACAca-3' UTR 18799<br>3'-auacaUGUACAUGUGUGUGUau-5' miRNA<br>3'pairing Seed | CACACA Offset 6mer | | | M |
| Offset 6mer<br>26313 5'-cacacAGAAGCACACACACAgc-3' UTR 26334<br>3'-auacaUGUACAUGUGUGUGUau-5' miRNA<br>3'pairing Seed | CACACA Offset 6mer | | | M |
| Offset 6mer<br>30100 5'-atatcACACCTAGACACACAgg-3' UTR 30121<br>3'-auacaUGUACAUGUGUGUGUau-5' miRNA<br>3'pairing Seed | CACACA Offset 6mer | | | M |
| 7mer-m8<br>33689 5'-tagacACATGTATGCACACACATg-3' UTR 33712<br>3'-auacaUGUACAU--GUGUGUGUAu-5' miRNA<br>3'pairing Seed | CACACAT 7mer-m8 | | | M T |
| Offset 6mer<br>33703 5'-cacacACATGTACACACACAca-3' UTR 33724<br>3'-auacaUGUACAUGUGUGUGUau-5' miRNA<br>3'pairing Seed | CACACA Offset 6mer | | | M |
| Offset 6mer<br>33719 5'-cacacACACACACACACACAca-3' UTR 33740<br>3'-auacaUGUACAUGUGUGUGUau-5' miRNA<br>3'pairing Seed | CACACA Offset 6mer | | | M |
| Imperfect match<br>35699 5'-caTATATACATATACACATATa-3' UTR 35720<br>3'-auACAUGUACAUGUGUGUGUAu-5' miRNA<br>3'pairing Seed | ACATAT Imperfect | | | M |
| Imperfect match<br>35715 5'-caTATATATTTACACACACTTa-3' UTR 35736<br>3'-auACAUGUACAUGUGUGUGUAu-5' miRNA<br>3'pairing Seed | ACACTT Imperfect | | | M |
| Imperfect match<br>35735 5'-tatacATATGCATAAACATACATa-3' UTR 35758<br>3'-auacaUGUAC--AUGUGUGUGUAu-5' miRNA<br>3'pairing Seed | ATACAT Imperfect | | | M |
| Imperfect match<br>36463 5'-agTGTACTCACGTATTCACATATa-3' UTR 36486<br>3'-auACAU--GUACAUGUGUGUGUAu-5' miRNA<br>3'pairing Seed | ACATAT Imperfect | | | M |

| 2D Structure | Local AU | Position | Conservation | Predicted By |
|---|---|---|---|---|
| Imperfect match<br>2610 2630<br>5'-ttTTTAC-TTTGTGCATACATt-3' UTR<br>3'-auACAUGUACAUGUGUGUGUAg-5' miRNA<br>16 15 14 13 7 6 5 4 3 2<br>3'pairing Seed | ATACAT<br>Imperfect | | | M |
| Offset 6mer<br>2860 2880<br>5'-agTCTCCAT-TACCCACACAgt-3' UTR<br>3'-auACAUGUACAUGUGUGUGUag-5' miRNA<br>16 15 14 13 7 6 5 4 3 2<br>3'pairing Seed | CACACA<br>Offset 6mer | | | M |
| Offset 6mer<br>2946 2967<br>5'-caTGTGTGTGCTCACACACAaa-3' UTR<br>3'-auACAUGUACAUGUGUGUGUag-5' miRNA<br>16 15 14 13 7 6 5 4 3 2<br>3'pairing Seed | CACACA<br>Offset 6mer | | | M |
| Offset 6mer<br>2956 2977<br>5'-ctcacACACAAACACACACAaa-3' UTR<br>3'-auacaUGUACAUGUGUGUGUag-5' miRNA<br>16 15 14 13 7 6 5 4 3 2<br>3'pairing Seed | CACACA<br>Offset 6mer | | | M |
| 7mer-m8<br>4375 4395<br>5'-aaaGTGC-TTTTCTCACACATg-3' UTR<br>3'-auaCAUGUACAUGUGUGUGUAg-5' miRNA<br>16 15 14 13 7 6 5 4 3 2<br>3'pairing Seed | CACACAT<br>7mer-m8 | | | M T |

| 2D Structure | Local AU | Position | Conservation | Predicted By |
|---|---|---|---|---|
| Offset 6mer; 172 5'-agTGTGCGCATGCGCACACAga-3' UTR 193; 3'-auACAUGUACAUGUGUGUGUAg-5' miRNA; 3'pairing Seed | CACACA Offset 6mer | | | M |
| Imperfect match; 951 5'-cagcctCTTGGCCACACGCATc-3' UTR 972; 3'-auacauGUACAUGUGUGUGUAg-5' miRNA; 3'pairing Seed | ACGCAT Imperfect | | | M |
| Imperfect match; 5167 5'-tgTGTAGCACGGTGTTCATACATg-3' UTR 5190; 3'-auACAU-GU-ACAUGUGUGUGUAg-5' miRNA; 3'pairing Seed | ATACAT Imperfect | | | M |
| Imperfect match; 5499 5'-caTG-ACAATGTAAACATACATa-3' UTR 5520; 3'-auACAUG-UACAUGUGUGUGUAg-5' miRNA; 3'pairing Seed | ATACAT Imperfect | | | M |
| Imperfect match; 6811 5'-tgTGTGGGAGCACACGCACATg-3' UTR 6832; 3'-auACAUGUACAUGUGUGUGUAg-5' miRNA; 3'pairing Seed | GCACAT Imperfect | | | M |
| Offset 6mer; 8363 5'-ccgccACACACACACACACAca-3' UTR 8384; 3'-auacaUGUACAUGUGUGUGUAg-5' miRNA; 3'pairing Seed | CACACA Offset 6mer | | | M |
| Offset 6mer; 11629 5'-atcacATATGTGCAGCACACAgg-3' UTR 11651; 3'-auacaUGUACAUGU-GUGUGUAg-5' miRNA; 3'pairing Seed | CACACA Offset 6mer | | | M |
| Imperfect match; 11839 5'-caTGTATAT-TACACCACAAATa-3' UTR 11860; 3'-auACAUGUACAUGU-GUGUGUAg-5' miRNA; 3'pairing Seed | ACAAAT Imperfect | | | M |
| Imperfect match; 12035 5'-tgTATATATGTGCACCACATGTg-3' UTR 12057; 3'-auACAUGUACAUGU-GUGUGUAg-5' miRNA; 3'pairing Seed | ACATGT Imperfect | | | M |
| 8mer; 12727 5'-tgTATATACTCTCTCTCACACATa-3' UTR 12750; 3'-auACAUGU-ACA-UGUGUGUGUAg-5' miRNA; 3'pairing Seed | CACACATA 8mer | | | M T |
| Imperfect match; 12839 5'-aaaagATGTGTGGTCACATATc-3' UTR 12860; 3'-auacaUGUACAUGUGUGUGUAg-5' miRNA; 3'pairing Seed | ACATAT Imperfect | | | M |
| Offset 6mer; 14872 5'-taTGTACGTACACACCACACACa-3' UTR 14894; 3'-auACAUGUACAUGU-GUGUGUAg-5' miRNA; 3'pairing Seed | CACACA Offset 6mer | | | M |
| Imperfect match; 15001 5'-tgTATATATATATACATACATa-3' UTR 15022; 3'-auACAUGUACAUGUGUGUGUAg-5' miRNA; 3'pairing Seed | ATACAT Imperfect | | | M |
| 6mer; 15021 5'-tacaTATATATATATACACATt-3' UTR 15042; 3'-auacAUGUACAUGUGUGUGUAg-5' miRNA; 3'pairing Seed | ACACAT 6mer | | | M |
| Offset 6mer; 18766 5'-caccatggcGTGCACACACACt-3' UTR 18787; 3'-auacauguaCAUGUGUGUGUAg-5' miRNA; 3'pairing Seed | CACACA Offset 6mer | | | M |
| Offset 6mer; 18778 5'-cacacACACTCACACACACAca-3' UTR 18799; 3'-auacaUGUACAUGUGUGUGUAg-5' miRNA; 3'pairing Seed | CACACA Offset 6mer | | | M |
| Offset 6mer; 26313 5'-cacacAGAAGCACACACACAgc-3' UTR 26334; 3'-auacaUGUACAUGUGUGUGUAg-5' miRNA; 3'pairing Seed | CACACA Offset 6mer | | | M |
| Offset 6mer; 30100 5'-atatcACACCTAGACACACAgg-3' UTR 30121; 3'-auacaUGUACAUGUGUGUGUAg-5' miRNA; 3'pairing Seed | CACACA Offset 6mer | | | M |
| 7mer-m8; 33689 5'-tagacACATGTATGCACACACATg-3' UTR 33712; 3'-auacaUGUACAU--GUGUGUGUAg-5' miRNA; 3'pairing Seed | CACACAT 7mer-m8 | | | M T |
| Offset 6mer; 33703 5'-cacacACATGTACACACACAca-3' UTR 33724; 3'-auacaUGUACAUGUGUGUGUAg-5' miRNA; 3'pairing Seed | CACACA Offset 6mer | | | M |
| Offset 6mer; 33719 5'-cacacACACACACACACACAca-3' UTR 33740; 3'-auacaUGUACAUGUGUGUGUAg-5' miRNA; 3'pairing Seed | CACACA Offset 6mer | | | M |
| Imperfect match; 35699 5'-caTATATACATATACACATATa-3' UTR 35720; 3'-auACAUGUACAUGUGUGUGUAg-5' miRNA; 3'pairing Seed | ACATAT Imperfect | | | M |
| Imperfect match; 35715 5'-caTATATATTTACACACACTTa-3' UTR 35736; 3'-auACAUGUACAUGUGUGUGUAg-5' miRNA; 3'pairing Seed | ACACTT Imperfect | | | M |
| Imperfect match; 35735 5'-tatacATATGCATAAACATACATa-3' UTR 35758; 3'-auacaUGUAC--AUGUGUGUGUAg-5' miRNA; 3'pairing Seed | ATACAT Imperfect | | | M |
| Imperfect match; 36463 5'-agTGTACTCACGTATTCACATATa-3' UTR 36486; 3'-auACAU--GUACAUGUGUGUGUAg-5' miRNA; 3'pairing Seed | ACATAT Imperfect | | | M |

| 2D Structure | Local AU | Position | Conservation | Predicted By |
|---|---|---|---|---|
| Imperfect match<br>96 118<br>5'-tttaaCATGTTTATCACATACGg-3' UTR<br>\|\|::\| :\| \|\|\|\|:\|\|\|<br>3'-uguacGUGUACGU-GUGUGUGCa-5' miRNA<br>16 15 14 13 7 6 5 4 3 2<br>3'pairing Seed | 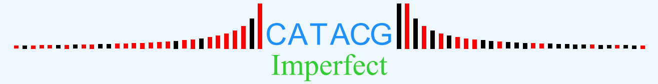 | 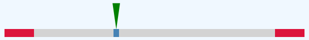 | ☐ | M |
| 7mer-m8<br>252 271<br>5'-gcAAGCA-AGGTG-ACACACGc-3' UTR<br>\| \|\|\| \| \|:: \|\|\|\|\|\|\|<br>3'-ugUACGUGUACGUGUGUGUGCa-5' miRNA<br>16 15 14 13 7 6 5 4 3 2<br>3'pairing Seed | 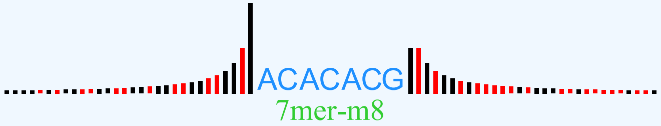 | 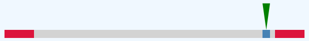 | ☐ | M T |

| 2D Structure | Local AU | Position | Conservation | Predicted By |
|---|---|---|---|---|
| Offset 6mer<br>190 210<br>5'-aggcTACCAGC-CCTGCACATt-3' UTR<br>3'-auguGUGUUCGUGUACGUGUGu-5' miRNA<br>16 15 14 13 7 6 5 4 3 2<br>3'pairing Seed | TGCACA<br>Offset 6mer | | | M |
| 7mer-m8<br>324 343<br>5'-ttTGCACATAGC---TGCACACt-3' UTR<br>3'-auGUGUGU-UCGUGUACGUGUGu-5' miRNA<br>16 15 14 13 7 6 5 4 3 2<br>3'pairing Seed | TGCACAC<br>7mer-m8 | | | M T |

| 2D Structure | Local AU | Position | Conservation | Predicted By |
|---|---|---|---|---|
| 1067 7mer-m8 1095<br>5'-tttCATGTACTAATGAAGAGTCACACACt-3' UTR<br>3'-aguGUACAUG--UACAU----GUGUGUGu-5' miRNA<br>3'pairing Seed | CACACAC<br>7mer-m8 | | | M T |
| 4895 Offset 6mer 4920<br>5'-agACATTGTACCTGAGAGCACACAga-3' UTR<br>3'-agUGU-ACAUGUA--CAUGUGUGUgu-5' miRNA<br>3'pairing Seed | CACACA<br>Offset 6mer | | | M |
| 11354 Imperfect match 11376<br>5'-gcACATGTTAC-TGTGCACATAac -3' UTR<br>3'-agUGUAC-AUGUACAUGUGUGUgu-5' miRNA<br>3'pairing Seed | ACATAA<br>Imperfect | | | M |
| 13692 Imperfect match 13713<br>5'-actCATGCAT-TCCACATACACt -3' UTR<br>3'-aguGUACAUGUACAUGUGUGUGu-5' miRNA<br>3'pairing Seed | ATACAC<br>Imperfect | | | M |
| 15078 Imperfect match 15100<br>5'-gagtcTGTGTAGAGACACACGCa -3' UTR<br>3'-aguguACAUGUACAUGUGUGUGu-5' miRNA<br>3'pairing Seed | ACACGC<br>Imperfect | | | M |
| 15551 8mer 15571<br>5'-tcACATGCA-AT-TACACACACa-3' UTR<br>3'-agUGUACAUGUACAUGUGUGUGu-5' miRNA<br>3'pairing Seed | CACACACA<br>8mer | | | M T |
| 15591 Offset 6mer 15613<br>5'-acACACACACACGAACACACAat-3' UTR<br>3'-agUGUACAUGUACAUGUGUGUgu-5' miRNA<br>3'pairing Seed | CACACA<br>Offset 6mer | | | M |
| 16910 7mer-m8 16935<br>5'-tctgGTATGCATTTCAAACACACACc-3' UTR<br>3'-aguguACAUGUACA---UGUGUGUGu-5' miRNA<br>3'pairing Seed | CACACAC<br>7mer-m8 | | | M T |
| 17735 Imperfect match 17759<br>5'-tcATGTGCTGCAATGTATACACATg -3' UTR<br>3'-agUGUAC-AUG-UACAUGUGUGUGu-5' miRNA<br>3'pairing Seed | ACACAT<br>Imperfect | | | M |
| 18980 Imperfect match 19002<br>5'-atATATGTATACATATACATACa -3' UTR<br>3'-agUGUACAUGUACAUGUGUGUGu-5' miRNA<br>3'pairing Seed | ACATAC<br>Imperfect | | | M |
| 23762 Imperfect match 23783<br>5'-taATATTGACGT-TTCACATACt -3' UTR<br>3'-agUGUACAUGUACAUGUGUGUGu-5' miRNA<br>3'pairing Seed | ACATAC<br>Imperfect | | | M |
| 24168 Offset 6mer 24192<br>5'-gctgtgGTAAATGTAAACACACATa-3' UTR<br>3'-aguguaCAUGUACA--UGUGUGUGu-5' miRNA<br>3'pairing Seed | CACACA<br>Offset 6mer | | | M |
| 24392 Imperfect match 24412<br>5'-aaACATCTG-AT-TACACATACa -3' UTR<br>3'-agUGUACAUGUACAUGUGUGUGu-5' miRNA<br>3'pairing Seed | ACATAC<br>Imperfect | | | M |
| 24787 8mer 24808<br>5'-tcctcTG-ACCTACACACACACa-3' UTR<br>3'-aguguACAUGUACAUGUGUGUGu-5' miRNA<br>3'pairing Seed | CACACACA<br>8mer | | | M T |
| 24951 8mer 24974<br>5'-atATATATATAATATACACACACa-3' UTR<br>3'-agUGUACAUG-UACAUGUGUGUGu-5' miRNA<br>3'pairing Seed | CACACACA<br>8mer | | | M T |
| 24968 8mer 24990<br>5'-acACACACACACACACACACACa-3' UTR<br>3'-agUGUACAUGUACAUGUGUGUGu-5' miRNA<br>3'pairing Seed | CACACACA<br>8mer | | | M T |
| 28144 Offset 6mer 28166<br>5'-agATAAGCAAATATACACACATa-3' UTR<br>3'-agUGUACAUGUACAUGUGUGUGu-5' miRNA<br>3'pairing Seed | CACACA<br>Offset 6mer | | | M |

| 2D Structure | Local AU | Position | Conservation | Predicted By |
|---|---|---|---|---|
| Offset 6mer; 173 5'-gtGTGCGCATGCGCACACAga-3' UTR 193; 3'-uaCAUGUACAUGUGUGUGUAu-5' miRNA; 3'pairing Seed | CACACA Offset 6mer | | | M |
| Imperfect match; 952 5'-agcctCTTGGCCACACGCATc -3' UTR 972; 3'-uacauGUACAUGUGUGUGUAu-5' miRNA; 3'pairing Seed | ACGCAT Imperfect | | | M |
| Imperfect match; 5499 5'-catgACAATGTAAACATACATa -3' UTR 5520; 3'-uacaUG-UACAUGUGUGUGUAu-5' miRNA; 3'pairing Seed | ATACAT Imperfect | | | M |
| Imperfect match; 6812 5'-gtGTGGGAGCACACGCACATg -3' UTR 6832; 3'-uaCAUGUACAUGUGUGUGUAu-5' miRNA; 3'pairing Seed | GCACAT Imperfect | | | M |
| Offset 6mer; 8364 5'-cgccACACACACACACACACa-3' UTR 8384; 3'-uacaUGUACAUGUGUGUGUAu-5' miRNA; 3'pairing Seed | CACACA Offset 6mer | | | M |
| Offset 6mer; 11630 5'-tcacATATGTGCAGCACACAgg-3' UTR 11651; 3'-uacaUGUACAUGU-GUGUGUAu-5' miRNA; 3'pairing Seed | CACACA Offset 6mer | | | M |
| Imperfect match; 12036 5'-gtaTATATGTGCACCACATGTg -3' UTR 12057; 3'-uacAUGUACAUGU-GUGUGUAu-5' miRNA; 3'pairing Seed | ACATGT Imperfect | | | M |
| 8mer; 12728 5'-gtaTATACTCTCTCTCACACATa-3' UTR 12750; 3'-uacAUGU-ACA-UGUGUGUGUAu-5' miRNA; 3'pairing Seed | CACACATA 8mer | | | M T |
| Imperfect match; 12840 5'-aaagATGTGTGGTCACATATc -3' UTR 12860; 3'-uacaUGUACAUGUGUGUGUAu-5' miRNA; 3'pairing Seed | ACATAT Imperfect | | | M |
| Offset 6mer; 14873 5'-atGTACGTACACACCACACAca-3' UTR 14894; 3'-uaCAUGUACAUGU-GUGUGUau-5' miRNA; 3'pairing Seed | CACACA Offset 6mer | | | M |
| Imperfect match; 15002 5'-gtaTATATATATACATACATa -3' UTR 15022; 3'-uacAUGUACAUGUGUGUGUAu-5' miRNA; 3'pairing Seed | ATACAT Imperfect | | | M |
| 6mer; 15022 5'-acaTATATATATATACACATt-3' UTR 15042; 3'-uacAUGUACAUGUGUGUGUAu-5' miRNA; 3'pairing Seed | ACACAT 6mer | | | M |
| Offset 6mer; 18767 5'-accatggcGTGCACACACAct-3' UTR 18787; 3'-uacauguaCAUGUGUGUGUAu-5' miRNA; 3'pairing Seed | CACACA Offset 6mer | | | M |
| Offset 6mer; 18779 5'-acacACACTCACACACACACa-3' UTR 18799; 3'-uacaUGUACAUGUGUGUGUAu-5' miRNA; 3'pairing Seed | CACACA Offset 6mer | | | M |
| Offset 6mer; 26314 5'-acacAGAAGCACACACACAgc-3' UTR 26334; 3'-uacaUGUACAUGUGUGUGUAu-5' miRNA; 3'pairing Seed | CACACA Offset 6mer | | | M |
| Offset 6mer; 30101 5'-tatcACACCTAGACACACAgg-3' UTR 30121; 3'-uacaUGUACAUGUGUGUGUau-5' miRNA; 3'pairing Seed | CACACA Offset 6mer | | | M |
| 7mer-m8; 33690 5'-agacACATGTATGCACACACATg-3' UTR 33712; 3'-uacaUGUACAU--GUGUGUGUAu-5' miRNA; 3'pairing Seed | CACACAT 7mer-m8 | | | M T |
| Offset 6mer; 33704 5'-acacACATGTACACACACACa-3' UTR 33724; 3'-uacaUGUACAUGUGUGUGUAu-5' miRNA; 3'pairing Seed | CACACA Offset 6mer | | | M |
| Offset 6mer; 33720 5'-acacACACACACACACACACa-3' UTR 33740; 3'-uacaUGUACAUGUGUGUGUAu-5' miRNA; 3'pairing Seed | CACACA Offset 6mer | | | M |
| Offset 6mer; 35714 5'-acaTATATATTTACACACAct-3' UTR 35734; 3'-uacAUGUACAUGUGUGUGUau-5' miRNA; 3'pairing Seed | CACACA Offset 6mer | | | M |
| Imperfect match; 35736 5'-atacATATGCATAAACATACATa -3' UTR 35758; 3'-uacaUGUAC--AUGUGUGUGUAu-5' miRNA; 3'pairing Seed | ATACAT Imperfect | | | M |
| Imperfect match; 36464 5'-gtGTACTCACGTATTCACATATa -3' UTR 36486; 3'-uaCAU--GUACAUGUGUGUGUAu-5' miRNA; 3'pairing Seed | ACATAT Imperfect | | | M |

| 2D Structure | Local AU | Position | Conservation | Predicted By |
| --- | --- | --- | --- | --- |
| Imperfect match<br>273 293<br>5'-gaGGCAGATG-GC-TCAGTCGTc -3' UTR<br>\|:\|\| \|:\| :\| \|\|\|\|\|\|:\|<br>3'-guCUGUGUGCGUGUAGUCAGUAu-5' miRNA<br>16 15 14 13 7 6 5 4 3 2<br>3'pairing Seed | 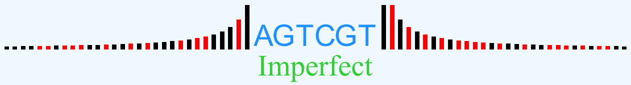 | 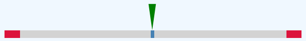 | | M |
| 7mer-m8<br>449 473<br>5'-cgGAAAAACTCATCTTCCAGTCATg-3' UTR<br>\|\| \| \|\| \|\|: \| \|\|\|\|\|\|\|<br>3'-guCUGUGUGCGUGUA--GUCAGUAu-5' miRNA<br>16 15 14 13 7 6 5 4 3 2<br>3'pairing Seed | 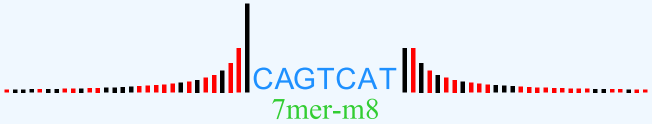 | 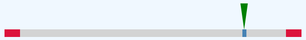 | | M T |

| 2D Structure | Local AU | Position | Conservation | Predicted By |
|---|---|---|---|---|
| Offset 6mer<br>48 70<br>5'-gaTGGCGTGGGAGTAGTGTCAga-3' UTR<br>3'-cgACGGUAUAUACACCACAGUaa-5' miRNA<br>16 15 14 13 7 6 5 4 3 2<br>3'pairing Seed | 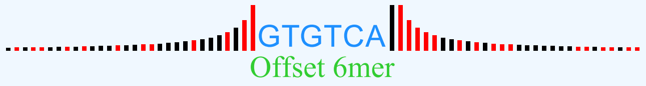<br> | 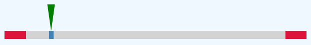 | | M |
| 286 7mer-m8 308<br>5'-gaatggATAGACACCGTGTCATg-3' UTR<br>3'-cgacggUAUAUACACCACAGUAa-5' miRNA<br>16 15 14 13 7 6 5 4 3 2<br>3'pairing Seed | 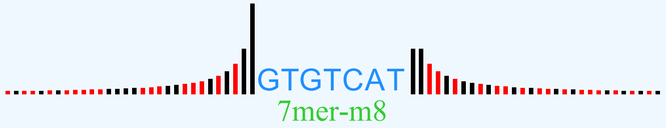<br> | 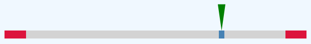 | | M T |

| 2D Structure | Local AU | Position | Conservation | Predicted By |
|---|---|---|---|---|

```
202            7mer-m8  223
   5'-caCAAAACTTACTCTGCTGCTg-3'  UTR
        ||||  | :|  | |||||||
   3'-auGUUUGGUGUCACACGACGAc-5'  miRNA
            16 15 14 13    7 6 5 4 3 2
         3'pairing       Seed
```

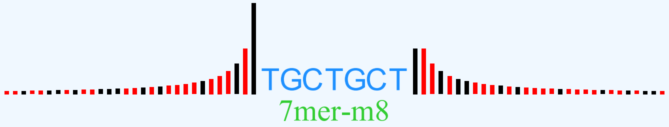


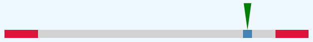

M T

| 2D Structure | Local AU | Position | Conservation | Predicted By |
|---|---|---|---|---|
| 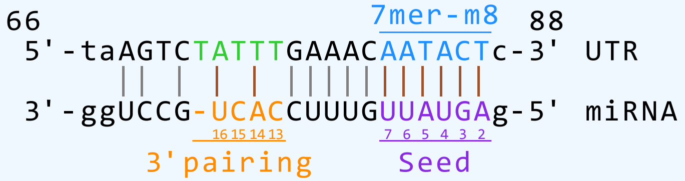  | 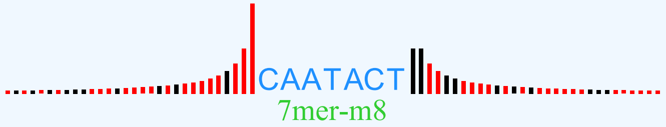  | 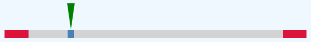 | 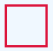 | 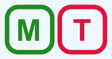  |

<table>
<tr><th>2D Structure</th><th>Local AU</th><th>Position</th><th>Conservation</th><th>Predicted By</th></tr>
<tr><td>86 7mer-m8 105<br>5'-agAACCCTG--CAGCTCTCCCt-3' UTR<br>3'-cuUUGGGACGGGACGAGAGGGa-5' miRNA<br>16 15 14 13 7 6 5 4 3 2<br>3'pairing Seed</td><td>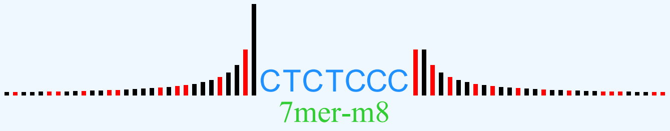
</td><td>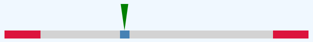</td><td></td><td>M T</td></tr>
<tr><td>98 Offset 6mer 118<br>5'-ctctCCCTG-ACTGCTCTCCTc-3' UTR<br>3'-cuuuGGGACGGGACGAGAGGGa-5' miRNA<br>16 15 14 13 7 6 5 4 3 2<br>3'pairing Seed</td><td>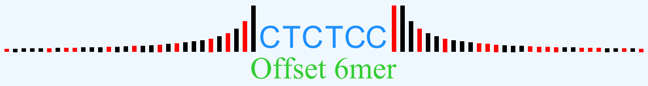
</td><td>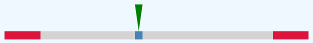</td><td></td><td>M</td></tr>
</table>

<table>
<tr><th>2D Structure</th><th>Local AU</th><th>Position</th><th>Conservation</th><th>Predicted By</th></tr>
<tr><td>83 8mer 108<br>5'-acTAAGTACCTTAGGCAGGTGGGAGa-3' UTR<br>:||| |||| || |||||||<br>3'-acGUUC--GGAACCC--ACACCCUCc-5' miRNA<br>16 15 14 13 7 6 5 4 3 2<br>3'pairing Seed</td><td>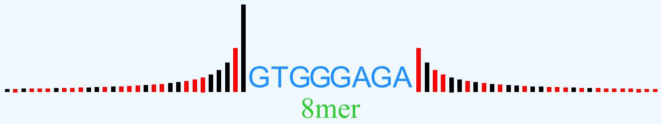<br></td><td>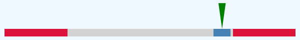</td><td></td><td>M T</td></tr>
</table>

| 2D Structure | Local AU | Position | Conservation | Predicted By |
|---|---|---|---|---|
| 134 7mer-m8 155<br>5'-gaggaggacAAGAATGCAGAAg-3' UTR<br>\|\|:\|\|\|\|\|\|\|\|\|<br>3'-cucgaacgaUUUUUACGUCUUa-5' miRNA<br>16 15 14 13 7 6 5 4 3 2<br>3'pairing Seed | 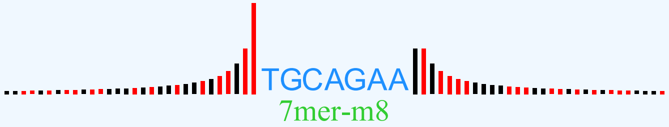 | 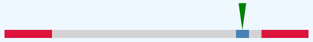 | | M T |

<table>
<tr><th>2D Structure</th><th>Local AU</th><th>Position</th><th>Conservation</th><th>Predicted By</th></tr>
<tr><td>65 7mer-m8 80<br>5'-acCCTCATGCCACCAt-3' UTR<br>| :| ||||||||<br>3'-cuGAGGCACGGUGGUa-5' miRNA<br>16 15 14 13 7 6 5 4 3 2<br>3'pairing Seed</td><td>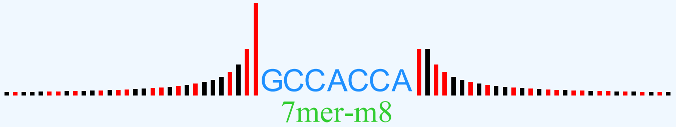</td><td>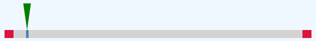</td><td>☐</td><td>M T</td></tr>
<tr><td>147 7mer-m8 160<br>5'-acaTCC--GCCACCAc-3' UTR<br>||| |||||||<br>3'-cugAGGCACGGUGGUa -5' miRNA<br>3'pairing Seed</td><td>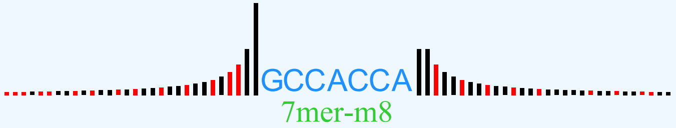</td><td>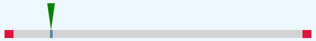</td><td>☐</td><td>M T</td></tr>
<tr><td>Imperfect match<br>556 571<br>5'-ccCTCAGTGCCATCAt -3' UTR<br>||| |||||:||<br>3'-cuGAGGCACGGUGGUa-5' miRNA<br>16 15 14 13 7 6 5 4 3 2<br>3'pairing Seed</td><td>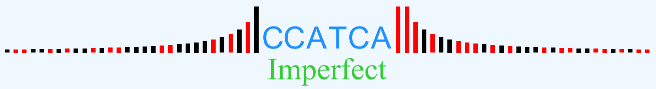</td><td>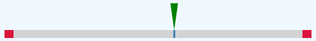</td><td>☐</td><td>M</td></tr>
<tr><td>809 7mer-m8 825<br>5'-agCTCCAAGGCCACCAg-3' UTR<br>|||| |||||||<br>3'-cuGAGG-CACGGUGGUa -5' miRNA<br>3'pairing Seed</td><td>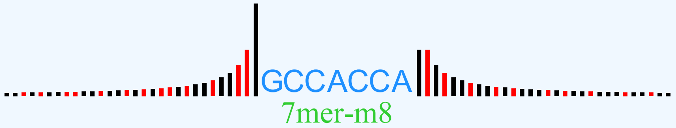</td><td>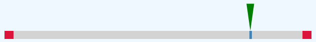</td><td>☐</td><td>M T</td></tr>
</table>

| 2D Structure | Local AU | Position | Conservation | Predicted By |
| --- | --- | --- | --- | --- |
| see below | 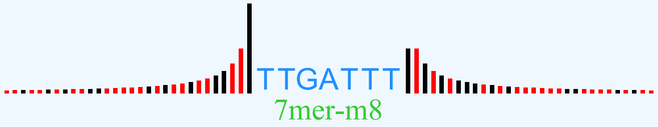 | 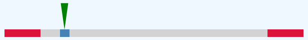 | | M T |

```
32               7mer-m8    54
 5'-taGTCTTCAAGC-ATCTTGATTTg-3'  UTR
      ||| |||  | | ||||||||
 3'-ucCAG-AGUGGGUUCGAACUAAAg-5'  miRNA
           16 15 14 13   7 6 5 4 3 2
          3'pairing        Seed
```

<table>
<tr><th>2D Structure</th><th>Local AU</th><th>Position</th><th>Conservation</th><th>Predicted By</th></tr>
<tr><td>308 7mer-m8 330<br>5'-atGTTTAATCAAGTTTTGATTTt-3' UTR<br>||:| |:| |: :|||||||<br>3'-ucCAGAGUGGGUUCGAACUAAAg-5' miRNA<br>16 15 14 13 7 6 5 4 3 2<br>3'pairing Seed</td><td>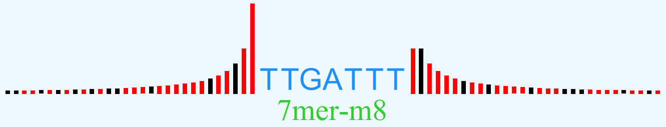</td><td>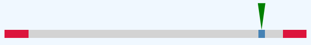</td><td></td><td>M T</td></tr>
</table>

| 2D Structure | Local AU | Position | Conservation | Predicted By |
| --- | --- | --- | --- | --- |
| Imperfect match<br>42 64<br>5'-atATTAATTGCATCGAGGCACAAg -3' UTR<br>\|\|:\| :\|\|\|\| \|:\|\|\|\|\|<br>3'-ugUAGUAGCGUAUAACUGUGUUa-5' miRNA<br>16 15 14 13 7 6 5 4 3 2<br>3'pairing Seed | 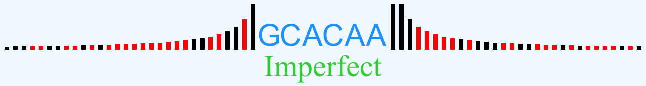 | 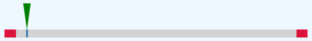 | ☐ | M |
| Imperfect match<br>171 191<br>5'-tgATTAT-GAGTA-TGACGCAAa -3' UTR<br>\|\|:\|\| \| :\|\| \|\|\|\|:\|\|\|<br>3'-ugUAGUAGCGUAUAACUGUGUUa-5' miRNA<br>16 15 14 13 7 6 5 4 3 2<br>3'pairing Seed | 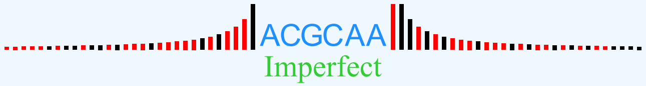 | 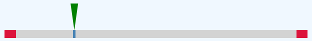 | ☐ | M |

<table>
<tr><th>2D Structure</th><th>Local AU</th><th>Position</th><th>Conservation</th><th>Predicted By</th></tr>
<tr><td>108 7mer-m8 126<br>5'-atcacATACGGTGACACAt-3' UTR<br>||| : ||||||<br>3'-caaacUAUUUGACUGUGUu-5' miRNA<br>16 15 14 13 7 6 5 4 3 2<br>3'pairing Seed</td><td>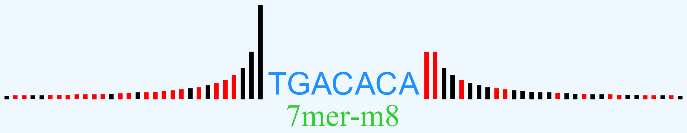</td><td>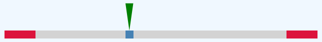</td><td></td><td>M T</td></tr>
<tr><td>251 7mer-m8 269<br>5'-agcaagcAAGGTGACACAc-3' UTR<br>||: ||||||<br>3'-caaacuaUUUGACUGUGUu-5' miRNA<br>16 15 14 13 7 6 5 4 3 2<br>3'pairing Seed</td><td>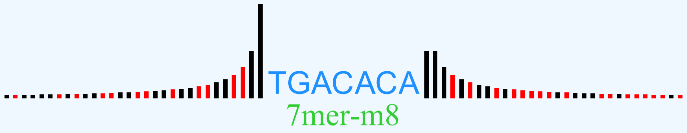</td><td>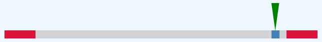</td><td></td><td>M T</td></tr>
</table>

| 2D Structure | Local AU | Position | Conservation | Predicted By |
|---|---|---|---|---|
| 158 7mer-m8 180<br>5'-caTTCCCCTACTTCATGCCCGCt-3' UTR<br>:\|\| \| :\| :\| \|\|\|\|\|\|\|<br>3'-ccGAGAGUGUCGA-CACGGGCGa-5' miRNA<br>16 15 14 13 7 6 5 4 3 2<br>3'pairing Seed | 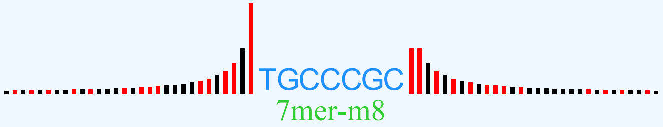 | 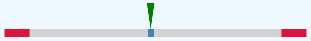 | | M T |
| Offset 6mer<br>178 200<br>5'-gcTTCTCTCAGTATGTGCCCGgg-3' UTR<br>:\|\|\|\| \|\|\|: \|\|\|\|\|\|\|\|<br>3'-ccGAGAGUGUCG-ACACGGGCga-5' miRNA<br>16 15 14 13 7 6 5 4 3 2<br>3'pairing Seed | 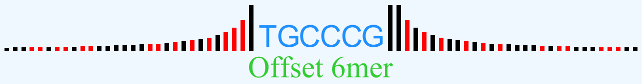 | 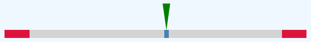 | | M |

| 2D Structure | Local AU | Position | Conservation | Predicted By |
|---|---|---|---|---|
| Imperfect match<br>270 294<br>5'-aaTGCT-GCTGCTGTCACCACCACCa -3' UTR<br>\|\|\|\| :\|\|\|\|\| :\|\|\|\|\| \|\|\|\|<br>3'-gcACGAGUGACGA-GGUGGUCGUGGc-5' miRNA<br>16 15 14 13 7 6 5 4 3 2<br>3'pairing Seed | 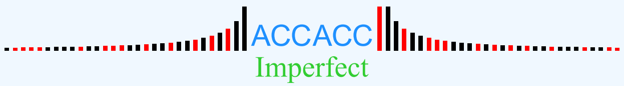 ACCACC Imperfect | 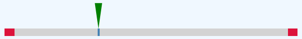 | □ | M |
| 7mer-m8<br>592 615<br>5'-agTTCTCGC-GGGACGCCAGCACCt-3' UTR<br>\| \|\|\|:\| \| \|:\|\|\|\|\|\|\|\|<br>3'-gcACGAGUGACGAGGUGGUCGUGGc-5' miRNA<br>16 15 14 13 7 6 5 4 3 2<br>3'pairing Seed | 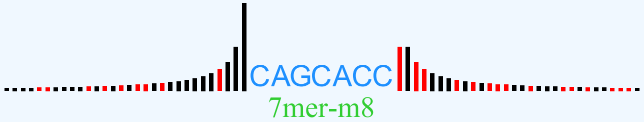 CAGCACC 7mer-m8 | 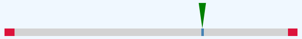 | □ | M T |

<table>
<tr><th>2D Structure</th><th>Local AU</th><th>Position</th><th>Conservation</th><th>Predicted By</th></tr>
<tr><td><pre>
                       Imperfect
                         match
59                                81
5'-caTGCTCTCTG--CCGGTGGCACCa -3'  UTR
     ||||| |||  ||: ::|||||
3'-gcACGAGUGACGAGGUGGUCGUGGc-5'  miRNA
            16 15 14 13     7 6 5 4 3 2
          3'pairing          Seed
</pre></td><td>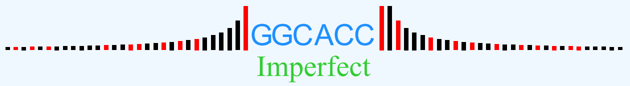
</td><td>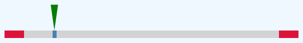</td><td></td><td>M</td></tr>
<tr><td><pre>
                          7mer-m8
74                                98
5'-tggcacCACAGCCACCA-CAGCACCt-3'  UTR
         ||| | | ||| |||||||
3'-gcacgaGUGAC-GAGGUGGUCGUGGc-5'  miRNA
            16 15  14 13     7 6 5 4 3 2
          3'pairing           Seed
</pre></td><td>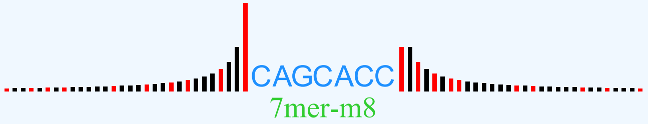
</td><td>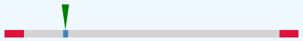</td><td></td><td>M T</td></tr>
</table>

| 2D Structure | Local AU | Position | Conservation | Predicted By |
|---|---|---|---|---|
| 207 7mer-m8 235<br>5'-tttCTACTTGGTGGCAGATAGTGAGGAAg-3' UTR<br>|| || ::|| |||| |||||||<br>3'-uguGAGGACUUAC---CUAU-ACUCCUUu-5' miRNA<br>16 15 14 13 7 6 5 4 3 2<br>3'pairing Seed | 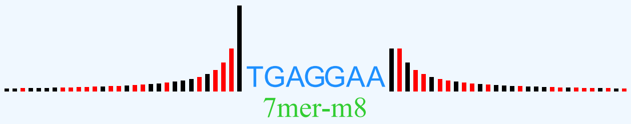 | 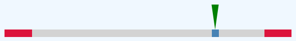 | | M T |

| 2D Structure | Local AU | Position | Conservation | Predicted By |
| --- | --- | --- | --- | --- |
| 73 8mer 92<br>5'-agCACTGC-TTCA-TCAGGGAa-3' UTR<br>\|\|\|::\| \| \|\| \|\|\|\|\|\|\|<br>3'-aaGUGGUGUAUGUGAGUCCCUa-5' miRNA<br>16 15 14 13 7 6 5 4 3 2<br>3'pairing Seed | 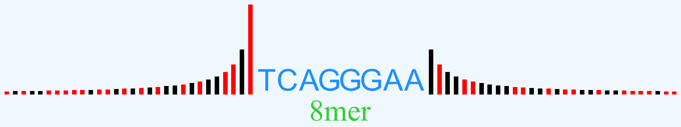 | 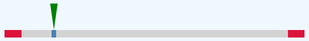 | | M T |
| Imperfect match<br>320 341<br>5'-agtcCCAGATACACTCAGGAAg-3' UTR<br>\|\|\| \|\|\|\|\|\|\|\|\|\|\| \|<br>3'-aaguGGUGUAUGUGAGUCCCUa-5' miRNA<br>16 15 14 13 7 6 5 4 3 2<br>3'pairing Seed | 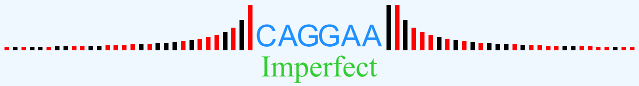 | 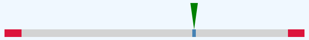 | | M |

| 2D Structure | Local AU | Position | Conservation | Predicted By |
| --- | --- | --- | --- | --- |
| 80 7mer-m8 102<br>5'-ctgaACCGACTCTATGGCTTCCt-3' UTR<br>3'-gaggUCGGGGAGGUCCCGAAGGa-5' miRNA<br>16 15 14 13 7 6 5 4 3 2<br>3'pairing Seed | 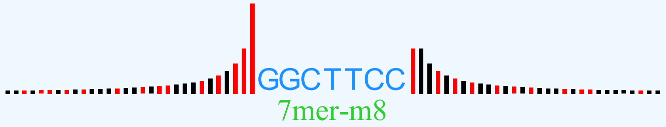 | 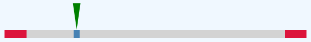 | | M T |

<table>
<tr><th>2D Structure</th><th>Local AU</th><th>Position</th><th>Conservation</th><th>Predicted By</th></tr>
<tr><td>86 7mer-m8 105<br>5'-acctcgccGGT-ACTGTGTGc-3' UTR<br>:|| ||||||||<br>3'-agcuucuaUCACUGACACACa-5' miRNA<br>16 15 14 13 7 6 5 4 3 2<br>3'pairing Seed</td><td>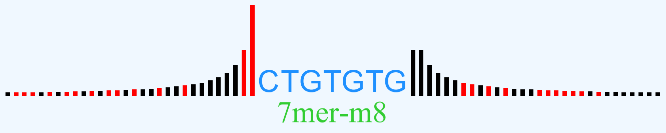
</td><td>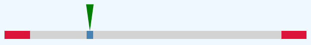</td><td></td><td>M T</td></tr>
<tr><td>238 7mer-m8 260<br>5'-tttcAGATATGGAAGCTGTGTGg-3' UTR<br>||||| || |||||||<br>3'-agcuUCUAUCACU--GACACACa-5' miRNA<br>16 15 14 13 7 6 5 4 3 2<br>3'pairing Seed</td><td>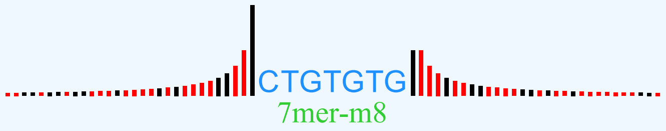
</td><td>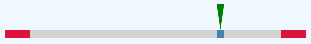</td><td></td><td>M T</td></tr>
</table>

| 2D Structure | Local AU | Position | Conservation | Predicted By |
|---|---|---|---|---|
| 422 7mer-m8 443<br>5'-cataAACGGACAAAGCTGTGAt-3' UTR<br>\|\| \|: \|\| \|\|\|\|\|\|\|<br>3'-gaggUUCCUGGUCUCGACACUc-5' miRNA<br>16 15 14 13 7 6 5 4 3 2<br>3'pairing Seed | 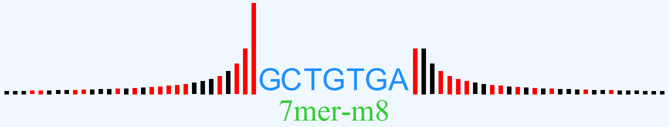 | 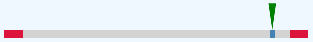 | | M T |

| 2D Structure | Local AU | Position | Conservation | Predicted By |
|---|---|---|---|---|
| 323 8mer 344<br>5'-tgATTATGTTGGGGACCTTGCa-3' UTR<br>\|:\| \|\| \|:\|\|\|\|\|\|\|\|\|<br>3'-gaUGACACUCCUCCUGGAACGa-5' miRNA<br>16 15 14 13 7 6 5 4 3 2<br>3'pairing Seed | 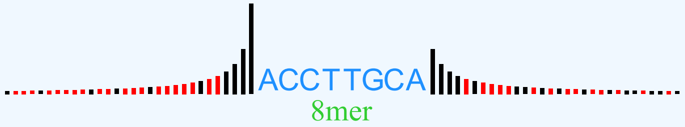 | 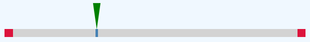 | ☐ | M T |
| Offset 6mer<br>491 512<br>5'-tgccgcaGAGGATGACCTTGTt-3' UTR<br>\|\|\|\|\| \|\|\|\|\|\|:<br>3'-gaugacaCUCCUCCUGGAACGa-5' miRNA<br>16 15 14 13 7 6 5 4 3 2<br>3'pairing Seed | 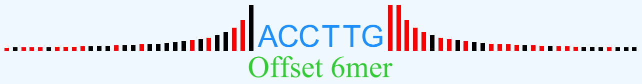 | 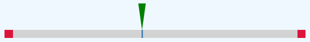 | ☐ | M |

<table>
<tr><th>2D Structure</th><th>Local AU</th><th>Position</th><th>Conservation</th><th>Predicted By</th></tr>
<tr><td>92 7mer-m8 111<br>5'-attctgACAG-TGACAGCAGt-3' UTR<br>||||  |  |||||||<br>3'-cuccugUGUCGAAUGUCGUCc-5' miRNA<br>16 15 14 13 7 6 5 4 3 2<br>3'pairing Seed</td><td>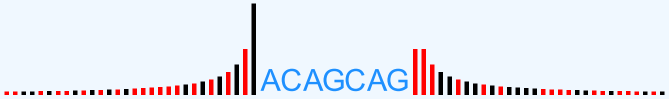<br>ACAGCAG<br>7mer-m8</td><td>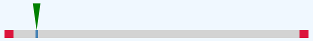</td><td>☐</td><td>M T</td></tr>
<tr><td>Offset 6mer<br>147 168<br>5'-caaGATTGCATCTTACAGCAaa-3' UTR<br>||  ::||  |||||||||<br>3'-cucCU-GUGUCGAAUGUCGUcc-5' miRNA<br>16 15 14 13 7 6 5 4 3 2<br>3'pairing Seed</td><td>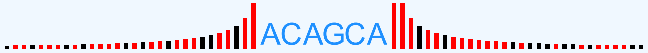<br>ACAGCA<br>Offset 6mer</td><td>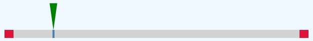</td><td>☐</td><td>M</td></tr>
<tr><td>Offset 6mer<br>468 488<br>5'-cctaATGCAGATGACAGCAag-3' UTR<br>|::|||  |  ||||||<br>3'-cuccUGUGUCGAAUGUCGUcc-5' miRNA<br>16 15 14 13 7 6 5 4 3 2<br>3'pairing Seed</td><td>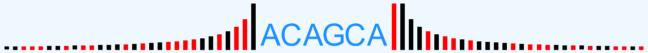<br>ACAGCA<br>Offset 6mer</td><td>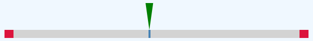</td><td>☐</td><td>M</td></tr>
</table>

<table>
<tr><th>2D Structure</th><th>Local AU</th><th>Position</th><th>Conservation</th><th>Predicted By</th></tr>
<tr>
<td>123 7mer-m8 146<br>5'-aaaatgGCCTCCTTAATCAGGAAt-3' UTR<br>||||| :| |||||||<br>3'-aaaagaCGGAGAGA--AGUCCUUa-5' miRNA<br>16 15 14 13 7 6 5 4 3 2<br>3'pairing Seed</td>
<td>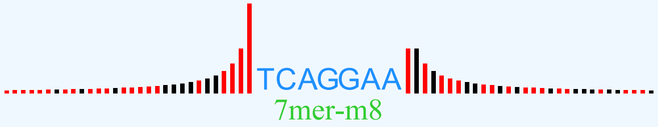
</td>
<td>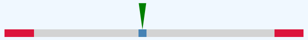</td>
<td></td>
<td>M T</td>
</tr>
</table>

| 2D Structure | Local AU | Position | Conservation | Predicted By |
| --- | --- | --- | --- | --- |
| 243 7mer-m8 263<br>5'-gcGAGAGCCGAGCAAAGAGAg-3' UTR<br>3'-cuCACUCCGCGAGUUUCUCUa-5' miRNA<br>16 15 14 13 7 6 5 4 3 2<br>3'pairing Seed | 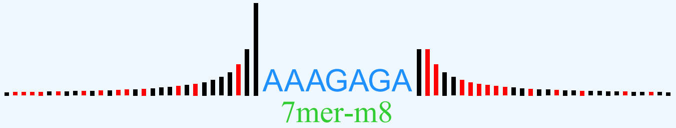<br> | 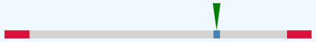 | | M T |

<table>
<tr><th>2D Structure</th><th>Local AU</th><th>Position</th><th>Conservation</th><th>Predicted By</th></tr>
<tr><td>Offset 6mer<br>41 63<br>5'-cgACAGGCGGATACAAAAGCTat-3' UTR<br>3'-aaUGUCGGUGUAGACUUUCGACg-5' miRNA<br>16 15 14 13 7 6 5 4 3 2<br>3'pairing Seed</td><td>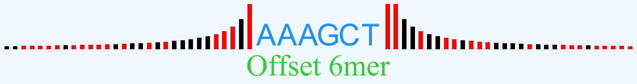
</td><td>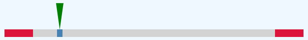</td><td></td><td>M</td></tr>
<tr><td>7mer-m8<br>232 255<br>5'-ggGCTGCAAAGGTTCAAAAGCTGg-3' UTR<br>3'-aaUGUCG-GUGUAGACUUUCGACg-5' miRNA<br>16 15 14 13 7 6 5 4 3 2<br>3'pairing Seed</td><td>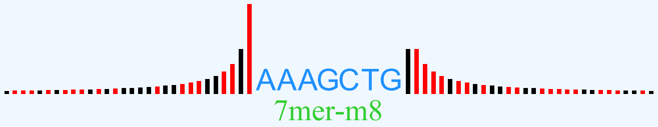
</td><td>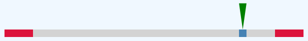</td><td></td><td>M T</td></tr>
</table>

| 2D Structure | Local AU | Position | Conservation | Predicted By |
|---|---|---|---|---|
| 56 8mer 78<br>5'-gtATGGATGCCTGAGAAAGCTGa-3' UTR<br>\|::\| ::\| \| \|\|\|\|\|\|\|<br>3'-aaUGUCGGUGUAGACUUUCGACg-5' miRNA<br>16 15 14 13 7 6 5 4 3 2<br>3'pairing Seed | 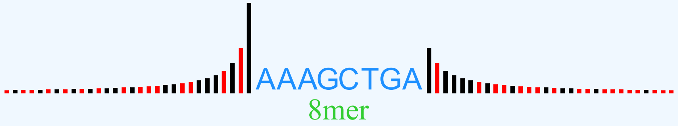 | 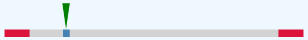 | | M T |
| 147 8mer 169<br>5'-gaaatggggaagagGAAAGCTGa-3' UTR<br>\|\|\|\|\|\|\|<br>3'-aaugucgguguagaCUUUCGACg-5' miRNA<br>16 15 14 13 7 6 5 4 3 2<br>3'pairing Seed | 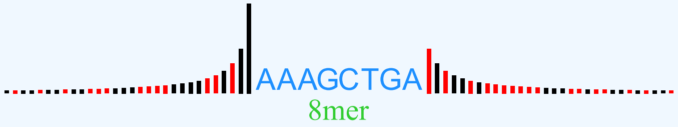 | 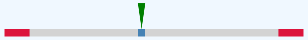 | | M T |

| 2D Structure | Local AU | Position | Conservation | Predicted By |
|---|---|---|---|---|
| 162 6mer 181<br>5'-caagaagaTCACTTCCCACc-3' UTR<br>:\|\|\|:\|\|\|\|\|\|<br>3'-acgggugggGUGGAGGGUGg-5' miRNA<br>16 15 14 13 7 6 5 4 3 2<br>3'pairing Seed | 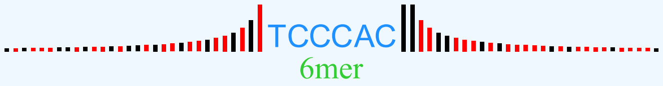 | 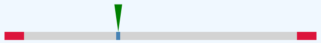 | ☐ | M |
| Imperfect match<br>219 238<br>5'-ctTCCAGGTTACCTTCCACc -3' UTR<br>:\|\|\| ::\|\|\|\|:\|\|\|\|<br>3'-acGGGUGGGGUGGAGGGUGg-5' miRNA<br>16 15 14 13 7 6 5 4 3 2<br>3'pairing Seed | 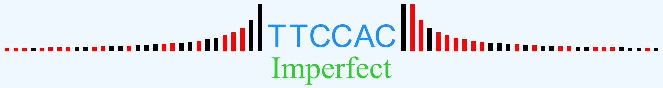 | 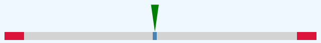 | ☐ | M |

<table>
<tr><th>2D Structure</th><th>Local AU</th><th>Position</th><th>Conservation</th><th>Predicted By</th></tr>
<tr><td>193 8mer 213<br>5'-tccAGCTCATCTGTGAAGGAa-3' UTR<br>| | | : | | | | | | | |<br>3'-ccaUGGCGGGCCCACUUCCUc-5' miRNA<br>16 15 14 13 7 6 5 4 3 2<br>3'pairing Seed</td><td>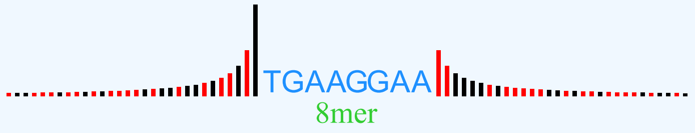
</td><td>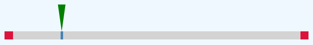</td><td></td><td>M T</td></tr>
<tr><td>431 7mer-m8 449<br>5'-ggaACTGGTC--CTGAAGGAg-3' UTR<br>| | : | : | | | | | | | |<br>3'-ccaUGGCGGGCCCACUUCCUc-5' miRNA<br>16 15 14 13 7 6 5 4 3 2<br>3'pairing Seed</td><td>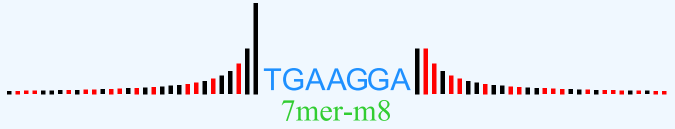
</td><td>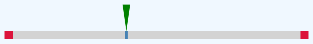</td><td></td><td>M T</td></tr>
<tr><td>994 7mer-m8 1014<br>5'-gactaattacctcTGAAGGAg-3' UTR<br>| | | | | | |<br>3'-ccauggcgggcccACUUCCUc-5' miRNA<br>16 15 14 13 7 6 5 4 3 2<br>3'pairing Seed</td><td>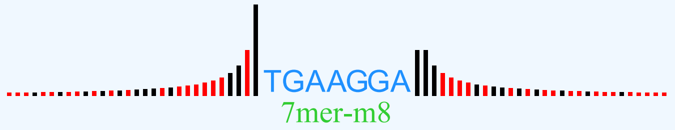
</td><td>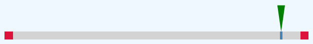</td><td></td><td>M T</td></tr>
</table>

| 2D Structure | Local AU | Position | Conservation | Predicted By |
|---|---|---|---|---|
| 159 7mer-m8 179<br>5'-acTTTCCAGTAT-TTGTCTTTt-3' UTR<br>\| \| \|\|\|:\|\| \|\|\|\|\|\|\|<br>3'-agAUAAGUCGUACUACAGAAAg-5' miRNA<br>16 15 14 13 7 6 5 4 3 2<br>3'pairing Seed | 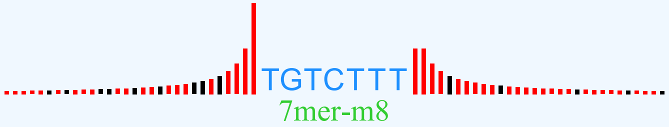  | 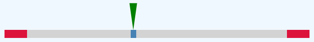 | | M T |

| 2D Structure | Local AU | Position | Conservation | Predicted By |
| --- | --- | --- | --- | --- |
| 30 7mer-m8 50<br>5'-caCAACA-TGTCTTCACTCTGt-3' UTR<br>3'-caGUUUUAACAAACGUGAGACg-5' miRNA<br>16 15 14 13 7 6 5 4 3 2<br>3'pairing Seed | 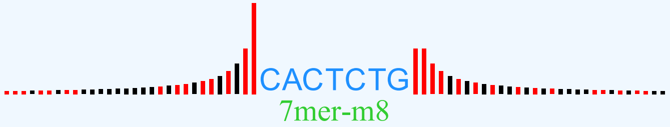 | 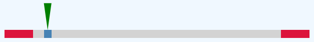 | | M T |

| 2D Structure | Local AU | Position | Conservation | Predicted By |
| --- | --- | --- | --- | --- |
| 123 8mer 144<br>5'-aaggctataatgaaCCAGCTGa-3' UTR<br>\|\|\|\|\|\|\|<br>3'-cggugaccaagaagGGUCGACc-5' miRNA<br>16 15 14 13 7 6 5 4 3 2<br>3'pairing Seed | 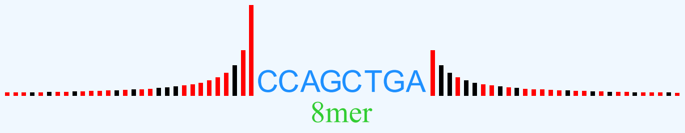 | 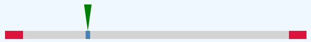 | | M T |
| 393 7mer-m8 414<br>5'-atggggtccaggaaCCAGCTGc-3' UTR<br>\|\|\|\|\|\|\|<br>3'-cggugaccaagaagGGUCGACc-5' miRNA<br>16 15 14 13 7 6 5 4 3 2<br>3'pairing Seed | 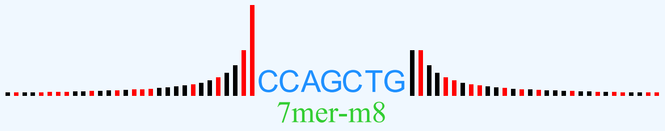 | 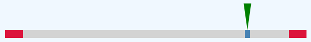 | | M T |

| 2D Structure | Local AU | Position | Conservation | Predicted By |
| --- | --- | --- | --- | --- |
| 175 7mer-m8 195<br>5'-ccctGCTCA-CCGCGTGGTGCt-3' UTR<br>\|\|:\|\|  \|:\|\|\|\|\|\|\|\|<br>3'-gcacCGGGUCUGUGCACCACGa-5' miRNA<br>16 15 14 13 7 6 5 4 3 2<br>3'pairing Seed | 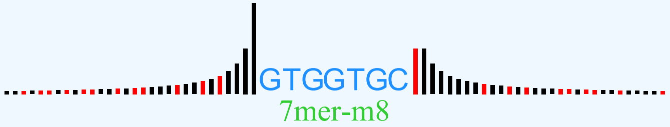 | 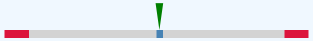 | 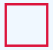 | M T |

| 2D Structure | Local AU | Position | Conservation | Predicted By |
|---|---|---|---|---|
| 142 8mer 163<br>5'-ccaTGGATGAGCAGGAACTTGa-3' UTR<br>3'-cacACGUAAACGUCCUUGAACa-5' miRNA<br>16 15 14 13 7 6 5 4 3 2<br>3'pairing Seed | 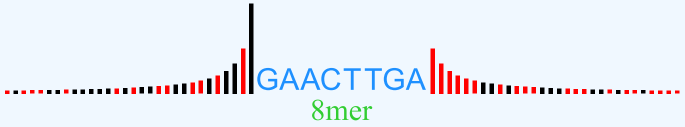 | 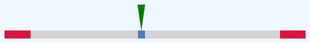 | | M T |

<table>
<tr><th>2D Structure</th><th>Local AU</th><th>Position</th><th>Conservation</th><th>Predicted By</th></tr>
<tr>
<td>309 7mer-m8 330<br>5'-atcaaAGTAACAATGAAATCCt-3' UTR<br>| | ||| |||||||<br>3'-aucacUAAGUGUCUCUUUAGGu-5' miRNA<br>16 15 14 13 7 6 5 4 3 2<br>3'pairing Seed</td>
<td>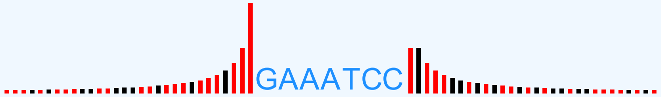</td>
<td>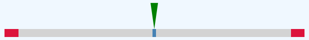</td>
<td></td>
<td>M T</td>
</tr>
<tr>
<td>Imperfect match<br>336 357<br>5'-gaGTGATGTCA-GGAGGAATTCt -3' UTR<br>||||| ||| :|||:|||:|<br>3'-auCACUA-AGUGUCUCUUUAGGu-5' miRNA<br>16 15 14 13 7 6 5 4 3 2<br>3'pairing Seed</td>
<td>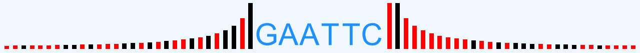</td>
<td>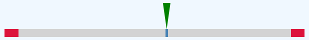</td>
<td></td>
<td>M</td>
</tr>
</table>

| 2D Structure | Local AU | Position | Conservation | Predicted By |
|---|---|---|---|---|
| 185 7mer-m8 206<br>5'-aaCTGCCTGTTTCGTCCTCTAc-3' UTR<br>\|\|\|\| : : \|\| \|\|\|\|\|\|\|<br>3'-ggGACGCG-GUAG-AGGAGAUg-5' miRNA<br>16 15 14 13 7 6 5 4 3 2<br>3'pairing Seed | 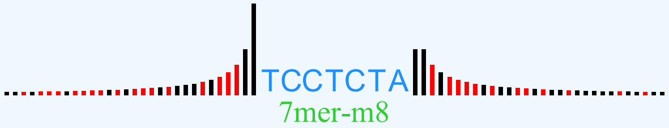 | 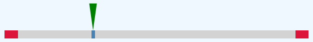 | | M T |
| 393 7mer-m8 411<br>5'-gagaGC-CCATGTCCTCTAt-3' UTR<br>\|\| \|\|\|\| \|\|\|\|\|\|\|<br>3'-gggaCGCGGUAGAGGAGAUg-5' miRNA<br>16 15 14 13 7 6 5 4 3 2<br>3'pairing Seed | 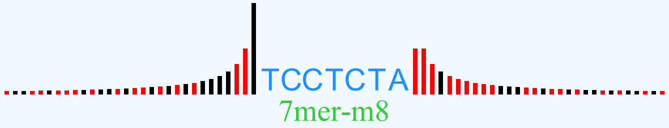 | 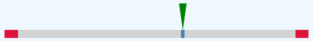 | | M T |

<table>
<tr><th>2D Structure</th><th>Local AU</th><th>Position</th><th>Conservation</th><th>Predicted By</th></tr>
<tr><td>37 7mer-m8 58<br>5'-atgggGTTTACAGGTCTCAGTg-3' UTR<br>|:| | : |||||||<br>3'-acugaCGAUGGGUAAGAGUCAu-5' miRNA<br>16 15 14 13 7 6 5 4 3 2<br>3'pairing Seed</td><td>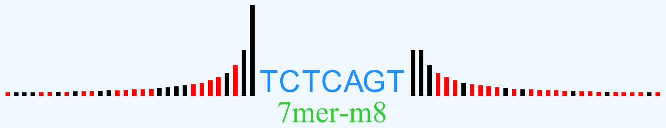</td><td>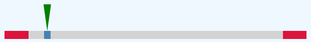</td><td></td><td>M T</td></tr>
<tr><td>83 7mer-m8 104<br>5'-atACTCCTTGGTTA-TCTCAGTt-3' UTR<br>||| | |: ::| |||||||<br>3'-acUGACG-AUGGGUAAGAGUCAu-5' miRNA<br>16 15 14 13 7 6 5 4 3 2<br>3'pairing Seed</td><td>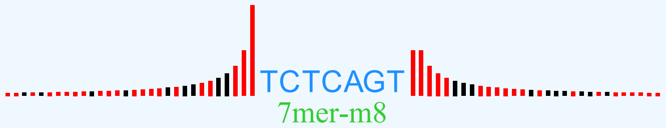</td><td>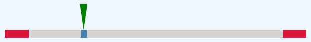</td><td></td><td>M T</td></tr>
</table>

| 2D Structure | Local AU | Position | Conservation | Predicted By |
|---|---|---|---|---|
| Offset 6mer<br>171 193<br>5'-gaGTGTGCGCATGCGCACACAga-3' UTR<br>3'-aaUGUGUGUAUGUGUGUGUGUau-5' miRNA<br>3'pairing Seed | CACACA Offset 6mer | | | M |
| Imperfect match<br>5496 5520<br>5'-aaACATGACAATGTAAACATACATa -3' UTR<br>3'-aaUGUG-UG-UAUGUGUGUGUGUAu-5' miRNA<br>3'pairing Seed | ATACAT Imperfect | | | M |
| Imperfect match<br>6810 6832<br>5'-atgtgtgggaGCACACGCACATg -3' UTR<br>3'-aauguguguauGUGUGUGUGUAu-5' miRNA<br>3'pairing Seed | GCACAT Imperfect | | | M |
| Offset 6mer<br>8366 8388<br>5'-ccACACACACACACACACACAga-3' UTR<br>3'-aaUGUGUGUAUGUGUGUGUGUau-5' miRNA<br>3'pairing Seed | CACACA Offset 6mer | | | M |
| Imperfect match<br>8386 8410<br>5'-agACACACAAACACAGGCAGACATt -3' UTR<br>3'-aaUGUGUGUAUGUGU--GUGUGUAu-5' miRNA<br>3'pairing Seed | AGACAT Imperfect | | | M |
| Offset 6mer<br>11628 11651<br>5'-tatCACATATGTGCAGCACACAgg-3' UTR<br>3'-aauGUGUGUAUGUGU-GUGUGUau-5' miRNA<br>3'pairing Seed | CACACA Offset 6mer | | | M |
| 8mer<br>12728 12750<br>5'-gtATATACTCTCTCTCACACATa-3' UTR<br>3'-aaUGUGUGUAUGUGUGUGUGUAu-5' miRNA<br>3'pairing Seed | CACACATA 8mer | | | M T |
| 7mer-m8<br>14884 14906<br>5'-cacCACACACACACACACACATt-3' UTR<br>3'-aauGUGUGUAUGUGUGUGUGUAu-5' miRNA<br>3'pairing Seed | CACACAT 7mer-m8 | | | M T |
| Imperfect match<br>15004 15026<br>5'-atATATATATACATACATACATa -3' UTR<br>3'-aaUGUGUGUAUGUGUGUGUGUAu-5' miRNA<br>3'pairing Seed | ATACAT Imperfect | | | M |
| 6mer<br>15020 15042<br>5'-atACATATATATATATACACATt-3' UTR<br>3'-aaUGUGUGUAUGUGUGUGUGUAu-5' miRNA<br>3'pairing Seed | ACACAT 6mer | | | M |
| Offset 6mer<br>18309 18330<br>5'-tgtaATACA-AAATGCACACAgg-3' UTR<br>3'-aaugUGUGUAUGUGUGUGUGUau-5' miRNA<br>3'pairing Seed | CACACA Offset 6mer | | | M |
| Offset 6mer<br>18761 18785<br>5'-atGCACACCATGGCGTGCACACACa-3' UTR<br>3'-aaUGUGU-GUA-UGUGUGUGUGUau-5' miRNA<br>3'pairing Seed | CACACA Offset 6mer | | | M |
| Offset 6mer<br>18777 18799<br>5'-gcACACACACTCACACACACAca-3' UTR<br>3'-aaUGUGUGUAUGUGUGUGUGUau-5' miRNA<br>3'pairing Seed | CACACA Offset 6mer | | | M |
| Offset 6mer<br>18962 18984<br>5'-atATATAAACACATACACACAgt-3' UTR<br>3'-aaUGUGUGUAUGUGUGUGUGUau-5' miRNA<br>3'pairing Seed | CACACA Offset 6mer | | | M |
| Imperfect match<br>22561 22583<br>5'-gcGCGCGCATGCATGCATGCATg -3' UTR<br>3'-aaUGUGUGUAUGUGUGUGUGUAu-5' miRNA<br>3'pairing Seed | ATGCAT Imperfect | | | M |
| Imperfect match<br>22577 22599<br>5'-atGCATGCATGCATGCATGCATg -3' UTR<br>3'-aaUGUGUGUAUGUGUGUGUGUAu-5' miRNA<br>3'pairing Seed | ATGCAT Imperfect | | | M |
| Offset 6mer<br>25155 25177<br>5'-ctGCACCCCCACCCCCACACAct-3' UTR<br>3'-aaUGUGUGUAUGUGUGUGUGUau-5' miRNA<br>3'pairing Seed | CACACA Offset 6mer | | | M |
| Offset 6mer<br>26298 26320<br>5'-gtACACATACACACTCACACAga-3' UTR<br>3'-aaUGUGUGUAUGUGUGUGUGUau-5' miRNA<br>3'pairing Seed | CACACA Offset 6mer | | | M |
| Offset 6mer<br>26312 26334<br>5'-tcACACAGAAGCACACACACAgc-3' UTR<br>3'-aaUGUGUGUAUGUGUGUGUGUau-5' miRNA<br>3'pairing Seed | CACACA Offset 6mer | | | M |
| Offset 6mer<br>30098 30121<br>5'-ccATATCACACCTAGACACACAgg-3' UTR<br>3'-aaUGU-GUGUAUGUGUGUGUGUau-5' miRNA<br>3'pairing Seed | CACACA Offset 6mer | | | M |
| Offset 6mer<br>30290 30312<br>5'-ggagagtgggcCACACACACAcc-3' UTR<br>3'-aauguguguauGUGUGUGUGUau-5' miRNA<br>3'pairing Seed | CACACA Offset 6mer | | | M |
| 7mer-m8<br>33690 33712<br>5'-agACACATGTATGCACACACATg-3' UTR<br>3'-aaUGUGUGUAUGUGUGUGUGUAu-5' miRNA<br>3'pairing Seed | CACACAT 7mer-m8 | | | M T |
| Offset 6mer<br>33712 33734<br>5'-gtACACACACACACACACACAca-3' UTR<br>3'-aaUGUGUGUAUGUGUGUGUGUau-5' miRNA<br>3'pairing Seed | CACACA Offset 6mer | | | M |
| Offset 6mer<br>33933 33956<br>5'-tgACGCTTCTAAAGTACACACAcc-3' UTR<br>3'-aaUGUGUGUAUGU-GUGUGUGUau-5' miRNA<br>3'pairing Seed | CACACA Offset 6mer | | | M |
| 7mer-A1<br>35696 35718<br>5'-tatCATATATACATATACACATa-3' UTR<br>3'-aauGUGUGUAUGUGUGUGUGUAu-5' miRNA<br>3'pairing Seed | TACACATA 7mer-A1 | | | M T |
| Offset 6mer<br>35712 35734<br>5'-acACATATATATTTACACACAct-3' UTR<br>3'-aaUGUGUGUAUGUGUGUGUGUau-5' miRNA<br>3'pairing Seed | CACACA Offset 6mer | | | M |
| Imperfect match<br>35740 35762<br>5'-atATGCATAAACATACATACATc -3' UTR<br>3'-aaUGUGUGUAUGUGUGUGUGUAu-5' miRNA<br>3'pairing Seed | ATACAT Imperfect | | | M |
| Imperfect match<br>36464 36486<br>5'-gtGTACTCACGTATTCACATATa -3' UTR<br>3'-aaUGUGUGUAUGUGUGUGUGUAu-5' miRNA<br>3'pairing Seed | ACATAT Imperfect | | | M |

<table>
<tr><th>2D Structure</th><th>Local AU</th><th>Position</th><th>Conservation</th><th>Predicted By</th></tr>
<tr><td>289 7mer-m8 308<br>5'-tgTGGAGCGG--CCTCAGCTGc-3' UTR<br>::||| ||  |||||||||<br>3'-cuGUCUCCCCUUGGAGUCGACu-5' miRNA<br>16 15 14 13 7 6 5 4 3 2<br>3'pairing Seed</td><td>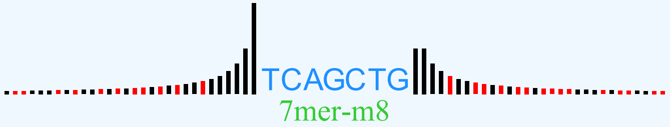<br></td><td>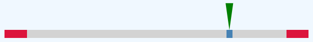</td><td>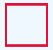</td><td>M T</td></tr>
</table>

| 2D Structure | Local AU | Position | Conservation | Predicted By |
| --- | --- | --- | --- | --- |
| 174 7mer-m8 198<br>5'-gcAAGACAGACCAGCTTGTGTCCTt-3' UTR<br>\|\|\| \|\|\|\|\|\| \|\|\|\|\|\|\|\|<br>3'-ucUUC-AUCUGGU---ACACAGGAu-5' miRNA<br>16 15 14 13 7 6 5 4 3 2<br>3'pairing Seed | 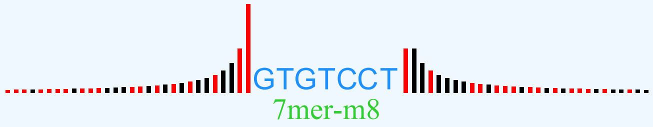 | 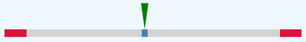 | | M T |

| 2D Structure | Local AU | Position | Conservation | Predicted By |
|---|---|---|---|---|
| 427 8mer 447<br>5'-gaactGACATTAGAGGTGGGa-3' UTR<br>3'-gacucCUCUCUUCUCCACCCc-5' miRNA<br>16 15 14 13 7 6 5 4 3 2<br>3'pairing Seed | 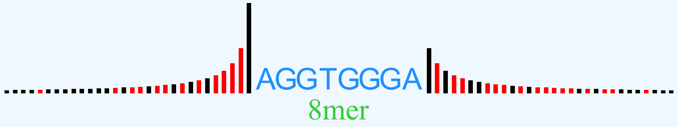 | 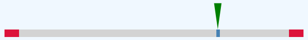 | | M T |
| 503 8mer 525<br>5'-gcaAGCATGGATTTCAGGTGGGa-3' UTR<br>3'-gacUCCUCUCU--UCUCCACCCc-5' miRNA<br>16 15 14 13 7 6 5 4 3 2<br>3'pairing Seed | 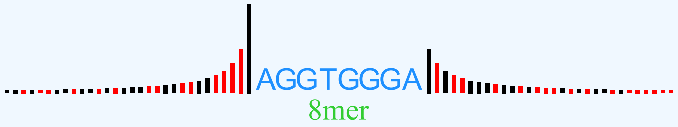 | 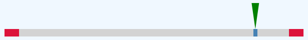 | | M T |

| 2D Structure | Local AU | Position | Conservation | Predicted By |
| --- | --- | --- | --- | --- |
| 169 8mer 188<br>5'-ttgATTATGAT--CCGAATGAa-3' UTR<br>\|\|: \|\|: \|\|\|\|\|\|\|\|<br>3'-augUAGACCUGUCGGCUUACUu-5' miRNA<br>16 15 14 13 7 6 5 4 3 2<br>3'pairing Seed | 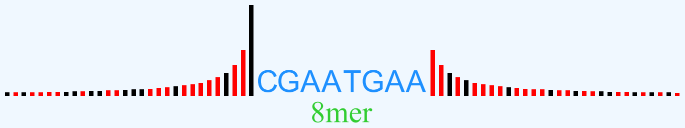 | 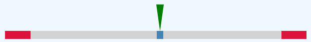 | | M T |
| 305 8mer 326<br>5'-tgTGTTTGAAATGCCGAATGAa-3' UTR<br>::\|:\|\| \| \|\|\|\|\|\|\|\|\|<br>3'-auGUAGACCUGUCGGCUUACUu-5' miRNA<br>16 15 14 13 7 6 5 4 3 2<br>3'pairing Seed | 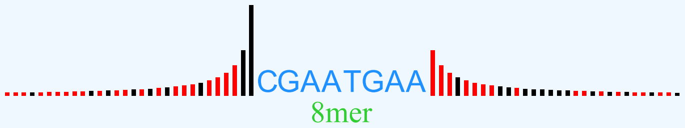 | 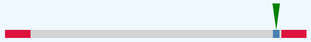 | | M T |

| 2D Structure | Local AU | Position | Conservation | Predicted By |
|---|---|---|---|---|
| Offset 6mer<br>185 207<br>5'-caCCTCCTTGCCAATCCCCAAcc-3' UTR<br>3'-uaGGAGGGA-GGGCAGGGGUUua-5' miRNA<br>16 15 14 13 7 6 5 4 3 2<br>3'pairing Seed | CCCCAA<br>Offset 6mer | | | M |
| Imperfect match<br>482 504<br>5'-caCATCCACTGCCACCTCCAAAa -3' UTR<br>3'-uaGGAGG-GAGGGCAGGGGUUUa-5' miRNA<br>16 15 14 13 7 6 5 4 3 2<br>3'pairing Seed | TCCAAA<br>Imperfect | | | M |
| Imperfect match<br>939 960<br>5'-cttaTCCATGCTGACCTCAAAc -3' UTR<br>3'-uaggAGGGAGGGCAGGGGUUUa-5' miRNA<br>16 15 14 13 7 6 5 4 3 2<br>3'pairing Seed | CTCAAA<br>Imperfect | | | M |

| 2D Structure | Local AU | Position | Conservation | Predicted By |
|---|---|---|---|---|
| 620 7mer-m8 642<br>5'-gagacaaaaaACAAGTCAGAGAg-3' UTR<br>3'-gaggaguggg UGGU-AGUCUCUc-5' miRNA<br>16 15 14 13 7 6 5 4 3 2<br>3'pairing Seed | 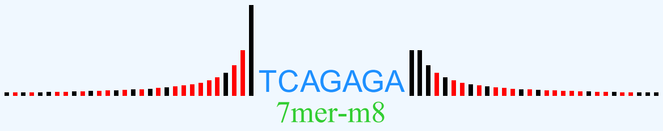 | 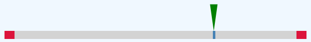 | | M T |
| 863 7mer-m8 883<br>5'-agaagTACCTGCT-TCAGAGAc-3' UTR<br>3'-gaggaGUGGGUGGUAGUCUCUc-5' miRNA<br>16 15 14 13 7 6 5 4 3 2<br>3'pairing Seed | 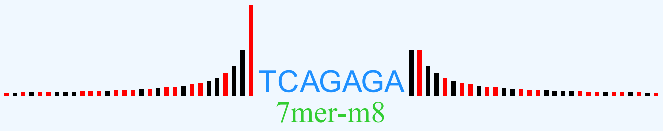 | 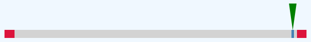 | | M T |

| 2D Structure | Local AU | Position | Conservation | Predicted By |
|---|---|---|---|---|
| 64 7mer-m8 85<br>5'-taaaTTTGGACCAGGAAGAAGt-3' UTR<br>\|: \|\|\| \|:\|\|\|\|\|\|\|<br>3'-acagAGUCCUUCUUCUUCUUCc-5' miRNA<br>16 15 14 13 7 6 5 4 3 2<br>3'pairing Seed | 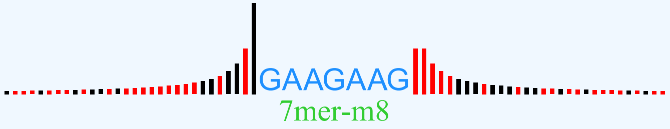 | 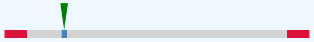 | | M T |

| 2D Structure | Local AU | Position | Conservation | Predicted By |
|---|---|---|---|---|
| Imperfect match<br>365 386<br>5'-gccCTTACCAAGATGAAGAGGt-3' UTR<br>\|\|:\| \|\|\|\| \|\|\|\|\|:\|<br>3'-acaGAGUCCUUCUUCUUCUUCc-5' miRNA<br>16 15 14 13 7 6 5 4 3 2<br>3'pairing Seed | 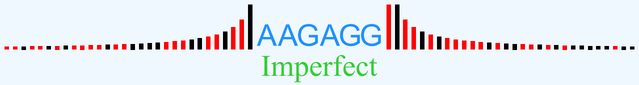  | 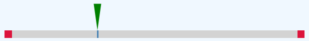 | | M |
| Imperfect match<br>977 998<br>5'-caTCCCAGAAAAAAGAAGAGCa-3' UTR<br>\|\| \|\|\| \|\| \|\|\|\|\|\|\|:<br>3'-acAGAGUCCUUCUUCUUCUUcc-5' miRNA<br>16 15 14 13 7 6 5 4 3 2<br>3'pairing Seed | 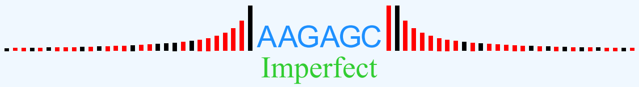  | 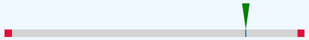 | | M |
| 8mer<br>1136 1157<br>5'-aaaaTGAAGAGGAGGAAGAAGa-3' UTR<br>\| \| \|\|:\|\|:\|\|\|\|\|\|\|<br>3'-acagAGUCCUUCUUCUUCUUCc-5' miRNA<br>16 15 14 13 7 6 5 4 3 2<br>3'pairing Seed | 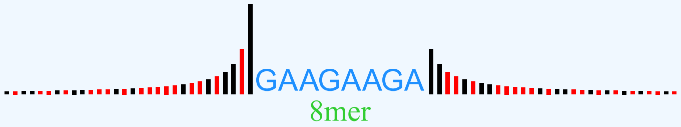  | 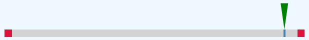 | | M T |

| 2D Structure | Local AU | Position | Conservation | Predicted By |
| --- | --- | --- | --- | --- |
| Imperfect match<br>126 147<br>5'-ggctgaAGTGTGGAGAGGAAGt -3' UTR<br>|| : |:|||:||||<br>3'-acagagUCCUUCUUCUUCUUCc-5' miRNA<br>16 15 14 13 7 6 5 4 3 2<br>3'pairing Seed | 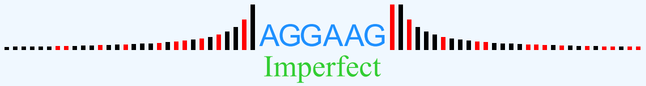 | 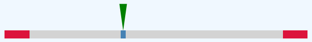 | | M |
| 7mer-m8<br>200 218<br>5'-ggcCTC--GCAG-AGAAGAAGt-3' UTR<br>||| | || ||||||||<br>3'-acaGAGUCCUUCUUCUUCUUCc-5' miRNA<br>16 15 14 13 7 6 5 4 3 2<br>3'pairing Seed | 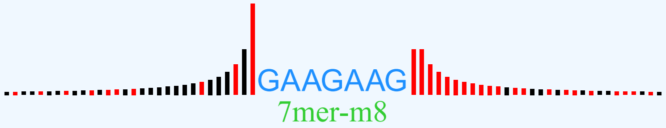 | 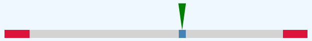 | | M T |

| 2D Structure | Local AU | Position | Conservation | Predicted By |
|---|---|---|---|---|
| Imperfect match<br>92 112<br>5'-ctTCACTGGGAG-TGAAGAGGa -3' UTR<br>\|\| \| \|\|:\|\| \|\|\|\|\|:\|<br>3'-acAGAGUCCUUCUUCUUCUUCc-5' miRNA<br>16 15 14 13 7 6 5 4 3 2<br>3'pairing Seed | 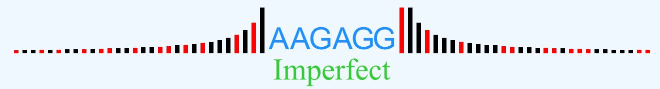<br> | 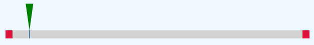 | | M |
| 7mer-m8<br>1055 1074<br>5'-aagCTCAGGA--ATGAAGAAGg-3' UTR<br>\|\|\|\|\|\|\| \| \|\|\|\|\|\|\|<br>3'-acaGAGUCCUUCUUCUUCUUCc-5' miRNA<br>16 15 14 13 7 6 5 4 3 2<br>3'pairing Seed | 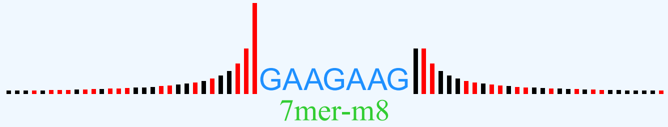<br> | 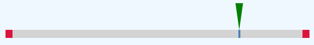 | | M T |
| Imperfect match<br>1315 1336<br>5'-aggaggAGGAGGAAGAGGAAGa -3' UTR<br>\|\|\|\|:\|\|\|\|\|:\|\|\|\|<br>3'-acagagUCCUUCUUCUUCUUCc-5' miRNA<br>16 15 14 13 7 6 5 4 3 2<br>3'pairing Seed | 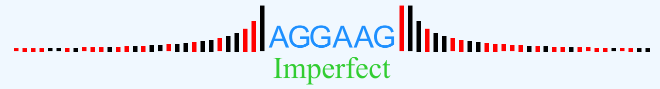<br> | 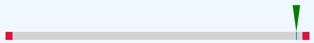 | | M |

| 2D Structure | Local AU | Position | Conservation | Predicted By |
|---|---|---|---|---|
| 260 7mer-m8 279<br>5'-caaagCA-GAAG-AGAAGAAGt-3' UTR<br>\|\| \|\|\|\| \|\|\|\|\|\|\|\|<br>3'-acagaGUCCUUCUUCUUCUUCc-5' miRNA<br>16 15 14 13 7 6 5 4 3 2<br>3'pairing Seed | 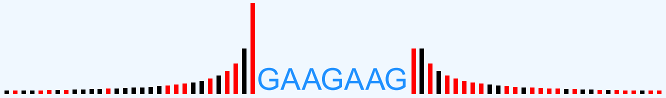 GAAGAAG 7mer-m8 | | | M T |
| Imperfect match<br>298 319<br>5'-agcagatacAAGAGGAGGAAGa -3' UTR<br>\|\|\|\|:\|\|:\|\|\|\|<br>3'-acagaguccUUCUUCUUCUUCc-5' miRNA<br>16 15 14 13 7 6 5 4 3 2<br>3'pairing Seed | 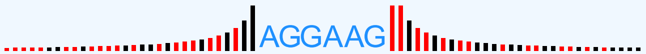 AGGAAG Imperfect | | | M |

| 2D Structure | Local AU | Position | Conservation | Predicted By |
|---|---|---|---|---|
| 76 7mer-m8 97<br>5'-tggCACTGACAACAGAAGAAGg-3' UTR<br>\| \| \| \| \|\|\|\|\|\|\|<br>3'-acaGAGUCCUUCUUCUUCUUCc-5' miRNA<br>16 15 14 13 7 6 5 4 3 2<br>3'pairing Seed | 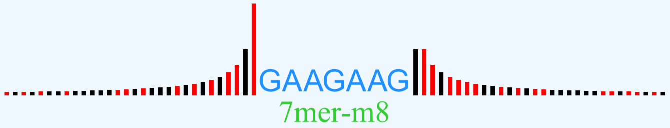 | 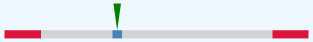 | | M T |
| 157 7mer-m8 178<br>5'-aggtagttGTGGATGAAGAAGc-3' UTR<br>\| : \|\| \|\|\|\|\|\|<br>3'-acagagucCUUCUUCUUCUUCc-5' miRNA<br>16 15 14 13 7 6 5 4 3 2<br>3'pairing Seed | 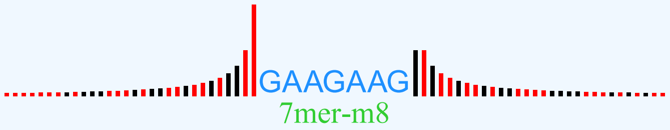 | 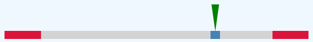 | | M T |

| 2D Structure | Local AU | Position | Conservation | Predicted By |
|---|---|---|---|---|
| 301 7mer-m8 322<br>5'-gttgaCACCTACCATGCTGCTg-3' UTR<br>3'-uggagGUCUAGGAGACGACGAg-5' miRNA<br>16 15 14 13 7 6 5 4 3 2<br>3'pairing Seed | TGCTGCT<br>7mer-m8 | | | M T |
| 351 7mer-m8 373<br>5'-ttTTTCTGACATATTTGCTGCTc-3' UTR<br>3'-ugGAGGUCU-AGGAGACGACGAg-5' miRNA<br>16 15 14 13 7 6 5 4 3 2<br>3'pairing Seed | TGCTGCT<br>7mer-m8 | | | M T |

<table>
<tr><th>2D Structure</th><th>Local AU</th><th>Position</th><th>Conservation</th><th>Predicted By</th></tr>
<tr><td>116 7mer-m8 135<br>5'-ccaagcCATGGA--ATGGAGGg-3' UTR<br>||  |  |  |||||||<br>3'-ugcgacGUCCAUGAUACCUCCa-5' miRNA<br>16 15 14 13 7 6 5 4 3 2<br>3'pairing Seed</td><td>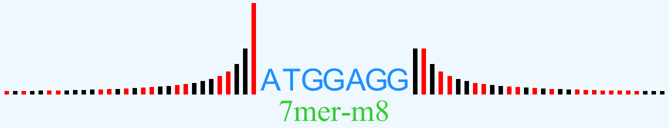
</td><td>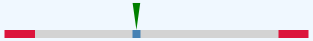</td><td></td><td>M T</td></tr>
<tr><td>Offset 6mer<br>176 197<br>5'-gcctcaCAGGGGTTATGGAGTt-3' UTR<br>||||  ::|||||||<br>3'-ugcgacGUCCAUGAUACCUCca-5' miRNA<br>16 15 14 13 7 6 5 4 3 2<br>3'pairing Seed</td><td>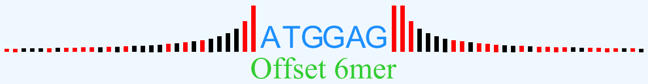
</td><td>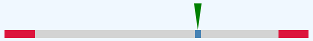</td><td></td><td>M</td></tr>
</table>

| 2D Structure | Local AU | Position | Conservation | Predicted By |
|---|---|---|---|---|
| 58 8mer 85<br>5'-ccAGGAAGCTAAGAGAGGAAGGACCAGa-3' UTR<br>3'-ucUCCU--GCUUC-GUCC--CCUGGUCu-5' miRNA<br>16 15 14 13 7 6 5 4 3 2<br>3'pairing Seed | 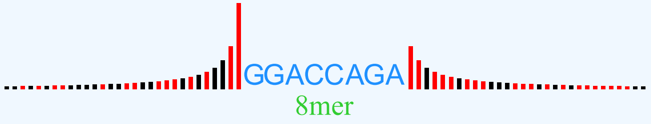 | 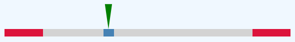 | | M T |
| 171 7mer-m8 192<br>5'-tggcacCGCTGT-GGGGACCAGc-3' UTR<br>3'-ucuccuGCUUCGUCCCCUGGUCu-5' miRNA<br>16 15 14 13 7 6 5 4 3 2<br>3'pairing Seed | 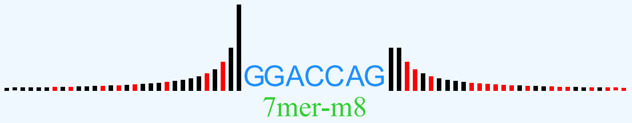 | 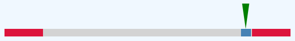 | | M T |

| 2D Structure | Local AU | Position | Conservation | Predicted By |
|---|---|---|---|---|
| 36 7mer-m8 59<br>5'-agtgatTGCTCAAGGACCTCCCTg-3' UTR<br>|||:| ||: |||||||<br>3'-agguccACGGG-UCUAGGAGGGAa-5' miRNA<br>16 15 14 13   7 6 5 4 3 2<br>3'pairing   Seed | 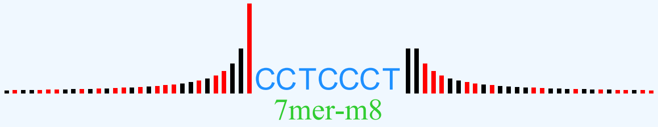 | 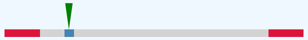 | | M T |

| 2D Structure | Local AU | Position | Conservation | Predicted By |
| --- | --- | --- | --- | --- |
| 56 7mer-m8 79<br>5'-cgagtcgggCGGATGCCTCCCTc-3' UTR<br>\|:\|\|\| \|\|\|\|\|\|\|<br>3'-agguccacggGUCUA-GGAGGGAa-5' miRNA<br>16 15 14 13 7 6 5 4 3 2<br>3'pairing Seed | 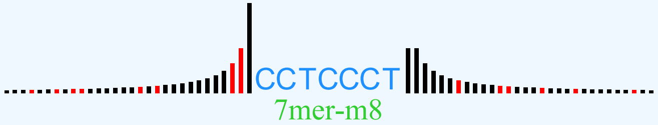 | 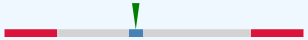 | ☐ | M T |
| 79 7mer-m8 100<br>5'-cgCCGGAG--CGGATGCCTCCCTc-3' UTR<br>\| \|\| \| \|:\|\|\| \|\|\|\|\|\|\|<br>3'-agGUCCACGGGUCUA-GGAGGGAa-5' miRNA<br>16 15 14 13 7 6 5 4 3 2<br>3'pairing Seed | 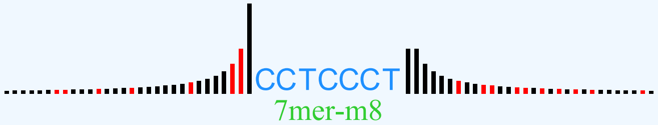 | 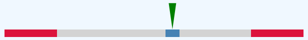 | ☐ | M T |

| 2D Structure | Local AU | Position | Conservation | Predicted By |
| --- | --- | --- | --- | --- |
| see below | 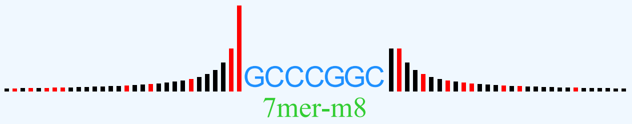 | 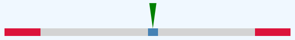 | | M T |

```
101                          7mer-m8  128
  5'-cgcccTG-GTGCCCACCGGTAGCCCGGCc-3'  UTR
     || |||  |||||: | |||||||
  3'-uuuucACACAC-GGUGGUGA-CGGGCCGa-5'  miRNA
              16 15 14 13    7 6 5 4 3 2
           3'pairing          Seed
```

<table>
<tr><th>2D Structure</th><th>Local AU</th><th>Position</th><th>Conservation</th><th>Predicted By</th></tr>
<tr><td><pre>152            7mer-m8  173
   5'-aaAGGCCGCACTGGCTCAGTGg-3'  UTR
         |||   |   ||:|||||||
   3'-ccUCCCUCUCGAUCGAGUCACg-5'  miRNA
            16 15 14 13   7 6 5 4 3 2
         3'pairing     Seed</pre></td><td>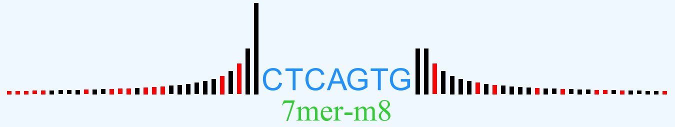
</td><td>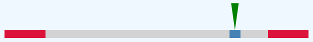</td><td></td><td>M T</td></tr>
</table>

| 2D Structure | Local AU | Position | Conservation | Predicted By |
| --- | --- | --- | --- | --- |
| 123 7mer-m8 144<br>5'-aagtttttCTTTGTCCTGGACt-3' UTR<br>\|\|: \|:\|\|\|\|\|\|\|<br>3'-aggcgacuGAGUCGGGACCUGa-5' miRNA<br>16 15 14 13 7 6 5 4 3 2<br>3'pairing Seed | 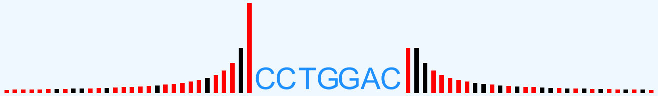  | 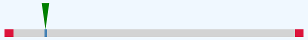 | ☐ | M T |
| 233 7mer-m8 256<br>5'-aagaTTTACTATATGCCCTGGACt-3' UTR<br>:\| \|\|\| :\| \|\|\|\|\|\|\|\|<br>3'-aggcGACUGA-GU-CGGGACCUGa-5' miRNA<br>16 15 14 13 7 6 5 4 3 2<br>3'pairing Seed | 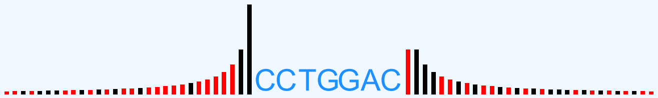  | 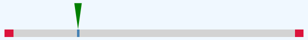 | ☐ | M T |

| 2D Structure | Local AU | Position | Conservation | Predicted By |
|---|---|---|---|---|
| 170 8mer 191<br>5'-tctagagtCATCATCTTTCCTa-3' UTR<br>3'-ugaaugacGAAGGUGAAAGGAu-5' miRNA<br>16 15 14 13 7 6 5 4 3 2<br>3'pairing Seed | 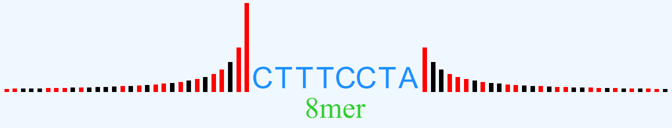 | 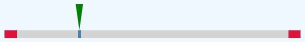 | | M T |
| 366 7mer-m8 389<br>5'-aaTTGCAGCTGTCTTACTTTCCTt-3' UTR<br>3'-ugAAUGACGA-AG-GUGAAAGGAu-5' miRNA<br>16 15 14 13 7 6 5 4 3 2<br>3'pairing Seed | 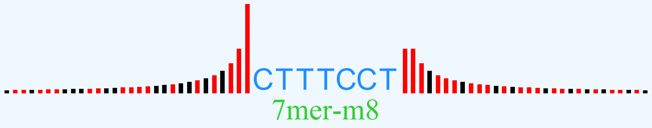 | 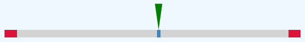 | | M T |

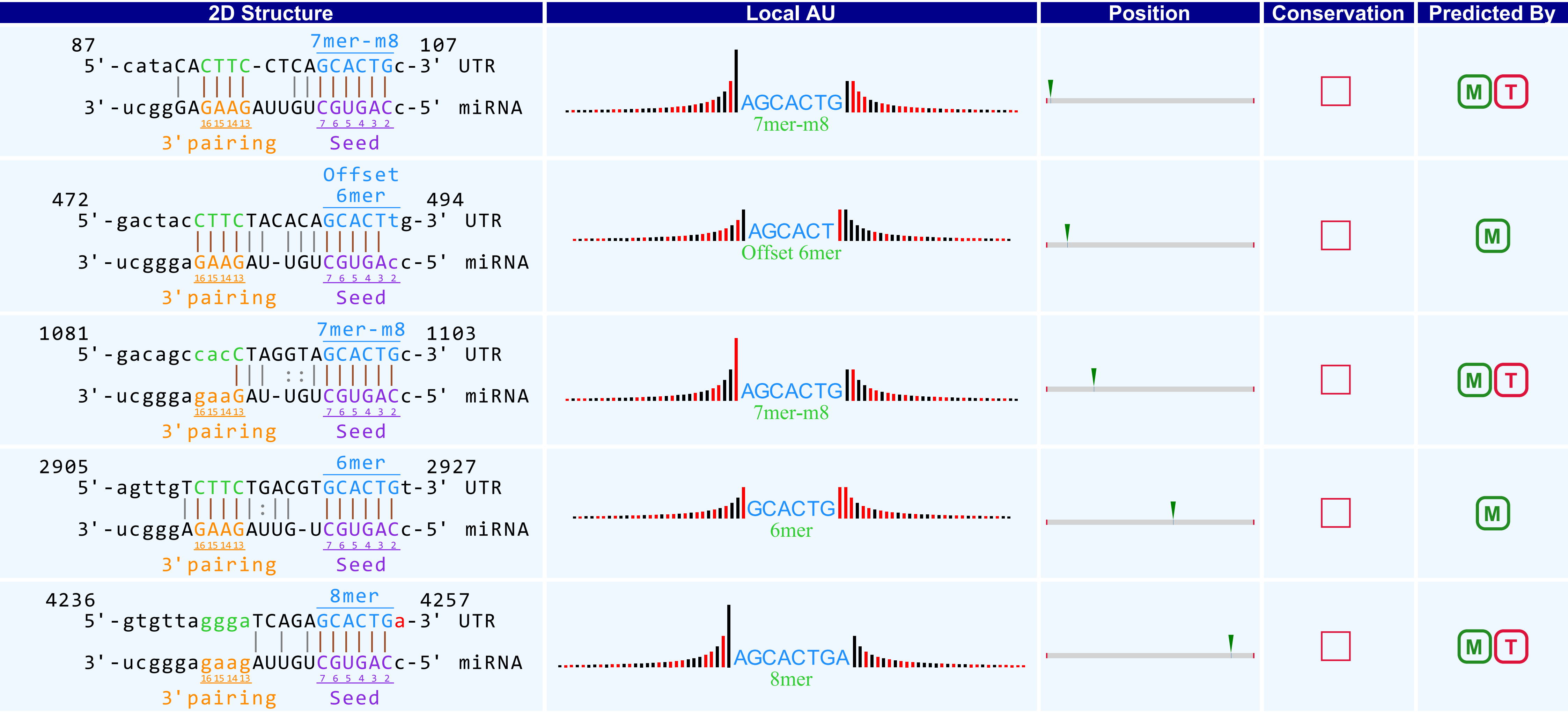
2D Structure
Local AU
Position
Conservation
Predicted By
87
7mer-m8
107
5'-cataCACTTC-CTCAGCACTGc-3' UTR
3'-ucggGAGAAGAUUGUCGUGACc-5' miRNA
16 15 14 13
7 6 5 4 3 2
3'pairing
Seed
AGCACTG
7mer-m8
M
T
Offset
6mer
472
494
5'-gactacCTTCTACACAGCACTtg-3' UTR
3'-ucgggaGAAGAU-UGUCGUGAcc-5' miRNA
16 15 14 13
7 6 5 4 3 2
3'pairing
Seed
AGCACT
Offset 6mer
M
1081
7mer-m8
1103
5'-gacagccacCTAGGTAGCACTGc-3' UTR
3'-ucgggagaaGAU-UGUCGUGACc-5' miRNA
16 15 14 13
7 6 5 4 3 2
3'pairing
Seed
AGCACTG
7mer-m8
M
T
2905
6mer
2927
5'-agttgTCTTCTGACGTGCACTGt-3' UTR
3'-ucgggAGAAGAUUG-UCGUGACc-5' miRNA
16 15 14 13
7 6 5 4 3 2
3'pairing
Seed
GCACTG
6mer
M
4236
8mer
4257
5'-gtgttagggaTCAGAGCACTGa-3' UTR
3'-ucgggagaagAUUGUCGUGACc-5' miRNA
16 15 14 13
7 6 5 4 3 2
3'pairing
Seed
AGCACTGA
8mer
M
T

| 2D Structure | Local AU | Position | Conservation | Predicted By |
|---|---|---|---|---|
| 247 7mer-m8 272<br>5'-tcaaCCCTACTGTTACTCGCTCCCTc-3' UTR<br>3'-cugaGAGAGGAC-UCGGUCGAGGGAa-5' miRNA<br>16 15 14 13 7 6 5 4 3 2<br>3'pairing Seed | 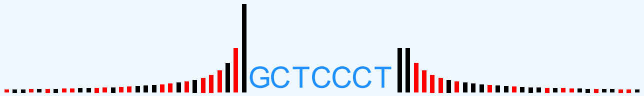<br>GCTCCCT<br>7mer-m8 | | | M T |
| Imperfect match<br>276 301<br>5'-tacagcgTCCTGCGAGATGCTCCTTc-3' UTR<br>3'-cugagagAGGACUCGGU-CGAGGGAa-5' miRNA<br>16 15 14 13 7 6 5 4 3 2<br>3'pairing Seed | 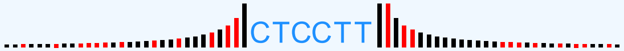<br>CTCCTT<br>Imperfect | | | M |
| Imperfect match<br>381 406<br>5'-gtgaCTCTCCTGGATCTAGTTCCTTt-3' UTR<br>3'-cugaGAGAGGA-CUCGGUCGAGGGAa-5' miRNA<br>16 15 14 13 7 6 5 4 3 2<br>3'pairing Seed | 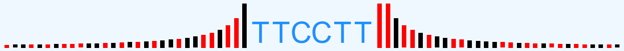<br>TTCCTT<br>Imperfect | | | M |

<table>
<tr><th>2D Structure</th><th>Local AU</th><th>Position</th><th>Conservation</th><th>Predicted By</th></tr>
<tr><td>23 7mer-m8 43<br>5'-atggaattCTGTGGAGGTGGc-3' UTR<br>|||::|||||||<br>3'-gaauugucGACGUCUCCACCa-5' miRNA<br>16 15 14 13 7 6 5 4 3 2<br>3'pairing Seed</td><td>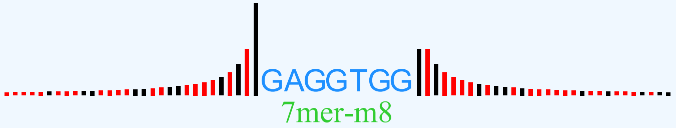</td><td>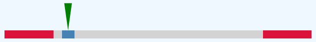</td><td></td><td>M T</td></tr>
</table>

<table>
<tr><th>2D Structure</th><th>Local AU</th><th>Position</th><th>Conservation</th><th>Predicted By</th></tr>
<tr><td>732 7mer-m8 754<br>5'-tgcCTACTAGTTCCT--CAGCTGCt-3' UTR<br>|:||:| |:| | |||||||<br>3'-ucaGGUGGU-AGGAAUUGUCGACGu-5' miRNA<br>16 15 14 13 7 6 5 4 3 2<br>3'pairing Seed</td><td>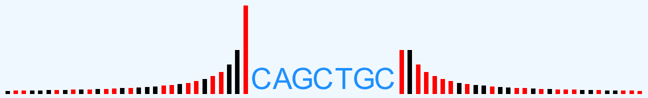<br>CAGCTGC<br>7mer-m8</td><td>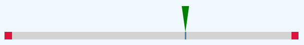</td><td>☐</td><td>M T</td></tr>
<tr><td>900 7mer-m8 921<br>5'-ttTCTATGAAAC--ATCAGCTGCc-3' UTR<br>||:|: | | | |||||||<br>3'-ucAGGUGGUAGGAAUUGUCGACGu-5' miRNA<br>16 15 14 13 7 6 5 4 3 2<br>3'pairing Seed</td><td>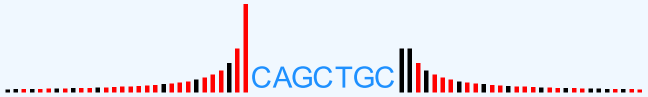<br>CAGCTGC<br>7mer-m8</td><td>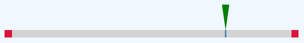</td><td>☐</td><td>M T</td></tr>
<tr><td>1045 8mer 1069<br>5'-tggCTTTTCTCAGTGTACAGCTGCa-3' UTR<br>|: :: || |: ||||||||<br>3'-ucaGGUGGUAGGAAU-UGUCGACGu-5' miRNA<br>16 15 14 13 7 6 5 4 3 2<br>3'pairing Seed</td><td>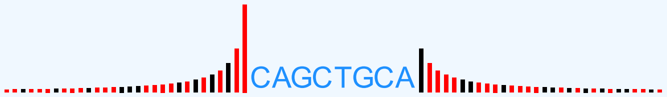<br>CAGCTGCA<br>8mer</td><td>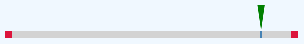</td><td>☐</td><td>M T</td></tr>
</table>

<table>
<tr><th>2D Structure</th><th>Local AU</th><th>Position</th><th>Conservation</th><th>Predicted By</th></tr>
<tr><td>110 8mer 133<br>5'-ttaCGATGGGCTTGAGCAGCTGCa-3' UTR<br>|  |:  :  |:|  |:||||||<br>3'-ucaGGUGGUAGGAAUUGUCGACGu-5' miRNA<br>16 15 14 13 7 6 5 4 3 2<br>3'pairing Seed</td><td>CAGCTGCA<br>8mer</td><td></td><td></td><td>M T</td></tr>
<tr><td>362 Offset 6mer 385<br>5'-caTCCA-GATCCCTAGGCAGCTGgg-3' UTR<br>||||  ||||  ||  :||||||<br>3'-ucAGGUGGUAGGAAU-UGUCGACgu-5' miRNA<br>16 15 14 13 7 6 5 4 3 2<br>3'pairing Seed</td><td>CAGCTG<br>Offset 6mer</td><td></td><td></td><td>M</td></tr>
<tr><td>418 7mer-m8 438<br>5'-agcCCAGTGTCC---GCAGCTGCt-3' UTR<br>|||  ::|||  :|||||||<br>3'-ucaGGUGGUAGGAAUUGUCGACGu-5' miRNA<br>16 15 14 13 7 6 5 4 3 2<br>3'pairing Seed</td><td>CAGCTGC<br>7mer-m8</td><td></td><td></td><td>M T</td></tr>
<tr><td>527 7mer-m8 551<br>5'-ggTTCTGCATCGGGTCGCAGCTGCt-3' UTR<br>|:|  ||||  |  :|||||||<br>3'-ucAGGUGGUAG-GAAUUGUCGACGu-5' miRNA<br>16 15 14 13 7 6 5 4 3 2<br>3'pairing Seed</td><td>CAGCTGC<br>7mer-m8</td><td></td><td></td><td>M T</td></tr>
</table>

| 2D Structure | Local AU | Position | Conservation | Predicted By |
|---|---|---|---|---|
| 110 8mer 133<br>5'-ttaCGATGGGCTTGAGCAGCTGCa-3' UTR<br>\| \|: : \|:\| \|:\|\|\|\|\|\|\|<br>3'-ucaGGUGGUAGGAAUUGUCGACGu-5' miRNA<br>16 15 14 13 7 6 5 4 3 2<br>3'pairing Seed | 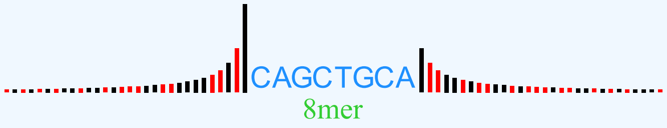 | 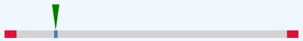 | ☐ | M T |
| 362 Offset 6mer 385<br>5'-caTCCA-GATCCCTAGGCAGCTGgg-3' UTR<br>\|\|\|\| \|\|\|\| \|\| :\|\|\|\|\|\|<br>3'-ucAGGUGGUAGGAAU-UGUCGACgu-5' miRNA<br>16 15 14 13 7 6 5 4 3 2<br>3'pairing Seed | 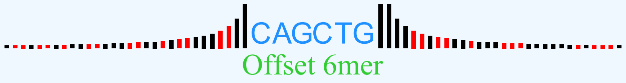 | 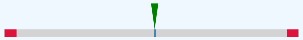 | ☐ | M |
| 418 7mer-m8 438<br>5'-agcCCAGTGTCC---GCAGCTGCt-3' UTR<br>\|\|\| ::\|\|\| :\|\|\|\|\|\|\|<br>3'-ucaGGUGGUAGGAAUUGUCGACGu-5' miRNA<br>16 15 14 13 7 6 5 4 3 2<br>3'pairing Seed | 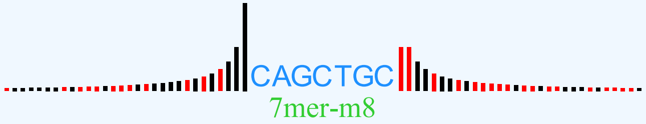 | 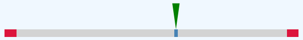 | ☐ | M T |
| 527 7mer-m8 551<br>5'-ggTTCTGCATCGGGTCGCAGCTGCt-3' UTR<br>\|:\| \|\|\|\| \| :\|\|\|\|\|\|\|<br>3'-ucAGGUGGUAG-GAAUUGUCGACGu-5' miRNA<br>16 15 14 13 7 6 5 4 3 2<br>3'pairing Seed | 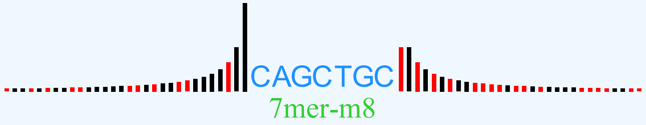 | 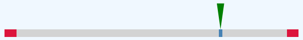 | ☐ | M T |

<table>
<tr><th>2D Structure</th><th>Local AU</th><th>Position</th><th>Conservation</th><th>Predicted By</th></tr>
<tr><td>100 8mer 125<br>5'-acATGTTTATCACATACGGTGACACa-3' UTR<br>| |::|||| | |:|||||||<br>3'-guUUCGGAUAGGG---GUCACUGUGu-5' miRNA<br>16 15 14 13 7 6 5 4 3 2<br>3'pairing Seed</td><td>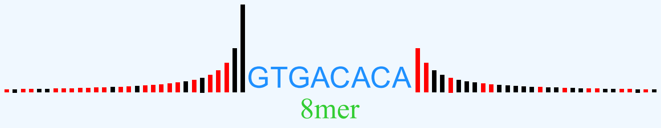
</td><td>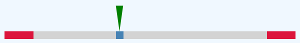</td><td></td><td>M T</td></tr>
<tr><td>245 8mer 268<br>5'-ggAAGCAGCAAGCAAGGTGACACa-3' UTR<br>|||| | | :|||||||<br>3'-guUUCG-GAUAGGGGUCACUGUGu-5' miRNA<br>16 15 14 13 7 6 5 4 3 2<br>3'pairing Seed</td><td>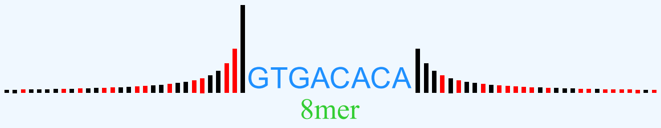
</td><td>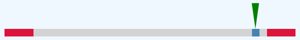</td><td></td><td>M T</td></tr>
</table>

| 2D Structure | Local AU | Position | Conservation | Predicted By |
|---|---|---|---|---|
| 97 7mer-m8 114<br>5'-ctTCGACAG--CCCTCCCAt-3' UTR<br>\|\| \|\|\| \|\|\|\|\|\|\|<br>3'-ggAGGGUCCUGGGAGGGUu-5' miRNA<br>16 15 14 13 7 6 5 4 3 2<br>3'pairing Seed | 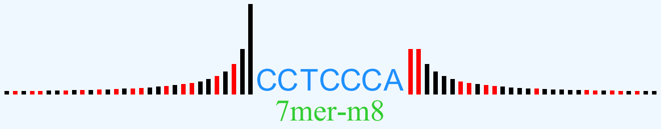 | 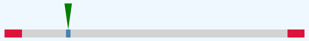 | | M T |
| Imperfect match<br>191 213<br>5'-ccTTCCCAGGCCTTCCCTCTCAt -3' UTR<br>\|:\|\|\|\|\|\| \|\|\|\|\|:\|\|<br>3'-ggAGGGUCC---UGGGAGGGUu-5' miRNA<br>16 15 14 13 7 6 5 4 3 2<br>3'pairing Seed | 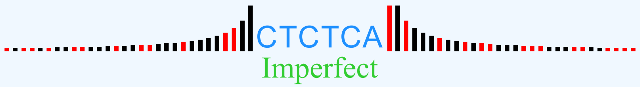 | 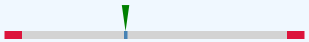 | | M |

| 2D Structure | Local AU | Position | Conservation | Predicted By |
|---|---|---|---|---|
| 88 8mer 109<br>5'-agTGTGGAGTT-GGCCTGTGGGa-3' UTR<br>\|:\| \|\|\|:\| \|\|\|\|\|\|\|\|\|\|<br>3'-cgAUA-CUCGACCCGGACACCCu-5' miRNA<br>16 15 14 13 7 6 5 4 3 2<br>3'pairing Seed | 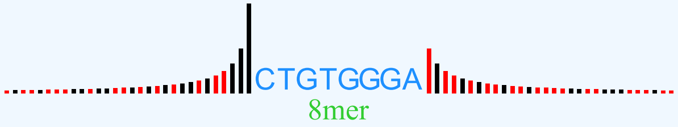 | 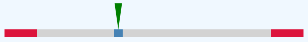 | | M T |

| 2D Structure | Local AU | Position | Conservation | Predicted By |
|---|---|---|---|---|

```
91                     7mer-m8  118
5'-gaTTCCACAGATAACCCAAGGTGGCTGg-3' UTR
     |||  ||| |||  |||   |||||||
3'-agAAG--GUCCAUUCGG---CACCGACu-5' miRNA
             16151413      7 6 5 4 3 2
         3'pairing          Seed
```

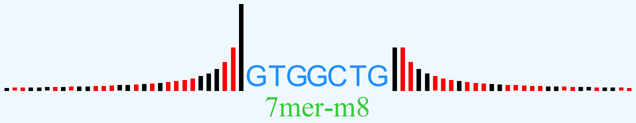


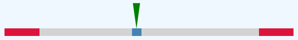

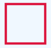

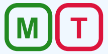

<table>
<tr><th>2D Structure</th><th>Local AU</th><th>Position</th><th>Conservation</th><th>Predicted By</th></tr>
<tr><td>Offset<br>6mer<br>93 114<br>5'-tcCTGCTCTTCGTGGCCCACat-3' UTR<br>||:|: |||::||||||<br>3'-ccGAUGGAAAGUGCCGGGUGac-5' miRNA<br>16 15 14 13 7 6 5 4 3 2<br>3'pairing Seed</td><td>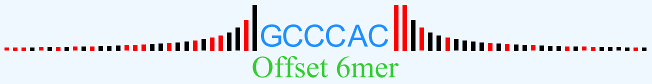</td><td>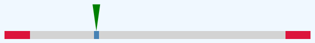</td><td></td><td>M</td></tr>
<tr><td>Imperfect<br>match<br>163 181<br>5'-ccCTAC--TTCAT-GCCCGCTt-3' UTR<br>||||  ||||: |||:||<br>3'-ccGAUGGAAAGUGCCGGGUGAc-5' miRNA<br>16 15 14 13 7 6 5 4 3 2<br>3'pairing Seed</td><td>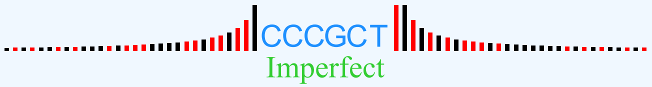</td><td>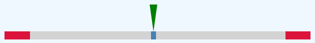</td><td></td><td>M</td></tr>
</table>

<table>
<tr><th>2D Structure</th><th>Local AU</th><th>Position</th><th>Conservation</th><th>Predicted By</th></tr>
<tr><td>140 7mer-m8 161<br>5'-ttctatgccccGTGGTGTGGCc-3' UTR<br>|::|||||||<br>3'-acauuccacaaCGUCACACCGu-5' miRNA<br>16 15 14 13 7 6 5 4 3 2<br>3'pairing Seed</td><td>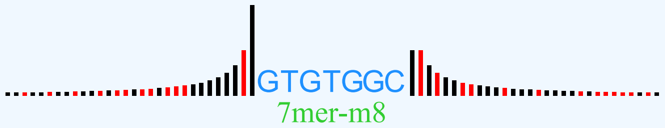</td><td>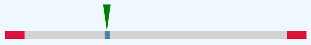</td><td></td><td>M T</td></tr>
<tr><td>212 7mer-A1 232<br>5'-gaTCAGGTGTTGT-TTGTGGCa-3' UTR<br>| |||||||: ||||||<br>3'-acAUUCCACAACGUCACACCGu-5' miRNA<br>16 15 14 13 7 6 5 4 3 2<br>3'pairing Seed</td><td>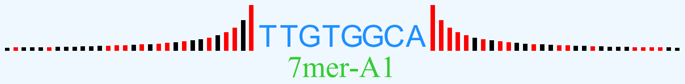</td><td>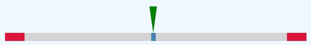</td><td></td><td>M T</td></tr>
</table>

<table>
<tr><th>2D Structure</th><th>Local AU</th><th>Position</th><th>Conservation</th><th>Predicted By</th></tr>
<tr><td>Imperfect match<br>188 209<br>5'-ccctacCACCTGGGCAGTCAGt-3' UTR<br>||||  ::|||:|||<br>3'-aaacguGUGGUGUUGUCGGUCu-5' miRNA<br>16 15 14 13 7 6 5 4 3 2<br>3'pairing Seed</td><td>AGTCAG<br>Imperfect</td><td></td><td></td><td>M</td></tr>
<tr><td>7mer-m8<br>232 253<br>5'-tcTGGGCATCCTGTCAGCCAGc-3' UTR<br>||  :||:|  ::  |||||||<br>3'-aaACGUGUGGUGUUGUCGGUCu-5' miRNA<br>16 15 14 13 7 6 5 4 3 2<br>3'pairing Seed</td><td>CAGCCAG<br>7mer-m8</td><td></td><td></td><td>M T</td></tr>
<tr><td>Offset 6mer<br>321 343<br>5'-aggcCACTATCACTCCAGCCAac-3' UTR<br>|||  |:|||   ||||||<br>3'-aaacGUG-UGGUGUUGUCGGUcu-5' miRNA<br>16 15 14 13 7 6 5 4 3 2<br>3'pairing Seed</td><td>CAGCCA<br>Offset 6mer</td><td></td><td></td><td>M</td></tr>
<tr><td>Offset 6mer<br>830 851<br>5'-cagcTGTGCCACAGCAGCCAcc-3' UTR<br>::::|||||:||||||<br>3'-aaacGUGUGGUGUUGUCGGUcu-5' miRNA<br>16 15 14 13 7 6 5 4 3 2<br>3'pairing Seed</td><td>CAGCCA<br>Offset 6mer</td><td></td><td></td><td>M</td></tr>
</table>

**2D Structure**

```
179                        7mer-m8  202
   5'-caCCAGACAGTCTTCAGGTGCTGc-3'  UTR
          |||  |||:|  |:|  ||||||
   3'-ggGGU-UGUUAUAGGACCACGACu-5'  miRNA
               16151413     7 6 5 4 3 2
            3'pairing       Seed
```

**Local AU**

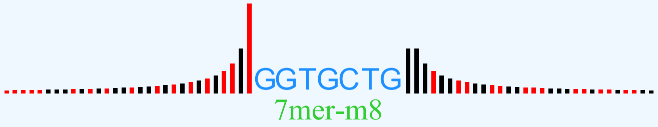


**Position**

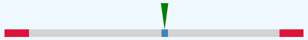

**Conservation**

☐

**Predicted By**

M T

| 2D Structure | Local AU | Position | Conservation | Predicted By |
|---|---|---|---|---|
| 30 7mer-m8 54<br>5'-caGTTAAAGCTATGTTAGCAACCAt-3' UTR<br>3'-gaUGAAUUAGAU---GUCGUUGGUu-5' miRNA<br>16 15 14 13 7 6 5 4 3 2<br>3'pairing Seed | GCAACCA<br>7mer-m8 | | | M T |
| 465 7mer-m8 487<br>5'-ccagcggcgCTCACAGCAACCAg-3' UTR<br>3'-gaugaauuaGA-UGUCGUUGGUu-5' miRNA<br>16 15 14 13 7 6 5 4 3 2<br>3'pairing Seed | GCAACCA<br>7mer-m8 | | | M T |
| 590 7mer-m8 611<br>5'-cctgTTTTCCTGAGCAACCAg-3' UTR<br>3'-gaugAAUUAGAUGUCGUUGGUu-5' miRNA<br>16 15 14 13 7 6 5 4 3 2<br>3'pairing Seed | GCAACCA<br>7mer-m8 | | | M T |

| 2D Structure | Local AU | Position | Conservation | Predicted By |
| --- | --- | --- | --- | --- |
| 250 8mer 271<br>5'-cttgaaacataaaaTTGATGAa-3' UTR<br>|||||||<br>3'-ccugagguaaacaaAACUACUa-5' miRNA<br>16 15 14 13   7 6 5 4 3 2<br>3'pairing Seed | 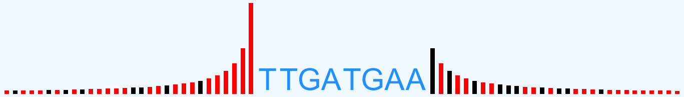 TTGATGAA 8mer | 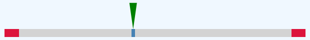 | ☐ | M T |
| 353 7mer-m8 373<br>5'-gaACT-TAGTGGCATTGATGAt-3' UTR<br>||| :| | | |||||||<br>3'-ccUGAGGUAAACAAAACUACUa-5' miRNA<br>16 15 14 13   7 6 5 4 3 2<br>3'pairing Seed | 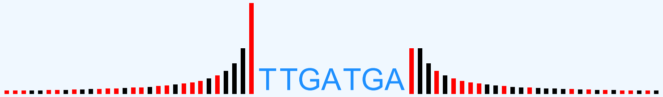 TTGATGA 7mer-m8 | 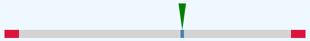 | ☐ | M T |

| 2D Structure | Local AU | Position | Conservation | Predicted By |
|---|---|---|---|---|
| 44 7mer-m8 65<br>5'-ctttaagGATATGCTTTGATGAg-3' UTR<br>3'-ccugaggUAAACAAAACUACUa-5' miRNA<br>16 15 14 13 7 6 5 4 3 2<br>3'pairing Seed | 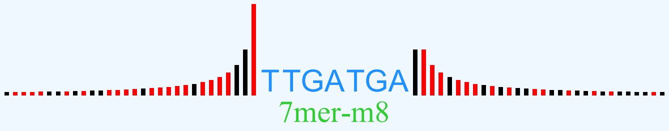 | | | M T |
| Offset 6mer<br>107 128<br>5'-cttCTTCAATGGCTTTGATGca-3' UTR<br>3'-ccuGAGGUAAACAAAACUACua-5' miRNA<br>16 15 14 13 7 6 5 4 3 2<br>3'pairing Seed | 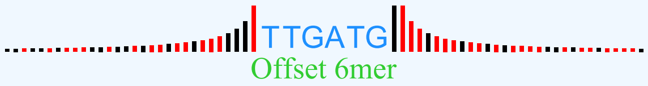 | | | M |

<table>
<tr><th>2D Structure</th><th>Local AU</th><th>Position</th><th>Conservation</th><th>Predicted By</th></tr>
<tr><td>274 Offset 6mer 296<br>5'-tgaTCAGAAACATCTCCCAGCat-3' UTR<br>|| |||| || ||||||||<br>3'-uagAGACUUUAUA-AGGGUCGga-5' miRNA<br>16 15 14 13 7 6 5 4 3 2<br>3'pairing Seed</td><td>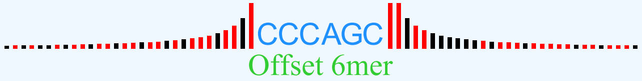
</td><td>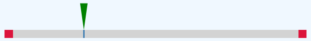</td><td></td><td>M</td></tr>
<tr><td>576 7mer-m8 597<br>5'-aaaTCTGGGATAATCCCAGCCc-3' UTR<br>||||::||| ||||||||<br>3'-uagAGACUUUAUAAGGGUCGGa-5' miRNA<br>16 15 14 13 7 6 5 4 3 2<br>3'pairing Seed</td><td>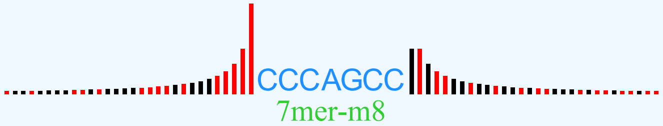
</td><td>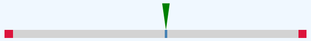</td><td></td><td>M T</td></tr>
</table>

| 2D Structure | Local AU | Position | Conservation | Predicted By |
| --- | --- | --- | --- | --- |
| 19 6mer 42<br>5'-gtTCTGTTGGATATAGTCTTCAAg-3' UTR<br>\|\|\|\|:\|  :\|\|  \|\|::\|\|\|\|\|\|<br>3'-ugAGACGA-UUA-AUUGGAAGUUg-5' miRNA<br>16 15 14 13 7 6 5 4 3 2<br>3'pairing Seed | 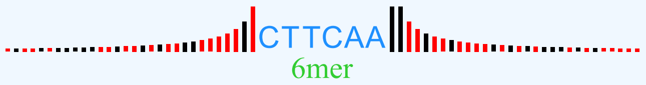 | 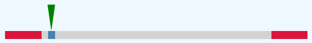 | | M |

<table>
<tr><th>2D Structure</th><th>Local AU</th><th>Position</th><th>Conservation</th><th>Predicted By</th></tr>
<tr><td>52 8mer 73<br>5'-gcctacCTGGTCAGCCTTCAAa-3' UTR<br>||::| |:|||||||<br>3'-ugagacGAUUAAUUGGAAGUUg-5' miRNA<br>16 15 14 13 7 6 5 4 3 2<br>3'pairing Seed</td><td>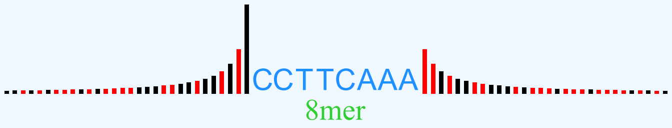<br></td><td></td><td></td><td>M T</td></tr>
<tr><td>355 7mer-m8 378<br>5'-ttTCTGGGTGTGTGCCCCTTCAAg-3' UTR<br>||| | |: :| |||||||<br>3'-ugAGA-CGAU-UAAUUGGAAGUUg-5' miRNA<br>16 15 14 13 7 6 5 4 3 2<br>3'pairing Seed</td><td>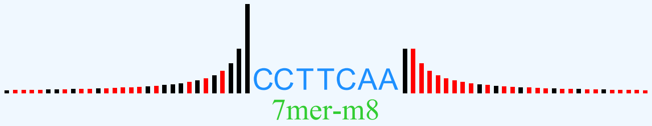<br></td><td></td><td></td><td>M T</td></tr>
</table>

| 2D Structure | Local AU | Position | Conservation | Predicted By |
|---|---|---|---|---|
| 236 7mer-m8 258<br>5'-ggagacactttctctCAGATGCt-3' UTR<br>|||||||<br>3'-ugucgaguccuagucGUCUACGa-5' miRNA<br>16 15 14 13 7 6 5 4 3 2<br>3'pairing Seed | CAGATGC<br>7mer-m8 | | | M T |
| 830 7mer-m8 855<br>5'-agAACTCAGAAACACTTGCAGATGCt-3' UTR<br>| ||||| | || ||||||||<br>3'-ugUCGAGUCCUAGU---CGUCUACGa-5' miRNA<br>16 15 14 13 7 6 5 4 3 2<br>3'pairing Seed | CAGATGC<br>7mer-m8 | | | M T |
| 874 Offset 6mer 897<br>5'-taAGCACTATGGTCAACAGATGaa-3' UTR<br>||| | | |:||| ||||||<br>3'-ugUCGAG-UCCUAGUCGUCUACga-5' miRNA<br>16 15 14 13 7 6 5 4 3 2<br>3'pairing Seed | CAGATG<br>Offset 6mer | | | M |

| 2D Structure | Local AU | Position | Conservation | Predicted By |
|---|---|---|---|---|
| 26 7mer-m8 44<br>5'-accCTAAG--CCTACCCAGAg-3' UTR<br>|| || ||:|||||||<br>3'-cuaGAGUCCGGGGUGGGUCUg-5' miRNA<br>16 15 14 13 7 6 5 4 3 2<br>3'pairing Seed | 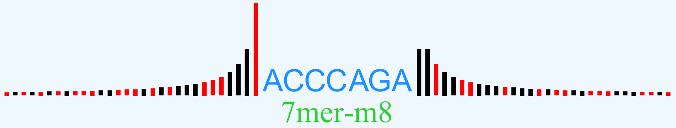 | 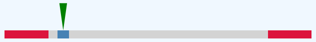 | 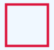 | M T |

| 2D Structure | Local AU | Position | Conservation | Predicted By |
| --- | --- | --- | --- | --- |
| 88 8mer 109<br>5'-gaaacaAGGCTGCTCAGGCCCa-3' UTR<br>\|\|:\|\|\| \|\|\|\|\|\|\|\|<br>3'-cagaacUCUGACCAGUCCGGGc-5' miRNA<br>16 15 14 13 7 6 5 4 3 2<br>3'pairing Seed | 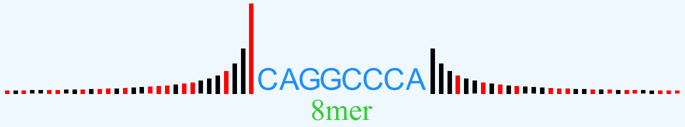<br> | 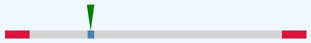 | | M T |

<table>
<tr><th>2D Structure</th><th>Local AU</th><th>Position</th><th>Conservation</th><th>Predicted By</th></tr>
<tr><td>143 8mer 163<br>5'-atcagaaaCCA-GTTGTCCACa-3' UTR<br>||| | |||||||<br>3'-gguuggagGGUGCCACAGGUGu-5' miRNA<br>16 15 14 13 7 6 5 4 3 2<br>3'pairing Seed</td><td>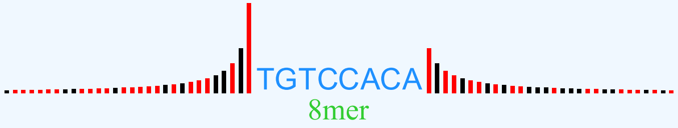</td><td>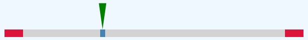</td><td>☐</td><td>M T</td></tr>
<tr><td>290 7mer-m8 311<br>5'-ctcACTTGTC-CGATTGTCCACg-3' UTR<br>||:| :| || |||||||<br>3'-gguUGGAGGGUGC-CACAGGUGu-5' miRNA<br>16 15 14 13 7 6 5 4 3 2<br>3'pairing Seed</td><td>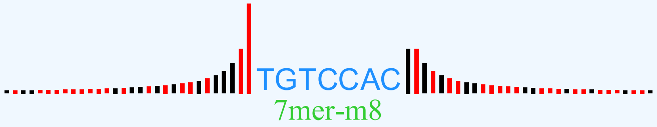</td><td>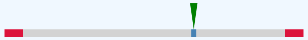</td><td>☐</td><td>M T</td></tr>
</table>

| 2D Structure | Local AU | Position | Conservation | Predicted By |
|---|---|---|---|---|
| 188 8mer 208<br>5'-tcTTTGTAAGTGACCCCAACa-3' UTR<br>\|\|\|: \|\|:\|\| \|\|\|\|\|\|\|<br>3'-cgAAAUUUUUACAGGGGUUGu-5' miRNA<br>16 15 14 13 7 6 5 4 3 2<br>3'pairing Seed | 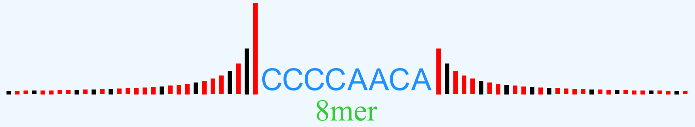 | 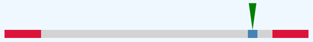 | | M T |

| 2D Structure | Local AU | Position | Conservation | Predicted By |
|---|---|---|---|---|
| Imperfect match<br>226 249<br>5'-tggaggAAAGATGACGGAGAGCTg-3' UTR<br>3'-ucacagUGUCUACU--CUUUCGAa-5' miRNA<br>16 15 14 13 7 6 5 4 3 2<br>3'pairing Seed | AGAGCT<br>Imperfect | | | M |
| 7mer-m8<br>340 361<br>5'-gaaaaTATGGTCGGGAAAGCTg-3' UTR<br>3'-ucacaGUGUCUACUCUUUCGAa-5' miRNA<br>16 15 14 13 7 6 5 4 3 2<br>3'pairing Seed | GAAAGCT<br>7mer-m8 | | | M T |

| 2D Structure | Local AU | Position | Conservation | Predicted By |
|---|---|---|---|---|
| 56 7mer-m8 77<br>5'-gtatggATGCCTGAGAAAGCTg-3' UTR<br>\|:: \|\|\|\|\|\|\|\|\|<br>3'-ucacagUGUCUACUCUUUCGAa-5' miRNA<br>16 15 14 13 7 6 5 4 3 2<br>3'pairing Seed | 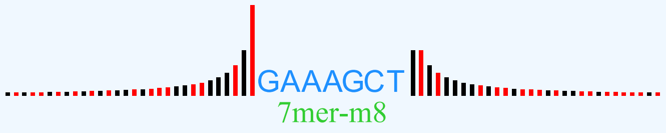 | 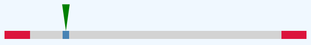 | ☐ | M T |
| 146 7mer-m8 168<br>5'-cgaaatggGGAAGAGGAAAGCTg-3' UTR<br>:\|\| \|\| \|\|\|\|\|\|\|<br>3'-ucacagugUCUACU-CUUUCGAa-5' miRNA<br>16 15 14 13 7 6 5 4 3 2<br>3'pairing Seed | 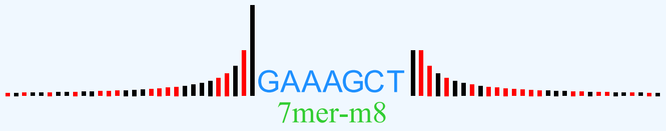 | 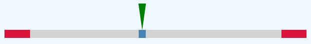 | ☐ | M T |

<table>
<tr><th>2D Structure</th><th>Local AU</th><th>Position</th><th>Conservation</th><th>Predicted By</th></tr>
<tr><td>53 7mer-m8 72<br>5'-ctccgcAGGCA-GCAGCAGAt-3' UTR<br>||||| ||||||||<br>3'-aggauuUCCGUCCGUCGUCUu-5' miRNA<br>16 15 14 13 7 6 5 4 3 2<br>3'pairing Seed</td><td>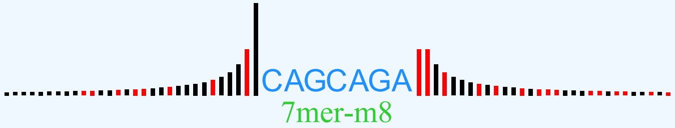</td><td>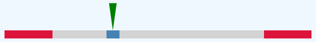</td><td></td><td>M T</td></tr>
</table>

| 2D Structure | Local AU | Position | Conservation | Predicted By |
|---|---|---|---|---|
| Imperfect match<br>170 190<br>5'-tatccgCATGCACCAGAGCTt-3' UTR<br>\|\|\| \|\|\|\|\|:\|\|\|\|<br>3'-uuucugGUAGGUGGUUUCGAu-5' miRNA<br>16 15 14 13 7 6 5 4 3 2<br>3'pairing Seed | 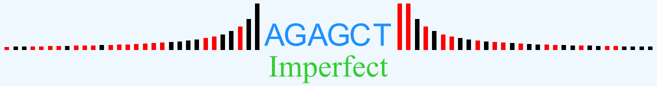 AGAGCT Imperfect | 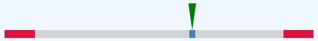 | ☐ | M |
| Imperfect match<br>214 234<br>5'-ttcGGCTTTCCACCAACGCTg-3' UTR<br>\|:\|: \|\|\|\|\|\|\|\| \|\|\|<br>3'-uuuCUGGUAGGUGGUUUCGAu-5' miRNA<br>16 15 14 13 7 6 5 4 3 2<br>3'pairing Seed | 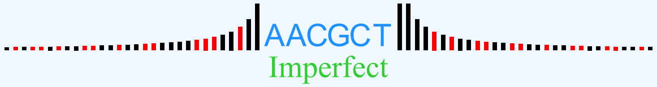 AACGCT Imperfect | 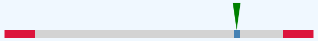 | ☐ | M |

| 2D Structure | Local AU | Position | Conservation | Predicted By |
|---|---|---|---|---|
| Offset 6mer<br>46 65<br>5'-agGATGGCGT-GGGAGTAGtg-3' UTR<br>\| \|\| \|: \|\|\|\|\|\|\|\|<br>3'-acCGACCCCGUCCCUCAUCuc-5' miRNA<br>16 15 14 13 7 6 5 4 3 2<br>3'pairing Seed | 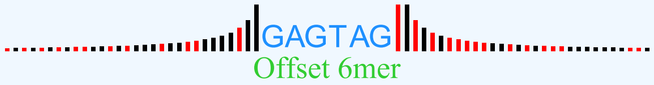 | 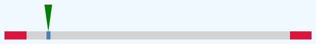 | | M |
| 7mer-m8<br>156 176<br>5'-gggtctcctCATCGAGTAGAt-3' UTR<br>\|\| \|\|\|\|\|\|\|<br>3'-accgaccccGUCCCUCAUCUc-5' miRNA<br>16 15 14 13 7 6 5 4 3 2<br>3'pairing Seed | 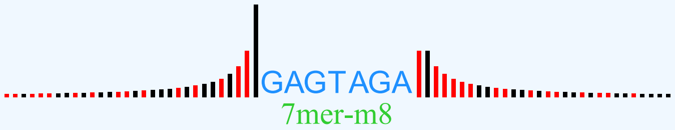 | 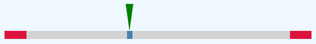 | | M T |

**2D Structure**

```
74                        8mer     97
 5'-cccCCTCACCCCCTCGCCACTCAa-3'  UTR
       ||:||  ||||  ||||||||
 3'-gucGGGGU--GGGACCGGUGAGUg-5'  miRNA
         16 15    14 13   7 6 5 4 3 2
          3'pairing          Seed
```

**Local AU**

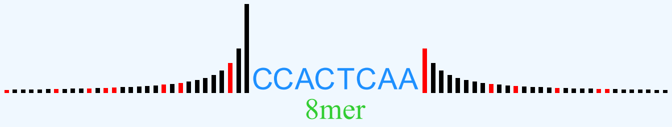


**Position**

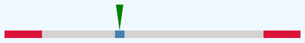

**Conservation**

**Predicted By**

M T

| 2D Structure | Local AU | Position | Conservation | Predicted By |
|---|---|---|---|---|

**Site 1**

```
59                7mer-m8  80
 5'-gtcggGGCGGATGCCTCCCTCg-3'  UTR
         :||:||| ||||||||
 3'-gauugUCGUCUA-GGAGGGAGg-5'  miRNA
         16 15 14 13   7 6 5 4 3 2
      3'pairing       Seed
```

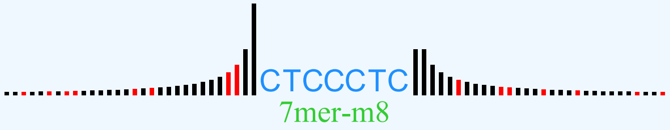


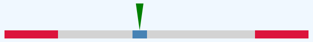

Predicted By: M T

**Site 2**

```
80                7mer-m8  101
 5'-gccggAGCGGATGCCTCCCTCg-3'  UTR
         |||:||| ||||||||
 3'-gauugUCGUCUA-GGAGGGAGg-5'  miRNA
         16 15 14 13   7 6 5 4 3 2
      3'pairing       Seed
```

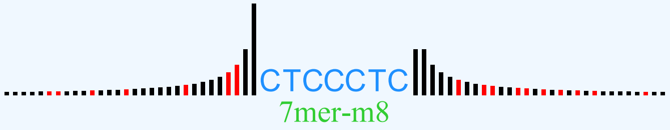


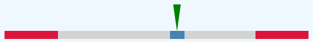

Predicted By: M T

| 2D Structure | Local AU | Position | Conservation | Predicted By |
|---|---|---|---|---|
| 77 7mer-m8 101<br>5'-ctAAGGACTAAGTACCTTAGGCAGg-3' UTR<br>\|\|:\| \|:\| \|\|\| \|\|\|\|\|\|\|<br>3'-ccUUUCGGGUACAU---AUCCGUCg-5' miRNA<br>16 15 14 13 7 6 5 4 3 2<br>3'pairing Seed | 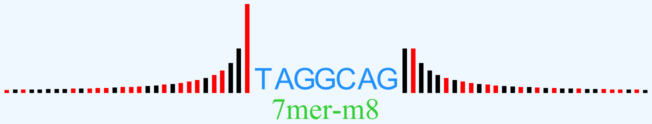 | 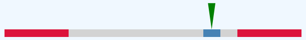 | 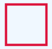 | M T |

| 2D Structure | Local AU | Position | Conservation | Predicted By |
|---|---|---|---|---|
| Imperfect match<br>439 5'-tccTGAAGGAG-TACAAGCTg-3' UTR 458<br>::|||:|| ||||:||<br>3'-ucuGUUUCUUCUCUGUUUGAc-5' miRNA<br>16 15 14 13 7 6 5 4 3 2<br>3'pairing Seed | 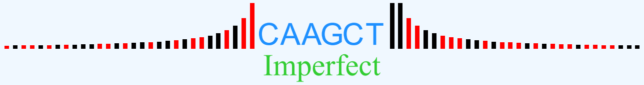 | 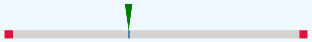 | ☐ | M |
| 8mer<br>462 5'-ccattcctcAAAGACAAACTa-3' UTR 482<br>| |||||||||<br>3'-ucuguuucuUCUCUGUUUGAc-5' miRNA<br>16 15 14 13 7 6 5 4 3 2<br>3'pairing Seed | 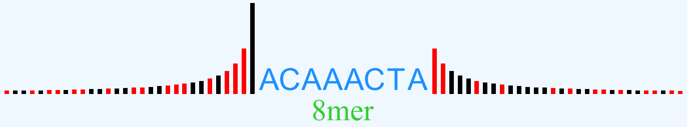 | 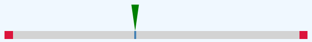 | ☐ | M T |
| Imperfect match<br>701 5'-aatCAAGGAA-ACACAAGCTc-3' UTR 720<br>|||:||| | ||||:||<br>3'-ucuGUUUCUUCUCUGUUUGAc-5' miRNA<br>16 15 14 13 7 6 5 4 3 2<br>3'pairing Seed | 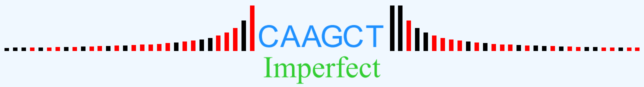 | 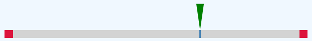 | ☐ | M |

| 2D Structure | Local AU | Position | Conservation | Predicted By |
| --- | --- | --- | --- | --- |
| 114 7mer-m8 137<br>5'-tccTCCTGCT-GCCTATGGCTATTg-3' UTR<br>|||| |: || | |||||||<br>3'-ccaAGGAAGGUCGAAGACCGAUAAu-5' miRNA<br>16 15 14 13 7 6 5 4 3 2<br>3'pairing Seed | 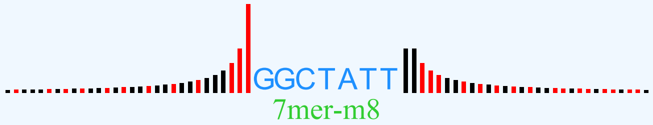 | 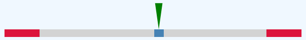 | | M T |

| 2D Structure | Local AU | Position | Conservation | Predicted By |
|---|---|---|---|---|
| 91 7mer-m8 112<br>5'-ttctgcAAGGAACCTGGAGAGg-3' UTR<br>3'-gaauaaUACCCCCGACCUCUCu-5' miRNA<br>16 15 14 13 7 6 5 4 3 2<br>3'pairing Seed | 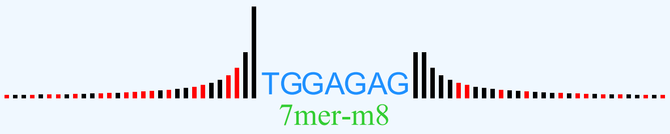 | 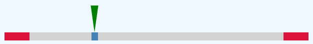 | | M T |
| 122 7mer-m8 143<br>5'-acctggcTGAAGTGTGGAGAGg-3' UTR<br>3'-gaauaauACCCCCGACCUCUCu-5' miRNA<br>16 15 14 13 7 6 5 4 3 2<br>3'pairing Seed | 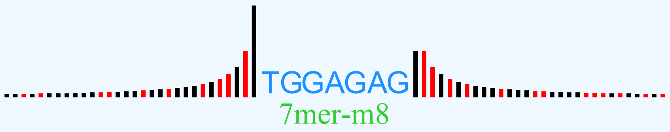 | 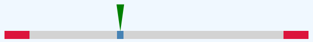 | | M T |

<table>
<tr><th>2D Structure</th><th>Local AU</th><th>Position</th><th>Conservation</th><th>Predicted By</th></tr>
<tr><td>255 7mer-m8 276<br>5'-tccccgatcaaaGCTGGAGAGg-3' UTR<br>|||||||||<br>3'-gaauaauaccccCGACCUCUCu-5' miRNA<br>16 15 14 13 7 6 5 4 3 2<br>3'pairing Seed</td><td>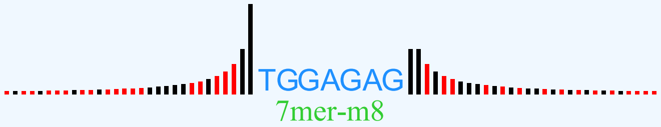
</td><td>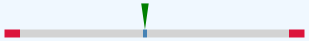</td><td></td><td>M T</td></tr>
<tr><td>Imperfect match<br>355 377<br>5'-caTAATACAGGGGACTGGGGAGa -3' UTR<br>|| || |||| ||||:|||<br>3'-gaAUAAU-ACCCCCGACCUCUCu-5' miRNA<br>16 15 14 13 7 6 5 4 3 2<br>3'pairing Seed</td><td>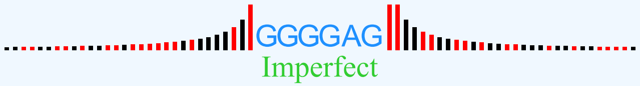
</td><td>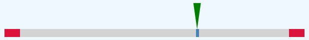</td><td></td><td>M</td></tr>
</table>

<table>
<tr><th>2D Structure</th><th>Local AU</th><th>Position</th><th>Conservation</th><th>Predicted By</th></tr>
<tr><td>139 7mer-m8 161<br>5'-actggaAAGTCTGTTTCCCAGGt-3' UTR<br>| |||| ::|||||||<br>3'-cuuaggUGCAGA-GGAGGGUCCc-5' miRNA<br>16 15 14 13 7 6 5 4 3 2<br>3'pairing Seed</td><td>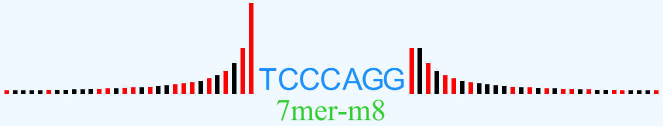
</td><td></td><td></td><td>M T</td></tr>
<tr><td>180 7mer-m8 201<br>5'-actggaACGCACCTTCCCAGGc-3' UTR<br>||| |:|||||||<br>3'-cuuaggUGCAGAGGAGGGUCCc-5' miRNA<br>16 15 14 13 7 6 5 4 3 2<br>3'pairing Seed</td><td>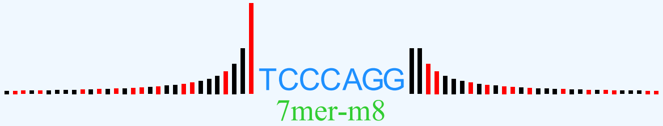
</td><td></td><td></td><td>M T</td></tr>
</table>

| 2D Structure | Local AU | Position | Conservation | Predicted By |
|---|---|---|---|---|
| 23 7mer-m8 44<br>5'-cgcCTTTTG-GGGATGCCTTTCg-3' UTR<br>\|\|::\|: : \|\|\|\|\|\|\|\|\|<br>3'-gucGAGGAUAUACUACGGAAAGa-5' miRNA<br>16 15 14 13 7 6 5 4 3 2<br>3'pairing Seed | GCCTTTC<br>7mer-m8 | | ☐ | M T |
| 851 8mer 871<br>5'-gcaCTCCTGCTT--TGCCTTTCa-3' UTR<br>\|\|\|\|\|: \| \|\|\|\|\|\|\|\|\|<br>3'-gucGAGGAUAUACUACGGAAAGa-5' miRNA<br>16 15 14 13 7 6 5 4 3 2<br>3'pairing Seed | GCCTTTCA<br>8mer | | ☐ | M T |
| 932 7mer-m8 954<br>5'-cagcagagagtggcaGCCTTTCt-3' UTR<br>\|\|\|\|\|\|\|<br>3'-gucgaggauauacuaCGGAAAGa-5' miRNA<br>16 15 14 13 7 6 5 4 3 2<br>3'pairing Seed | GCCTTTC<br>7mer-m8 | | ☐ | M T |

<table>
<tr><th>2D Structure</th><th>Local AU</th><th>Position</th><th>Conservation</th><th>Predicted By</th></tr>
<tr><td>Imperfect match<br>62 84<br>5'-ctaagTTTATTCATCTGATGTAt -3' UTR<br>|:| ||||| |:|||||<br>3'-uaacgAGAAAAGUA-AUUACAUc-5' miRNA<br>16 15 14 13 7 6 5 4 3 2<br>3'pairing Seed</td><td>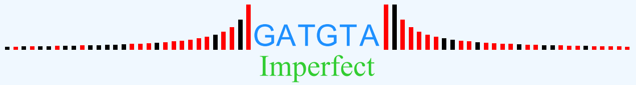</td><td>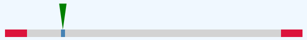</td><td></td><td>M</td></tr>
<tr><td>8mer<br>144 166<br>5'-ttttgcagaaaCATTTAATGTAa-3' UTR<br>|| ||||||||<br>3'-uaacgagaaaaGU-AAUUACAUc-5' miRNA<br>16 15 14 13 7 6 5 4 3 2<br>3'pairing Seed</td><td>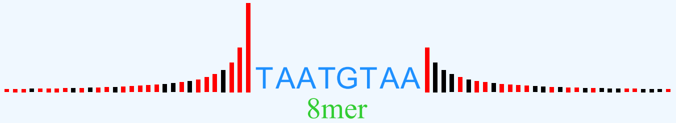</td><td>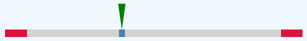</td><td></td><td>M T</td></tr>
</table>

<table>
<tr><th>2D Structure</th><th>Local AU</th><th>Position</th><th>Conservation</th><th>Predicted By</th></tr>
<tr><td>181 7mer-m8 202<br>5'-tgagacaTACGACAGTGGGGAt-3' UTR<br>|||: |||||||<br>3'-agugaccAUGUCCCCACCCCUc-5' miRNA<br>16 15 14 13 7 6 5 4 3 2<br>3'pairing Seed</td><td>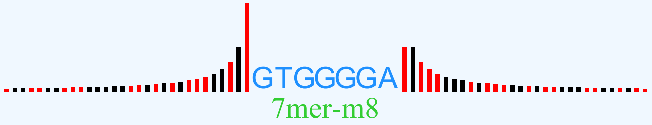</td><td>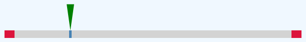</td><td></td><td>M T</td></tr>
<tr><td>279 7mer-m8 303<br>5'-tgtCAGGCTCCAAGGAGGTGGGGAt-3' UTR<br>| || | | ||| |||||||<br>3'-aguGACC-AUG-UCC-CCACCCCUc-5' miRNA<br>16 15 14 13 7 6 5 4 3 2<br>3'pairing Seed</td><td>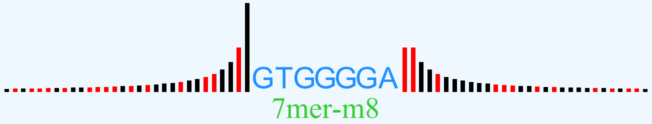</td><td>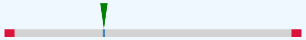</td><td></td><td>M T</td></tr>
<tr><td>636 7mer-m8 657<br>5'-agagaaGTGAAGGCGTGGGGAc-3' UTR<br>||: ||| |||||||<br>3'-agugacCAUGUCCCCACCCCUc-5' miRNA<br>16 15 14 13 7 6 5 4 3 2<br>3'pairing Seed</td><td>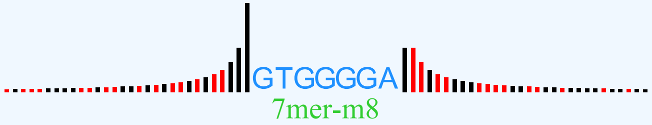</td><td>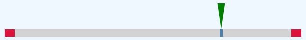</td><td></td><td>M T</td></tr>
</table>

| 2D Structure | Local AU | Position | Conservation | Predicted By |
|---|---|---|---|---|

```
48              7mer-m8  69
5'-taCAGGACATATATCATGTAAc-3'  UTR
     | || | | :|:|||||||
3'-uuGACCCG-AAGUGGUACAUUg-5'  miRNA
       16 15 14 13  7 6 5 4 3 2
      3'pairing        Seed
```

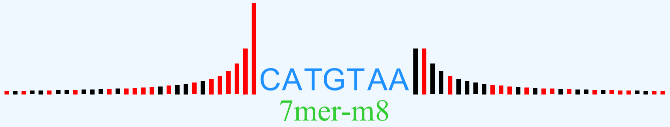


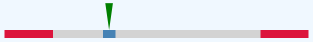

M T

<table>
<tr><th>2D Structure</th><th>Local AU</th><th>Position</th><th>Conservation</th><th>Predicted By</th></tr>
<tr><td>141 7mer-A1 166<br>5'-caTTGC-TCACCATCAGGGTCTCCTCa-3' UTR<br>|| | :||| || ::||||||<br>3'-uuAAGGUGGUGGGAG--AUGGAGGAGg-5' miRNA<br>16 15 14 13 7 6 5 4 3 2<br>3'pairing Seed</td><td>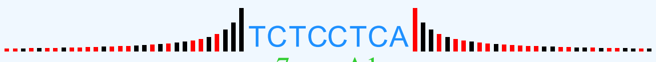</td><td>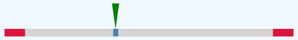</td><td></td><td>M T</td></tr>
<tr><td>201 8mer 223<br>5'-tcgcCTAGTGCCC-CT-CCTCCTCa-3' UTR<br>|:| ::||| || |||||||<br>3'-uuaaGGUGGUGGGAGAUGGAGGAGg-5' miRNA<br>16 15 14 13 7 6 5 4 3 2<br>3'pairing Seed</td><td>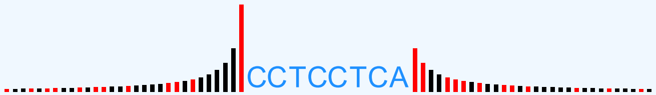</td><td>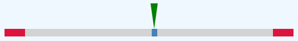</td><td></td><td>M T</td></tr>
</table>

<table>
<tr><th>2D Structure</th><th>Local AU</th><th>Position</th><th>Conservation</th><th>Predicted By</th></tr>
<tr><td>Imperfect match<br>369 392<br>5'-agaTCTGCAACCATTT-CCTTCTCc -3' UTR<br>||::| ||| |:| |||:|||<br>3'-uuaAGGUGGUGGGAGAUGGAGGAGg-5' miRNA<br>16 15 14 13 7 6 5 4 3 2<br>3'pairing Seed</td><td>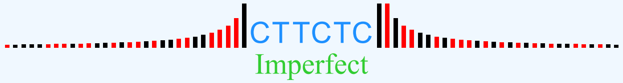
</td><td>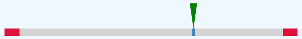</td><td></td><td>M</td></tr>
<tr><td>8mer<br>439 463<br>5'-ggagTCAGTGACTGTGACCTCCTCa-3' UTR<br>:|| :: |: : |||||||<br>3'-uuaaGGUGGUGGGAGAUGGAGGAGg-5' miRNA<br>16 15 14 13 7 6 5 4 3 2<br>3'pairing Seed</td><td>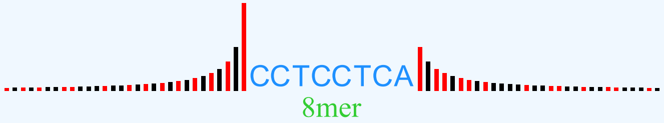
</td><td>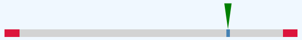</td><td></td><td>M T</td></tr>
</table>

<table>
<tr><th>2D Structure</th><th>Local AU</th><th>Position</th><th>Conservation</th><th>Predicted By</th></tr>
<tr><td>32 6mer 55<br>5'-ccgcCTGTCAGCTTTT-TCTCCTCg-3' UTR<br>|:::|| |:|:| :||||||<br>3'-uuaaGGUGGUGGGAGAUGGAGGAGg-5' miRNA<br>16 15 14 13 7 6 5 4 3 2<br>3'pairing Seed</td><td>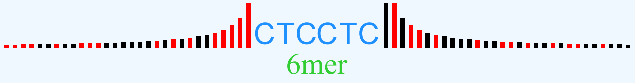</td><td>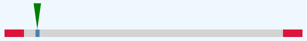</td><td></td><td>M</td></tr>
<tr><td>Imperfect<br>56 match 80<br>5'-agaTCTCCGGTCCTTGACCTCTTCc -3' UTR<br>||: | ::|||: ||||:||<br>3'-uuaAGGUGGUGGGAGAUGGAGGAGg-5' miRNA<br>16 15 14 13 7 6 5 4 3 2<br>3'pairing Seed</td><td>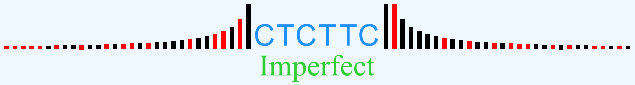</td><td>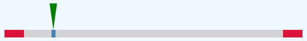</td><td></td><td>M</td></tr>
</table>

<table>
<tr><th>2D Structure</th><th>Local AU</th><th>Position</th><th>Conservation</th><th>Predicted By</th></tr>
<tr><td>8mer<br>116 137<br>5'-gtggtaATCACACT---CCTCCTCa-3' UTR<br>|:|| || |||||||<br>3'-uuaaggUGGUGGGAGAUGGAGGAGg-5' miRNA<br>16 15 14 13 7 6 5 4 3 2<br>3'pairing Seed</td><td>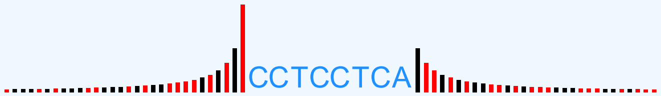</td><td>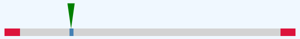</td><td></td><td>M T</td></tr>
<tr><td>Offset<br>6mer<br>379 404<br>5'-ccTTCCATCACCTTGCGACCTCCTaa-3' UTR<br>|||||:||||:| | |||||||<br>3'-uuAAGGUGGUGGGA-GAUGGAGGAgg-5' miRNA<br>16 15 14 13 7 6 5 4 3 2<br>3'pairing Seed</td><td>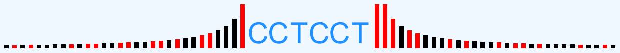</td><td>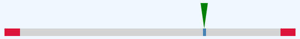</td><td></td><td>M</td></tr>
</table>

| 2D Structure: Start | End | Site type | UTR (5'→3') | miRNA (3'→5') | Local AU | Position | Conservation | Predicted By |
|---|---|---|---|---|---|---|---|---|
| 2350 | 2373 | Imperfect match | 5'-gtcaaaaggCTTTCCGTTCTCTGt-3' UTR | 3'-uguccuagaGAGAGG-AGGAGACa-5' miRNA | | | | M |
| 3274 | 3297 | Imperfect match | 5'-gtcaaaatgCTTTCCGTTCTCTGt-3' UTR | 3'-uguccuagaGAGAGG-AGGAGACa-5' miRNA | | | | M |
| 5472 | 5494 | Imperfect match | 5'-tgGGTGTCTTTTGTTTCTTCTGt-3' UTR | 3'-ugUCCUAGAGAGAGGAGGAGACa-5' miRNA | | | | M |
| 5824 | 5850 | 8mer | 5'-taattATCTCTGAATGTTGTCCTCTGa-3' UTR | 3'-uguccUAGAG---AGAGGAGGAGACa-5' miRNA | | | | M T |
| 6092 | 6114 | Offset 6mer | 5'-catGGATCTGGTTGGTCCTCTTc-3' UTR | 3'-uguCCUAGAGAGAGGAGGAGAca-5' miRNA | | | | M |
| 6104 | 6129 | Imperfect match | 5'-tggtccTCTTCCTCCATCTTCTCTGt-3' UTR | 3'-uguccuAGAGAGAG---GAGGAGACa-5' miRNA | | | | M |
| 6131 | 6155 | Offset 6mer | 5'-cttctGTCTTTCCCCTGTCCTCTct-3' UTR | 3'-uguccUAGAGAGAGG--AGGAGAca-5' miRNA | | | | M |
| 6550 | 6572 | Imperfect match | 5'-ggAGTGACTCTTTTTTTCTCTGa-3' UTR | 3'-ugUCCUAGAGAGAGGAGGAGACa-5' miRNA | | | | M |
| 7487 | 7509 | 7mer-m8 | 5'-ctctttaaTCACAATTCCTCTGt-3' UTR | 3'-uguccuagAGAGAGGAGGAGACa-5' miRNA | | | | M T |
| 7731 | 7752 | Imperfect match | 5'-ggAAGATCT-TTACATCTTCTGa-3' UTR | 3'-ugUCCUAGAGAGAGGAGGAGACa-5' miRNA | | | | M |
| 10362 | 10384 | Imperfect match | 5'-tctctcTCTCTCTCTTTCTCTct-3' UTR | 3'-uguccuAGAGAGAGGAGGAGAca-5' miRNA | | | | M |
| 10376 | 10400 | Imperfect match | 5'-tttctcTCTCTCTCTCTCTCTGt-3' UTR | 3'-uguccuAGAGAGAG-GAG-GAGACa-5' miRNA | | | | M |
| 10571 | 10597 | Imperfect match | 5'-gtAAGATATCTGTCTGAGATCTTCTGg-3' UTR | 3'-ugUCCUAGAGAGAGG---AGGAGACa-5' miRNA | | | | M |
| 11377 | 11400 | Offset 6mer | 5'-aagatATTTATTTCTTTCCTCTTt-3' UTR | 3'-uguccUAGAGAGAG-GAGGAGAca-5' miRNA | | | | M |
| 13322 | 13343 | Imperfect match | 5'-aaAGGA-CATTCCATTTCTCTGt-3' UTR | 3'-ugUCCUAGAGAGAGGAGGAGACa-5' miRNA | | | | M |
| 15515 | 15537 | Imperfect match | 5'-tcaaaATCTGTTAGCTTCTCTGt-3' UTR | 3'-uguccUAGAGAGAGGAGGAGACa-5' miRNA | | | | M |
| 16218 | 16240 | Imperfect match | 5'-ccaattTTCCTATTTTCCTTTGg-3' UTR | 3'-uguccuAGAGAGAGGAGGAGACa-5' miRNA | | | | M |
| 16421 | 16447 | Offset 6mer | 5'-cgGGGATTTCTTTTTCCTTTCCTCTTc-3' UTR | 3'-ugUCCU--AGAGAGAGG--AGGAGAca-5' miRNA | | | | M |
| 16863 | 16886 | Imperfect match | 5'-acAGGATCTGATGTCCTCTTCTGg-3' UTR | 3'-ugUCCUAGA-GAGAGGAGGAGACa-5' miRNA | | | | M |
| 23514 | 23536 | Imperfect match | 5'-ctttcATTCCCCTCTTCTTCTGt-3' UTR | 3'-uguccUAGAGAGAGGAGGAGACa-5' miRNA | | | | M |
| 26504 | 26529 | Imperfect match | 5'-aagtaATCTCGCCCCTCTTCTTCTGt-3' UTR | 3'-uguccUAGA---GAGAGGAGGAGACa-5' miRNA | | | | M |
| 27057 | 27078 | Imperfect match | 5'-gccGGAT-TCCAGTCTCTTCTGc-3' UTR | 3'-uguCCUAGAGAGAGGAGGAGACa-5' miRNA | | | | M |
| 29043 | 29066 | Imperfect match | 5'-ccAGGCGCTGCCCGCCTTCTCTGg-3' UTR | 3'-ugUCCUAGA-GAGAGGAGGAGACa-5' miRNA | | | | M |
| 29180 | 29205 | Offset 6mer | 5'-ctcctGTCTCTCCTATCCTCCTCTTc-3' UTR | 3'-uguccUAGAGAG---AGGAGGAGAca-5' miRNA | | | | M |
| 29226 | 29247 | Imperfect match | 5'-tctctAACT-TTTCATCTTCTGt-3' UTR | 3'-uguccUAGAGAGAGGAGGAGACa-5' miRNA | | | | M |
| 29691 | 29715 | Imperfect match | 5'-ggAGAATCCTCTCTCTCTCCTCGGt-3' UTR | 3'-ugUCCUA-GAGAGAG-GAGGAGACa-5' miRNA | | | | M |
| 33717 | 33739 | Offset 6mer | 5'-cccctAATTTACTCTTCCTCTaa-3' UTR | 3'-uguccUAGAGAGAGGAGGAGAca-5' miRNA | | | | M |
| 34748 | 34771 | Offset 6mer | 5'-caAGGAGCTCTGCCCCTCCTCTtg-3' UTR | 3'-ugUCCUAGAGA-GAGGAGGAGAca-5' miRNA | | | | M |
| 36524 | 36547 | Imperfect match | 5'-attGGATCTGATGTTCTCTTCTGg-3' UTR | 3'-uguCCUAGA-GAGAGGAGGAGACa-5' miRNA | | | | M |
| 41959 | 41981 | Imperfect match | 5'-ttctttaaTTACTCTTTCTCTGt-3' UTR | 3'-uguccuagAGAGAGGAGGAGACa-5' miRNA | | | | M |
| 43731 | 43753 | 7mer-m8 | 5'-agtaaAACTCTCTGTTCCTCTGc-3' UTR | 3'-uguccUAGAGAGAGGAGGAGACa-5' miRNA | | | | M T |
| 44069 | 44091 | Offset 6mer | 5'-cttttccCTTTCCTTTCCTCTTt-3' UTR | 3'-uguccuaGAGAGAGGAGGAGAca-5' miRNA | | | | M |
| 44095 | 44118 | Imperfect match | 5'-ttctttTTTTTCTTTATCCTTTGg-3' UTR | 3'-uguccuAGAGAGAGG-AGGAGACa-5' miRNA | | | | M |
| 44157 | 44180 | Imperfect match | 5'-tcctGCTCTCTCATCTTTCTCTGt-3' UTR | 3'-ugucCUAGAGAG-AGGAGGAGACa-5' miRNA | | | | M |
| 47940 | 47962 | 7mer-m8 | 5'-tgAGAGAT-TGTCTGATCCTCTGt-3' UTR | 3'-ugUC-CUAGAGAGAGGAGGAGACa-5' miRNA | | | | M T |
| 50431 | 50454 | Offset 6mer | 5'-ctcccGTCCCTTTCCTTCCTCTcc-3' UTR | 3'-uguccUAGAGAGAGG-AGGAGAca-5' miRNA | | | | M |
| 52116 | 52135 | Imperfect match | 5'-aaAGG--CT-TTTCGTTCTCTGt-3' UTR | 3'-ugUCCUAGAGAGAGGAGGAGACa-5' miRNA | | | | M |
| 54965 | 54987 | Imperfect match | 5'-tgGGTGTGTTTTGTTTCTTCTGt-3' UTR | 3'-ugUCCUAGAGAGAGGAGGAGACa-5' miRNA | | | | M |
| 57596 | 57618 | Offset 6mer | 5'-tacGGATCTGGTTGGTCCTCTTc-3' UTR | 3'-uguCCUAGAGAGAGGAGGAGAca-5' miRNA | | | | M |
| 57621 | 57644 | Imperfect match | 5'-ccatctTCTCTGTCCTTCTTCTGt-3' UTR | 3'-uguccuAGAGAGAGG-AGGAGACa-5' miRNA | | | | M |
| 60987 | 61010 | Imperfect match | 5'-tctcttTCTTTTTCTTTCTTCTGt-3' UTR | 3'-uguccuAGAGAGAG-GAGGAGACa-5' miRNA | | | | M |
| 61658 | 61681 | Imperfect match | 5'-tcaaccTCTAATTTACTCTTCTGc-3' UTR | 3'-uguccuAGA-GAGAGGAGGAGACa-5' miRNA | | | | M |
| 61757 | 61779 | Offset 6mer | 5'-cacaacTCGTCCTCCTCCTCTat-3' UTR | 3'-uguccuAGAGAGAGGAGGAGAca-5' miRNA | | | | M |
| 62670 | 62693 | Offset 6mer | 5'-caAGGAGCTCTGCCCCTCCTCTtg-3' UTR | 3'-ugUCCUAGAGA-GAGGAGGAGAca-5' miRNA | | | | M |
| 64689 | 64712 | Imperfect match | 5'-tttGGATATGATGTTCTCTTCTGg-3' UTR | 3'-uguCCUAGA-GAGAGGAGGAGACa-5' miRNA | | | | M |
| 69805 | 69827 | Imperfect match | 5'-ttctttaaTTACTCTTTCTCTGt-3' UTR | 3'-uguccuagAGAGAGGAGGAGACa-5' miRNA | | | | M |
| 71951 | 71975 | 7mer-m8 | 5'-acatttTCTGTATCCATTCCTCTGt-3' UTR | 3'-uguccuAGAGAGAGG--AGGAGACa-5' miRNA | | | | M T |
| 72824 | 72846 | Imperfect match | 5'-aaATGCTGTCTTTCTTCCACTGg-3' UTR | 3'-ugUCCUAGAGAGAGGAGGAGACa-5' miRNA | | | | M |
| 75112 | 75134 | 7mer-m8 | 5'-aaaaaaaaaaaaaaTTCCTCTGt-3' UTR | 3'-uguccuagagagagGAGGAGACa-5' miRNA | | | | M T |
| 75386 | 75408 | 7mer-m8 | 5'-tgAGAGAATGTCTGATCCTCTGt-3' UTR | 3'-ugUCCUAGAGAGAGGAGGAGACa-5' miRNA | | | | M T |
| 76198 | 76220 | Imperfect match | 5'-ttAGAATTTTGCTTGTCTTCTGg-3' UTR | 3'-ugUCCUAGAGAGAGGAGGAGACa-5' miRNA | | | | M |

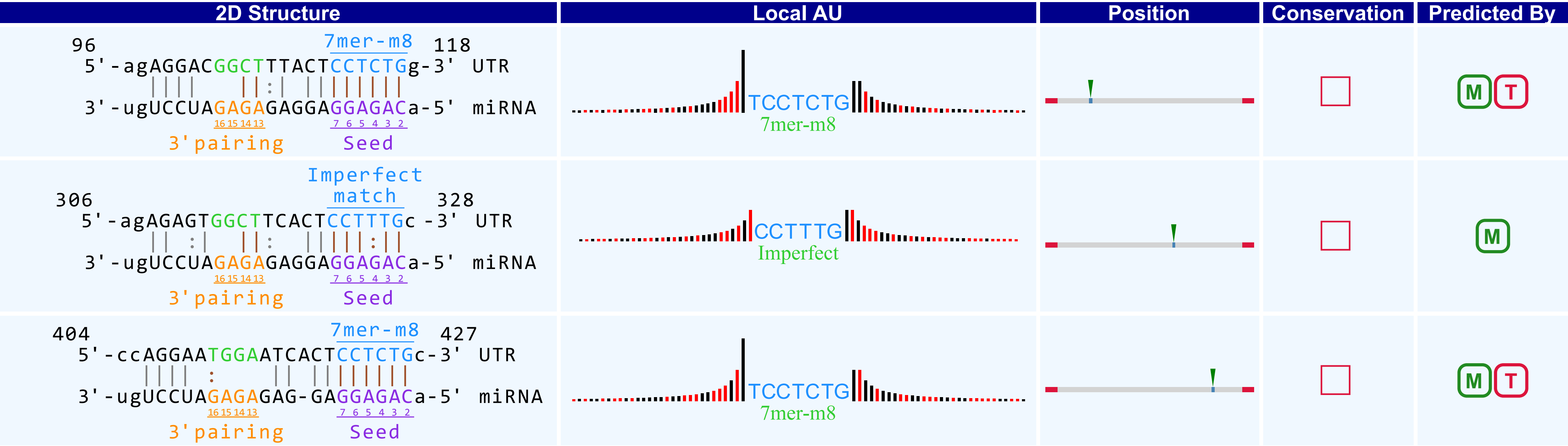
2D Structure
Local AU
Position
Conservation
Predicted By
96
7mer-m8
118
5'-agAGGACGGCTTTACTCCTCTGg-3' UTR
3'-ugUCCUAGAGAGAGGAGGAGACa-5' miRNA
16 15 14 13
7 6 5 4 3 2
3'pairing
Seed
TCCTCTG
7mer-m8
M
T
306
Imperfect match
328
5'-agAGAGTGGCTTCACTCCTTTGc -3' UTR
3'-ugUCCUAGAGAGAGGAGGAGACa-5' miRNA
16 15 14 13
7 6 5 4 3 2
3'pairing
Seed
CCTTTG
Imperfect
M
404
7mer-m8
427
5'-ccAGGAATGGAATCACTCCTCTGc-3' UTR
3'-ugUCCUAGAGAGAG-GAGGAGACa-5' miRNA
16 15 14 13
7 6 5 4 3 2
3'pairing
Seed
TCCTCTG
7mer-m8
M
T

| 2D Structure | Local AU | Position | Conservation | Predicted By |
|---|---|---|---|---|
| 90 7mer-m8 113<br>5'-cctcatccaGCACGTGCGCTCCAt-3' UTR<br>\|\| \|\|:\|\|\|\|\|\|\|\|<br>3'-cggacggagCGGGCGCGCGAGGUc-5' miRNA<br>16 15 14 13 7 6 5 4 3 2<br>3'pairing Seed | 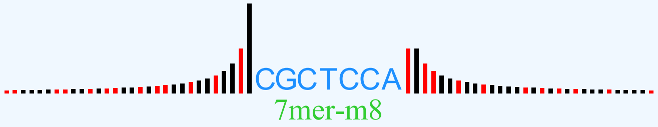 | 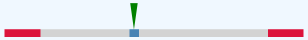 | 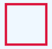 | 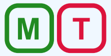 |

<table>
<tr><th>2D Structure</th><th>Local AU</th><th>Position</th><th>Conservation</th><th>Predicted By</th></tr>
<tr><td>Imperfect match<br>40 62<br>5'-atgGTCTTCAGAGAGCAACTAtc -3' UTR<br>||| |||||:|:||||:|<br>3'-agaCAG-AGUCUUUUGUUGGUuc-5' miRNA<br>16 15 14 13 7 6 5 4 3 2<br>3'pairing Seed</td><td>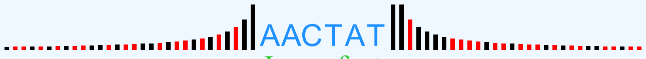<br>AACTAT<br>Imperfect</td><td>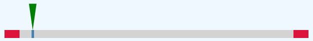</td><td></td><td>M</td></tr>
<tr><td>6mer<br>483 505<br>5'-aatccCTGCAGAAGAAAACCAAg-3' UTR<br>|| ||||||:| ||||||<br>3'-agacaGA-GUCUUUUGUUGGUUc-5' miRNA<br>16 15 14 13 7 6 5 4 3 2<br>3'pairing Seed</td><td>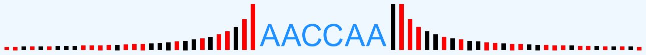<br>AACCAA<br>6mer</td><td>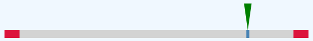</td><td></td><td>M</td></tr>
</table>

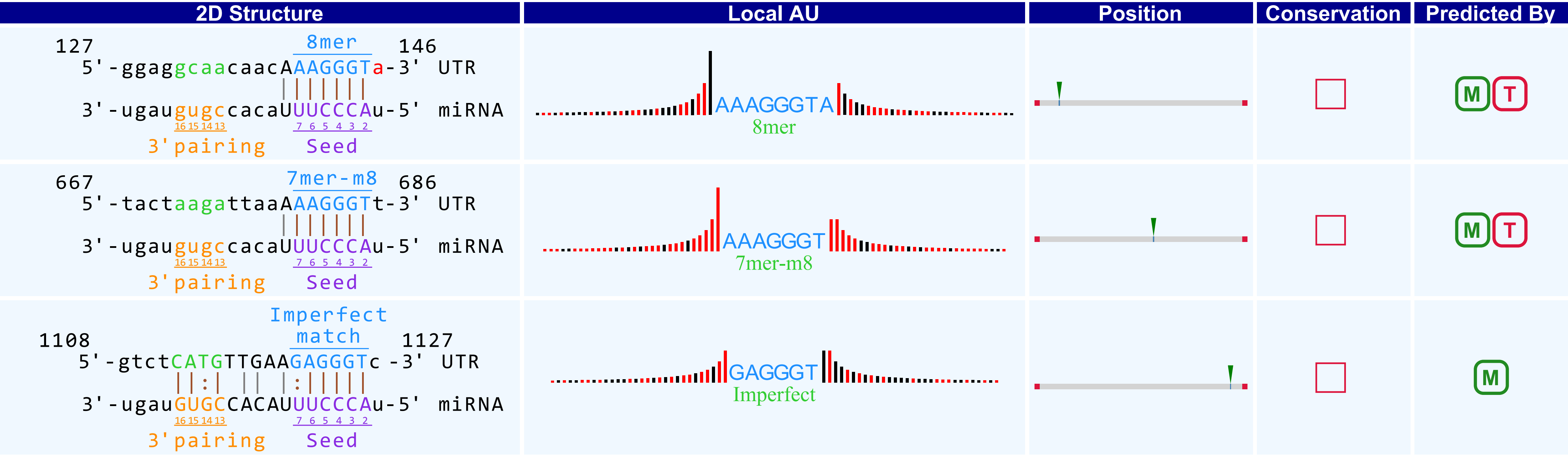

2D Structure
Local AU
Position
Conservation
Predicted By
127
8mer
146
5'-ggaggcaacaacAAAGGGTa-3' UTR
3'-ugaugugccacaUUCCCAu-5' miRNA
16 15 14 13
7 6 5 4 3 2
3'pairing
Seed
AAAGGGTA
8mer
M
T
667
7mer-m8
686
5'-tactaagattaaAAAGGGTt-3' UTR
3'-ugaugugccacaUUCCCAu-5' miRNA
16 15 14 13
7 6 5 4 3 2
3'pairing
Seed
AAAGGGT
7mer-m8
M
T
Imperfect match
1108
1127
5'-gtctCATGTTGAAGAGGGTc -3' UTR
3'-ugauGUGCCACAUUUCCCAu-5' miRNA
16 15 14 13
7 6 5 4 3 2
3'pairing
Seed
GAGGGT
Imperfect
M

| 2D Structure | Local AU | Position | Conservation | Predicted By |
|---|---|---|---|---|
| 34 7mer-m8 57<br>5'-gaAGTGATTGCTCAAGGACCTCCc-3' UTR<br>\|\| \| : \|\|:\| \|\|\|\|\|\|\|\|<br>3'-agUC-CCGCCGGGGUCCUGGAGGa-5' miRNA<br>16 15 14 13 7 6 5 4 3 2<br>3'pairing Seed | 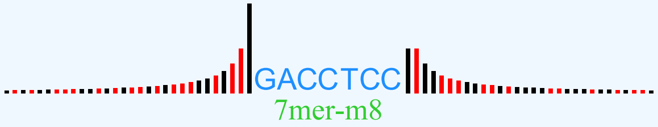 | 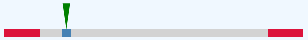 | 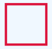 | M T |

| 2D Structure | Local AU | Position | Conservation | Predicted By |
|---|---|---|---|---|
| Imperfect match<br>284 … 309<br>5'-tttGGGGGGCTCTAGGGTGACTTCCc -3' UTR<br>\|\|\|  \|\|\|:\|:\|\|   \|\|\|:\|\|\|<br>3'-aguCCCGCCGGGGUC---CUGGAGGa-5' miRNA<br>16 15 14 13 … 7 6 5 4 3 2<br>3'pairing … Seed | 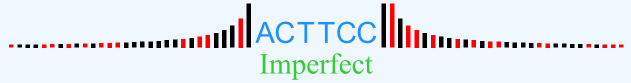 | 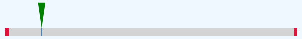 | ☐ | M |
| 7mer-m8<br>554 … 578<br>5'-agcctccacaTCCAGGTGACCTCCc-3' UTR<br>:\|\|\|\|  \|\|\|\|\|\|\|<br>3'-agucccgccgGGGUC--CUGGAGGa-5' miRNA<br>16 15 14 13 … 7 6 5 4 3 2<br>3'pairing … Seed | 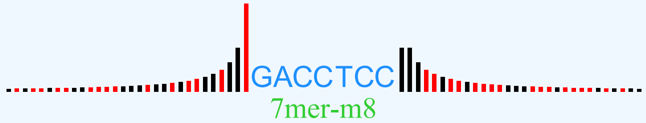 | 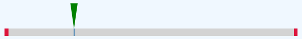 | ☐ | M T |
| Imperfect match<br>856 … 878<br>5'-aacacagGGCTCCAGGGCCTCat -3' UTR<br>\|\|\|:\|\|\|\|\|:\|\|\|\|<br>3'-agucccgCCGGGGUCCUGGAGga-5' miRNA<br>16 15 14 13 … 7 6 5 4 3 2<br>3'pairing … Seed | 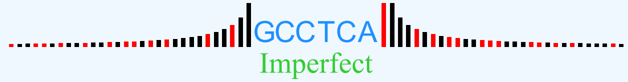 | 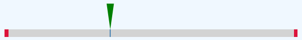 | ☐ | M |
| Imperfect match<br>2156 … 2181<br>5'-catGGGGGGCTCTAGGGTGACTTCCc -3' UTR<br>\|\|\|  \|\|\|:\|:\|\|   \|\|\|:\|\|\|<br>3'-aguCCCGCCGGGGUC---CUGGAGGa-5' miRNA<br>16 15 14 13 … 7 6 5 4 3 2<br>3'pairing … Seed | 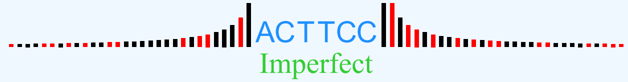 | 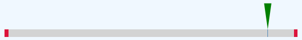 | ☐ | M |

<table>
<tr><th>2D Structure</th><th>Local AU</th><th>Position</th><th>Conservation</th><th>Predicted By</th></tr>
<tr><td>Offset 6mer<br>41 61<br>5'-gcTGGGAGC-AACAGCCAGAGa-3' UTR<br>||: |||  | ||||||:<br>3'-ccACUGUCGACUUUCGGUCUUg-5' miRNA<br>16 15 14 13 7 6 5 4 3 2<br>3'pairing Seed</td><td>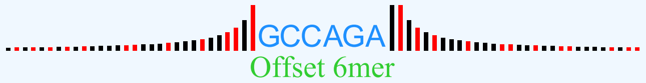</td><td>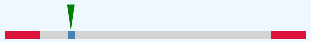</td><td></td><td>M</td></tr>
<tr><td>7mer-m8<br>68 90<br>5'-ctgGGAAGCACCATGGCCAGAAc-3' UTR<br>|: |||  | :|||||||<br>3'-ccaCUGUCG-ACUUUCGGUCUUg-5' miRNA<br>16 15 14 13 7 6 5 4 3 2<br>3'pairing Seed</td><td>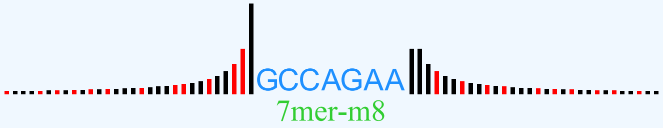</td><td>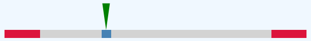</td><td></td><td>M T</td></tr>
</table>

| 2D Structure | Local AU | Position | Conservation | Predicted By |
|---|---|---|---|---|
| Offset 6mer<br>732 755<br>5'-tgCCTACTAGTTCCTCAGCTGCTg-3' UTR<br>3'-acGUAAGACUGAACGGUCGACGGu-5' miRNA<br>16 15 14 13 7 6 5 4 3 2<br>3'pairing Seed | 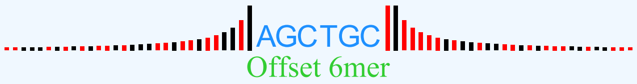 | 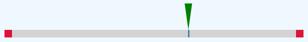 | | M |
| 7mer-m8<br>899 922<br>5'-ttttcTATGAAACATCAGCTGCCc-3' UTR<br>3'-acguaAGACUGAACGGUCGACGGu-5' miRNA<br>16 15 14 13 7 6 5 4 3 2<br>3'pairing Seed | 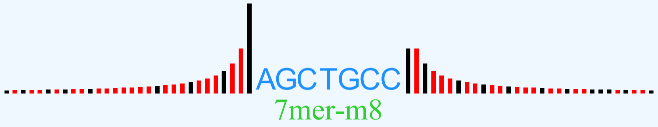 | 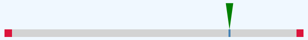 | | M T |
| Offset 6mer<br>1047 1070<br>5'-gcttTTCTCAGTGTACAGCTGCag-3' UTR<br>3'-acguAAGACUGAACGGUCGACGgu-5' miRNA<br>16 15 14 13 7 6 5 4 3 2<br>3'pairing Seed | 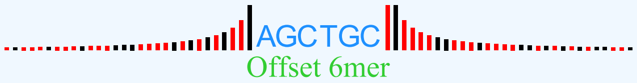 | 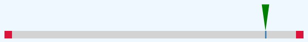 | | M |

| 2D Structure | Local AU | Position | Conservation | Predicted By |
|---|---|---|---|---|
| 200 7mer-m8 222<br>5'-gggaagatAGCTCAAATCCTTAt-3' UTR<br>|| :| |||||||<br>3'-cuuaagguUCUGGGUUAGGAAUu-5' miRNA<br>16 15 14 13 7 6 5 4 3 2<br>3'pairing Seed | 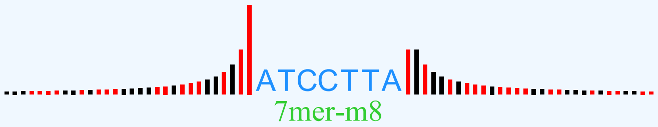  | 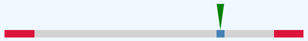 | | M T |
| Offset 6mer<br>219 242<br>5'-ttATGTCCAGTTTGAAATCCTTGg-3' UTR<br>|| ||||: : ||||||:<br>3'-cuUA-AGGUUCUGGGUUAGGAAUu-5' miRNA<br>16 15 14 13 7 6 5 4 3 2<br>3'pairing Seed | 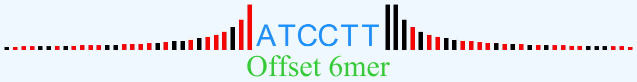  | 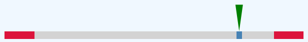 | | M |

| 2D Structure | Local AU | Position | Conservation | Predicted By |
|---|---|---|---|---|
| 457 7mer-m8 479<br>5'-tttgtGGTGACGGTGCTGCTGAt-3' UTR<br>:\|:\|\| \| \|\|\|\|\|\|\|\|\|<br>3'-cgguuUCGCUUCGACGACGACUg-5' miRNA<br>16 15 14 13 7 6 5 4 3 2<br>3'pairing Seed | 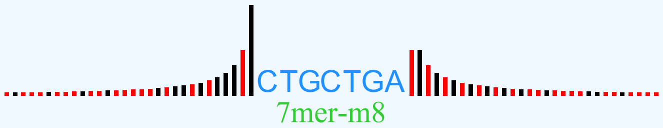 | 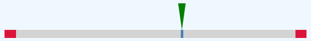 | | M T |
| 736 7mer-m8 758<br>5'-ggccttGCATGGTTTCTGCTGAc-3' UTR<br>\|\| :\|:\| \|\|\|\|\|\|\|<br>3'-cgguuuCGCUUCGACGACGACUg-5' miRNA<br>16 15 14 13 7 6 5 4 3 2<br>3'pairing Seed | 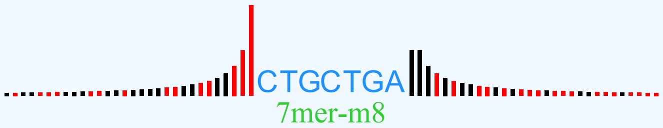 | 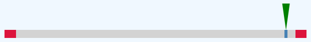 | | M T |

| 2D Structure | Local AU | Position | Conservation | Predicted By |
|---|---|---|---|---|

```
203                  7mer-m8  225
   5'-acaAAACTTACTCTGCTGCTGAt-3'  UTR
         |||  :  |  ||||||||||
   3'-cggUUUCGCUUCGACGACGACUg-5'  miRNA
             16 15 14 13  7 6 5 4 3 2
          3'pairing        Seed
```

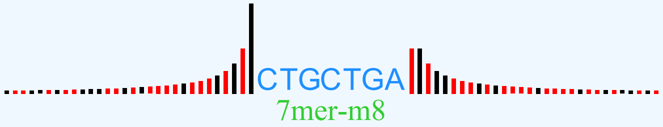


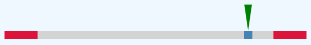

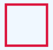

M T

| 2D Structure | Local AU | Position | Conservation | Predicted By |
|---|---|---|---|---|
| Offset 6mer<br>132 155<br>5'-caGAGGCAGAAACAGGAGGAAAtg-3' UTR<br>3'-uuUUCC--CUUUGUCAUCCUUUUg-5' miRNA<br>16 15 14 13 · 7 6 5 4 3 2<br>3'pairing Seed | 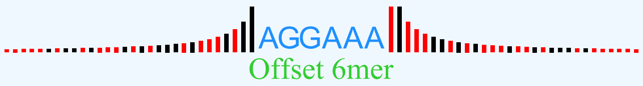 AGGAAA Offset 6mer | 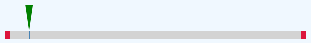 | | M |
| Imperfect match<br>594 615<br>5'-ctGATGGAGAGACAAGGAAGAt -3' UTR<br>3'-uuUUCCCUUUGUCAUCCUUUUg-5' miRNA<br>16 15 14 13 · 7 6 5 4 3 2<br>3'pairing Seed | 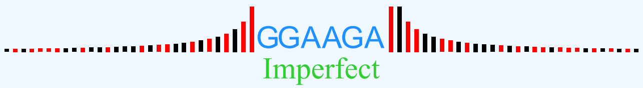 GGAAGA Imperfect | 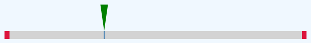 | | M |
| Offset 6mer<br>1424 1447<br>5'-ccAGGGGAGACCCAGAAGGAAAtc-3' UTR<br>3'-uuUUCCCUUU--GUCAUCCUUUUg-5' miRNA<br>16 15 14 13 · 7 6 5 4 3 2<br>3'pairing Seed | 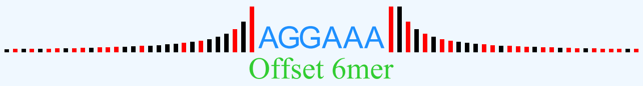 AGGAAA Offset 6mer | 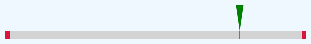 | | M |

<table>
<tr><th>2D Structure</th><th>Local AU</th><th>Position</th><th>Conservation</th><th>Predicted By</th></tr>
<tr><td>Imperfect match<br>77 101<br>5'-gaGAGGGGAGAAGTTACAGGAAGAa -3' UTR<br>:||||:|:  |||   |||||:|<br>3'-uuUUCCCUUUGUCA---UCCUUUUg-5' miRNA<br>16 15 14 13 7 6 5 4 3 2<br>3'pairing Seed</td><td>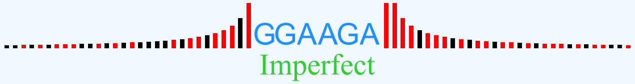</td><td>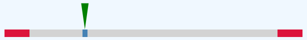</td><td></td><td>M</td></tr>
<tr><td>Offset 6mer<br>147 166<br>5'-gaAATGGGGA-AG-AGGAAAGc-3' UTR<br>||  ||::|  ||  ||||||:<br>3'-uuUUCCCUUUGUCAUCCUUUUg-5' miRNA<br>16 15 14 13 7 6 5 4 3 2<br>3'pairing Seed</td><td>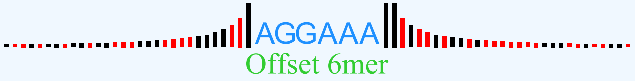</td><td>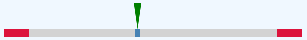</td><td></td><td>M</td></tr>
</table>

| 2D Structure | Local AU | Position | Conservation | Predicted By |
|---|---|---|---|---|

```
159                        7mer-m8  185
   5'-ccATCCTCCAGCTTTGCTGCCGCACTg-3'  UTR
        || |||||||    | ||||||||
   3'-cgUA-GAGGUCG----GUCGGCGUGAc-5'  miRNA
            16 15 14 13        7 6 5 4 3 2
          3'pairing          Seed
```

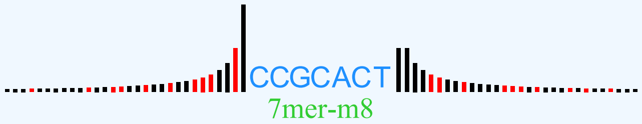


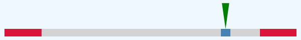

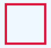

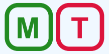

| 2D Structure | Local AU | Position | Conservation | Predicted By |
|---|---|---|---|---|
| 173 7mer-m8 196<br>5'-atgttGACACCTACCATGCTGCTg-3' UTR<br>3'-uuaucCUAUUUAU-GCACGACGAu-5' miRNA<br>16 15 14 13 7 6 5 4 3 2<br>3'pairing Seed | 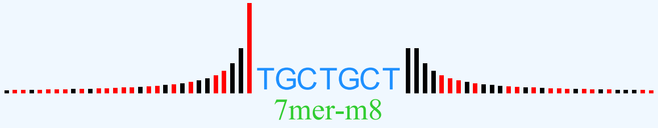 | 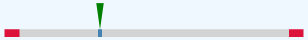 | | M T |
| 225 7mer-m8 247<br>5'-tttttctgACATATTTGCTGCTc-3' UTR<br>3'-uuauccuaUUUAUGCACGACGAu-5' miRNA<br>16 15 14 13 7 6 5 4 3 2<br>3'pairing Seed | 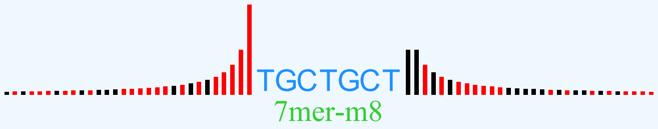 | 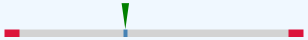 | | M T |

| 2D Structure | Local AU | Position | Conservation | Predicted By |
| --- | --- | --- | --- | --- |
| 299 7mer-m8 322<br>5'-atgttGACACCTACCATGCTGCTg-3' UTR<br>3'-uuaucCUAUUUAU-GCACGACGAu-5' miRNA<br>16 15 14 13 7 6 5 4 3 2<br>3'pairing Seed | 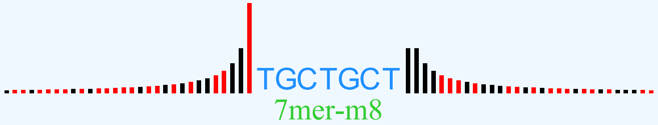 | 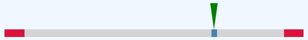 | | M T |
| 351 7mer-m8 373<br>5'-tttttctgACATATTTGCTGCTc-3' UTR<br>3'-uuauccuaUUUAUGCACGACGAu-5' miRNA<br>16 15 14 13 7 6 5 4 3 2<br>3'pairing Seed | 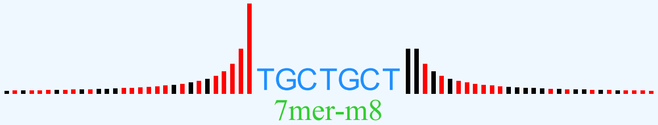 | 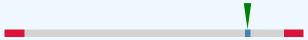 | | M T |

| 2D Structure | Local AU | Position | Conservation | Predicted By |
|---|---|---|---|---|
| 101 7mer-m8 124<br>5'-gtACAGTATGTGATTCAAGGCTAt-3' UTR<br>|||: ||:| ||| |||||||<br>3'-guUGUU-UAUA-UAAUUUCCGAUu-5' miRNA<br>16 15 14 13 7 6 5 4 3 2<br>3'pairing Seed | 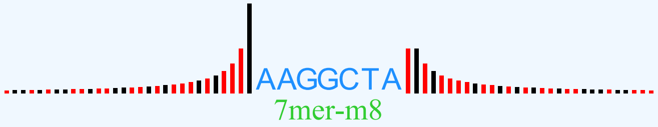 | 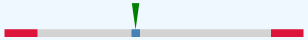 | | M T |

| 2D Structure | Local AU | Position | Conservation | Predicted By |
|---|---|---|---|---|

```
34                      8mer     58
 5'-gggccCTGTGGAGTCACTCCAGGGa-3'  UTR
        ||||::|   |||||||||
 3'-agaucGACAUUU---UGAGGUCCCu-5'  miRNA
         16 15 14 13  7 6 5 4 3 2
       3'pairing         Seed
```

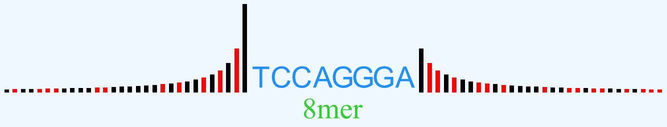


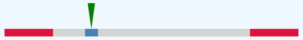

M T

<table>
<tr><th>2D Structure</th><th>Local AU</th><th>Position</th><th>Conservation</th><th>Predicted By</th></tr>
<tr><td>Imperfect match<br>280 301<br>5'-actctTTGGGGGGCTCTAGGGt-3' UTR<br>:||  ::::|||:||||<br>3'-agaucGACAUUUUGAGGUCCCu-5' miRNA<br>16 15 14 13 7 6 5 4 3 2<br>3'pairing Seed</td><td>CTAGGG<br>Imperfect</td><td></td><td></td><td>M</td></tr>
<tr><td>Imperfect match<br>347 368<br>5'-aggaaaTGTGGACATCTAGGGt-3' UTR<br>|||::|  ||:||||<br>3'-agaucgACAUUUUGAGGUCCCu-5' miRNA<br>16 15 14 13 7 6 5 4 3 2<br>3'pairing Seed</td><td>CTAGGG<br>Imperfect</td><td></td><td></td><td>M</td></tr>
<tr><td>7mer-m8<br>852 873<br>5'-ggacaacacAGGGCTCCAGGGc-3' UTR<br>|:::||||||||<br>3'-agaucgacaUUUUGAGGUCCCu-5' miRNA<br>16 15 14 13 7 6 5 4 3 2<br>3'pairing Seed</td><td>TCCAGGG<br>7mer-m8</td><td></td><td></td><td>M T</td></tr>
<tr><td>Imperfect match<br>2152 2173<br>5'-actccaTGGGGGGCTCTAGGGt-3' UTR<br>||  ::::|||:||||<br>3'-agaucgACAUUUUGAGGUCCCu-5' miRNA<br>16 15 14 13 7 6 5 4 3 2<br>3'pairing Seed</td><td>CTAGGG<br>Imperfect</td><td></td><td></td><td>M</td></tr>
<tr><td>Imperfect match<br>2219 2240<br>5'-aggaaaTGTGGACATCTAGGGt-3' UTR<br>|||::|  ||:||||<br>3'-agaucgACAUUUUGAGGUCCCu-5' miRNA<br>16 15 14 13 7 6 5 4 3 2<br>3'pairing Seed</td><td>CTAGGG<br>Imperfect</td><td></td><td></td><td>M</td></tr>
</table>

<table>
<tr><th>2D Structure</th><th>Local AU</th><th>Position</th><th>Conservation</th><th>Predicted By</th></tr>
<tr><td>62 8mer 82<br>5'-atcaaGTTGGACAGCACTTTa-3' UTR<br>|| : ||||||||||<br>3'-acaggCACUGUGUCGUGAAAu-5' miRNA<br>16 15 14 13 7 6 5 4 3 2<br>3'pairing Seed</td><td>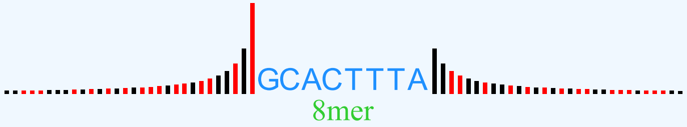</td><td>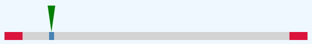</td><td></td><td>M T</td></tr>
<tr><td>Imperfect match<br>377 397<br>5'-gaaaCCTGACCGAGTACTTTg-3' UTR<br>| |||| ||:|||||<br>3'-acagGCACUGUGUCGUGAAAu-5' miRNA<br>16 15 14 13 7 6 5 4 3 2<br>3'pairing Seed</td><td>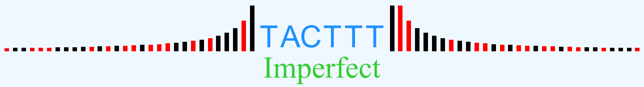</td><td>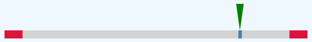</td><td></td><td>M</td></tr>
</table>

| 2D Structure | Local AU | Position | Conservation | Predicted By |
|---|---|---|---|---|
| 47 8mer 66<br>5'-tcaCTC-CAGGGACATCTGCa-3' UTR<br>\|\|\| \| \| \| \|\|\|\|\|\|\|<br>3'-ccgGAGUGACACGGUAGACGu-5' miRNA<br>16 15 14 13 7 6 5 4 3 2<br>3'pairing Seed | 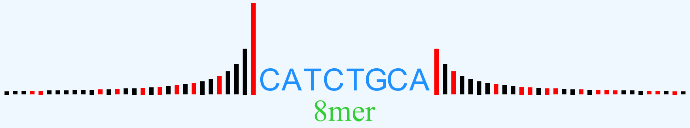 | 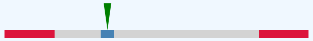 | | M T |
| Offset 6mer<br>100 119<br>5'-gggTTTACTG-GTCATCTGga-3' UTR<br>:\|:\|\|\|\| \|:\|\|\|\|\|\|<br>3'-ccgGAGUGACACGGUAGACgu-5' miRNA<br>16 15 14 13 7 6 5 4 3 2<br>3'pairing Seed | 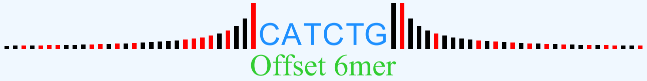 | 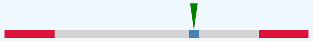 | | M |

| 2D Structure | Local AU | Position | Conservation | Predicted By |
| --- | --- | --- | --- | --- |
| 77 7mer-m8 98<br>5'-ctTCAGAA-CCTATCCATTGCAt-3' UTR<br>\|\|\|\|\|\|   \|:\|\|  \|\|\|\|\|\|\|<br>3'-ggAGUCUUAUGGUA-GUAACGUu-5' miRNA<br>16 15 14 13 7 6 5 4 3 2<br>3'pairing Seed | 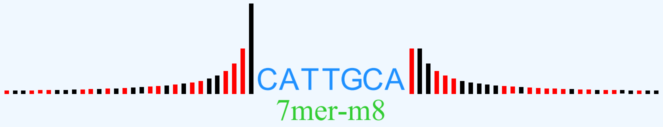 | 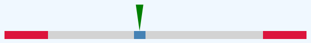 | □ | M T |

<table>
<tr><th>2D Structure</th><th>Local AU</th><th>Position</th><th>Conservation</th><th>Predicted By</th></tr>
<tr><td>134 7mer-m8 155<br>5'-ctcTAGATTATTCTCATTGCAg-3' UTR<br>:||| ||:: ||||||||<br>3'-ggaGUCUUAUGGUAGUAACGUu-5' miRNA<br>16 15 14 13 7 6 5 4 3 2<br>3'pairing Seed</td><td>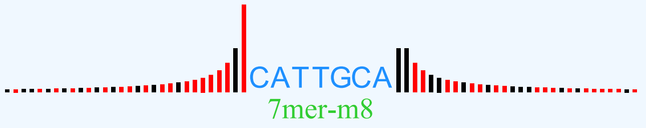</td><td>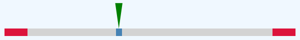</td><td></td><td>M T</td></tr>
<tr><td>300 8mer 325<br>5'-aacaACATTACCAGCCTCCATTGCAa-3' UTR<br>| | ||||| | |||||||<br>3'-ggagUCUUAUGGU---A-GUAACGUu-5' miRNA<br>16 15 14 13 7 6 5 4 3 2<br>3'pairing Seed</td><td>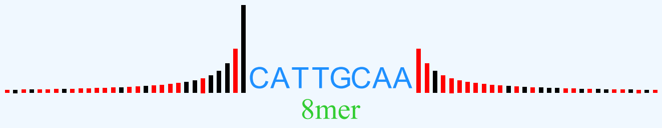</td><td>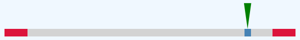</td><td></td><td>M T</td></tr>
</table>

<table>
<tr><th>2D Structure</th><th>Local AU</th><th>Position</th><th>Conservation</th><th>Predicted By</th></tr>
<tr><td>Imperfect match<br>292 314<br>5'-tgcGGGTCTTGGAGAAAATGCTt-3' UTR<br>|| ||:||| :||| ||||<br>3'-cgcCCAAGGACCCUUUUGACGAg-5' miRNA<br>16 15 14 13 7 6 5 4 3 2<br>3'pairing Seed</td><td>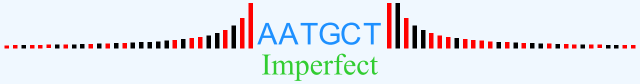</td><td>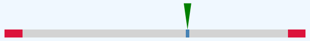</td><td></td><td>M</td></tr>
<tr><td>Imperfect match<br>393 416<br>5'-atGGGGTCCAGGAACCAGCTGCTg-3' UTR<br>||| ||| || | |:||||<br>3'-cgCCCAAGGACCCU-UUUGACGAg-5' miRNA<br>16 15 14 13 7 6 5 4 3 2<br>3'pairing Seed</td><td>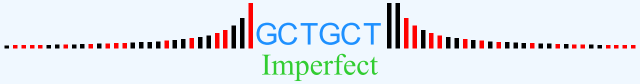</td><td>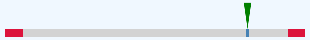</td><td></td><td>M</td></tr>
</table>

<table>
<tr><th>2D Structure</th><th>Local AU</th><th>Position</th><th>Conservation</th><th>Predicted By</th></tr>
<tr><td>383 7mer-m8 405<br>5'-caggACTTCA-GAGAGCCCATGTc-3' UTR<br>| :: || | :|||||||||<br>3'-cuggUAGGGUACGUUCGGGUACAc-5' miRNA<br>16 15 14 13 7 6 5 4 3 2<br>3'pairing Seed</td><td>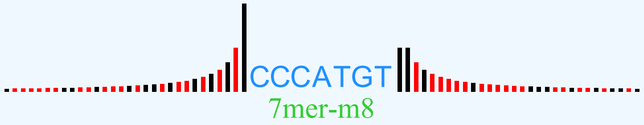</td><td>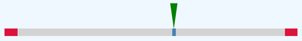</td><td></td><td>M T</td></tr>
<tr><td>Imperfect match<br>635 660<br>5'-acaCACTCCCTCGCAGAGCCCGTGTc -3' UTR<br>|| |||| ||| |||||:|||<br>3'-cugGU-AGGGUACGU-UCGGGUACAc-5' miRNA<br>16 15 14 13 7 6 5 4 3 2<br>3'pairing Seed</td><td>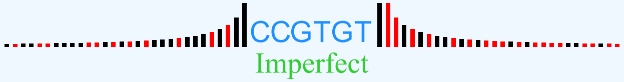</td><td>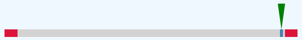</td><td></td><td>M</td></tr>
</table>

| 2D Structure | Local AU | Position | Conservation | Predicted By |
|---|---|---|---|---|
| 141 8mer 163<br>5'-tgttGGATGTGTTCACACCCGCa-3' UTR<br>\| \|::::\|:\| \|\|\|\|\|\|\|<br>3'-uccuCGUGUGUAGGAGUGGGCGu-5' miRNA<br>16 15 14 13 7 6 5 4 3 2<br>3'pairing Seed | 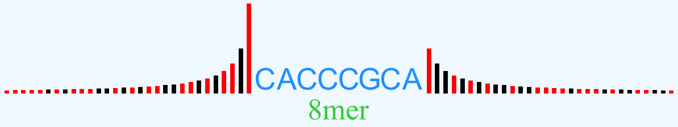 | 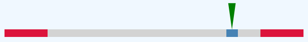 | | M T |

| 2D Structure | Local AU | Position | Conservation | Predicted By |
|---|---|---|---|---|
| 42 8mer 62<br>5'-gaCA-GGCGGATACAAAAGCTa-3' UTR<br>\|\| \|\| \| : \|\|\|\|\|\|\|<br>3'-acGUACCCCGGGAGUUUUCGAg-5' miRNA<br>16 15 14 13 7 6 5 4 3 2<br>3'pairing Seed | 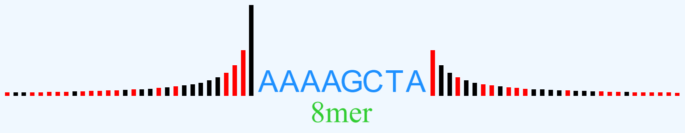 | 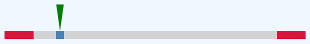 | | M T |
| 233 7mer-m8 254<br>5'-ggctgcaaaggTTCAAAAGCTg-3' UTR<br>:\|\|\|\|\|\|\|\|<br>3'-acguaccccggGAGUUUUCGAg-5' miRNA<br>16 15 14 13 7 6 5 4 3 2<br>3'pairing Seed | 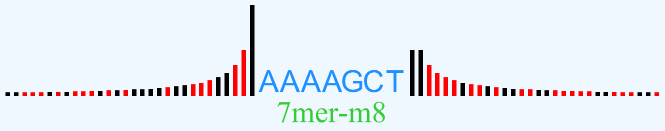 | 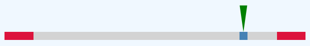 | | M T |

| 2D Structure | Local AU | Position | Conservation | Predicted By |
|---|---|---|---|---|
| Imperfect match<br>606 626<br>5'-gcATTTTCTATATTTCCCTGc -3' UTR<br>3'-cgUAGAGAAGGGAAGGGGACu-5' miRNA<br>16 15 14 13 7 6 5 4 3 2<br>3'pairing Seed | TCCCTG<br>Imperfect | | | M |
| Offset 6mer<br>3760 3777<br>5'-caATCTCTTC---TCCCCTcc-3' UTR<br>3'-cgUAGAGAAGGGAAGGGGACu-5' miRNA<br>16 15 14 13 7 6 5 4 3 2<br>3'pairing Seed | TCCCCT<br>Offset 6mer | | | M |
| Imperfect match<br>4276 4296<br>5'-tttTGTGTTTTGTTCCTCTGc -3' UTR<br>3'-cguAGAGAAGGGAAGGGGACu-5' miRNA<br>16 15 14 13 7 6 5 4 3 2<br>3'pairing Seed | CCTCTG<br>Imperfect | | | M |
| Imperfect match<br>8590 8609<br>5'-tttcCTATTCTC-TCTCCTGc -3' UTR<br>3'-cguaGAGAAGGGAAGGGGACu-5' miRNA<br>16 15 14 13 7 6 5 4 3 2<br>3'pairing Seed | CTCCTG<br>Imperfect | | | M |
| Imperfect match<br>8608 8629<br>5'-gctcaaTTTCCTGTTCTCCTGt -3' UTR<br>3'-cguagaGAAGGG-AAGGGGACu-5' miRNA<br>16 15 14 13 7 6 5 4 3 2<br>3'pairing Seed | CTCCTG<br>Imperfect | | | M |
| Offset 6mer<br>8629 8653<br>5'-tttgCTCTTCTGTAGCTTCCCCTtt-3' UTR<br>3'-cguaGAGAAGG----GAAGGGGAcu-5' miRNA<br>16 15 14 13 7 6 5 4 3 2<br>3'pairing Seed | TCCCCT<br>Offset 6mer | | | M |
| Imperfect match<br>8704 8725<br>5'-ctGTCTCCTCCCTTCCTCCTGa-3' UTR<br>3'-cgUAGAGAAGGGAAGG-GGACu-5' miRNA<br>16 15 14 13 7 6 5 4 3 2<br>3'pairing Seed | CCTCCTG<br>Imperfect | | | M |

| 2D Structure | Local AU | Position | Conservation | Predicted By |
|---|---|---|---|---|
| 296 7mer-m8 322<br>5'-atTATGTTGACACCTACCATGCTGCTg-3' UTR<br>:\|\|\| \|\| \|\| \| \|\|\|\|\|\|\|<br>3'-agGUAC-ACAGUCUA----ACGACGAc-5' miRNA<br>16 15 14 13 7 6 5 4 3 2<br>3'pairing Seed | 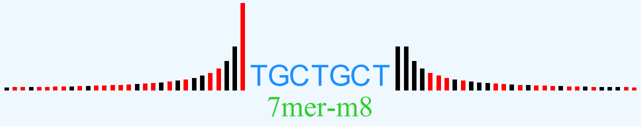  | 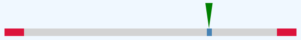 | ☐ | M T |
| 351 7mer-m8 373<br>5'-ttttTCTGACATATTTGCTGCTc-3' UTR<br>\| \|\| \|\| \| \|\|\|\|\|\|\|\|<br>3'-agguACACAGUCU-AACGACGAc-5' miRNA<br>16 15 14 13 7 6 5 4 3 2<br>3'pairing Seed | 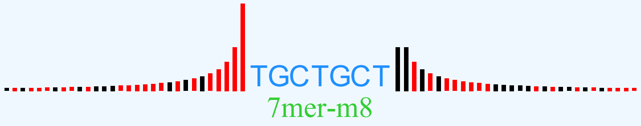  | 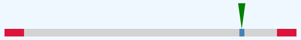 | ☐ | M T |

| 2D Structure | Local AU | Position | Conservation | Predicted By |
| --- | --- | --- | --- | --- |

```
111                     7mer-m8  132
  5'-tcTTTAAGTCCATATTTCAGTg-3'  UTR
       |:||||  ||| |||||||
  3'-guAGAUUC--GUA-AAAGUCAc-5'  miRNA
       16 15 14 13        7 6 5 4 3 2
       3'pairing          Seed
```

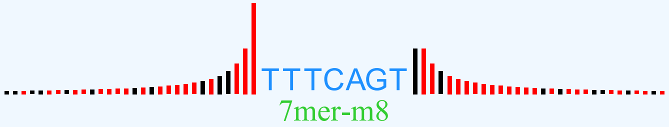


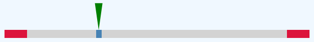

M T

| 2D Structure | Local AU | Position | Conservation | Predicted By |
| --- | --- | --- | --- | --- |
| `111            7mer-m8  132`<br>`5'-tcTTTAAGTCCATATTTCAGTg-3'  UTR`<br>`     \|:\|\|\|\|  \|\|\| \|\|\|\|\|\|\|`<br>`3'-guAGAUUC--GUA-AAAGUCAc-5'  miRNA`<br>`16 15 14 13    7 6 5 4 3 2`<br>`3'pairing     Seed` | 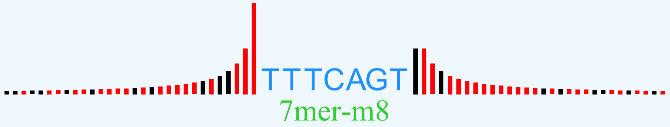 | 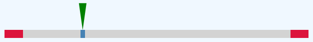 | 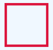 | M T |

| 2D Structure | Local AU | Position | Conservation | Predicted By |
|---|---|---|---|---|
| 169 8mer 192<br>5'-gaGTAGATGTTATAGGAAGGACAa-3' UTR<br>\|:\|\|\| \| :\|:\| \|\|\|\|\|\|\|\|<br>3'-gaCGUCUUCUGUGU-CUUCCUGUu-5' miRNA<br>16 15 14 13 7 6 5 4 3 2<br>3'pairing Seed | 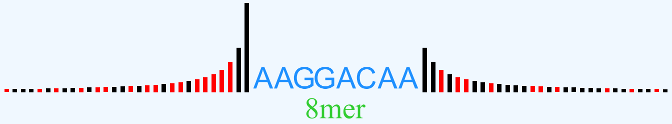 | 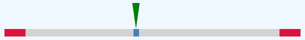 | | M T |
| Imperfect match<br>234 255<br>5'-gaaCAG-GGGCACCGAGGGACAc -3' UTR<br>\|\|\| :\|:\|\|\| \|\|:\|\|\|\|\|<br>3'-gacGUCUUCUGUGUCUUCCUGUu-5' miRNA<br>16 15 14 13 7 6 5 4 3 2<br>3'pairing Seed | 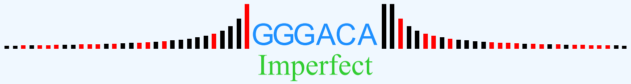 | 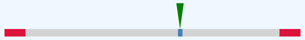 | | M |

| 2D Structure | Local AU | Position | Conservation | Predicted By |
|---|---|---|---|---|
| Imperfect match<br>472 494<br>5'-atGCAGATGACAGCA-AGGGACAa -3' UTR<br>3'-gaCGUCUUCUGU-GUCUUCCUGUu-5' miRNA<br>16 15 14 13 7 6 5 4 3 2<br>3'pairing Seed | 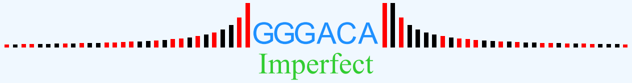 | 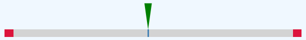 | | M |
| Offset 6mer<br>773 798<br>5'-gcGCAGACGTTAACACACAAGGACtg-3' UTR<br>3'-gaCGUCUUC---UGUGUCUUCCUGuu-5' miRNA<br>16 15 14 13 7 6 5 4 3 2<br>3'pairing Seed | 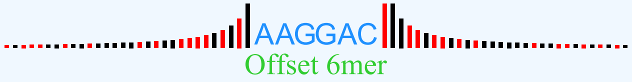 | 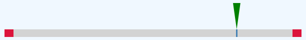 | | M |

| 2D Structure | Local AU | Position | Conservation | Predicted By |
|---|---|---|---|---|
| 7mer-m8<br>83 103<br>5'-agccccctcGGTCCATCACCc-3' UTR<br>:\|:\|\|\|\|\|\|\|\|<br>3'-gauguucucUCGGGUAGUGGu-5' miRNA<br>16 15 14 13 7 6 5 4 3 2<br>3'pairing Seed | 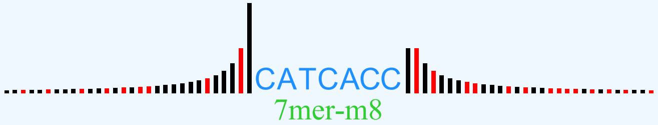 | 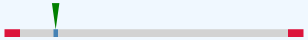 | ☐ | M T |
| 7mer-m8<br>368 391<br>5'-actgGGGAGAACCTTCCATCACCt-3' UTR<br>::\|\|\|\| \| \|\|\|\|\|\|\|\|<br>3'-gaugUUCUCUCG---GGUAGUGGu-5' miRNA<br>16 15 14 13 7 6 5 4 3 2<br>3'pairing Seed | 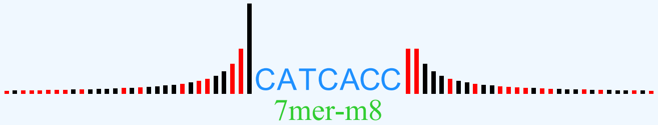 | 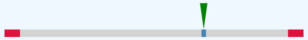 | ☐ | M T |

<table>
<tr><th>2D Structure</th><th>Local AU</th><th>Position</th><th>Conservation</th><th>Predicted By</th></tr>
<tr><td>86 8mer 105<br>5'-cgacTCTATGGCTTCCTGCa-3' UTR<br>:|| || |||||||<br>3'-uccaGGAGACGUAAGGACGu-5' miRNA<br>16 15 14 13 7 6 5 4 3 2<br>3'pairing Seed</td><td>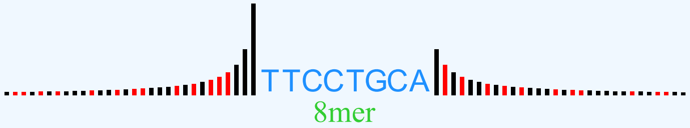
</td><td>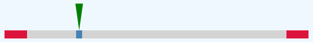</td><td></td><td>M T</td></tr>
<tr><td>184 7mer-m8 204<br>5'-ccagCTTGTGTCCTTCCTGCg-3' UTR<br>|:| || | ||||||<br>3'-uccaGGAGAC-GUAAGGACGu-5' miRNA<br>16 15 14 13 7 6 5 4 3 2<br>3'pairing Seed</td><td>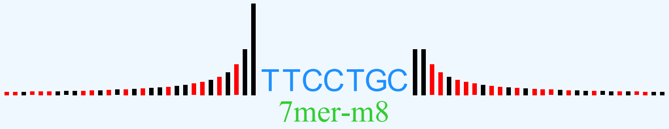
</td><td>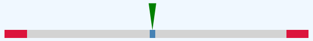</td><td></td><td>M T</td></tr>
</table>

| 2D Structure | Local AU | Position | Conservation | Predicted By |
|---|---|---|---|---|
| Offset 6mer<br>178 200<br>5'-agattcGGGACAATGGTGAGGGc-3' UTR<br>\| \|\|\|\|\|\| \|\|\|\|\|\|:<br>3'-gaaguaCACUGUUA-CACUCCUu-5' miRNA<br>16 15 14 13 7 6 5 4 3 2<br>3'pairing Seed | 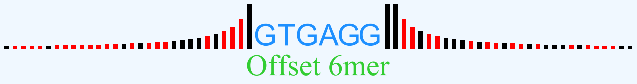<br> | 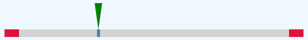 | | M |
| 7mer-m8<br>560 583<br>5'-tgTCCTGAAACCTGATGTGAGGAt-3' UTR<br>\|\| \|\| \|\| :\|\|\|\|\|\|\|\|\|<br>3'-gaAGUACACUG--UUACACUCCUu-5' miRNA<br>16 15 14 13 7 6 5 4 3 2<br>3'pairing Seed | 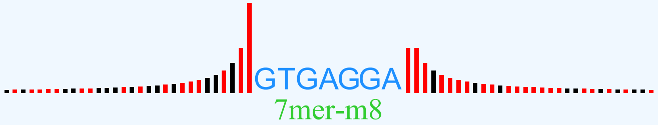<br> | 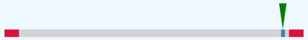 | | M T |

| 2D Structure | Local AU | Position | Conservation | Predicted By |
|---|---|---|---|---|
| (see below) | 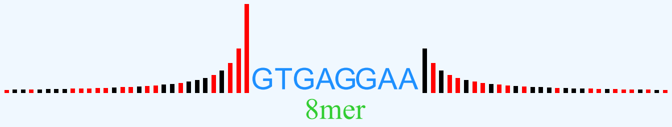 | 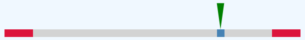 | | M T |

```
211                   8mer       234
  5'-tacttgGTGGCAGATAGTGAGGAa-3'  UTR
         |||:|| || |||||||
  3'-gaaguaCACUGU-UA-CACUCCUu-5'  miRNA
         16 15 14 13    7 6 5 4 3 2
        3'pairing        Seed
```

| 2D Structure | Local AU | Position | Conservation | Predicted By |
|---|---|---|---|---|
| 168 7mer-m8 188<br>5'-caaCCA-TGTAAGGGAGAGTGt-3' UTR<br>\|\|\| :::\|\|\|\|\|\|\|\|\|\|\|<br>3'-gacGGUCGUGUUCCCUCUCACa-5' miRNA<br>16 15 14 13 7 6 5 4 3 2<br>3'pairing Seed | 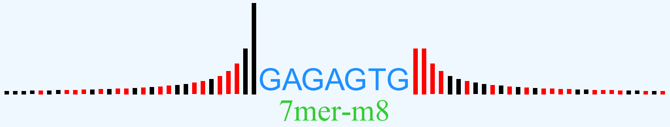 | 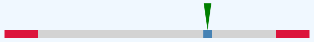 | | M T |

| 2D Structure | Local AU | Position | Conservation | Predicted By |
|---|---|---|---|---|
| Imperfect match<br>61 5'-ccGCCACCGTCAACCCCCTCACCCCc-3' 86 UTR<br>3'-uaCGGUGUC---UGGGUGAGUCGGGu-5' miRNA<br>16 15 14 13 7 6 5 4 3 2<br>3'pairing Seed | CACCCC<br>Imperfect | | | M |
| Imperfect match<br>127 5'-ccaCCAGCAGTACGCTGTCGGCCCa-3' 151 UTR<br>3'-uacGGU-GUC-UGGGUGAGUCGGGu-5' miRNA<br>16 15 14 13 7 6 5 4 3 2<br>3'pairing Seed | CGGCCC<br>Imperfect | | | M |

<table>
<tr><th>2D Structure</th><th>Local AU</th><th>Position</th><th>Conservation</th><th>Predicted By</th></tr>
<tr><td>Imperfect match<br>315 341<br>5'-ccacTGCCCAACCCCACTTCTCCAGGt-3' UTR<br>|| ||| ||||: |:|||||<br>3'-agacACCGGU---GGUGGUGGGGUCCu-5' miRNA<br>16 15 14 13 7 6 5 4 3 2<br>3'pairing Seed</td><td>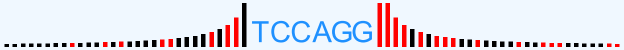<br>TCCAGG<br>Imperfect</td><td>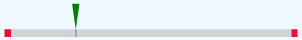</td><td></td><td>M</td></tr>
<tr><td>Imperfect match<br>477 500<br>5'-aaTGTGCGTGCAGGTGCTCCAGGt-3' UTR<br>|||| ::| : ::|:|||||<br>3'-agACACCGGUGGUGGUGGGGUCCu-5' miRNA<br>16 15 14 13 7 6 5 4 3 2<br>3'pairing Seed</td><td>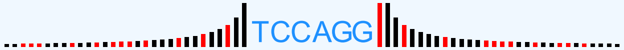<br>TCCAGG<br>Imperfect</td><td>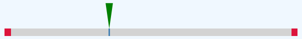</td><td></td><td>M</td></tr>
<tr><td>Offset 6mer<br>810 835<br>5'-accacGGCCAGCCTGCCACCCCAGAt-3' UTR<br>||||| |::|||||||||<br>3'-agacaCCGGU--GGUGGUGGGGUCCu-5' miRNA<br>16 15 14 13 7 6 5 4 3 2<br>3'pairing Seed</td><td>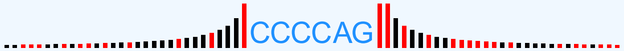<br>CCCCAG<br>Offset 6mer</td><td>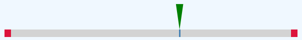</td><td></td><td>M</td></tr>
<tr><td>Imperfect match<br>1002 1025<br>5'-tccagaaCCACCATGCCTCCAGGc-3' UTR<br>||||||: |:|||||<br>3'-agacaccGGUGGUGGUGGGGUCCu-5' miRNA<br>16 15 14 13 7 6 5 4 3 2<br>3'pairing Seed</td><td>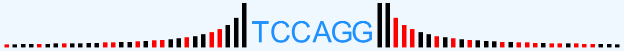<br>TCCAGG<br>Imperfect</td><td>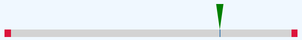</td><td></td><td>M</td></tr>
</table>

<table>
<tr><th>2D Structure</th><th>Local AU</th><th>Position</th><th>Conservation</th><th>Predicted By</th></tr>
<tr><td>165 8mer 189<br>5'-gtGAACACTTGTTTGAATCTGACCa-3' UTR<br>||||   :::|:||   |||||||<br>3'-ugCUUGGAGGUAGAC-GAGACUGGa-5' miRNA<br>16 15 14 13   7 6 5 4 3 2<br>3'pairing   Seed</td><td>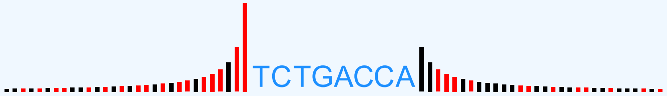<br>TCTGACCA<br>8mer</td><td>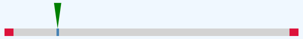</td><td></td><td>M T</td></tr>
<tr><td>Offset<br>210 6mer 234<br>5'-agGACCCTCTACAGGCTTCTGACaa-3' UTR<br>||  ||||:|    ||  ||||||<br>3'-ugCUUGGAGGUAGACG-AGACUGga-5' miRNA<br>16 15 14 13   7 6 5 4 3 2<br>3'pairing   Seed</td><td>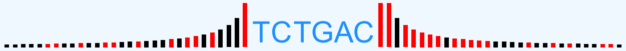<br>TCTGAC<br>Offset 6mer</td><td>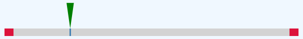</td><td></td><td>M</td></tr>
</table>

<table>
<tr><th>2D Structure</th><th>Local AU</th><th>Position</th><th>Conservation</th><th>Predicted By</th></tr>
<tr><td>Offset 6mer<br>370 391<br>5'-gaaaccGAATGGAGAGACCAcc-3' UTR<br>||| :| |||||||<br>3'-gacaucCUUCUCCCUCUGGUUc-5' miRNA<br>16 15 14 13 7 6 5 4 3 2<br>3'pairing Seed</td><td>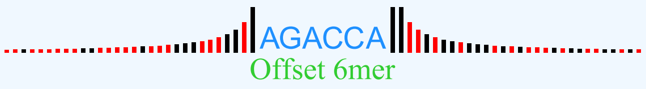</td><td></td><td></td><td>M</td></tr>
<tr><td>Imperfect match<br>597 619<br>5'-atGGAGAGACAAGGAAGATCAAg -3' UTR<br>| || || ||| |||:|||<br>3'-gaCAUC-CUUCUCCCUCUGGUUc-5' miRNA<br>16 15 14 13 7 6 5 4 3 2<br>3'pairing Seed</td><td>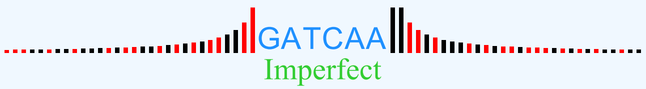</td><td></td><td></td><td>M</td></tr>
<tr><td>Offset 6mer<br>852 873<br>5'-aacatGAAGGAGACAGACCAGc-3' UTR<br>| |:||| |||||:<br>3'-gacauCCUUCUCCCUCUGGUUc-5' miRNA<br>16 15 14 13 7 6 5 4 3 2<br>3'pairing Seed</td><td>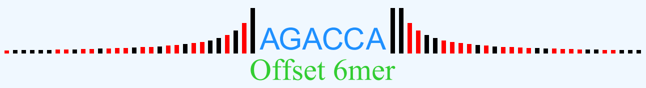</td><td></td><td></td><td>M</td></tr>
<tr><td>6mer<br>953 974<br>5'-gaagttGATGAGAGGGACCAAg-3' UTR<br>|| ||| |:||||||<br>3'-gacaucCUUCUCCCUCUGGUUc-5' miRNA<br>16 15 14 13 7 6 5 4 3 2<br>3'pairing Seed</td><td>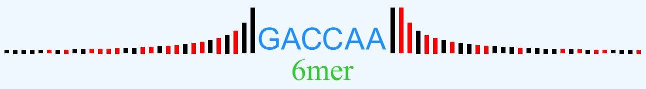</td><td></td><td></td><td>M</td></tr>
</table>

| 2D Structure | Local AU | Position | Conservation | Predicted By |
|---|---|---|---|---|
| 233 7mer-m8 256<br>5'-ctgcggcacattgGTACCTCAGCc-3' UTR<br>3'-ucuuggauuagggCA-GGAGUCGu-5' miRNA<br>16 15 14 13 7 6 5 4 3 2<br>3'pairing Seed | 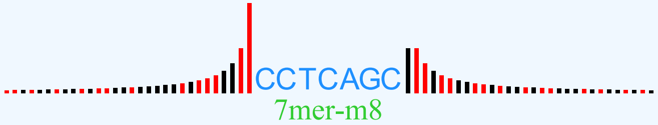 | | | M T |
| 284 7mer-m8 306<br>5'-acttgtgtggagCGGCCTCAGCt-3' UTR<br>3'-ucuuggauuaggGCAGGAGUCGu-5' miRNA<br>16 15 14 13 7 6 5 4 3 2<br>3'pairing Seed | 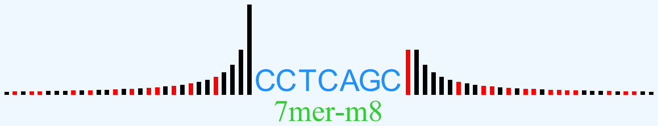 | | | M T |

| 2D Structure | Local AU | Position | Conservation | Predicted By |
|---|---|---|---|---|
| 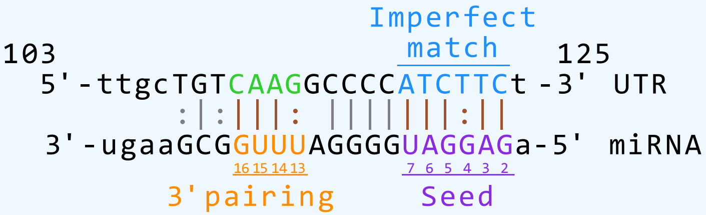  | 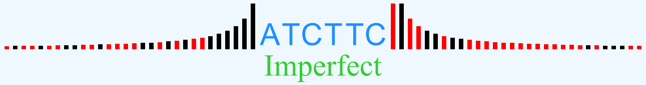  | 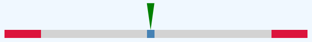 | | M |

<table>
<tr><th>2D Structure</th><th>Local AU</th><th>Position</th><th>Conservation</th><th>Predicted By</th></tr>
<tr><td>Imperfect match<br>145 168<br>5'-aacTCAACCATGATTTCTTTCTCa -3' UTR<br>:||| | | |:||||:|||<br>3'-ucaGGUUCGGA--AGAGAAGGAGu-5' miRNA<br>16 15 14 13 7 6 5 4 3 2<br>3'pairing Seed</td><td>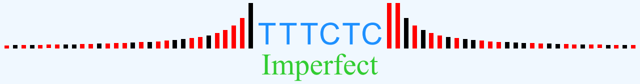<br></td><td>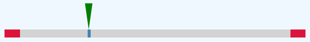</td><td>☐</td><td>M</td></tr>
<tr><td>Offset 6mer<br>169 191<br>5'-acTCAACAGCATTCCCTTCCTTg-3' UTR<br>|| | ||| ||| |||||:<br>3'-ucAGGU-UCGGAAGAGAAGGAGu-5' miRNA<br>16 15 14 13 7 6 5 4 3 2<br>3'pairing Seed</td><td>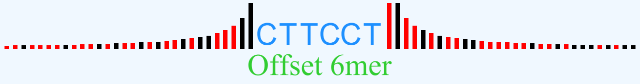<br></td><td>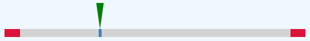</td><td>☐</td><td>M</td></tr>
</table>

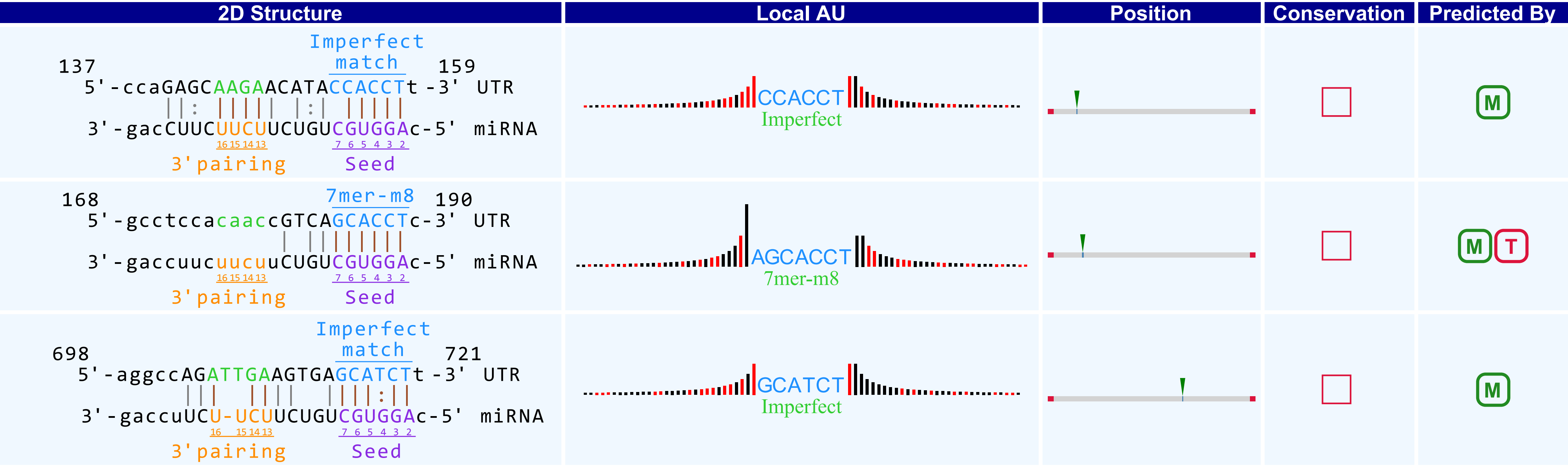
2D Structure
Local AU
Position
Conservation
Predicted By
Imperfect match
137
159
5'-ccaGAGCAAGAACATACCACCTt-3' UTR
3'-gacCUUCUUCUUCUGUCGUGGAc-5' miRNA
16 15 14 13
7 6 5 4 3 2
3'pairing
Seed
CCACCT
Imperfect
M
168
7mer-m8
190
5'-gcctccacaaccGTCAGCACCTc-3' UTR
3'-gaccuucuucuuCUGUCGUGGAc-5' miRNA
16 15 14 13
7 6 5 4 3 2
3'pairing
Seed
AGCACCT
7mer-m8
M
T
Imperfect match
698
721
5'-aggccAGATTGAAGTGAGCATCTt-3' UTR
3'-gaccuUCU-UCUUCUGUCGUGGAc-5' miRNA
16 15 14 13
7 6 5 4 3 2
3'pairing
Seed
GCATCT
Imperfect
M

| 2D Structure | Local AU | Position | Conservation | Predicted By |
|---|---|---|---|---|
| Imperfect match<br>79 99<br>5'-attgAGATACATTTTCTCCCa -3' UTR<br>\|\|\|\| \|  :\|:\|\|\|\|\|<br>3'-agugUCUACGACGAGGAGGGa-5' miRNA<br>16 15 14 13 7 6 5 4 3 2<br>3'pairing Seed | 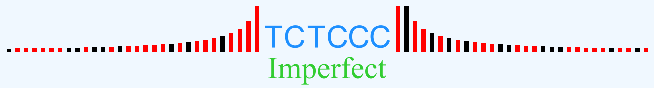 | 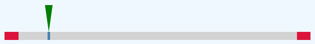 | | M |
| 7mer-m8<br>276 296<br>5'-tgATGGCTGATCCTCCTCCCc-3' UTR<br>\|::\| \|\| \| \|\|\|\|\|\|\|\|<br>3'-agUGUCUACGACGAGGAGGGa-5' miRNA<br>16 15 14 13 7 6 5 4 3 2<br>3'pairing Seed | 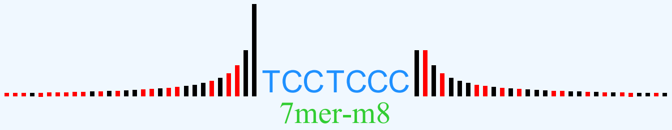 | 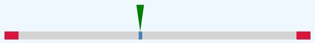 | | M T |

| 2D Structure | Local AU | Position | Conservation | Predicted By |
|---|---|---|---|---|
| 100 8mer 120<br>5'-gtggatcttCAAG-GCAGAGGa-3' UTR<br>|| | |||||||<br>3'-gaccaccccGUCCUCGUCUCCa-5' miRNA<br>16 15 14 13 7 6 5 4 3 2<br>3'pairing Seed | 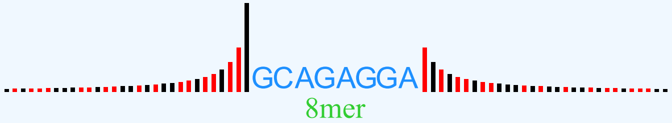 | 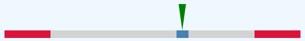 | ☐ | M T |
| 115 8mer 138<br>5'-agaggaaGTCATGTTGGCAGAGGa-3' UTR<br>| || | :|||||||<br>3'-gaccaccCCGUCC--UCGUCUCCa-5' miRNA<br>16 15 14 13 7 6 5 4 3 2<br>3'pairing Seed | 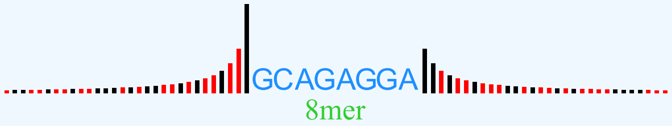 | 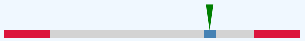 | ☐ | M T |

<table>
<tr><th>2D Structure</th><th>Local AU</th><th>Position</th><th>Conservation</th><th>Predicted By</th></tr>
<tr><td>232 6mer 257<br>5'-ggGAGGAGACACTTTCTCTCAGATGc-3' UTR<br>:||||||||  :|:||||||<br>3'-gaUUCCUCUGU----CGGGGUCUACu-5' miRNA<br>16 15 14 13 7 6 5 4 3 2<br>3'pairing Seed</td><td>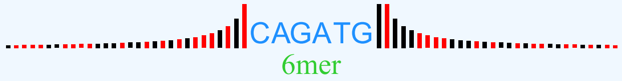</td><td>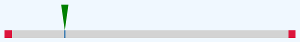</td><td></td><td>M</td></tr>
<tr><td>709 6mer 732<br>5'-aattGGACCTAGCCACTCAGATGt-3' UTR<br>|||  :|||  |:||||||<br>3'-gauuCCUCUGUCG--GGGUCUACu-5' miRNA<br>16 15 14 13 7 6 5 4 3 2<br>3'pairing Seed</td><td>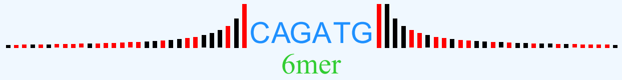</td><td>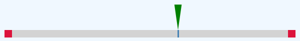</td><td></td><td>M</td></tr>
<tr><td>1138 7mer-A1 1161<br>5'-cgccaccGATAGCAGCTCAGATGa-3' UTR<br>||:|||  |:||||||<br>3'-gauuccuCUGUCG--GGGUCUACu-5' miRNA<br>16 15 14 13 7 6 5 4 3 2<br>3'pairing Seed</td><td>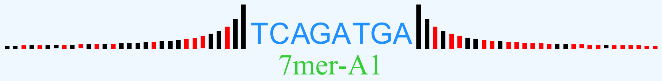</td><td>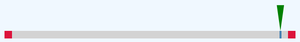</td><td></td><td>M T</td></tr>
</table>

| 2D Structure | Local AU | Position | Conservation | Predicted By |
|---|---|---|---|---|
| 83 7mer-m8 104<br>5'-agatacattttcTCCCAGATGt-3' UTR<br>3'-gauuccucugucGGGGUCUACu-5' miRNA<br>16 15 14 13 7 6 5 4 3 2<br>3'pairing Seed | 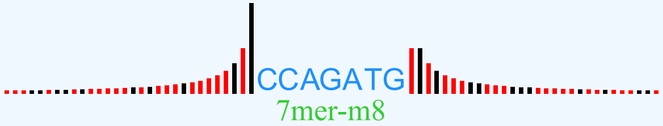 | 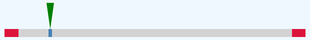 | | M T |
| 476 8mer 497<br>5'-cacAGCAACCAGCACCAGATGa-3' UTR<br>3'-gauUCCUCUGUCGGGGUCUACu-5' miRNA<br>16 15 14 13 7 6 5 4 3 2<br>3'pairing Seed | 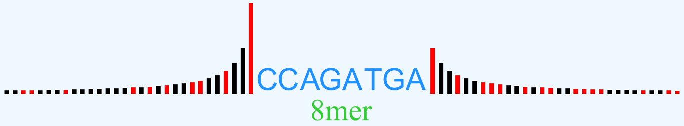 | 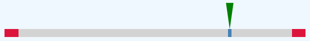 | | M T |

| 2D Structure | Local AU | Position | Conservation | Predicted By |
| --- | --- | --- | --- | --- |
| 102 8mer 124<br>5'-agtgaagaggagCAGCCAGCCCa-3' UTR<br>3'-gcuuuuuggaagGUCGGUCGGGa-5' miRNA<br>16 15 14 13 7 6 5 4 3 2<br>3'pairing Seed | CCAGCCCA<br>8mer | | | M T |
| Imperfect match<br>155 176<br>5'-tttcgctCCAT-CAGCCAGTCCa -3' UTR<br>3'-gcuuuuuGGAAGGUCGGUCGGGa-5' miRNA<br>16 15 14 13 7 6 5 4 3 2<br>3'pairing Seed | CAGTCC<br>Imperfect | | | M |
| Imperfect match<br>431 455<br>5'-tcAAAGCCCCACACAGCCTAGCCCt -3' UTR<br>3'-gcUUUUUGGAAG-GUC-GGUCGGGa-5' miRNA<br>16 15 14 13 7 6 5 4 3 2<br>3'pairing Seed | TAGCCC<br>Imperfect | | | M |
| 779 8mer 800<br>5'-acttcAACC-TCTGGCCAGCCCa-3' UTR<br>3'-gcuuuUUGGAAGGUCGGUCGGGa-5' miRNA<br>16 15 14 13 7 6 5 4 3 2<br>3'pairing Seed | CCAGCCCA<br>8mer | | | M T |
| 980 7mer-m8 1001<br>5'-cccaccACCACCCA-CCAGCCCt-3' UTR<br>3'-gcuuuuUGGAAGGUCGGUCGGGa-5' miRNA<br>16 15 14 13 7 6 5 4 3 2<br>3'pairing Seed | CCAGCCC<br>7mer-m8 | | | M T |

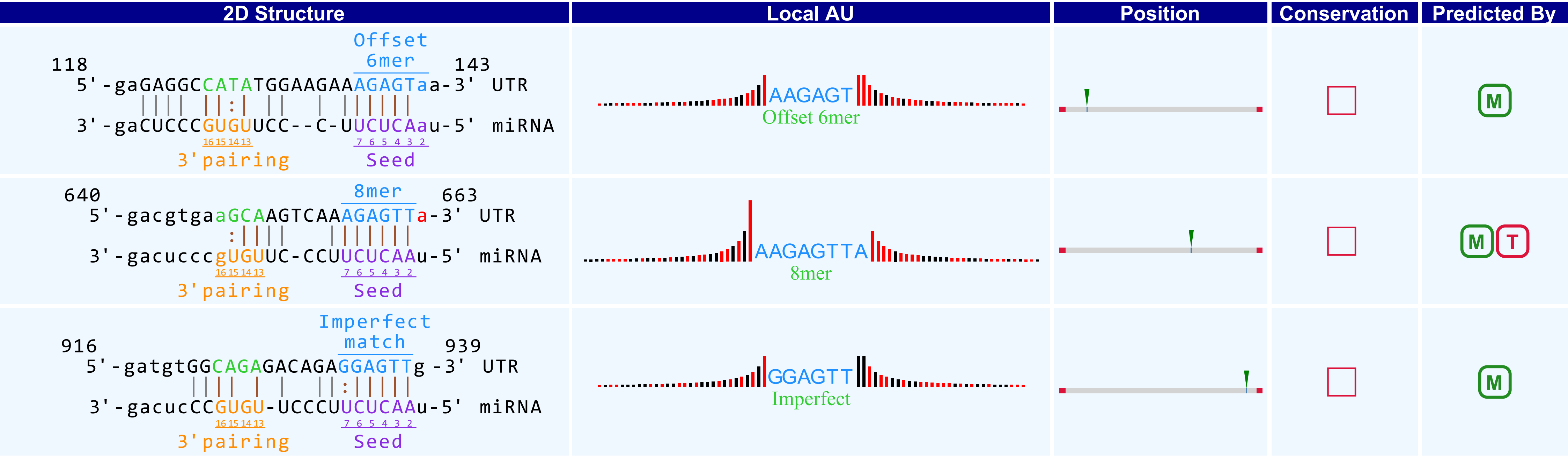
2D Structure
Local AU
Position
Conservation
Predicted By
Offset 6mer
118
143
5'-gaGAGGCCATATGGAAGAAAGAGTaa-3' UTR
3'-gaCUCCCGUGUUCC--C-UUCUCAau-5' miRNA
16 15 14 13
7 6 5 4 3 2
3'pairing
Seed
AAGAGT
Offset 6mer
M
8mer
640
663
5'-gacgtgaaGCAAGTCAAAGAGTTa-3' UTR
3'-gacucccgUGUUC-CCUUCUCAAu-5' miRNA
16 15 14 13
7 6 5 4 3 2
3'pairing
Seed
AAGAGTTA
8mer
M
T
Imperfect match
916
939
5'-gatgtGGCAGAGACAGAGGAGTTg-3' UTR
3'-gacucCCGUGU-UCCCUUCUCAAu-5' miRNA
16 15 14 13
7 6 5 4 3 2
3'pairing
Seed
GGAGTT
Imperfect
M

| 2D Structure | Local AU | Position | Conservation | Predicted By |
|---|---|---|---|---|
| 142 7mer-m8 161<br>5'-ggaaaGTCTG--TTTCCCAGGt-3' UTR<br>\|  \|\|\|   ::\|\|\|\|\|\|\|<br>3'-gguccCCGACGGGGAGGGUCCu-5' miRNA<br>16 15 14 13 7 6 5 4 3 2<br>3'pairing Seed | 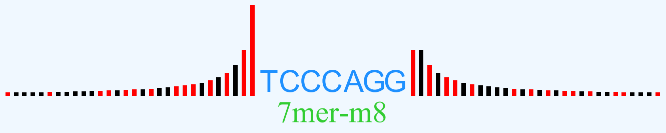 | 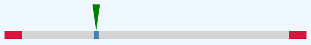 | | M T |
| 179 7mer-m8 201<br>5'-aactGGAACGCACCTTCCCAGGc-3' UTR<br>\|\|    \|\|  \|\|:\|\|\|\|\|\|\|<br>3'-ggucCCCGACG-GGGAGGGUCCu-5' miRNA<br>16 15 14 13 7 6 5 4 3 2<br>3'pairing Seed | 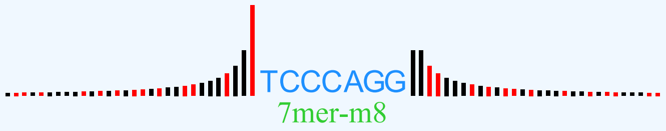 | 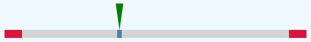 | | M T |

| 2D Structure | Local AU | Position | Conservation | Predicted By |
|---|---|---|---|---|
| Imperfect match<br>127 146<br>5'-gctGAAGTGTGGAGAGGAAg-3' UTR<br>: \| \| \| \| : \| \| \| \| : \| \| \|<br>3'-gacUUUCCCUUCUCUUCUUu-5' miRNA<br>16 15 14 13 7 6 5 4 3 2<br>3'pairing Seed | 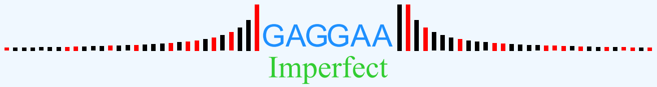 | 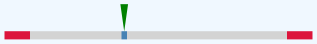 | | M |
| 7mer-m8<br>198 217<br>5'-ggggcctcGCAGAGAAGAAg-3' UTR<br>\| \| \| \| \| \| \| \| \| \| \|<br>3'-gacuuuccCUUCUCUUCUUu-5' miRNA<br>16 15 14 13 7 6 5 4 3 2<br>3'pairing Seed | 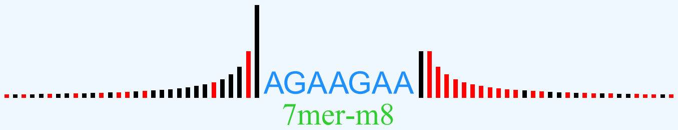 | 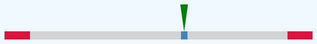 | | M T |

| 2D Structure | Local AU | Position | Conservation | Predicted By |
|---|---|---|---|---|
| 7mer-m8<br>1110 1130<br>5'-ttgaTTTTTTTTTCCCTTTt-3' UTR<br>\|\|:\|\|\|:\|:\|\|\|\|\|\|<br>3'-cguaAAGAAAGAGAGGGAAAa-5' miRNA<br>16 15 14 13 7 6 5 4 3 2<br>3'pairing Seed | TCCCTTT<br>7mer-m8 | | | M T |
| 7mer-m8<br>3960 3980<br>5'-ctgactgaTCTTTTCCCTTTc-3' UTR<br>\| :\|:\|\|\|\|\|\|<br>3'-cguaaagaAAGAGAGGGAAAa-5' miRNA<br>16 15 14 13 7 6 5 4 3 2<br>3'pairing Seed | TCCCTTT<br>7mer-m8 | | | M T |
| 7mer-m8<br>4036 4056<br>5'-taaaagaTTTAGTTCCCTTTt-3' UTR<br>\|\|\| :\|\|\|\|\|\|<br>3'-cguaaagAAAGAGAGGGAAAa-5' miRNA<br>16 15 14 13 7 6 5 4 3 2<br>3'pairing Seed | TCCCTTT<br>7mer-m8 | | | M T |
| Imperfect match<br>4285 4305<br>5'-ttGTTCCTCTGCCTCCTTTTc -3' UTR<br>:\|\| \|\| \| \|\|\|\|:\|\|\|<br>3'-cgUAAAGAAAGAGAGGGAAAa-5' miRNA<br>16 15 14 13 7 6 5 4 3 2<br>3'pairing Seed | CCTTTT<br>Imperfect | | | M |
| Imperfect match<br>5187 5209<br>5'-cagcTTTTTCATCTTCTCTTTt -3' UTR<br>\|\|:\|\|\|\| \|\| \|\|:\|\|\|\|<br>3'-cguaAAGAAAG-AG-AGGGAAAa-5' miRNA<br>16 15 14 13 7 6 5 4 3 2<br>3'pairing Seed | CTCTTT<br>Imperfect | | | M |
| Imperfect match<br>8573 8593<br>5'-ttGTTAAGTTCTTTCTCTTTc -3' UTR<br>:\|\| \|\|\|\|:\|\|:\|\|\|\|<br>3'-cgUAAAGAAAGAGAGGGAAAa-5' miRNA<br>16 15 14 13 7 6 5 4 3 2<br>3'pairing Seed | CTCTTT<br>Imperfect | | | M |
| Offset 6mer<br>8698 8719<br>5'-gctgTGCTGTCTCCTCCCTTcc-3' UTR<br>\| \|\| \|\|\| \|\|\|\|\|\|\|<br>3'-cguaAAGAAAGA-GAGGGAAAa-5' miRNA<br>16 15 14 13 7 6 5 4 3 2<br>3'pairing Seed | TCCCTT<br>Offset 6mer | | | M |

| 2D Structure | Local AU | Position | Conservation | Predicted By |
|---|---|---|---|---|
| Imperfect match<br>201 221<br>5'-actTTTCTGTCTTTTTCTTTg-3' UTR<br>3'-cguAAAGAAAGAGAGGGAAAa-5' miRNA<br>3'pairing Seed | TTCTTT<br>Imperfect | | | M |
| 6mer<br>8345 8365<br>5'-tgATTTTTTTAACCCCCTTTt-3' UTR<br>3'-cgUAAAGAAAGAGAGGGAAAa-5' miRNA<br>3'pairing Seed | CCCTTT<br>6mer | | | M |
| Imperfect match<br>9149 9169<br>5'-tcAATTTTATGTCTTCCTTTt-3' UTR<br>3'-cgUAAAGAAAGAGAGGGAAAa-5' miRNA<br>3'pairing Seed | TCCTTT<br>Imperfect | | | M |
| Imperfect match<br>10079 10099<br>5'-ccATCTCTTTTGATTCCTTTt-3' UTR<br>3'-cgUAAAGAAAGAGAGGGAAAa-5' miRNA<br>3'pairing Seed | TCCTTT<br>Imperfect | | | M |
| Imperfect match<br>10359 10379<br>5'-ttcTCTCTCTCTCTCTTTc-3' UTR<br>3'-cguAAAGAAAGAGAGGGAAAa-5' miRNA<br>3'pairing Seed | CTCTTT<br>Imperfect | | | M |
| Imperfect match<br>11010 11034<br>5'-agATATTCGTTCTTCCATCTCTTTt-3' UTR<br>3'-cgUA-AAGAAAGAG---AGGGAAAa-5' miRNA<br>3'pairing Seed | CTCTTT<br>Imperfect | | | M |
| Imperfect match<br>11274 11294<br>5'-accccTCTTTCTCTTTCTTTa-3' UTR<br>3'-cguaaAGAAAGAGAGGGAAAa-5' miRNA<br>3'pairing Seed | TTCTTT<br>Imperfect | | | M |
| Imperfect match<br>11380 11401<br>5'-atATTTATTTCTTTCCTCTTTa-3' UTR<br>3'-cgUAAAGAAAGAGAGG-GAAAa-5' miRNA<br>3'pairing Seed | CCTCTTT<br>Imperfect | | | M |
| Imperfect match<br>11425 11445<br>5'-attTTTCTTTCTCTCCTTTTt-3' UTR<br>3'-cguAAAGAAAGAGAGGGAAAa-5' miRNA<br>3'pairing Seed | CCTTTT<br>Imperfect | | | M |
| Imperfect match<br>11436 11456<br>5'-tctccTTTTTCTTTTTCTTTt-3' UTR<br>3'-cguaaAGAAAGAGAGGGAAAa-5' miRNA<br>3'pairing Seed | TTCTTT<br>Imperfect | | | M |
| Offset 6mer<br>13935 13954<br>5'-taAATTC-TTCTTTCCCTTac-3' UTR<br>3'-cgUAAAGAAAGAGAGGGAAaa-5' miRNA<br>3'pairing Seed | TCCCTT<br>Offset 6mer | | | M |
| Imperfect match<br>13969 13989<br>5'-attTTTATTTATTTCTCTTTc-3' UTR<br>3'-cguAAAGAAAGAGAGGGAAAa-5' miRNA<br>3'pairing Seed | CTCTTT<br>Imperfect | | | M |
| Imperfect match<br>15474 15495<br>5'-cccaTCCTTAACTTTCTCTTTg-3' UTR<br>3'-cguaAAGAA-AGAGAGGGAAAa-5' miRNA<br>3'pairing Seed | CTCTTT<br>Imperfect | | | M |
| Imperfect match<br>16219 16239<br>5'-caATTTTCCTATTTTCCTTTg-3' UTR<br>3'-cgUAAAGAAAGAGAGGGAAAa-5' miRNA<br>3'pairing Seed | TCCTTT<br>Imperfect | | | M |
| Imperfect match<br>16424 16441<br>5'-ggATTTCTTT---TTCCTTTc-3' UTR<br>3'-cgUAAAGAAAGAGAGGGAAAa-5' miRNA<br>3'pairing Seed | TCCTTT<br>Imperfect | | | M |
| Imperfect match<br>23496 23518<br>5'-gaATTACAGTTTATTTCCTTTc-3' UTR<br>3'-cgUAAAG--AAAGAGAGGGAAAa-5' miRNA<br>3'pairing Seed | TCCTTT<br>Imperfect | | | M |
| Imperfect match<br>26397 26419<br>5'-ctATCTCTTCCACAATTCCTTTt-3' UTR<br>3'-cgUAAAGAAAGAG--AGGGAAAa-5' miRNA<br>3'pairing Seed | TCCTTT<br>Imperfect | | | M |
| Imperfect match<br>29117 29139<br>5'-cctcTTCTTCCATTATCTCTTTc-3' UTR<br>3'-cguaAAGAAAG--AGAGGGAAAa-5' miRNA<br>3'pairing Seed | CTCTTT<br>Imperfect | | | M |
| Imperfect match<br>29199 29220<br>5'-cctcTTCTCTTTTCTCTCTTTt-3' UTR<br>3'-cguaAAGA-AAGAGAGGGAAAa-5' miRNA<br>3'pairing Seed | CTCTTT<br>Imperfect | | | M |
| Imperfect match<br>31480 31502<br>5'-gaATTACAGTTTATTTCCTTTc-3' UTR<br>3'-cgUAAAG--AAAGAGAGGGAAAa-5' miRNA<br>3'pairing Seed | TCCTTT<br>Imperfect | | | M |
| Imperfect match<br>32811 32831<br>5'-ttcTTTCTTTCTTTTCTTTTt-3' UTR<br>3'-cguAAAGAAAGAGAGGGAAAa-5' miRNA<br>3'pairing Seed | TCTTTT<br>Imperfect | | | M |
| Imperfect match<br>33836 33854<br>5'-cccaTTC-TTC-CTCCTTTTc-3' UTR<br>3'-cguaAAGAAAGAGAGGGAAAa-5' miRNA<br>3'pairing Seed | CCTTTT<br>Imperfect | | | M |
| Imperfect match<br>33861 33881<br>5'-tctTTTCCTTCTCTCTCTTCt-3' UTR<br>3'-cguAAAGAAAGAGAGGGAAaa-5' miRNA<br>3'pairing Seed | CTCTTC<br>Imperfect | | | M |
| Imperfect match<br>35861 35881<br>5'-atGTTAGTTCCTGTTCCTTTg-3' UTR<br>3'-cgUAAAGAAAGAGAGGGAAAa-5' miRNA<br>3'pairing Seed | TCCTTT<br>Imperfect | | | M |
| Imperfect match<br>38520 38540<br>5'-tgATGTCATTTTCTGCCTTTa-3' UTR<br>3'-cgUAAAGAAAGAGAGGGAAAa-5' miRNA<br>3'pairing Seed | GCCTTT<br>Imperfect | | | M |
| Imperfect match<br>39225 39244<br>5'-ctATTTC-CTCAGTCTCTTTt-3' UTR<br>3'-cgUAAAGAAAGAGAGGGAAAa-5' miRNA<br>3'pairing Seed | CTCTTT<br>Imperfect | | | M |
| Imperfect match<br>43870 43889<br>5'-tccTTTCTTTC-CTTCCTTTc-3' UTR<br>3'-cguAAAGAAAGAGAGGGAAAa-5' miRNA<br>3'pairing Seed | TCCTTT<br>Imperfect | | | M |
| Imperfect match<br>43883 43902<br>5'-tccTTTCTTTC-CTTCTTTTc-3' UTR<br>3'-cguAAAGAAAGAGAGGGAAAa-5' miRNA<br>3'pairing Seed | TCTTTT<br>Imperfect | | | M |
| Imperfect match<br>43896 43915<br>5'-tctTTTCTTTC-CTTCCTTTc-3' UTR<br>3'-cguAAAGAAAGAGAGGGAAAa-5' miRNA<br>3'pairing Seed | TCCTTT<br>Imperfect | | | M |
| Imperfect match<br>43918 43938<br>5'-tccTTCCTTTCTTTTCCTTTt-3' UTR<br>3'-cguAAAGAAAGAGAGGGAAAa-5' miRNA<br>3'pairing Seed | TCCTTT<br>Imperfect | | | M |
| Imperfect match<br>43951 43971<br>5'-tccTTCCTTTCTTTTCCTTTc-3' UTR<br>3'-cguAAAGAAAGAGAGGGAAAa-5' miRNA<br>3'pairing Seed | TCCTTT<br>Imperfect | | | M |
| Imperfect match<br>43971 43991<br>5'-cctTTCCTTTCTTTTCCTTTc-3' UTR<br>3'-cguAAAGAAAGAGAGGGAAAa-5' miRNA<br>3'pairing Seed | TCCTTT<br>Imperfect | | | M |
| Imperfect match<br>44005 44026<br>5'-tctTTCCTTTCCTTTCTCTTTt-3' UTR<br>3'-cguAAAGAAA-GAGAGGGAAAa-5' miRNA<br>3'pairing Seed | CTCTTT<br>Imperfect | | | M |
| Imperfect match<br>44021 44043<br>5'-tctTTTCTTTCCTTTTTCCTTTc-3' UTR<br>3'-cguAAAGAAAG--AGAGGGAAAa-5' miRNA<br>3'pairing Seed | TCCTTT<br>Imperfect | | | M |
| Imperfect match<br>44043 44063<br>5'-cctTTCCTTTCCTTTCCTTTc-3' UTR<br>3'-cguAAAGAAAGAGAGGGAAAa-5' miRNA<br>3'pairing Seed | TCCTTT<br>Imperfect | | | M |
| 7mer-m8<br>44058 44080<br>5'-cctTTCCTTTCCTTTTCCCTTTc-3' UTR<br>3'-cguAAAGAAAG--AGAGGGAAAa-5' miRNA<br>3'pairing Seed | TCCCTTT<br>7mer-m8 | | | M T |
| Imperfect match<br>44075 44097<br>5'-cctTTCCTTTCCTCTTTCCTTTc-3' UTR<br>3'-cguAAAGAAA-GAG-AGGGAAAa-5' miRNA<br>3'pairing Seed | TCCTTT<br>Imperfect | | | M |
| Imperfect match<br>44091 44110<br>5'-tccTTTCTTT-TTTTTCTTTa-3' UTR<br>3'-cguAAAGAAAGAGAGGGAAAa-5' miRNA<br>3'pairing Seed | TTCTTT<br>Imperfect | | | M |
| Imperfect match<br>44114 44134<br>5'-tttggctcaTCTTTTCCTTTt-3' UTR<br>3'-cguaaagaaAGAGAGGGAAAa-5' miRNA<br>3'pairing Seed | TCCTTT<br>Imperfect | | | M |
| Offset 6mer<br>46389 46409<br>5'-agtTTACATTCCTTCCCTTga-3' UTR<br>3'-cguAAAGAAAGAGAGGGAAaa-5' miRNA<br>3'pairing Seed | TCCCTT<br>Offset 6mer | | | M |
| Imperfect match<br>46818 46840<br>5'-acATTTCCTATATATTTCCTTTc-3' UTR<br>3'-cgUAAA-GAAAGA-GAGGGAAAa-5' miRNA<br>3'pairing Seed | TCCTTT<br>Imperfect | | | M |
| Offset 6mer<br>60046 60067<br>5'-attcTACTGTCTCCTCCCTTct-3' UTR<br>3'-cguaAAGAAAGA-GAGGGAAaa-5' miRNA<br>3'pairing Seed | TCCCTT<br>Offset 6mer | | | M |
| Imperfect match<br>60984 61004<br>5'-ggATCTCTTTCTTTTTCTTTc-3' UTR<br>3'-cgUAAAGAAAGAGAGGGAAAa-5' miRNA<br>3'pairing Seed | TTCTTT<br>Imperfect | | | M |
| Imperfect match<br>61787 61807<br>5'-cctTTTCCTTCTCTCTCTTCa-3' UTR<br>3'-cguAAAGAAAGAGAGGGAAaa-5' miRNA<br>3'pairing Seed | CTCTTC<br>Imperfect | | | M |
| Imperfect match<br>67498 67517<br>5'-ctATTTC-CTCAGTCTCTTTt-3' UTR<br>3'-cgUAAAGAAAGAGAGGGAAAa-5' miRNA<br>3'pairing Seed | CTCTTT<br>Imperfect | | | M |
| Offset 6mer<br>73842 73862<br>5'-agtTTACATTCCTTCCCTTga-3' UTR<br>3'-cguAAAGAAAGAGAGGGAAaa-5' miRNA<br>3'pairing Seed | TCCCTT<br>Offset 6mer | | | M |
| Imperfect match<br>74070 74090<br>5'-tgATGTTCTGTAT-TCTCTTTg-3' UTR<br>3'-cgUA-AAGAAAGAGAGGGAAAa-5' miRNA<br>3'pairing Seed | CTCTTT<br>Imperfect | | | M |
| Imperfect match<br>74215 74237<br>5'-acATTTCCTATATATTTCCTTTc-3' UTR<br>3'-cgUAAA-GAAAGA-GAGGGAAAa-5' miRNA<br>3'pairing Seed | TCCTTT<br>Imperfect | | | M |

| 2D Structure | Local AU | Position | Conservation | Predicted By |
|---|---|---|---|---|
| 62 7mer-m8 82<br>5'-gaaGCTAAGAGAGGAAGGACc-3' UTR<br>3'-acaCCAGUCUGUCCUUCCUGu-5' miRNA<br>16 15 14 13 7 6 5 4 3 2<br>3'pairing Seed | 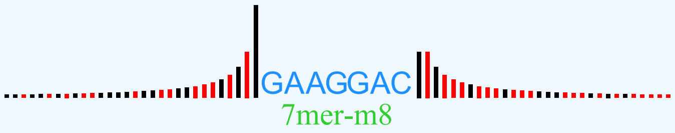 | 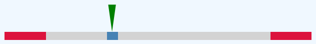 | | M T |
| 110 7mer-m8 130<br>5'-ttaacccactatGGAAGGACg-3' UTR<br>3'-acaccagucuguCCUUCCUGu-5' miRNA<br>16 15 14 13 7 6 5 4 3 2<br>3'pairing Seed | 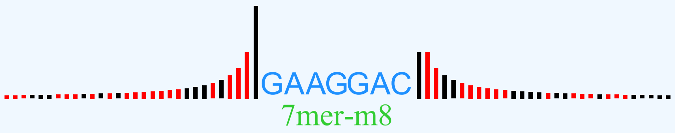 | 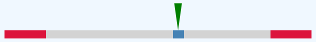 | | M T |

| 2D Structure | Local AU | Position | Conservation | Predicted By |
|---|---|---|---|---|
| Offset 6mer<br>56 78<br>5'-gacTTACTGATGATGTCTGAATt-3' UTR<br>\|:\|\| \| \| \|\|\|\|\|\|\|\|:<br>3'-cacAGUGUC-AGGACAGACUUGa-5' miRNA<br>16 15 14 13 7 6 5 4 3 2<br>3'pairing Seed | 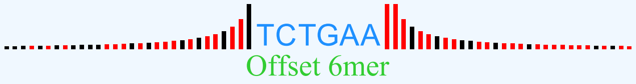 | 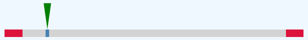 | ☐ | M |
| 8mer<br>149 170<br>5'-ccatcagaaaaagaTCTGAACa-3' UTR<br>\|\|\|\|\|\|\|<br>3'-cacaguguc aggacAGACUUGa-5' miRNA<br>16 15 14 13 7 6 5 4 3 2<br>3'pairing Seed | 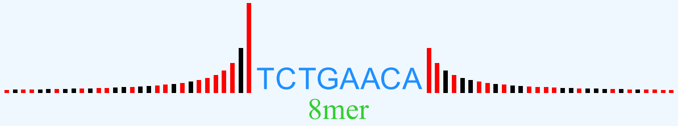 | 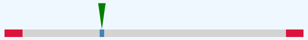 | ☐ | M T |

| 2D Structure | Local AU | Position | Conservation | Predicted By |
|---|---|---|---|---|
| 84 7mer-m8 105<br>5'-catTTGCTGTTCAGTCTGAACt-3' UTR<br>\|::\| \|\|:\| \|\|\|\|\|\|\|\|<br>3'-cacAGUGUCAGGACAGACUUGa-5' miRNA<br>16 15 14 13 7 6 5 4 3 2<br>3'pairing Seed | 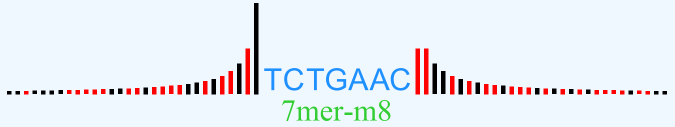 | 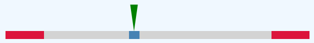 | | M T |

| 2D Structure | Local AU | Position | Conservation | Predicted By |
| --- | --- | --- | --- | --- |
| Imperfect match<br>176 197<br>5'-ccCAGCTCACAGAGCTTCCCCg-3' UTR<br>\| \|\|:\| \|\|\|\|:\|\| \|\|\|\|<br>3'-uaGACGGGGGUCUUGACGGGGu-5' miRNA<br>16 15 14 13 7 6 5 4 3 2<br>3'pairing Seed | 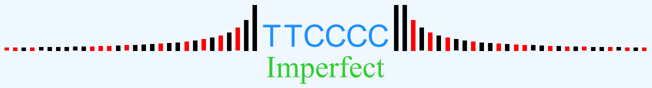 | 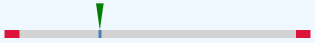 | | M |
| Offset 6mer<br>372 393<br>5'-tcCTGCTGTCGGTGCTGCCCat-3' UTR<br>\|\|\|\|: :\|:\| :\|\|\|\|\|\|<br>3'-uaGACGGGGGUCUUGACGGGGu-5' miRNA<br>16 15 14 13 7 6 5 4 3 2<br>3'pairing Seed | 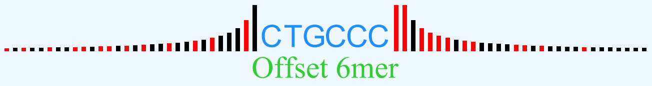 | 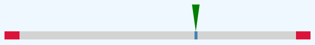 | | M |

| 2D Structure | Local AU | Position | Conservation | Predicted By |
|---|---|---|---|---|
| see site 235–259 below | 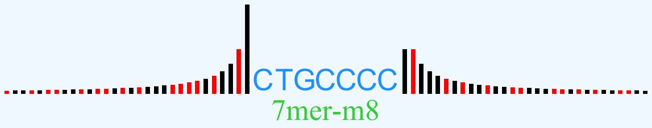  | 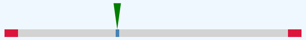 | ☐ | M T |
| see site 464–484 below | 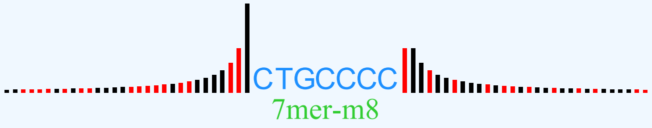  | 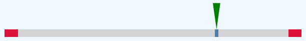 | ☐ | M T |
| see site 592–613 below | 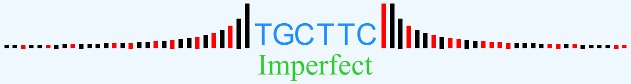  | 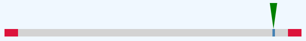 | ☐ | M |

```
235                7mer-m8  259
  5'-ctCTGGCTAATCAGCAGCTGCCCCc-3'  UTR
       ||| |:  :||| |:|||||||
  3'-uaGACGGG--GGUC-UUGACGGGGu-5'  miRNA
          16 15   14 13   7 6 5 4 3 2
          3'pairing        Seed
```

```
464                7mer-m8  484
  5'-tttgaaggCCA-ACCTGCCCCt-3'  UTR
             ||| | |||||||
  3'-uagacgggGGUCUUGACGGGGu-5'  miRNA
          16 15 14 13  7 6 5 4 3 2
          3'pairing     Seed
```

```
                  Imperfect
592                 match    613
  5'-tcgacTCCTCAGAGCTGCTTCt -3'  UTR
          :||:||||:||||::|
  3'-uagacGGGGUCUUGACGGGGu-5'  miRNA
          16 15 14 13  7 6 5 4 3 2
          3'pairing     Seed
```

| 2D Structure | Local AU | Position | Conservation | Predicted By |
|---|---|---|---|---|
| 224 8mer 247<br>5'-tcaataggattacgCTGAGGCCCa-3' UTR<br>\|\|\|\|\|\|\|\|\|<br>3'-aguggaggacucagGACUCCGGGa-5' miRNA<br>16 15 14 13 7 6 5 4 3 2<br>3'pairing Seed | GAGGCCCA<br>8mer | | | M T |
| Imperfect match<br>301 328<br>5'-tgACTTCCCTGAAGTCCTCAGATGCCCt -3' UTR<br>\|\|:\| \|\|\|\| \|\|\|\|\|\| \|\| \|\|\|\|<br>3'-agUGGA-GGAC-UCAGGA--CUCCGGGa-5' miRNA<br>16 15 14 13 7 6 5 4 3 2<br>3'pairing Seed | ATGCCC<br>Imperfect | | | M |
| 889 8mer 912<br>5'-cttTCCCTTGACAGGGGAGGCCCa-3' UTR<br>:\| \|:\|\|\| \|\|\|\|\|\|\|<br>3'-aguGGAGGACUCAGGACUCCGGGa-5' miRNA<br>16 15 14 13 7 6 5 4 3 2<br>3'pairing Seed | GAGGCCCA<br>8mer | | | M T |
| 2096 8mer 2119<br>5'-tcaataggattacgCTGAGGCCCa-3' UTR<br>\|\|\|\|\|\|\|\|\|<br>3'-aguggaggacucagGACUCCGGGa-5' miRNA<br>16 15 14 13 7 6 5 4 3 2<br>3'pairing Seed | GAGGCCCA<br>8mer | | | M T |
| Imperfect match<br>2173 2200<br>5'-tgACTTCCCTGAAGTCCTCAGATGCCCt -3' UTR<br>\|\|:\| \|\|\|\| \|\|\|\|\|\| \|\| \|\|\|\|<br>3'-agUGGA-GGAC-UCAGGA--CUCCGGGa-5' miRNA<br>16 15 14 13 7 6 5 4 3 2<br>3'pairing Seed | ATGCCC<br>Imperfect | | | M |

<table>
<tr><th>2D Structure</th><th>Local AU</th><th>Position</th><th>Conservation</th><th>Predicted By</th></tr>
<tr><td>155 7mer-m8 177<br>5'-gtggcAGAAGACATGGAGATCCc-3' UTR<br>||||:||| :|||||||<br>3'-ccuucUCUUUUGU-UCUCUAGGu-5' miRNA<br>16 15 14 13 7 6 5 4 3 2<br>3'pairing Seed</td><td>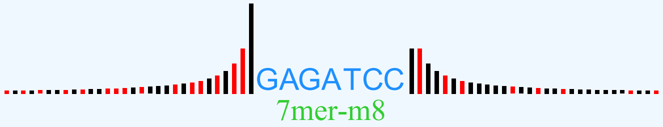</td><td>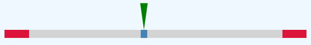</td><td>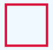</td><td>M T</td></tr>
</table>

<table>
<tr><th>2D Structure</th><th>Local AU</th><th>Position</th><th>Conservation</th><th>Predicted By</th></tr>
<tr><td>110 7mer-m8 132<br>5'-ttacGATGGGCTTGA-GCAGCTGc-3' UTR<br>||| ||| || |||||||<br>3'-gacaCUA-CCGUUCUCCGUCGACu-5' miRNA<br>16 15 14 13 7 6 5 4 3 2<br>3'pairing Seed</td><td>GCAGCTG<br>7mer-m8</td><td></td><td></td><td>M T</td></tr>
<tr><td>Offset 6mer<br>321 342<br>5'-atGTG-TGACCAGCGGCAGCTtg-3' UTR<br>||| || | || ||||||<br>3'-gaCACUACCGUUCUCCGUCGACu-5' miRNA<br>16 15 14 13 7 6 5 4 3 2<br>3'pairing Seed</td><td>GCAGCT<br>Offset 6mer</td><td></td><td></td><td>M</td></tr>
<tr><td>362 7mer-m8 384<br>5'-catccagatccctAGGCAGCTGg-3' UTR<br>|||||||||<br>3'-gacacuaccguucUCCGUCGACu-5' miRNA<br>16 15 14 13 7 6 5 4 3 2<br>3'pairing Seed</td><td>GCAGCTG<br>7mer-m8</td><td></td><td></td><td>M T</td></tr>
<tr><td>413 7mer-m8 437<br>5'-catTGA-GCCCAGTGTCCGCAGCTGc-3' UTR<br>||| | | || | |||||||<br>3'-gacACUACCGUUCUC---CGUCGACu-5' miRNA<br>16 15 14 13 7 6 5 4 3 2<br>3'pairing Seed</td><td>GCAGCTG<br>7mer-m8</td><td></td><td></td><td>M T</td></tr>
<tr><td>528 7mer-m8 550<br>5'-gttctgcatCGGGTCGCAGCTGc-3' UTR<br>|::| |||||||<br>3'-gacacuaccGUUCUCCGUCGACu-5' miRNA<br>16 15 14 13 7 6 5 4 3 2<br>3'pairing Seed</td><td>GCAGCTG<br>7mer-m8</td><td></td><td></td><td>M T</td></tr>
</table>

<table>
<tr><th>2D Structure</th><th>Local AU</th><th>Position</th><th>Conservation</th><th>Predicted By</th></tr>
<tr><td>110 7mer-m8 132<br>5'-ttacGATGGGCTTGA-GCAGCTGc-3' UTR<br>||| ||| || |||||||<br>3'-gacaCUA-CCGUUCUCCGUCGACu-5' miRNA<br>16 15 14 13 7 6 5 4 3 2<br>3'pairing Seed</td><td>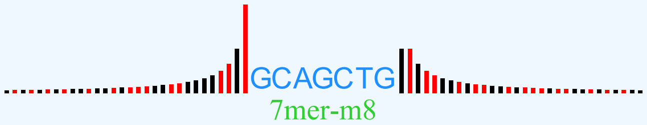</td><td>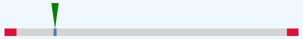</td><td></td><td>M T</td></tr>
<tr><td>Offset 6mer<br>321 342<br>5'-atGTG-TGACCAGCGGCAGCTtg-3' UTR<br>||| || | || ||||||<br>3'-gaCACUACCGUUCUCCGUCGACu-5' miRNA<br>16 15 14 13 7 6 5 4 3 2<br>3'pairing Seed</td><td>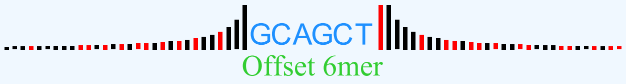</td><td>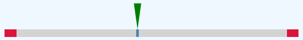</td><td></td><td>M</td></tr>
<tr><td>362 7mer-m8 384<br>5'-catccagatccctAGGCAGCTGg-3' UTR<br>||||||||||<br>3'-gacacuaccguucUCCGUCGACu-5' miRNA<br>16 15 14 13 7 6 5 4 3 2<br>3'pairing Seed</td><td>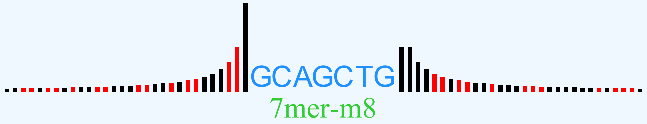</td><td>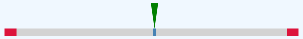</td><td></td><td>M T</td></tr>
<tr><td>413 7mer-m8 437<br>5'-catTGA-GCCCAGTGTCCGCAGCTGc-3' UTR<br>||| | | || | |||||||<br>3'-gacACUACCGUUCUC---CGUCGACu-5' miRNA<br>16 15 14 13 7 6 5 4 3 2<br>3'pairing Seed</td><td>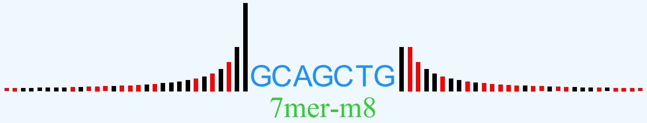</td><td>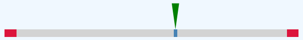</td><td></td><td>M T</td></tr>
<tr><td>528 7mer-m8 550<br>5'-gttctgcatCGGGTCGCAGCTGc-3' UTR<br>|::| |||||||<br>3'-gacacuaccGUUCUCCGUCGACu-5' miRNA<br>16 15 14 13 7 6 5 4 3 2<br>3'pairing Seed</td><td>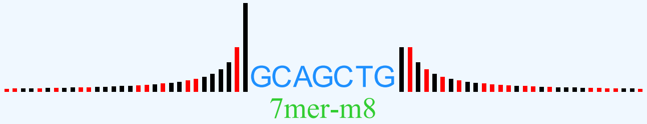</td><td>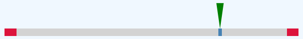</td><td></td><td>M T</td></tr>
</table>

| 2D Structure | Local AU | Position | Conservation | Predicted By |
|---|---|---|---|---|
| Imperfect match<br>119 142<br>5'-tcaTGTTGCCAGAGAGGCAGATGa -3' UTR<br>3'-gacACUACCGU-UCUCCGUCGACu-5' miRNA<br>16 15 14 13 7 6 5 4 3 2<br>3'pairing Seed | CAGATG<br>Imperfect | | | M |
| 8mer<br>203 225<br>5'-cgGCGAGTTCATACA-GCAGCTGa-3' UTR<br>3'-gaCACUACCGU-UCUCCGUCGACu-5' miRNA<br>16 15 14 13 7 6 5 4 3 2<br>3'pairing Seed | GCAGCTGA<br>8mer | | | M T |
| 8mer<br>495 517<br>5'-attacagacaAAGAGGCAGCTGa-3' UTR<br>3'-gacacuaccgUUCUCCGUCGACu-5' miRNA<br>16 15 14 13 7 6 5 4 3 2<br>3'pairing Seed | GCAGCTGA<br>8mer | | | M T |

<table>
<tr><th>2D Structure</th><th>Local AU</th><th>Position</th><th>Conservation</th><th>Predicted By</th></tr>
<tr><td>314 7mer-m8 335<br>5'-acatttggGGGACAAAAGAGGt-3' UTR<br>:||| |||||||<br>3'-gaucucggUCCUUCUUUCUCCu-5' miRNA<br>16 15 14 13 7 6 5 4 3 2<br>3'pairing Seed</td><td>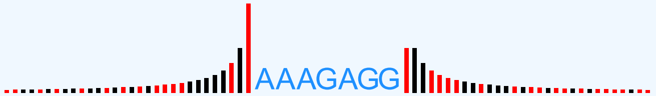<br>AAAGAGG<br>7mer-m8</td><td>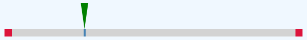</td><td></td><td>M T</td></tr>
<tr><td>807 Imperfect match 829<br>5'-aaAGAGACAGGAGAGAAGGGGGg -3' UTR<br>|||| ||||| ||||:|:||<br>3'-gaUCUCGGUCCU-UCUUUCUCCu-5' miRNA<br>16 15 14 13 7 6 5 4 3 2<br>3'pairing Seed</td><td>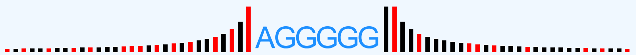<br>AGGGGG<br>Imperfect</td><td>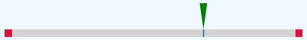</td><td></td><td>M</td></tr>
<tr><td>1141 7mer-A1 1160<br>5'-gaAGAG-GAGGAA-GAAGAGGa-3' UTR<br>|||| ||||| :||||||<br>3'-gaUCUCGGUCCUUCUUUCUCCu-5' miRNA<br>16 15 14 13 7 6 5 4 3 2<br>3'pairing Seed</td><td>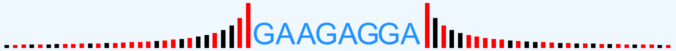<br>GAAGAGGA<br>7mer-A1</td><td>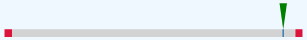</td><td></td><td>M T</td></tr>
</table>

| 2D Structure | Local AU | Position | Conservation | Predicted By |
|---|---|---|---|---|
| 7mer-m8<br>467 5'-ggAGCAGCCAGCAAGAAAGAGGt-3' 489 UTR<br>|| |||||| ||||||||||<br>3'-gaUC-UCGGUCCUUCUUUCUCCu-5' miRNA<br>16 15 14 13 · 7 6 5 4 3 2<br>3'pairing · Seed | 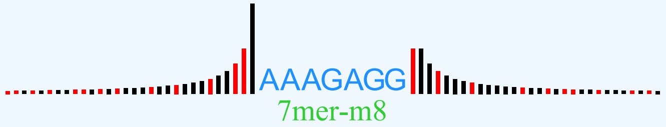 | 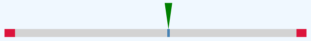 | | M T |
| Imperfect match<br>815 5'-ccGGAGCCAG--AGGAAGGGGa -3' 834 UTR<br>:||||||| ||:|||:||<br>3'-gaUCUCGGUCCUUCUUUCUCCu-5' miRNA<br>16 15 14 13 · 7 6 5 4 3 2<br>3'pairing · Seed | 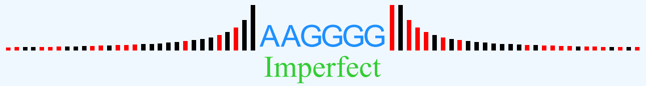 | 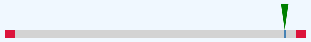 | | M |

<table>
<tr><th>2D Structure</th><th>Local AU</th><th>Position</th><th>Conservation</th><th>Predicted By</th></tr>
<tr><td>Offset<br>6mer<br>244 263<br>5'-cgAGAGCC--GAGCAAAGAGag-3' UTR<br>||||||  ||: ||||||<br>3'-gaUCUCGGUCCUUCUUUCUCCu-5' miRNA<br>16 15 14 13 7 6 5 4 3 2<br>3'pairing Seed</td><td>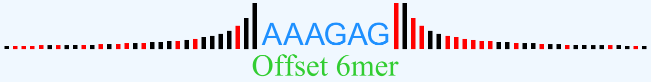<br></td><td>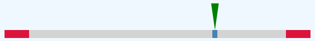</td><td></td><td>M</td></tr>
<tr><td>Imperfect<br>match<br>258 279<br>5'-agAGAGTTCGAATAAAGGAGGt-3' UTR<br>||||:: | |  ||:||||<br>3'-gaUCUCGGUCCUUCUUUCUCCu-5' miRNA<br>16 15 14 13 7 6 5 4 3 2<br>3'pairing Seed</td><td>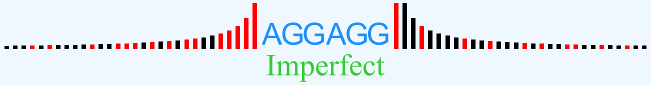<br></td><td>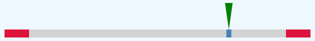</td><td></td><td>M</td></tr>
</table>

| 2D Structure | Local AU | Position | Conservation | Predicted By |
| --- | --- | --- | --- | --- |
| 490 7mer-m8 511<br>5'-agAAAATTACAGACAAAGAGGc-3' UTR<br>\| \| :: \| :\| \|\|\|\|\|\|\|<br>3'-gaUCUCGGUCCUUCUUUCUCCu-5' miRNA<br>16 15 14 13 7 6 5 4 3 2<br>3'pairing Seed | 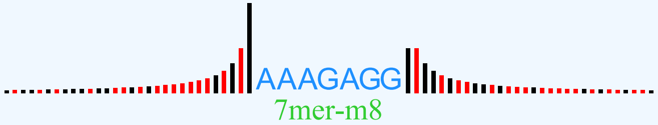 | 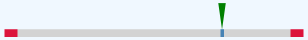 | | M T |
| 626 7mer-m8 649<br>5'-atACACCCAGAACCAGAAAGAGGt-3' UTR<br>\| \| \|\|\|\| \| \|\|\|\|\|\|\|\|\|<br>3'-gaUCUCGGUCCU--UCUUUCUCCu-5' miRNA<br>16 15 14 13 7 6 5 4 3 2<br>3'pairing Seed | 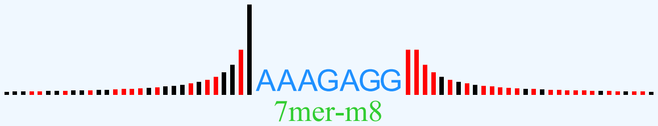 | 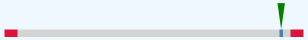 | | M T |

<table>
<tr><th>2D Structure</th><th>Local AU</th><th>Position</th><th>Conservation</th><th>Predicted By</th></tr>
<tr><td><pre>
129                 7mer-m8  148
  5'-tggcAGAGGAGGACAAGAAt-3'  UTR
         || |||:||||||||
  3'-gaucUCACCUUCUGUUCUUu-5'  miRNA
         16 15 14 13      7 6 5 4 3 2
          3'pairing      Seed
</pre></td><td>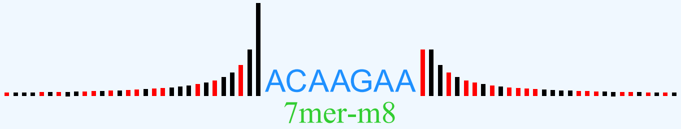
</td><td>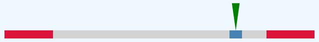</td><td></td><td>M T</td></tr>
</table>

| 2D Structure | Local AU | Position | Conservation | Predicted By |
| --- | --- | --- | --- | --- |
| 215 7mer-m8 234<br>5'-acAGAAACGTTTACAAGAAc-3' UTR<br>3'-gaUCUCACCUUCUGUUCUUu-5' miRNA<br>16 15 14 13 7 6 5 4 3 2<br>3'pairing Seed | 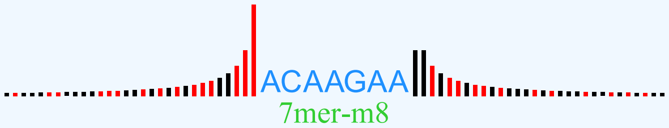 | | | M T |
| 223 7mer-m8 243<br>5'-gtttacaaGAACCACAAGAAg-3' UTR<br>3'-gaucucacCUU-CUGUUCUUu-5' miRNA<br>16 15 14 13 7 6 5 4 3 2<br>3'pairing Seed | 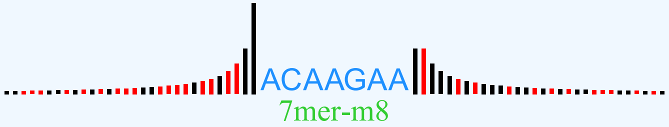 | | | M T |
| 414 7mer-m8 435<br>5'-ggtccaTGAAAGACACAAGAAg-3' UTR<br>3'-gaucucACCUUC--UGUUCUUu-5' miRNA<br>16 15 14 13 7 6 5 4 3 2<br>3'pairing Seed | 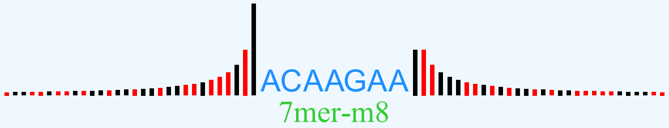 | | | M T |

| 2D Structure | Local AU | Position | Conservation | Predicted By |
| --- | --- | --- | --- | --- |
| 58 7mer-m8 80<br>5'-agTCGGGGCGGAT-GCCTCCCTCg-3' UTR<br>3'-agAGGACGGGUUAUCGGAGGGAGa-5' miRNA<br>16 15 14 13 7 6 5 4 3 2<br>3'pairing Seed | CTCCCTC<br>7mer-m8 | | | M T |
| 78 7mer-m8 101<br>5'-tcgCCGGAGCGGATGCCTCCCTCg-3' UTR<br>3'-agaGGACGGGUUAUCGGAGGGAGa-5' miRNA<br>16 15 14 13 7 6 5 4 3 2<br>3'pairing Seed | CTCCCTC<br>7mer-m8 | | | M T |

| 2D Structure | Local AU | Position | Conservation | Predicted By |
|---|---|---|---|---|
| 201 8mer 220<br>5'-cttacaAGAGGCAAAGATGa-3' UTR<br>3'-gaccccUCUCUAUUUCUACu-5' miRNA<br>16 15 14 13 7 6 5 4 3 2<br>3'pairing Seed | 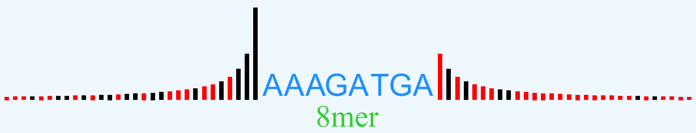 | 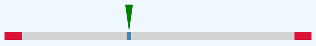 | | M T |
| 390 7mer-m8 409<br>5'-cttcttctAAACAAAGATGc-3' UTR<br>3'-gaccccucUCUAUUUCUACu-5' miRNA<br>16 15 14 13 7 6 5 4 3 2<br>3'pairing Seed | 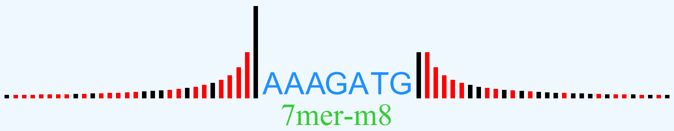 | 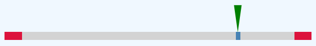 | | M T |

| 2D Structure | Local AU | Position | Conservation | Predicted By |
| --- | --- | --- | --- | --- |
| 59 7mer-m8 79<br>5'-gtcggggcggATGCCTCCCTc-3' UTR<br>3'-ugaccagaaaUAGGGAGGGAc-5' miRNA<br>16 15 14 13 7 6 5 4 3 2<br>3'pairing Seed | 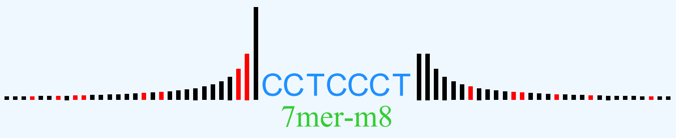 | 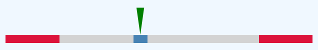 | | M T |
| 80 7mer-m8 100<br>5'-gccggagcggATGCCTCCCTc-3' UTR<br>3'-ugaccagaaaUAGGGAGGGAc-5' miRNA<br>16 15 14 13 7 6 5 4 3 2<br>3'pairing Seed | 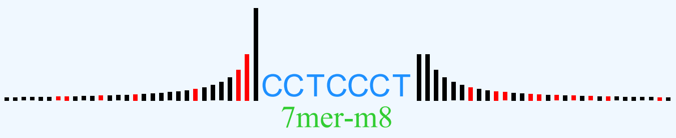 | 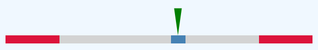 | | M T |

| 2D Structure | Local AU | Position | Conservation | Predicted By |
|---|---|---|---|---|
| 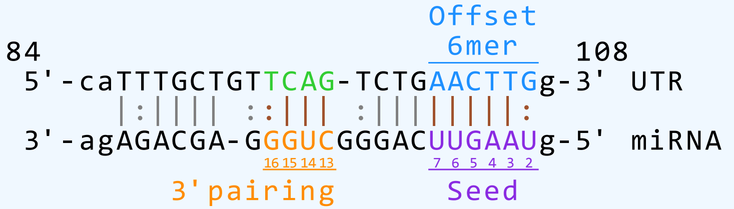 | 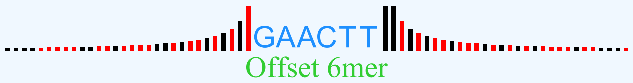 | 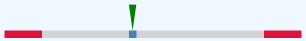 | 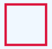 | 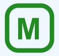 |

```
                          Offset
                           6mer
84                                  108
 5'-caTTTGCTGTTCAG-TCTGAACTTGg-3'  UTR
      |:|||| ::||| :||||||||:
 3'-agAGACGA-GGGUCGGGACUUGAAUg-5'  miRNA
             16 15 14 13  7 6 5 4 3 2
          3'pairing         Seed
```

| 2D Structure | Local AU | Position | Conservation | Predicted By |
|---|---|---|---|---|
| 175 8mer 195<br>5'-gtaattttAAACGGAGGCTGa-3' UTR<br>\|\| \|:\|\|\|\|\|\|\|<br>3'-gacucuucUUCGUCUCCGACa-5' miRNA<br>16 15 14 13 7 6 5 4 3 2<br>3'pairing Seed | 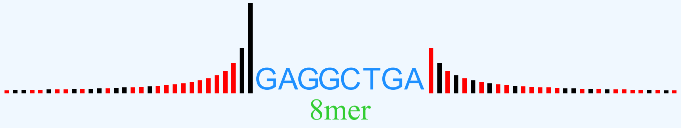 | 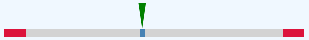 | | M T |
| Imperfect match<br>226 247<br>5'-gcGTGGAGAAGGCAGAGGATGg-3' UTR<br>\| \|:\|\|\|\| \|\|\|\|\|\|\|\| \|\|<br>3'-gaCUCUUCUU-CGUCUCCGACa-5' miRNA<br>16 15 14 13 7 6 5 4 3 2<br>3'pairing Seed | 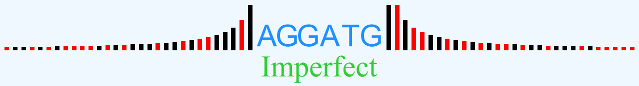 | 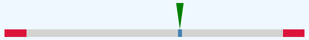 | | M |

| 2D Structure | Local AU | Position | Conservation | Predicted By |
| --- | --- | --- | --- | --- |
| 148 8mer 169<br>5'-atGATTTCCTCAG-GCCTCCAGa-3' UTR<br>\| \|:\| \|:\| \| \|\|\|\|\|\|\|\|<br>3'-gaCAAGACGGGACACGGAGGUCg-5' miRNA<br>16 15 14 13 7 6 5 4 3 2<br>3'pairing Seed | 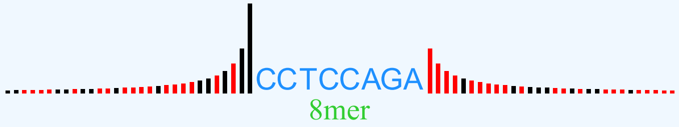 | 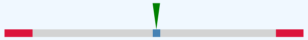 | | M T |

<table>
<tr><th>2D Structure</th><th>Local AU</th><th>Position</th><th>Conservation</th><th>Predicted By</th></tr>
<tr><td>Imperfect match<br>320 340<br>5'-ggGGGACAAAAGA-GGTGGAtt-3' UTR<br>|||||| |||| :|||||<br>3'-gaCCCUGUCUUCUCUCACCUCu-5' miRNA<br>16 15 14 13 7 6 5 4 3 2<br>3'pairing Seed</td><td>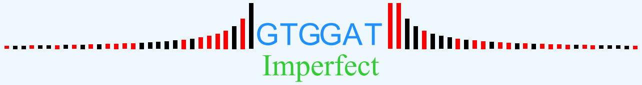</td><td>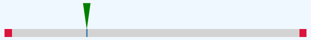</td><td></td><td>M</td></tr>
<tr><td>6mer<br>813 835<br>5'-acaGGAGAGAAGGGGGGTGGAGc-3' UTR<br>||| |||| |:|:||||||<br>3'-gacCCUGUCUU-CUCUCACCUCu-5' miRNA<br>16 15 14 13 7 6 5 4 3 2<br>3'pairing Seed</td><td>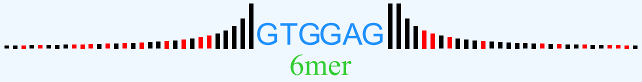</td><td>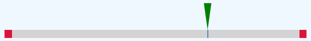</td><td></td><td>M</td></tr>
<tr><td>Imperfect match<br>926 948<br>5'-ttgccACAGCAGAGAGTGGCAGc-3' UTR<br>|||| |||||||||| ||<br>3'-gacccUGUCUUCUCUCACC-UCu-5' miRNA<br>16 15 14 13 7 6 5 4 3 2<br>3'pairing Seed</td><td>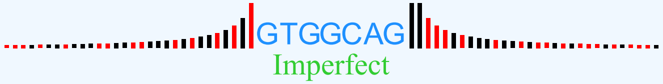</td><td>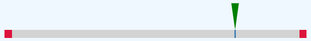</td><td></td><td>M</td></tr>
</table>

<table>
<tr><th>2D Structure</th><th>Local AU</th><th>Position</th><th>Conservation</th><th>Predicted By</th></tr>
<tr><td>125 7mer-m8 147<br>5'-aagatgtccttGAGCAGTGGAGc-3' UTR<br>||| ||||||<br>3'-gacccugucuuCUC-UCACCUCu-5' miRNA<br>16 15 14 13 7 6 5 4 3 2<br>3'pairing Seed</td><td>AGTGGAG<br>7mer-m8</td><td></td><td></td><td>M T</td></tr>
<tr><td>Offset 6mer<br>149 170<br>5'-tgcGGAGGGAAAGGAGTGGAtg-3' UTR<br>||| :||| :|||||||<br>3'-gacCCUGUCUUCUCUCACCUcu-5' miRNA<br>16 15 14 13 7 6 5 4 3 2<br>3'pairing Seed</td><td>AGTGGA<br>Offset 6mer</td><td></td><td></td><td>M</td></tr>
<tr><td>Imperfect match<br>180 201<br>5'-ttGAGACATACGACAGTGGGGa -3' UTR<br>| |||| | || ||||:|<br>3'-gaCCCUGUCUUCUCUCACCUCu-5' miRNA<br>16 15 14 13 7 6 5 4 3 2<br>3'pairing Seed</td><td>GTGGGG<br>Imperfect</td><td></td><td></td><td>M</td></tr>
</table>

| 2D Structure | Local AU | Position | Conservation | Predicted By |
|---|---|---|---|---|
| Offset 6mer<br>595 613<br>5'-caaaaAGGA-AAAGGAGAAc-3' UTR<br>3'-gauccUCCUCUUUCCUCUCu-5' miRNA<br>16 15 14 13 7 6 5 4 3 2<br>3'pairing Seed | 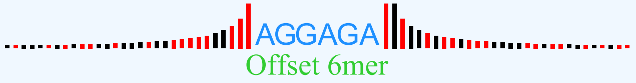 AGGAGA Offset 6mer | 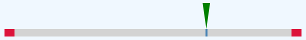 | | M |
| 8mer<br>621 640<br>5'-ctAAGGGCAAGAAGGAGAGa-3' UTR<br>3'-gaUCCUCCUCUUUCCUCUCu-5' miRNA<br>16 15 14 13 7 6 5 4 3 2<br>3'pairing Seed | 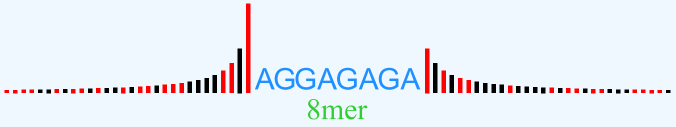 AGGAGAGA 8mer | 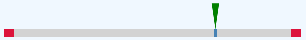 | | M T |
| Imperfect match<br>814 836<br>5'-gccGGAGCCAGAGGAAGGGGAGa -3' UTR<br>3'-gauCCUC---CUCUUUCCUCUCu-5' miRNA<br>16 15 14 13 7 6 5 4 3 2<br>3'pairing Seed | 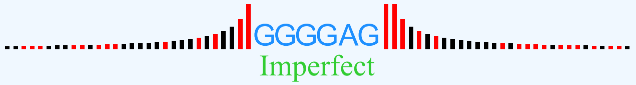 GGGGAG Imperfect | 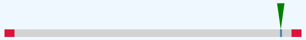 | | M |

| 2D Structure | Local AU | Position | Conservation | Predicted By |
|---|---|---|---|---|
| 7mer-m8<br>644 664<br>5'-ggtgaaTCCTTTC--CTTCCCTc-3' UTR<br>3'-guuaaaAGGAGAGUUGAAGGGAc-5' miRNA<br>3'pairing Seed | CTTCCCT<br>7mer-m8 | | ☐ | M T |
| 7mer-m8<br>697 719<br>5'-ccccagcCTTCTCTTCTTCCCTg-3' UTR<br>3'-guuaaaaGGAGAGUUGAAGGGAc-5' miRNA<br>3'pairing Seed | CTTCCCT<br>7mer-m8 | | ☐ | M T |
| Imperfect match<br>1438 1459<br>5'-gaATTTTGCTCT-ACCTTCACTa-3' UTR<br>3'-guUAAAAGGAGAGUUGAAGGGAc-5' miRNA<br>3'pairing Seed | TTCACT<br>Imperfect | | ☐ | M |
| Imperfect match<br>4292 4315<br>5'-ccATTATCCCTCACGACTTTCCaa-3' UTR<br>3'-guUAA-AAGGAGAGUUGAAGGGac-5' miRNA<br>3'pairing Seed | TTTCCA<br>Imperfect | | ☐ | M |
| Imperfect match<br>5451 5473<br>5'-ggATTTGGGGCTGATCTTTCCTg-3' UTR<br>3'-guUAAAAGGAGAGUUGAAGGGAc-5' miRNA<br>3'pairing Seed | TTTCCT<br>Imperfect | | ☐ | M |
| Imperfect match<br>6107 6128<br>5'-tccTCTTCCTC-CATCTTCTCTg-3' UTR<br>3'-guuAAAAGGAGAGUUGAAGGGAc-5' miRNA<br>3'pairing Seed | TTCTCT<br>Imperfect | | ☐ | M |
| Imperfect match<br>7971 7996<br>5'-tgtTTCTCAGCTCTAGGACTTCTCTg-3' UTR<br>3'-guuAAAAG--GAGA-GUUGAAGGGAc-5' miRNA<br>3'pairing Seed | TTCTCT<br>Imperfect | | ☐ | M |
| Imperfect match<br>15509 15536<br>5'-tgATTTTCAAAATCTGTTAGCTTCTCTg-3' UTR<br>3'-guUAAAAG-----GAGAGUUGAAGGGAc-5' miRNA<br>3'pairing Seed | TTCTCT<br>Imperfect | | ☐ | M |
| Imperfect match<br>16429 16451<br>5'-tctTTTTCCTTTCCTCTTCTCTa-3' UTR<br>3'-guuAAAAGGAGAGUUGAAGGGAc-5' miRNA<br>3'pairing Seed | TTCTCT<br>Imperfect | | ☐ | M |
| Imperfect match<br>24832 24854<br>5'-aatcccTCATCATATCTTTCCTt-3' UTR<br>3'-guuaaaAGGAGAGUUGAAGGGAc-5' miRNA<br>3'pairing Seed | TTTCCT<br>Imperfect | | ☐ | M |
| 6mer<br>26618 26640<br>5'-tgGTCTGCCATTTGATTTCCCTt-3' UTR<br>3'-guUAAAAGGAGAGUUGAAGGGAc-5' miRNA<br>3'pairing Seed | TTCCCT<br>6mer | | ☐ | M |
| Imperfect match<br>28206 28228<br>5'-gaACTCTCTTGTTGACTTCTCTa-3' UTR<br>3'-guUAAAAGGAGAGUUGAAGGGAc-5' miRNA<br>3'pairing Seed | TTCTCT<br>Imperfect | | ☐ | M |
| Imperfect match<br>28349 28372<br>5'-cgtatacCCTCTTGAGCTTCTCTg-3' UTR<br>3'-guuaaaaGGAGAG-UUGAAGGGAc-5' miRNA<br>3'pairing Seed | TTCTCT<br>Imperfect | | ☐ | M |
| Imperfect match<br>28799 28820<br>5'-tcATTTTTCT-TTACTTTTCCTa-3' UTR<br>3'-guUAAAAGGAGAGUUGAAGGGAc-5' miRNA<br>3'pairing Seed | TTTCCT<br>Imperfect | | ☐ | M |
| 6mer<br>29086 29108<br>5'-cctTTTGTCTCCCAGGTTCCCTg-3' UTR<br>3'-guuAAAAGGAGAGUUGAAGGGAc-5' miRNA<br>3'pairing Seed | TTCCCT<br>6mer | | ☐ | M |
| Imperfect match<br>32766 32789<br>5'-ggATGTTTCTTATTGGCTTGCCTc-3' UTR<br>3'-guUA-AAAGGAGAGUUGAAGGGAc-5' miRNA<br>3'pairing Seed | TTGCCT<br>Imperfect | | ☐ | M |
| Imperfect match<br>34485 34508<br>5'-caAGTTTCCAGTCTAACTTCTTTa-3' UTR<br>3'-guUAAAAGGAGAG-UUGAAGGGAc-5' miRNA<br>3'pairing Seed | TTCTTT<br>Imperfect | | ☐ | M |
| Imperfect match<br>35318 35341<br>5'-ggccatcggaCTCGAACTTCCTTa-3' UTR<br>3'-guuaaaaggaGAG-UUGAAGGGAc-5' miRNA<br>3'pairing Seed | TTCCTT<br>Imperfect | | ☐ | M |
| Imperfect match<br>35339 35362<br>5'-ttATTCTTCCTTCAAACTTCACTc-3' UTR<br>3'-guUAA-AAGGAGAGUUGAAGGGAc-5' miRNA<br>3'pairing Seed | TTCACT<br>Imperfect | | ☐ | M |
| Imperfect match<br>38567 38589<br>5'-acACTTTTCTTTCTTCTTTCTTg-3' UTR<br>3'-guUAAAAGGAGAGUUGAAGGGAc-5' miRNA<br>3'pairing Seed | TTTCTT<br>Imperfect | | ☐ | M |
| 8mer<br>39347 39370<br>5'-ccATTTTAGGTTGCATCTTCCCTa-3' UTR<br>3'-guUAAAA-GGAGAGUUGAAGGGAc-5' miRNA<br>3'pairing Seed | CTTCCCTA<br>8mer | | ☐ | M T |
| 6mer<br>41080 41102<br>5'-tcACTTTGATTTGCATTTCCCTg-3' UTR<br>3'-guUAAAAGGAGAGUUGAAGGGAc-5' miRNA<br>3'pairing Seed | TTCCCT<br>6mer | | ☐ | M |
| Imperfect match<br>43868 43888<br>5'-aatccTTTCTTTC--CTTCCTTt-3' UTR<br>3'-guuaaAAGGAGAGUUGAAGGGAc-5' miRNA<br>3'pairing Seed | TTCCTT<br>Imperfect | | ☐ | M |
| Imperfect match<br>43887 43909<br>5'-ttcTTTCCTTCTTTTCTTTCCTt-3' UTR<br>3'-guuAAAAGGAGAGUUGAAGGGAc-5' miRNA<br>3'pairing Seed | TTTCCT<br>Imperfect | | ☐ | M |
| Imperfect match<br>43933 43955<br>5'-cctTTTTCCTTTTTTCTTTCCTt-3' UTR<br>3'-guuAAAAGGAGAGUUGAAGGGAc-5' miRNA<br>3'pairing Seed | TTTCCT<br>Imperfect | | ☐ | M |
| Imperfect match<br>43959 43979<br>5'-ttcTTTTCCTTTC--CTTTCCTt-3' UTR<br>3'-guuAAAAGGAGAGUUGAAGGGAc-5' miRNA<br>3'pairing Seed | TTTCCT<br>Imperfect | | ☐ | M |
| Imperfect match<br>43974 43994<br>5'-ttccTTTCTTTTC--CTTTCCTt-3' UTR<br>3'-guuaAAAGGAGAGUUGAAGGGAc-5' miRNA<br>3'pairing Seed | TTTCCT<br>Imperfect | | ☐ | M |
| Imperfect match<br>43991 44013<br>5'-cctTTTTCCTATCCTCTTTCCTt-3' UTR<br>3'-guuAAAAGGAGAGUUGAAGGGAc-5' miRNA<br>3'pairing Seed | TTTCCT<br>Imperfect | | ☐ | M |
| Imperfect match<br>44012 44034<br>5'-tttccTTTCTCTTTTCTTTCCTt-3' UTR<br>3'-guuaaAAGGAGAGUUGAAGGGAc-5' miRNA<br>3'pairing Seed | TTTCCT<br>Imperfect | | ☐ | M |
| Imperfect match<br>44031 44051<br>5'-cctTTTTCCTTTC--CTTTCCTt-3' UTR<br>3'-guuAAAAGGAGAGUUGAAGGGAc-5' miRNA<br>3'pairing Seed | TTTCCT<br>Imperfect | | ☐ | M |
| 6mer<br>44056 44078<br>5'-ttccTTTCCTTTCCTTTTCCCTt-3' UTR<br>3'-guuaAAAGGAGAGUUGAAGGGAc-5' miRNA<br>3'pairing Seed | TTCCCT<br>6mer | | ☐ | M |
| Imperfect match<br>44073 44095<br>5'-tcccTTTCCTTTCCTCTTTCCTt-3' UTR<br>3'-guuaAAAGGAGAGUUGAAGGGAc-5' miRNA<br>3'pairing Seed | TTTCCT<br>Imperfect | | ☐ | M |
| Imperfect match<br>44175 44198<br>5'-ctcTGTTTCTGACATACTTTCCTg-3' UTR<br>3'-guuAAAAGGAGAGU-UGAAGGGAc-5' miRNA<br>3'pairing Seed | TTTCCT<br>Imperfect | | ☐ | M |
| 8mer<br>45609 45633<br>5'-atATTTATGTTCTTCCACTTCCCTa-3' UTR<br>3'-guUAAA-AGGAG-AGUUGAAGGGAc-5' miRNA<br>3'pairing Seed | CTTCCCTA<br>8mer | | ☐ | M T |
| Imperfect match<br>45644 45666<br>5'-cacaaTTCTTCCCAATTTCCCca-3' UTR<br>3'-guuaaAAGGAGAGUUGAAGGGac-5' miRNA<br>3'pairing Seed | TTCCCC<br>Imperfect | | ☐ | M |
| Imperfect match<br>49411 49433<br>5'-aatgaaTCCTCTATACTTCTCTa-3' UTR<br>3'-guuaaaAGGAGAGUUGAAGGGAc-5' miRNA<br>3'pairing Seed | TTCTCT<br>Imperfect | | ☐ | M |
| Imperfect match<br>51200 51221<br>5'-gaATTTTGCTCT-ACCTTCACTa-3' UTR<br>3'-guUAAAAGGAGAGUUGAAGGGAc-5' miRNA<br>3'pairing Seed | TTCACT<br>Imperfect | | ☐ | M |
| Imperfect match<br>53805 53828<br>5'-ccATTATCCCTCACGACTTTCCaa-3' UTR<br>3'-guUAA-AAGGAGAGUUGAAGGGac-5' miRNA<br>3'pairing Seed | TTTCCA<br>Imperfect | | ☐ | M |
| Imperfect match<br>54944 54966<br>5'-ggATTTGGGGCTGATCTTTCCTg-3' UTR<br>3'-guUAAAAGGAGAGUUGAAGGGAc-5' miRNA<br>3'pairing Seed | TTTCCT<br>Imperfect | | ☐ | M |
| Imperfect match<br>57611 57632<br>5'-tccTCTTCCTC-CATCTTCTCTg-3' UTR<br>3'-guuAAAAGGAGAGUUGAAGGGAc-5' miRNA<br>3'pairing Seed | TTCTCT<br>Imperfect | | ☐ | M |
| Imperfect match<br>61675 61697<br>5'-cttctgcTTTCTTTGCTTCCTTc-3' UTR<br>3'-guuaaaaGGAGAGUUGAAGGGAc-5' miRNA<br>3'pairing Seed | TTCCTT<br>Imperfect | | ☐ | M |
| Imperfect match<br>62408 62431<br>5'-caAGTTTCCAGTCTAACTTCTTTa-3' UTR<br>3'-guUAAAAGGAGAG-UUGAAGGGAc-5' miRNA<br>3'pairing Seed | TTCTTT<br>Imperfect | | ☐ | M |
| 8mer<br>67620 67643<br>5'-ccATTTTAGGTTGCATCTTCCCTa-3' UTR<br>3'-guUAAAA-GGAGAGUUGAAGGGAc-5' miRNA<br>3'pairing Seed | CTTCCCTA<br>8mer | | ☐ | M T |
| 6mer<br>72293 72315<br>5'-ttGTTTTGATTTGCATTTCCCTg-3' UTR<br>3'-guUAAAAGGAGAGUUGAAGGGAc-5' miRNA<br>3'pairing Seed | TTCCCT<br>6mer | | ☐ | M |
| 8mer<br>73453 73473<br>5'-ttATGTTCTTCT--ACTTCCCTa-3' UTR<br>3'-guUAAAAGGAGAGUUGAAGGGAc-5' miRNA<br>3'pairing Seed | CTTCCCTA<br>8mer | | ☐ | M T |
| Imperfect match<br>73484 73506<br>5'-cacaaTTCTTCCCAATTTCCCca-3' UTR<br>3'-guuaaAAGGAGAGUUGAAGGGac-5' miRNA<br>3'pairing Seed | TTCCCC<br>Imperfect | | ☐ | M |
| 7mer-m8<br>75591 75615<br>5'-ggaccTTCTGTCATCCACTTCCCTt-3' UTR<br>3'-guuaaAAGG-AG-AGUUGAAGGGAc-5' miRNA<br>3'pairing Seed | CTTCCCT<br>7mer-m8 | | ☐ | M T |
| 8mer<br>75655 75679<br>5'-gtATTTTATGGATTTTACTTCCCTa-3' UTR<br>3'-guUAAAAGG--AGAGUUGAAGGGAc-5' miRNA<br>3'pairing Seed | CTTCCCTA<br>8mer | | ☐ | M T |
| Offset 6mer<br>76005 76027<br>5'-ctcaTGTCCTCCCATCTTCCCct-3' UTR<br>3'-guuaAAAGGAGAGUUGAAGGGac-5' miRNA<br>3'pairing Seed | CTTCCC<br>Offset 6mer | | ☐ | M |

<table>
<tr><th>2D Structure</th><th>Local AU</th><th>Position</th><th>Conservation</th><th>Predicted By</th></tr>
<tr><td>410 8mer 431<br>5'-agGTGGTCCATGAAAGACACAa-3' UTR<br>| |: ||| || |||||||<br>3'-gaCCCUCGGU-CUGUCUGUGUu-5' miRNA<br>16 15 14 13 7 6 5 4 3 2<br>3'pairing Seed</td><td>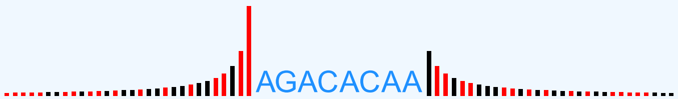</td><td>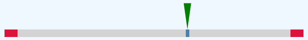</td><td></td><td>M T</td></tr>
<tr><td>482 7mer-m8 504<br>5'-ttaGGAGTCATAAGAAGACACAc-3' UTR<br>||||:|| | |||||||<br>3'-gacCCUCGGUCU--GUCUGUGUu-5' miRNA<br>16 15 14 13 7 6 5 4 3 2<br>3'pairing Seed</td><td>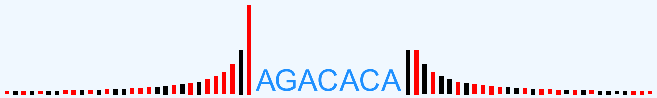</td><td>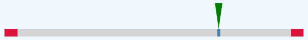</td><td></td><td>M T</td></tr>
</table>

<table>
<tr><th>2D Structure</th><th>Local AU</th><th>Position</th><th>Conservation</th><th>Predicted By</th></tr>
<tr><td>242 8mer 262<br>5'-ctccaagAGCC-CTCCCCCCAa-3' UTR<br>|||| |:|||||||<br>3'-gauuggaUCGGAGGGGGGGGUg-5' miRNA<br>16 15 14 13 7 6 5 4 3 2<br>3'pairing Seed</td><td>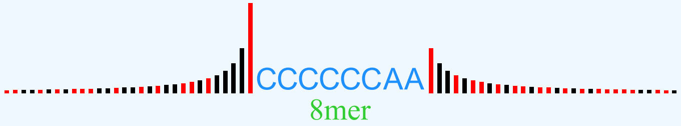
</td><td>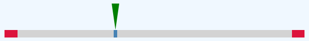</td><td></td><td>M T</td></tr>
<tr><td>Imperfect match<br>329 350<br>5'-ccctggaAGCTACTCTCCCCAa -3' UTR<br>|||: |:|:||||||<br>3'-gauuggaUCGGAGGGGGGGGUg-5' miRNA<br>16 15 14 13 7 6 5 4 3 2<br>3'pairing Seed</td><td>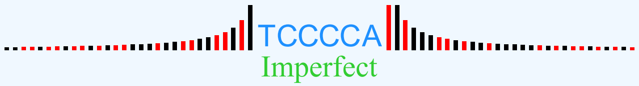
</td><td>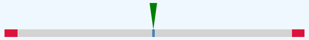</td><td></td><td>M</td></tr>
</table>

<table>
<tr><th>2D Structure</th><th>Local AU</th><th>Position</th><th>Conservation</th><th>Predicted By</th></tr>
<tr><td>143 7mer-m8 167<br>5'-ttgcTCACCATC-AGGGTCTCCTCAt-3' UTR<br>||| | || ||||||||<br>3'-cggaAGUCGGAGAAGGAAGAGGAGUg-5' miRNA<br>16 15 14 13 7 6 5 4 3 2<br>3'pairing Seed</td><td>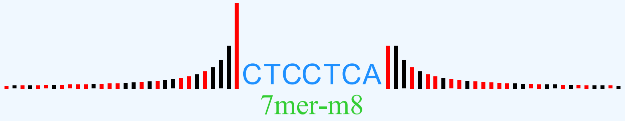<br></td><td>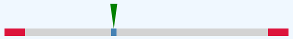</td><td></td><td>M T</td></tr>
<tr><td>197 8mer 224<br>5'-atggTC-GCCTAGTGCCCCTCCTCCTCAa-3' UTR<br>|| |||| | ||| |||||||<br>3'-cggaAGUCGGAGAA---GGAAGAGGAGUg-5' miRNA<br>16 15 14 13 7 6 5 4 3 2<br>3'pairing Seed</td><td>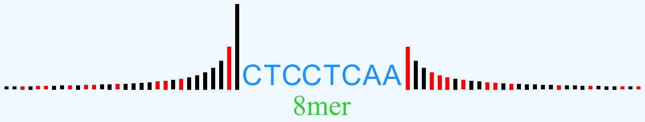<br></td><td>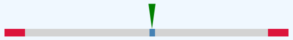</td><td></td><td>M T</td></tr>
</table>

| 2D Structure | Local AU | Position | Conservation | Predicted By |
|---|---|---|---|---|

```
149                   7mer-m8  169
  5'-ccAGGCCGCTCC----ATCCTCCAg-3'  UTR
       |||||:|:||    ||||||||
  3'-ggUCCGGUGGGGACGCUAGGAGGUc-5'  miRNA
              16 15 14 13   7 6 5 4 3 2
          3'pairing       Seed
```

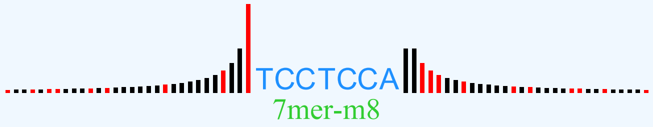


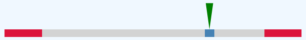

M T

| 2D Structure | Local AU | Position | Conservation | Predicted By |
|---|---|---|---|---|
| 198 7mer-m8 225<br>5'-gtGAATACAAAACCTTGCAGCCTTCCAg-3' UTR<br>\|\|\|\| \|\| \| \|\|\| \|\|\|\|\|\|\|<br>3'-ggCUUA-GUCAU---ACG--GGAAGGUu-5' miRNA<br>16 15 14 13 7 6 5 4 3 2<br>3'pairing Seed | 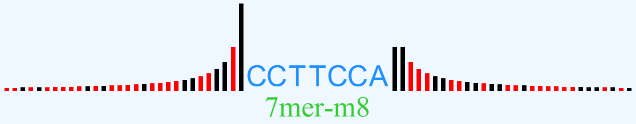 | 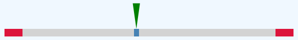 | | M T |
| 217 7mer-m8 237<br>5'-gccttcCAG-GTTACCTTCCAc-3' UTR<br>\|\|\| :\| \|\|\|\|\|\|\|<br>3'-ggcuuaGUCAUACGGGAAGGUu-5' miRNA<br>16 15 14 13 7 6 5 4 3 2<br>3'pairing Seed | 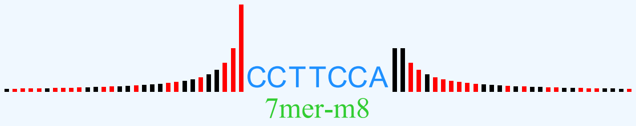 | 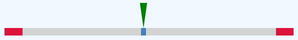 | | M T |

<table>
<tr><th>2D Structure</th><th>Local AU</th><th>Position</th><th>Conservation</th><th>Predicted By</th></tr>
<tr><td>141 7mer-m8 161<br>5'-acATCGCAGCT-TTCCTCAGTc-3' UTR<br>|| |||||: ::|||||||<br>3'-ugUAUCGUCGGUGGGGAGUCAu-5' miRNA<br>16 15 14 13 7 6 5 4 3 2<br>3'pairing Seed</td><td>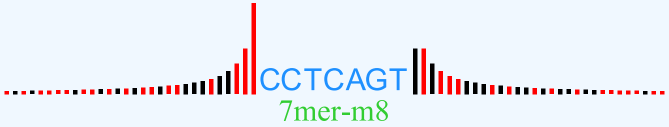</td><td>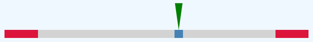</td><td></td><td>M T</td></tr>
</table>

<table>
<tr><th>2D Structure</th><th>Local AU</th><th>Position</th><th>Conservation</th><th>Predicted By</th></tr>
<tr><td>Imperfect match<br>76 95<br>5'-ccACAGAAGC--CCCCTCGGTc -3' UTR<br>|  ||  |||  ||||||:||<br>3'-ugUAUCGUCGGUGGGGAGUCAu-5' miRNA<br>16 15 14 13 7 6 5 4 3 2<br>3'pairing Seed</td><td>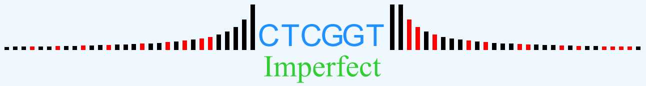</td><td>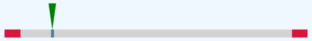</td><td></td><td>M</td></tr>
<tr><td>7mer-m8<br>120 140<br>5'-taATCACA-CTCCTCCTCAGTt-3' UTR<br>||  ||  |:  |:|||||||<br>3'-ugUAUCGUCGGUGGGGAGUCAu-5' miRNA<br>16 15 14 13 7 6 5 4 3 2<br>3'pairing Seed</td><td>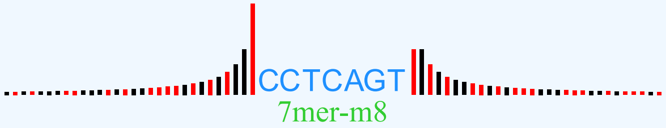</td><td>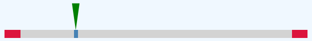</td><td></td><td>M T</td></tr>
</table>

| 2D Structure | Local AU | Position | Conservation | Predicted By |
| --- | --- | --- | --- | --- |
| 254 7mer-m8 275<br>5'-gcGACAGG-GTCACAAGAGGTCc-3' UTR<br>3'-gaCCGUCCUCUAUG-UCUCCAGu-5' miRNA<br>16 15 14 13 7 6 5 4 3 2<br>3'pairing Seed | 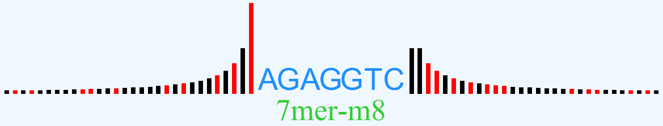 | 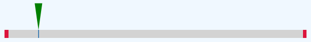 | ☐ | M T |
| 1408 7mer-m8 1429<br>5'-aacatagacctTACAGAGGTCt-3' UTR<br>3'-gaccguccucuAUGUCUCCAGu-5' miRNA<br>16 15 14 13 7 6 5 4 3 2<br>3'pairing Seed | 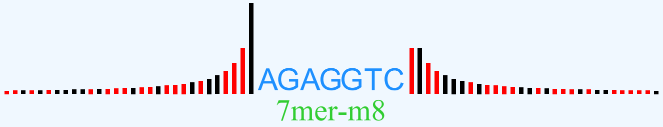 | 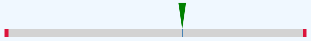 | ☐ | M T |
| Offset 6mer<br>1709 1730<br>5'-ctGGTAGTA-ATACAAGAGGTaa-3' UTR<br>3'-gaCCGUCCUCUAUG-UCUCCAgu-5' miRNA<br>16 15 14 13 7 6 5 4 3 2<br>3'pairing Seed | 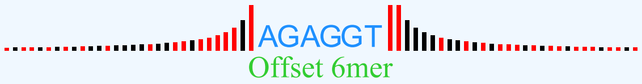 | 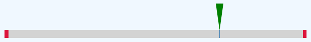 | ☐ | M |
| 2126 7mer-m8 2147<br>5'-gcGACAGGGTCAGAAGAGGTCc-3' UTR<br>3'-gaCCGUCCUCUAUGUCUCCAGu-5' miRNA<br>16 15 14 13 7 6 5 4 3 2<br>3'pairing Seed | 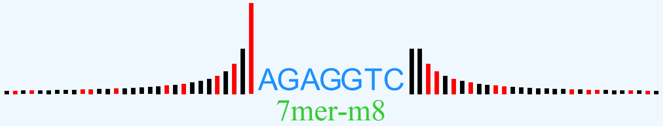 | 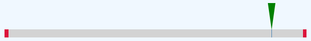 | ☐ | M T |

| 2D Structure | Local AU | Position | Conservation | Predicted By |
|---|---|---|---|---|
| 151 7mer-m8 172<br>5'-cacaAAGTGATCCACAGTGAGg-3' UTR<br>\|\|:\|\|\|\|   \|\|\|\|\|\|\|<br>3'-gacgUUUACUAGUAGUCACUCu-5' miRNA<br>16 15 14 13   7 6 5 4 3 2<br>3'pairing   Seed | 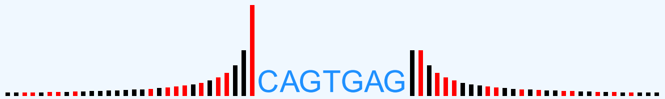<br>CAGTGAG<br>7mer-m8 | 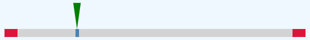 | ☐ | M T |
| 322 8mer 343<br>5'-ctGTTAATCCACAACAGTGAGa-3' UTR<br>\|:  \|\|   \|\|  \|\|\|\|\|\|\|<br>3'-gaCGUUUACUAGUAGUCACUCu-5' miRNA<br>16 15 14 13   7 6 5 4 3 2<br>3'pairing   Seed | 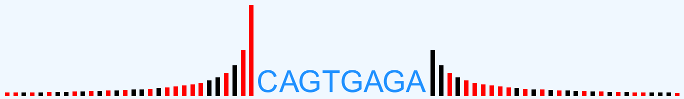<br>CAGTGAGA<br>8mer | 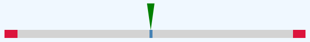 | ☐ | M T |

<table>
<tr><th>2D Structure</th><th>Local AU</th><th>Position</th><th>Conservation</th><th>Predicted By</th></tr>
<tr><td>Imperfect match<br>70 92<br>5'-cccCAGCAAGATCCTCATAGGCt-3' UTR<br>|||| |:|:| |||:||||<br>3'-ugaGUCGGUUUGGUAGUGUCCGu-5' miRNA<br>16 15 14 13 7 6 5 4 3 2<br>3'pairing Seed</td><td>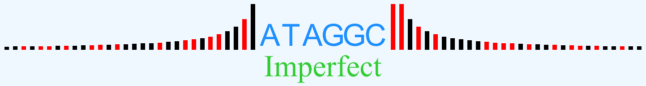</td><td>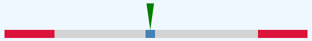</td><td></td><td>M</td></tr>
<tr><td>Imperfect match<br>115 136<br>5'-ctggAGCCAGGCCA-CACAGTCt-3' UTR<br>|||||::||| ||||| |<br>3'-ugagUCGGUUUGGUAGUGUCCGu-5' miRNA<br>16 15 14 13 7 6 5 4 3 2<br>3'pairing Seed</td><td>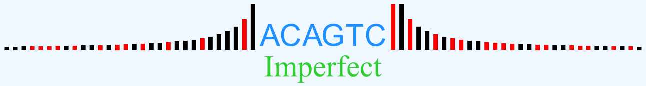</td><td>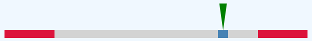</td><td></td><td>M</td></tr>
</table>

| 2D Structure | Local AU | Position | Conservation | Predicted By |
|---|---|---|---|---|
| 44 8mer 64<br>5'-actaccaCTGTGTCCTGTGCa-3' UTR<br>3'-cugacacGAGACAGGACACGu-5' miRNA<br>16 15 14 13 7 6 5 4 3 2<br>3'pairing Seed | 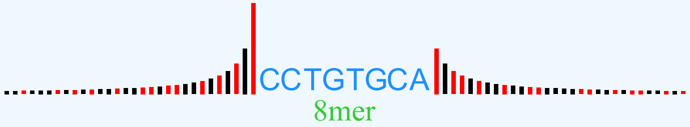 | 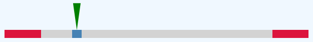 | | M T |

| 2D Structure | Local AU | Position | Conservation | Predicted By |
|---|---|---|---|---|
| Offset 6mer<br>234 … 259<br>5'-tggGCATCCTGTCAGCCAGCCTGTgc-3' UTR<br>3'-agaCGU-GGAGA---GGUCGGACAAu-5' miRNA<br>16 15 14 13 … 7 6 5 4 3 2<br>3'pairing … Seed | GCCTGT<br>Offset 6mer | | ☐ | M |
| 8mer<br>289 … 311<br>5'-tcacCACAGATGCCAGCCTGTTa-3' UTR<br>3'-agacGUGGAGA-GGUCGGACAAu-5' miRNA<br>16 15 14 13 … 7 6 5 4 3 2<br>3'pairing … Seed | GCCTGTTA<br>8mer | | ☐ | M T |
| 7mer-m8<br>611 … 634<br>5'-atcagACTTATCAACAGCCTGTTg-3' UTR<br>3'-agacgUGGAGAG--GUCGGACAAu-5' miRNA<br>16 15 14 13 … 7 6 5 4 3 2<br>3'pairing … Seed | GCCTGTT<br>7mer-m8 | | ☐ | M T |

| 2D Structure | Local AU | Position | Conservation | Predicted By |
|---|---|---|---|---|
| 206 7mer-m8 227<br>5'-cgagttcatacaGCAGCTGAAg-3' UTR<br>3'-ugucuuuccuccUGUCGACUUc-5' miRNA<br>16 15 14 13 7 6 5 4 3 2<br>3'pairing Seed | 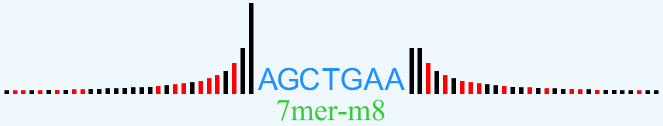 | 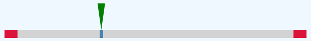 | | M T |
| 498 7mer-m8 519<br>5'-acAGACAAAGAGG-CAGCTGAAc-3' UTR<br>3'-ugUCU-UUCCUCCUGUCGACUUc-5' miRNA<br>16 15 14 13 7 6 5 4 3 2<br>3'pairing Seed | 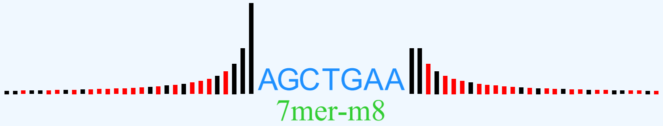 | 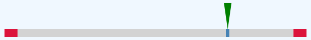 | | M T |

| 2D Structure | Local AU | Position | Conservation | Predicted By |
|---|---|---|---|---|
| Offset 6mer<br>86 107<br>5'-aagtaccctTAGGCAGGTGGGag-3' UTR<br>:\|\|\|\|\|\|\|\|\|\|\|<br>3'-gaccuccuGUCCGUCCACCCac-5' miRNA<br>16 15 14 13 7 6 5 4 3 2<br>3'pairing Seed | 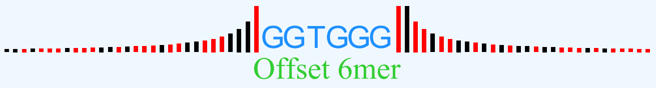 | 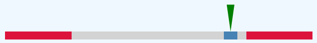 | | M |

| 2D Structure | Local AU | Position | Conservation | Predicted By |
|---|---|---|---|---|

**Site 1**

```
                          Imperfect
 208                        match       231
    5'-cgTTCTTCCACTGTGTTCTTTTCct -3'  UTR
        :||:|||||||:  :||||:|
    3'-uaGAGGAGGUGACG-GAGAAGAcu-5'  miRNA
              16 15 14 13   7 6 5 4 3 2
       3'pairing          Seed
```

Local AU: CTTTTC — Imperfect | Conservation: ☐ | Predicted By: M

**Site 2**

```
                            Offset
 412                         6mer      434
    5'-ctCTGCTTTGCTCAATCTTCTTt-3'  UTR
         ||  ||:::||    ||||||
    3'-uaGAGGAGGUGACGGAGAAGAcu-5'  miRNA
               16 15 14 13  7 6 5 4 3 2
       3'pairing          Seed
```

Local AU: TCTTCT — Offset 6mer | Conservation: ☐ | Predicted By: M

**Site 3**

```
 523                       7mer-m8   545
    5'-cagTCTTTGACCAGTTCTTCTGt-3'  UTR
          ||:|:  ||    :|||||||
    3'-uagAGGAGGUGACGGAGAAGACu-5'  miRNA
               16 15 14 13  7 6 5 4 3 2
       3'pairing          Seed
```

Local AU: TCTTCTG — 7mer-m8 | Conservation: ☐ | Predicted By: M T

**Site 4**

```
                             Offset
 3832                         6mer      3853
    5'-ttCTGCCTTCA--GCCTCTTCTag-3'  UTR
        ||  |||:||   |||||||||
    3'-uaGA-GGAGGUGACGGAGAAGAcu-5'  miRNA
                16 15 14 13  7 6 5 4 3 2
       3'pairing          Seed
```

Local AU: TCTTCT — Offset 6mer | Conservation: ☐ | Predicted By: M

**Site 5**

```
                         Imperfect
 4286                      match       4308
    5'-tgTTCCTCTGCCTCCTTTTCTaa -3'  UTR
         :|||||::|   |||:||||
    3'-uaGAGGAGGUGACGGAGAAGAcu-5'  miRNA
               16 15 14 13  7 6 5 4 3 2
       3'pairing          Seed
```

Local AU: TTTCTA — Imperfect | Conservation: ☐ | Predicted By: M

**Site 6**

```
                          Imperfect
 4544                       match       4565
    5'-gaCTCTTGTTAC--CTTTTTCTGg -3'  UTR
         |||:|  ::||  |:|:|||||
    3'-uaGAGGA-GGUGACGGAGAAGACu-5'  miRNA
                16 15 14 13  7 6 5 4 3 2
       3'pairing          Seed
```

Local AU: TTTCTG — Imperfect | Conservation: ☐ | Predicted By: M

**Site 7**

```
                            Imperfect
 4828                         match       4852
    5'-ccCTCTCTCTGTTCACCTCTTTTGa -3'  UTR
         |||  ||||:::|  ||||||:||
    3'-uaGAG-GAGGUGA-CGGAGAAGACu-5'  miRNA
               16 15 14 13    7 6 5 4 3 2
       3'pairing            Seed
```

Local AU: CTTTTG — Imperfect | Conservation: ☐ | Predicted By: M

**Site 8**

```
 8618                       7mer-m8   8641
    5'-ctgTTCTCCTGTTTGCTCTTCTGt-3'  UTR
          |:|||| ::|  ||||||||
    3'-uagAGGAGG-UGACGGAGAAGACu-5'  miRNA
              16 15   14 13  7 6 5 4 3 2
       3'pairing          Seed
```

Local AU: TCTTCTG — 7mer-m8 | Conservation: ☐ | Predicted By: M T

<table>
<tr><th>2D Structure</th><th>Local AU</th><th>Position</th><th>Conservation</th><th>Predicted By</th></tr>
<tr><td>Offset 6mer<br>23 46<br>5'-gaCTACTCCACAGATCTCTTCTaa-3' UTR<br>3'-uaGAGGAGGUGAC-GGAGAAGACu-5' miRNA<br>16 15 14 13 7 6 5 4 3 2<br>3'pairing Seed</td><td>TCTTCT<br>Offset 6mer</td><td></td><td></td><td>M</td></tr>
<tr><td>7mer-m8<br>335 355<br>5'-agagaCTCCAT--CATCTTCTGc-3' UTR<br>3'-uagagGAGGUGACGGAGAAGACu-5' miRNA<br>16 15 14 13 7 6 5 4 3 2<br>3'pairing Seed</td><td>TCTTCTG<br>7mer-m8</td><td></td><td></td><td>M T</td></tr>
<tr><td>Imperfect match<br>434 457<br>5'-ggCACCTTCACTGCTTTCCTCTGg -3' UTR<br>3'-uaGAGGAGGUGACG-GAGAAGACu-5' miRNA<br>16 15 14 13 7 6 5 4 3 2<br>3'pairing Seed</td><td>CCTCTG<br>Imperfect</td><td></td><td></td><td>M</td></tr>
<tr><td>7mer-m8<br>2385 2407<br>5'-acacagctggagggaTCTTCTGc-3' UTR<br>3'-uagaggaggugacggAGAAGACu-5' miRNA<br>16 15 14 13 7 6 5 4 3 2<br>3'pairing Seed</td><td>TCTTCTG<br>7mer-m8</td><td></td><td></td><td>M T</td></tr>
<tr><td>7mer-m8<br>2504 2530<br>5'-acCTTCTGACACTCCGCACTCTTCTGc-3' UTR<br>3'-uaGAGGA-GGUGA--CG-GAGAAGACu-5' miRNA<br>16 15 14 13 7 6 5 4 3 2<br>3'pairing Seed</td><td>TCTTCTG<br>7mer-m8</td><td></td><td></td><td>M T</td></tr>
</table>

| 2D Structure | Local AU | Position | Conservation | Predicted By |
| --- | --- | --- | --- | --- |
| Imperfect match<br>75 95<br>5'-aaGCTGGGGGCAAGGAAGAGc -3' UTR<br>3'-gaCGUCCCUCUCCCCUUUUCu-5' miRNA<br>16 15 14 13 7 6 5 4 3 2<br>3'pairing Seed | 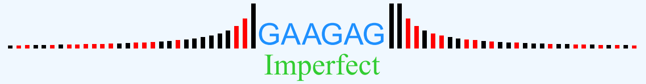 | 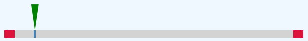 | | M |
| 7mer-m8<br>240 260<br>5'-tggacgccgatctGGAAAAGg-3' UTR<br>3'-gacgucccucuccCCUUUUCu-5' miRNA<br>16 15 14 13 7 6 5 4 3 2<br>3'pairing Seed | 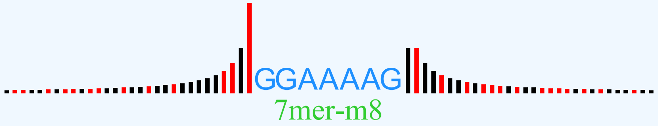 | 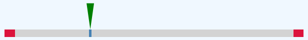 | | M T |
| 7mer-m8<br>588 608<br>5'-ctggctccAAAAAGGAAAAGg-3' UTR<br>3'-gacgucccUCUCCCCUUUUCu-5' miRNA<br>16 15 14 13 7 6 5 4 3 2<br>3'pairing Seed | 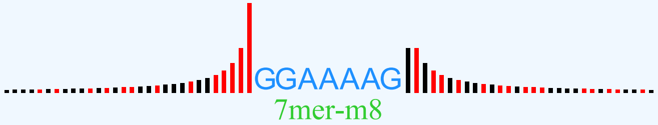 | 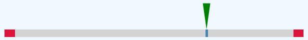 | | M T |
| 7mer-m8<br>648 671<br>5'-gcGTGGGGACTTGTTCGGAAAAGg-3' UTR<br>3'-gaCGUCCCU---CUCCCCUUUUCu-5' miRNA<br>16 15 14 13 7 6 5 4 3 2<br>3'pairing Seed | 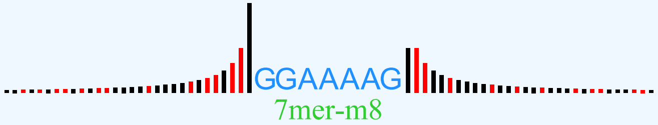 | 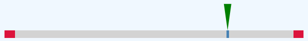 | | M T |

<table>
<tr><th>2D Structure</th><th>Local AU</th><th>Position</th><th>Conservation</th><th>Predicted By</th></tr>
<tr><td>91 7mer-m8 116<br>5'-agCTCAGGCTTCTATGAAGACCCCAg-3' UTR<br>||| || |:|: | |||||||<br>3'-ucGAGACC-AGGGGA---CUGGGGGUu-5' miRNA<br>16 15 14 13 7 6 5 4 3 2<br>3'pairing Seed</td><td>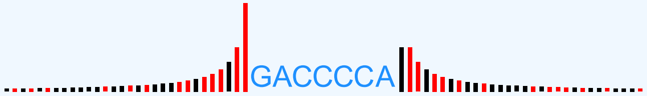</td><td>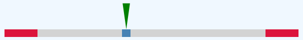</td><td>☐</td><td>M T</td></tr>
<tr><td>Offset 6mer<br>192 215<br>5'-acgcCTGGGTCCCTCGGACCCCCg-3' UTR<br>|||| :|||| ||||||<br>3'-ucgaGACCAGGGGA--CUGGGGuu-5' miRNA<br>16 15 14 13 7 6 5 4 3 2<br>3'pairing Seed</td><td>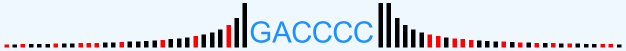</td><td>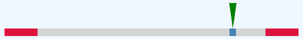</td><td>☐</td><td>M</td></tr>
</table>

<table>
<tr><th>2D Structure</th><th>Local AU</th><th>Position</th><th>Conservation</th><th>Predicted By</th></tr>
<tr><td><pre>77                 8mer      99
  5'-aaAGTGCATGAAGATGAAGCAGa-3'  UTR
       || |  |  || |  |||||||
  3'-gaUCUCCUCCUCC--CUUCGUCu-5'  miRNA
         16 15 14 13   7 6 5 4 3 2
        3'pairing      Seed</pre></td><td>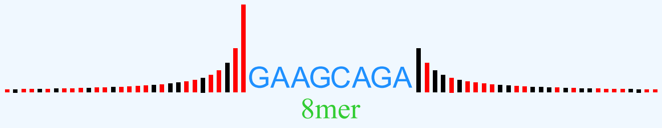
</td><td>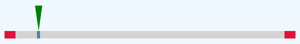</td><td></td><td>M T</td></tr>
<tr><td><pre>564                7mer-m8  589
  5'-ctGGAGCAGGGCCTTGTGGAAGCAGc-3'  UTR
       :||| |||:    | ||||||||
  3'-gaUCUCCUCCU----C-CCUUCGUCu-5'  miRNA
        16 15 14 13      7 6 5 4 3 2
        3'pairing         Seed</pre></td><td>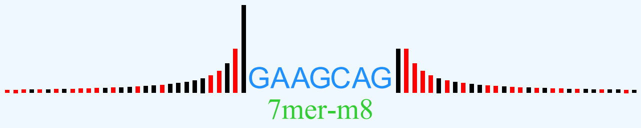
</td><td>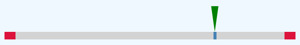</td><td></td><td>M T</td></tr>
</table>

| 2D Structure | | | | | Local AU | Position | Conservation | Predicted By |
|---|---|---|---|---|---|---|---|---|
| 7mer-m8 | 657 | 5'-ctTCCCT-CCTGTCCCTCTCCTt-3' UTR | 3'-ucAGGGACGGUUGAGGAGAGGAa-5' miRNA | 678 | 7mer-m8 | | | M T |
| Imperfect match | 689 | 5'-tccCCCAGCCCCAGCCTTCTCTTCTt-3' UTR | 3'-ucaGGGAC--GGUU-GAGGAGAGGAa-5' miRNA | 714 | Imperfect | | | M |
| Imperfect match | 724 | 5'-tgcagaTGTCCAACT-CTTTCCTc-3' UTR | 3'-ucagggAC-GGUUGAGGAGAGGAa-5' miRNA | 746 | Imperfect | | | M |
| Offset 6mer | 6691 | 5'-atagCCACCCACCCCCTCTCCCc-3' UTR | 3'-ucagGGACGGUUGAGGAGAGGaa-5' miRNA | 6713 | Offset 6mer | | | M |
| Imperfect match | 7201 | 5'-gcTCTTTCCTACTTTCTCTTCTa-3' UTR | 3'-ucAGGGACGGUUGAGGAGAGGAa-5' miRNA | 7223 | Imperfect | | | M |
| 7mer-m8 | 11420 | 5'-actgtatttttcTTTCTCTCCTt-3' UTR | 3'-ucagggacgguuGAGGAGAGGAa-5' miRNA | 11442 | 7mer-m8 | | | M T |
| Imperfect match | 11506 | 5'-tagggCTCCCTAAGCCTTTCCTg-3' UTR | 3'-ucaggGACGGUUGAGGAGAGGAa-5' miRNA | 11528 | Imperfect | | | M |
| 6mer | 15767 | 5'-ctgCCTTAAATAGTCTTCTCCTc-3' UTR | 3'-ucaGGGACGGUUGAGGAGAGGAa-5' miRNA | 15789 | 6mer | | | M |
| Imperfect match | 18867 | 5'-ctggCTTGCTCAGCCTGCTCTCTTa-3' UTR | 3'-ucagGGACG-GUU-GAGGAGAGGAa-5' miRNA | 18891 | Imperfect | | | M |
| Imperfect match | 21326 | 5'-ctggCTTGCATAGCCTGCTCTCTTa-3' UTR | 3'-ucagGGACG-GUU-GAGGAGAGGAa-5' miRNA | 21350 | Imperfect | | | M |
| 6mer | 22420 | 5'-ggcTTTTCCCCACT-TTCTCCTc-3' UTR | 3'-ucaGGGACGGUUGAGGAGAGGAa-5' miRNA | 22441 | 6mer | | | M |
| Imperfect match | 23509 | 5'-atTTCCTTTCATTCCCCTCTTCTt-3' UTR | 3'-ucAGGGACGGU-UGAGGAGAGGAa-5' miRNA | 23532 | Imperfect | | | M |
| Imperfect match | 24832 | 5'-aaTCCCTCATCATATCTTTCCTt-3' UTR | 3'-ucAGGGACGGUUGAGGAGAGGAa-5' miRNA | 24854 | Imperfect | | | M |
| Imperfect match | 28441 | 5'-cagTTTTGCCATCCCCACTCCTc-3' UTR | 3'-ucaGGGACGGUUGAGGAGAGGAa-5' miRNA | 28463 | Imperfect | | | M |
| Imperfect match | 29101 | 5'-gtTCCCTGACTGTCTACCTCTTCTt-3' UTR | 3'-ucAGGGAC-GGUUGA-GGAGAGGAa-5' miRNA | 29125 | Imperfect | | | M |
| Imperfect match | 29155 | 5'-gaTCCTAGTCAAAACCTTTTCTt-3' UTR | 3'-ucAGGGACGGUUGAGGAGAGGAa-5' miRNA | 29177 | Imperfect | | | M |
| 8mer | 29171 | 5'-ttTTCTTGCCTCCTGTCTCTCCTa-3' UTR | 3'-ucAGGGACGGUUGA-GGAGAGGAa-5' miRNA | 29194 | 8mer | | | M T |
| 8mer | 29235 | 5'-ttTCATCT-TCTGTTTCTCTCCTa-3' UTR | 3'-ucAG-GGACGGUUGAGGAGAGGAa-5' miRNA | 29257 | 8mer | | | M T |
| Imperfect match | 29471 | 5'-gcTCCCTGCATAA--CCTCTCTTa-3' UTR | 3'-ucAGGGACG-GUUGAGGAGAGGAa-5' miRNA | 29492 | Imperfect | | | M |
| Imperfect match | 33729 | 5'-tcTTCCT-CTAACTGCTTTCTTg-3' UTR | 3'-ucAGGGACGGUUGAGGAGAGGAa-5' miRNA | 33750 | Imperfect | | | M |
| Imperfect match | 33859 | 5'-ccTCTTTTCCTTCTCTCTCTTCTc-3' UTR | 3'-ucAGGGACGGUUGAG-GAGAGGAa-5' miRNA | 33882 | Imperfect | | | M |
| Imperfect match | 35343 | 5'-tcTTCCTTCAAACTTCACTCCTc-3' UTR | 3'-ucAGGGACGGUUGAGGAGAGGAa-5' miRNA | 35365 | Imperfect | | | M |
| Imperfect match | 38633 | 5'-cccCCCCGCCCAGTCTTCTCCCa-3' UTR | 3'-ucaGGGACGGUUGAGGAGAGGaa-5' miRNA | 38655 | Imperfect | | | M |
| Offset 6mer | 38658 | 5'-accCCCTCCCATTGCCCCTCTCCCc-3' UTR | 3'-ucaGGGACGGU--UGAGGAGAGGaa-5' miRNA | 38682 | Offset 6mer | | | M |
| Offset 6mer | 39547 | 5'-taTCCTTCCCAATCCCCTTCCTCTCCAt-3' UTR | 3'-ucAGGGACGGUU-----GAGGAGAGGaa-5' miRNA | 39574 | Offset 6mer | | | M |
| Imperfect match | 41460 | 5'-gcTACTCCCCACTTTCTCTTCTa-3' UTR | 3'-ucAGGGACGGUUGAGGAGAGGAa-5' miRNA | 41482 | Imperfect | | | M |
| Imperfect match | 43876 | 5'-ctTTCCTTCC--TTTCTTTCCTt-3' UTR | 3'-ucAGGGACGGUUGAGGAGAGGAa-5' miRNA | 43896 | Imperfect | | | M |
| Imperfect match | 43889 | 5'-ctTTCCTTCT--TTTCTTTCCTt-3' UTR | 3'-ucAGGGACGGUUGAGGAGAGGAa-5' miRNA | 43909 | Imperfect | | | M |
| Imperfect match | 43902 | 5'-ctTTCCTTCC--TTTCTTTCCTt-3' UTR | 3'-ucAGGGACGGUUGAGGAGAGGAa-5' miRNA | 43922 | Imperfect | | | M |
| Imperfect match | 43952 | 5'-ccTTCCTTTCTTTTCCTTTCCTt-3' UTR | 3'-ucAGGGACGGUUGAGGAGAGGAa-5' miRNA | 43974 | Imperfect | | | M |
| Imperfect match | 43972 | 5'-ctTTCCTTTCTTTTCCTTTCCTt-3' UTR | 3'-ucAGGGACGGUUGAGGAGAGGAa-5' miRNA | 43994 | Imperfect | | | M |
| Imperfect match | 43991 | 5'-ccTTTTTCCTATCCTCTTTCCTt-3' UTR | 3'-ucAGGGACGGUUGAGGAGAGGAa-5' miRNA | 44013 | Imperfect | | | M |
| Imperfect match | 44012 | 5'-ttTCCTTTCTCTTTTCTTTCCTt-3' UTR | 3'-ucAGGGACGGUUGAGGAGAGGAa-5' miRNA | 44034 | Imperfect | | | M |
| Imperfect match | 44034 | 5'-ttTTCCTTTCCTTTCCTTTCCTt-3' UTR | 3'-ucAGGGACGGUUGAGGAGAGGAa-5' miRNA | 44056 | Imperfect | | | M |
| Imperfect match | 44060 | 5'-ttTCCTTTCCTTTTCCCTTTCCTt-3' UTR | 3'-ucAGGGACGGUUGA-GGAGAGGAa-5' miRNA | 44083 | Imperfect | | | M |
| Imperfect match | 44172 | 5'-ttTCTCTGTTTCTGACATACTTTCCTg-3' UTR | 3'-ucAGGGAC---GGUUG-AGGAGAGGAa-5' miRNA | 44198 | Imperfect | | | M |
| 6mer | 46765 | 5'-atTCCCTGGAGGTTGTTCTCCTt-3' UTR | 3'-ucAGGGACGGUUGAGGAGAGGAa-5' miRNA | 46787 | 6mer | | | M |
| Imperfect match | 48486 | 5'-atTCCTTGCTGATTGAGACTTTCCTg-3' UTR | 3'-ucAGGGACGGUUGA---GGAGAGGAa-5' miRNA | 48511 | Imperfect | | | M |
| 6mer | 48604 | 5'-atgTCCTGCCGCCTTCCCCTTCTCCTt-3' UTR | 3'-ucaGGGACGGUUGA----GGAGAGGAa-5' miRNA | 48630 | 6mer | | | M |
| Imperfect match | 50426 | 5'-ctTCCCTCCCGTC-CCTTTCCTt-3' UTR | 3'-ucAGGGACGGUUGAGGAGAGGAa-5' miRNA | 50447 | Imperfect | | | M |
| Imperfect match | 50485 | 5'-tgcagaTGTCCAACT-CTTTCCTc-3' UTR | 3'-ucagggAC-GGUUGAGGAGAGGAa-5' miRNA | 50507 | Imperfect | | | M |
| Imperfect match | 60209 | 5'-taTCCC--CCGACTTTTTTCCTa-3' UTR | 3'-ucAGGGACGGUUGAGGAGAGGAa-5' miRNA | 60229 | Imperfect | | | M |
| Imperfect match | 61658 | 5'-tcaaCCT-CTAATTTACTCTTCTg-3' UTR | 3'-ucagGGACGGUUGAG-GAGAGGAa-5' miRNA | 61680 | Imperfect | | | M |
| Imperfect match | 63447 | 5'-ttaTTCTTCAAACTTCACTCCTc-3' UTR | 3'-ucaGGGACGGUUGAGGAGAGGAa-5' miRNA | 63469 | Imperfect | | | M |
| 7mer-m8 | 65965 | 5'-gtTCAAAGTCA--TTCTCTCCTt-3' UTR | 3'-ucAGGGACGGUUGAGGAGAGGAa-5' miRNA | 65985 | 7mer-m8 | | | M T |
| Offset 6mer | 66930 | 5'-accCCCTCCCATTGCCCCTCTCCCc-3' UTR | 3'-ucaGGGACGGU--UGAGGAGAGGaa-5' miRNA | 66954 | Offset 6mer | | | M |
| Offset 6mer | 67820 | 5'-taTCCTTCCCAATCCCCTTCCTCTCCAt-3' UTR | 3'-ucAGGGACGGUU-----GAGGAGAGGaa-5' miRNA | 67847 | Offset 6mer | | | M |
| Imperfect match | 69298 | 5'-taTTCC--CCACTTTCTCTTCTc-3' UTR | 3'-ucAGGGACGGUUGAGGAGAGGAa-5' miRNA | 69318 | Imperfect | | | M |
| 6mer | 72665 | 5'-ggcTTTTCCCCACT-TTCTCCTc-3' UTR | 3'-ucaGGGACGGUUGAGGAGAGGAa-5' miRNA | 72686 | 6mer | | | M |
| 6mer | 74162 | 5'-atTGCCTGGAGGTTTTTCTCCTt-3' UTR | 3'-ucAGGGACGGUUGAGGAGAGGAa-5' miRNA | 74184 | 6mer | | | M |
| 6mer | 75036 | 5'-agcCCCATCCAGGT-TTCTCCTt-3' UTR | 3'-ucaGGGACGGUUGAGGAGAGGAa-5' miRNA | 75057 | 6mer | | | M |
| Imperfect match | 75946 | 5'-atTCCTTGCTGATTGAGTCTTTCCTg-3' UTR | 3'-ucAGGGACGGUUGA---GGAGAGGAa-5' miRNA | 75971 | Imperfect | | | M |
| 6mer | 76012 | 5'-ccTCCCATCTTCCCCTTCTCCTc-3' UTR | 3'-ucAGGGACGGUUGAGGAGAGGAa-5' miRNA | 76034 | 6mer | | | M |

| 2D Structure | Local AU | Position | Conservation | Predicted By |
|---|---|---|---|---|
| 56 7mer-m8 76<br>5'-cttTGATGAGAATGAAAGAGt-3' UTR<br>3'-gacACCAACCAUACUUUCUCc-5' miRNA<br>16 15 14 13 · 7 6 5 4 3 2<br>3'pairing · Seed | 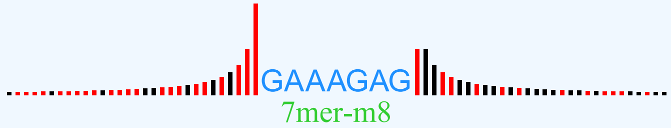<br> | 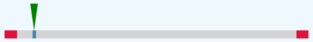 | | M T |
| 274 8mer 293<br>5'-ttaaGGCT-GCAGGAAAGAGa-3' UTR<br>3'-gacaCCAACCAUACUUUCUCc-5' miRNA<br>16 15 14 13 · 7 6 5 4 3 2<br>3'pairing · Seed | 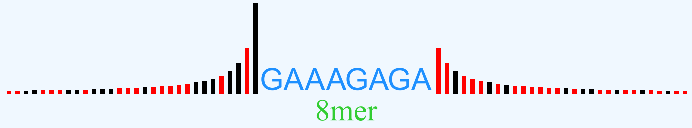<br> | 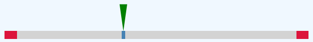 | | M T |

| 2D Structure | Local AU | Position | Conservation | Predicted By |
|---|---|---|---|---|
| Imperfect match<br>209 230<br>5'-ttGTATTTTGTGTAGAGAGAGt -3' UTR<br>|| || ||:| ||:||||<br>3'-gaCACCAACCAUA-CUUUCUCc-5' miRNA<br>16 15 14 13 7 6 5 4 3 2<br>3'pairing Seed | AGAGAG<br>Imperfect | | | M |
| 7mer-m8<br>246 266<br>5'-cttTGGTGTCTGGGAAAGAGg-3' UTR<br>|||| |: |||||||<br>3'-gacACCAACCAUACUUUCUCc-5' miRNA<br>16 15 14 13 7 6 5 4 3 2<br>3'pairing Seed | GAAAGAG<br>7mer-m8 | | | M T |

<table>
<tr><th>2D Structure</th><th>Local AU</th><th>Position</th><th>Conservation</th><th>Predicted By</th></tr>
<tr><td>Imperfect match<br>257 276<br>5'-tgttaCTCGCT-CCCTCCTTt-3' UTR<br>|||||| ||||||:|<br>3'-gaugaGAGCGAGGGGAGGGAc-5' miRNA<br>16 15 14 13 7 6 5 4 3 2<br>3'pairing Seed</td><td>CTCCTT<br>Imperfect</td><td></td><td></td><td>M</td></tr>
<tr><td>Imperfect match<br>456 476<br>5'-acgtgTTTGCTCCCCTTTCTc-3' UTR<br>:|:|||||||||::||<br>3'-gaugaGAGCGAGGGGAGGGAc-5' miRNA<br>16 15 14 13 7 6 5 4 3 2<br>3'pairing Seed</td><td>CTTTCT<br>Imperfect</td><td></td><td></td><td>M</td></tr>
<tr><td>Imperfect match<br>517 537<br>5'-caACGCTAGCCACCTTCCCTc-3' UTR<br>|| || || ||:|||||<br>3'-gaUGAGAGCGAGGGGAGGGAc-5' miRNA<br>16 15 14 13 7 6 5 4 3 2<br>3'pairing Seed</td><td>TTCCCT<br>Imperfect</td><td></td><td></td><td>M</td></tr>
</table>

| 2D Structure | Local AU | Position | Conservation | Predicted By |
| --- | --- | --- | --- | --- |
| 307 7mer-m8 329<br>5'-tggtggatggtaaagTCCCAGAt-3' UTR<br>|||||||<br>3'-ugaggacugucggggAGGGUCUa-5' miRNA<br>16 15 14 13 7 6 5 4 3 2<br>3'pairing Seed | TCCCAGA<br>7mer-m8 | | | M T |
| Imperfect match<br>331 353<br>5'-cacTCAGGAAGTGTTTCTCAGAg-3' UTR<br>:| |: ||: ::||:||||<br>3'-ugaGGACUGUCGGGGAGGGUCUa-5' miRNA<br>16 15 14 13 7 6 5 4 3 2<br>3'pairing Seed | CTCAGA<br>Imperfect | | | M |

<table>
<tr><th>2D Structure</th><th>Local AU</th><th>Position</th><th>Conservation</th><th>Predicted By</th></tr>
<tr><td>54 7mer-m8 75<br>5'-caGTCTAGGCTGA-CTCCCCCAg-3' UTR<br>|:|:|: |: | |||||||<br>3'-ugCGGGUUAGGGUCGAGGGGGUa-5' miRNA<br>16 15 14 13 7 6 5 4 3 2<br>3'pairing Seed</td><td>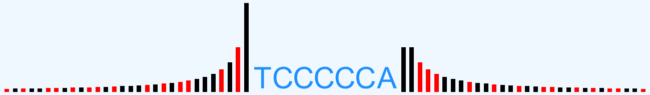</td><td>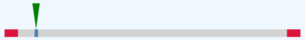</td><td>☐</td><td>M T</td></tr>
<tr><td>Imperfect match<br>71 93<br>5'-cccagCAATGCCACCTCCTCCAa -3' UTR<br>|||| ||| ||||:|||<br>3'-ugcggGUUAGGGUCGAGGGGGUa-5' miRNA<br>16 15 14 13 7 6 5 4 3 2<br>3'pairing Seed</td><td>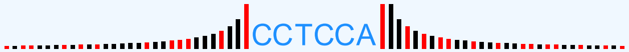</td><td>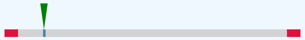</td><td>☐</td><td>M</td></tr>
<tr><td>Imperfect match<br>166 189<br>5'-cgGCCTTGAATGGC-GCTCCCTCAc -3' UTR<br>|||: ||| | |||||||:||<br>3'-ugCGGG--UUAGGGUCGAGGGGGUa-5' miRNA<br>16 15 14 13 7 6 5 4 3 2<br>3'pairing Seed</td><td>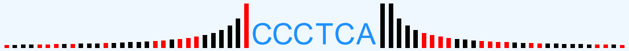</td><td>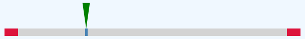</td><td>☐</td><td>M</td></tr>
<tr><td>Imperfect match<br>237 261<br>5'-ctGGCTAATCAGCAGCTGCCCCCAg-3' UTR<br>| |:|||| ||||| ||||||<br>3'-ugCGGGUUAG-GGUCGA-GGGGGUa-5' miRNA<br>16 15 14 13 7 6 5 4 3 2<br>3'pairing Seed</td><td>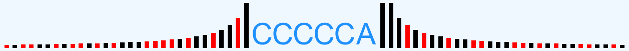</td><td>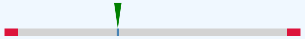</td><td>☐</td><td>M</td></tr>
</table>

| 2D Structure | Local AU | Position | Conservation | Predicted By |
|---|---|---|---|---|
| 161 8mer 186<br>5'-atGA-CCGCCGATTCTCCCAGCTCACa-3' UTR<br>\|\| \|\|:\|\|: :\| \| \|\|\|\|\|\|\|\|<br>3'-ccCUCGGUGGUCGA-A--GUCGAGUGc-5' miRNA<br>16 15 14 13 7 6 5 4 3 2<br>3'pairing Seed | 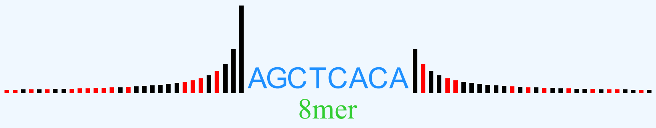 | 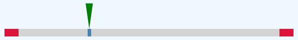 | ☐ | M T |
| Imperfect match<br>229 253<br>5'-gttACTCAGATGCTTACAGCTTACc-3' UTR<br>\| :\|\| \|\|\|\| \|\|\|\|\|:\|\|<br>3'-cccUCGGUGGUCGAA-GUCGAGUGc-5' miRNA<br>16 15 14 13 7 6 5 4 3 2<br>3'pairing Seed | 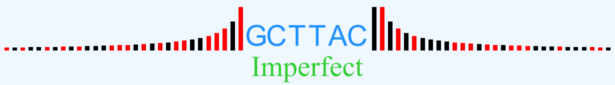 | 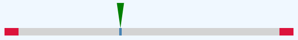 | ☐ | M |
| Imperfect match<br>328 352<br>5'-aaGGGAAACCATCTTCTAGTTCACg-3' UTR<br>\|:\| \|\|\|\| \|\|\|\| \|\|:\|\|\|\|<br>3'-ccCUCGGUGGUCGAAG-UCGAGUGc-5' miRNA<br>16 15 14 13 7 6 5 4 3 2<br>3'pairing Seed | 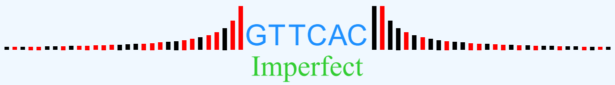 | 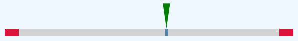 | ☐ | M |

| 2D Structure | Local AU | Position | Conservation | Predicted By |
|---|---|---|---|---|
| Imperfect match<br>1825 1846<br>5'-tcAAACT-AGACCCGAGAGCACt-3' UTR<br>3'-gaUUCGGUUCUGGUCUUUCGUGu-5' miRNA<br>3' pairing Seed | GAGCAC<br>Imperfect | | | M |
| 8mer<br>3567 3591<br>5'-tgtGGTAAATTATCAAAAAAGCACa-3' UTR<br>3'-gauUCGGUU-CUGG-UCUUUCGUGu-5' miRNA<br>3' pairing Seed | AAAGCACA<br>8mer | | | M T |
| Offset 6mer<br>3796 3818<br>5'-gccAGCCTGGTATACAAAGCAAg-3' UTR<br>3'-gauUCGGUUCUGGUCUUUCGUGu-5' miRNA<br>3' pairing Seed | AAAGCA<br>Offset 6mer | | | M |
| 6mer<br>4214 4240<br>5'-caGGGCCATGTGCCAGAGAGAAGCACc-3' UTR<br>3'-gaUUCGGUUC-UGGUC---UUUCGUGu-5' miRNA<br>3' pairing Seed | AAGCAC<br>6mer | | | M |
| Imperfect match<br>4892 4916<br>5'-ttAAGACATTGTACCTGAGAGCACa-3' UTR<br>3'-gaUUCGGU--UCUGGUCUUUCGUGu-5' miRNA<br>3' pairing Seed | GAGCAC<br>Imperfect | | | M |
| Imperfect match<br>5671 5693<br>5'-gacGGCCGAGCTCTGGAAGCGCa-3' UTR<br>3'-gauUCGGUUCUGGUCUUUCGUGu-5' miRNA<br>3' pairing Seed | AAGCGC<br>Imperfect | | | M |
| Offset 6mer<br>9302 9327<br>5'-agcAGCTTAGAACATCTGAAAGCAac-3' UTR<br>3'-gauUCGGUUCUGGU---CUUUCGUgu-5' miRNA<br>3' pairing Seed | AAAGCA<br>Offset 6mer | | | M |
| Offset 6mer<br>9340 9360<br>5'-cttGGCTTAGATC--AAAGCATa-3' UTR<br>3'-gauUCGGUUCUGGUCUUUCGUGu-5' miRNA<br>3' pairing Seed | AAAGCA<br>Offset 6mer | | | M |
| 7mer-m8<br>9842 9865<br>5'-aaAAGCCAAGCATTACAAAGCACt-3' UTR<br>3'-gaUUCGGUUC-UGGUCUUUCGUGu-5' miRNA<br>3' pairing Seed | AAAGCAC<br>7mer-m8 | | | M T |
| Imperfect match<br>11767 11790<br>5'-tgAAGTCTGAGATGGCAAAGTACt-3' UTR<br>3'-gaUUC-GGUUCUGGUCUUUCGUGu-5' miRNA<br>3' pairing Seed | AAGTAC<br>Imperfect | | | M |
| Offset 6mer<br>15988 16011<br>5'-accAGCAAAGACGAGCAAAGCATt-3' UTR<br>3'-gauUCGGUUCUGGUC-UUUCGUGu-5' miRNA<br>3' pairing Seed | AAAGCA<br>Offset 6mer | | | M |
| 7mer-m8<br>17948 17969<br>5'-tgGAGTATACA-TAAAAAGCACt-3' UTR<br>3'-gaUUCGGUUCUGGUCUUUCGUGu-5' miRNA<br>3' pairing Seed | AAAGCAC<br>7mer-m8 | | | M T |
| 7mer-m8<br>20176 20198<br>5'-gttAGTTTTGTTTTTAAAGCACg-3' UTR<br>3'-gauUCGGUUCUGGUCUUUCGUGu-5' miRNA<br>3' pairing Seed | AAAGCAC<br>7mer-m8 | | | M T |
| Imperfect match<br>25520 25541<br>5'-aacACCCATGACT-GAAAGTACg-3' UTR<br>3'-gauUCGGUUCUGGUCUUUCGUGu-5' miRNA<br>3' pairing Seed | AAGTAC<br>Imperfect | | | M |
| Imperfect match<br>25668 25690<br>5'-cctAGGCGACATTAGAAAGGACt-3' UTR<br>3'-gauUCGGUUCUGGUCUUUCGUGu-5' miRNA<br>3' pairing Seed | AAGGAC<br>Imperfect | | | M |
| Offset 6mer<br>27220 27243<br>5'-attGGCACTAGATGGGAAAAGCAgt-3' UTR<br>3'-gauUCG-GUUCUGGUCUUUCGUGu-5' miRNA<br>3' pairing Seed | AAAGCA<br>Offset 6mer | | | M |
| 8mer<br>29307 29327<br>5'-acAAGCAATAGCC--AAAGCACa-3' UTR<br>3'-gaUUCGGUUCUGGUCUUUCGUGu-5' miRNA<br>3' pairing Seed | AAAGCACA<br>8mer | | | M T |

| 2D Structure | Local AU | Position | Conservation | Predicted By |
|---|---|---|---|---|
| 51 7mer-m8 70<br>5'-ctcctgTCCA-GTTCTCAGAc-3' UTR<br>:\|\|\| \| \|\|\|\|\|\|\|<br>3'-uauaugGGGUACCAGAGUCUu-5' miRNA<br>16 15 14 13 7 6 5 4 3 2<br>3'pairing Seed | 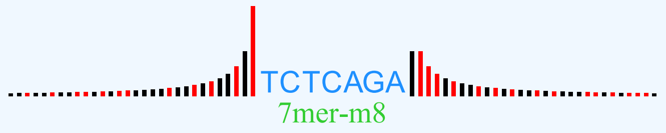 | 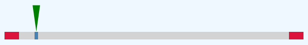 | | M T |
| 396 8mer 418<br>5'-agtgGCCACATACTTTCTCAGAa-3' UTR<br>:\|\| \|\|\| \|\|\|\|\|\|\|<br>3'-uauaUGGGGUA--CCAGAGUCUu-5' miRNA<br>16 15 14 13 7 6 5 4 3 2<br>3'pairing Seed | 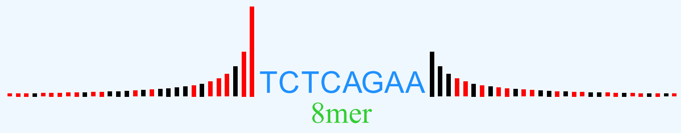 | 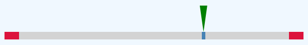 | | M T |

| 2D Structure | Local AU | Position | Conservation | Predicted By |
|---|---|---|---|---|
| Imperfect match<br>29 51<br>5'-gccTTGGGCCCTGTGGAGTCACt -3' UTR<br>:\|\|\|:\| \| :::\|\|\|:\|\|\|<br>3'-cuaGACCUGUG-UGUCUCGGUGu-5' miRNA<br>16 15 14 13 7 6 5 4 3 2<br>3'pairing Seed | 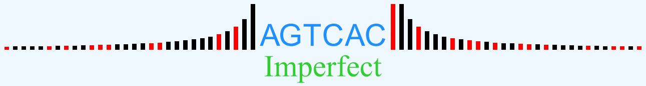 | 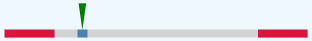 | | M |
| Offset 6mer<br>104 125<br>5'-ttaCTGGTCATCTGGAGCCAgg-3' UTR<br>\|\|\|\| \|\|: ::\|\|\|\|\|\|<br>3'-cuaGACCUGUGUGUCUCGGUGu-5' miRNA<br>16 15 14 13 7 6 5 4 3 2<br>3'pairing Seed | 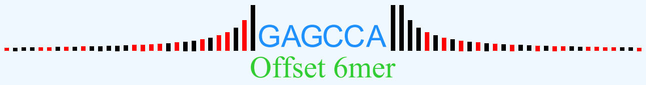 | 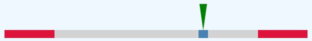 | | M |

| 2D Structure | Local AU | Position | Conservation | Predicted By |
|---|---|---|---|---|
| Imperfect match<br>70 … 91<br>5'-ccCCAGCAATGCCACCTCCTCc -3' UTR<br>3'-cuGAUCGUGUCGGUGGAGGGGu-5' miRNA<br>16 15 14 13 … 7 6 5 4 3 2<br>3'pairing Seed | 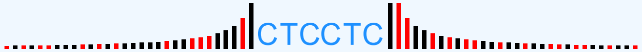 | 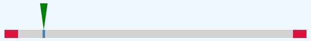 | ☐ | M |
| 7mer-m8<br>569 … 590<br>5'-ggaTGCCAGCA-CCACCTCCCCt-3' UTR<br>3'-cugAUCGU-GUCGGUGGAGGGGu-5' miRNA<br>16 15 14 13 … 7 6 5 4 3 2<br>3'pairing Seed | 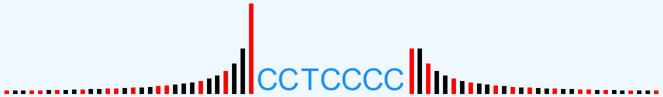 | 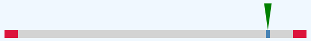 | ☐ | M T |

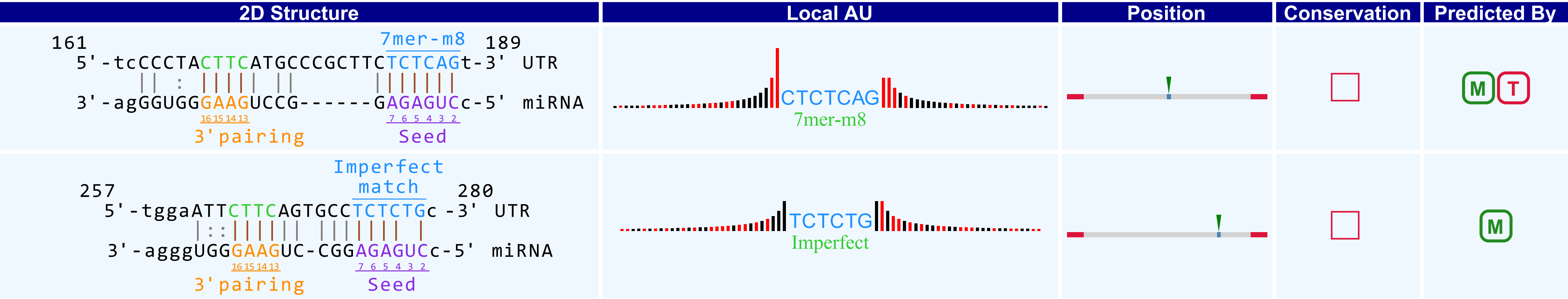
2D Structure
Local AU
Position
Conservation
Predicted By
7mer-m8
161
189
5'-tcCCCTACTTCATGCCCGCTTCTCTCAGt-3' UTR
3'-agGGUGGGAAGUCCG------GAGAGUCc-5' miRNA
16 15 14 13
7 6 5 4 3 2
3'pairing
Seed
CTCTCAG
7mer-m8
M
T
Imperfect match
257
280
5'-tggaATTCTTCAGTGCCTCTCTGc -3' UTR
3'-agggUGGGAAGUC-CGGAGAGUCc-5' miRNA
16 15 14 13
7 6 5 4 3 2
3'pairing
Seed
TCTCTG
Imperfect
M

| 2D Structure | Local AU | Position | Conservation | Predicted By |
|---|---|---|---|---|
| Imperfect match<br>274 297<br>5'-caTGGTCTACATGTTCCTTGGAGt-3' UTR<br>\|\| :\|: \|\| ::\| \|:\|\|\|\|\|<br>3'-ggACGGGGAGUCUGA-GGACCUCu-5' miRNA<br>16 15 14 13 7 6 5 4 3 2<br>3'pairing Seed | TTGGAG<br>Imperfect | | | M |
| 7mer-m8<br>628 655<br>5'-tgTGTTCTTTGTGACAGCAGCCTGGAGc-3' UTR<br>\|\|::\|:\|:: \|\|\| \|\|\|\|\|\|\|<br>3'-ggACGGGGAGU-CUG----AGGACCUCu-5' miRNA<br>16 15 14 13 7 6 5 4 3 2<br>3'pairing Seed | CCTGGAG<br>7mer-m8 | | | M T |
| 7mer-m8<br>685 708<br>5'-ctTGTCTGTCAGCTCTCCTGGAGt-3' UTR<br>\|\|:\|: \|\|\|\| \|\|\|\|\|\|\|\|\|<br>3'-ggACGGGGAGUC-UGAGGACCUCu-5' miRNA<br>16 15 14 13 7 6 5 4 3 2<br>3'pairing Seed | CCTGGAG<br>7mer-m8 | | | M T |

| 2D Structure | Local AU | Position | Conservation | Predicted By |
|---|---|---|---|---|
| 76 7mer-m8 101<br>5'-gaaaTTCCTCAGAGTGTTCCTGGAGt-3' UTR<br>::||||||| :|||||||<br>3'-ggacGGGGAGUCU---GAGGACCUCu-5' miRNA<br>16 15 14 13 7 6 5 4 3 2<br>3'pairing Seed | 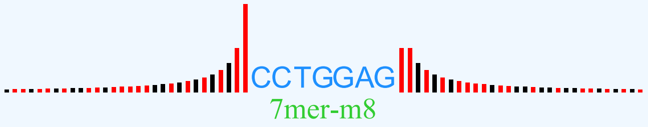 | 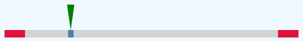 | | M T |

| 2D Structure | Local AU | Position | Conservation | Predicted By |
|---|---|---|---|---|
| 160 8mer 182<br>5'-aagggcctcccGGAGGAGGACGa-3' UTR<br>\|\|\|\| \|\|\|\|\|\|\|<br>3'-gacccuccucGCCUACUCCUGCc-5' miRNA<br>16 15 14 13 7 6 5 4 3 2<br>3'pairing Seed | 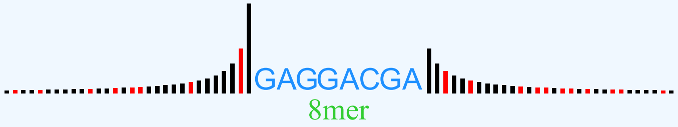 | 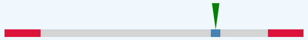 | | M T |

| 2D Structure | Local AU | Position | Conservation | Predicted By |
| --- | --- | --- | --- | --- |
| 202 7mer-m8 223<br>5'-tgaaAACAAGTCTGGAGCAGTg-3' UTR<br>3'-gacaUUCUUCCGUCCUCGUCAc-5' miRNA<br>16 15 14 13 7 6 5 4 3 2<br>3'pairing Seed | 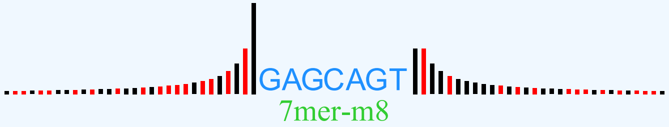  | 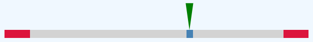 | | M T |

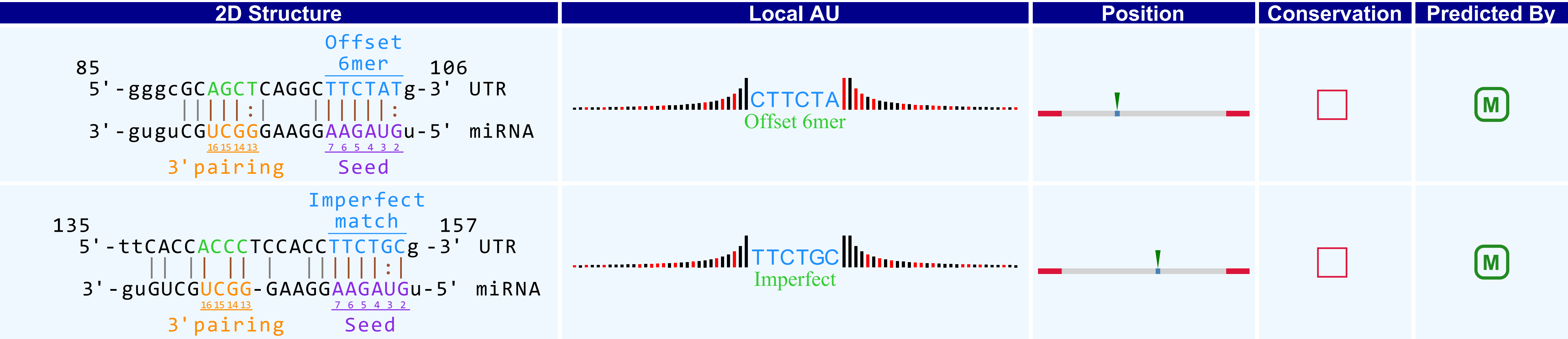
2D Structure
Local AU
Position
Conservation
Predicted By
Offset 6mer
85
106
5'-gggcGCAGCTCAGGCTTCTATg-3' UTR
3'-guguCGUCGGGAAGGAAGAUGu-5' miRNA
16 15 14 13
7 6 5 4 3 2
3'pairing
Seed
CTTCTA
Offset 6mer
M
Imperfect match
135
157
5'-ttCACCACCCTCCACCTTCTGCg-3' UTR
3'-guGUCGUCGG-GAAGGAAGAUGu-5' miRNA
16 15 14 13
7 6 5 4 3 2
3'pairing
Seed
TTCTGC
Imperfect
M

<table>
<tr><th>2D Structure</th><th>Local AU</th><th>Position</th><th>Conservation</th><th>Predicted By</th></tr>
<tr><td>Offset<br>6mer<br>124 146<br>5'-ggTGGTGGTGCTGTTCTTCTATg-3' UTR<br>::|::|: || |:||||||:<br>3'-guGUCGUCGGGA-AGGAAGAUGu-5' miRNA<br>16 15 14 13 7 6 5 4 3 2<br>3'pairing Seed</td><td>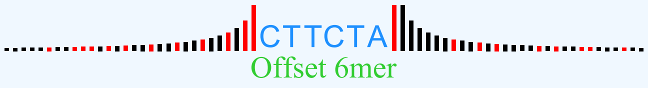</td><td>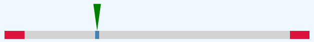</td><td></td><td>M</td></tr>
<tr><td>Offset<br>6mer<br>267 287<br>5'-aaCAGAAG-CATTTCTTCTATt-3' UTR<br>||| || | ||:||||||:<br>3'-guGUCGUCGGGAAGGAAGAUGu-5' miRNA<br>16 15 14 13 7 6 5 4 3 2<br>3'pairing Seed</td><td>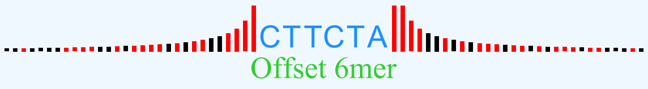</td><td>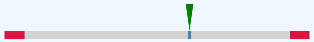</td><td></td><td>M</td></tr>
</table>

<table>
<tr><th>2D Structure</th><th>Local AU</th><th>Position</th><th>Conservation</th><th>Predicted By</th></tr>
<tr><td>352 7mer-m8 373<br>5'-aattctgatttataAGAACAGg-3' UTR<br>|||||||<br>3'-gaccuucccucccgUCUUGUCc-5' miRNA<br>16 15 14 13 7 6 5 4 3 2<br>3'pairing Seed</td><td>AGAACAG<br>7mer-m8</td><td></td><td></td><td>M T</td></tr>
<tr><td>Offset<br>524 6mer 545<br>5'-tgGAAAGCGTGTGTAGAACAtc-3' UTR<br>| ||| | | | :|||||<br>3'-gaCCUUCCCUCCCGUCUUGUcc-5' miRNA<br>16 15 14 13 7 6 5 4 3 2<br>3'pairing Seed</td><td>AGAACA<br>Offset 6mer</td><td></td><td></td><td>M</td></tr>
</table>

<table>
<tr><th>2D Structure</th><th>Local AU</th><th>Position</th><th>Conservation</th><th>Predicted By</th></tr>
<tr><td>Imperfect match<br>285 303<br>5'-acCACCACCACCACCATAg -3' UTR<br>| ||||||||||||:|<br>3'-cgGCGGUGGUGGUGGUGUg-5' miRNA<br>16 15 14 13 7 6 5 4 3 2<br>3'pairing Seed</td><td>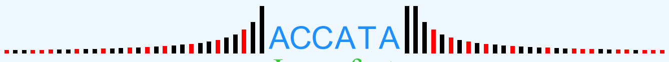<br></td><td>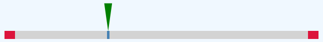</td><td>☐</td><td>M</td></tr>
<tr><td>Offset 6mer<br>300 318<br>5'-ataGCCACTGTCACCACcg-3' UTR<br>|||||:::|||||<br>3'-cggCGGUGGUGGUGGUGug-5' miRNA<br>16 15 14 13 7 6 5 4 3 2<br>3'pairing Seed</td><td>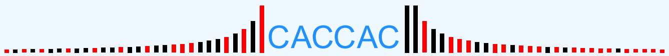<br></td><td>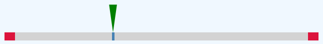</td><td>☐</td><td>M</td></tr>
</table>

| 2D Structure | Local AU | Position | Conservation | Predicted By |
|---|---|---|---|---|
| Offset 6mer<br>109 132<br>5'-ccgctgaCAGCCGTGCCTCCCTac-3' UTR<br>||||| | |||||||<br>3'-agucuggGUCGGGA-GGAGGGAgu-5' miRNA<br>16 15 14 13 7 6 5 4 3 2<br>3'pairing Seed | 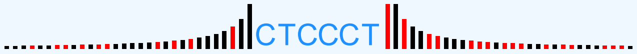<br>CTCCCT<br>Offset 6mer | 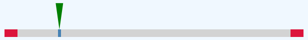 | | M |
| 7mer-m8<br>627 647<br>5'-gaAGGCCTA-CAC-ACTCCCTCg-3' UTR<br>||:||:| | | |||||||<br>3'-agUCUGGGUCGGGAGGAGGGAGu-5' miRNA<br>16 15 14 13 7 6 5 4 3 2<br>3'pairing Seed | 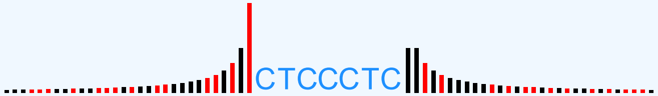<br>CTCCCTC<br>7mer-m8 | 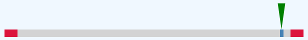 | | M T |

| 2D Structure | Local AU | Position | Conservation | Predicted By |
| --- | --- | --- | --- | --- |
| Offset 6mer<br>50 73<br>5'-ttGCCTACCTGGTCAGCCTTCAaa-3' UTR<br>3'-ggCGGAUGGACU-GACGGAAGUcu-5' miRNA<br>16 15 14 13 7 6 5 4 3 2<br>3'pairing Seed | CCTTCA<br>Offset 6mer | | | M |
| 7mer-m8<br>292 312<br>5'-aactcatCCT--CCGCCTTCAGg-3' UTR<br>3'-ggcggauGGACUGACGGAAGUCu-5' miRNA<br>16 15 14 13 7 6 5 4 3 2<br>3'pairing Seed | CCTTCAG<br>7mer-m8 | | | M T |

| 2D Structure | Local AU | Position | Conservation | Predicted By |
|---|---|---|---|---|
| 99 7mer-m8 119<br>5'-agtggatcttcaaGGCAGAGg-3' UTR<br>\|\|\|\|\|\|\|<br>3'-gacccuuccucccCCGUCUCg-5' miRNA<br>16 15 14 13 7 6 5 4 3 2<br>3'pairing Seed | 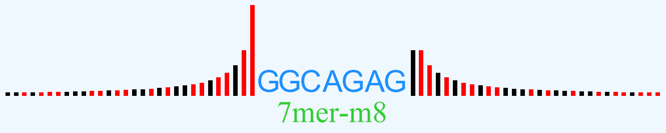 | 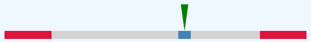 | | M T |
| 115 7mer-m8 137<br>5'-agaGGAAGTCATGTTGGCAGAGg-3' UTR<br>\|\|\|\|\| \| \| \|\|\|\|\|\|\|<br>3'-gacCCUUC-CU-CCCCCGUCUCg-5' miRNA<br>16 15 14 13 7 6 5 4 3 2<br>3'pairing Seed | 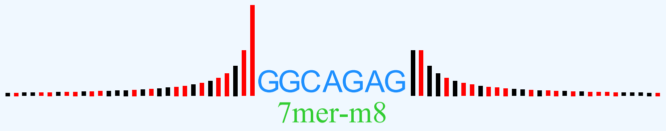 | 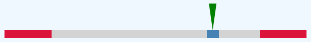 | | M T |

| 2D Structure | Local AU | Position | Conservation | Predicted By |
|---|---|---|---|---|
| 173 7mer-m8 197<br>5'-gtgACTGTAACAAGCGGCCTTCCTg-3' UTR<br>3'-ucaUGAGUCGGGUUGGUGGAAGGAu-5' miRNA<br>3' pairing Seed | CCTTCCT 7mer-m8 | | | M T |
| 1855 Offset 6mer 1879<br>5'-agTCCTTA-CTGAACCCTCCTTCCAc-3' UTR<br>3'-ucAUGAGUCGGGU-GGUGGAAGGau-5' miRNA<br>3' pairing Seed | CCTTCC Offset 6mer | | | M |
| 4321 Offset 6mer 4345<br>5'-ttTCTTTAGATGAGCTTCCTTCCCc-3' UTR<br>3'-ucAUGAGUCGGGUUGGUGGAAGGau-5' miRNA<br>3' pairing Seed | CCTTCC Offset 6mer | | | M |
| 4465 7mer-m8 4489<br>5'-gtaaaaattaaCATTGTCCTTCCTc-3' UTR<br>3'-ucaugagucggGUUGGUGGAAGGAu-5' miRNA<br>3' pairing Seed | CCTTCCT 7mer-m8 | | | M T |
| 10243 Imperfect match 10265<br>5'-gggcCTTGGTC--ACGGCCTTCTTg-3' UTR<br>3'-ucauGAGUCGGGUUGGUGGAAGGAu-5' miRNA<br>3' pairing Seed | CTTCTT Imperfect | | | M |
| 11525 7mer-m8 11547<br>5'-gagATCCTGCCT--CTACCTTCCTt-3' UTR<br>3'-ucaUGAGUCGGGUUGGUGGAAGGAu-5' miRNA<br>3' pairing Seed | CCTTCCT 7mer-m8 | | | M T |
| 12381 Imperfect match 12407<br>5'-acTACACAGTAAAGGACCACCTTCTTt-3' UTR<br>3'-ucAUGAGUCGGGU--UGGUGGAAGGAu-5' miRNA<br>3' pairing Seed | CTTCTT Imperfect | | | M |
| 13206 7mer-m8 13227<br>5'-gcccCTCAGCT---TTACCTTCCTg-3' UTR<br>3'-ucauGAGUCGGGUUGGUGGAAGGAu-5' miRNA<br>3' pairing Seed | CCTTCCT 7mer-m8 | | | M T |
| 14537 6mer 14561<br>5'-tgTGC-CAGACTCCACTCTCTTCCTc-3' UTR<br>3'-ucAUGAGUC-GGGUUGGUGGAAGGAu-5' miRNA<br>3' pairing Seed | CTTCCT 6mer | | | M |
| 15686 Imperfect match 15710<br>5'-ttaATTAGGGTCAACCTCCTTTCTa-3' UTR<br>3'-ucaUGAGUCGGGUUGGUGGAAGGAu-5' miRNA<br>3' pairing Seed | CTTTCT Imperfect | | | M |
| 16825 Imperfect match 16847<br>5'-aaTATTCAGGCT--CCACTTTCCTt-3' UTR<br>3'-ucAUGAGUCGGGUUGGUGGAAGGAu-5' miRNA<br>3' pairing Seed | TTTCCT Imperfect | | | M |
| 22407 Imperfect match 22435<br>5'-agTTCTTACCTTCAACGCTCTCCTTTCTc-3' UTR<br>3'-ucAUGAGUCG-GGUUG---GUGGAAGGAu-5' miRNA<br>3' pairing Seed | CTTTCT Imperfect | | | M |
| 25117 Offset 6mer 25140<br>5'-caaACTGCAAACCAA--ACCTTCCaa-3' UTR<br>3'-ucaUGA-GUCGGGUUGGUGGAAGGau-5' miRNA<br>3' pairing Seed | CCTTCC Offset 6mer | | | M |
| 26103 Imperfect match 26128<br>5'-tggGCTTAGCAGCCTTCACCTTCTTg-3' UTR<br>3'-ucaUGAGUCG-GGUUGGUGGAAGGAu-5' miRNA<br>3' pairing Seed | CTTCTT Imperfect | | | M |
| 26211 Imperfect match 26236<br>5'-gtgtggCAGCACCAGTCGCCTTCTTa-3' UTR<br>3'-ucaugaGUCG-GGUUGGUGGAAGGAu-5' miRNA<br>3' pairing Seed | CTTCTT Imperfect | | | M |
| 26265 Imperfect match 26290<br>5'-tggcCTTGGCCGCGCCTGCCTTCTTa-3' UTR<br>3'-ucauGAGUCGG-GUUGGUGGAAGGAu-5' miRNA<br>3' pairing Seed | CTTCTT Imperfect | | | M |
| 26301 Imperfect match 26326<br>5'-tagcCTCACCGGAAGCCGCCTTCTTg-3' UTR<br>3'-ucauGAGUCGGGU-GGUGGAAGGAu-5' miRNA<br>3' pairing Seed | CTTCTT Imperfect | | | M |
| 26439 Imperfect match 26464<br>5'-acccCGCGGCCGCCAGCGCCTTCTTg-3' UTR<br>3'-ucauGAGUCGG-GUUGGUGGAAGGAu-5' miRNA<br>3' pairing Seed | CTTCTT Imperfect | | | M |
| 29981 Offset 6mer 30002<br>5'-cggATACAG---AATCACCTTCCCg-3' UTR<br>3'-ucaUGAGUCGGGUUGGUGGAAGGau-5' miRNA<br>3' pairing Seed | CCTTCC Offset 6mer | | | M |

| 2D Structure | Local AU | Position | Conservation | Predicted By |
|---|---|---|---|---|
| 112 7mer-m8 135<br>5'-tttgaagtaACAAAATGGCCTCCt-3' UTR<br>3'-ucugugaggUGUUCGACCGGAGGu-5' miRNA<br>16 15 14 13 7 6 5 4 3 2<br>3'pairing Seed | 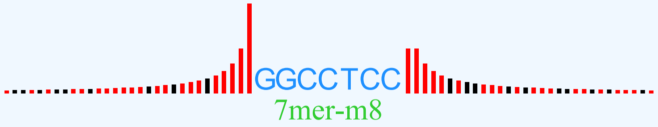 | 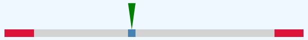 | 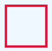 | M T |

| 2D Structure | Local AU | Position | Conservation | Predicted By |
|---|---|---|---|---|
| 126 7mer-m8 149<br>5'-aaaaCTTGGCAGGCTACCTCTCAg-3' UTR<br>3'-acacGAGUCGGACG-UGGAGAGUa-5' miRNA<br>16 15 14 13 7 6 5 4 3 2<br>3'pairing Seed | 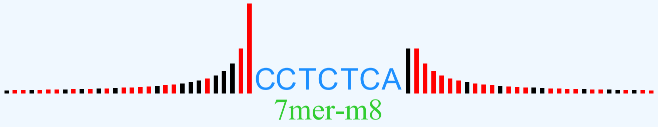 | 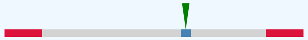 | | M T |

| 2D Structure | Local AU | Position | Conservation | Predicted By |
| --- | --- | --- | --- | --- |
| 152 8mer 173<br>5'-ggCTGCAGAAGGGC-CTCCCGGa-3' UTR<br>3'-gcGACGUCGUGGAGCGAGGGCCc-5' miRNA<br>16 15 14 13 7 6 5 4 3 2<br>3'pairing Seed | 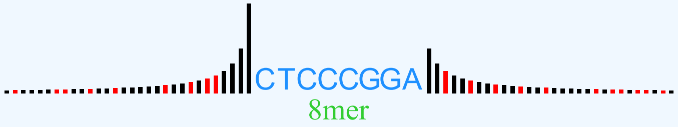 | 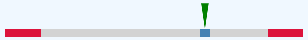 | | M T |

| 2D Structure | Local AU | Position | Conservation | Predicted By |
|---|---|---|---|---|
| Imperfect match<br>1107 1128<br>5'-taTTTGATTTTTTTTTCCCTt-3' UTR<br>:\|\|\| :\|:\|\| \|:\|:\|\|\|\|<br>3'-cgGAACGGAGAACAGAGGGGAg-5' miRNA<br>16 15 14 13 7 6 5 4 3 2<br>3'pairing Seed | TTCCCT<br>Imperfect | | | M |
| Imperfect match<br>1766 1789<br>5'-tgtgTGTTTTCATGTTCTCTCCTa-3' UTR<br>\|\| ::\|\| \|\| \|\|\|\|:\|\|\|<br>3'-cggaAC-GGAGAAC-AGAGGGGAg-5' miRNA<br>16 15 14 13 7 6 5 4 3 2<br>3'pairing Seed | TCTCCT<br>Imperfect | | | M |
| 7mer-m8<br>3755 3776<br>5'-ggagacaaTCTCTTCTCCCCTc-3' UTR<br>\|\|\| \|\|\|\|\|\|\|<br>3'-cggaacggAGAACAGAGGGGAg-5' miRNA<br>16 15 14 13 7 6 5 4 3 2<br>3'pairing Seed | CTCCCCT<br>7mer-m8 | | | M T |
| Offset 6mer<br>4490 4510<br>5'-tgCCTGCCTC-TGTCTCCCCag-3' UTR<br>\| \|\|\|\|\|\| \|\|\|\|\|\|\|\|<br>3'-cgGAACGGAGAACAGAGGGGag-5' miRNA<br>16 15 14 13 7 6 5 4 3 2<br>3'pairing Seed | CTCCCC<br>Offset 6mer | | | M |
| Imperfect match<br>8587 8608<br>5'-ctCTTTCCTATTCTCTCTCCTg-3' UTR<br>\|\|\| \|\|\| \|\| \|\|\|\|:\|\|\|<br>3'-cgGAACGGAGAACAGAGGGGAg-5' miRNA<br>16 15 14 13 7 6 5 4 3 2<br>3'pairing Seed | TCTCCT<br>Imperfect | | | M |
| 6mer<br>8627 8652<br>5'-tgTTTGCTCTTCTGTAGCTTCCCCTt-3' UTR<br>:\|\|\|\| \|\|: \|\|\| :\|\|\|\|\|<br>3'-cgGAACG-GAGAACA---GAGGGGAg-5' miRNA<br>16 15 14 13 7 6 5 4 3 2<br>3'pairing Seed | TCCCCT<br>6mer | | | M |

| 2D Structure | Local AU | Position | Conservation | Predicted By |
| --- | --- | --- | --- | --- |
| Offset 6mer<br>166 185<br>5'-aagaTCACTTCCCACCCAac-3' UTR<br>|| |:| ||||||<br>3'-ucgcAGAGGAUGGUGGGUgu-5' miRNA<br>16 15 14 13 7 6 5 4 3 2<br>3'pairing Seed | 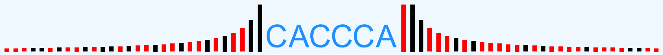 | 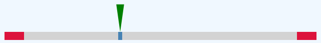 | ☐ | M |
| Imperfect match<br>223 242<br>5'-cagGTTACCTTCCACCTACt-3' UTR<br>||: ||| |||||:||<br>3'-ucgCAGAGGAUGGUGGGUGu-5' miRNA<br>16 15 14 13 7 6 5 4 3 2<br>3'pairing Seed | 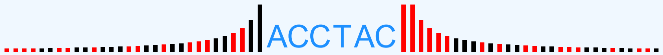 | 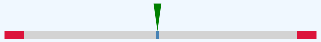 | ☐ | M |

<table>
<tr><th>2D Structure</th><th>Local AU</th><th>Position</th><th>Conservation</th><th>Predicted By</th></tr>
<tr><td>Imperfect match<br>83 105<br>5'-gtGGGCGCAGCTCAGGCTTCTAt -3' UTR<br>:::| || ||| :|||:|||<br>3'-ggUUUGGGUGGAGGUCGAGGAUg-5' miRNA<br>16 15 14 13 7 6 5 4 3 2<br>3'pairing Seed</td><td>CTTCTA<br>Imperfect</td><td></td><td></td><td>M</td></tr>
<tr><td>7mer-m8<br>167 189<br>5'-gcttcTCCGGATCTGGCTCCTAt-3' UTR<br>:||: ||::|||||||<br>3'-gguuuGGGUGGAGGUCGAGGAUg-5' miRNA<br>16 15 14 13 7 6 5 4 3 2<br>3'pairing Seed</td><td>GCTCCTA<br>7mer-m8</td><td></td><td></td><td>M T</td></tr>
</table>

| 2D Structure | Local AU | Position | Conservation | Predicted By |
|---|---|---|---|---|
| 240 7mer-m8 261<br>5'-ggattcTCCAACTGAGAGCAGt-3' UTR<br>3'-gaccccAGGU--CCUCUCGUCu-5' miRNA<br>16 15 14 13 7 6 5 4 3 2<br>3'pairing Seed | 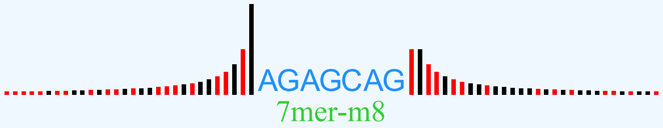 | 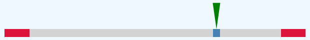 | | M T |
| 275 8mer 292<br>5'-gtcagacCCA--AGAGCAGa-3' UTR<br>3'-gaccccaGGUCCUCUCGUCu-5' miRNA<br>16 15 14 13 7 6 5 4 3 2<br>3'pairing Seed | 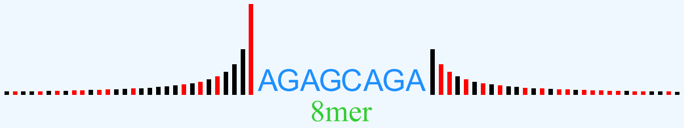 | 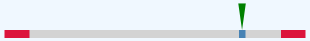 | | M T |

<table>
<tr><th>2D Structure</th><th>Local AU</th><th>Position</th><th>Conservation</th><th>Predicted By</th></tr>
<tr><td>97 7mer-m8 117<br>5'-gaggacGGCTTTACTCCTCTg-3' UTR<br>::|:||  |||||||<br>3'-acggguUUGGAAAGAGGAGAa-5' miRNA<br>16 15 14 13  7 6 5 4 3 2<br>3'pairing  Seed</td><td>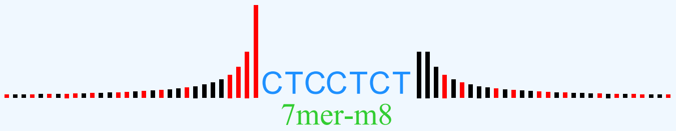</td><td>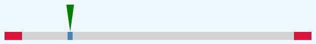</td><td>☐</td><td>M T</td></tr>
<tr><td>408 7mer-m8 426<br>5'-gaatgGAATC--ACTCCTCTg-3' UTR<br>:||:|  |||||||<br>3'-acgggUUUGGAAAGAGGAGAa-5' miRNA<br>16 15 14 13  7 6 5 4 3 2<br>3'pairing  Seed</td><td>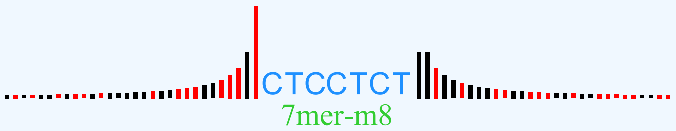</td><td>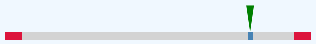</td><td>☐</td><td>M T</td></tr>
</table>

<table>
<tr><th>2D Structure</th><th>Local AU</th><th>Position</th><th>Conservation</th><th>Predicted By</th></tr>
<tr><td>475 7mer-m8 497<br>5'-aggcTGGTACACTGACTCTGAGg-3' UTR<br>||||   |::|||||||<br>3'-gaguACCACAGGGUUGAGACUCu-5' miRNA<br>16 15 14 13 7 6 5 4 3 2<br>3'pairing Seed</td><td>CTCTGAG<br>7mer-m8</td><td></td><td></td><td>M T</td></tr>
<tr><td>779 7mer-m8 801<br>5'-agataaagcTCTAGACTCTGAGg-3' UTR<br>||:  :||||||||<br>3'-gaguaccacAGGGUUGAGACUCu-5' miRNA<br>16 15 14 13 7 6 5 4 3 2<br>3'pairing Seed</td><td>CTCTGAG<br>7mer-m8</td><td></td><td></td><td>M T</td></tr>
<tr><td>Imperfect match<br>961 984<br>5'-aagATGGTGATGAGGACTCTGGGa -3' UTR<br>||||||| |   :||||||:|<br>3'-gagUACCAC-AGGGUUGAGACUCu-5' miRNA<br>16 15 14 13 7 6 5 4 3 2<br>3'pairing Seed</td><td>TCTGGG<br>Imperfect</td><td></td><td></td><td>M</td></tr>
</table>

| 2D Structure | Local AU | Position | Conservation | Predicted By |
| --- | --- | --- | --- | --- |
| 153 8mer 173<br>5'-aacatttaatgtaAAGCCACa-3' UTR<br>\|\|\|\|\|\|\|<br>3'-gaucucugcugucUUCGGUGa-5' miRNA<br>16 15 14 13 7 6 5 4 3 2<br>3'pairing Seed | 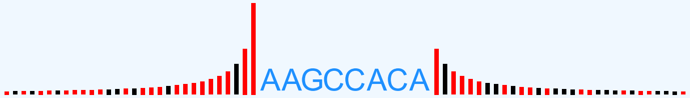 | 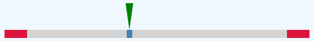 | | M T |
| Offset 6mer<br>185 205<br>5'-agtgccttTGCCAGAAGCCATg-3' UTR<br>:\| \|\|\|\|\|\|\|\|\|:<br>3'-gaucucuGCUGUCUUCGGUGa-5' miRNA<br>16 15 14 13 7 6 5 4 3 2<br>3'pairing Seed | 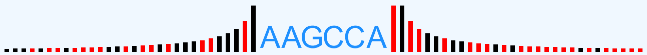 | 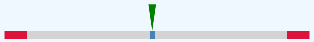 | | M |

| 2D Structure | Local AU | Position | Conservation | Predicted By |
|---|---|---|---|---|
| Imperfect match<br>681 703<br>5'-tcccttCAAATAAAA-AAGGAATc -3' UTR<br>\|\|\|\|:\|\|\|\| \|\|:\|\|\|:<br>3'-gauuccGUUUGUUUUGUUUCUUGu-5' miRNA<br>16 15 14 13 7 6 5 4 3 2<br>3'pairing Seed | 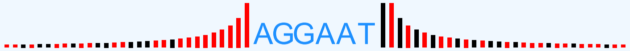<br>AGGAAT<br>Imperfect | 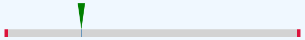 | | M |
| Imperfect match<br>1124 1149<br>5'-ggAGGGATCAGACAATCCAGAGAACc -3' UTR<br>\|:\|\| \|\|:\|\|\|\| \|\|:\|\|\|\|\|<br>3'-gaUUCC--GUUUGUUUUGUUUCUUGu-5' miRNA<br>16 15 14 13 7 6 5 4 3 2<br>3'pairing Seed | 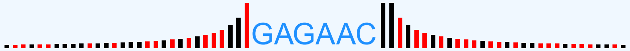<br>GAGAAC<br>Imperfect | 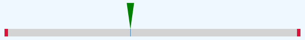 | | M |
| Imperfect match<br>1193 1216<br>5'-atcGGGGAACCAAGTTAAGGAACa -3' UTR<br>:\|\| \|\| \|\|\|: :\|\|:\|\|\|\|<br>3'-gauUCCGUUUGUUUUGUUUCUUGu-5' miRNA<br>16 15 14 13 7 6 5 4 3 2<br>3'pairing Seed | 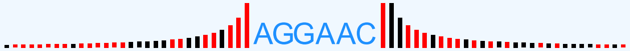<br>AGGAAC<br>Imperfect | 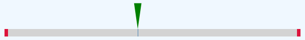 | | M |
| Offset 6mer<br>1741 1764<br>5'-agGAGGTTGATGTAGAAAAGAATa-3' UTR<br>:\|\|\|: :\|:: \|: \|\|\|\|\|\|:<br>3'-gaUUCCGUUUGUUUUGUUUCUUGu-5' miRNA<br>16 15 14 13 7 6 5 4 3 2<br>3'pairing Seed | 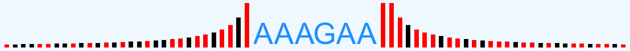<br>AAAGAA<br>Offset 6mer | 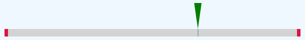 | | M |
| Offset 6mer<br>1979 2002<br>5'-gggtGATAAACCACACAAAGAAac-3' UTR<br>\| :\|\|\| \| \|\|\|\|\|\|\|<br>3'-gauuCCGUUUGUUUUGUUUCUUgu-5' miRNA<br>16 15 14 13 7 6 5 4 3 2<br>3'pairing Seed | 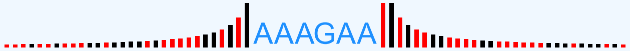<br>AAAGAA<br>Offset 6mer | 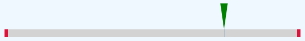 | | M |
| Offset 6mer<br>2321 2346<br>5'-agAAGCACAAAAAGAAAAAAGAAgc-3' UTR<br>\|\|\| \|\|\|\| \|\| \|\| \|\|\|\|\|\|<br>3'-gaUUC-CGUUUGUU-UUGUUUCUUgu-5' miRNA<br>16 15 14 13 7 6 5 4 3 2<br>3'pairing Seed | 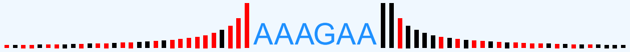<br>AAAGAA<br>Offset 6mer | 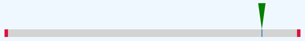 | | M |

| 2D Structure | | | | Local AU | Conservation | Predicted By |
|---|---|---|---|---|---|---|
| Site (start–end) | Site type | 5' UTR | 3' miRNA | | | |
| 2238–2261 | Offset 6mer | 5'-aaAAAGTTGACTATCCAAAGAAat-3' UTR | 3'-gaUUCCGUUUGUUUUGUUUCUUgu-5' miRNA (3' pairing; Seed) | AAAGAA Offset 6mer | ☐ | M |
| 2895–2916 | Offset 6mer | 5'-ggGAGG-AAAGAAAA-AAAGAAag-3' UTR | 3'-gaUUCCGUUUGUUUUGUUUCUUgu-5' miRNA (3' pairing; Seed) | AAAGAA Offset 6mer | ☐ | M |
| 2908–2932 | Offset 6mer | 5'-aaAAGAAAGAAAGAAAGAAAGAAag-3' UTR | 3'-gaUUCCGU-UUGUUUUGUUUCUUgu-5' miRNA (3' pairing; Seed) | AAAGAA Offset 6mer | ☐ | M |
| 2939–2962 | Offset 6mer | 5'-agAGAGAGAAAGAAAGAAAGAAag-3' UTR | 3'-gaUUCCGUUUGUUUUGUUUCUUgu-5' miRNA (3' pairing; Seed) | AAAGAA Offset 6mer | ☐ | M |
| 2954–2978 | Offset 6mer | 5'-gaAAGAAAGAAAGAAAGAAAGAAag-3' UTR | 3'-gaUUCCGU-UUGUUUUGUUUCUUgu-5' miRNA (3' pairing; Seed) | AAAGAA Offset 6mer | ☐ | M |
| 3007–3029 | Imperfect match | 5'-ctGAGG-GAAGAAAACAGAGAAga-3' UTR | 3'-gaUUCCGUUUGUUUUGUUUCUUgu-5' miRNA (3' pairing; Seed) | GAGAAG Imperfect | ☐ | M |
| 3127–3148 | Offset 6mer | 5'-ggGAGG-AAAGAAAA-AAAGAAag-3' UTR | 3'-gaUUCCGUUUGUUUUGUUUCUUgu-5' miRNA (3' pairing; Seed) | AAAGAA Offset 6mer | ☐ | M |
| 3140–3164 | Offset 6mer | 5'-aaAAGAAAGAAAGAAAGAAAGAAag-3' UTR | 3'-gaUUCCGU-UUGUUUUGUUUCUUgu-5' miRNA (3' pairing; Seed) | AAAGAA Offset 6mer | ☐ | M |
| 3177–3200 | Offset 6mer | 5'-agAGAGAGAAAGAAAGAAAGAAag-3' UTR | 3'-gaUUCCGUUUGUUUUGUUUCUUgu-5' miRNA (3' pairing; Seed) | AAAGAA Offset 6mer | ☐ | M |
| 3192–3216 | Offset 6mer | 5'-gaAAGAAAGAAAGAAAGAAAGAAag-3' UTR | 3'-gaUUCCGU-UUGUUUUGUUUCUUgu-5' miRNA (3' pairing; Seed) | AAAGAA Offset 6mer | ☐ | M |
| 3208–3232 | Offset 6mer | 5'-gaAAGAAAGAAAGAAAGAAAGAAag-3' UTR | 3'-gaUUCCGU-UUGUUUUGUUUCUUgu-5' miRNA (3' pairing; Seed) | AAAGAA Offset 6mer | ☐ | M |
| 3257–3279 | Imperfect match | 5'-ctGAGG-GAAGAAAACAGAGAAga-3' UTR | 3'-gaUUCCGUUUGUUUUGUUUCUUgu-5' miRNA (3' pairing; Seed) | GAGAAG Imperfect | ☐ | M |
| 3406–3429 | Offset 6mer | 5'-ttAAAAAAAAAAAAAAAAAGAAga-3' UTR | 3'-gaUUCCGUUUGUUUUGUUUCUUgu-5' miRNA (3' pairing; Seed) | AAAGAA Offset 6mer | ☐ | M |
| 3853–3876 | Imperfect match | 5'-ctgtGTCAGAAAAAACAAAGCACa-3' UTR | 3'-gauuCCGUUUGUUUUGUUUCUUGu-5' miRNA (3' pairing; Seed) | AAGCAC Imperfect | ☐ | M |
| 5998–6022 | Imperfect match | 5'-tacataTATATAAAGCGAAAGAGCc-3' UTR | 3'-gauuccGUUUGUUUUG-UUUCUUGu-5' miRNA (3' pairing; Seed) | AAGAGC Imperfect | ☐ | M |
| 8316–8339 | Imperfect match | 5'-agGGGGTATTCAAAGGAAGGAACc-3' UTR | 3'-gaUUCCGUUUGUUUUGUUUCUUGu-5' miRNA (3' pairing; Seed) | AGGAAC Imperfect | ☐ | M |
| 8386–8409 | Offset 6mer | 5'-accAGTTAAGAGTCACAAAGAAgt-3' UTR | 3'-gauUCCGUUUGUUUUGUUUCUUgu-5' miRNA (3' pairing; Seed) | AAAGAA Offset 6mer | ☐ | M |
| 9083–9107 | Imperfect match | 5'-ggtAGACTGTCCAAACAAAAGAGCa-3' UTR | 3'-gauUCCGUUUGUUUUG-UUUCUUGu-5' miRNA (3' pairing; Seed) | AAGAGC Imperfect | ☐ | M |
| 9842–9865 | Imperfect match | 5'-aaAAGCCAAGCATTACAAAGCACt-3' UTR | 3'-gaUUCCGUUUGUUUUGUUUCUUGu-5' miRNA (3' pairing; Seed) | AAGCAC Imperfect | ☐ | M |
| 12375–12398 | Imperfect match | 5'-cagtagactACACAGTAAAGGACc-3' UTR | 3'-gauuccguuUGUUUUGUUUCUUGu-5' miRNA (3' pairing; Seed) | AAGGAC Imperfect | ☐ | M |
| 14732–14758 | Imperfect match | 5'-caataGTAAAGACCTAAATAAAGAGCt-3' UTR | 3'-gauucCGUUU---GUUUUGUUUCUUGu-5' miRNA (3' pairing; Seed) | AAGAGC Imperfect | ☐ | M |
| 14874–14895 | Imperfect match | 5'-taAAGG-AAA-AAAAAAAAAAACc-3' UTR | 3'-gaUUCCGUUUGUUUUGUUUCUUGu-5' miRNA (3' pairing; Seed) | AAAAAC Imperfect | ☐ | M |
| 15083–15107 | Offset 6mer | 5'-tgtgtagAGACACACGCAAAGAAgt-3' UTR | 3'-gauuccgUUUGU-UUUGUUUCUUgu-5' miRNA (3' pairing; Seed) | AAAGAA Offset 6mer | ☐ | M |
| 16576–16602 | Imperfect match | 5'-agAAGGCCAAAGAGGAAATAAAGGACa-3' UTR | 3'-gaUUCC-GUUUGU--UUUGUUUCUUGu-5' miRNA (3' pairing; Seed) | AAGGAC Imperfect | ☐ | M |
| 18583–18607 | Imperfect match | 5'-gaaccGCAAGTCAACACAAAGGATc-3' UTR | 3'-gauucCGUUU-GUUUUGUUUCUUGu-5' miRNA (3' pairing; Seed) | AAGGAT Imperfect | ☐ | M |
| 18660–18684 | Imperfect match | 5'-ctgctcCAACACAAAACAAAGAGat-3' UTR | 3'-gauuccGUU-UGUUUUGUUUCUUgu-5' miRNA (3' pairing; Seed) | AAGAGA Imperfect | ☐ | M |
| 18768–18793 | Imperfect match | 5'-ccGAGGAAAATAATAAACAAGGAAag-3' UTR | 3'-gaUUCCGUUUGU--UUUGUUUCUUgu-5' miRNA (3' pairing; Seed) | AGGAAA Imperfect | ☐ | M |
| 22660–22685 | Offset 6mer | 5'-gaGATGTAGCACAGAGGCAAAGAAga-3' UTR | 3'-gaUUCCGUU-UGU-UUUGUUUCUUgu-5' miRNA (3' pairing; Seed) | AAAGAA Offset 6mer | ☐ | M |
| 24020–24043 | Imperfect match | 5'-taGAGGGAAACAAGAGAAAGAtga-3' UTR | 3'-gaUUCCGUUUGUUUUGUUUCUUugu-5' miRNA (3' pairing; Seed) | AAGATG Imperfect | ☐ | M |
| 25668–25690 | Imperfect match | 5'-cctAGGC-GACATTAGAAAGGACt-3' UTR | 3'-gauUCCGUUUGUUUUGUUUCUUGu-5' miRNA (3' pairing; Seed) | AAGGAC Imperfect | ☐ | M |
| 27692–27718 | Offset 6mer | 5'-taAAAACAAGCAGAATGTGAAAGAAaa-3' UTR | 3'-gaUUCCGUUUGUUUUG---UUUCUUgu-5' miRNA (3' pairing; Seed) | AAAGAA Offset 6mer | ☐ | M |
| 27778–27801 | Offset 6mer | 5'-tagtctgtcACAAACAAAAGAATt-3' UTR | 3'-gauuccguuUGUUUUGUUUCUUGu-5' miRNA (3' pairing; Seed) | AAAGAA Offset 6mer | ☐ | M |
| 28471–28492 | Offset 6mer | 5'-aatAGGT-AGCAAGA-AAAGAAat-3' UTR | 3'-gauUCCGUUUGUUUUGUUUCUUgu-5' miRNA (3' pairing; Seed) | AAAGAA Offset 6mer | ☐ | M |

| 2D Structure | Local AU | Position | Conservation | Predicted By |
|---|---|---|---|---|
| Offset 6mer<br>341 … 365<br>5'-acAGGACATACTCAGAAAAAGAAgg-3' UTR<br>|:| || || |:| ||||||<br>3'-gaUUCCGUUUG-UUUUGUUUCUUgu-5' miRNA<br>16 15 14 13 … 7 6 5 4 3 2<br>3'pairing Seed | 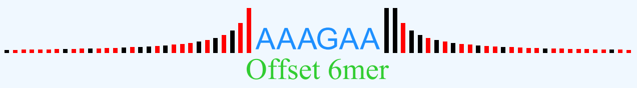 | | ☐ | M |
| Offset 6mer<br>373 … 395<br>5'-aaGAAGAAAACAAAA-AAAGAAgc-3' UTR<br>:| | ||||||| ||||||<br>3'-gaUUCCGUUUGUUUUGUUUCUUgu-5' miRNA<br>16 15 14 13 … 7 6 5 4 3 2<br>3'pairing Seed | 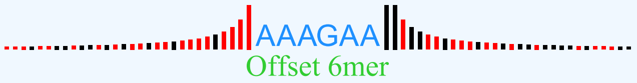 | | ☐ | M |
| Imperfect match<br>469 … 493<br>5'-ctAATGCAGATGACAGCAAGGGACa-3' UTR<br>|| |||:|::| |:|||:|:||<br>3'-gaUUCCGUUUGUU-UUGUUUCUUGu-5' miRNA<br>16 15 14 13 … 7 6 5 4 3 2<br>3'pairing Seed | 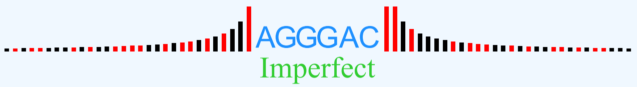 | | ☐ | M |
| Offset 6mer<br>573 … 597<br>5'-gaAAGATAAAATTAATAAAAGAAat-3' UTR<br>||| : |||: || ||||||<br>3'-gaUUCCG-UUUGUUUUGUUUCUUgu-5' miRNA<br>16 15 14 13 … 7 6 5 4 3 2<br>3'pairing Seed | 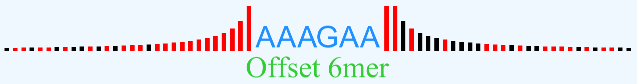 | | ☐ | M |
| Imperfect match<br>884 … 908<br>5'-tccAAGCTAATAAACATGGAGAACg-3' UTR<br>| || ||:||| |:::|||||<br>3'-gauUCCGUUUGUU-UGUUUCUUGu-5' miRNA<br>16 15 14 13 … 7 6 5 4 3 2<br>3'pairing Seed | 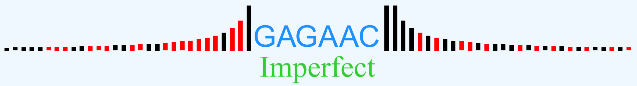 | | ☐ | M |

| 2D Structure | Local AU | Position | Conservation | Predicted By |
|---|---|---|---|---|

```
203              8mer      222
   5'-accgAAGA-GACTGATGGAAa-3'  UTR
          |  ||  |||  |||||||
   3'-gacgUCCUACUGUCUACCUUu-5'  miRNA
          16 15 14 13   7 6 5 4 3 2
        3'pairing       Seed
```

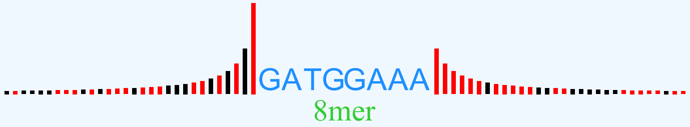


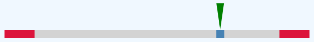

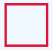

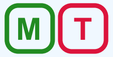

| 2D Structure | Local AU | Position | Conservation | Predicted By |
|---|---|---|---|---|

```
205              8mer     226
   5'-cgaAGAGACTGATGGAAAAAAa-3'  UTR
         |||:  ::| |||||||
   3'-gacUCUUCCGUUUCCUUUUUUu-5'  miRNA
             16 15 14 13  7 6 5 4 3 2
         3'pairing       Seed
```

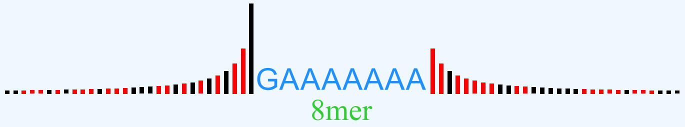


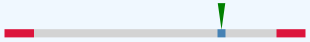

M T

| 2D Structure | Local AU | Position | Conservation | Predicted By |
|---|---|---|---|---|
| Imperfect match<br>1171 1192<br>5'-actcttctagAAAGGAAAAGAa-3' UTR<br>3'-gacucuuccgUUUCCUUUUUUu-5' miRNA<br>3'pairing Seed | AAAAGA<br>Imperfect | | | M |
| Imperfect match<br>2908 2927<br>5'-aaaAGAAAG-AAA-GAAAGAAa-3' UTR<br>3'-gacUCUUCCGUUUCCUUUUUUu-5' miRNA<br>3'pairing Seed | AAAGAA<br>Imperfect | | | M |
| Imperfect match<br>2936 2957<br>5'-gaGAGAGAGAGAAAGAAAGAAa-3' UTR<br>3'-gaCUCUUCCGUUUCCUUUUUUu-5' miRNA<br>3'pairing Seed | AAAGAA<br>Imperfect | | | M |
| Imperfect match<br>2954 2973<br>5'-gaaAGAAAG-AAA-GAAAGAAa-3' UTR<br>3'-gacUCUUCCGUUUCCUUUUUUu-5' miRNA<br>3'pairing Seed | AAAGAA<br>Imperfect | | | M |
| Imperfect match<br>2997 3020<br>5'-gaGGGAGGGACTGAGGGAAGAAAa-3' UTR<br>3'-gaCUCUUCC--GUUUCCUUUUUUu-5' miRNA<br>3'pairing Seed | AAGAAA<br>Imperfect | | | M |
| Imperfect match<br>3058 3080<br>5'-gaGAGGAGAGGAGAGGAGAGAAg-3' UTR<br>3'-gaCUCUUC-CGUUUCCUUUUUUu-5' miRNA<br>3'pairing Seed | AGAGAA<br>Imperfect | | | M |
| Imperfect match<br>3140 3159<br>5'-aaaAGAAAG-AAA-GAAAGAAa-3' UTR<br>3'-gacUCUUCCGUUUCCUUUUUUu-5' miRNA<br>3'pairing Seed | AAAGAA<br>Imperfect | | | M |
| Imperfect match<br>3174 3195<br>5'-gaGAGAGAGAGAAAGAAAGAAa-3' UTR<br>3'-gaCUCUUCCGUUUCCUUUUUUu-5' miRNA<br>3'pairing Seed | AAAGAA<br>Imperfect | | | M |
| Imperfect match<br>3192 3211<br>5'-gaaAGAAAG-AAA-GAAAGAAa-3' UTR<br>3'-gacUCUUCCGUUUCCUUUUUUu-5' miRNA<br>3'pairing Seed | AAAGAA<br>Imperfect | | | M |
| Imperfect match<br>3208 3227<br>5'-gaaAGAAAG-AAA-GAAAGAAa-3' UTR<br>3'-gacUCUUCCGUUUCCUUUUUUu-5' miRNA<br>3'pairing Seed | AAAGAA<br>Imperfect | | | M |
| Imperfect match<br>3247 3270<br>5'-gaGGGAGGGACTGAGGGAAGAAAa-3' UTR<br>3'-gaCUCUUCC--GUUUCCUUUUUUu-5' miRNA<br>3'pairing Seed | AAGAAA<br>Imperfect | | | M |
| Imperfect match<br>3318 3340<br>5'-gaGAGGAGAGGAGAGGAGAGAAg-3' UTR<br>3'-gaCUCUUC-CGUUUCCUUUUUUu-5' miRNA<br>3'pairing Seed | AGAGAA<br>Imperfect | | | M |
| Imperfect match<br>3345 3367<br>5'-gaGGGAGGGAGAAAGGAAGGAAg-3' UTR<br>3'-gaCUCUUCC-GUUUCCUUUUUUu-5' miRNA<br>3'pairing Seed | AAGGAA<br>Imperfect | | | M |
| Imperfect match<br>3757 3778<br>5'-ctGAGGAGACAGAGGAAGAAGg-3' UTR<br>3'-gaCUCUUCCGUUUCCUUUUUUu-5' miRNA<br>3'pairing Seed | AAGAAG<br>Imperfect | | | M |
| Imperfect match<br>4658 4679<br>5'-agttccctGCAAGAGAAGAAAg-3' UTR<br>3'-gacucuucCGUUUCCUUUUUUu-5' miRNA<br>3'pairing Seed | AAGAAA<br>Imperfect | | | M |
| Offset 6mer<br>5263 5287<br>5'-aactGAAGGTAATTAAAGAAAAAGa-3' UTR<br>3'-gacuCUUCCGU---UUCCUUUUUUu-5' miRNA<br>3'pairing Seed | GAAAAA<br>Offset 6mer | | | M |
| Imperfect match<br>8317 8338<br>5'-ggGGGTATTCAAAGGAAGGAAc-3' UTR<br>3'-gaCUCUUCCGUUUCCUUUUUUu-5' miRNA<br>3'pairing Seed | AAGGAA<br>Imperfect | | | M |
| Imperfect match<br>15099 15122<br>5'-caaAGAAGTACCTGGGGAAGAAAa-3' UTR<br>3'-gacUCUUC--CGUUUCCUUUUUUu-5' miRNA<br>3'pairing Seed | AAGAAA<br>Imperfect | | | M |
| Imperfect match<br>15718 15739<br>5'-gactaGAGGTTGGGGAGAAAAg-3' UTR<br>3'-gacucUUCCGUUUCCUUUUUUu-5' miRNA<br>3'pairing Seed | AGAAAA<br>Imperfect | | | M |
| Imperfect match<br>16573 16594<br>5'-cacAGAAGGCCAAAGAGGAAAt-3' UTR<br>3'-gacUCUUCCGUUUCCUUUUUUu-5' miRNA<br>3'pairing Seed | AGGAAA<br>Imperfect | | | M |
| Offset 6mer<br>17779 17800<br>5'-tcattttaaAAAGGAAAAAGa-3' UTR<br>3'-gacucuuccgUUUCCUUUUUUu-5' miRNA<br>3'pairing Seed | GAAAAA<br>Offset 6mer | | | M |
| Offset 6mer<br>20797 20818<br>5'-atGATATAGAGGGGGAAAAAGa-3' UTR<br>3'-gaCUCUUCCGUUUCCUUUUUUu-5' miRNA<br>3'pairing Seed | GAAAAA<br>Offset 6mer | | | M |
| Imperfect match<br>22668 22688<br>5'-gcacaGAGGCAAA-GAAGAAAt-3' UTR<br>3'-gacucUUCCGUUUCCUUUUUUu-5' miRNA<br>3'pairing Seed | AAGAAA<br>Imperfect | | | M |
| Imperfect match<br>29882 29902<br>5'-ctaAGAACTCCAA-GAGAAAAg-3' UTR<br>3'-gacUCUUCCGUUUCCUUUUUUu-5' miRNA<br>3'pairing Seed | AGAAAA<br>Imperfect | | | M |

| 2D Structure | Local AU | Position | Conservation | Predicted By |
|---|---|---|---|---|
| 62 7mer-m8 82<br>5'-gaagctaAGAGAGGAAGGACc-3' UTR<br>\|:\|\|\| \|\|\|\|\|\|\|<br>3'-gacccccUUUCU-CUUCCUGu-5' miRNA<br>16 15 14 13 7 6 5 4 3 2<br>3'pairing Seed | 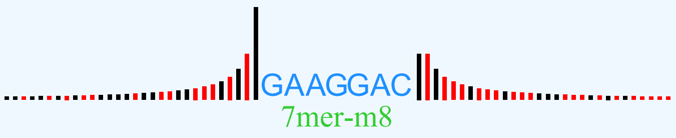 | 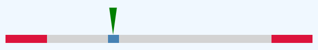 | | M T |
| 111 7mer-m8 130<br>5'-taacccactATGGAAGGACg-3' UTR<br>\| :\|\|\|\|\|\|\|<br>3'-gacccccuuUCUCUUCCUGu-5' miRNA<br>16 15 14 13 7 6 5 4 3 2<br>3'pairing Seed | 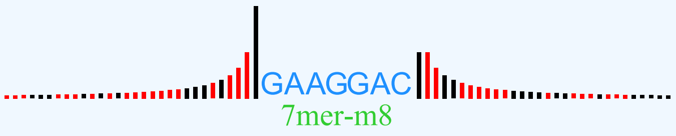 | 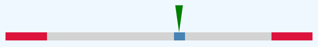 | | M T |

| 2D Structure | Local AU | Position | Conservation | Predicted By |
| --- | --- | --- | --- | --- |
| 54 7mer-m8 76<br>5'-cagTCTAGGCTGACTCCCCCAGc-3' UTR<br>3'-auaGGAGUCGACU-CGGGGGUCu-5' miRNA<br>16 15 14 13 7 6 5 4 3 2<br>3'pairing Seed | 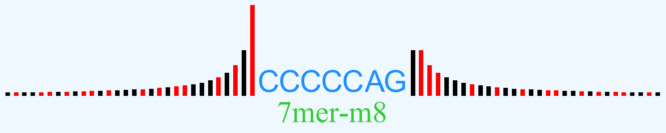 | 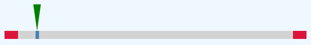 | | M T |
| 243 7mer-m8 262<br>5'-aaTCAGCAGCT--GCCCCCAGc-3' UTR<br>3'-auAGGAGUCGACUCGGGGGUCu-5' miRNA<br>16 15 14 13 7 6 5 4 3 2<br>3'pairing Seed | 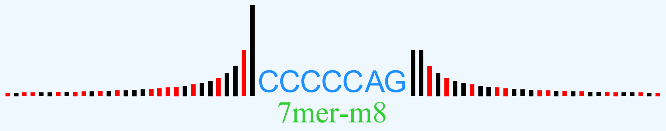 | 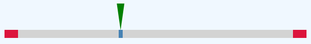 | | M T |

<table>
<tr><th>2D Structure</th><th>Local AU</th><th>Position</th><th>Conservation</th><th>Predicted By</th></tr>
<tr><td>290 7mer-m8 313<br>5'-cagcGTACACAGTCTGCAATGTTt-3' UTR<br>|| | | ||||:||||||<br>3'-gaaaCACG-GACAGAUGUUACAAu-5' miRNA<br>16 15 14 13 7 6 5 4 3 2<br>3'pairing Seed</td><td>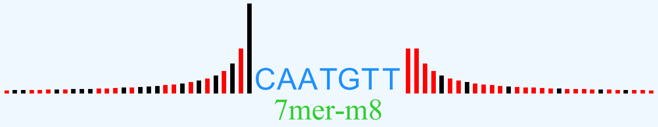</td><td>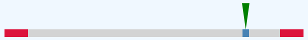</td><td></td><td>M T</td></tr>
</table>

| 2D Structure | Local AU | Position | Conservation | Predicted By |
|---|---|---|---|---|
| Imperfect match<br>75 96<br>5'-caTGGTGGGGGAACGGGAGAGt -3' UTR<br>3'-ccACCUCACCGUCUCCCUUUCu-5' miRNA<br>16 15 14 13 7 6 5 4 3 2<br>3'pairing Seed | 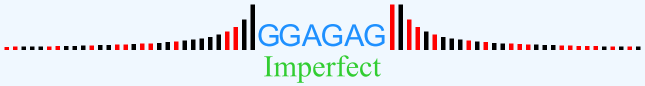 | 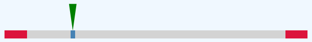 | | M |
| 7mer-m8<br>338 359<br>5'-cagaaaatatggtcGGGAAAGc-3' UTR<br>3'-ccaccucaccgucuCCUUUCu-5' miRNA<br>16 15 14 13 7 6 5 4 3 2<br>3'pairing Seed | 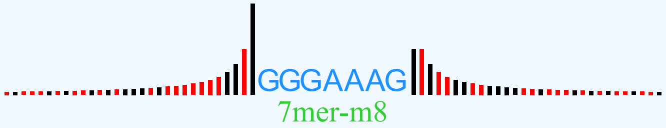 | 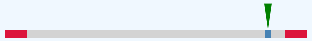 | | M T |

| 2D Structure | Local AU | Position | Conservation | Predicted By |
|---|---|---|---|---|
| 47 7mer-m8 67<br>5'-atcggatAGAAAAGTGTGTTt-3' UTR<br>3'-cuggaccUCUGCACACACAAu-5' miRNA<br>16 15 14 13 7 6 5 4 3 2<br>3'pairing Seed | 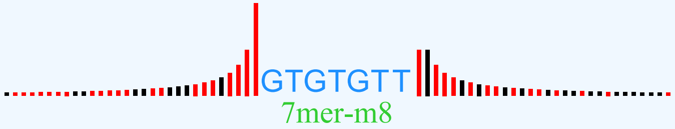 | 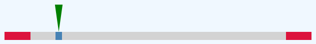 | | M T |
| 293 7mer-m8 311<br>5'-taaaTTGAGA--TGTGTGTTt-3' UTR<br>3'-cuggACCUCUGCACACACAAu-5' miRNA<br>16 15 14 13 7 6 5 4 3 2<br>3'pairing Seed | 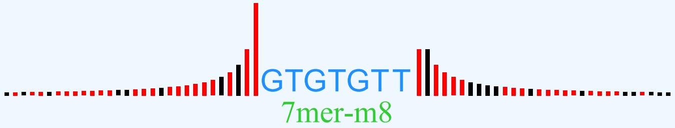 | 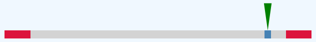 | | M T |

| 2D Structure | Local AU | Position | Conservation | Predicted By |
|---|---|---|---|---|
| 131 7mer-m8 150<br>5'-cgtttgTGAAGAGACCCAGt-3' UTR<br>3'-cgauggAGUUCUCUGGGUCa-5' miRNA<br>16 15 14 13 7 6 5 4 3 2<br>3'pairing Seed | 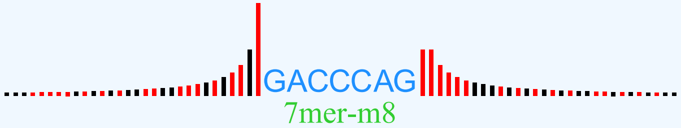 | 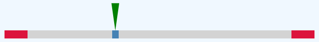 | | M T |

| 2D Structure | Local AU | Position | Conservation | Predicted By |
|---|---|---|---|---|
| 7mer-m8<br>110 5'-gaCTACAATC--AGTCAGAAAACt-3' 131 UTR<br>3'-aaGA-GUAAGGUUGGGUCUUUUGu-5' miRNA<br>16 15 14 13 / 7 6 5 4 3 2<br>3'pairing Seed | AGAAAAC<br>7mer-m8 | | | M T |
| Offset 6mer<br>435 5'-ttTTTATT-CAACTTAGAAAAga-3' 456 UTR<br>3'-aaGAGUAAGGUUGGGUCUUUUgu-5' miRNA<br>16 15 14 13 / 7 6 5 4 3 2<br>3'pairing Seed | AGAAAA<br>Offset 6mer | | | M |

<table>
<tr><th>2D Structure</th><th>Local AU</th><th>Position</th><th>Conservation</th><th>Predicted By</th></tr>
<tr><td>113 7mer-m8 139<br>5'-attaCATTTGGAAAATATGAGAAAACt-3' UTR<br>||||: ||: : |||||||<br>3'-aagaGUAAG---GUUG-GGUCUUUUGu-5' miRNA<br>16 15 14 13 7 6 5 4 3 2<br>3'pairing Seed</td><td>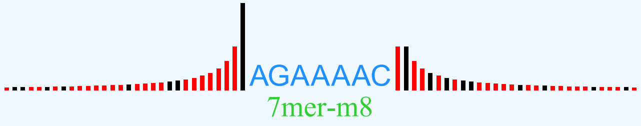</td><td>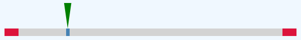</td><td></td><td>M T</td></tr>
<tr><td>479 7mer-m8 502<br>5'-tttgaATCCCTGCAGAAGAAAACc-3' UTR<br>|| || :| |||||||<br>3'-aagagUAAGGUUG-GGUCUUUUGu-5' miRNA<br>16 15 14 13 7 6 5 4 3 2<br>3'pairing Seed</td><td>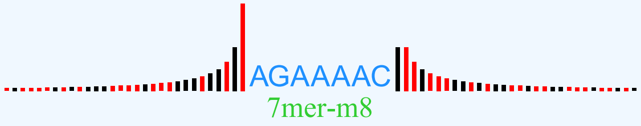</td><td>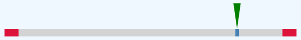</td><td></td><td>M T</td></tr>
</table>

<table>
<tr><th>2D Structure</th><th>Local AU</th><th>Position</th><th>Conservation</th><th>Predicted By</th></tr>
<tr><td>Imperfect match<br>756 778<br>5'-ttCGGAGAGTACTACAGATTCCa-3' UTR<br>||||:|: || ||:|||||<br>3'-cgGCCUUUUCUG--GUUUAAGGu-5' miRNA<br>16 15 14 13 7 6 5 4 3 2<br>3'pairing Seed</td><td>GATTCC<br>Imperfect</td><td></td><td></td><td>M</td></tr>
<tr><td>8mer<br>985 1005<br>5'-aaaaccAGAAAACAAATTCCa-3' UTR<br>|:| | ||||||||<br>3'-cggccuUUUCUGGUUUAAGGu-5' miRNA<br>16 15 14 13 7 6 5 4 3 2<br>3'pairing Seed</td><td>AAATTCCA<br>8mer</td><td></td><td></td><td>M T</td></tr>
</table>

| 2D Structure | Local AU | Position | Conservation | Predicted By |
|---|---|---|---|---|
| 82 7mer-m8 104<br>5'-ggccagaaCCCTG-CAGCTCTCCc-3' UTR<br>3'-agacccagGAGACGGUCGAGAGGu-5' miRNA<br>16 15 14 13 7 6 5 4 3 2<br>3'pairing Seed | 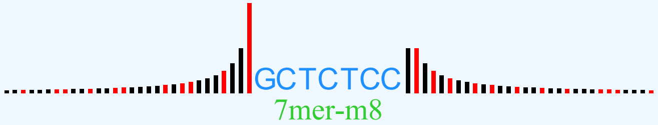 | 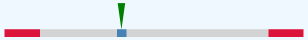 | | M T |
| 95 7mer-m8 117<br>5'-cagctcTCC-CTGACTGCTCTCCt-3' UTR<br>3'-agacccAGGAGACGGUCGAGAGGu-5' miRNA<br>16 15 14 13 7 6 5 4 3 2<br>3'pairing Seed | 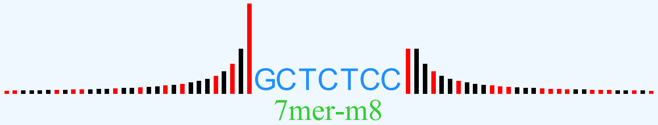 | 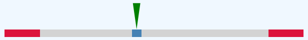 | | M T |

<table>
<tr><th>2D Structure</th><th>Local AU</th><th>Position</th><th>Conservation</th><th>Predicted By</th></tr>
<tr><td>Imperfect<br>match<br>350 374<br>5'-atgTCTGTCTATTGGGATACAGGTg -3' UTR<br>|||||||  :::||||||:||<br>3'-ggaAGACAGAGUGUUCUAUGUUCAa-5' miRNA<br>16 15 14 13 7 6 5 4 3 2<br>3'pairing Seed</td><td>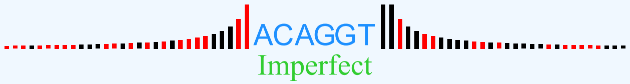</td><td>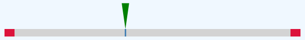</td><td></td><td>M</td></tr>
<tr><td>Imperfect<br>match<br>425 449<br>5'-gtTCCAGTC-CGTGGGAGTGCAAGTa -3' UTR<br>|  |  |||  |::::||  |:|||||<br>3'-ggAAGACAGAGUGUUCU-AUGUUCAa-5' miRNA<br>16 15 14 13 7 6 5 4 3 2<br>3'pairing Seed</td><td>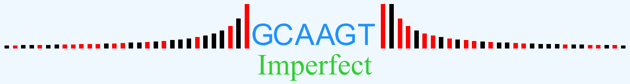</td><td>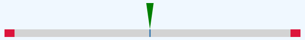</td><td></td><td>M</td></tr>
</table>

| 2D Structure | Local AU | Position | Conservation | Predicted By |
|---|---|---|---|---|
| 38 7mer-m8 58<br>5'-gtttgacTGGAGACCCGGATg-3' UTR<br>\|:\|\|:\|\|\|\|\|\|\|<br>3'-ucacacaAUCUUUGGGCCUAu-5' miRNA<br>16 15 14 13 7 6 5 4 3 2<br>3'pairing Seed | 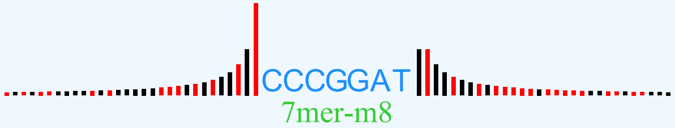 | 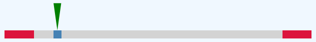 | ☐ | M T |

<table>
<tr><th>2D Structure</th><th>Local AU</th><th>Position</th><th>Conservation</th><th>Predicted By</th></tr>
<tr><td>76 6mer 99<br>5'-caAGATCCTCATAGGCTACAGTCg-3' UTR<br>|| |||:| || |:||||||<br>3'-ggUC-AGGGG--UCGGGUGUCAGu-5' miRNA<br>16 15 14 13 7 6 5 4 3 2<br>3'pairing Seed</td><td>ACAGTC<br>6mer</td><td></td><td></td><td>M</td></tr>
<tr><td>115 7mer-m8 136<br>5'-ctggagCCAGGCCACACAGTCt-3' UTR<br>|| :||| |||||||<br>3'-ggucagGGGUCGG-GUGUCAGu-5' miRNA<br>16 15 14 13 7 6 5 4 3 2<br>3'pairing Seed</td><td>CACAGTC<br>7mer-m8</td><td></td><td></td><td>M T</td></tr>
</table>

<table>
<tr><th>2D Structure</th><th>Local AU</th><th>Position</th><th>Conservation</th><th>Predicted By</th></tr>
<tr><td>Imperfect<br>match<br>251 279<br>5'-acCTGTCACGCCTTTCAACTATCGCTGGc -3' UTR<br>|| ||||: | |||||| || ||||<br>3'-ggGA-AGUGU-GUUAGUUGA-AGGGACCg-5' miRNA<br>16 15 14 13 7 6 5 4 3 2<br>3'pairing Seed</td><td>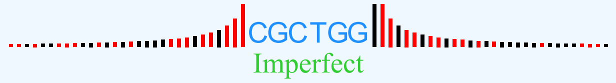</td><td>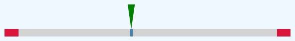</td><td></td><td>M</td></tr>
<tr><td>7mer-m8<br>512 537<br>5'-gaTTTCAGGTGGGACTCTTCCCTGGt-3' UTR<br>:|||| :::: ||||||||<br>3'-ggGAAGUGUGUUAGUUGAAGGGACCg-5' miRNA<br>16 15 14 13 7 6 5 4 3 2<br>3'pairing Seed</td><td>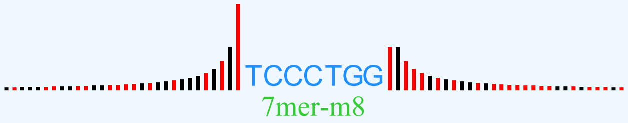</td><td>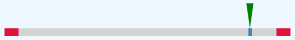</td><td></td><td>M T</td></tr>
</table>

| 2D Structure | Local AU | Position | Conservation | Predicted By |
|---|---|---|---|---|
| 31 7mer-m8 57<br>5'-aagccTACCCAGAGGACGTTCCCTGGc-3' UTR<br>3'-gggaaGUGUGUUAGUUG-AAGGGACCg-5' miRNA<br>16 15 14 13 7 6 5 4 3 2<br>3'pairing Seed | 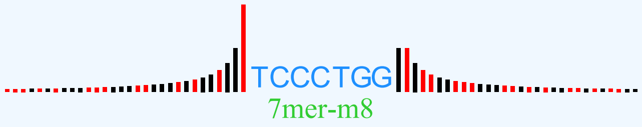 | 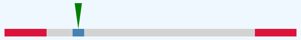 | | M T |

| 2D Structure | Local AU | Position | Conservation | Predicted By |
|---|---|---|---|---|
| see alignment below | 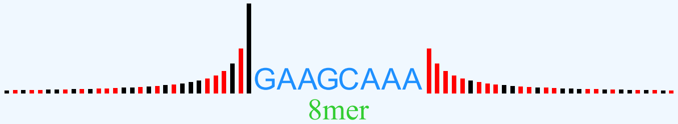 | 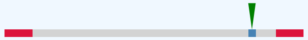 | | M T |

```
253                8mer      273
   5'-gtCT-GGGAAA-GAGGAAGCAAa-3'  UTR
          ||  ||||||  :|:|||||||
   3'-aaGACCCCUUUGUUUCUUCGUUc-5'  miRNA
              16 15 14 13  7 6 5 4 3 2
            3'pairing       Seed
```

<table>
<tr><th>2D Structure</th><th>Local AU</th><th>Position</th><th>Conservation</th><th>Predicted By</th></tr>
<tr><td>350 6mer 371<br>5'-ctGGAGAACTGC-AGGAGTCAGt-3' UTR<br>||||| || ||:||||||<br>3'-cuCCUCUCUUCGUUCUUCAGUCu-5' miRNA<br>16 15 14 13 7 6 5 4 3 2<br>3'pairing Seed</td><td>AGTCAG<br>6mer</td><td></td><td></td><td>M</td></tr>
<tr><td>617 8mer 639<br>5'-tgaGAGACAAAAAACAAGTCAGa-3' UTR<br>|||| || || ||||||<br>3'-cucCUCUCUUCGUUCUUCAGUCu-5' miRNA<br>16 15 14 13 7 6 5 4 3 2<br>3'pairing Seed</td><td>AAGTCAGA<br>8mer</td><td></td><td></td><td>M T</td></tr>
</table>

| 2D Structure | Local AU | Position | Conservation | Predicted By |
|---|---|---|---|---|
| 63 7mer-m8 80<br>5'-gggCGGATGCCTCCCTCg-3' UTR<br>\|:\|\|: \|\|\|\|\|\|\|\|<br>3'-cggGUCUGGGGAGGGAGg-5' miRNA<br>16 15 14 13 7 6 5 4 3 2<br>3'pairing Seed | CTCCCTC<br>7mer-m8 | | | M T |
| 84 7mer-m8 101<br>5'-gagCGGATGCCTCCCTCg-3' UTR<br>\|:\|\|: \|\|\|\|\|\|\|\|<br>3'-cggGUCUGGGGAGGGAGg-5' miRNA<br>16 15 14 13 7 6 5 4 3 2<br>3'pairing Seed | CTCCCTC<br>7mer-m8 | | | M T |

| 2D Structure | Local AU | Position | Conservation | Predicted By |
|---|---|---|---|---|
| Imperfect match<br>276 295<br>5'-tgggtgGCTTGACAGAGTCt-3' UTR<br>3'-gauggaCGGACUGUCUCGGu-5' miRNA<br>16 15 14 13 7 6 5 4 3 2<br>3'pairing Seed | 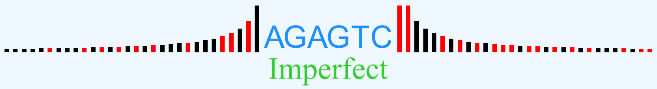 | 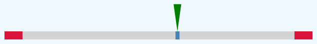 | | M |
| 302 7mer-m8 321<br>5'-agACATTCTACACAGAGCCt-3' UTR<br>3'-gaUGGACGGACUGUCUCGGu-5' miRNA<br>16 15 14 13 7 6 5 4 3 2<br>3'pairing Seed | 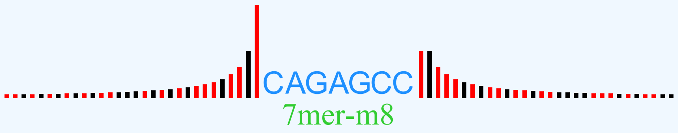 | 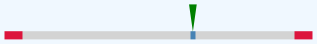 | | M T |

| 2D Structure | Local AU | Position | Conservation | Predicted By |
|---|---|---|---|---|
| 450 7mer-m8 469<br>5'-tagggaGCTTCTCAGAGCCg-3' UTR<br>\|\|:\| \|\|\|\|\|\|\|<br>3'-gauggaCGGACUGUCUCGGu-5' miRNA<br>16 15 14 13 7 6 5 4 3 2<br>3'pairing Seed | CAGAGCC<br>7mer-m8 | | | M T |
| 537 Offset 6mer 557<br>5'-ggGCATGTGTGAGCAGAGCTa-3' UTR<br>:\| \|\|: \|\|\| \|\|\|\|\|:<br>3'-gaUGGACGGACU-GUCUCGGu-5' miRNA<br>16 15 14 13 7 6 5 4 3 2<br>3'pairing Seed | CAGAGC<br>Offset 6mer | | | M |
| 635 7mer-m8 655<br>5'-acACACTCCCTCGCAGAGCCc-3' UTR<br>\|\| \|\| \|\|\| :\|\|\|\|\|\|\|<br>3'-gaUG-GACGGACUGUCUCGGu-5' miRNA<br>16 15 14 13 7 6 5 4 3 2<br>3'pairing Seed | CAGAGCC<br>7mer-m8 | | | M T |

| 2D Structure | Local AU | Position | Conservation | Predicted By |
|---|---|---|---|---|
| 237 8mer 256<br>5'-gttGGCTTTGCCAGGATGGa-3' UTR<br>3'-uucCCGAAACAGUCCUACCg-5' miRNA<br>16 15 14 13 7 6 5 4 3 2<br>3'pairing Seed | 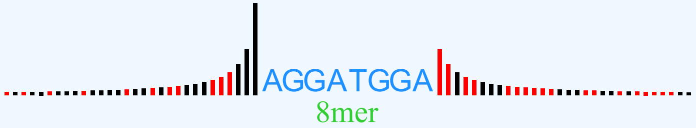 | 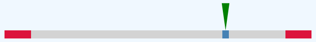 | | M T |

<table>
<tr><th>2D Structure</th><th>Local AU</th><th>Position</th><th>Conservation</th><th>Predicted By</th></tr>
<tr><td>Imperfect match<br>156 180<br>5'-gctttGTCTTGACAACCATGTAAGg -3' UTR<br>|| ||:|| ||||:|||<br>3'-ccaccCAUAAUUG--AGUACGUUCa-5' miRNA<br>16 15 14 13 7 6 5 4 3 2<br>3'pairing Seed</td><td>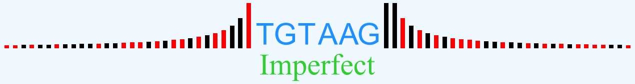</td><td>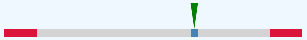</td><td></td><td>M</td></tr>
<tr><td>Imperfect match<br>216 237<br>5'-gaTGGG-ACCTGTTCATGCAGGg -3' UTR<br>|||| | ::||||||:|<br>3'-ccACCCAUAAUUGAGUACGUUCa-5' miRNA<br>16 15 14 13 7 6 5 4 3 2<br>3'pairing Seed</td><td>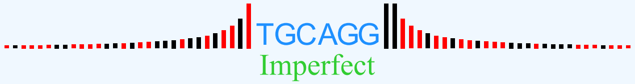</td><td>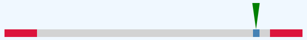</td><td></td><td>M</td></tr>
</table>

| 2D Structure | Local AU | Position | Conservation | Predicted By |
| --- | --- | --- | --- | --- |
| Offset 6mer<br>238 260<br>5'-agagaacATTATGTTATGCAAaa-3' UTR<br>\|\|\|\| \|:\|\|\|\|\|\|<br>3'-ccacccaUAAUUGAGUACGUUCa-5' miRNA<br>16 15 14 13 7 6 5 4 3 2<br>3'pairing Seed | 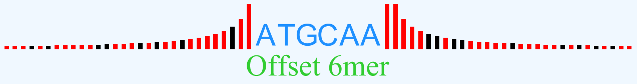 | 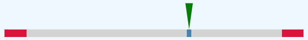 | ☐ | M |
| 8mer<br>290 309<br>5'-agcaaGTACGAA---ATGCAAGa-3' UTR<br>\|\|\| \|\| \|\|\|\|\|\|\|<br>3'-ccaccCAUAAUUGAGUACGUUCa-5' miRNA<br>16 15 14 13 7 6 5 4 3 2<br>3'pairing Seed | 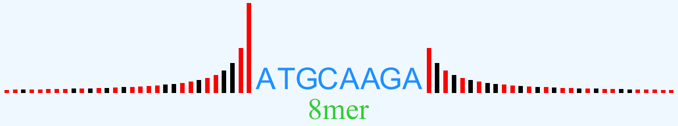 | 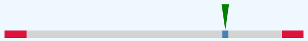 | ☐ | M T |

| 2D Structure | Local AU | Position | Conservation | Predicted By |
|---|---|---|---|---|
| 125 7mer-m8 145<br>5'-gttTTTCTTTGTCCTGGACTg-3' UTR<br>:\| \|: : \|\|\|\|\|\|\|\|<br>3'-cauGAUGGUGGAGGACCUGAa-5' miRNA<br>16 15 14 13 7 6 5 4 3 2<br>3'pairing Seed | 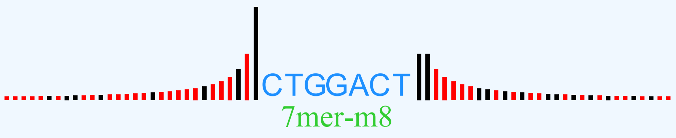 | 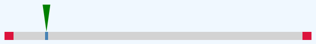 | | M T |
| 238 7mer-m8 257<br>5'-ttACTA-TATGCCCTGGACTc-3' UTR<br>\|\|\|\| :\|: \|\|\|\|\|\|\|\|<br>3'-caUGAUGGUGGAGGACCUGAa-5' miRNA<br>16 15 14 13 7 6 5 4 3 2<br>3'pairing Seed | 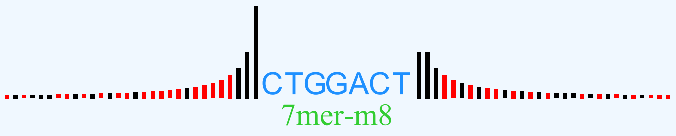 | 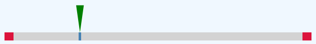 | | M T |

| 2D Structure | Local AU | Position | Conservation | Predicted By |
|---|---|---|---|---|
| see below | 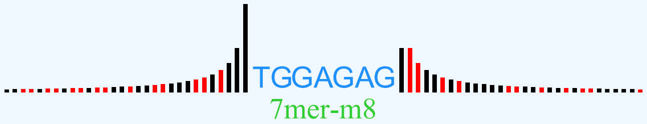 | 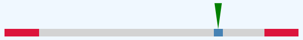 | | M T |

```
165                    7mer-m8  190
   5'-agGAGCAAGCCATGTGCCTGGAGAGg-3'  UTR
          :|:|||| ||  |||  ||||||||
   3'-ucUUUGUUC-GU-CAC-GACCUCUCu-5'  miRNA
              16 15  14 13    7 6 5 4 3 2
           3'pairing           Seed
```

| 2D Structure | Local AU | Position | Conservation | Predicted By |
|---|---|---|---|---|
| 118 7mer-m8 139<br>5'-ttcAACCAGCTGT-TTGGAGAGg-3' UTR<br>\|\|\| \|\|\| \|\| :\|\|\|\|\|\|\|<br>3'-ucuUUGUUCGUCACGACCUCUCu-5' miRNA<br>16 15 14 13 7 6 5 4 3 2<br>3'pairing Seed | 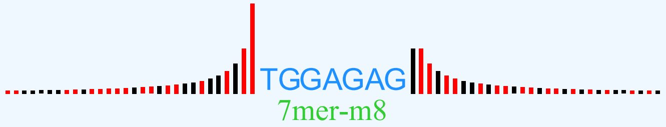 | 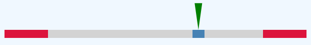 | | M T |

<table>
<tr><th>2D Structure</th><th>Local AU</th><th>Position</th><th>Conservation</th><th>Predicted By</th></tr>
<tr><td>91 7mer-m8 112<br>5'-ttctGCAAGGA-ACCTGGAGAGg-3' UTR<br>:||||  |   ||||||||<br>3'-ucuuUGUUCGUCACGACCUCUCu-5' miRNA<br>16 15 14 13 7 6 5 4 3 2<br>3'pairing Seed</td><td>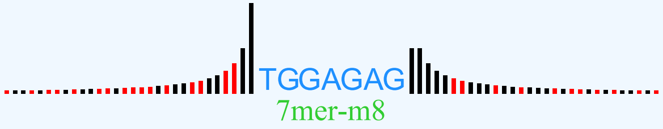<br></td><td>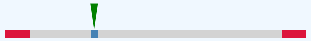</td><td></td><td>M T</td></tr>
<tr><td>122 7mer-m8 143<br>5'-acctggctGAAGTG-TGGAGAGg-3' UTR<br>|  ||||  |||||||<br>3'-ucuuuguuCGUCACGACCUCUCu-5' miRNA<br>16 15 14 13 7 6 5 4 3 2<br>3'pairing Seed</td><td>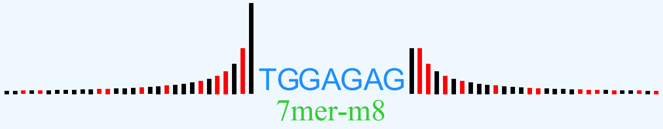<br></td><td>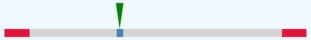</td><td></td><td>M T</td></tr>
</table>

<table>
<tr><th>2D Structure</th><th>Local AU</th><th>Position</th><th>Conservation</th><th>Predicted By</th></tr>
<tr><td>220 8mer 241<br>5'-taggaaaaGTAGCCATCAAAAa-3' UTR<br>||| |||||||<br>3'-aggaagaaCAUAUAUAGUUUUg-5' miRNA<br>16 15 14 13 7 6 5 4 3 2<br>3'pairing Seed</td><td>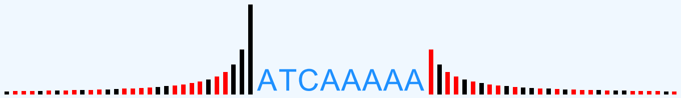<br>ATCAAAAA<br>8mer</td><td>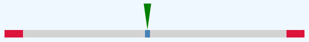</td><td></td><td>M T</td></tr>
<tr><td>Imperfect match<br>382 403<br>5'-agCTTGTTGTACACATCAAGGc -3' UTR<br>||| ||||| | |||||::<br>3'-agGAAGAACAUAUAUAGUUUUg-5' miRNA<br>16 15 14 13 7 6 5 4 3 2<br>3'pairing Seed</td><td>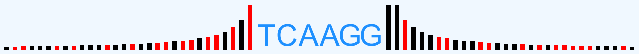<br>TCAAGG<br>Imperfect</td><td>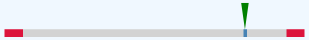</td><td></td><td>M</td></tr>
</table>

<table>
<tr><th>2D Structure</th><th>Local AU</th><th>Position</th><th>Conservation</th><th>Predicted By</th></tr>
<tr><td>Imperfect match<br>115 137<br>5'-ctGTCAGTCTGCTCGGTCTCTTc -3' UTR<br>||| ||||| || |:|:|||<br>3'-ggCAGACAGAC-AGGCGGGGAAu-5' miRNA<br>16 15 14 13 7 6 5 4 3 2<br>3'pairing Seed</td><td>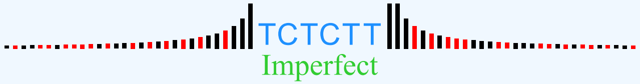</td><td>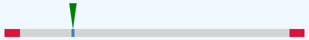</td><td>☐</td><td>M</td></tr>
<tr><td>7mer-m8<br>354 375<br>5'-tttTCTGGGTGTGTGCCCCTTc-3' UTR<br>|||| ||| :|||||||<br>3'-ggcAGACAGACAGGCGGGGAAu-5' miRNA<br>16 15 14 13 7 6 5 4 3 2<br>3'pairing Seed</td><td>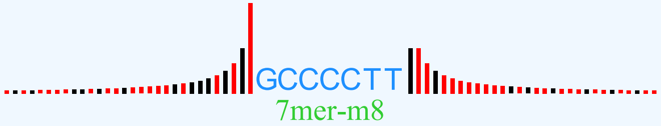</td><td>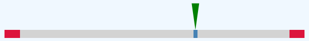</td><td>☐</td><td>M T</td></tr>
</table>

<table>
<tr><th>2D Structure</th><th>Local AU</th><th>Position</th><th>Conservation</th><th>Predicted By</th></tr>
<tr><td>236 7mer-m8 257<br>5'-ggcatcctGTCAGCCAGCCTGt-3' UTR<br>:|||| |||||||<br>3'-uggcacccUAGUCUGUCGGACa-5' miRNA<br>16 15 14 13 7 6 5 4 3 2<br>3'pairing Seed</td><td>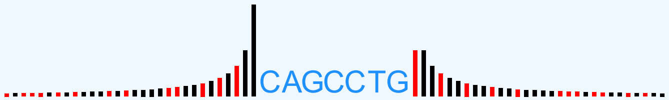<br>CAGCCTG<br>7mer-m8</td><td>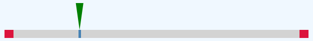</td><td></td><td>M T</td></tr>
<tr><td>290 7mer-m8 309<br>5'-caccacaGAT--GCCAGCCTGt-3' UTR<br>||| | |||||||<br>3'-uggcaccCUAGUCUGUCGGACa-5' miRNA<br>16 15 14 13 7 6 5 4 3 2<br>3'pairing Seed</td><td>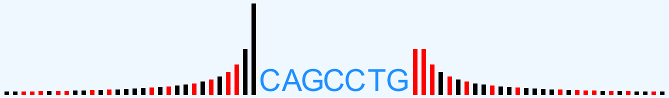<br>CAGCCTG<br>7mer-m8</td><td>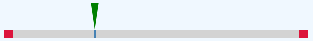</td><td></td><td>M T</td></tr>
<tr><td>389 7mer-m8 410<br>5'-accaaccactgccACAGCCTGc-3' UTR<br>||||||||<br>3'-uggcacccuagucUGUCGGACa-5' miRNA<br>16 15 14 13 7 6 5 4 3 2<br>3'pairing Seed</td><td>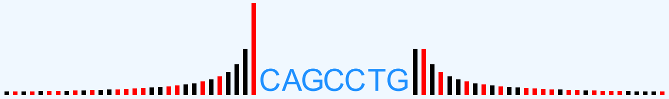<br>CAGCCTG<br>7mer-m8</td><td>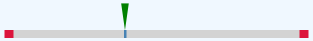</td><td></td><td>M T</td></tr>
<tr><td>612 7mer-m8 632<br>5'-tcagacttATCA-ACAGCCTGt-3' UTR<br>|||| ||||||||<br>3'-uggcacccUAGUCUGUCGGACa-5' miRNA<br>16 15 14 13 7 6 5 4 3 2<br>3'pairing Seed</td><td>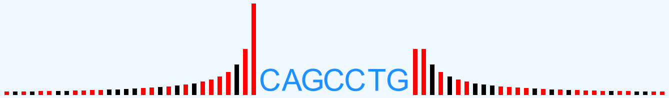<br>CAGCCTG<br>7mer-m8</td><td>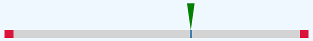</td><td></td><td>M T</td></tr>
</table>

<table>
<tr><th>2D Structure</th><th>Local AU</th><th>Position</th><th>Conservation</th><th>Predicted By</th></tr>
<tr><td>986 7mer-m8 1008<br>5'-gcCAGGCGTGAGATGGCAAGGAt-3' UTR<br>||:| | :||:|||||||<br>3'-ugGUUCAACCCUUAUCGUUCCUu-5' miRNA<br>16 15 14 13 7 6 5 4 3 2<br>3'pairing Seed</td><td>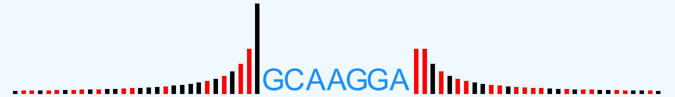<br>GCAAGGA<br>7mer-m8</td><td>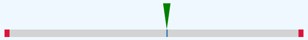</td><td>☐</td><td>M T</td></tr>
<tr><td>1167 8mer 1188<br>5'-acgcAGCTGATCA-AGCAAGGAa-3' UTR<br>|| || | |||||||||<br>3'-ugguUCAACCCUUAUCGUUCCUu-5' miRNA<br>16 15 14 13 7 6 5 4 3 2<br>3'pairing Seed</td><td>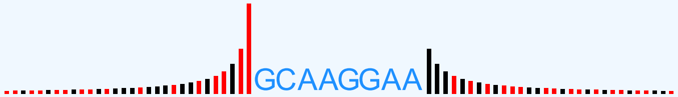<br>GCAAGGAA<br>8mer</td><td>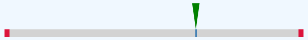</td><td>☐</td><td>M T</td></tr>
<tr><td>1243 8mer 1265<br>5'-taagatcttctttgAGCAAGGAa-3' UTR<br>||||||||<br>3'-ugguucaacccuuaUCGUUCCUu-5' miRNA<br>16 15 14 13 7 6 5 4 3 2<br>3'pairing Seed</td><td>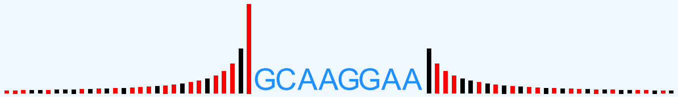<br>GCAAGGAA<br>8mer</td><td>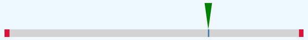</td><td>☐</td><td>M T</td></tr>
</table>

| 2D Structure | Local AU | Position | Conservation | Predicted By |
|---|---|---|---|---|
| 189 7mer-m8 214<br>5'-acCAATCTGGGGCATGGTGCAAGGAc-3' UTR<br>3'-ugGUUCAACCCU-UAU--CGUUCCUu-5' miRNA<br>16 15 14 13 7 6 5 4 3 2<br>3'pairing Seed | 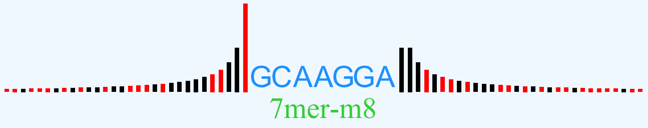 | 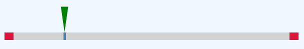 | | M T |
| 759 7mer-m8 781<br>5'-acaacacTGACCAAAGCAAGGAt-3' UTR<br>3'-ugguucaACCCUUAUCGUUCCUu-5' miRNA<br>16 15 14 13 7 6 5 4 3 2<br>3'pairing Seed | 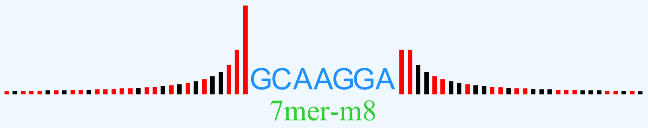 | 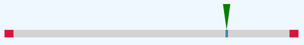 | | M T |

| 2D Structure | Local AU | Position | Conservation | Predicted By |
| --- | --- | --- | --- | --- |
| 135 6mer 157<br>5'-gttGCATTTGGAGTCTGGCTCCt-3' UTR<br>|  ||:|||||  |:||||||<br>3'-gucCUUAGACCUC-GGCCGAGGa-5' miRNA<br>16 15 14 13 7 6 5 4 3 2<br>3'pairing Seed | 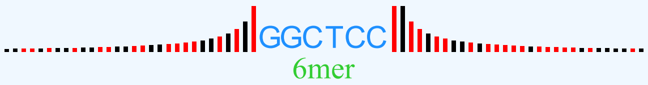 | 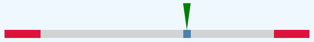 | | M |

| 2D Structure | Local AU | Position | Conservation | Predicted By |
|---|---|---|---|---|
| 636 8mer 659<br>5'-cagagaGCCAGTAGACTTACAGAa-3' UTR<br>3'-augaaaCGGGUA-GUGAAUGUCUa-5' miRNA<br>16 15 14 13 7 6 5 4 3 2<br>3'pairing Seed | 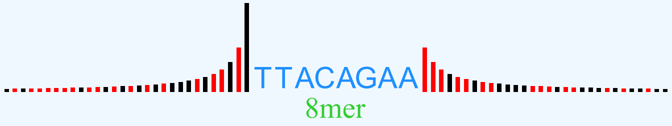 | 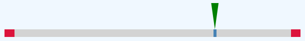 | | M T |
| 775 7mer-m8 797<br>5'-agaacgaaCTATCACTTACAGAg-3' UTR<br>3'-augaaacgGGUAGUGAAUGUCUa-5' miRNA<br>16 15 14 13 7 6 5 4 3 2<br>3'pairing Seed | 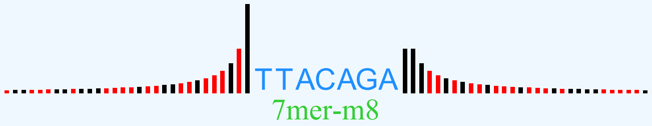 | 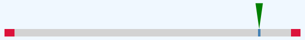 | | M T |

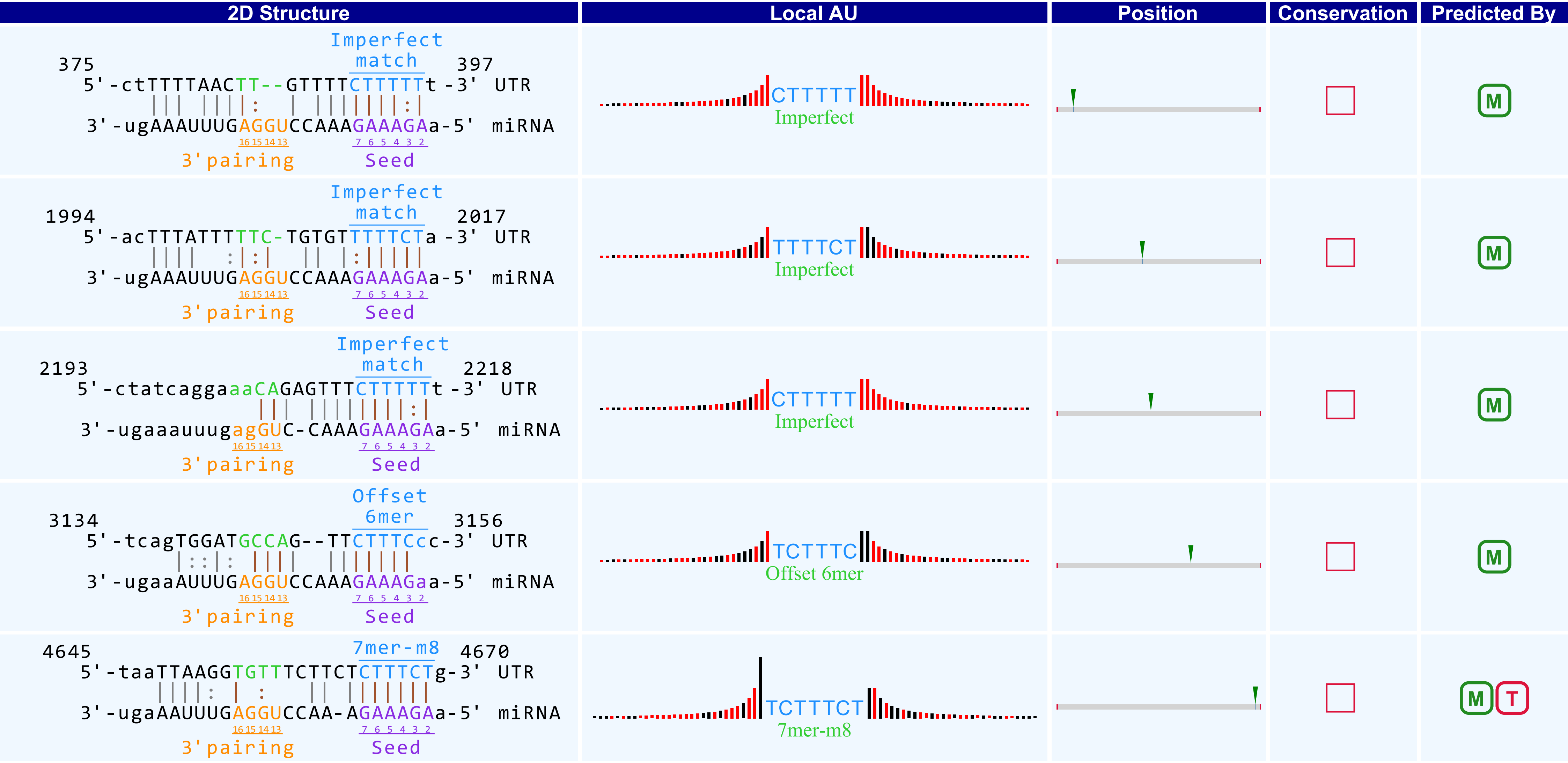

2D Structure
Local AU
Position
Conservation
Predicted By
Imperfect match
375
397
5'-ctTTTTAACTT--GTTTTCTTTTTt-3' UTR
3'-ugAAAUUUGAGGUCCAAAGAAAGAa-5' miRNA
16 15 14 13
7 6 5 4 3 2
3'pairing
Seed
CTTTTT
Imperfect
M
Imperfect match
1994
2017
5'-acTTTATTTTTC-TGTGTTTTTCTa-3' UTR
3'-ugAAAUUUGAGGUCCAAAGAAAGAa-5' miRNA
16 15 14 13
7 6 5 4 3 2
3'pairing
Seed
TTTTCT
Imperfect
M
Imperfect match
2193
2218
5'-ctatcaggaaaCAGAGTTTCTTTTTt-3' UTR
3'-ugaaauuugagGUC-CAAAGAAAGAa-5' miRNA
16 15 14 13
7 6 5 4 3 2
3'pairing
Seed
CTTTTT
Imperfect
M
Offset 6mer
3134
3156
5'-tcagTGGATGCCAG--TTCTTTCcc-3' UTR
3'-ugaaAUUUGAGGUCCAAAGAAAGaa-5' miRNA
16 15 14 13
7 6 5 4 3 2
3'pairing
Seed
TCTTTC
Offset 6mer
M
7mer-m8
4645
4670
5'-taaTTAAGGTGTTTCTTCTCTTTCTg-3' UTR
3'-ugaAAUUUGAGGUCCAA-AGAAAGAa-5' miRNA
16 15 14 13
7 6 5 4 3 2
3'pairing
Seed
TCTTTCT
7mer-m8
M
T

| 2D Structure | Local AU | Position | Conservation | Predicted By |
|---|---|---|---|---|
| 162 7mer-m8 187<br>5'-cagtGGCTTCTCCGGATCTGGCTCCt-3' UTR<br>\|\|\|\| \|\|\|\| :\|\|\|\|\|\|\|<br>3'-cguuCCGACUAGGC--GGACCGAGGu-5' miRNA<br>16 15 14 13 7 6 5 4 3 2<br>3'pairing Seed | 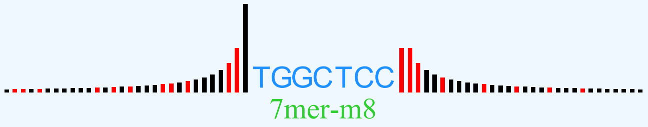 | 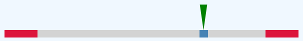 | | M T |

| 2D Structure | Local AU | Position | Conservation | Predicted By |
|---|---|---|---|---|
| 130 7mer-m8 157<br>5'-cgttGGTTGCATTTGGAGTCTGGCTCCt-3' UTR<br>3'-cguuCCGAC-UAGG---CGGACCGAGGu-5' miRNA<br>16 15 14 13 7 6 5 4 3 2<br>3'pairing Seed | 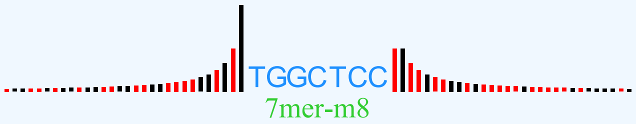 | 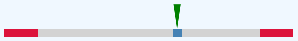 | | M T |

| 2D Structure | Local AU | Position | Conservation | Predicted By |
|---|---|---|---|---|
| 7mer-m8<br>643 664<br>5'-aggtgaaTC-CTTTCCTTCCCTc-3' UTR<br>3'-gucggaaAGAGGACGGAAGGGAa-5' miRNA<br>3' pairing Seed | 7mer-m8 | | ☐ | M T |
| 7mer-m8<br>700 719<br>5'-caGCC-TTCTCTT--CTTCCCTg-3' UTR<br>3'-guCGGAAAGAGGACGGAAGGGAa-5' miRNA<br>3' pairing Seed | 7mer-m8 | | ☐ | M T |
| Imperfect match<br>6107 6128<br>5'-tccTCTTCCTCC-ATCTTCTCTg-3' UTR<br>3'-gucGGAAAGAGGACGGAAGGGAa-5' miRNA<br>3' pairing Seed | Imperfect | | ☐ | M |
| Imperfect match<br>6134 6156<br>5'-ctGTCTTTCCCCTGTCCTCTCTc-3' UTR<br>3'-guCGGAAAGAGGACGGAAGGGAa-5' miRNA<br>3' pairing Seed | Imperfect | | ☐ | M |
| Imperfect match<br>7198 7220<br>5'-taGGCTCTTTCCTACTTTCTCTt-3' UTR<br>3'-guCGGAAAGAGGACGGAAGGGAa-5' miRNA<br>3' pairing Seed | Imperfect | | ☐ | M |
| Imperfect match<br>8049 8072<br>5'-atGCCTTGACTTCTTCCTTTCCaa-3' UTR<br>3'-guCGGAA-AGAGGACGGAAGGGaa-5' miRNA<br>3' pairing Seed | Imperfect | | ☐ | M |
| Imperfect match<br>9146 9168<br>5'-tcctcaaTTTTATGTCTTCCTTt-3' UTR<br>3'-gucggaaAGAGGACGGAAGGGAa-5' miRNA<br>3' pairing Seed | Imperfect | | ☐ | M |
| 6mer<br>13930 13952<br>5'-ttaTCTAAATTCTTCTTTCCCTt-3' UTR<br>3'-gucGGAAAGAGGACGGAAGGGAa-5' miRNA<br>3' pairing Seed | 6mer | | ☐ | M |
| Imperfect match<br>16215 16238<br>5'-aaGCCAATTTTCCTATTTTCCTTt-3' UTR<br>3'-guCGG-AAAGAGGACGGAAGGGAa-5' miRNA<br>3' pairing Seed | Imperfect | | ☐ | M |
| Imperfect match<br>16422 16444<br>5'-ggggaTTTCTTTTTCCTTTCCTc-3' UTR<br>3'-gucggAAAGAGGACGGAAGGGAa-5' miRNA<br>3' pairing Seed | Imperfect | | ☐ | M |
| Imperfect match<br>16438 16461<br>5'-tttCCTCTTCTCTACCCTTCCTTc-3' UTR<br>3'-gucGGA-AAGAGGACGGAAGGGAa-5' miRNA<br>3' pairing Seed | Imperfect | | ☐ | M |
| Imperfect match<br>23910 23931<br>5'-agcacacagTCCT-CCTTCCTTt-3' UTR<br>3'-gucggaaagAGGACGGAAGGGAa-5' miRNA<br>3' pairing Seed | Imperfect | | ☐ | M |
| Imperfect match<br>24059 24080<br>5'-actgaaTTC-CCTGTCTTCCTTc-3' UTR<br>3'-gucggaAAGAGGACGGAAGGGAa-5' miRNA<br>3' pairing Seed | Imperfect | | ☐ | M |
| Imperfect match<br>24832 24854<br>5'-aatCCCTCATCATATCTTTCCTt-3' UTR<br>3'-gucGGAAAGAGGACGGAAGGGAa-5' miRNA<br>3' pairing Seed | Imperfect | | ☐ | M |
| 6mer<br>26618 26640<br>5'-tgGTCTGCCATTTGATTTCCCTt-3' UTR<br>3'-guCGGAAAGAGGACGGAAGGGAa-5' miRNA<br>3' pairing Seed | 6mer | | ☐ | M |
| Imperfect match<br>28205 28228<br>5'-tgaaCTCTCTTGTTGACTTCTCTa-3' UTR<br>3'-gucgGAAAGAG-GACGGAAGGGAa-5' miRNA<br>3' pairing Seed | Imperfect | | ☐ | M |
| Imperfect match<br>29042 29065<br>5'-tccaggcgCTGCCCGCCTTCTCTg-3' UTR<br>3'-gucggaaaGA-GGACGGAAGGGAa-5' miRNA<br>3' pairing Seed | Imperfect | | ☐ | M |
| Imperfect match<br>29188 29209<br>5'-tctCCTATCCTCCT--CTTCTCTt-3' UTR<br>3'-gucGGA-AAGAGGACGGAAGGGAa-5' miRNA<br>3' pairing Seed | Imperfect | | ☐ | M |
| Imperfect match<br>32802 32824<br>5'-caGCTTTCTTTCTTTCTTTCTTt-3' UTR<br>3'-guCGGAAAGAGGACGGAAGGGAa-5' miRNA<br>3' pairing Seed | Imperfect | | ☐ | M |
| Offset 6mer<br>33739 33763<br>5'-actgCTTTCTTGGCTACCTTCCCag-3' UTR<br>3'-gucgGAAAGAG--GACGGAAGGGaa-5' miRNA<br>3' pairing Seed | Offset 6mer | | ☐ | M |
| Imperfect match<br>33834 33857<br>5'-cacCCATTCTTCCTCCTTTTCCTg-3' UTR<br>3'-gucGGAAAG-AGGACGGAAGGGAa-5' miRNA<br>3' pairing Seed | Imperfect | | ☐ | M |
| Imperfect match<br>33856 33875<br>5'-tgGCC--TCTTTT-CCTTCTCTc-3' UTR<br>3'-guCGGAAAGAGGACGGAAGGGAa-5' miRNA<br>3' pairing Seed | Imperfect | | ☐ | M |
| Imperfect match<br>35330 35351<br>5'-cgaaCTTCCTTAT-TCTTCCTTc-3' UTR<br>3'-gucgGAAAGAGGACGGAAGGGAa-5' miRNA<br>3' pairing Seed | Imperfect | | ☐ | M |
| 8mer<br>39346 39370<br>5'-accaTTTTAGGTTGCATCTTCCCTa-3' UTR<br>3'-gucgGAAAGAGGACG--GAAGGGAa-5' miRNA<br>3' pairing Seed | 8mer | | ☐ | M T |
| Offset 6mer<br>40282 40305<br>5'-ctGCCTTACTGGCTTACTTCCCct-3' UTR<br>3'-guCGGAAAGA-GGACGGAAGGGaa-5' miRNA<br>3' pairing Seed | Offset 6mer | | ☐ | M |
| 6mer<br>41079 41102<br>5'-gtcaCTTTGATTTGCATTTCCCTg-3' UTR<br>3'-gucgGAAAGAGGACG-GAAGGGAa-5' miRNA<br>3' pairing Seed | 6mer | | ☐ | M |
| Imperfect match<br>43872 43896<br>5'-cttTCTTTCCTTCCTTTCTTTCCTt-3' UTR<br>3'-gucGGAAAG--AGGACGGAAGGGAa-5' miRNA<br>3' pairing Seed | Imperfect | | ☐ | M |
| Imperfect match<br>43891 43914<br>5'-ttcCTTCTTTTCTTTCCTTCCTTt-3' UTR<br>3'-gucGGA-AAGAGGACGGAAGGGAa-5' miRNA<br>3' pairing Seed | Imperfect | | ☐ | M |
| Imperfect match<br>43907 43931<br>5'-cttCCTTTCTTTCCTTCCTTTCTTt-3' UTR<br>3'-gucGGAAAG--AGGACGGAAGGGAa-5' miRNA<br>3' pairing Seed | Imperfect | | ☐ | M |
| Imperfect match<br>43938 43960<br>5'-ttcCTTTTTTCTTTCCTTCCTTt-3' UTR<br>3'-gucGGAAAGAGGACGGAAGGGAa-5' miRNA<br>3' pairing Seed | Imperfect | | ☐ | M |
| Imperfect match<br>43953 43974<br>5'-cttCCTTTCTTTT-CCTTTCCTt-3' UTR<br>3'-gucGGAAAGAGGACGGAAGGGAa-5' miRNA<br>3' pairing Seed | Imperfect | | ☐ | M |
| Imperfect match<br>43973 43994<br>5'-tttCCTTTCTTTT-CCTTTCCTt-3' UTR<br>3'-gucGGAAAGAGGACGGAAGGGAa-5' miRNA<br>3' pairing Seed | Imperfect | | ☐ | M |
| Imperfect match<br>43995 44018<br>5'-tttCCTATCCTCTTTCCTTTCCTt-3' UTR<br>3'-gucGGA-AAGAGGACGGAAGGGAa-5' miRNA<br>3' pairing Seed | Imperfect | | ☐ | M |
| Imperfect match<br>44012 44034<br>5'-tttCCTTTCTCTTTTCTTTCCTt-3' UTR<br>3'-gucGGAAAGAGGACGGAAGGGAa-5' miRNA<br>3' pairing Seed | Imperfect | | ☐ | M |
| Imperfect match<br>44028 44051<br>5'-tttCCTTTTTCCTTTCCTTTCCTt-3' UTR<br>3'-gucGGAAAGAGG-ACGGAAGGGAa-5' miRNA<br>3' pairing Seed | Imperfect | | ☐ | M |
| Imperfect match<br>44050 44071<br>5'-tttCCTTTC-CTTTCCTTTCCTt-3' UTR<br>3'-gucGGAAAGAGGACGGAAGGGAa-5' miRNA<br>3' pairing Seed | Imperfect | | ☐ | M |
| Imperfect match<br>44066 44088<br>5'-ttcCTTTTCCCTTTCCTTTCCTc-3' UTR<br>3'-gucGGAAAGAGGACGGAAGGGAa-5' miRNA<br>3' pairing Seed | Imperfect | | ☐ | M |
| Offset 6mer<br>44435 44458<br>5'-gaGACTTACGTTATTCCTTCCCcg-3' UTR<br>3'-guCGGAAAG-AGGACGGAAGGGaa-5' miRNA<br>3' pairing Seed | Offset 6mer | | ☐ | M |
| 8mer<br>45610 45633<br>5'-tatttaTGTTCTTCCACTTCCCTa-3' UTR<br>3'-gucggaAAGAGGACG-GAAGGGAa-5' miRNA<br>3' pairing Seed | 8mer | | ☐ | M T |
| Offset 6mer<br>48601 48623<br>5'-ctcatgTCCTGCCGCCTTCCCct-3' UTR<br>3'-gucggaAAGAGGACGGAAGGGaa-5' miRNA<br>3' pairing Seed | Offset 6mer | | ☐ | M |
| Imperfect match<br>57611 57632<br>5'-tccTCTTCCTCC-ATCTTCTCTg-3' UTR<br>3'-gucGGAAAGAGGACGGAAGGGAa-5' miRNA<br>3' pairing Seed | Imperfect | | ☐ | M |
| Imperfect match<br>60996 61020<br>5'-tttTCTTTCTTCTGTTCTTTCTCTt-3' UTR<br>3'-gucGGAAAGAGGAC--GGAAGGGAa-5' miRNA<br>3' pairing Seed | Imperfect | | ☐ | M |
| Offset 6mer<br>61678 61701<br>5'-ctGCTTTCTTTGCTTCCTTCCCag-3' UTR<br>3'-guCGGAA-AGAGGACGGAAGGGaa-5' miRNA<br>3' pairing Seed | Offset 6mer | | ☐ | M |
| 8mer<br>67619 67643<br>5'-accaTTTTAGGTTGCATCTTCCCTa-3' UTR<br>3'-gucgGAAAGAGGACG--GAAGGGAa-5' miRNA<br>3' pairing Seed | 8mer | | ☐ | M T |
| 6mer<br>68987 69010<br>5'-gaaCCTTTTTAAAGTGTTTCCCTc-3' UTR<br>3'-gucGGAAAGAGGACG-GAAGGGAa-5' miRNA<br>3' pairing Seed | 6mer | | ☐ | M |
| 6mer<br>72292 72315<br>5'-gttgTTTTGATTTGCATTTCCCTg-3' UTR<br>3'-gucgGAAAGAGGACG-GAAGGGAa-5' miRNA<br>3' pairing Seed | 6mer | | ☐ | M |
| Imperfect match<br>72332 72355<br>5'-aacaTTTTTTCAGGTGCTTCTCTg-3' UTR<br>3'-gucgGAAAGAGGACG-GAAGGGAa-5' miRNA<br>3' pairing Seed | Imperfect | | ☐ | M |
| 8mer<br>73452 73473<br>5'-tttatgTTCTTCT-ACTTCCCTa-3' UTR<br>3'-gucggaAAGAGGACGGAAGGGAa-5' miRNA<br>3' pairing Seed | 8mer | | ☐ | M T |
| 7mer-m8<br>75591 75615<br>5'-ggaCCTTCTGTCATCCACTTCCCTt-3' UTR<br>3'-gucGGAA-AGAGGACG-GAAGGGAa-5' miRNA<br>3' pairing Seed | 7mer-m8 | | ☐ | M T |
| 8mer<br>75654 75679<br>5'-agtaTTTTATGGATTTTACTTCCCTa-3' UTR<br>3'-gucgGAAAGA---GGACGGAAGGGAa-5' miRNA<br>3' pairing Seed | 8mer | | ☐ | M T |

| 2D Structure | Local AU | Position | Conservation | Predicted By |
|---|---|---|---|---|
| 289 7mer-m8 311<br>5'-ggGGCTCTAGGGTGACTTCCCTg-3' UTR<br>3'-guCGGAAAGAGGACGGAAGGGAa-5' miRNA<br>16 15 14 13 7 6 5 4 3 2<br>3'pairing Seed | CTTCCCT<br>7mer-m8 | | ☐ | M T |
| 875 6mer 897<br>5'-tcatgggagTCTTCCTTTCCCTt-3' UTR<br>3'-gucggaaagAGGACGGAAGGGAa-5' miRNA<br>16 15 14 13 7 6 5 4 3 2<br>3'pairing Seed | TTCCCT<br>6mer | | ☐ | M |
| 1822 6mer 1844<br>5'-aaGCGTCTTGACTTCTTTCCCTt-3' UTR<br>3'-guCGGAAAGAGGACGGAAGGGAa-5' miRNA<br>16 15 14 13 7 6 5 4 3 2<br>3'pairing Seed | TTCCCT<br>6mer | | ☐ | M |
| 1879 7mer-m8 1901<br>5'-gaaagggagcCCTTGCTTCCCTg-3' UTR<br>3'-gucggaaagaGGACGGAAGGGAa-5' miRNA<br>16 15 14 13 7 6 5 4 3 2<br>3'pairing Seed | CTTCCCT<br>7mer-m8 | | ☐ | M T |
| 2161 7mer-m8 2183<br>5'-ggGGCTCTAGGGTGACTTCCCTg-3' UTR<br>3'-guCGGAAAGAGGACGGAAGGGAa-5' miRNA<br>16 15 14 13 7 6 5 4 3 2<br>3'pairing Seed | CTTCCCT<br>7mer-m8 | | ☐ | M T |

| 2D Structure | Local AU | Position | Conservation | Predicted By |
|---|---|---|---|---|
| Offset 6mer<br>60 83<br>5'-caATGGTACACCCGAACCCAGCac-3' UTR<br>3'-cgUA-CACGGUGGUGUGGGUCGag-5' miRNA<br>16 15 14 13 7 6 5 4 3 2<br>3'pairing Seed | 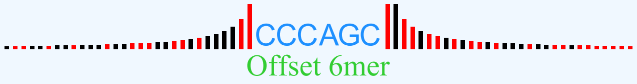 | 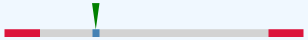 | | M |
| 7mer-m8<br>81 103<br>5'-cacaagatgagaAGACCCAGCTt-3' UTR<br>3'-cguacacgguggUGUGGGUCGAg-5' miRNA<br>16 15 14 13 7 6 5 4 3 2<br>3'pairing Seed | 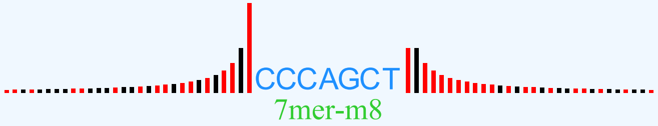 | 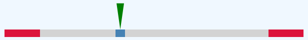 | | M T |

<table>
<tr><th>2D Structure</th><th>Local AU</th><th>Position</th><th>Conservation</th><th>Predicted By</th></tr>
<tr><td>36 8mer 63<br>5'-ccTCTTTGTGACAGTGGCTTACACCTCa-3' UTR<br>||:|::||  |   |  ||::|||||||<br>3'-ucAGGAGUAC-G--A-CGGGUGUGGAGu-5' miRNA<br>16 15 14 13 7 6 5 4 3 2<br>3'pairing Seed</td><td>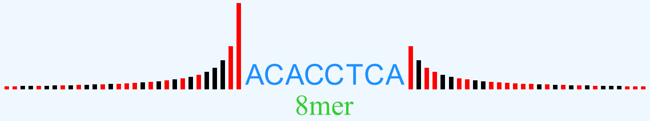
</td><td>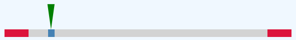</td><td></td><td>M T</td></tr>
<tr><td>Imperfect match<br>97 118<br>5'-gcTCTTCGTG--GCCCACATCTCc -3' UTR<br>||:||:||  |||||||:|||<br>3'-ucAGGAGUACGACGGGUGUGGAGu-5' miRNA<br>16 15 14 13 7 6 5 4 3 2<br>3'pairing Seed</td><td>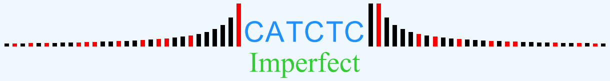
</td><td>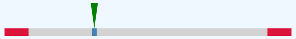</td><td></td><td>M</td></tr>
</table>

| 2D Structure | Local AU | Position | Conservation | Predicted By |
| --- | --- | --- | --- | --- |
| (see below) | 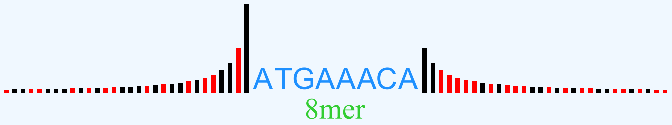 | 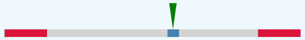 | | M T |



```
100                      8mer      123
  5'-gcGTCTGCTGAAGCACATGAAACa-3'  UTR
       |||  :| |||  ||  |||||||
  3'-cuCAG-UG-CUU-GUUUACUUUGu-5'  miRNA
           16 15  14 13  7 6 5 4 3 2
       3'pairing           Seed
```

| 2D Structure | Local AU | Position | Conservation | Predicted By |
|---|---|---|---|---|
| 268 7mer-m8 290<br>5'-gcaatGCTGC-TGCTG-TCACCACc-3' UTR<br>\|\|\|\|\| \| \|\|\| \|\|\|\|\|\|\|<br>3'-ggcgcCGACGAAAGACGAGUGGUGa-5' miRNA<br>16 15 14 13 7 6 5 4 3 2<br>3'pairing Seed | 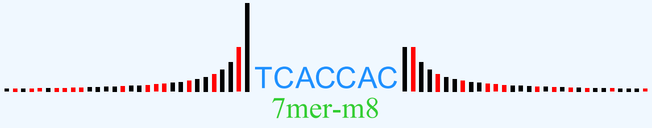 | 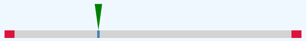 | | M T |
| 294 7mer-m8 317<br>5'-accaccatagccaCTG-TCACCACc-3' UTR<br>\|\|\| \|\|\|\|\|\|\|<br>3'-ggcgccgacgaaaGACGAGUGGUGa-5' miRNA<br>16 15 14 13 7 6 5 4 3 2<br>3'pairing Seed | 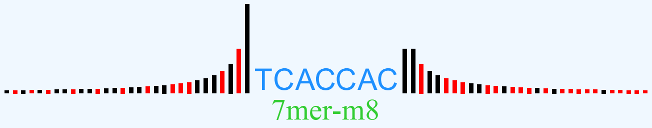 | 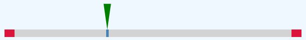 | | M T |

| 2D Structure | Local AU | Position | Conservation | Predicted By |
|---|---|---|---|---|
| 43 8mer 63<br>5'-aagaTTATCCTGTCCTTGTAa-3' UTR<br>:\|\|\|\|\| \|:\|\|\|\|\|\|\|<br>3'-uaagGAUAGGUCGGGAACAUu-5' miRNA<br>16 15 14 13 7 6 5 4 3 2<br>3'pairing Seed | 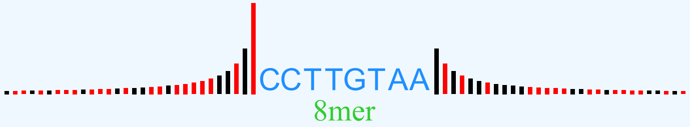 | 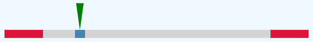 | | M T |

| 2D Structure | Local AU | Position | Conservation | Predicted By |
| --- | --- | --- | --- | --- |
| 210 7mer-m8 232<br>5'-cgtGATTCCAGTGGTGCCAGCCg-3' UTR<br>\|\|\|:\| \|\|\|\|\|\|\|\|\|\|\|<br>3'-uccCUAGG---ACCACGGUCGGa-5' miRNA<br>16 15 14 13 7 6 5 4 3 2<br>3'pairing Seed | 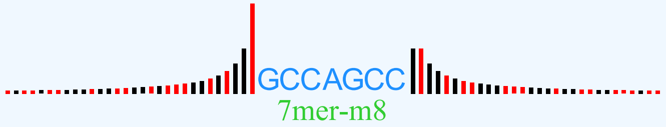 | 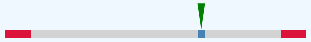 | | M T |

| 2D Structure | Local AU | Position | Conservation | Predicted By |
|---|---|---|---|---|
| 28 8mer 48<br>5'-atGAGTCTTGCCCGCCAGCCa-3' UTR<br>\| :\|\|:\|\| \|\|\|\|\|\|\|<br>3'-ucCCUAGGAC-CACGGUCGGa-5' miRNA<br>16 15 14 13 7 6 5 4 3 2<br>3'pairing Seed | 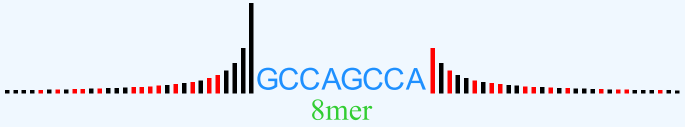 | 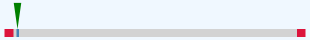 | ☐ | M T |
| 235 7mer-m8 255<br>5'-ggGCATCCTGTCAGCCAGCCt-3' UTR<br>\| \|\|\|\|\|\| \|\|\|\|\|\|\|<br>3'-ucCCUAGGAC-CACGGUCGGa-5' miRNA<br>16 15 14 13 7 6 5 4 3 2<br>3'pairing Seed | 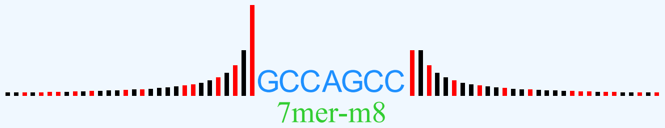 | 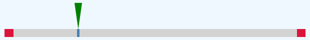 | ☐ | M T |
| 288 7mer-m8 307<br>5'-ctcaccaCAGATGCCAGCCt-3' UTR<br>\| \| \|\|\|\|\|\|\|\|\|<br>3'-uccc uagGACCACGGUCGGa-5' miRNA<br>16 15 14 13 7 6 5 4 3 2<br>3'pairing Seed | 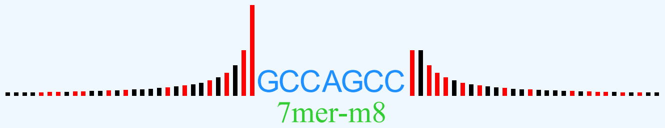 | 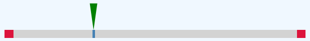 | ☐ | M T |
| 492 8mer 513<br>5'-gagccTCATGGGAGGCCAGCCa-3' UTR<br>\|\| \|\|\| \|\|\|\|\|\|\|<br>3'-ucccuAGGACC--ACGGUCGGa-5' miRNA<br>16 15 14 13 7 6 5 4 3 2<br>3'pairing Seed | 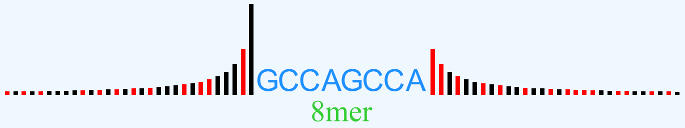 | 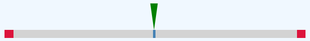 | ☐ | M T |

| 2D Structure | Local AU | Position | Conservation | Predicted By |
| --- | --- | --- | --- | --- |
| 58 8mer 82<br>5'-atATCATGTAACTGGACCCCTTTCa-3' UTR<br>\|\|\| \|\|\| \| \|\| \|\|\|\|\|\|\|<br>3'-gaUAGGACAGUCAC---GGGAAAGa-5' miRNA<br>16 15 14 13 7 6 5 4 3 2<br>3'pairing Seed | 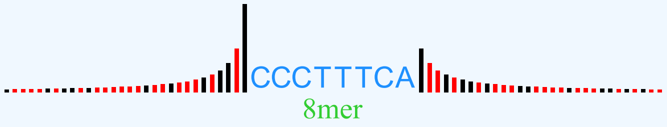 | 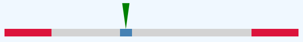 | | M T |

| 2D Structure | Local AU | Position | Conservation | Predicted By |
|---|---|---|---|---|
| 109 7mer-m8 132<br>5'-ttAGACCAATCCAAAACCCACAGc-3' UTR<br>\|\| \|\|\|\|:\|\| \|\|\|\|\|\|\|\|<br>3'-uuUC-GGUUGGG--UUGGGUGUCc-5' miRNA<br>16 15 14 13 7 6 5 4 3 2<br>3'pairing Seed | 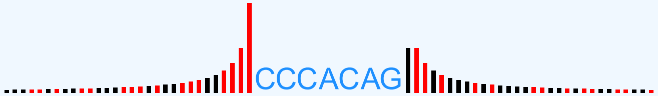 | 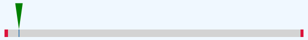 | | M T |
| Imperfect match<br>250 270<br>5'-atgcCCAGCCCAGCTTACAGc -3' UTR<br>\|\|\|:\|\|\|\|\|:\|::\|\|\|\|<br>3'-uuucGGUUGGGUUGGGUGUCc-5' miRNA<br>16 15 14 13 7 6 5 4 3 2<br>3'pairing Seed | 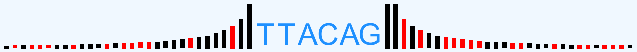 | 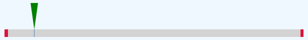 | | M |
| Imperfect match<br>1214 1233<br>5'-acAG-CAATCTAACTCACAGg -3' UTR<br>\|\| \|\|\|:\|:\|\|\|:\|\|\|\|\|<br>3'-uuUCGGUUGGGUUGGGUGUCc-5' miRNA<br>16 15 14 13 7 6 5 4 3 2<br>3'pairing Seed | 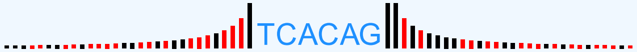 | 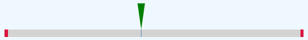 | | M |

| 2D Structure | Local AU | Position | Conservation | Predicted By |
|---|---|---|---|---|
| 232 7mer-m8 253<br>5'-aagctGCCTTTTACAGTCAGCc-3' UTR<br>\|\|\| :: \|\|\|\|\|\|\|<br>3'-gugucCGGUCGGGGUCAGUCGu-5' miRNA<br>16 15 14 13 7 6 5 4 3 2<br>3'pairing Seed | 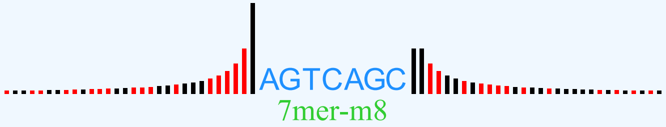 | 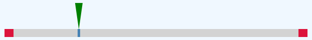 | | M T |
| Imperfect match<br>404 424<br>5'-agaAGTCCA-CCCCAGTTAGCa -3' UTR<br>\|\| \|\|\| \|\|\|\|\|\|\|:\|\|\|<br>3'-gugUCCGGUCGGGGUCAGUCGu-5' miRNA<br>16 15 14 13 7 6 5 4 3 2<br>3'pairing Seed | 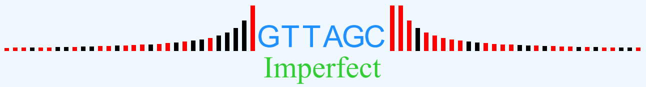 | 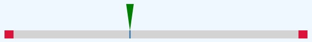 | | M |

| 2D Structure | Local AU | Position | Conservation | Predicted By |
|---|---|---|---|---|
| 279 7mer-m8 302<br>5'-tcATACTTTGAAAATTCGGAAGGt-3' UTR<br>\| \|\|\|\| :\| \|\| :\|\|\|\|\|\|\|<br>3'-ccUCUGAACUUCUUUGGCCUUCCa-5' miRNA<br>16 15 14 13 7 6 5 4 3 2<br>3'pairing Seed | 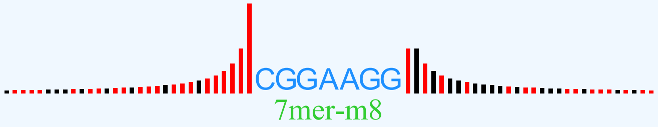 | 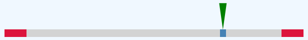 | | M T |

| 2D Structure | Local AU | Position | Conservation | Predicted By |
|---|---|---|---|---|
| Offset 6mer<br>56 77<br>5'-ggAAGCTGGAAG--ACCGGAAGtg-3' UTR<br>3'-ccUCUGAACUUCUUUGGCCUUCca-5' miRNA<br>16 15 14 13 7 6 5 4 3 2<br>3'pairing Seed | 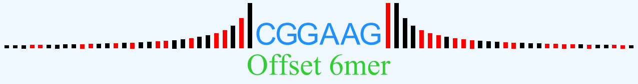  | 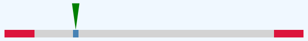 | ☐ | M |
| Offset 6mer<br>89 113<br>5'-caAGCCTTAACTGCATCCGGAAGtc-3' UTR<br>3'-ccUCUGAACU-UCUUUGGCCUUCca-5' miRNA<br>16 15 14 13 7 6 5 4 3 2<br>3'pairing Seed | 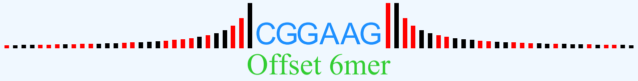  | 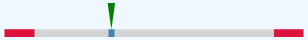 | ☐ | M |

<table>
<tr><th>2D Structure</th><th>Local AU</th><th>Position</th><th>Conservation</th><th>Predicted By</th></tr>
<tr><td><pre>
224                 7mer-m8  246
   5'-caCCAACGCTGG--CCAGTGGAAGg-3'  UTR
        ||| | |::|  ||||||||||
   3'-cgGGUCGGGGUCCAGGUCACCUUCc-5'  miRNA
               16 15 14 13    7 6 5 4 3 2
           3'pairing        Seed
</pre></td><td>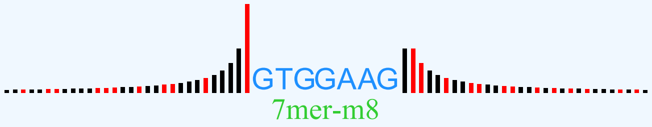
</td><td>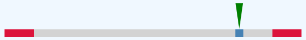</td><td></td><td>M T</td></tr>
</table>

| 2D Structure | Local AU | Position | Conservation | Predicted By |
|---|---|---|---|---|
| 304 7mer-m8 324<br>5'-acagAGAGTGGC-TTCACTCCt-3' UTR<br>3'-gugguCGAAUGGUGAGUGAGGa-5' miRNA<br>16 15 14 13 · 7 6 5 4 3 2<br>3'pairing · Seed | 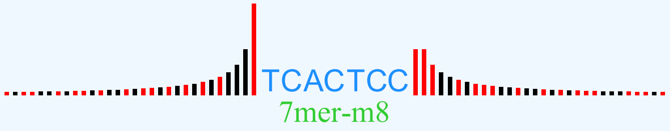 | 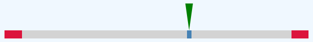 | | M T |
| 402 7mer-m8 423<br>5'-cgCCAGGAATGGAATCACTCCt-3' UTR<br>3'-guGGUCGAAUGGUGAGUGAGGa-5' miRNA<br>16 15 14 13 · 7 6 5 4 3 2<br>3'pairing · Seed | 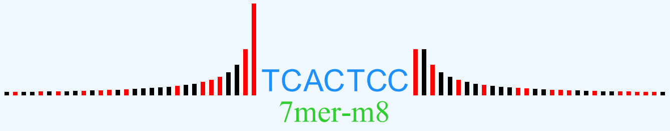 | 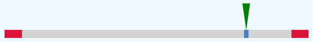 | | M T |

<table>
<tr><th>2D Structure</th><th>Local AU</th><th>Position</th><th>Conservation</th><th>Predicted By</th></tr>
<tr><td>199 8mer 221<br>5'-cgGGAAGATAGCTCAAATCCTTa-3' UTR<br>  |: |||:|||   |||||||<br>3'-ccCUGUCUGUCG-UCUUAGGAAa-5' miRNA<br>16 15 14 13 7 6 5 4 3 2<br>3'pairing Seed</td><td>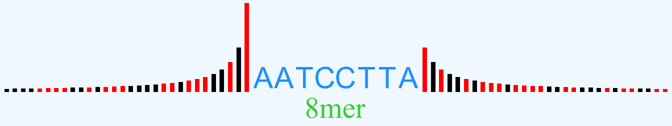</td><td>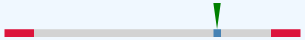</td><td></td><td>M T</td></tr>
<tr><td>218 7mer-m8 241<br>5'-cttATGTCCAGTTTGAAATCCTTg-3' UTR<br>   |::  |||  ::  |||||||<br>3'-cccUGUCUGUC--GUCUUAGGAAa-5' miRNA<br>16 15 14 13 7 6 5 4 3 2<br>3'pairing Seed</td><td>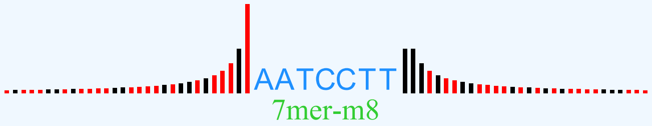</td><td>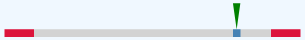</td><td></td><td>M T</td></tr>
</table>

| 2D Structure | Local AU | Position | Conservation | Predicted By |
| --- | --- | --- | --- | --- |
| 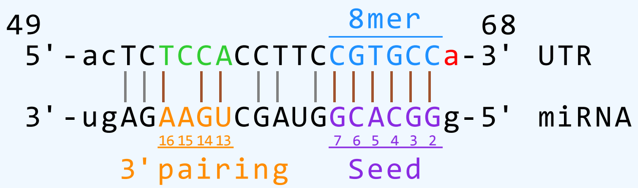 | 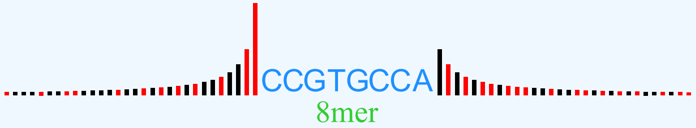 | 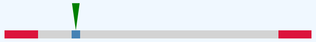 | 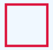 | 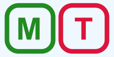 |

| 2D Structure | Local AU | Position | Conservation | Predicted By |
|---|---|---|---|---|
| 341 8mer 363<br>5'-aagcctgaaacaaCCTTCCTCCa-3' UTR<br>3'-uggacgaacgaggGAAAGGAGGa-5' miRNA<br>16 15 14 13 7 6 5 4 3 2<br>3'pairing Seed | TTCCTCCA<br>8mer | | | M T |
| Offset 6mer<br>938 963<br>5'-gtCTGCCTTGTTACATTTTTCCTCTt-3' UTR<br>3'-ugGAC-GAACGAGG--GAAAGGAGGa-5' miRNA<br>16 15 14 13 7 6 5 4 3 2<br>3'pairing Seed | TTCCTC<br>Offset 6mer | | | M |
| Imperfect match<br>5943 5965<br>5'-ccCTTCTTGCGTCTGTTTCTTCa -3' UTR<br>3'-ugGACGAACGAGGGAAAGGAGGa-5' miRNA<br>16 15 14 13 7 6 5 4 3 2<br>3'pairing Seed | TTCTTC<br>Imperfect | | | M |
| Imperfect match<br>8039 8063<br>5'-aaCTGTTTGCTGACTCTTTACTCCa -3' UTR<br>3'-ugGACGAACGA--GGGAAAGGAGGa-5' miRNA<br>16 15 14 13 7 6 5 4 3 2<br>3'pairing Seed | TACTCC<br>Imperfect | | | M |
| Imperfect match<br>8103 8125<br>5'-ttTTGTTTTTTCTATTTTCTCTg -3' UTR<br>3'-ugGACGAACGAGGGAAAGGAGGa-5' miRNA<br>16 15 14 13 7 6 5 4 3 2<br>3'pairing Seed | TTCTCT<br>Imperfect | | | M |
| 8702 7mer-m8 8723<br>5'-tgCTGTCTCCTCCC-TTCCTCCt-3' UTR<br>3'-ugGACGAACGAGGGAAAGGAGGa-5' miRNA<br>16 15 14 13 7 6 5 4 3 2<br>3'pairing Seed | TTCCTCC<br>7mer-m8 | | | M T |

<table>
<tr><th>2D Structure</th><th>Local AU</th><th>Position</th><th>Conservation</th><th>Predicted By</th></tr>
<tr><td>187 7mer-m8 207<br>5'-tttctAACAGCCTACAGGAGg-3' UTR<br>|||||   |||||||<br>3'-cccucUUGUCUCUUGUCCUCu-5' miRNA<br>16 15 14 13 7 6 5 4 3 2<br>3'pairing Seed</td><td>ACAGGAG<br>7mer-m8</td><td></td><td></td><td>M T</td></tr>
<tr><td>Offset 6mer<br>517 539<br>5'-ataAGACTAGGAGGAACAGGAag-3' UTR<br>|||  :||  :||||||||<br>3'-cccUCUUGUC--UCUUGUCCUCu-5' miRNA<br>16 15 14 13 7 6 5 4 3 2<br>3'pairing Seed</td><td>ACAGGA<br>Offset 6mer</td><td></td><td></td><td>M</td></tr>
</table>

<table>
<tr><th>2D Structure</th><th>Local AU</th><th>Position</th><th>Conservation</th><th>Predicted By</th></tr>
<tr><td>Offset<br>6mer<br>408 428<br>5'-gcaAGTACCGTGAACAGGAag-3' UTR<br>|| || | ||||||||<br>3'-cccUCUUGUCUCUUGUCCUCu-5' miRNA<br>16 15 14 13 7 6 5 4 3 2<br>3'pairing Seed</td><td>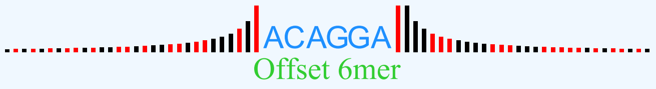</td><td>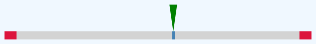</td><td></td><td>M</td></tr>
<tr><td>621 7mer-m8 641<br>5'-agtATATCAGTCAACAGGAGt-3' UTR<br>| | ||| ||||||||<br>3'-cccUCUUGUCUCUUGUCCUCu-5' miRNA<br>16 15 14 13 7 6 5 4 3 2<br>3'pairing Seed</td><td>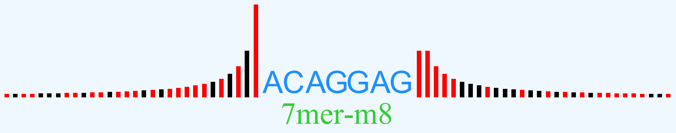</td><td>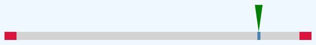</td><td></td><td>M T</td></tr>
</table>

| 2D Structure | Local AU | Position | Conservation | Predicted By |
|---|---|---|---|---|
| Imperfect match<br>131 151<br>5'-ttacGAATTCCA-TTCATCTTg-3' UTR<br>3'-gcgaCGUGAGGUGGAGUAGGAc-5' miRNA<br>16 15 14 13 7 6 5 4 3 2<br>3'pairing Seed | 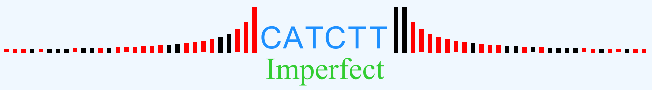 | 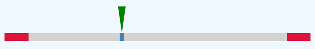 | | M |
| Imperfect match<br>160 181<br>5'-caaTGCAGTCCCCTTCATCATc-3' UTR<br>3'-gcgACGUGAGGUGGAGUAGGAc-5' miRNA<br>16 15 14 13 7 6 5 4 3 2<br>3'pairing Seed | 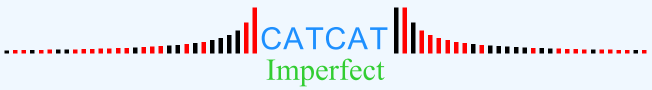 | 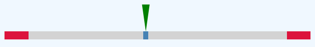 | | M |

<table>
<tr><th>2D Structure</th><th>Local AU</th><th>Position</th><th>Conservation</th><th>Predicted By</th></tr>
<tr><td>Imperfect match<br>131 5'-ttacGAATTCCA-TTCATCTTg-3' 151 UTR<br>|  |:||||  :|||||:|<br>3'-gcgaCGUGAGGUGGAGUAGGAc-5' miRNA<br>16 15 14 13 7 6 5 4 3 2<br>3'pairing Seed</td><td>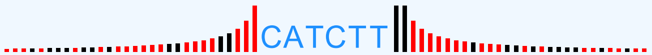<br>CATCTT<br>Imperfect</td><td>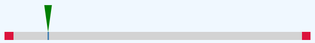</td><td></td><td>M</td></tr>
<tr><td>Imperfect match<br>160 5'-caaTGCAGTCCCCTTCATCATc-3' 181 UTR<br>||||  |||  |:|||||  |<br>3'-gcgACGUGAGGUGGAGUAGGAc-5' miRNA<br>16 15 14 13 7 6 5 4 3 2<br>3'pairing Seed</td><td>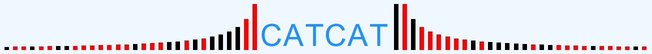<br>CATCAT<br>Imperfect</td><td>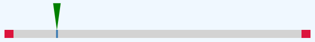</td><td></td><td>M</td></tr>
<tr><td>Imperfect match<br>337 5'-taCTTTACTATGCCTTATCTTc-3' 358 UTR<br>||  :|||  ::|||:|||:|<br>3'-gcGACGUGAGGUGGAGUAGGAc-5' miRNA<br>16 15 14 13 7 6 5 4 3 2<br>3'pairing Seed</td><td>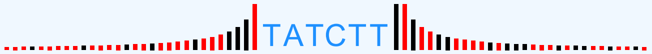<br>TATCTT<br>Imperfect</td><td>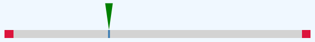</td><td></td><td>M</td></tr>
</table>

| 2D Structure | Local AU | Position | Conservation | Predicted By |
| --- | --- | --- | --- | --- |
| Offset 6mer<br>158 180<br>5'-catTCCCTACTTCATGCCCGct-3' UTR<br>3'-cacAUGUGAUG--GUACGGGCCa-5' miRNA<br>16 15 14 13 7 6 5 4 3 2<br>3'pairing Seed | 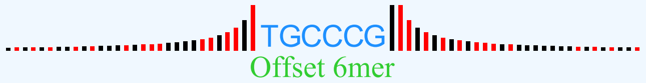 | 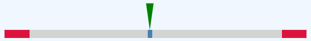 | | M |
| 7mer-m8<br>181 200<br>5'-tctctCAGTA-TGTGCCCGGg-3' UTR<br>3'-cacauGUGAUGGUACGGGCCa-5' miRNA<br>16 15 14 13 7 6 5 4 3 2<br>3'pairing Seed | 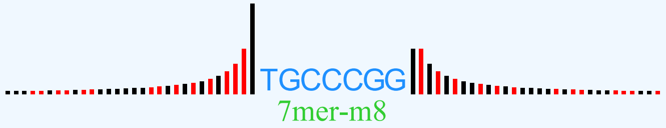 | 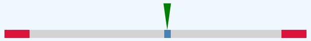 | | M T |

<table>
<tr><th>2D Structure</th><th>Local AU</th><th>Position</th><th>Conservation</th><th>Predicted By</th></tr>
<tr><td>Imperfect match<br>77 100<br>5'-ctggTAGCTCTGGTGGCTCTCTTg-3' UTR<br>|  ||:|:   ||:|||||:|<br>3'-gaugAACGGGG--ACUGAGAGGAc-5' miRNA<br>16 15 14 13 7 6 5 4 3 2<br>3'pairing Seed</td><td>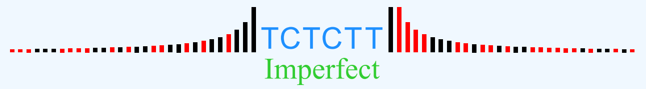</td><td>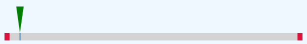</td><td>☐</td><td>M</td></tr>
<tr><td>6mer<br>452 475<br>5'-tcttcTGCTCCTGAGATTCTCCTg-3' UTR<br>|||:|||  ||:||||||<br>3'-gaugaACGGGGA--CUGAGAGGAc-5' miRNA<br>16 15 14 13 7 6 5 4 3 2<br>3'pairing Seed</td><td>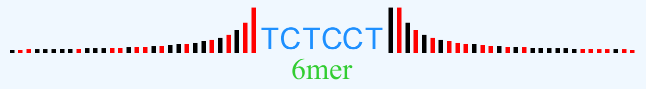</td><td>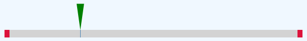</td><td>☐</td><td>M</td></tr>
<tr><td>7mer-m8<br>682 704<br>5'-aatCTTGTCTGTCAGCTCTCCTg-3' UTR<br>||||:|:  |  |  |||||||<br>3'-gauGAACGGGGACU-GAGAGGAc-5' miRNA<br>16 15 14 13 7 6 5 4 3 2<br>3'pairing Seed</td><td>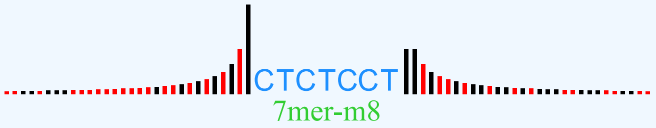</td><td>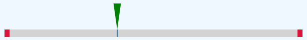</td><td>☐</td><td>M T</td></tr>
</table>

<table>
<tr><th>2D Structure</th><th>Local AU</th><th>Position</th><th>Conservation</th><th>Predicted By</th></tr>
<tr><td>227 6mer 246<br>5'-gaaCCAGGCTGCTTCTCCTc-3' UTR<br>|| :||| |:||||||<br>3'-cugGGAUCGAGGGAGAGGAg-5' miRNA<br>16 15 14 13 7 6 5 4 3 2<br>3'pairing Seed</td><td>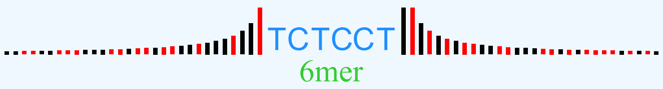<br></td><td>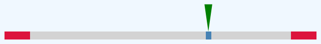</td><td></td><td>M</td></tr>
<tr><td>Imperfect match<br>236 255<br>5'-tgcttctcCTCTCTCTCTTc -3' UTR<br>|||:|||||:|<br>3'-cugggaucGAGGGAGAGGAg-5' miRNA<br>16 15 14 13 7 6 5 4 3 2<br>3'pairing Seed</td><td>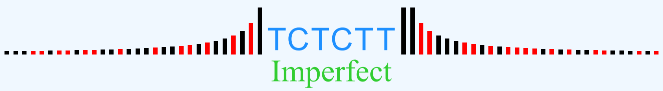<br></td><td>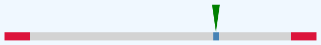</td><td></td><td>M</td></tr>
</table>
